# Supplementary material for: naRNA-LL37 composite DAMPs define sterile NETs as self-propagating drivers of inflammation
Source: EMBO Rep. 2024 May 23;25(7):10. doi: 10.1038/s44319-024-00150-5 (PMC11239898; doi:10.1038/s44319-024-00150-5)
Supplement: Supplementary file 1 — Appendix [file 44319_2024_150_MOESM1_ESM.pdf]

<u>Appendix</u>

naRNA-LL37 composite DAMPs define sterile NETs as self-propagating drivers of inflammation

Authors

Francesca Bork[1], Carsten L. Greve[1], Christine Youn[2], Sirui Chen[1], Vinicius N. C. Leal[1,3], Yu Wang[2], Berenice Fischer[4], Masoud Nasri[5], Jule Focken[6], Jasmin Scheurer[6], Pujan Engels[1], Marissa Dubbelaar[7], Katharina Hipp[8], Baher Zalat[1], Andras Szolek[1], Meng-Jen Wu[2], Birgit Schittek[6,9,10], Stefanie Bugl[1], Thomas A. Kufer[11], Markus W. Löffler[7,9,12], Mathias Chamaillard[13], Julia Skokowa[5,9], Daniela Kramer[4], Nathan K. Archer[2], Alexander N.R. Weber[1,9,10]*

Contents

Appendix Figures S1: page 2

Appendix Figures S2: page 3

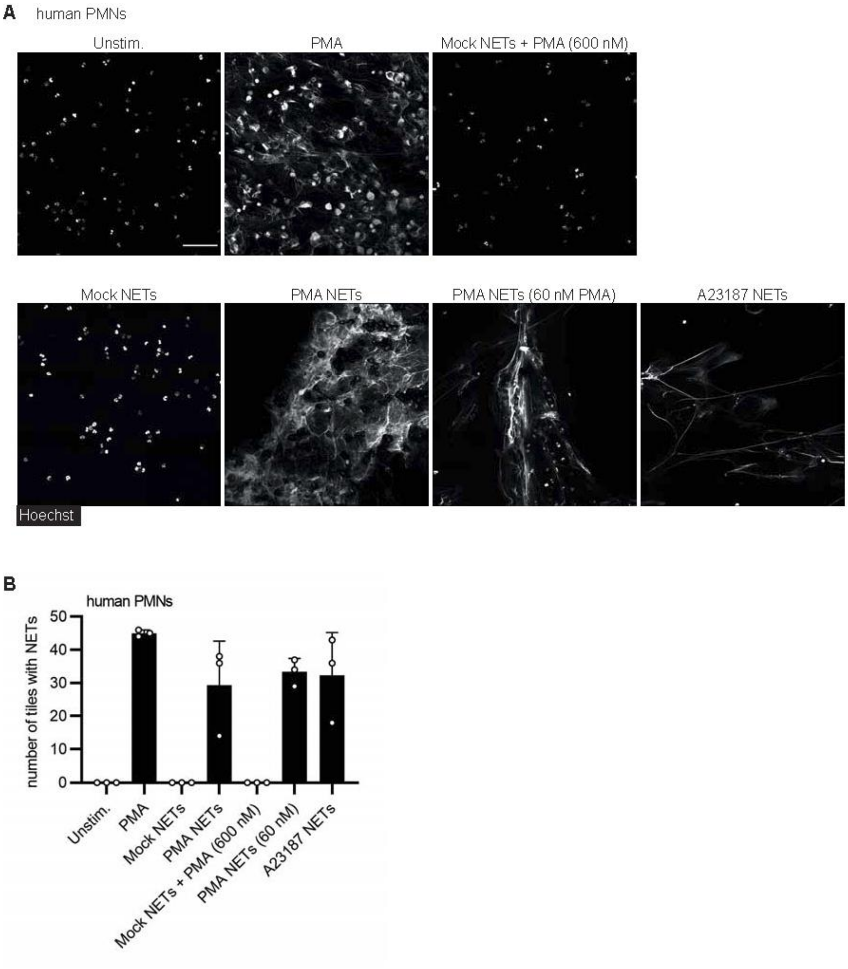

Appendix figure S1: Ruling out of PMA carry-over during PMA NET preparation and stimulatory activity of NETs prepared using low PMA concentrations and other stimuli.

(A) Confocal microscopy of primary human PMNs (first row from left to right) left unstimulated, or treated with PMA directly (600 nM), or treated with Mock NETs transiently exposed to 600 nM PMA, or (second row from left to right) treated with conventional Mock NETs (see Methods), or treated with conventional PMA NETs generated using 600 nM PMA, or treated with PMA NETs generated with 60 nM PMA, or treated with NETs generated with the ionophore A21187 (5 µM) for 3 h. All NET preparations were used at 1:100 dilution. After the 3 h, the cells were stained for DNA (Hoechst 33342, white, n= 1 donor, representative images, scale bar 10 µm).

(B) Quantification of A using DNA (Hoechst 33342) signal to quantify NET formation (n = 1 donor, mean+SD, each dot represents the number of NET-positive tiles in one image quantified from three images/condition).

Data information: In **B**, data are presented as mean + SD.

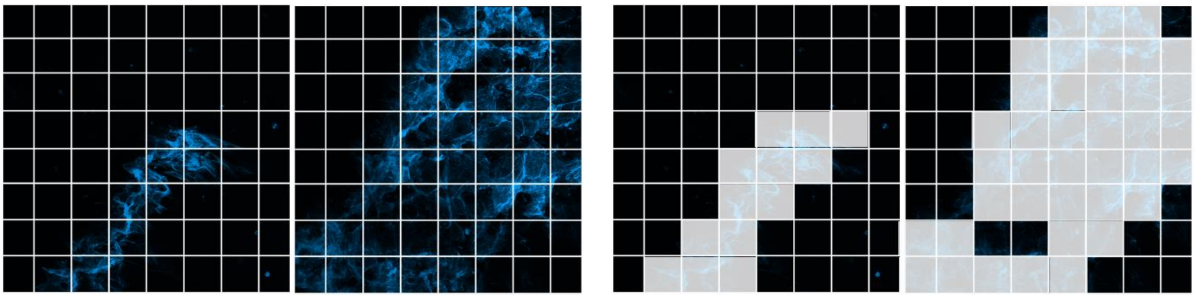

Appendix figure S2: Quantification of NETs via Hoechst signal.

Example of 8x8 tile counting for NETs. Left: original confocal image with DNA visualized by Hoechst staining. Right: scored tiles used for quantification labelled in white. As the selection of ROIs is indeed challenging due to the erratic shape of the NETs in vitro we harmonized quantification of in vitro results throughout as “tiles showing NETs”, i.e. showing diffuse DNA signal as in the example shown. In brief, an 8 by 8 grid was superimposed on each image (all images taken with the same settings, including objective, zoom and resolution) and then blinded but scored manually. A tile was counted as NET positive whenever a DNA (Hoechst) signal was clearly detected in shape of a fiber or mesh outside of a cell with a destroyed nucleus. A tile was counted as negative when only intact cells or no Hoechst signal were observed within this respective area.
