# Supplementary figures and images for: naRNA-LL37 composite DAMPs define sterile NETs as self-propagating drivers of inflammation (part 1 of 4)
Source: EMBO Rep. 2024 May 23;25(7):10. doi: 10.1038/s44319-024-00150-5 (PMC11239898; doi:10.1038/s44319-024-00150-5)

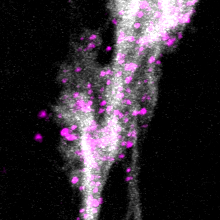

Supplement: Supplementary file 4 — Source data Fig. 1 [file 44319_2024_150_MOESM4_ESM.zip › Main Figure 1/Fig 1A/Images/bRNA-LL37/comp new gray crop.png]

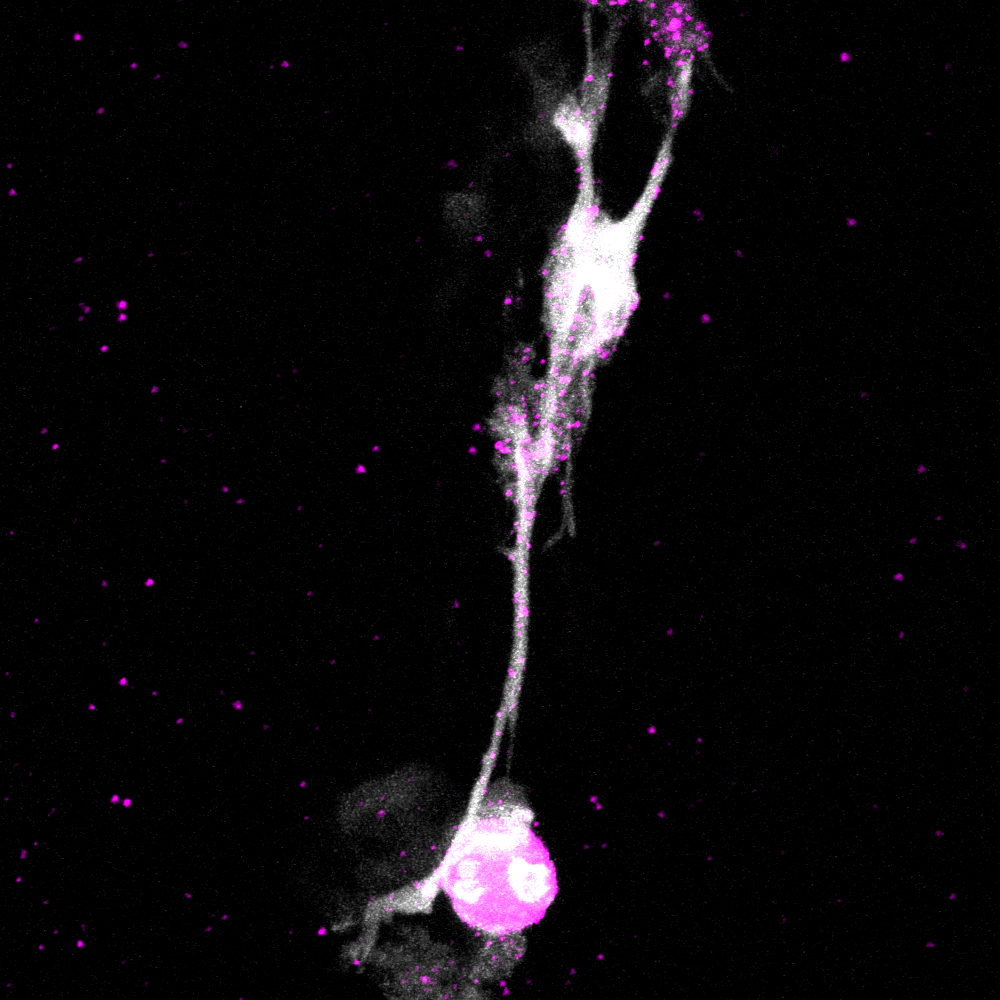

Supplement: Supplementary file 4 — Source data Fig. 1 [file 44319_2024_150_MOESM4_ESM.zip › Main Figure 1/Fig 1A/Images/bRNA-LL37/comp new gray.png]

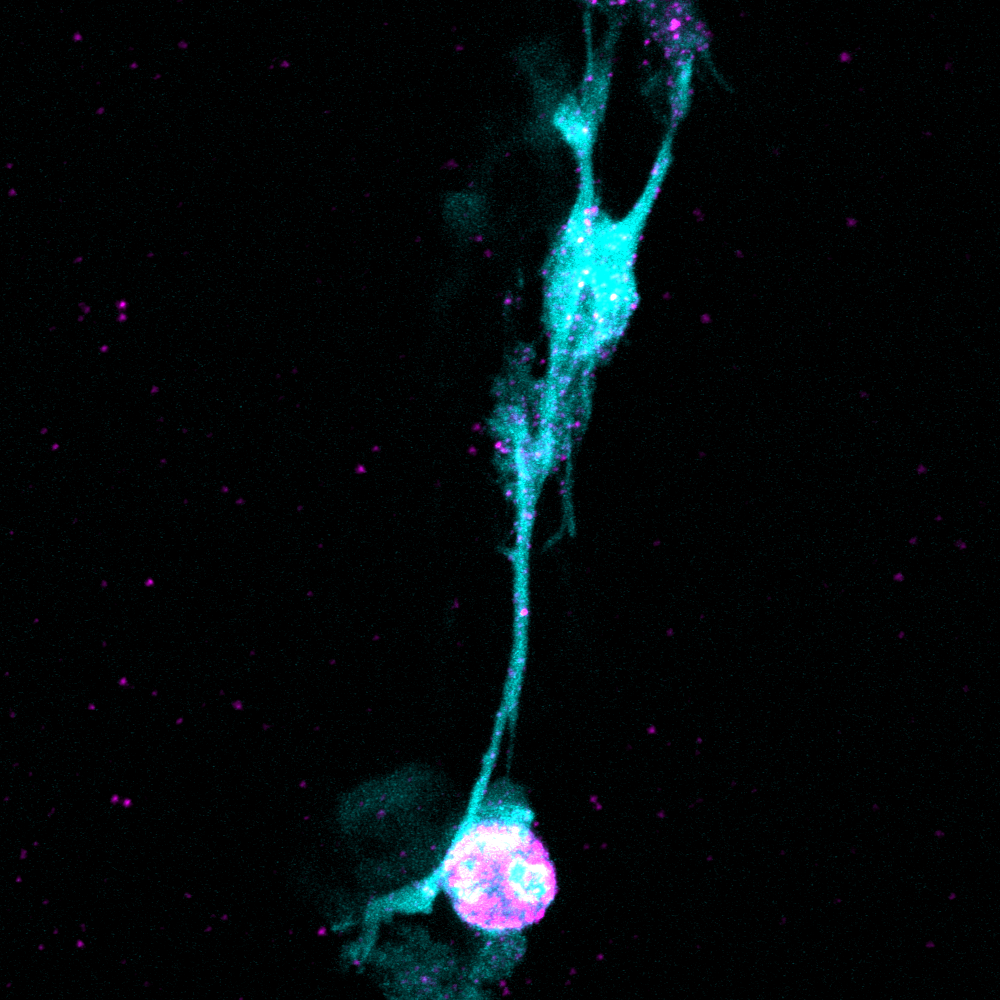

Supplement: Supplementary file 4 — Source data Fig. 1 [file 44319_2024_150_MOESM4_ESM.zip › Main Figure 1/Fig 1A/Images/bRNA-LL37/comp new.png]

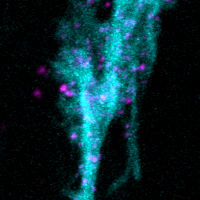

Supplement: Supplementary file 4 — Source data Fig. 1 [file 44319_2024_150_MOESM4_ESM.zip › Main Figure 1/Fig 1A/Images/bRNA-LL37/crop new.png]

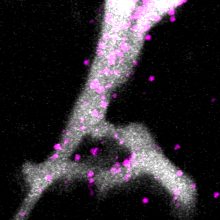

Supplement: Supplementary file 4 — Source data Fig. 1 [file 44319_2024_150_MOESM4_ESM.zip › Main Figure 1/Fig 1A/Images/fRNA-LL37/comp new gray crop.png]

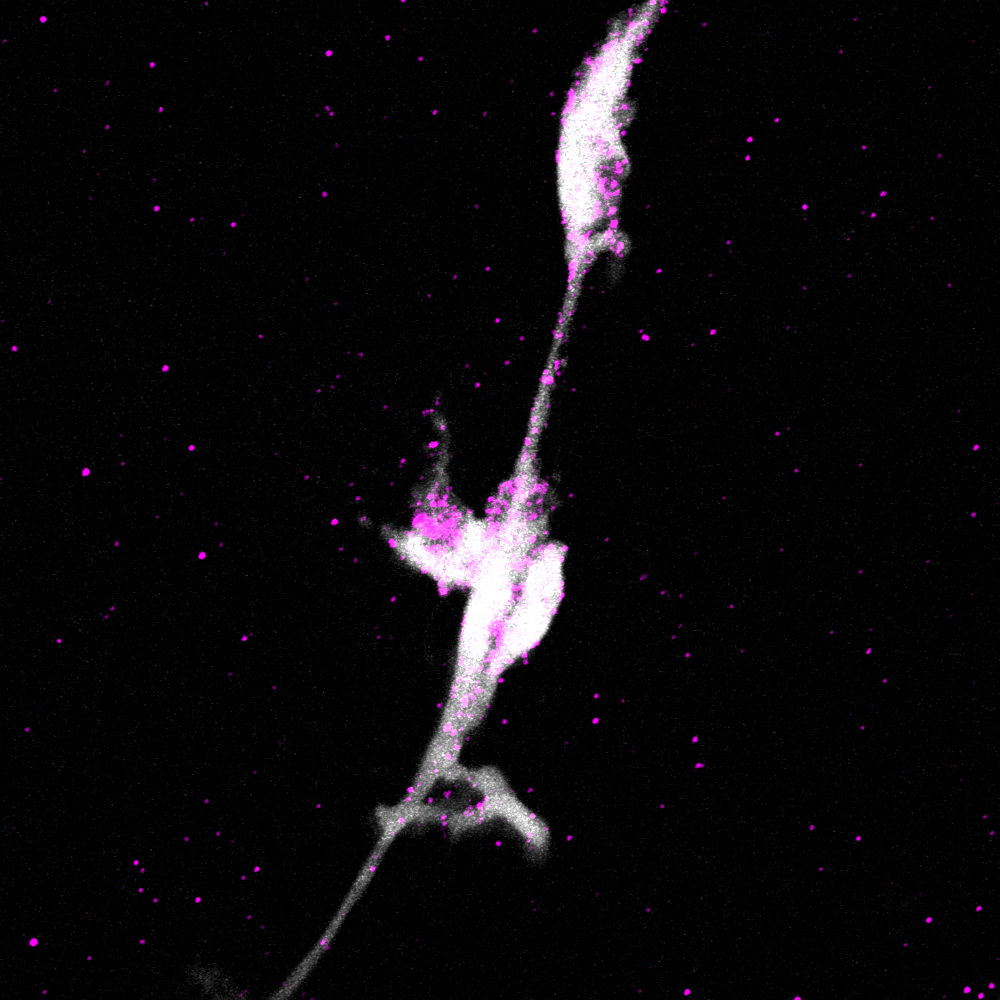

Supplement: Supplementary file 4 — Source data Fig. 1 [file 44319_2024_150_MOESM4_ESM.zip › Main Figure 1/Fig 1A/Images/fRNA-LL37/comp new gray.png]

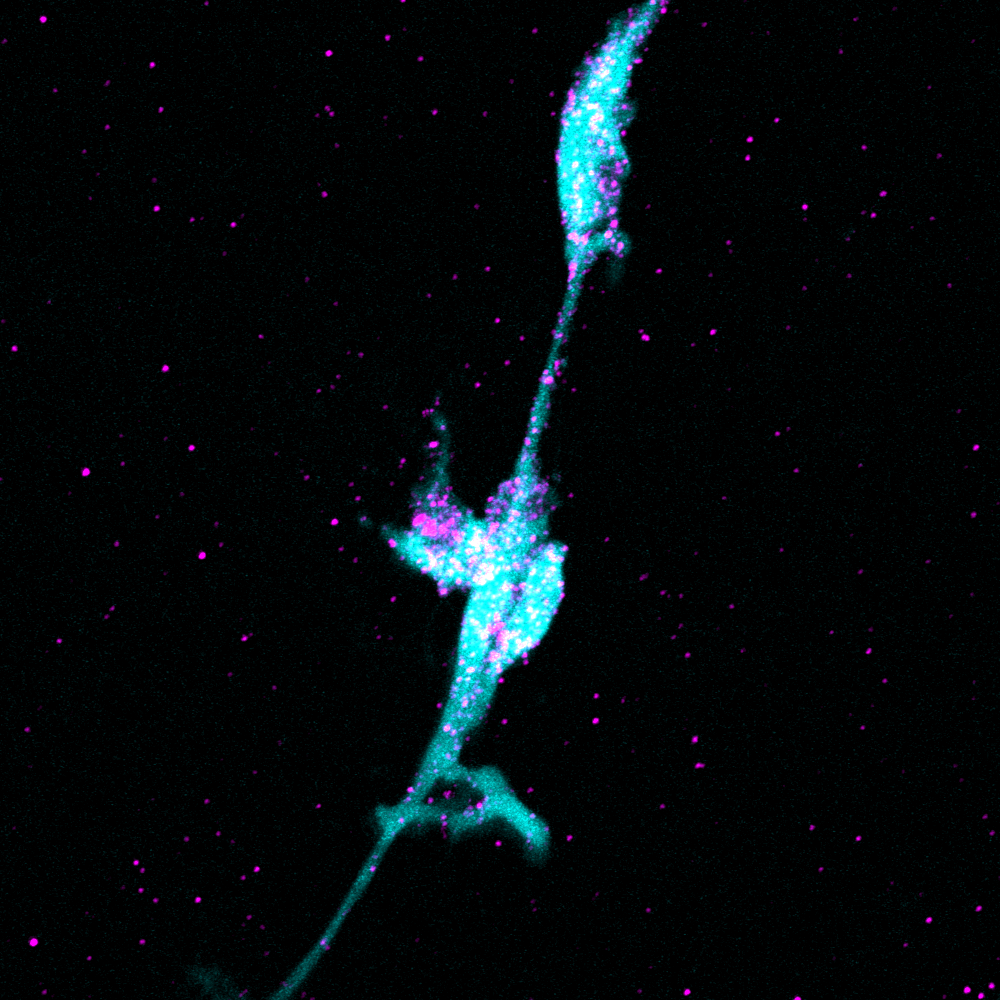

Supplement: Supplementary file 4 — Source data Fig. 1 [file 44319_2024_150_MOESM4_ESM.zip › Main Figure 1/Fig 1A/Images/fRNA-LL37/comp new.png]

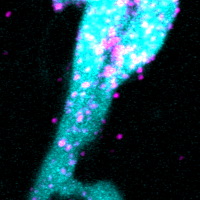

Supplement: Supplementary file 4 — Source data Fig. 1 [file 44319_2024_150_MOESM4_ESM.zip › Main Figure 1/Fig 1A/Images/fRNA-LL37/crop ne.png]

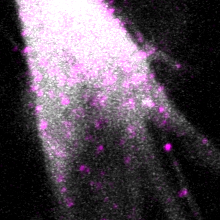

Supplement: Supplementary file 4 — Source data Fig. 1 [file 44319_2024_150_MOESM4_ESM.zip › Main Figure 1/Fig 1A/Images/live C. albicans/comp new gray crop.png]

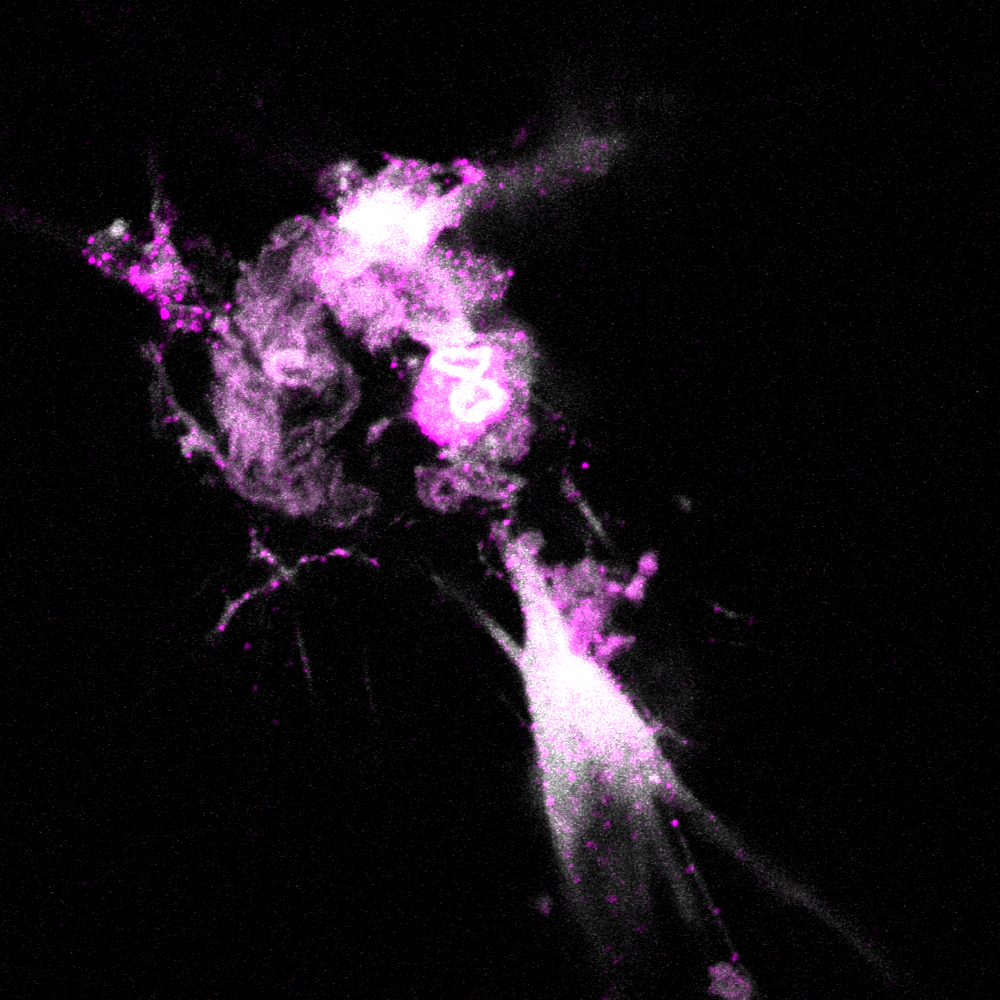

Supplement: Supplementary file 4 — Source data Fig. 1 [file 44319_2024_150_MOESM4_ESM.zip › Main Figure 1/Fig 1A/Images/live C. albicans/comp new gray.png]

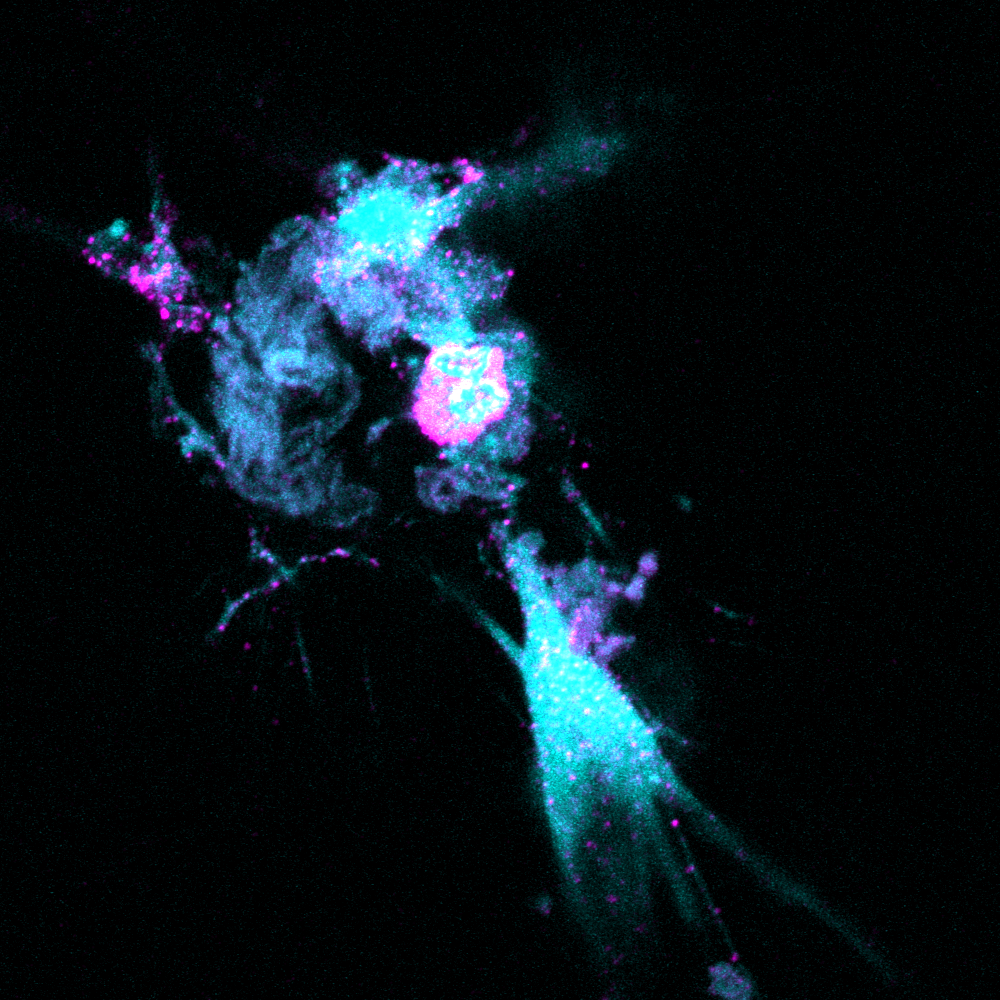

Supplement: Supplementary file 4 — Source data Fig. 1 [file 44319_2024_150_MOESM4_ESM.zip › Main Figure 1/Fig 1A/Images/live C. albicans/comp new.png]

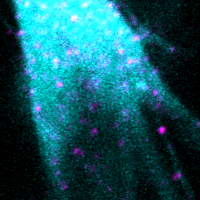

Supplement: Supplementary file 4 — Source data Fig. 1 [file 44319_2024_150_MOESM4_ESM.zip › Main Figure 1/Fig 1A/Images/live C. albicans/crop new.png]

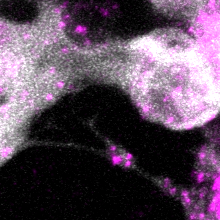

Supplement: Supplementary file 4 — Source data Fig. 1 [file 44319_2024_150_MOESM4_ESM.zip › Main Figure 1/Fig 1A/Images/Nigericin/comp new gray crop.png]

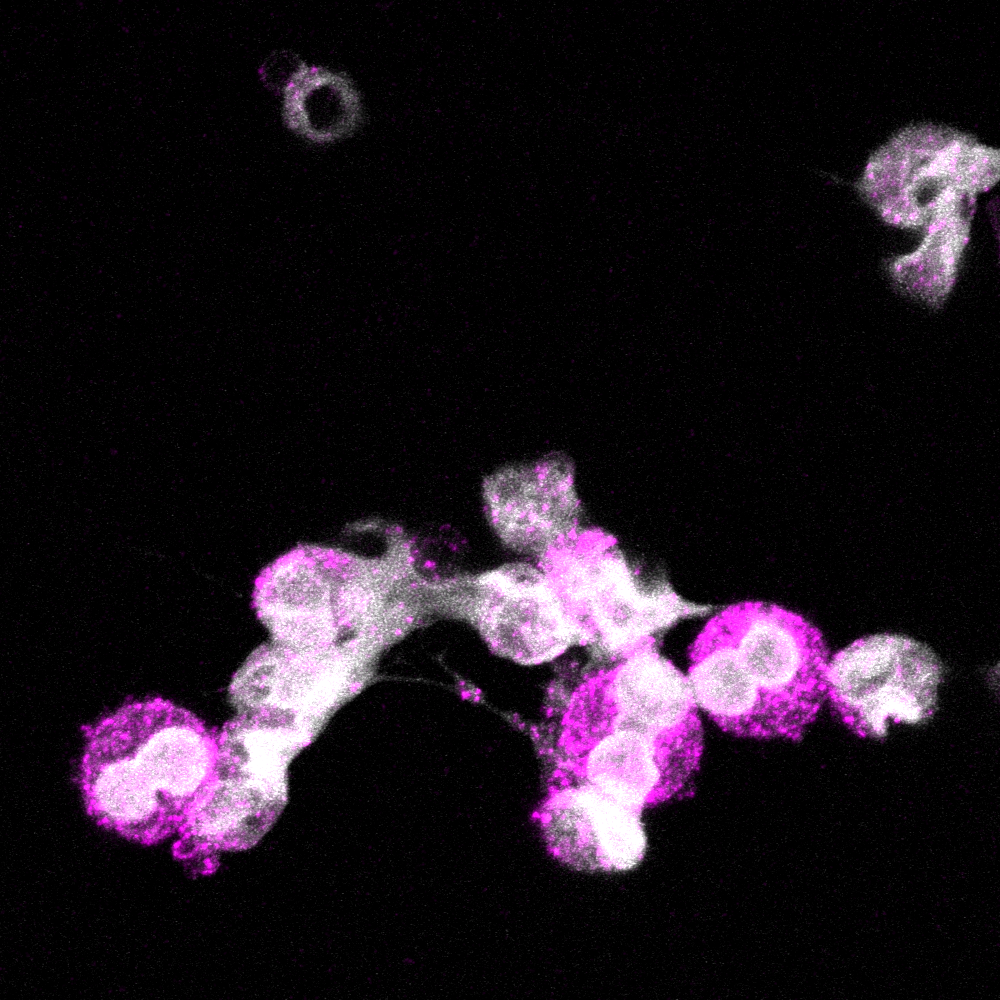

Supplement: Supplementary file 4 — Source data Fig. 1 [file 44319_2024_150_MOESM4_ESM.zip › Main Figure 1/Fig 1A/Images/Nigericin/comp new gray.png]

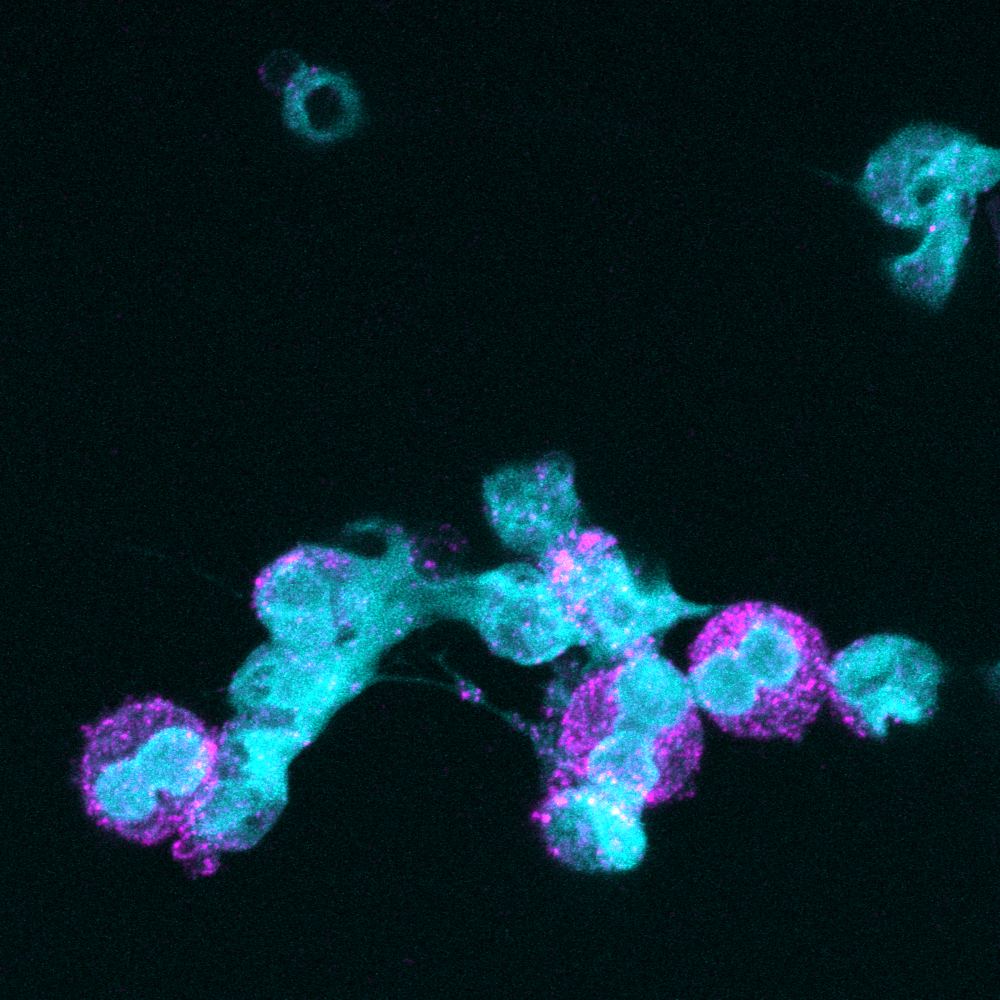

Supplement: Supplementary file 4 — Source data Fig. 1 [file 44319_2024_150_MOESM4_ESM.zip › Main Figure 1/Fig 1A/Images/Nigericin/comp new.png]

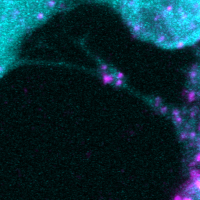

Supplement: Supplementary file 4 — Source data Fig. 1 [file 44319_2024_150_MOESM4_ESM.zip › Main Figure 1/Fig 1A/Images/Nigericin/crop new.png]

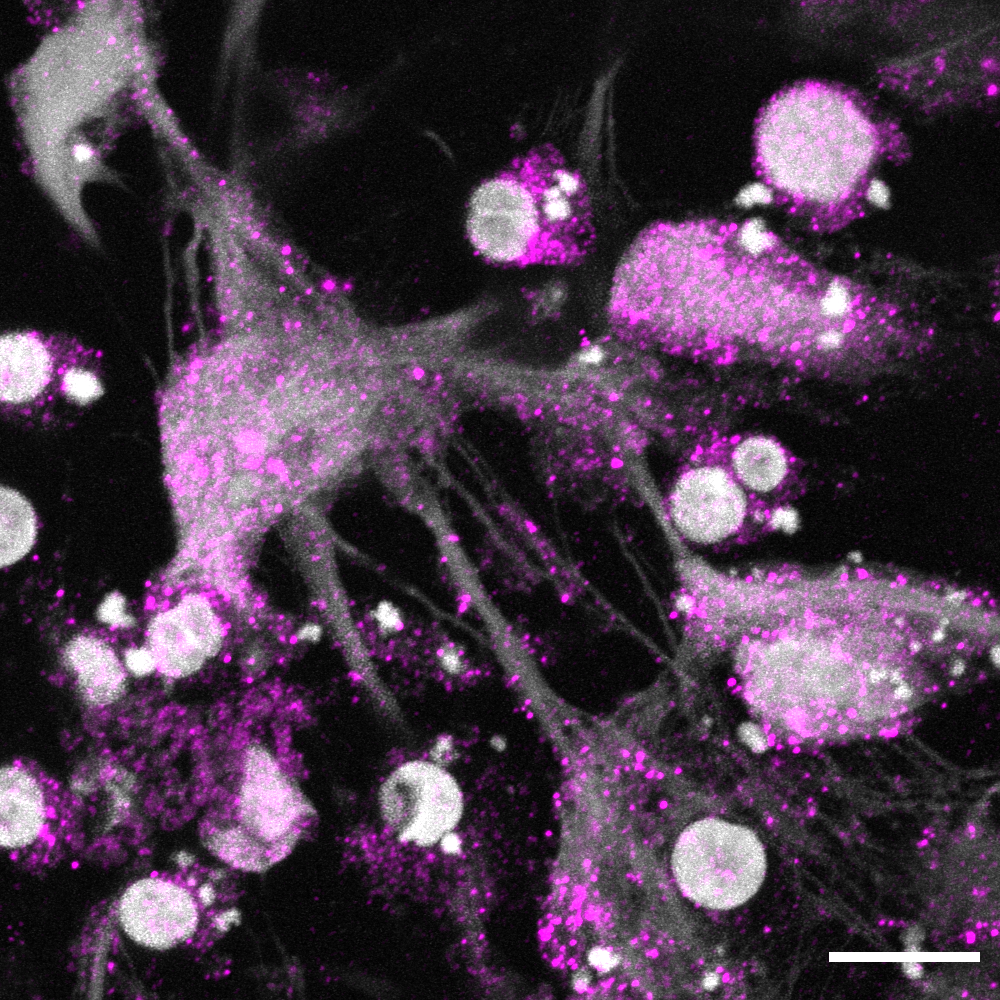

Supplement: Supplementary file 4 — Source data Fig. 1 [file 44319_2024_150_MOESM4_ESM.zip › Main Figure 1/Fig 1A/Images/PMA/comp scale bar new gray.png]

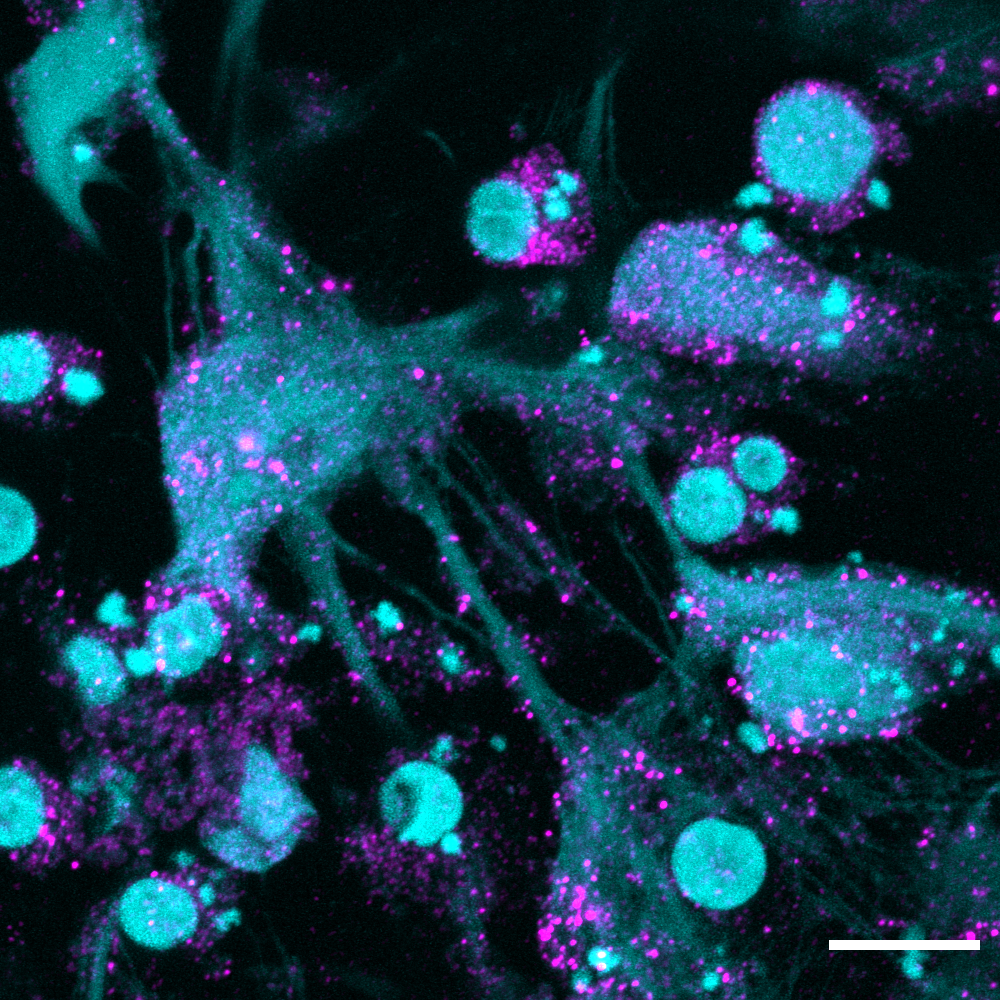

Supplement: Supplementary file 4 — Source data Fig. 1 [file 44319_2024_150_MOESM4_ESM.zip › Main Figure 1/Fig 1A/Images/PMA/comp scale bar new.png]

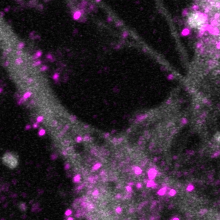

Supplement: Supplementary file 4 — Source data Fig. 1 [file 44319_2024_150_MOESM4_ESM.zip › Main Figure 1/Fig 1A/Images/PMA/crop new gray.png]

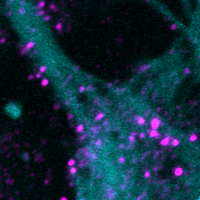

Supplement: Supplementary file 4 — Source data Fig. 1 [file 44319_2024_150_MOESM4_ESM.zip › Main Figure 1/Fig 1A/Images/PMA/crop new.png]

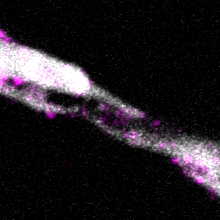

Supplement: Supplementary file 4 — Source data Fig. 1 [file 44319_2024_150_MOESM4_ESM.zip › Main Figure 1/Fig 1A/Images/ssRNA-LL37/comp gray crop.png]

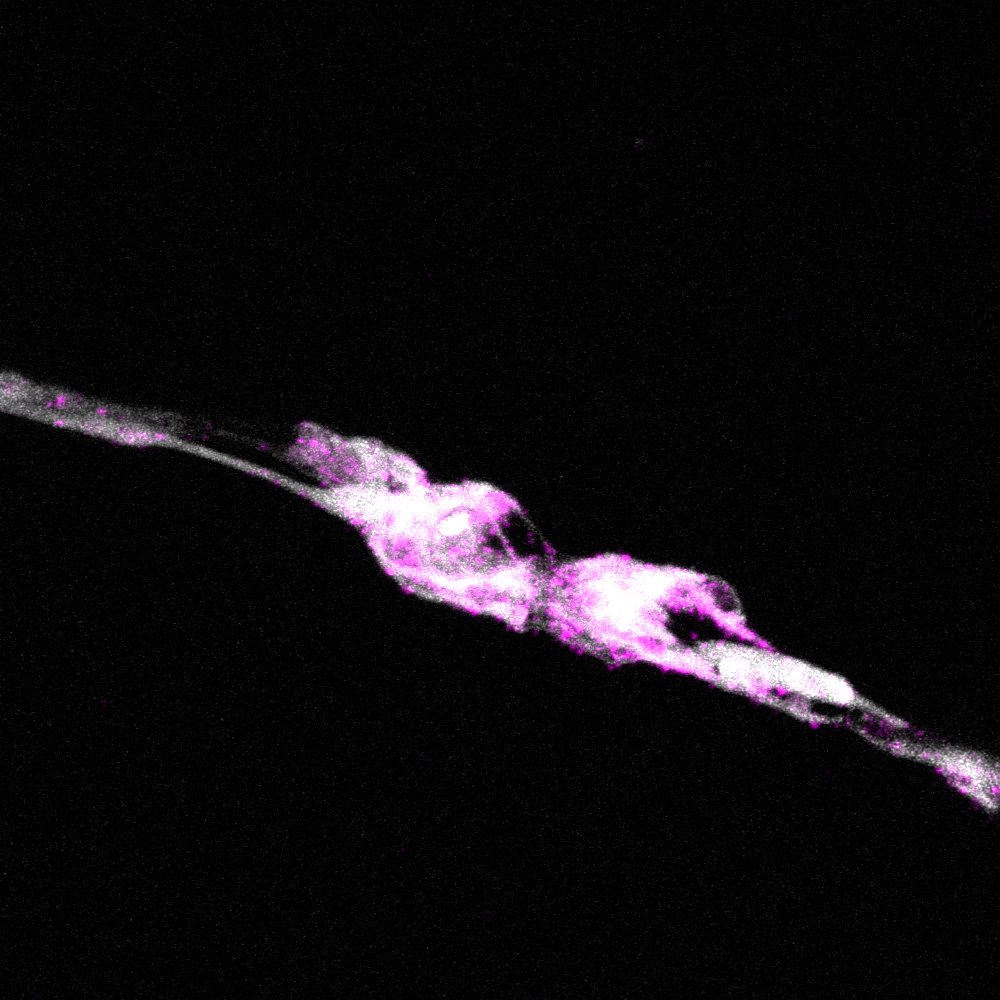

Supplement: Supplementary file 4 — Source data Fig. 1 [file 44319_2024_150_MOESM4_ESM.zip › Main Figure 1/Fig 1A/Images/ssRNA-LL37/comp gray.png]

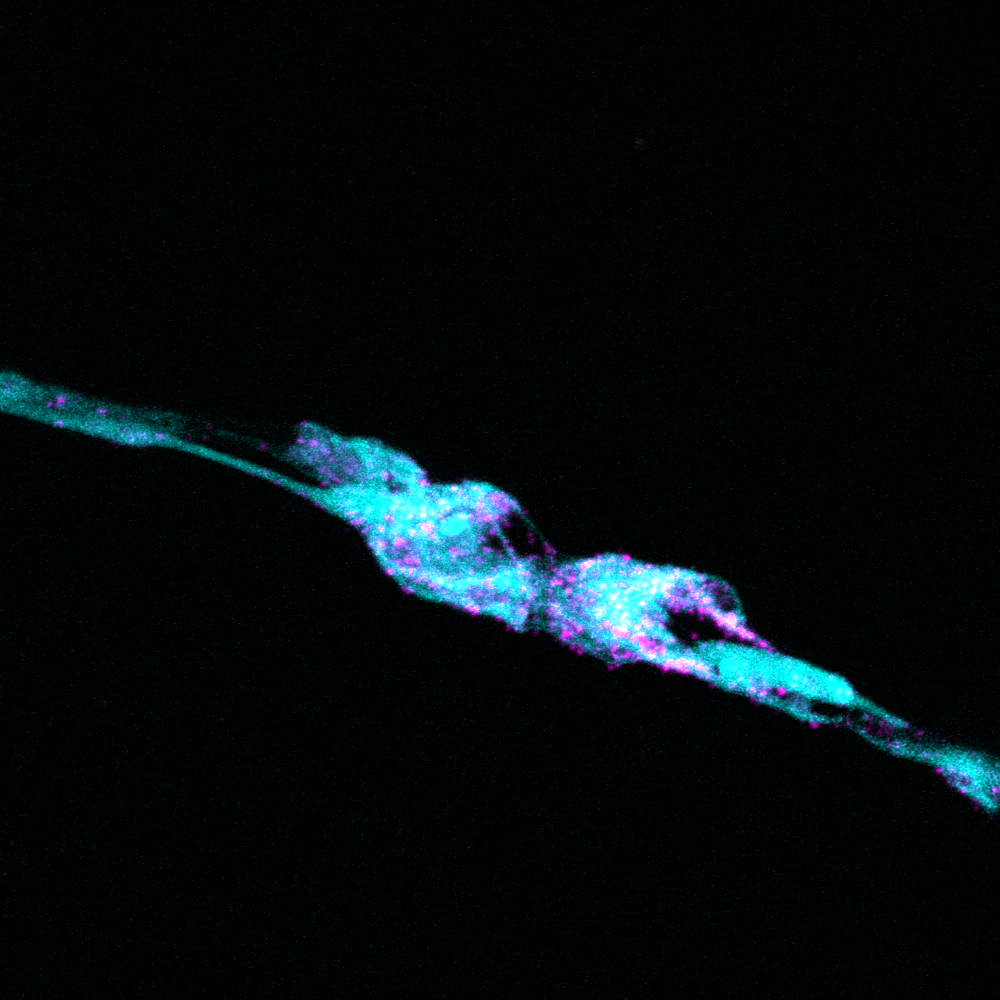

Supplement: Supplementary file 4 — Source data Fig. 1 [file 44319_2024_150_MOESM4_ESM.zip › Main Figure 1/Fig 1A/Images/ssRNA-LL37/comp new.png]

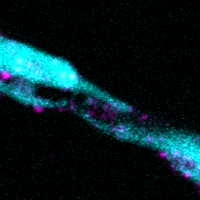

Supplement: Supplementary file 4 — Source data Fig. 1 [file 44319_2024_150_MOESM4_ESM.zip › Main Figure 1/Fig 1A/Images/ssRNA-LL37/crop new.png]

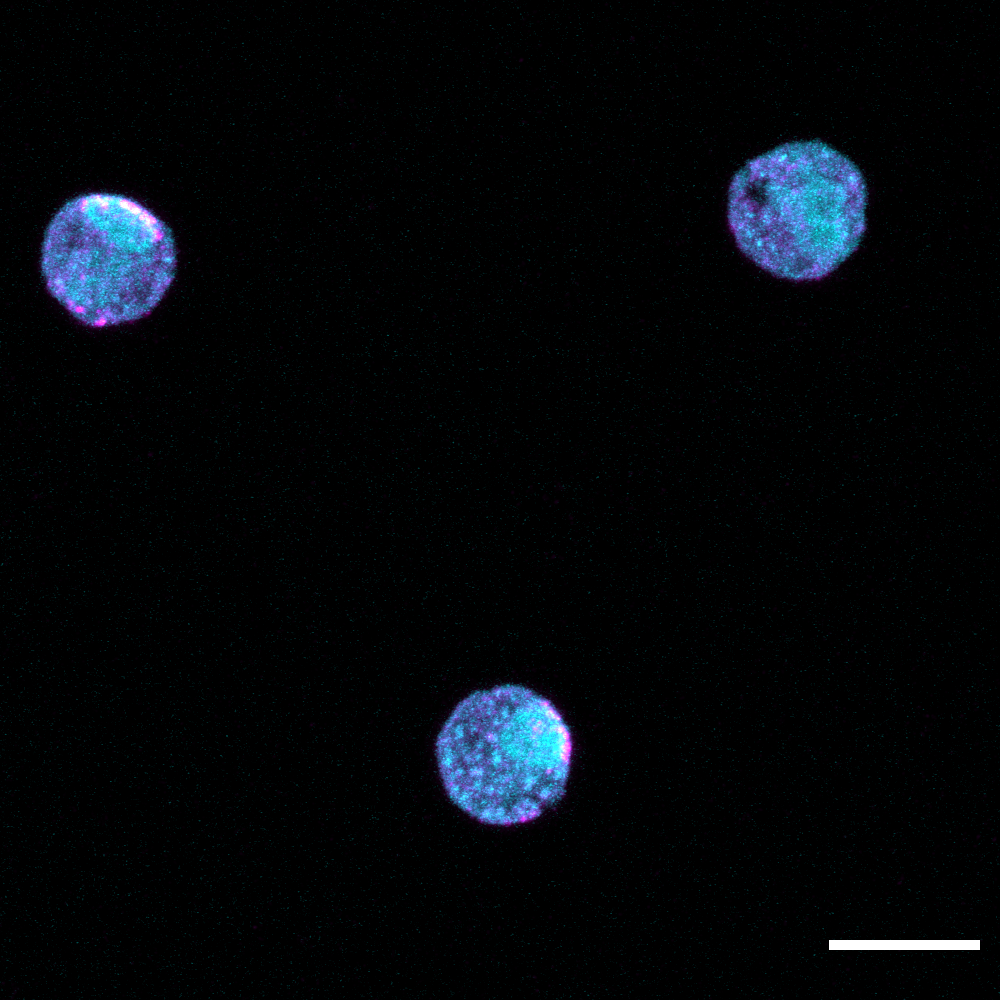

Supplement: Supplementary file 4 — Source data Fig. 1 [file 44319_2024_150_MOESM4_ESM.zip › Main Figure 1/Fig 1A/Images/unstimulated/comp new.png]

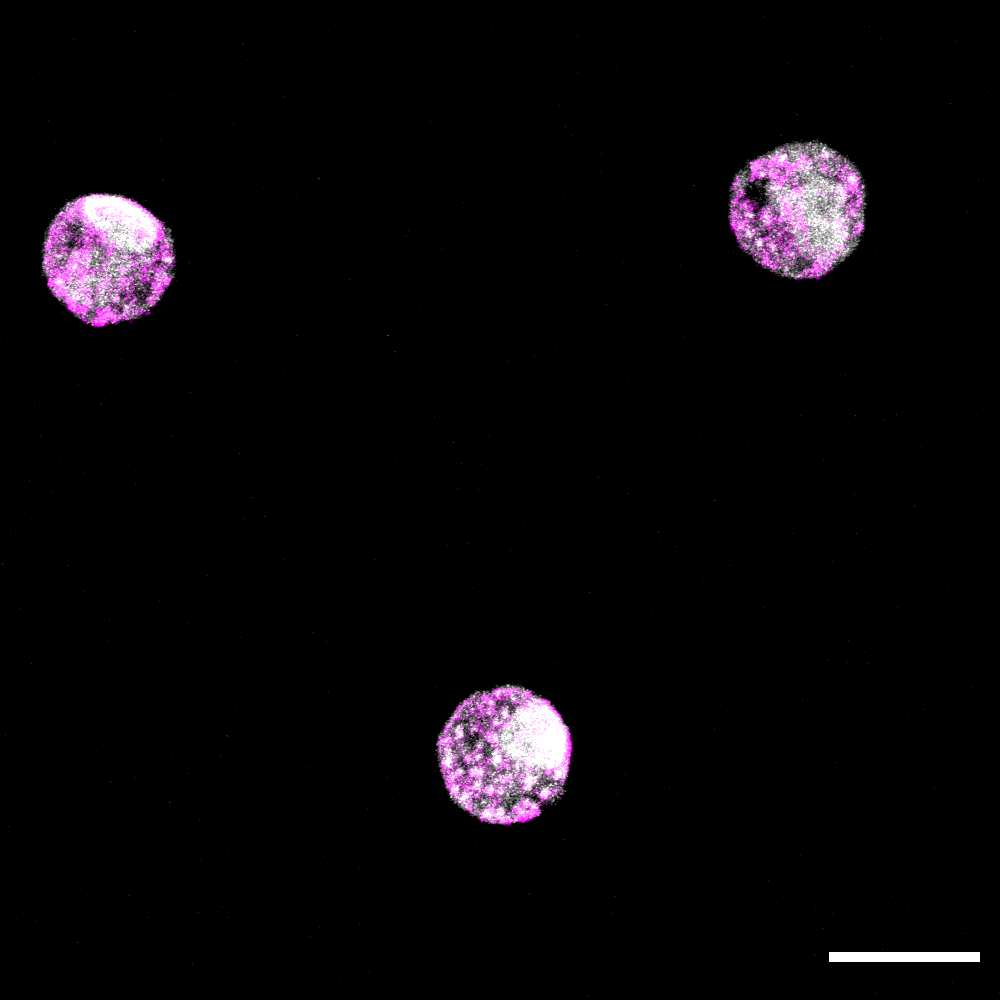

Supplement: Supplementary file 4 — Source data Fig. 1 [file 44319_2024_150_MOESM4_ESM.zip › Main Figure 1/Fig 1A/Images/unstimulated/gray new.png]

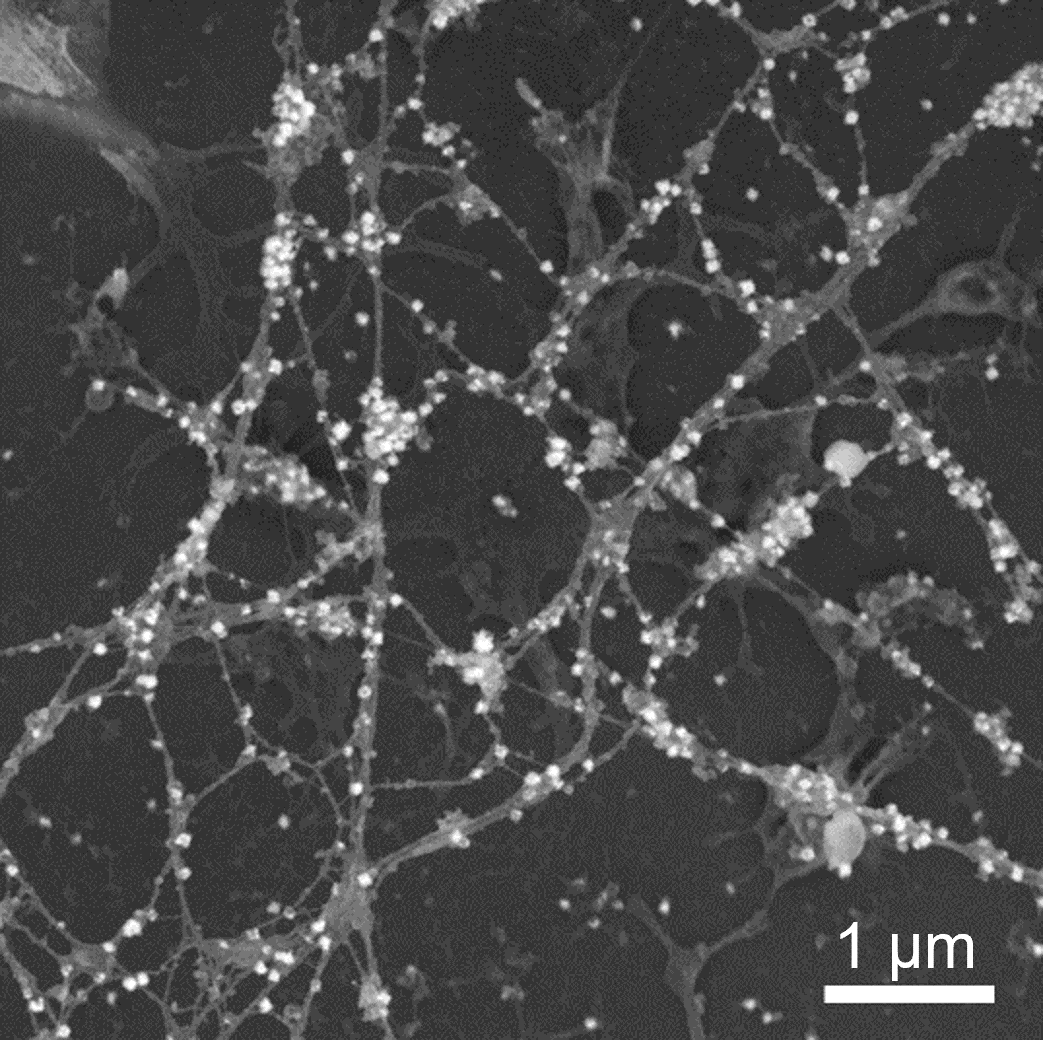

Supplement: Supplementary file 4 — Source data Fig. 1 [file 44319_2024_150_MOESM4_ESM.zip › Main Figure 1/Fig 1C/EM/PMA high zoom.png]

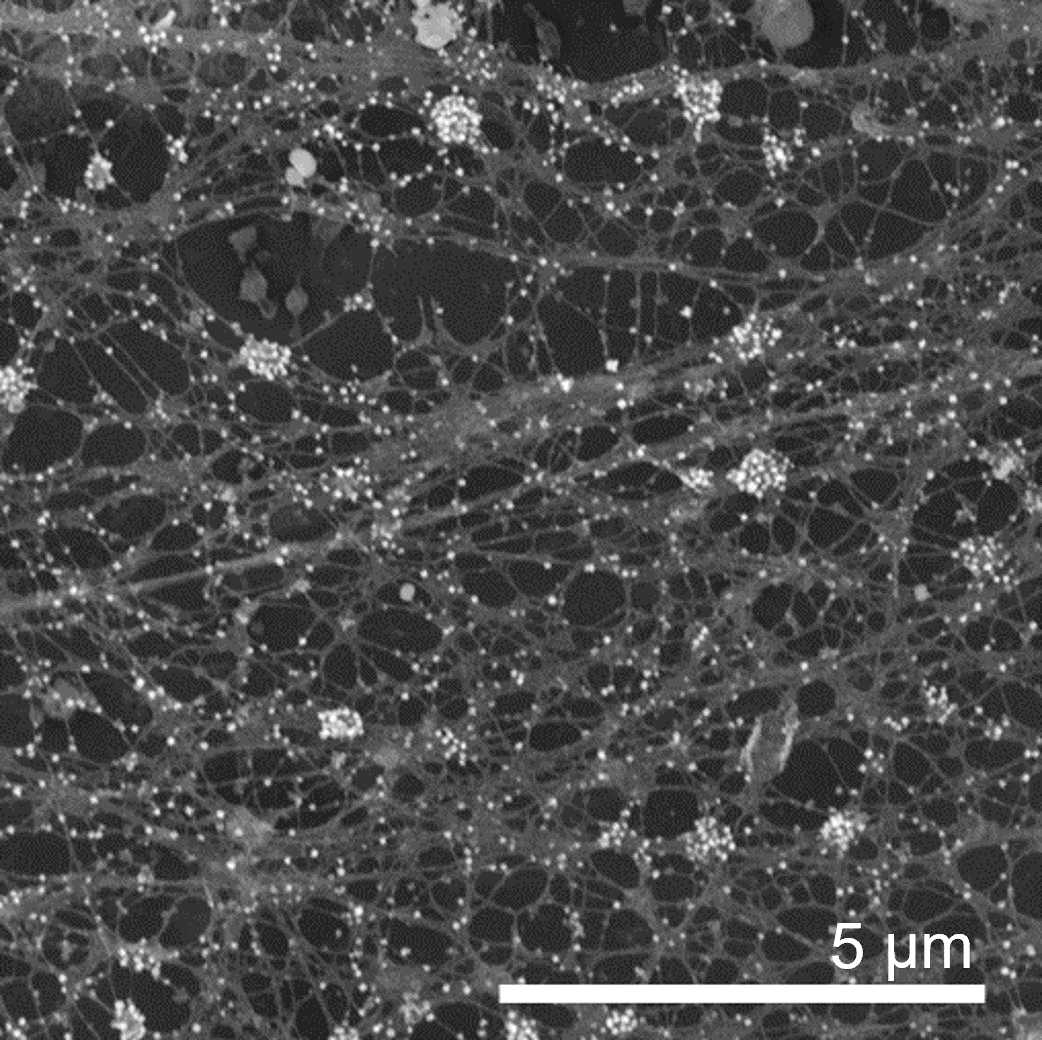

Supplement: Supplementary file 4 — Source data Fig. 1 [file 44319_2024_150_MOESM4_ESM.zip › Main Figure 1/Fig 1C/EM/PMA zoom.png]

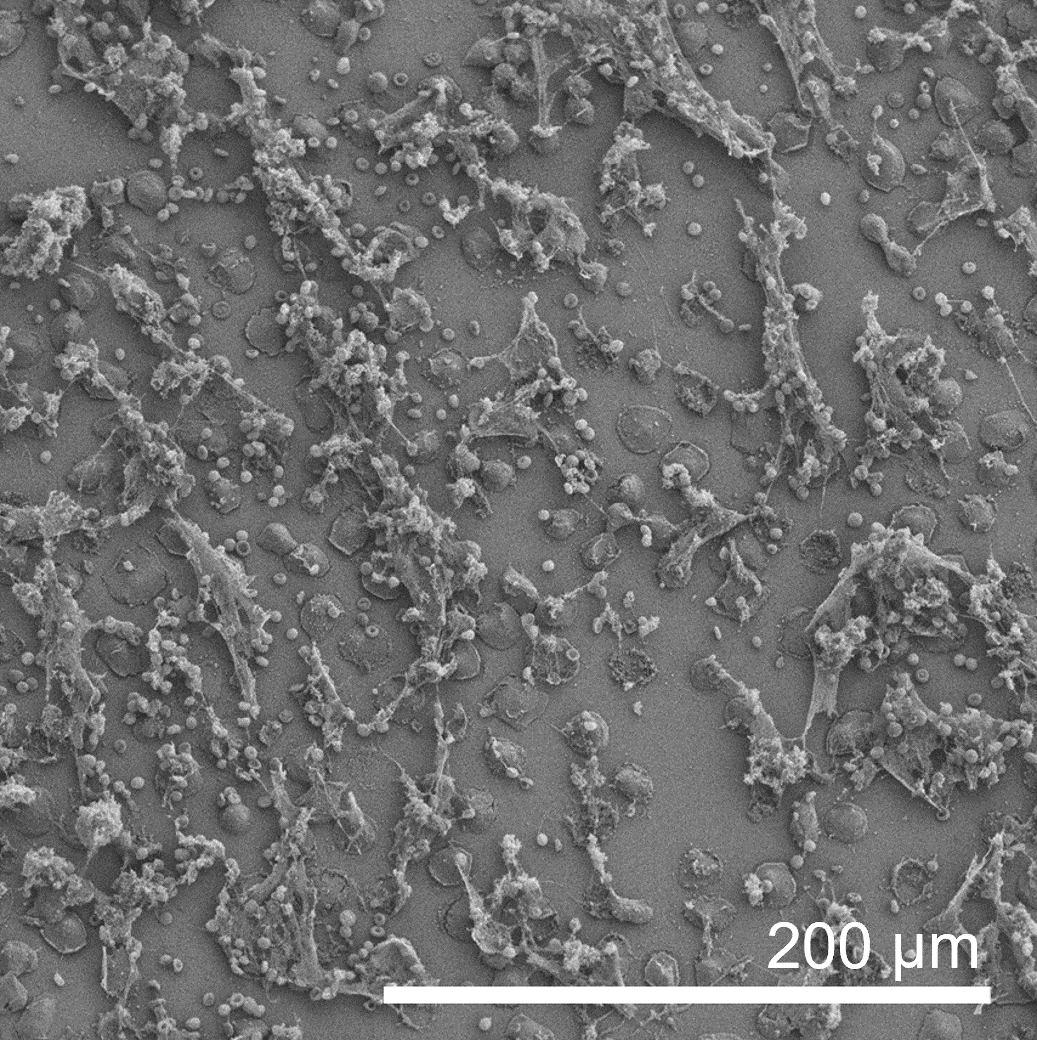

Supplement: Supplementary file 4 — Source data Fig. 1 [file 44319_2024_150_MOESM4_ESM.zip › Main Figure 1/Fig 1C/EM/PMA.png]

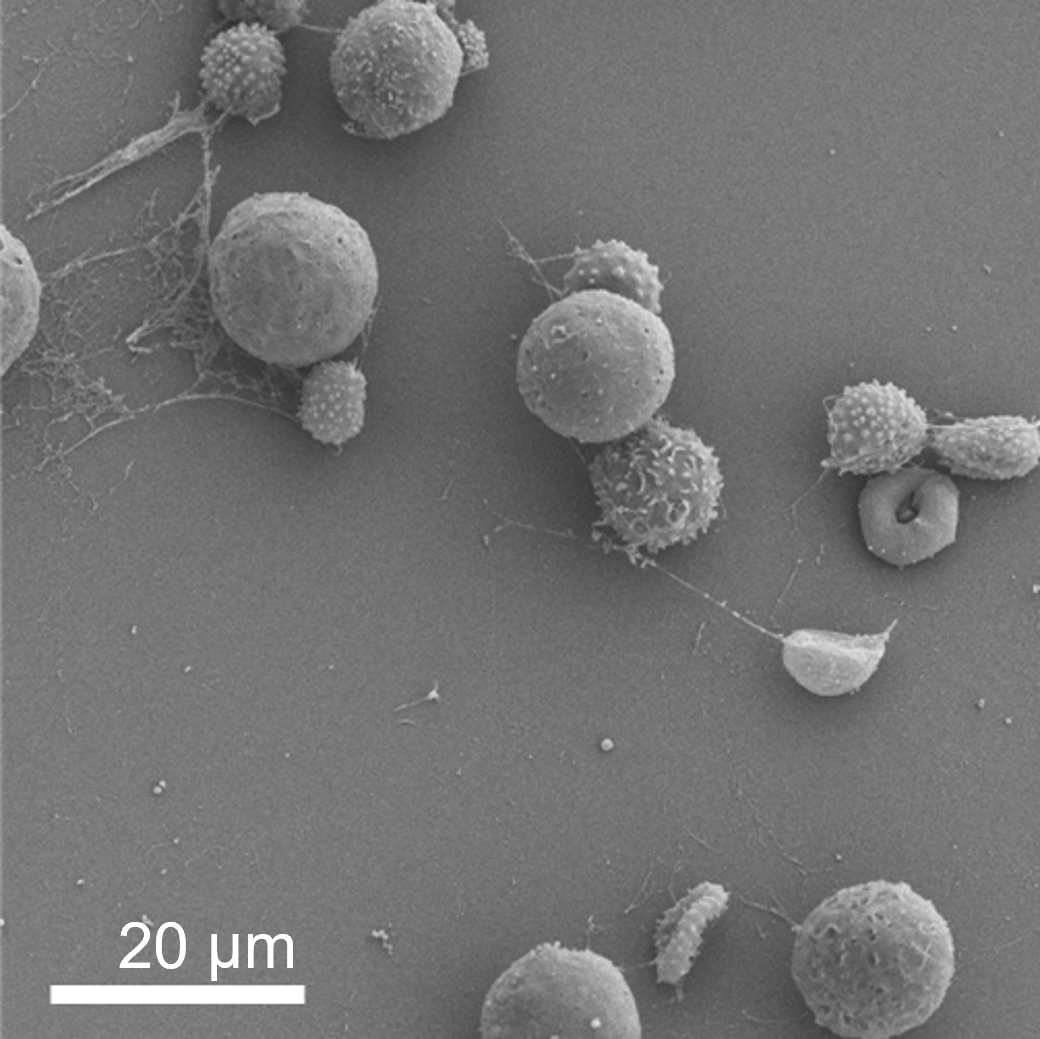

Supplement: Supplementary file 4 — Source data Fig. 1 [file 44319_2024_150_MOESM4_ESM.zip › Main Figure 1/Fig 1C/EM/unstim zoom.png]

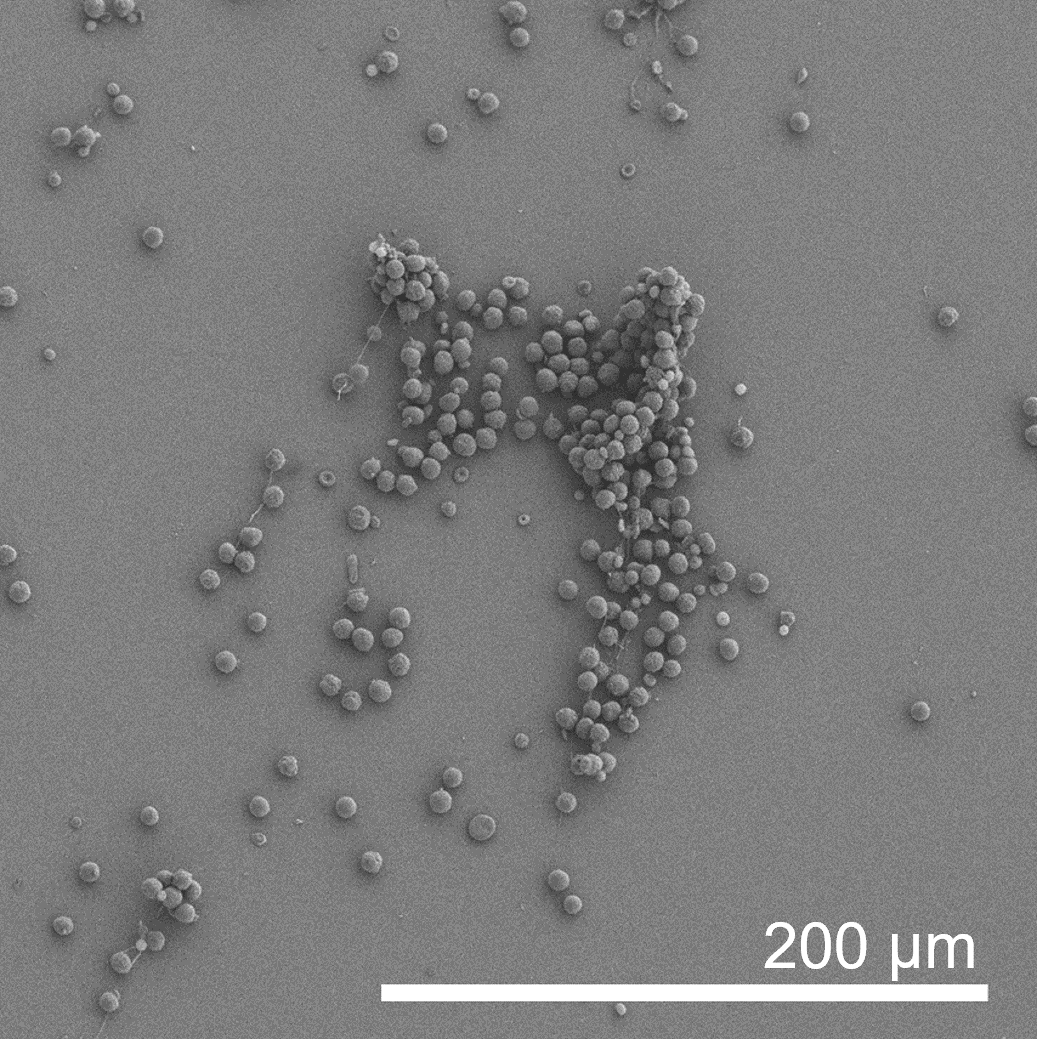

Supplement: Supplementary file 4 — Source data Fig. 1 [file 44319_2024_150_MOESM4_ESM.zip › Main Figure 1/Fig 1C/EM/unstim.png]

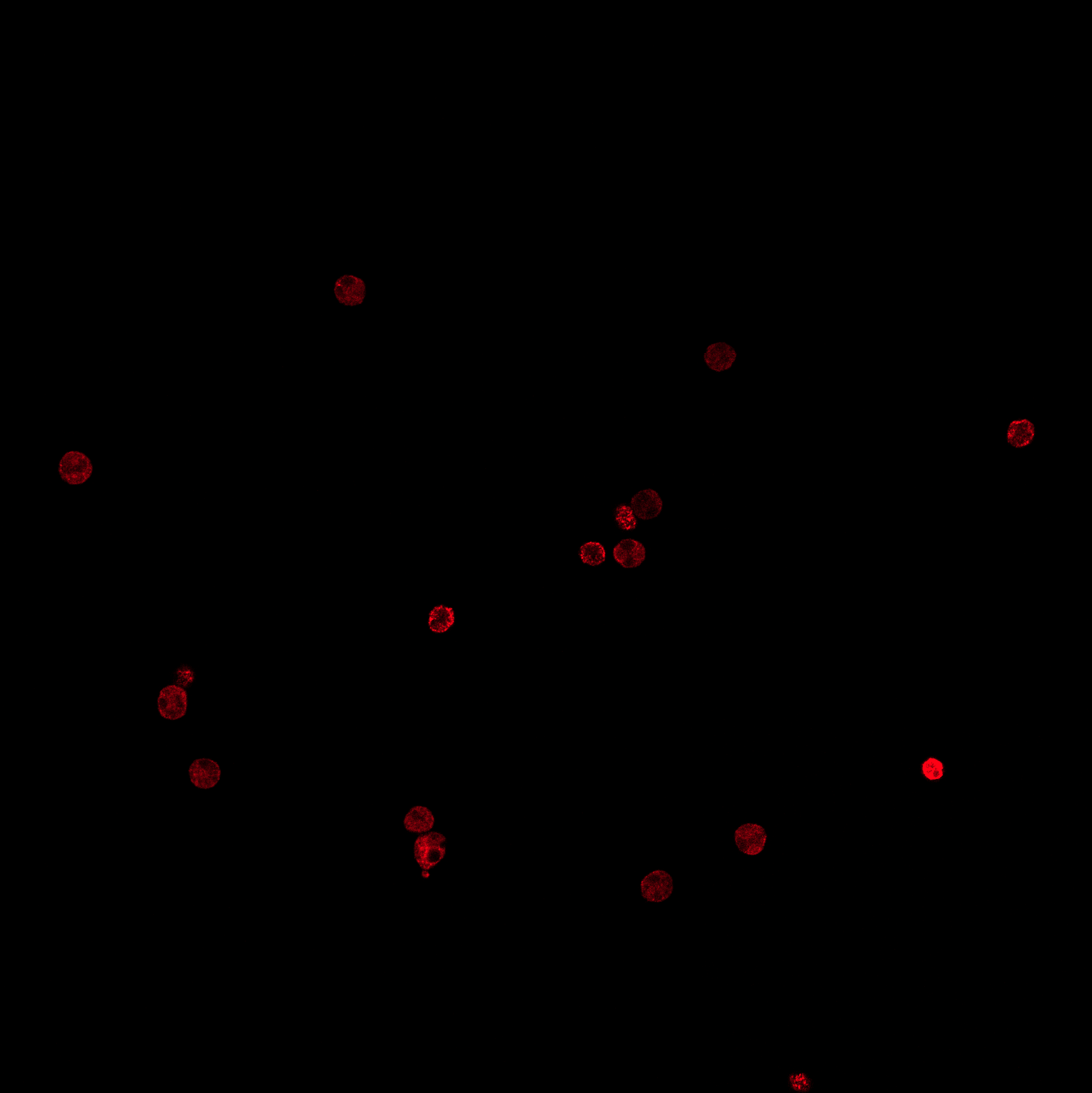

Supplement: Supplementary file 4 — Source data Fig. 1 [file 44319_2024_150_MOESM4_ESM.zip › Main Figure 1/Fig 1F/FB-175 images/Mock NETs/C1-Experiment-3210-Airyscan Processing-03.png]

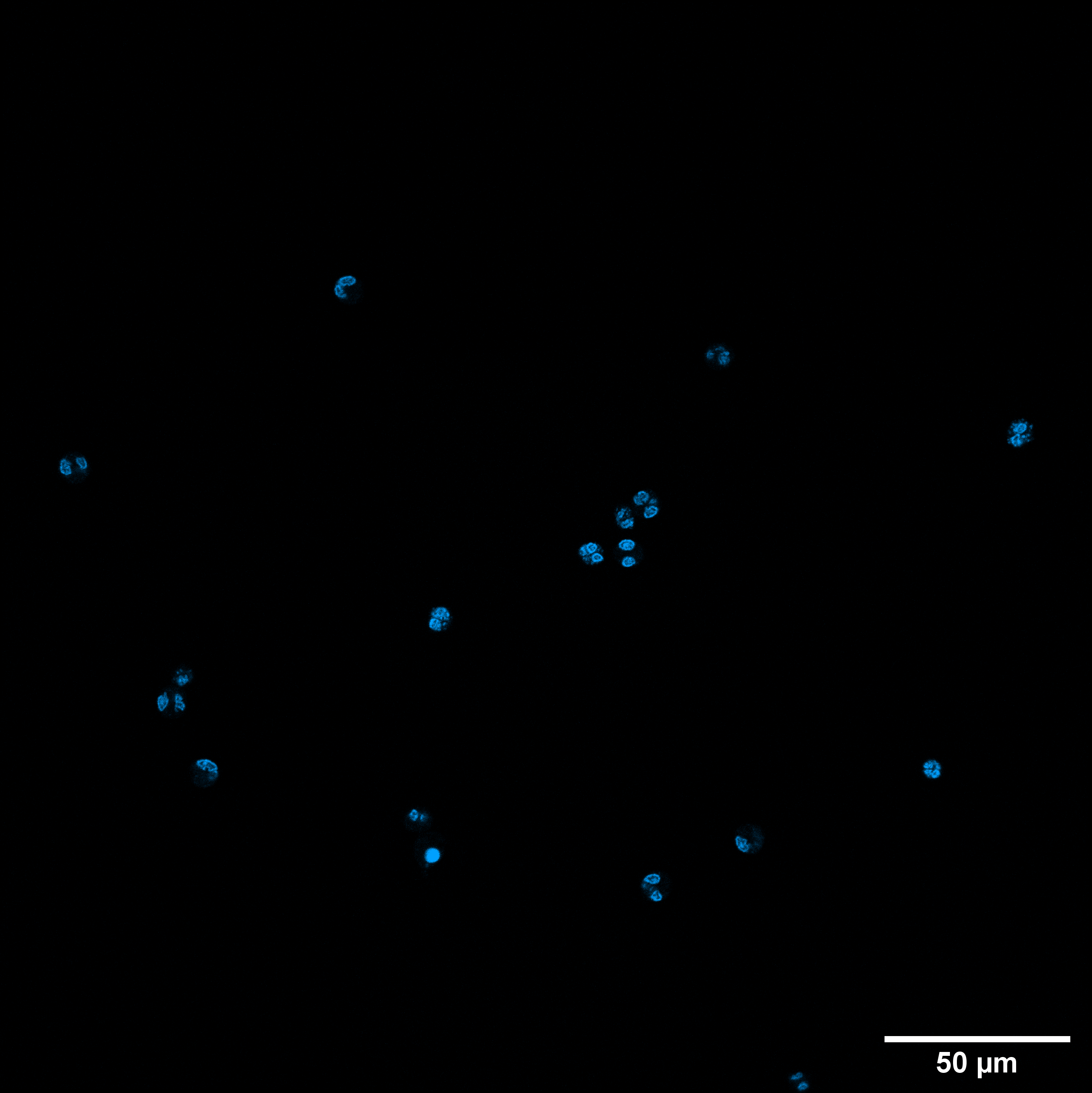

Supplement: Supplementary file 4 — Source data Fig. 1 [file 44319_2024_150_MOESM4_ESM.zip › Main Figure 1/Fig 1F/FB-175 images/Mock NETs/C2-Experiment-3210-Airyscan Processing-03.png]

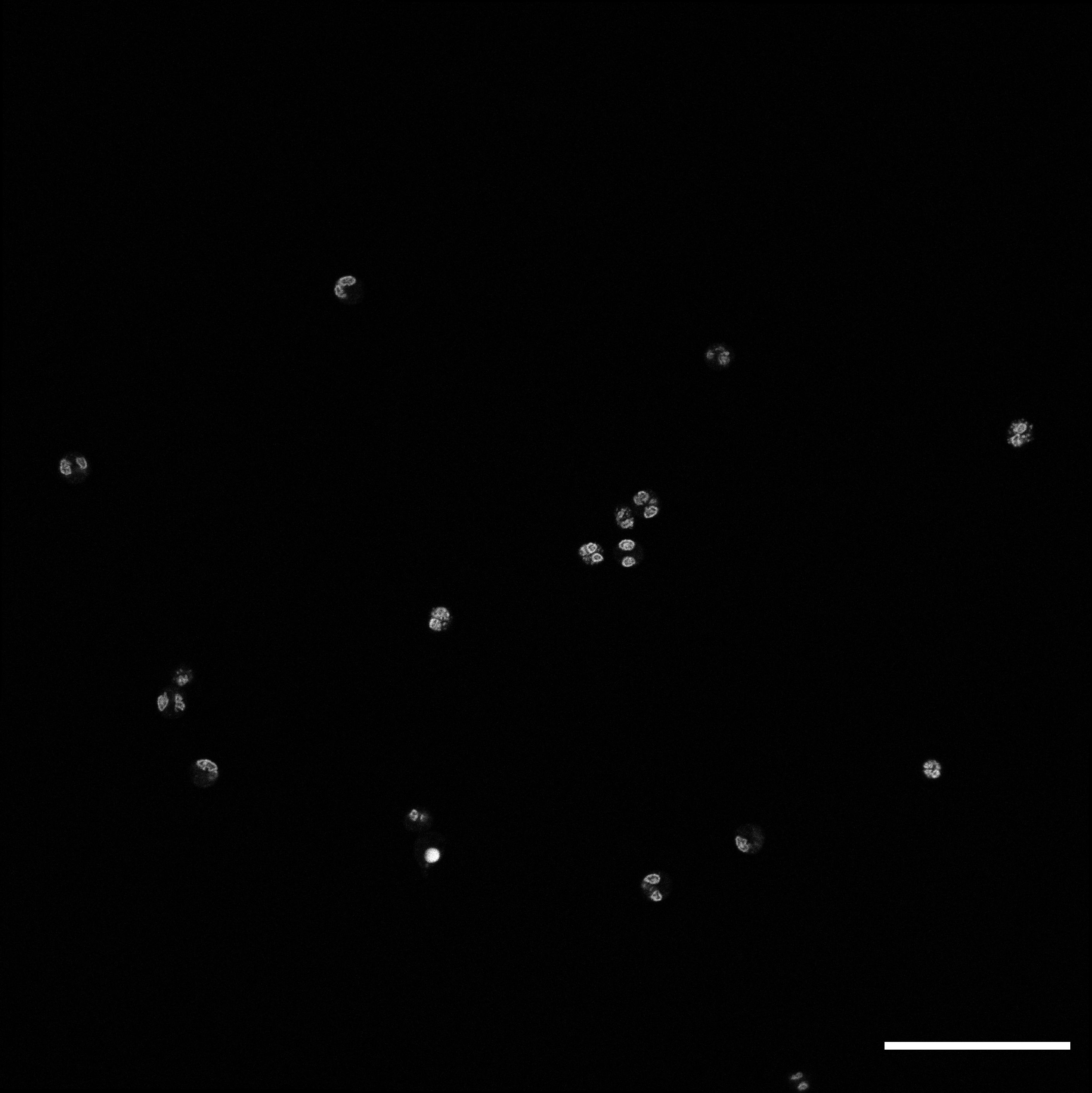

Supplement: Supplementary file 4 — Source data Fig. 1 [file 44319_2024_150_MOESM4_ESM.zip › Main Figure 1/Fig 1F/FB-175 images/Mock NETs/gray.png]

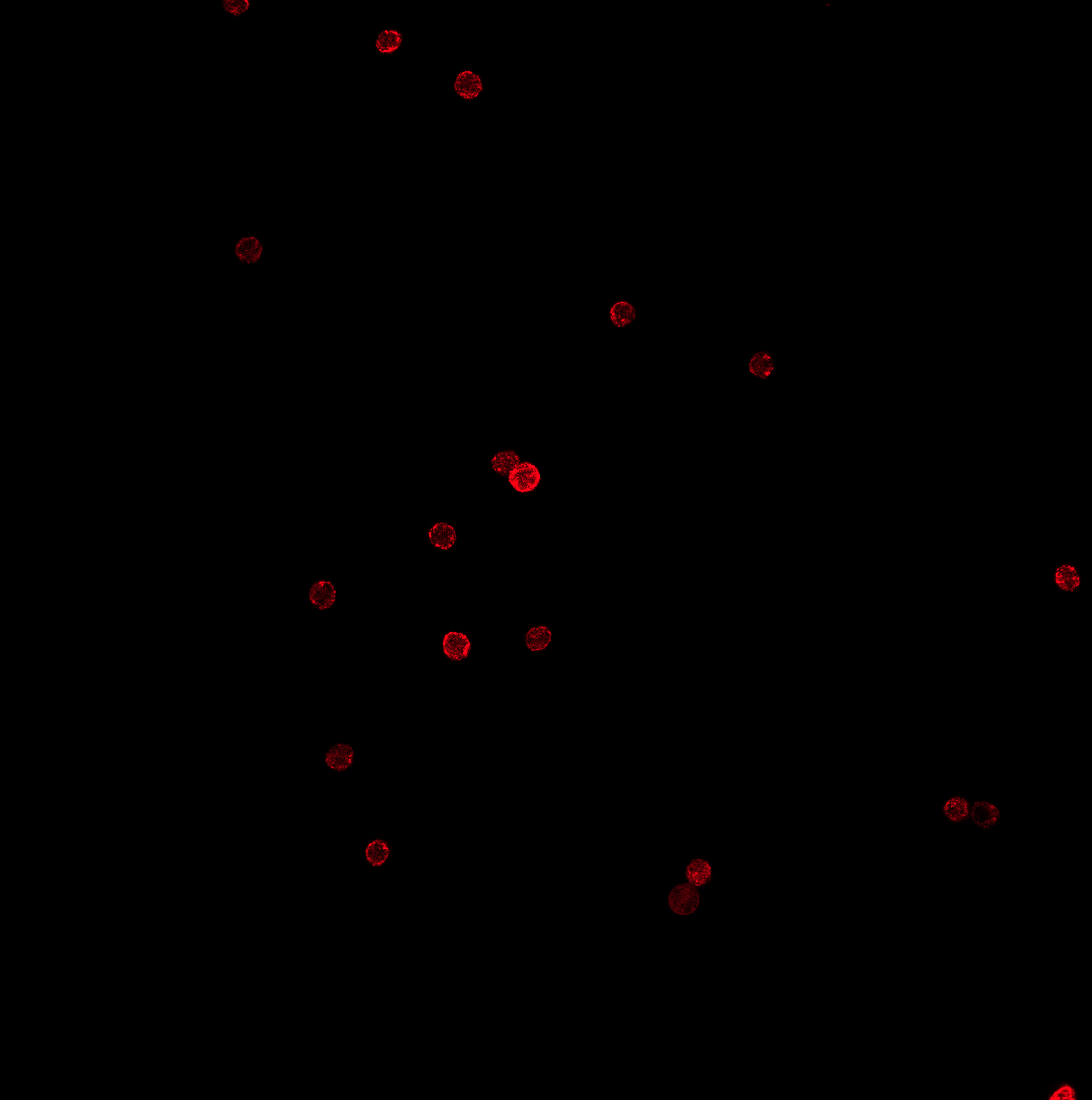

Supplement: Supplementary file 4 — Source data Fig. 1 [file 44319_2024_150_MOESM4_ESM.zip › Main Figure 1/Fig 1F/FB-175 images/Mock NETs RNase/C1-Experiment-3212-Airyscan Processing-05.png]

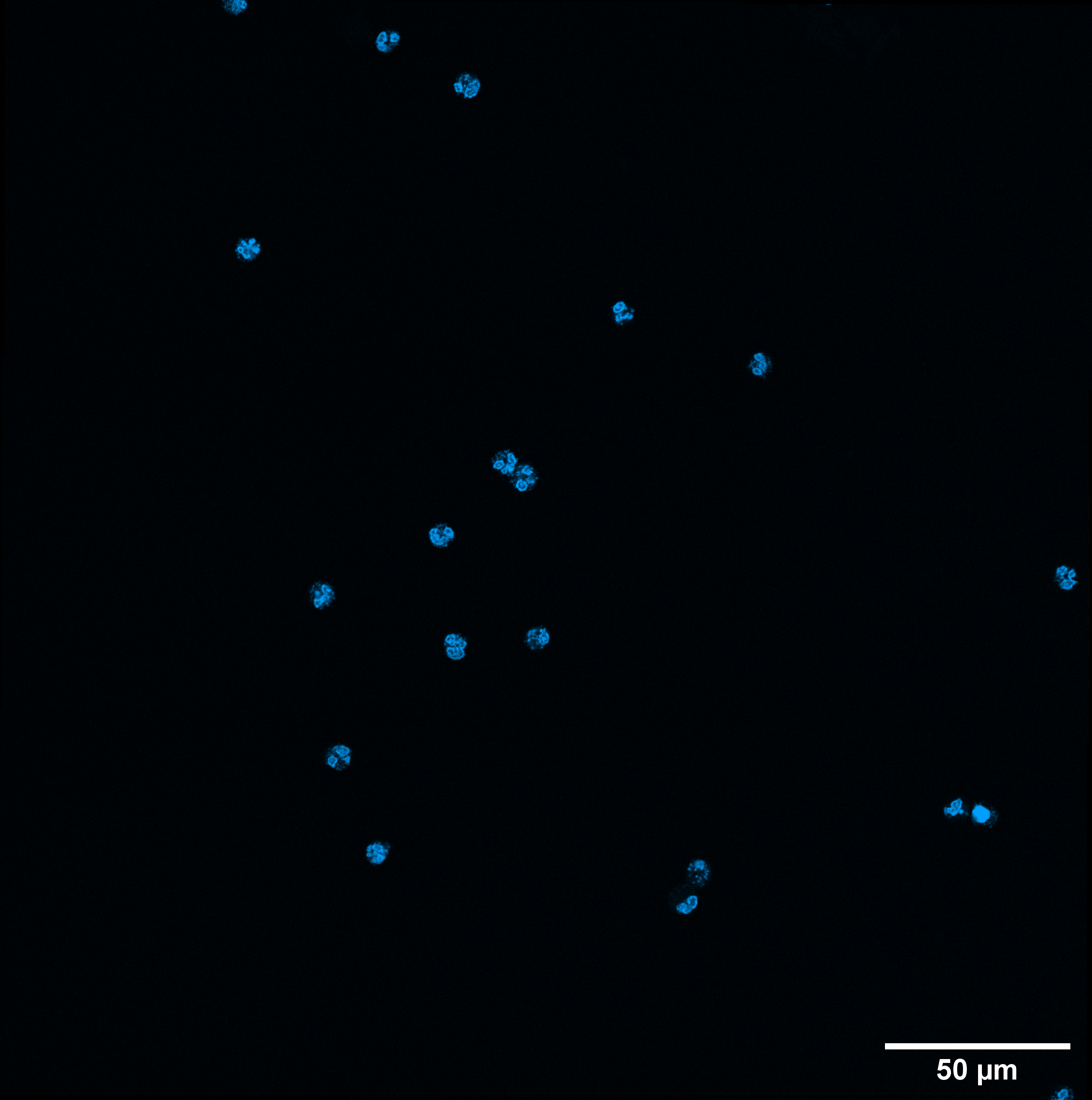

Supplement: Supplementary file 4 — Source data Fig. 1 [file 44319_2024_150_MOESM4_ESM.zip › Main Figure 1/Fig 1F/FB-175 images/Mock NETs RNase/C2-Experiment-3212-Airyscan Processing-05.png]

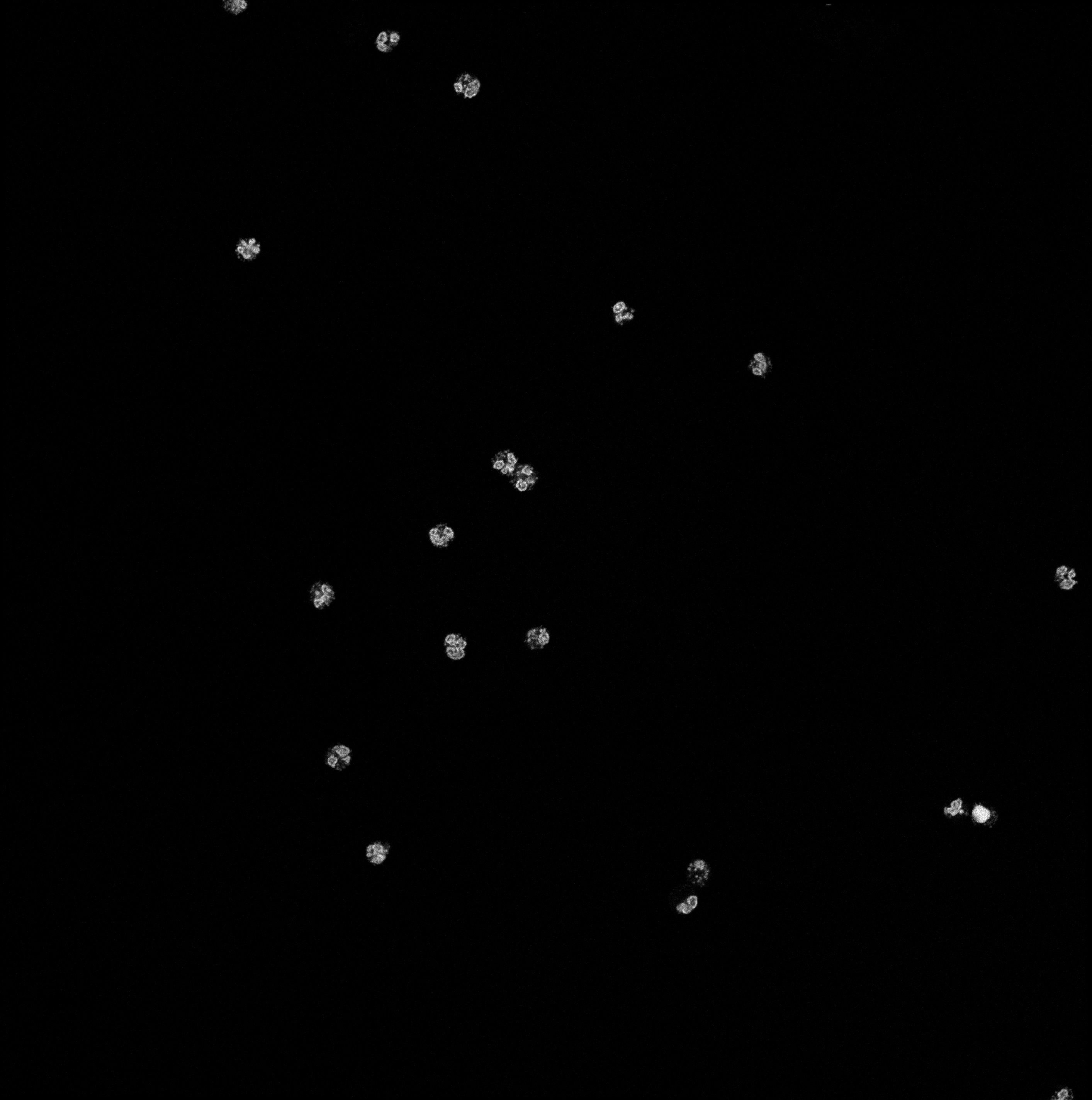

Supplement: Supplementary file 4 — Source data Fig. 1 [file 44319_2024_150_MOESM4_ESM.zip › Main Figure 1/Fig 1F/FB-175 images/Mock NETs RNase/gray.png]

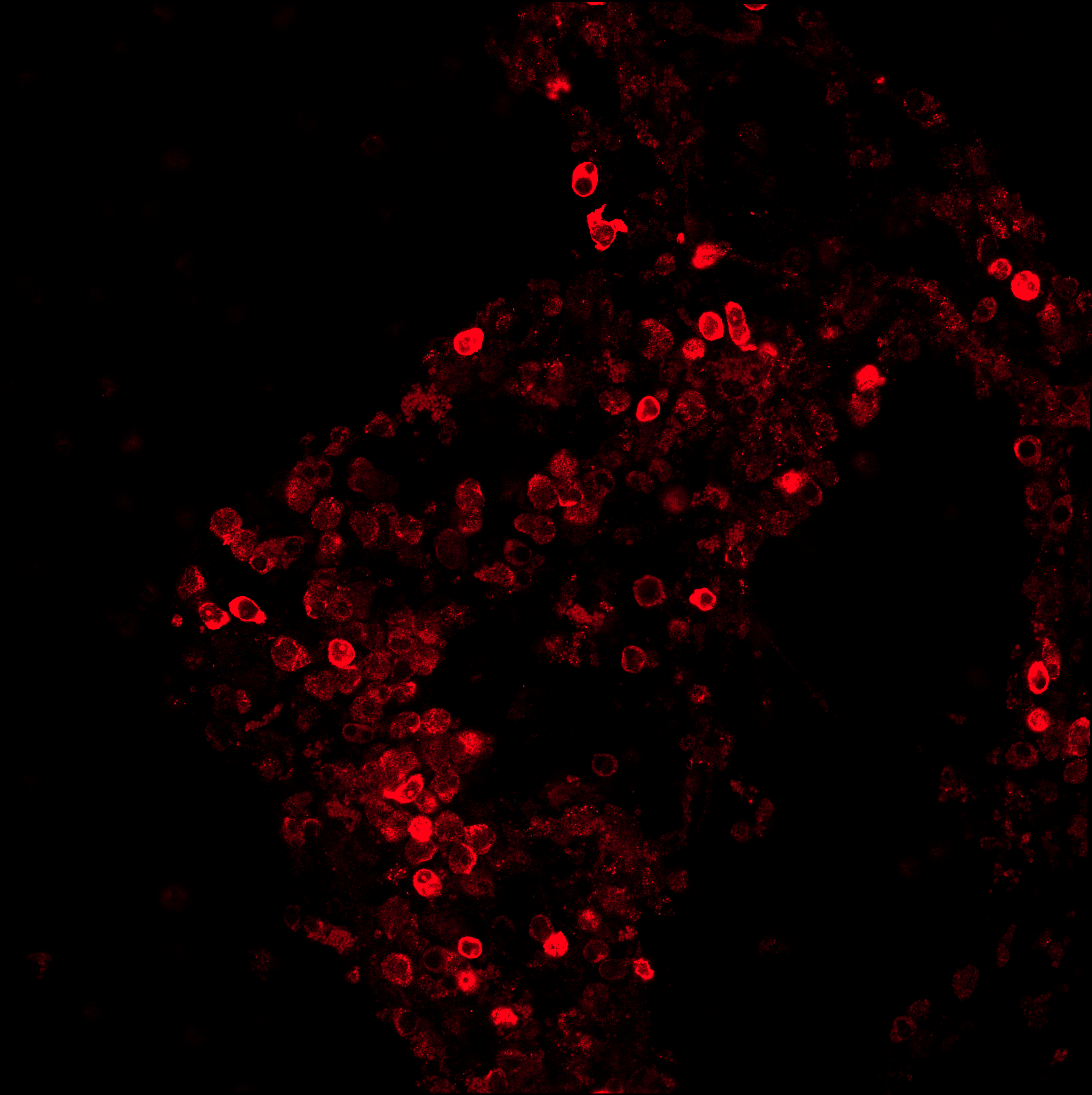

Supplement: Supplementary file 4 — Source data Fig. 1 [file 44319_2024_150_MOESM4_ESM.zip › Main Figure 1/Fig 1F/FB-175 images/PMA/C1-Experiment-3209-Airyscan Processing-02.png]

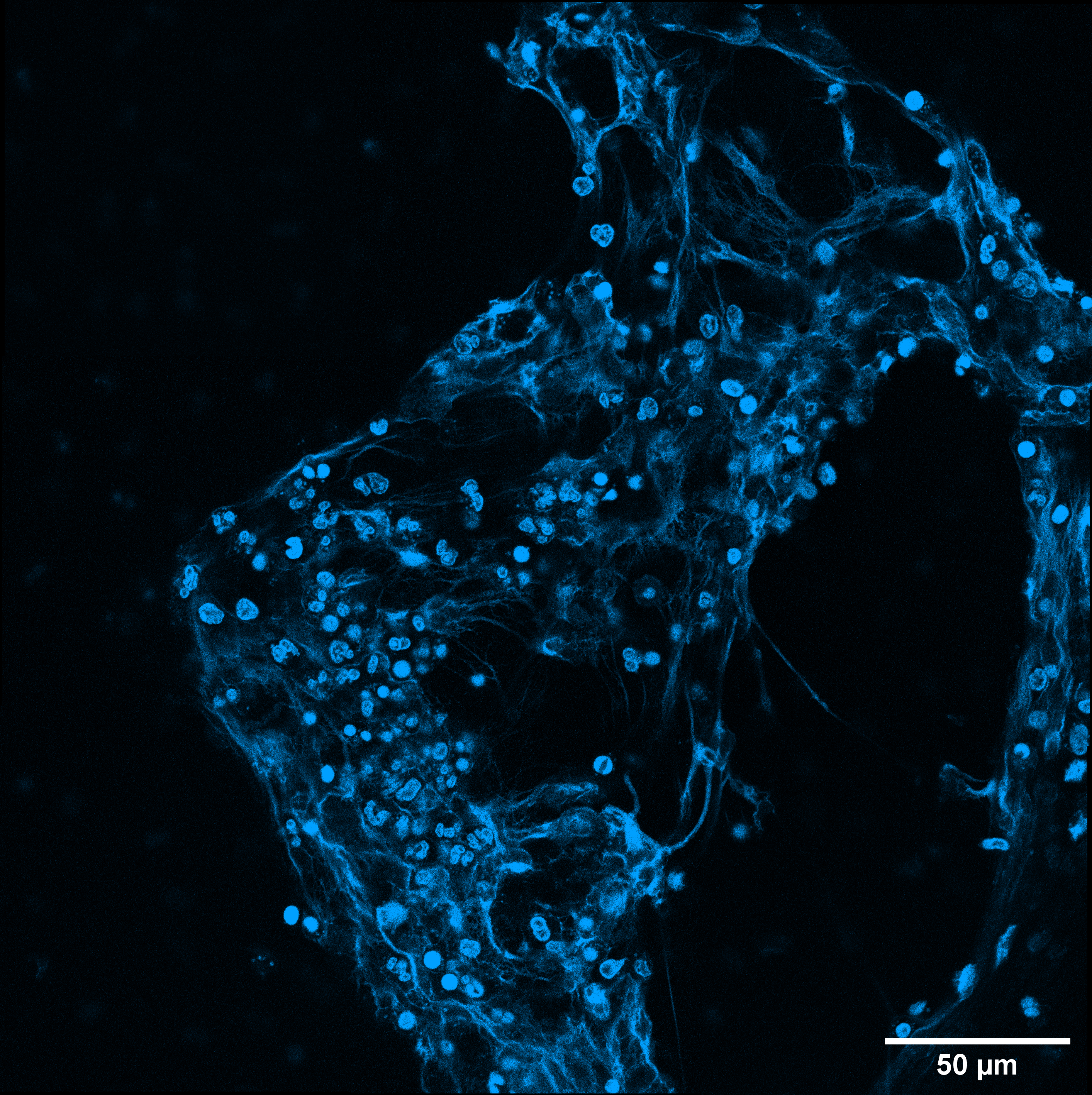

Supplement: Supplementary file 4 — Source data Fig. 1 [file 44319_2024_150_MOESM4_ESM.zip › Main Figure 1/Fig 1F/FB-175 images/PMA/C2-Experiment-3209-Airyscan Processing-02.png]

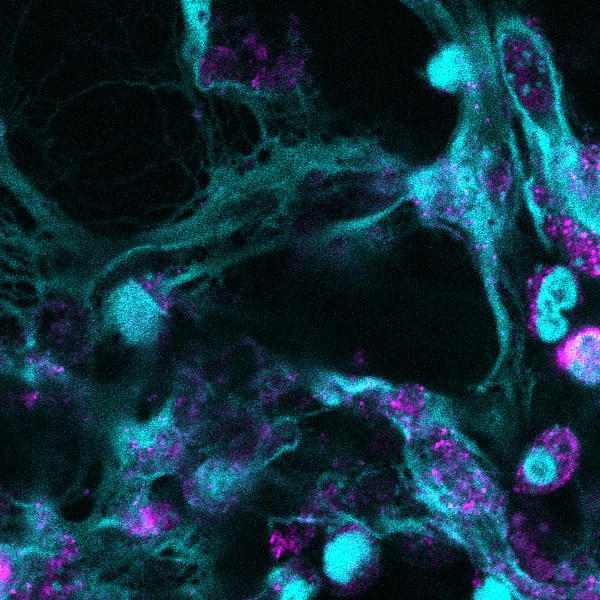

Supplement: Supplementary file 4 — Source data Fig. 1 [file 44319_2024_150_MOESM4_ESM.zip › Main Figure 1/Fig 1F/FB-175 images/PMA/comp new crop.png]

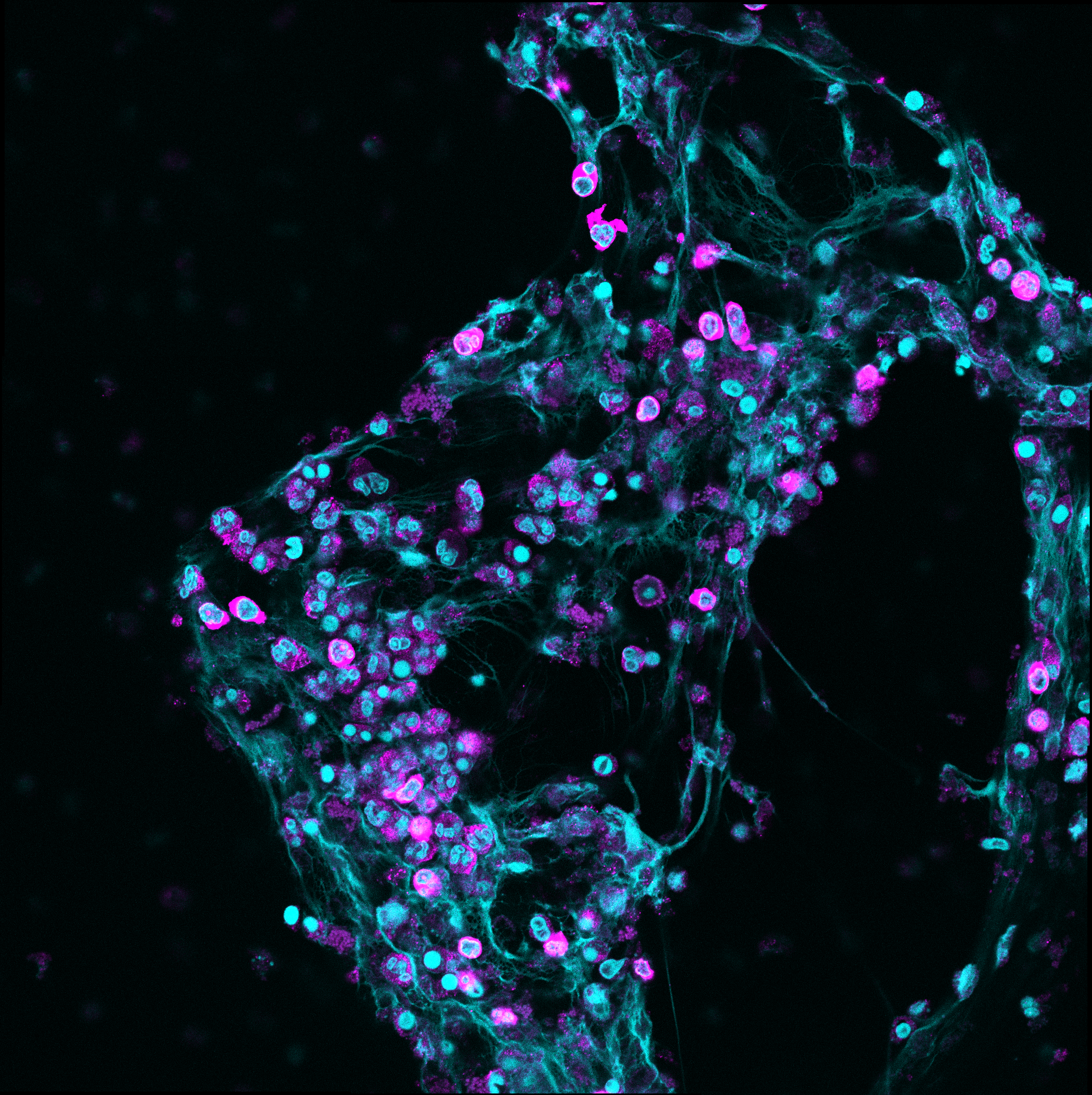

Supplement: Supplementary file 4 — Source data Fig. 1 [file 44319_2024_150_MOESM4_ESM.zip › Main Figure 1/Fig 1F/FB-175 images/PMA/comp new.png]

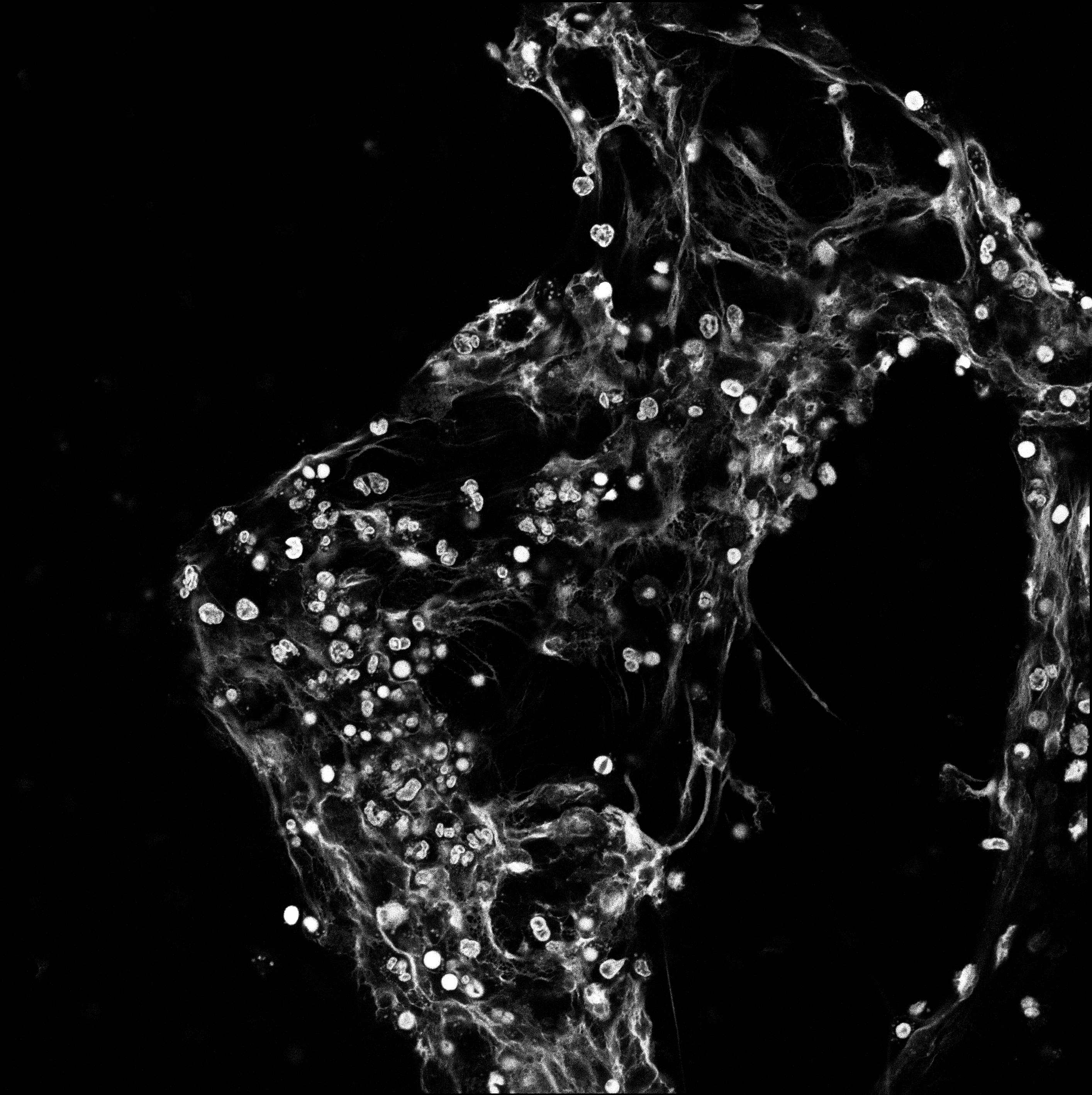

Supplement: Supplementary file 4 — Source data Fig. 1 [file 44319_2024_150_MOESM4_ESM.zip › Main Figure 1/Fig 1F/FB-175 images/PMA/gray.png]

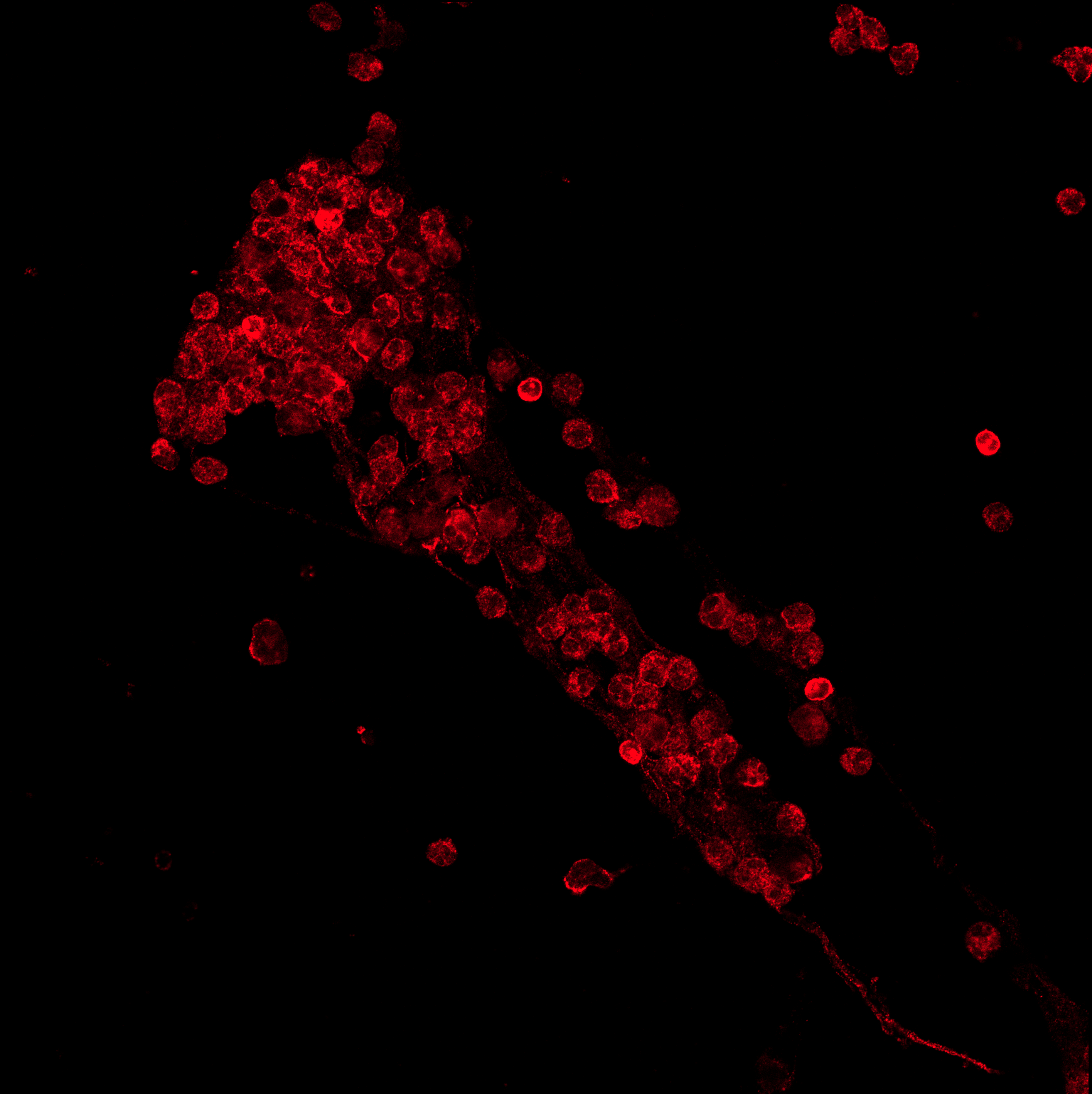

Supplement: Supplementary file 4 — Source data Fig. 1 [file 44319_2024_150_MOESM4_ESM.zip › Main Figure 1/Fig 1F/FB-175 images/PMA NETs/C1-Experiment-3211-Airyscan Processing-04.png]

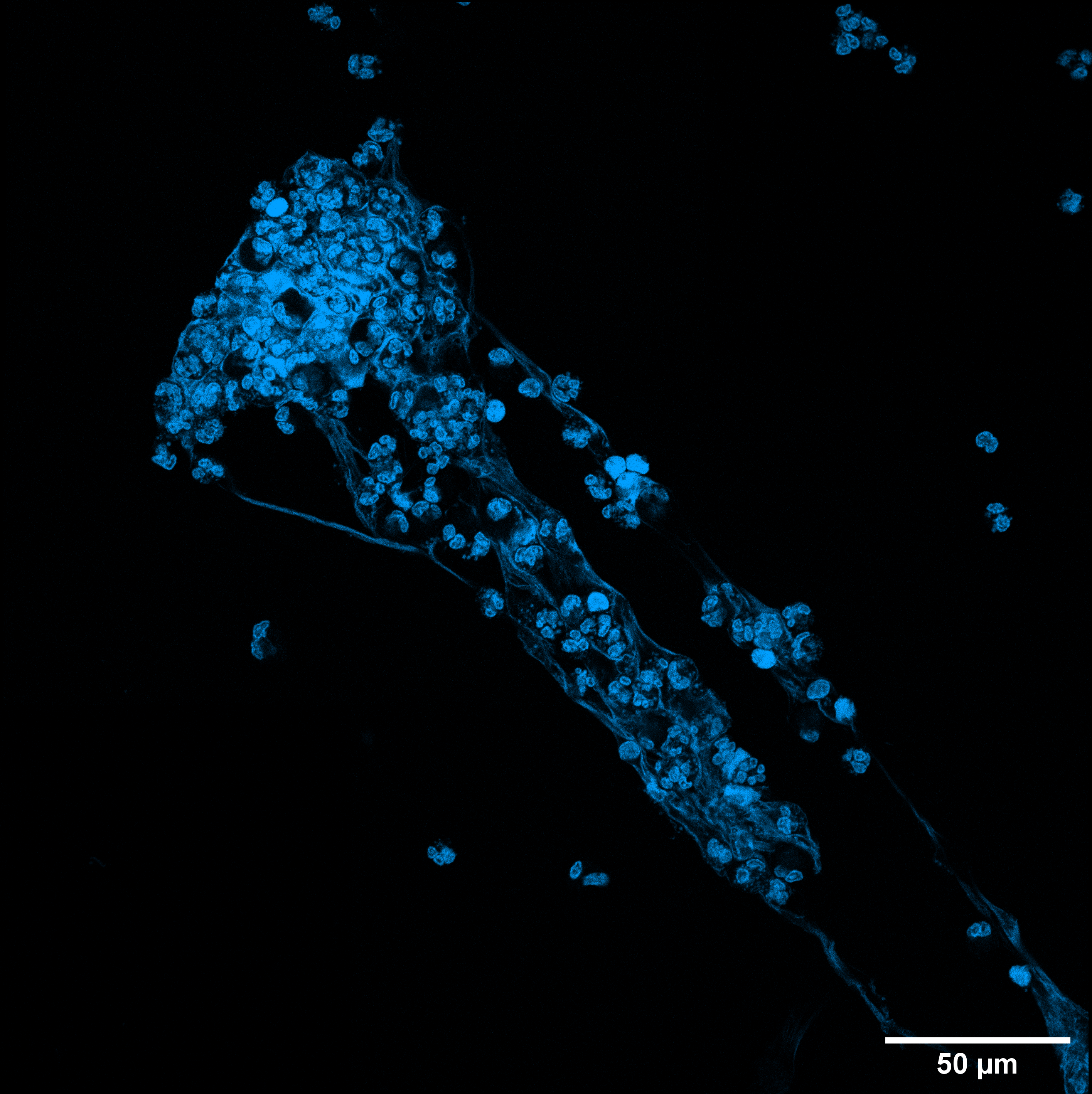

Supplement: Supplementary file 4 — Source data Fig. 1 [file 44319_2024_150_MOESM4_ESM.zip › Main Figure 1/Fig 1F/FB-175 images/PMA NETs/C2-Experiment-3211-Airyscan Processing-04.png]

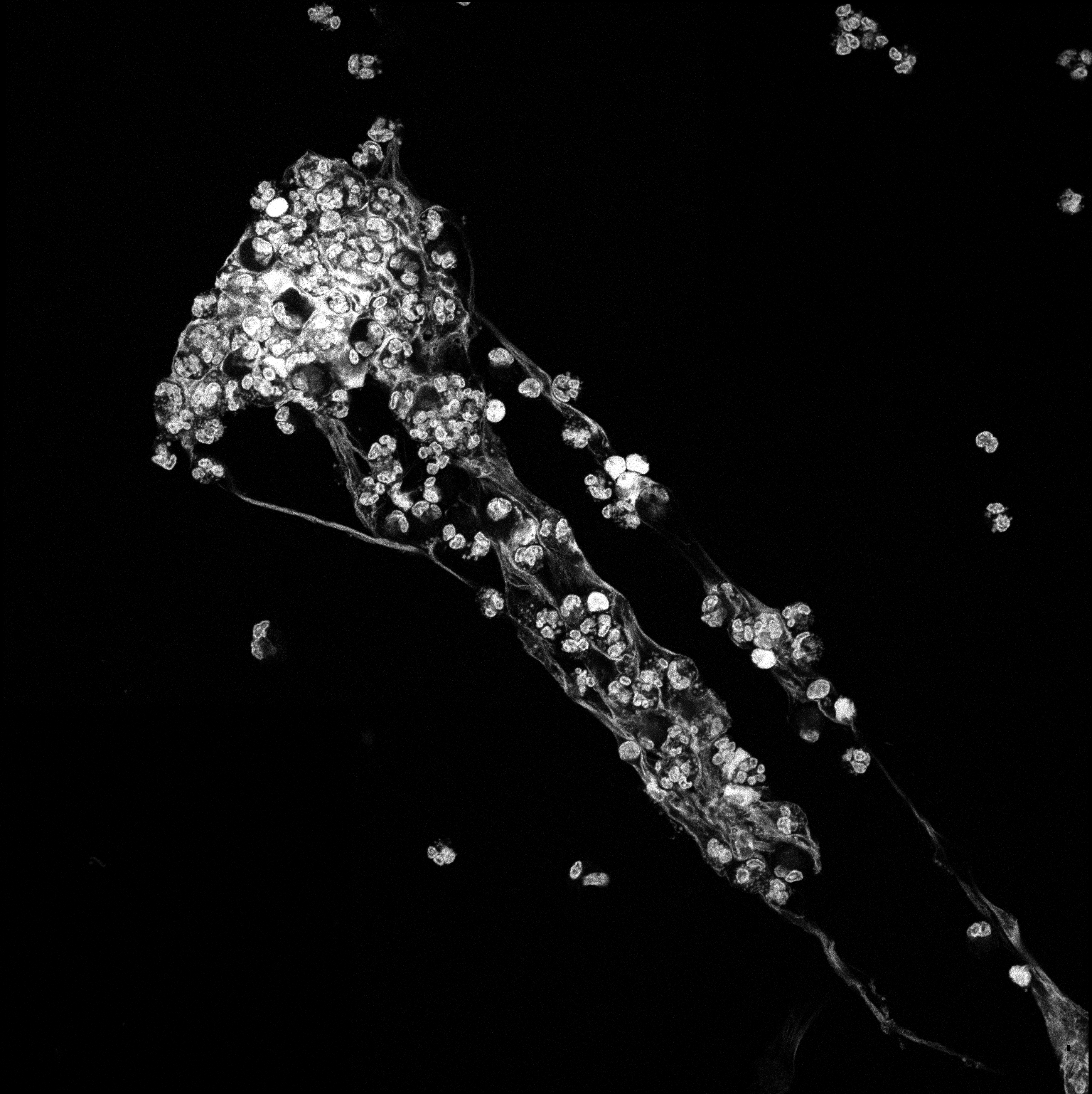

Supplement: Supplementary file 4 — Source data Fig. 1 [file 44319_2024_150_MOESM4_ESM.zip › Main Figure 1/Fig 1F/FB-175 images/PMA NETs/gray.png]

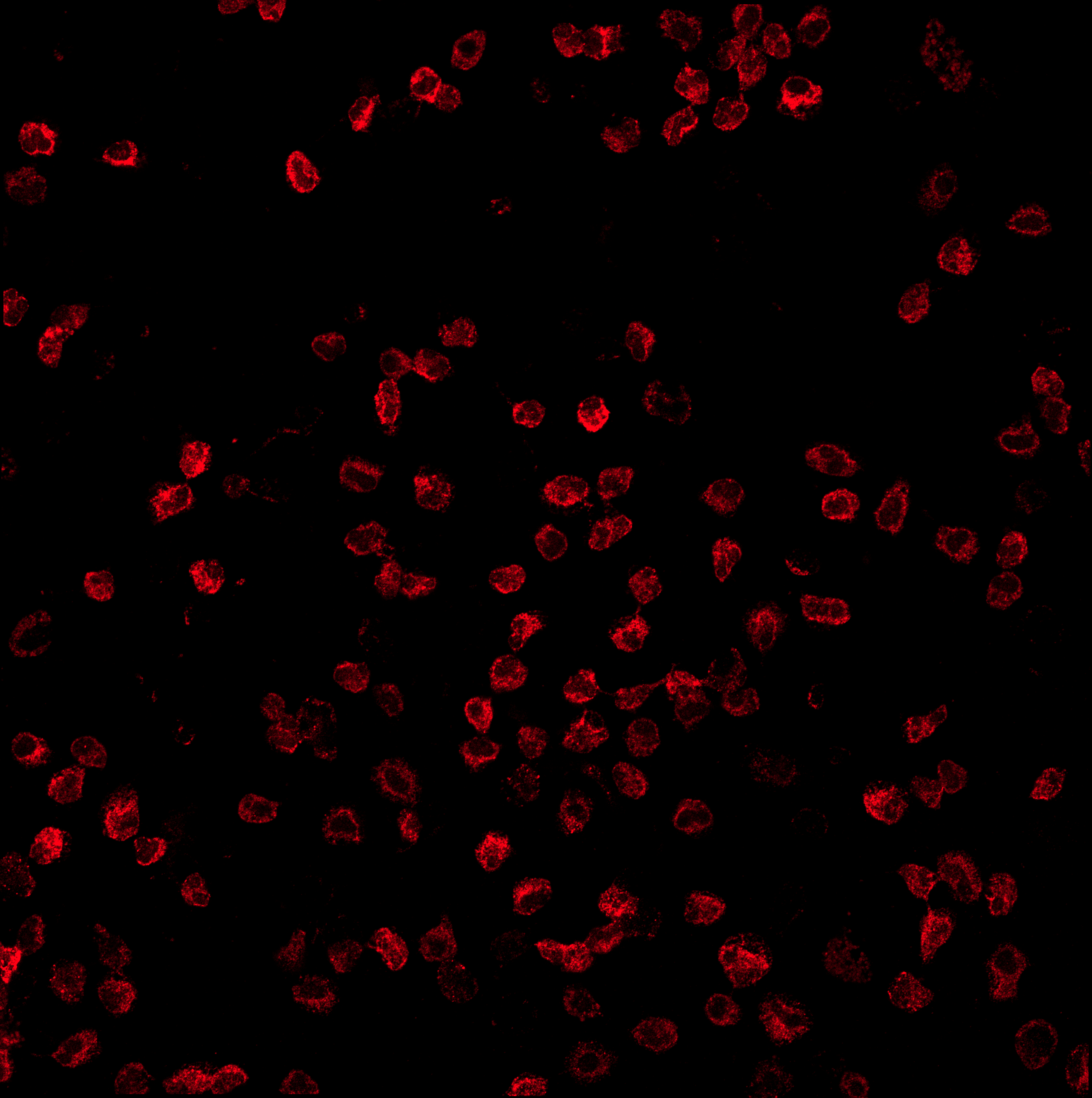

Supplement: Supplementary file 4 — Source data Fig. 1 [file 44319_2024_150_MOESM4_ESM.zip › Main Figure 1/Fig 1F/FB-175 images/PMA NETs rnase/C1-Experiment-3213-Airyscan Processing-06.png]

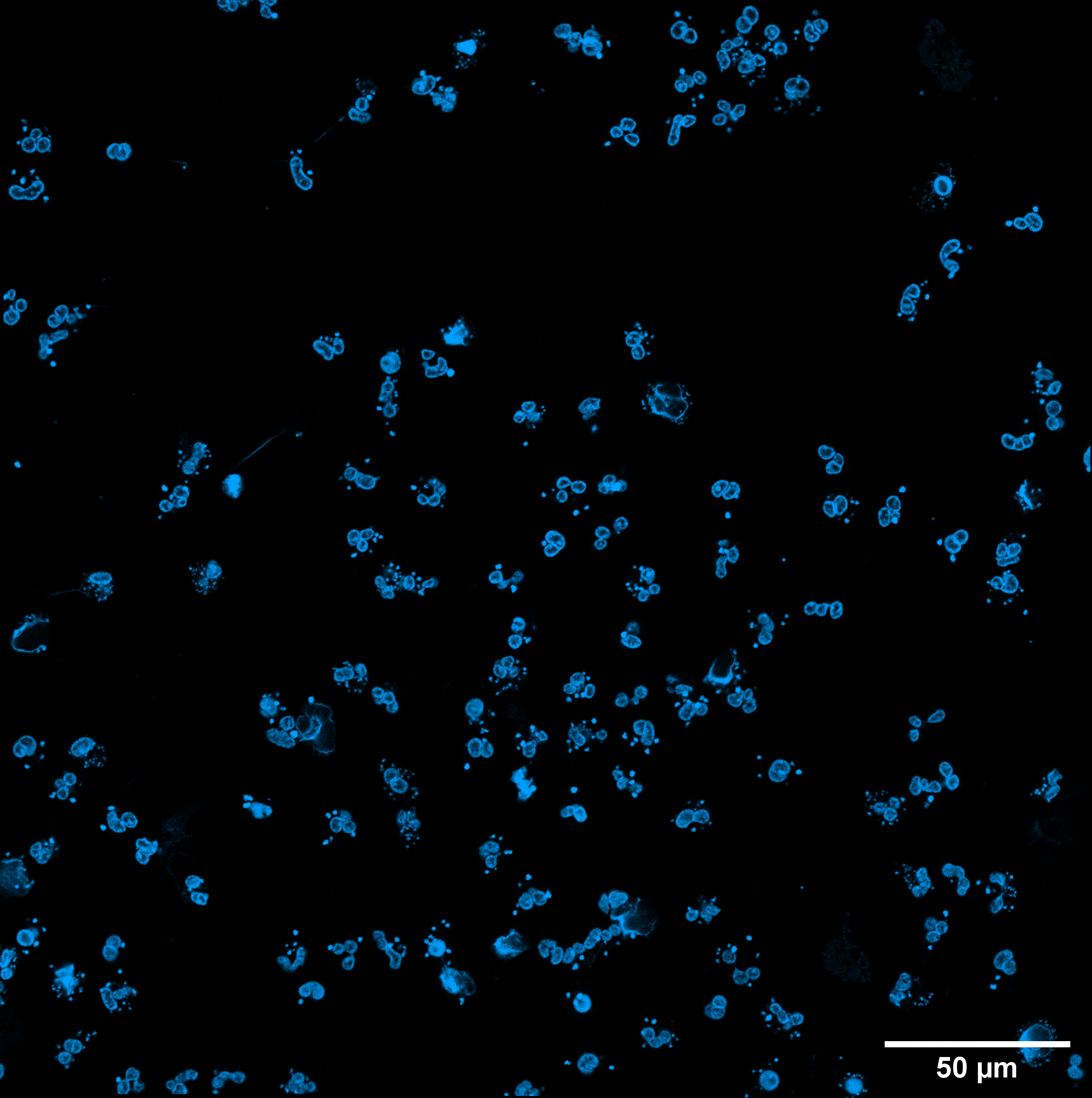

Supplement: Supplementary file 4 — Source data Fig. 1 [file 44319_2024_150_MOESM4_ESM.zip › Main Figure 1/Fig 1F/FB-175 images/PMA NETs rnase/C2-Experiment-3213-Airyscan Processing-06.png]

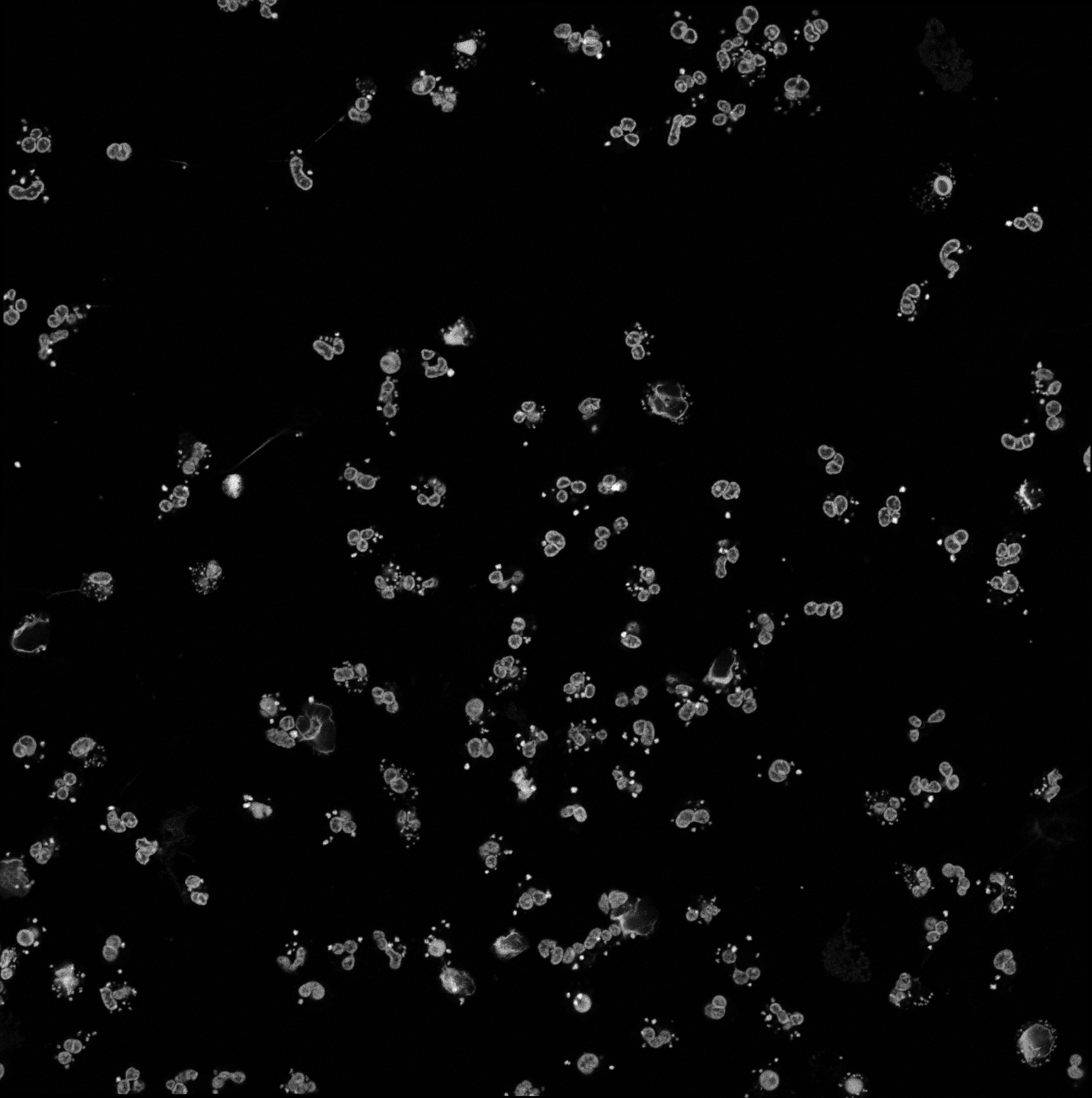

Supplement: Supplementary file 4 — Source data Fig. 1 [file 44319_2024_150_MOESM4_ESM.zip › Main Figure 1/Fig 1F/FB-175 images/PMA NETs rnase/gray.png]

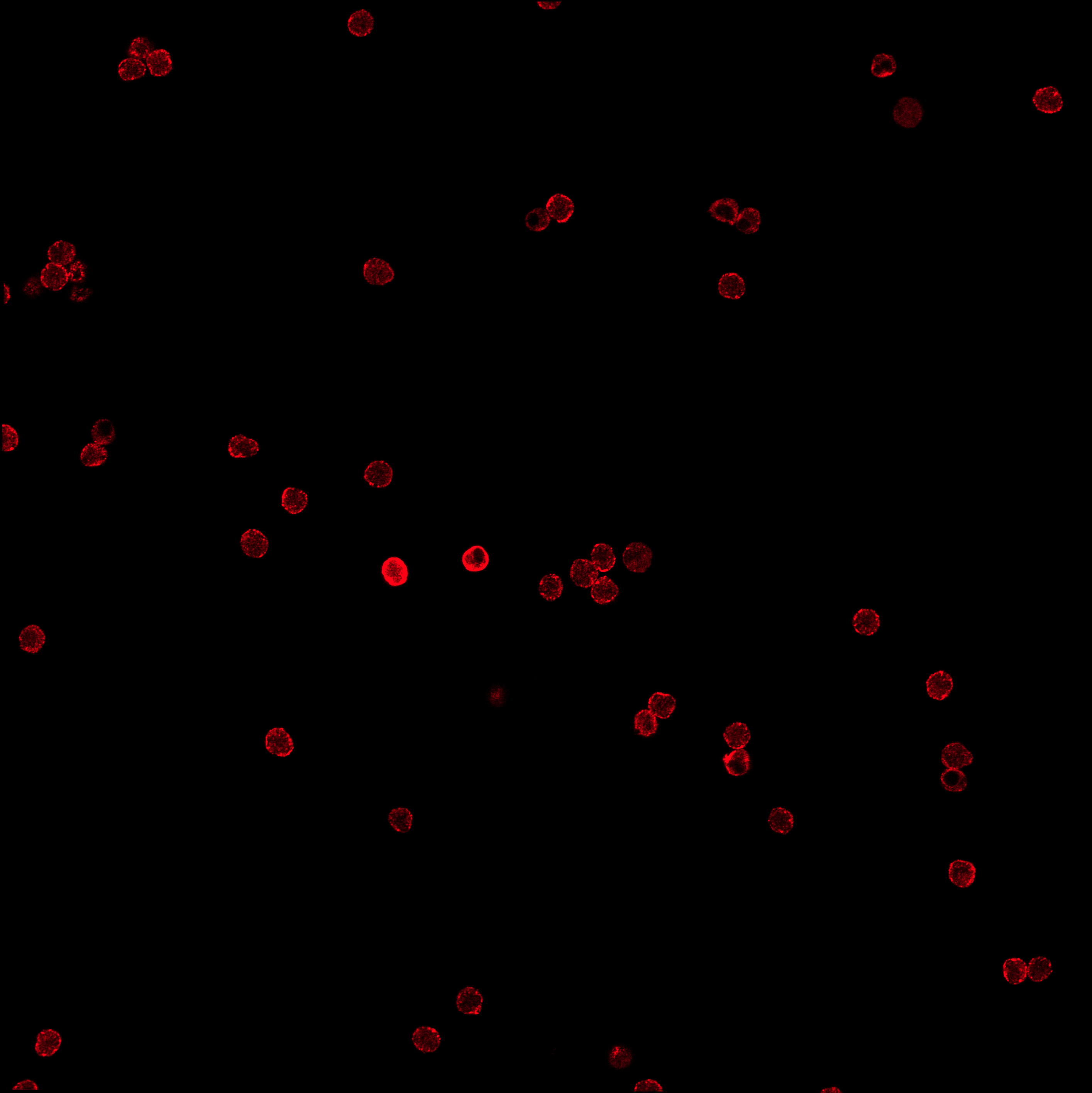

Supplement: Supplementary file 4 — Source data Fig. 1 [file 44319_2024_150_MOESM4_ESM.zip › Main Figure 1/Fig 1F/FB-175 images/Unstim/C1-Experiment-3208-Airyscan Processing-01.png]

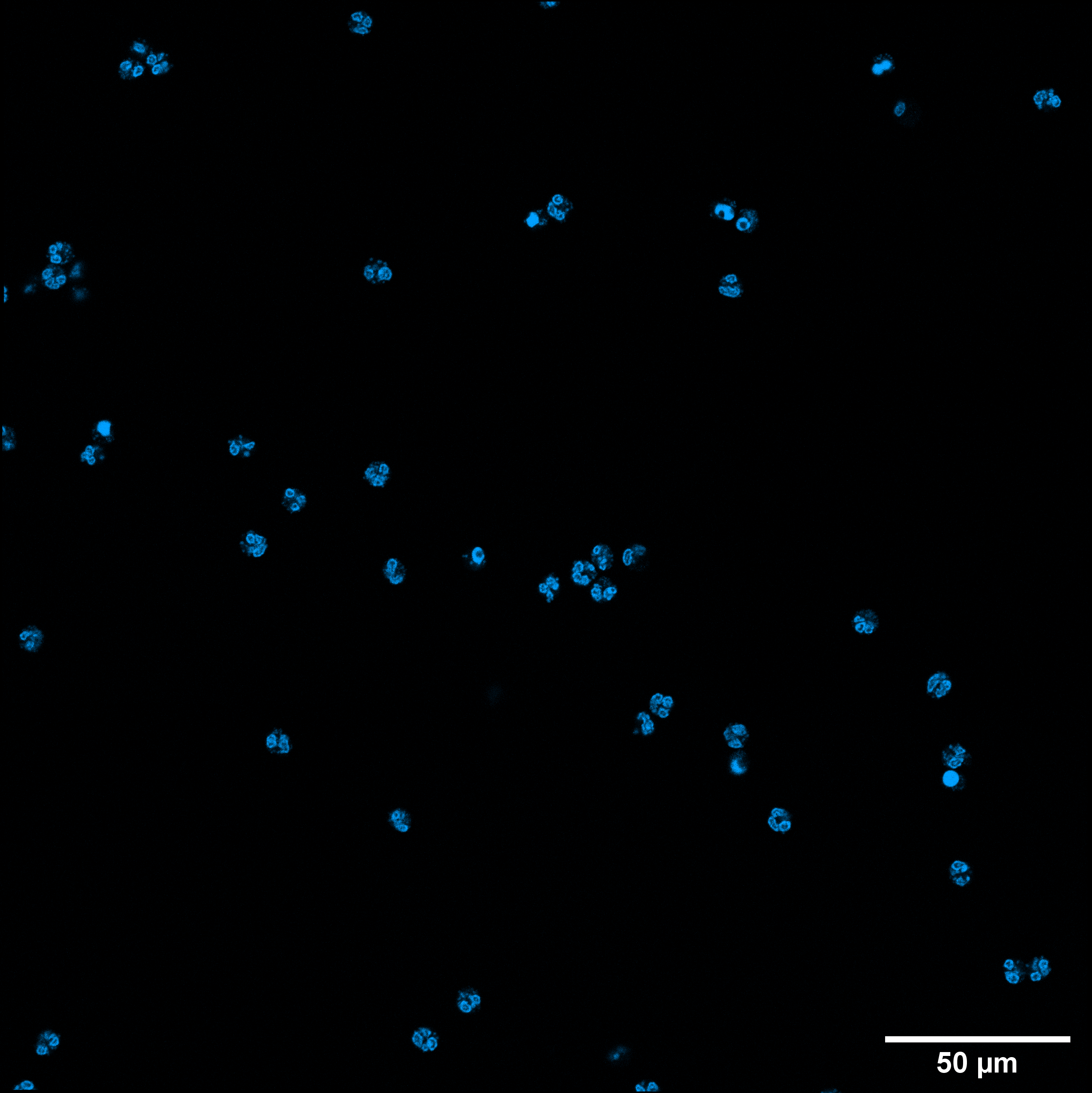

Supplement: Supplementary file 4 — Source data Fig. 1 [file 44319_2024_150_MOESM4_ESM.zip › Main Figure 1/Fig 1F/FB-175 images/Unstim/C2-Experiment-3208-Airyscan Processing-01.png]

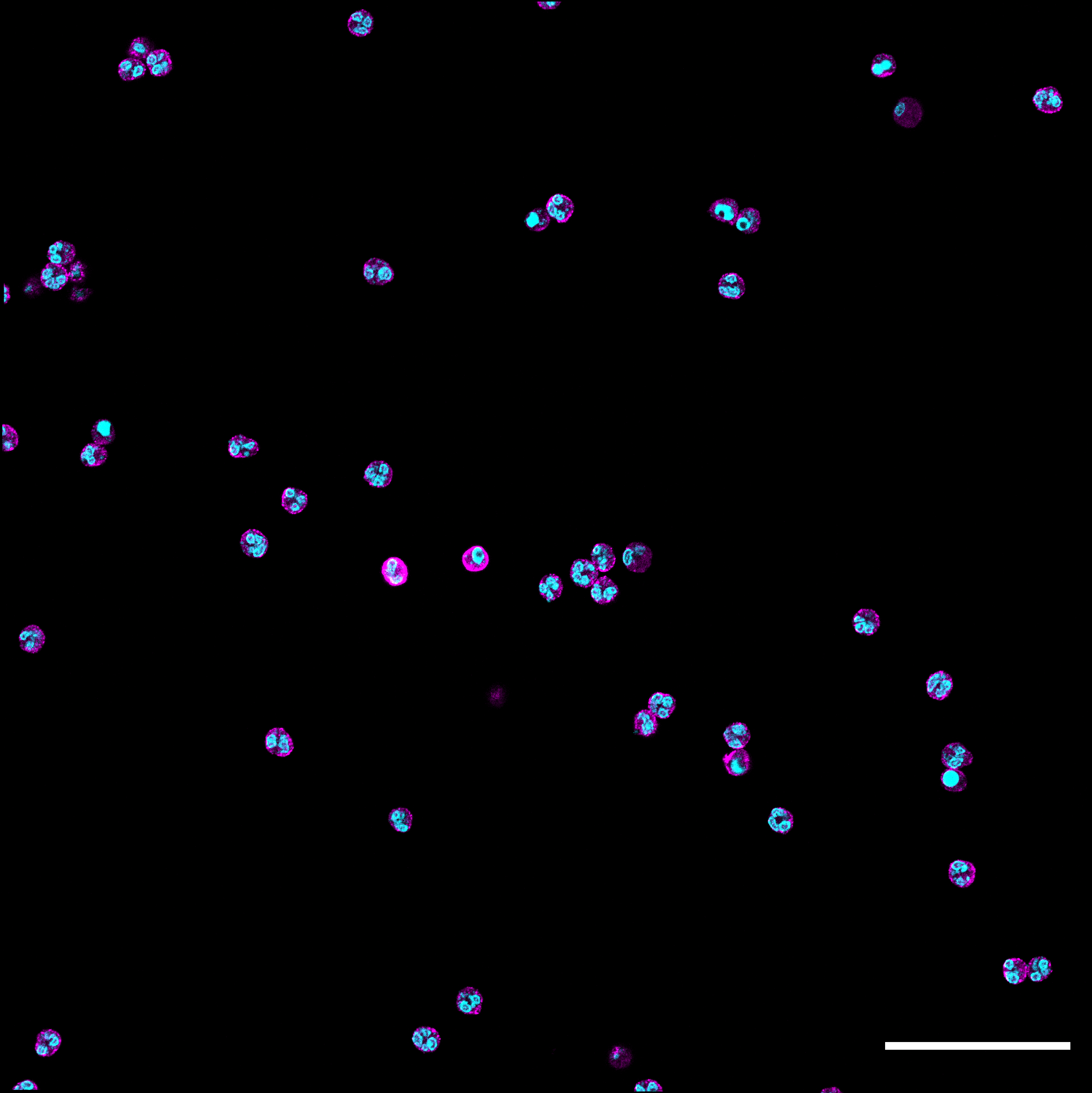

Supplement: Supplementary file 4 — Source data Fig. 1 [file 44319_2024_150_MOESM4_ESM.zip › Main Figure 1/Fig 1F/FB-175 images/Unstim/comp new.png]

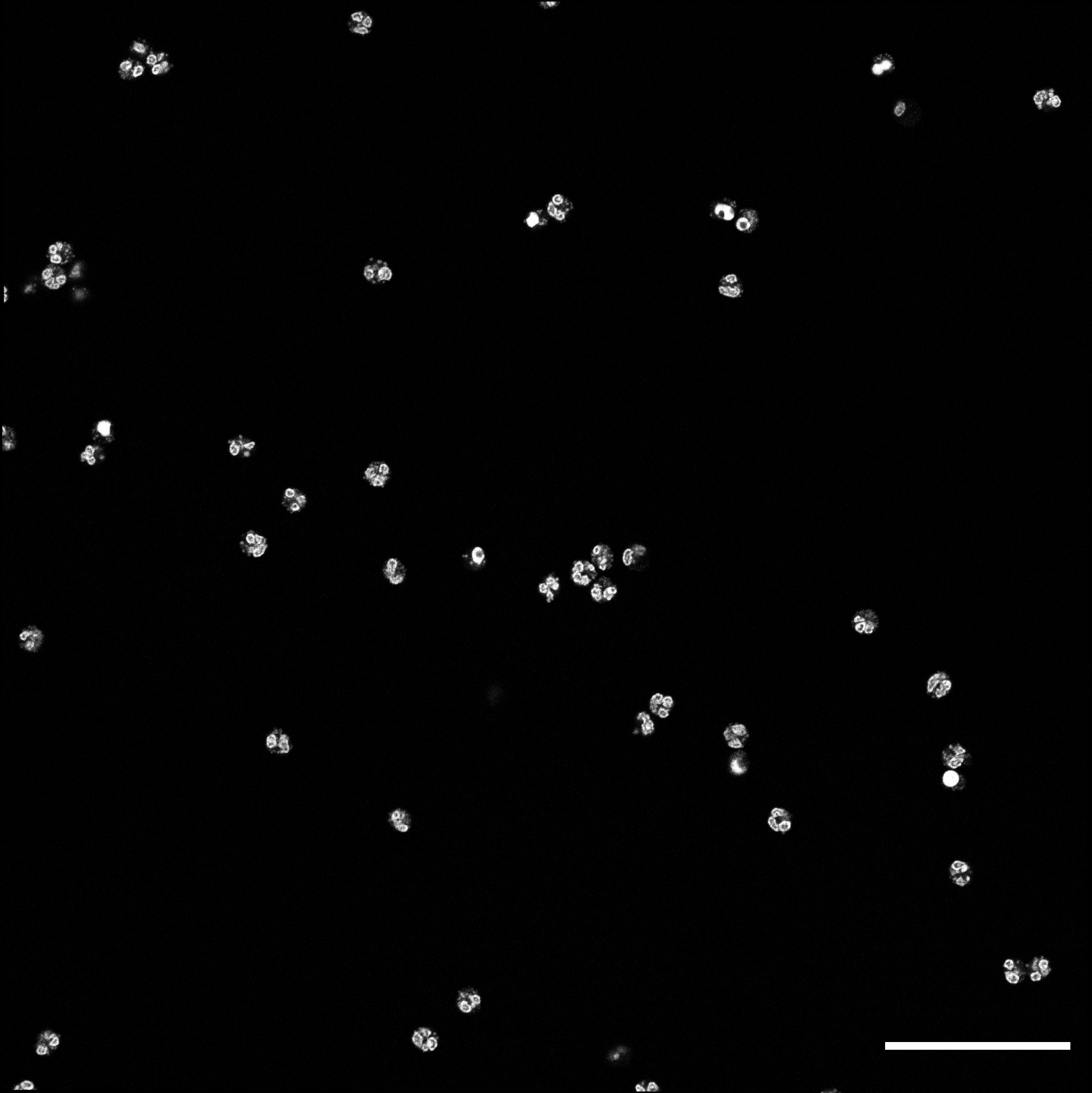

Supplement: Supplementary file 4 — Source data Fig. 1 [file 44319_2024_150_MOESM4_ESM.zip › Main Figure 1/Fig 1F/FB-175 images/Unstim/gray.png]

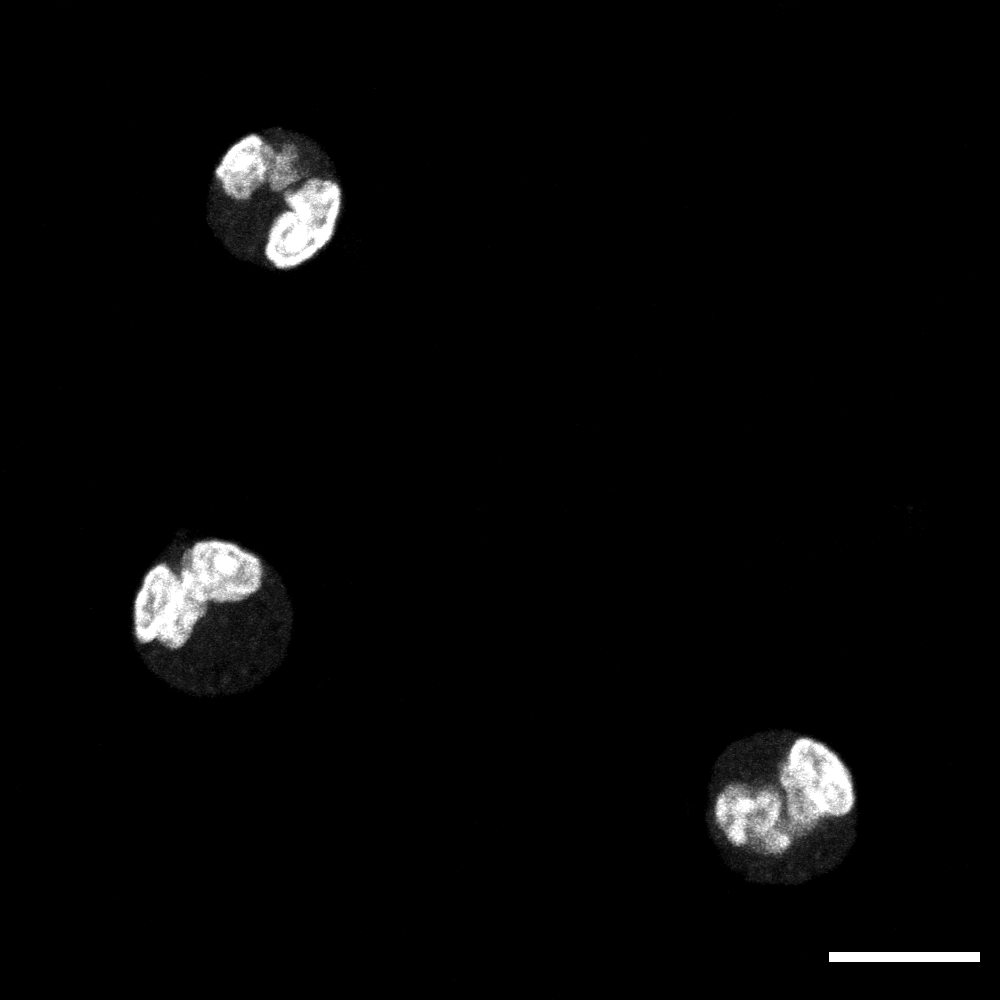

Supplement: Supplementary file 4 — Source data Fig. 1 [file 44319_2024_150_MOESM4_ESM.zip › Main Figure 1/Fig 1H/FB-168 images/mock narna/comp new sclae.png]

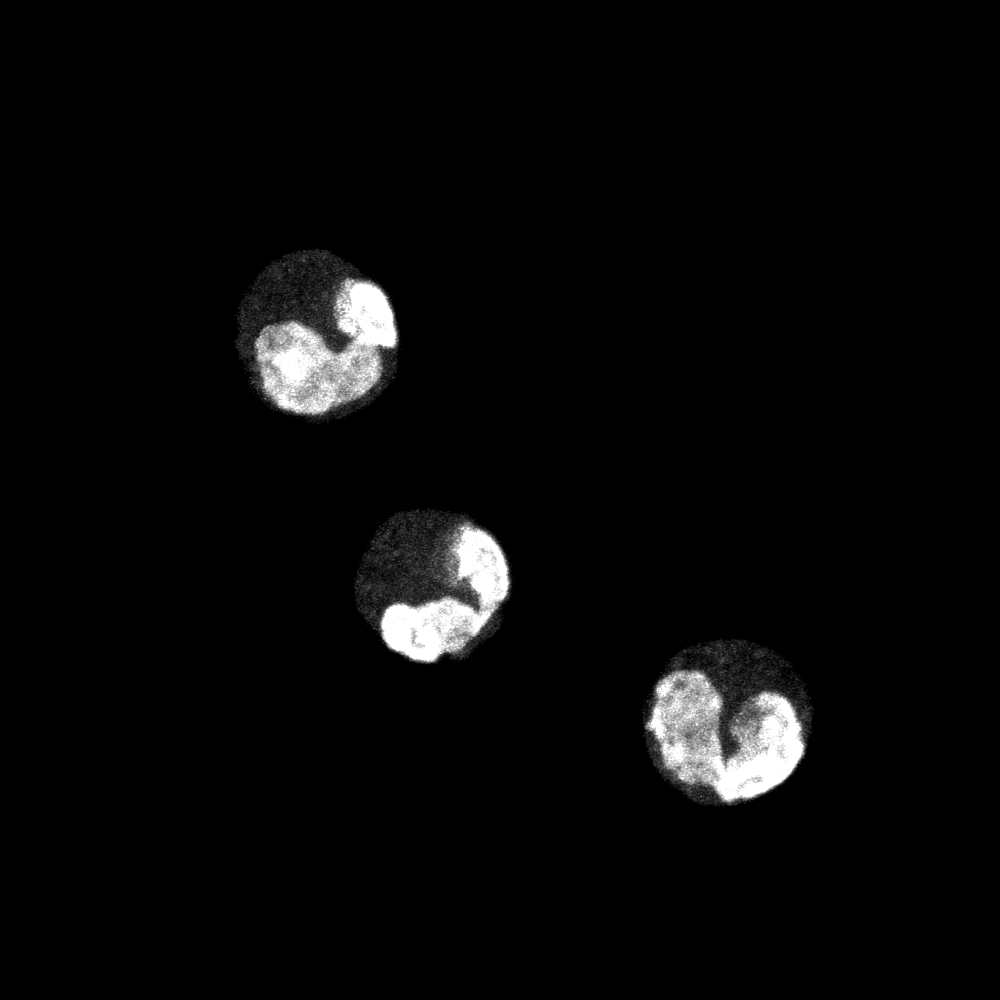

Supplement: Supplementary file 4 — Source data Fig. 1 [file 44319_2024_150_MOESM4_ESM.zip › Main Figure 1/Fig 1H/FB-168 images/mock narna+LL37/C2-MAX_Experiment-3516-Airyscan Processing-06.png]

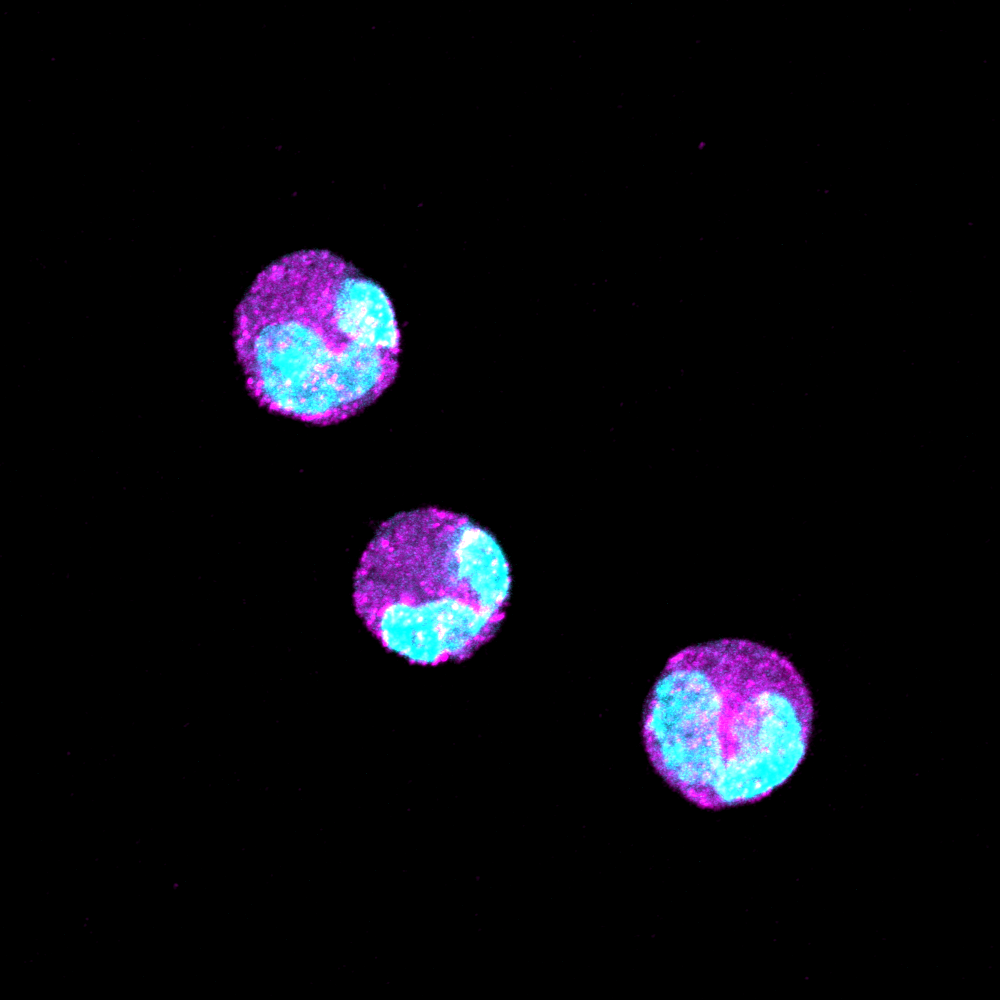

Supplement: Supplementary file 4 — Source data Fig. 1 [file 44319_2024_150_MOESM4_ESM.zip › Main Figure 1/Fig 1H/FB-168 images/mock narna+LL37/comp new.png]

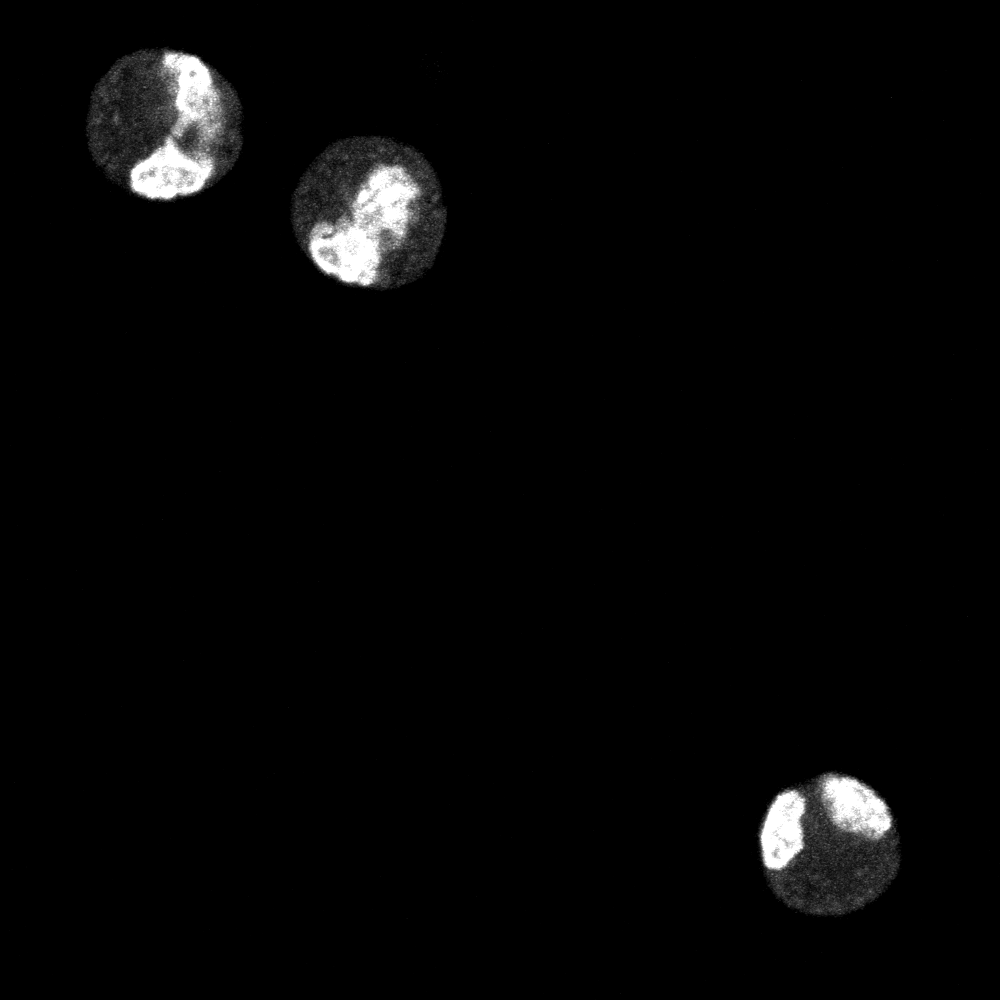

Supplement: Supplementary file 4 — Source data Fig. 1 [file 44319_2024_150_MOESM4_ESM.zip › Main Figure 1/Fig 1H/FB-168 images/pma narna/C2-MAX_Experiment-3517-Airyscan Processing-07.png]

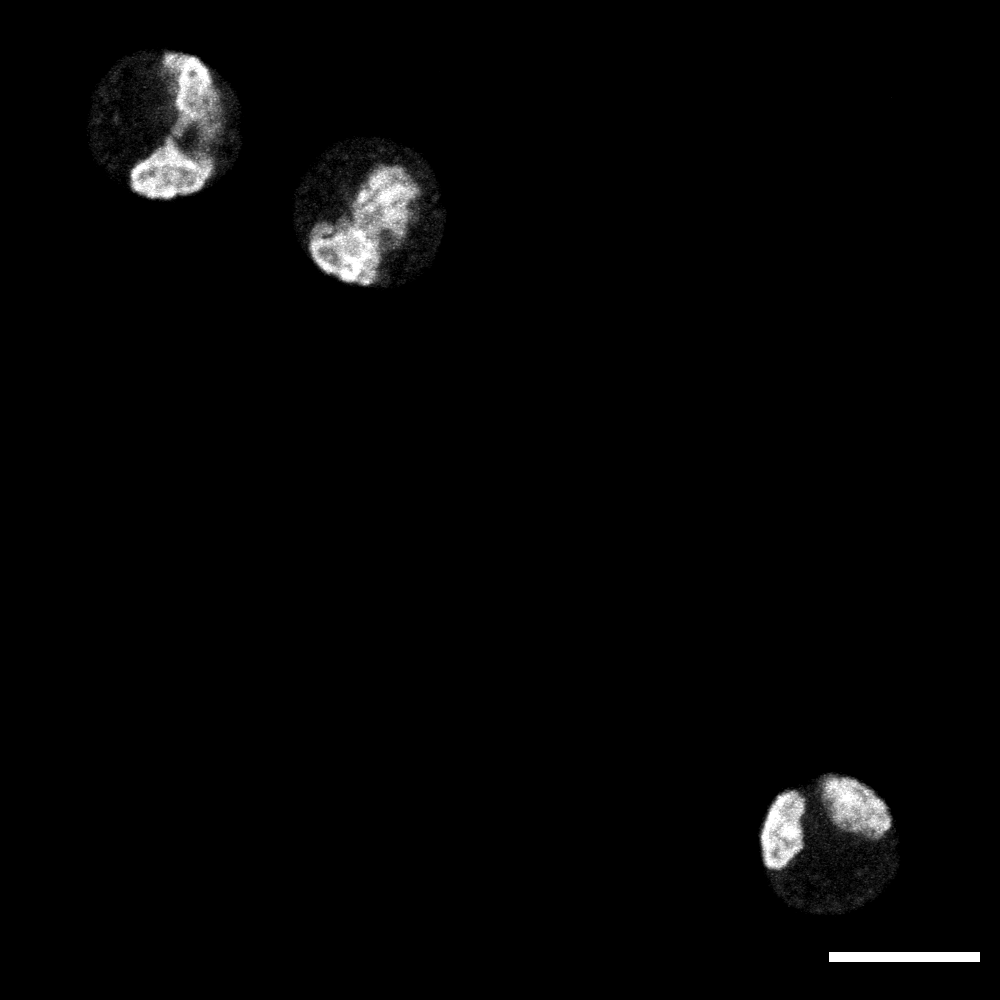

Supplement: Supplementary file 4 — Source data Fig. 1 [file 44319_2024_150_MOESM4_ESM.zip › Main Figure 1/Fig 1H/FB-168 images/pma narna/comp new scale bar.png]

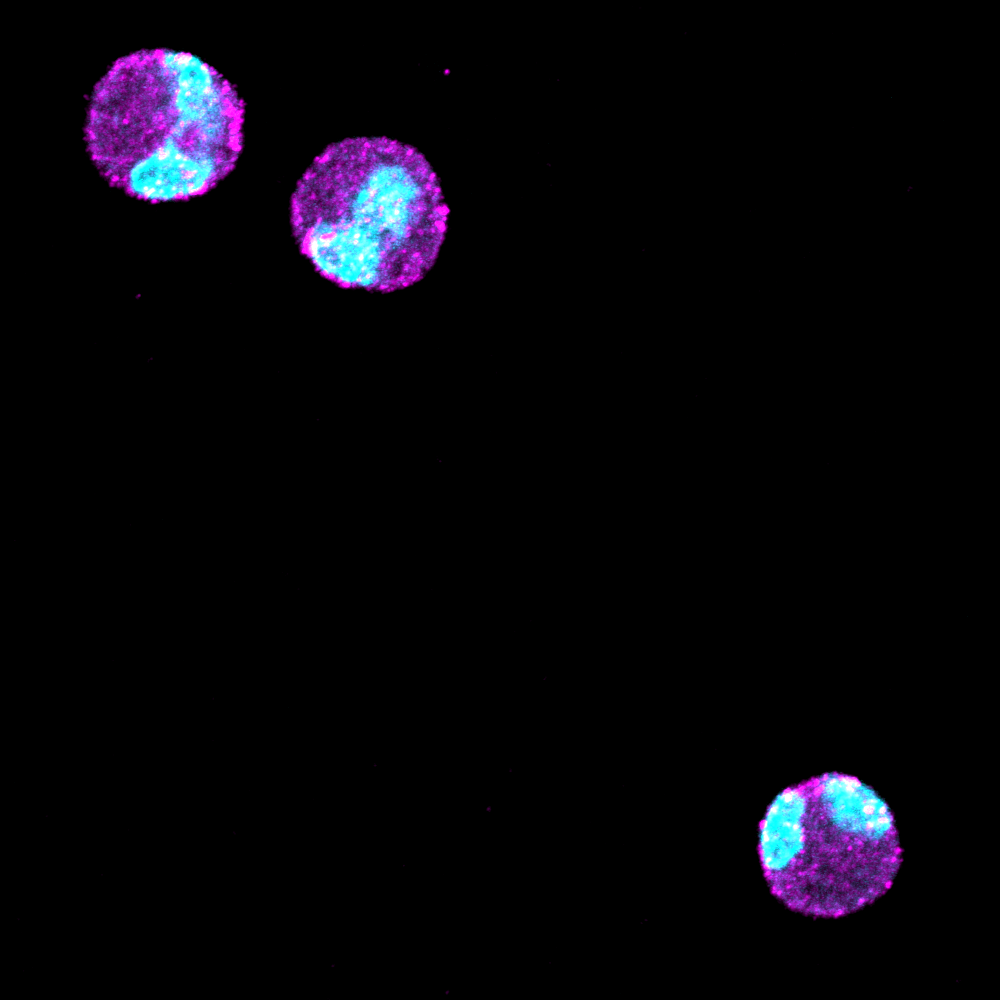

Supplement: Supplementary file 4 — Source data Fig. 1 [file 44319_2024_150_MOESM4_ESM.zip › Main Figure 1/Fig 1H/FB-168 images/pma narna/comp new.png]

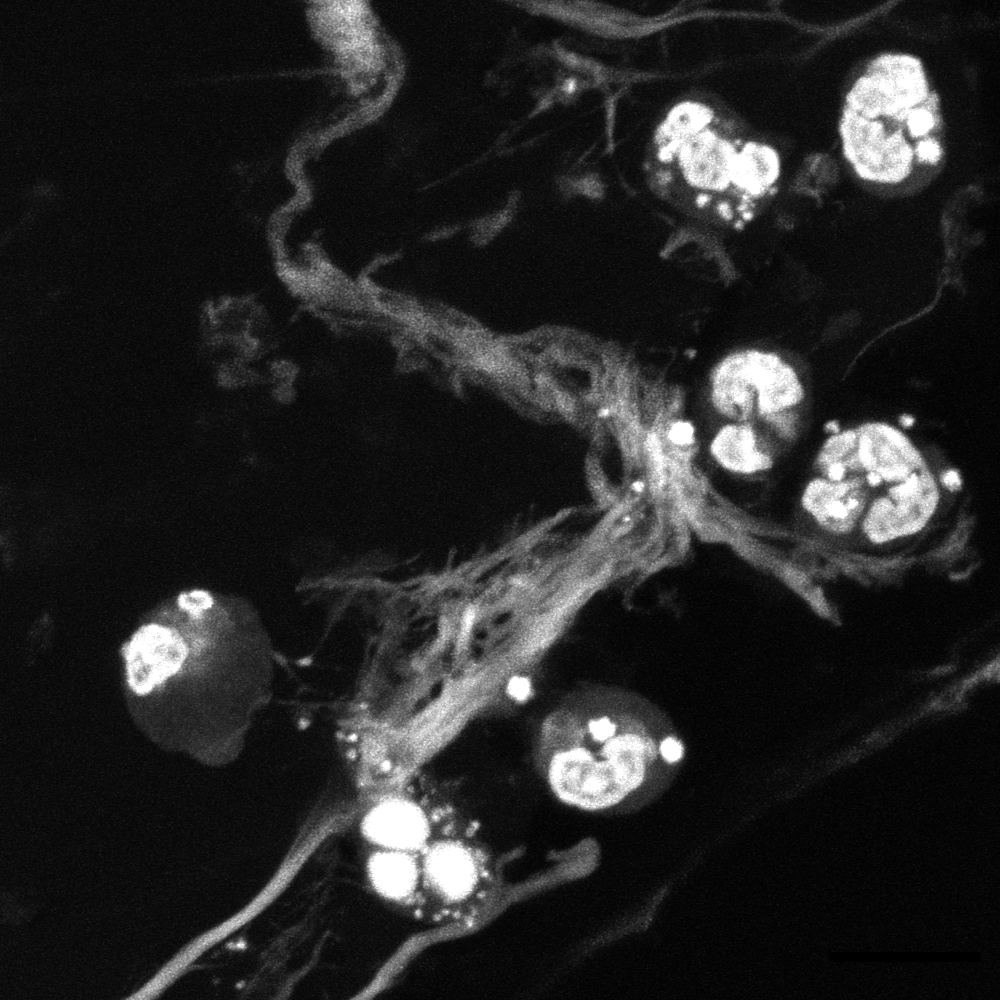

Supplement: Supplementary file 4 — Source data Fig. 1 [file 44319_2024_150_MOESM4_ESM.zip › Main Figure 1/Fig 1H/FB-168 images/pma narna+ll37/C2-MAX_Experiment-3518-Airyscan Processing-08.png]

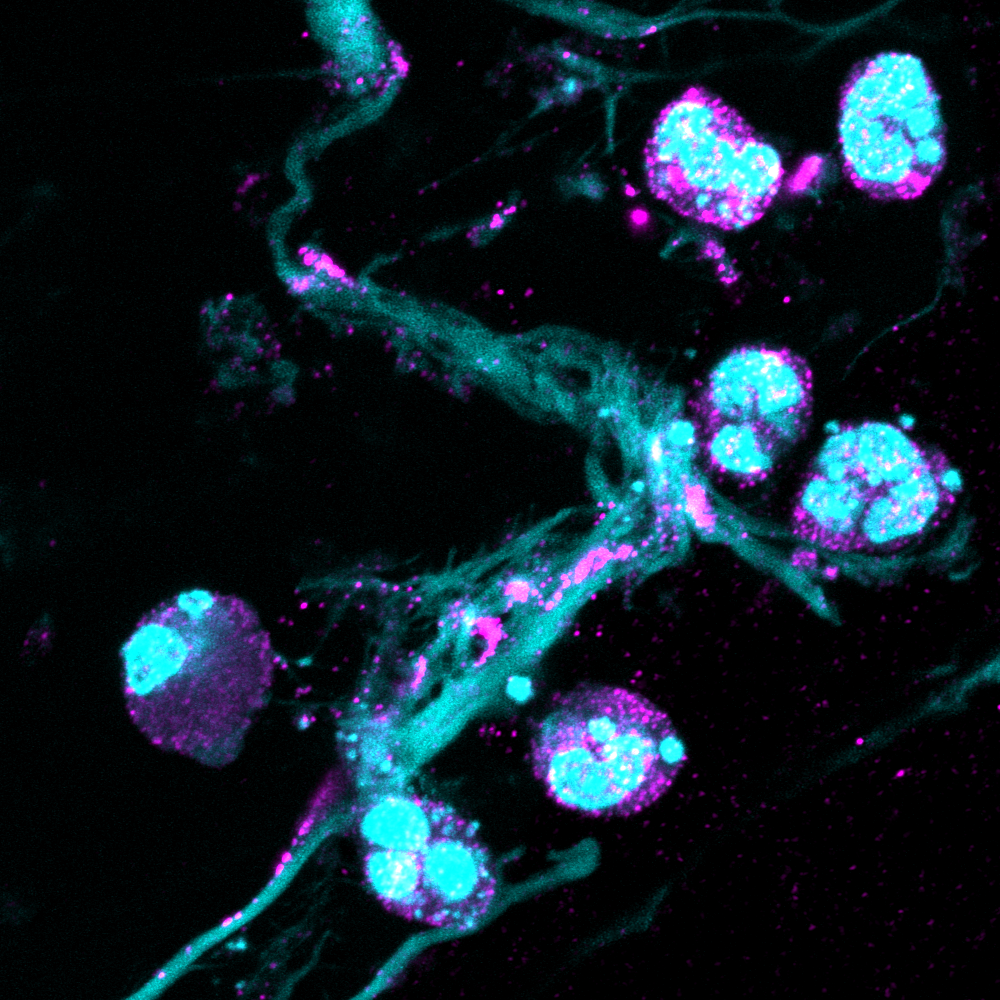

Supplement: Supplementary file 4 — Source data Fig. 1 [file 44319_2024_150_MOESM4_ESM.zip › Main Figure 1/Fig 1H/FB-168 images/pma narna+ll37/comp new.png]

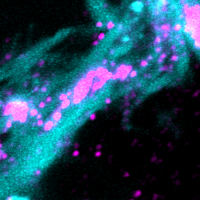

Supplement: Supplementary file 4 — Source data Fig. 1 [file 44319_2024_150_MOESM4_ESM.zip › Main Figure 1/Fig 1H/FB-168 images/pma narna+ll37/crop new.png]

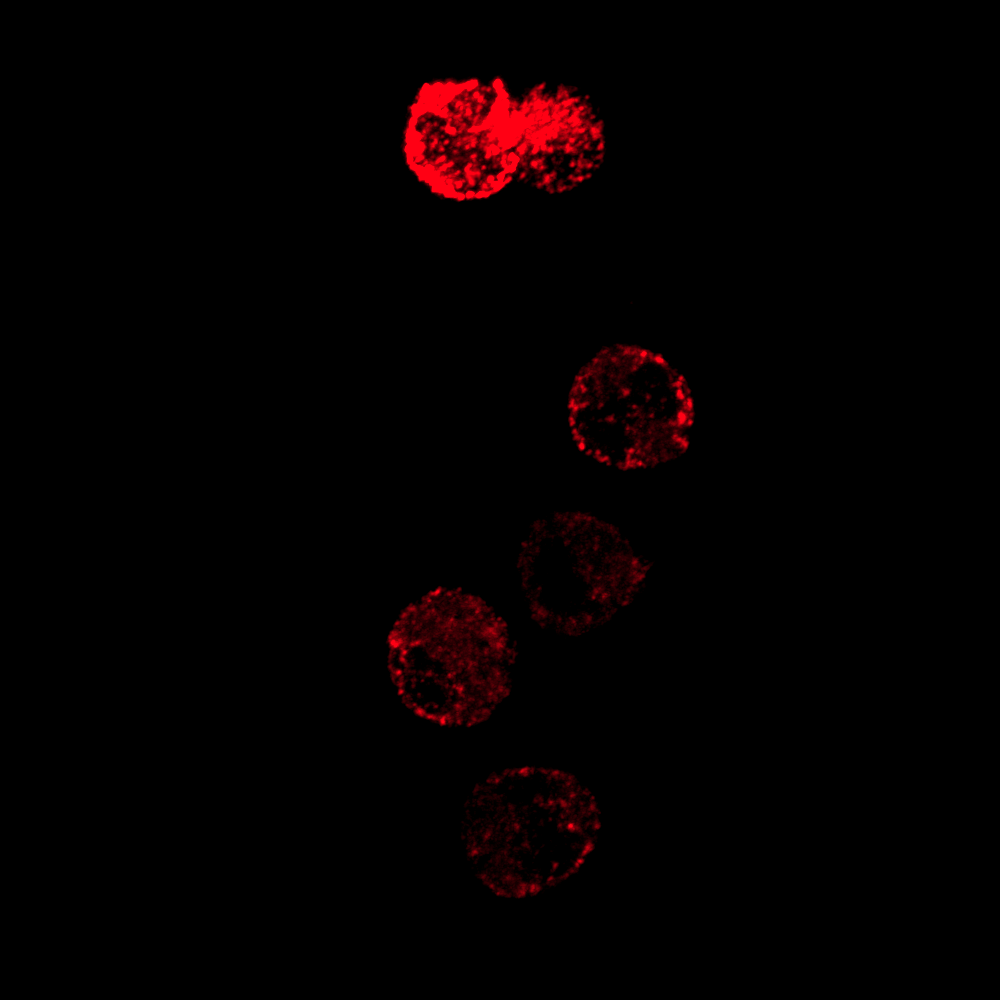

Supplement: Supplementary file 5 — Source data Fig. 2 [file 44319_2024_150_MOESM5_ESM.zip › Main Figure 2/Fig 2B/FB-175 images/trl8 inh/mock nets/C1-MAX_Experiment-3531-Airyscan Processing-21.png]

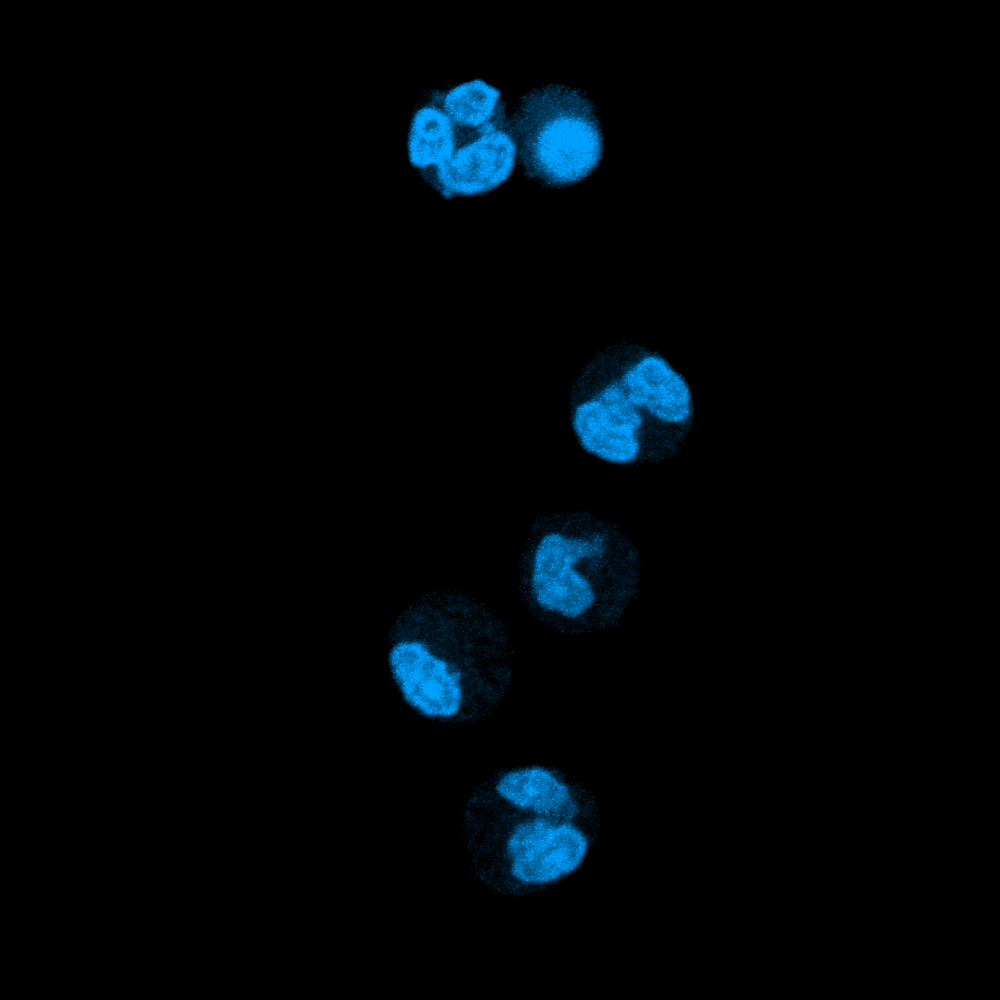

Supplement: Supplementary file 5 — Source data Fig. 2 [file 44319_2024_150_MOESM5_ESM.zip › Main Figure 2/Fig 2B/FB-175 images/trl8 inh/mock nets/C2-MAX_Experiment-3531-Airyscan Processing-21.png]

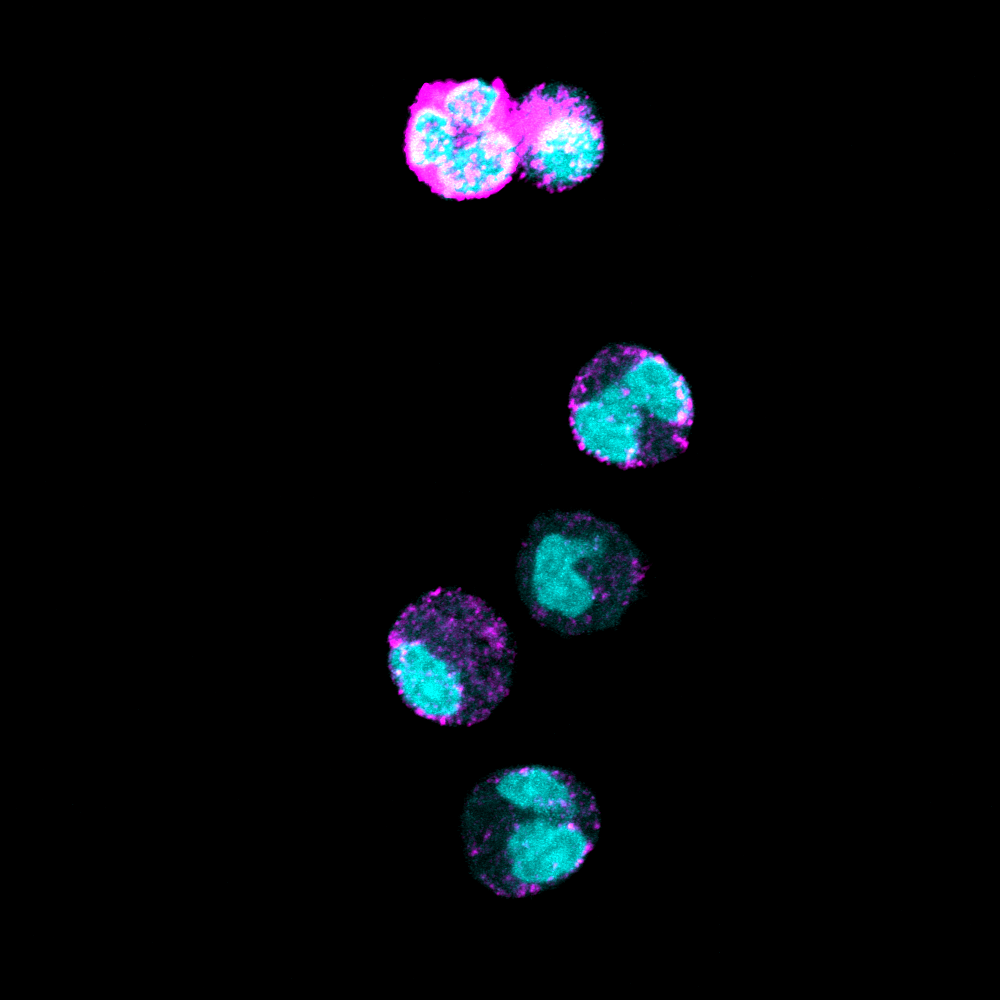

Supplement: Supplementary file 5 — Source data Fig. 2 [file 44319_2024_150_MOESM5_ESM.zip › Main Figure 2/Fig 2B/FB-175 images/trl8 inh/mock nets/comp new.png]

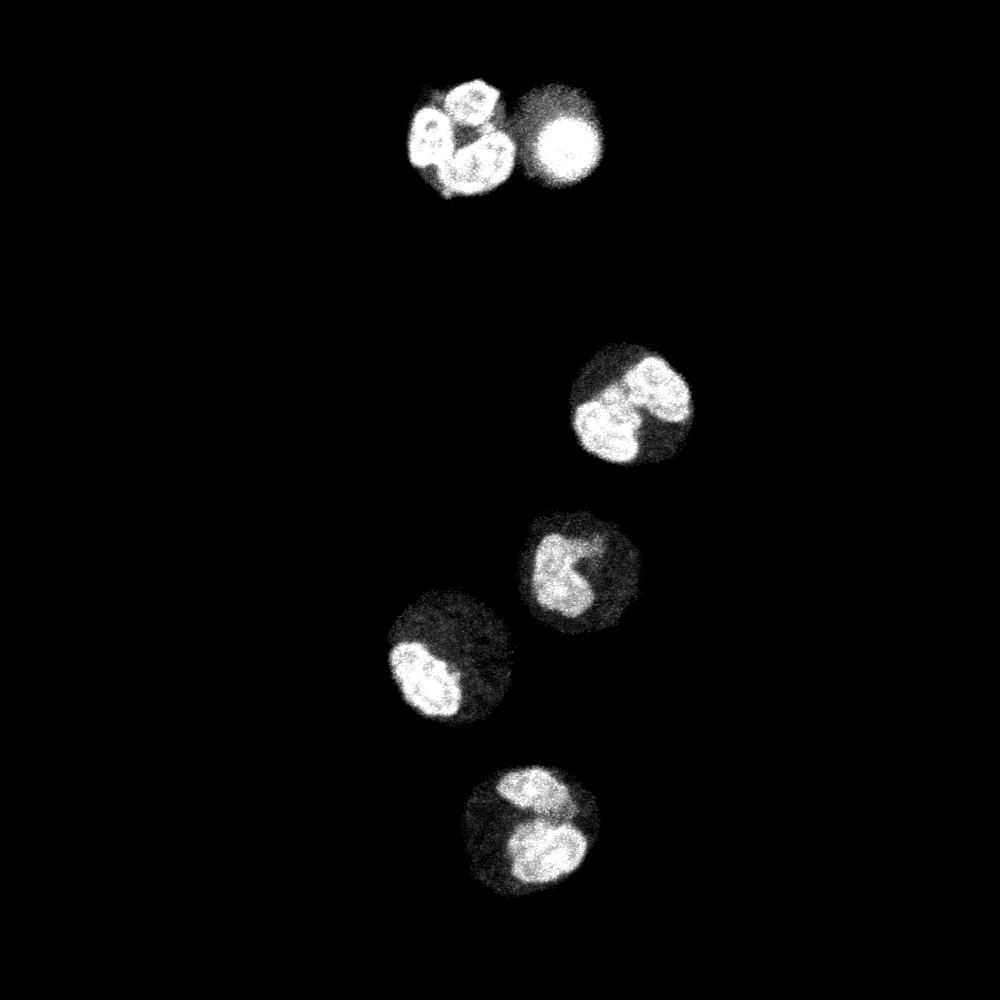

Supplement: Supplementary file 5 — Source data Fig. 2 [file 44319_2024_150_MOESM5_ESM.zip › Main Figure 2/Fig 2B/FB-175 images/trl8 inh/mock nets/gray.png]

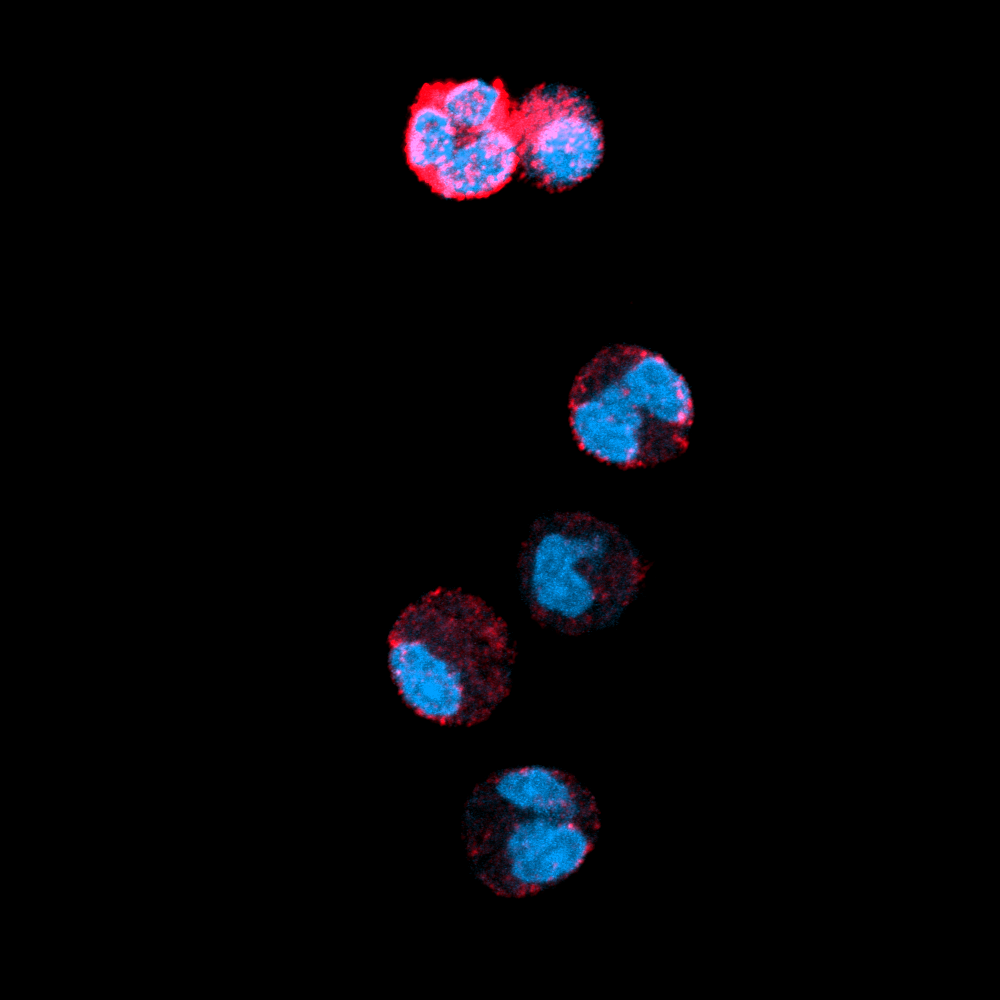

Supplement: Supplementary file 5 — Source data Fig. 2 [file 44319_2024_150_MOESM5_ESM.zip › Main Figure 2/Fig 2B/FB-175 images/trl8 inh/mock nets/MAX_Experiment-3531-Airyscan Processing-21.png]

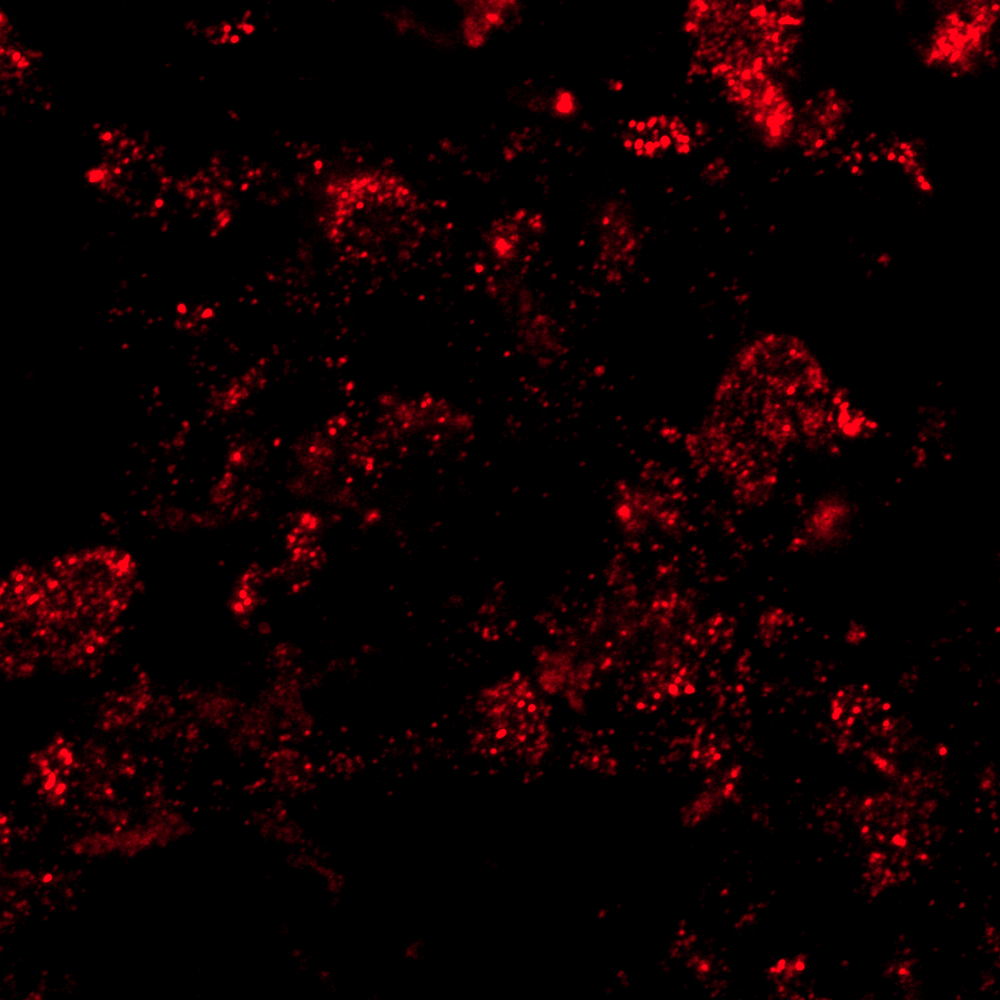

Supplement: Supplementary file 5 — Source data Fig. 2 [file 44319_2024_150_MOESM5_ESM.zip › Main Figure 2/Fig 2B/FB-175 images/trl8 inh/pma/C1-MAX_Experiment-3530-Airyscan Processing-20.png]

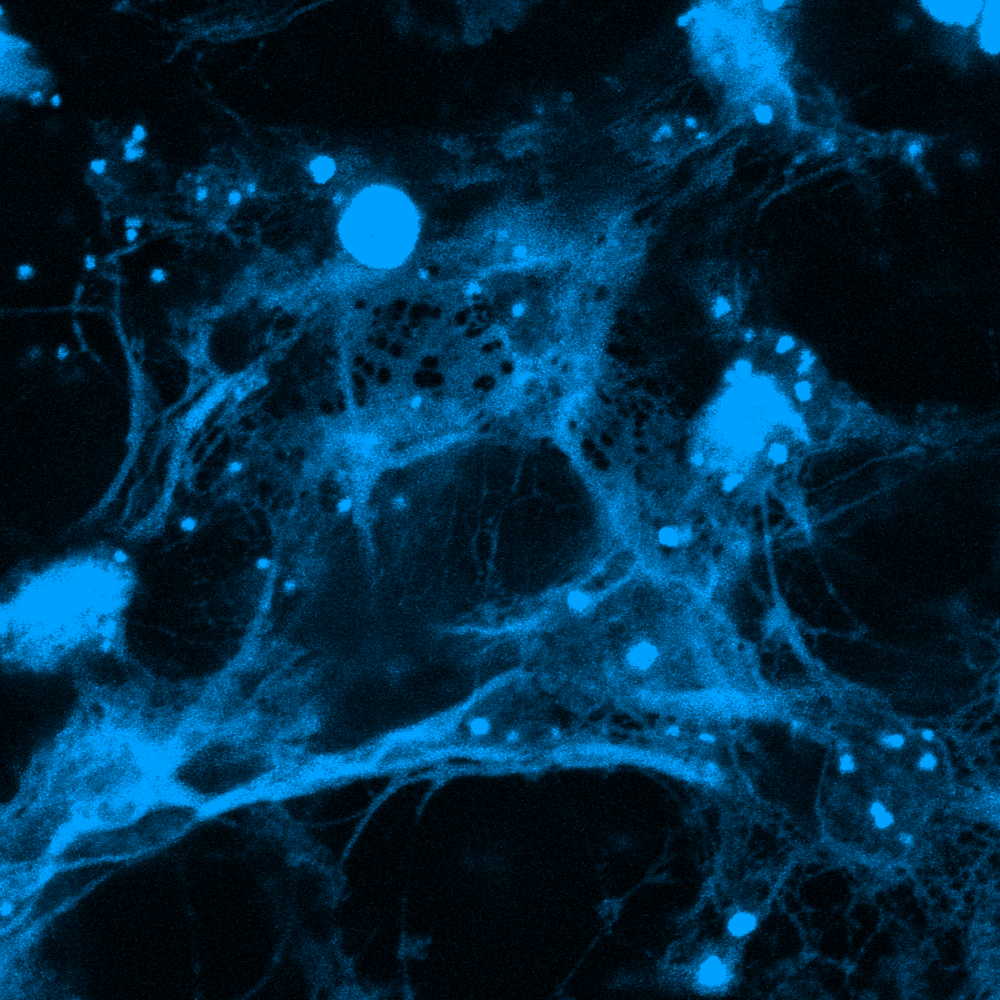

Supplement: Supplementary file 5 — Source data Fig. 2 [file 44319_2024_150_MOESM5_ESM.zip › Main Figure 2/Fig 2B/FB-175 images/trl8 inh/pma/C2-MAX_Experiment-3530-Airyscan Processing-20.png]

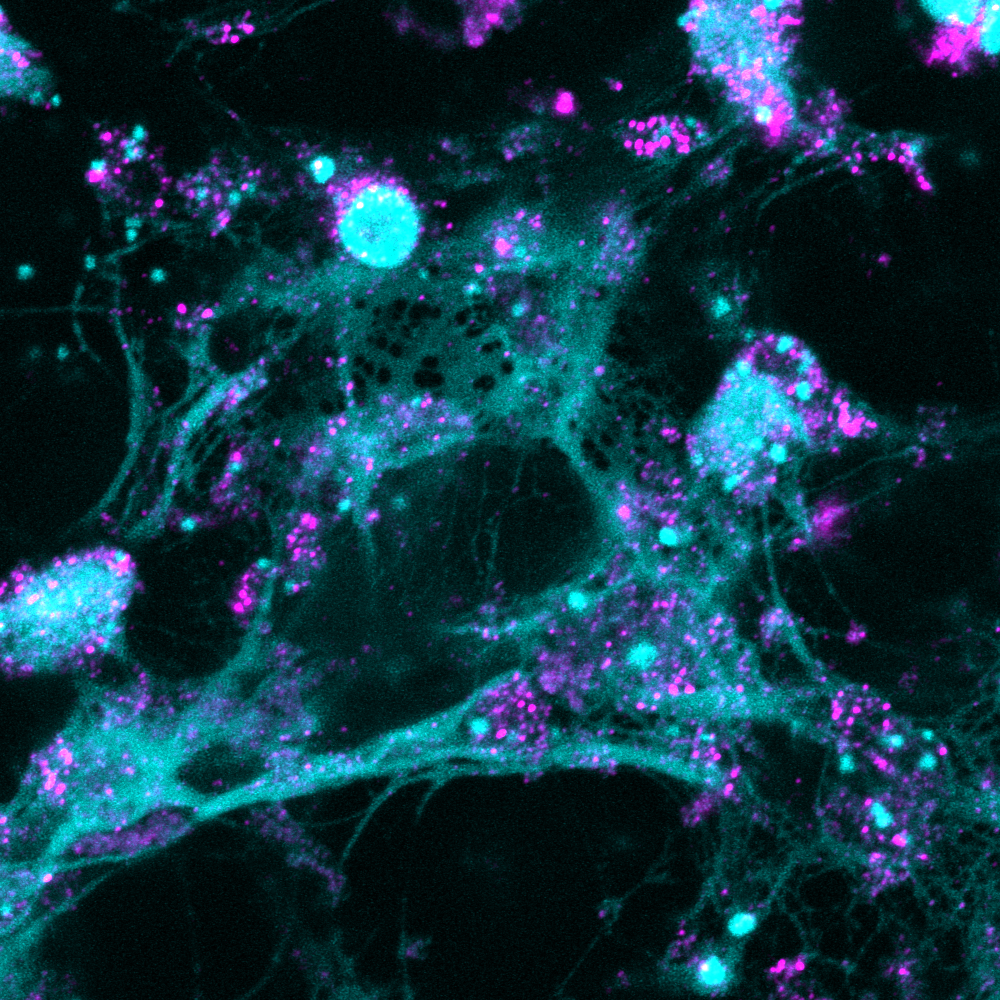

Supplement: Supplementary file 5 — Source data Fig. 2 [file 44319_2024_150_MOESM5_ESM.zip › Main Figure 2/Fig 2B/FB-175 images/trl8 inh/pma/comp new.png]

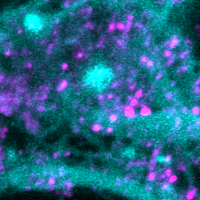

Supplement: Supplementary file 5 — Source data Fig. 2 [file 44319_2024_150_MOESM5_ESM.zip › Main Figure 2/Fig 2B/FB-175 images/trl8 inh/pma/crop new.png]

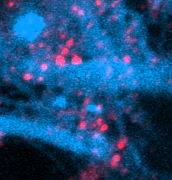

Supplement: Supplementary file 5 — Source data Fig. 2 [file 44319_2024_150_MOESM5_ESM.zip › Main Figure 2/Fig 2B/FB-175 images/trl8 inh/pma/crop.png]

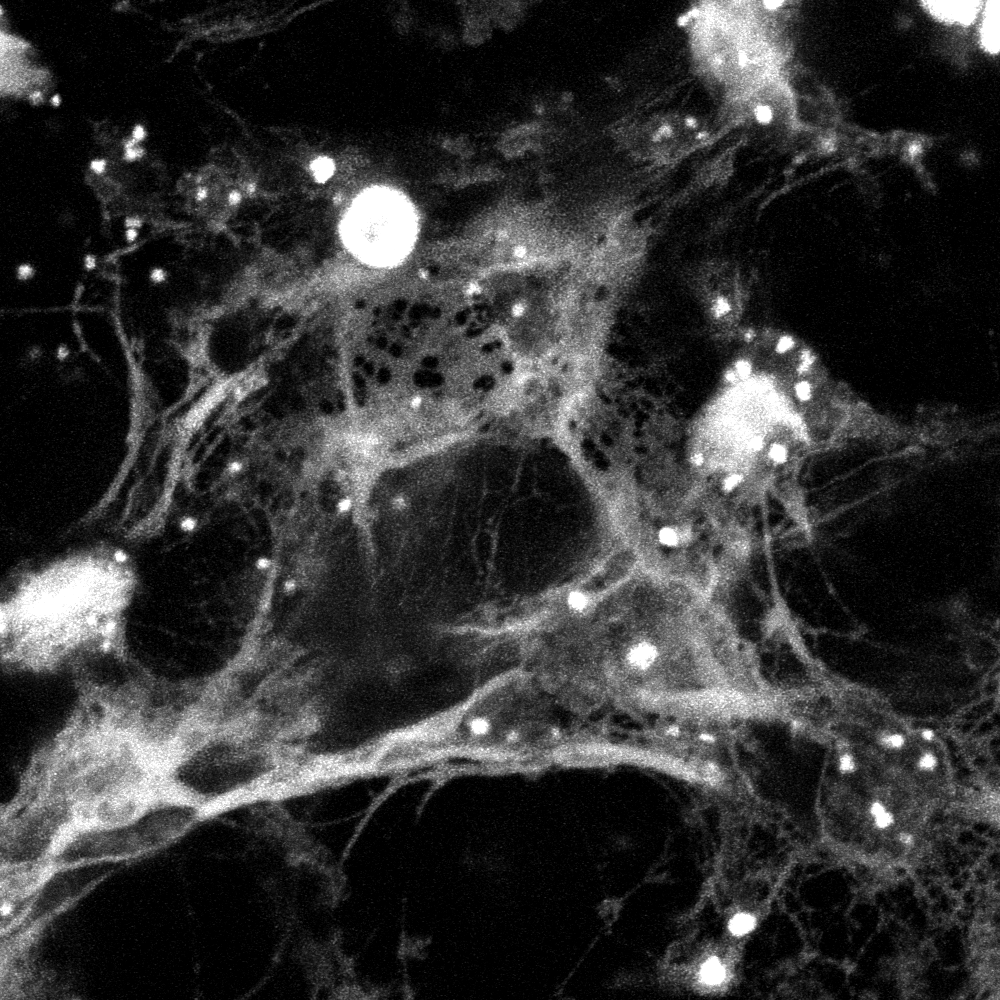

Supplement: Supplementary file 5 — Source data Fig. 2 [file 44319_2024_150_MOESM5_ESM.zip › Main Figure 2/Fig 2B/FB-175 images/trl8 inh/pma/gray.png]

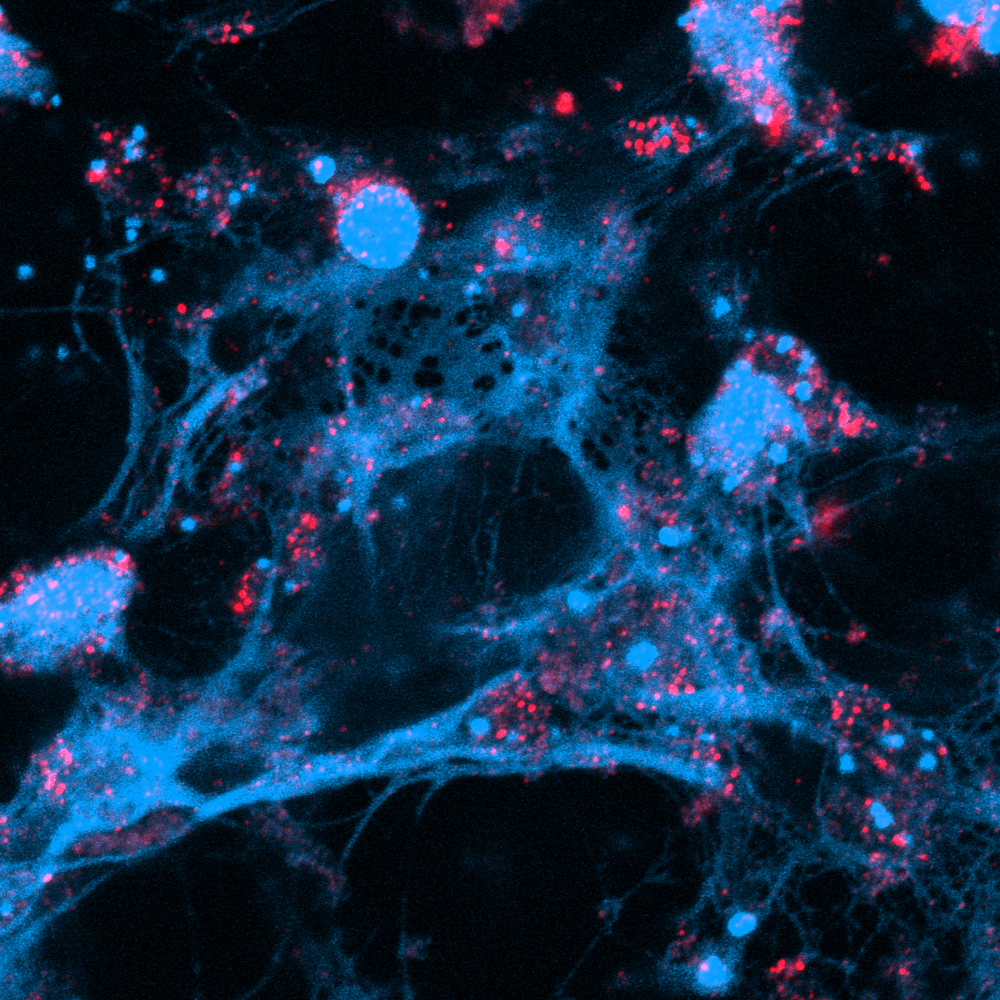

Supplement: Supplementary file 5 — Source data Fig. 2 [file 44319_2024_150_MOESM5_ESM.zip › Main Figure 2/Fig 2B/FB-175 images/trl8 inh/pma/MAX_Experiment-3530-Airyscan Processing-20.png]

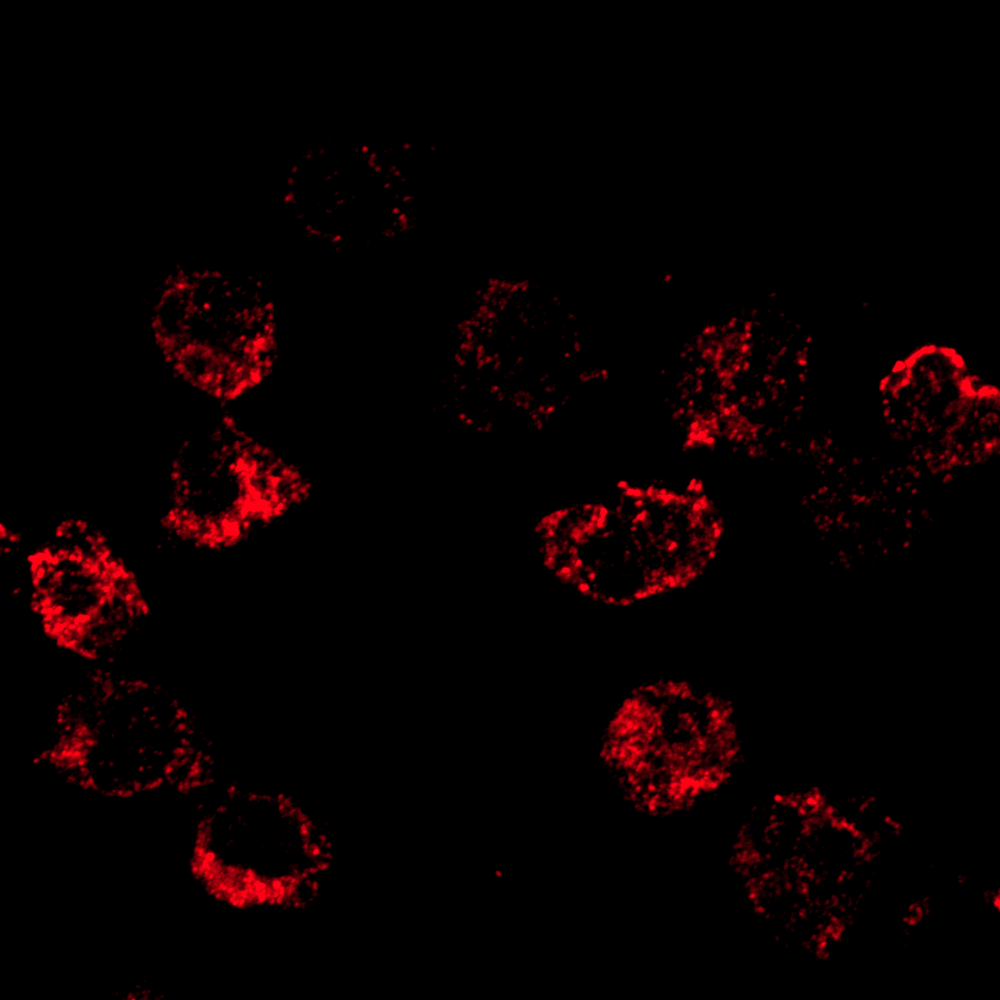

Supplement: Supplementary file 5 — Source data Fig. 2 [file 44319_2024_150_MOESM5_ESM.zip › Main Figure 2/Fig 2B/FB-175 images/trl8 inh/pma nets/C1-MAX_Experiment-3532-Airyscan Processing-22.png]

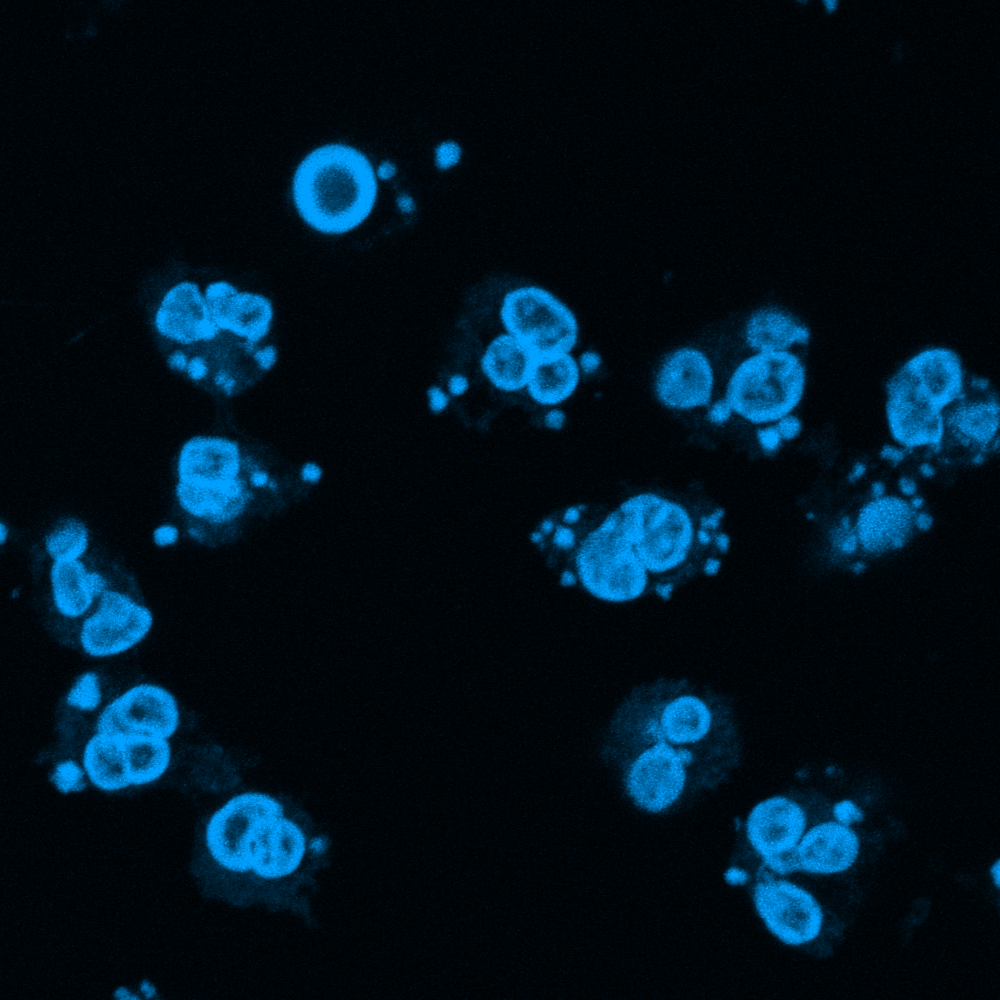

Supplement: Supplementary file 5 — Source data Fig. 2 [file 44319_2024_150_MOESM5_ESM.zip › Main Figure 2/Fig 2B/FB-175 images/trl8 inh/pma nets/C2-MAX_Experiment-3532-Airyscan Processing-22.png]

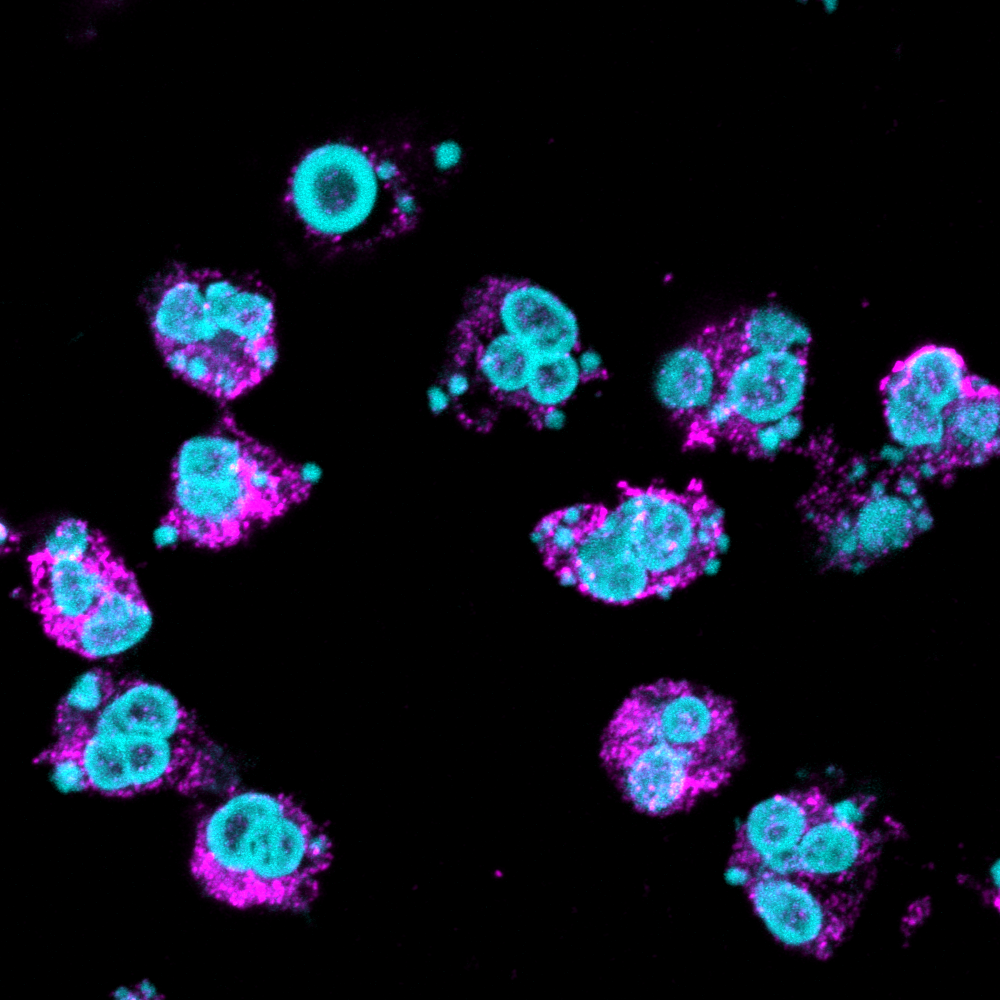

Supplement: Supplementary file 5 — Source data Fig. 2 [file 44319_2024_150_MOESM5_ESM.zip › Main Figure 2/Fig 2B/FB-175 images/trl8 inh/pma nets/comp new.png]

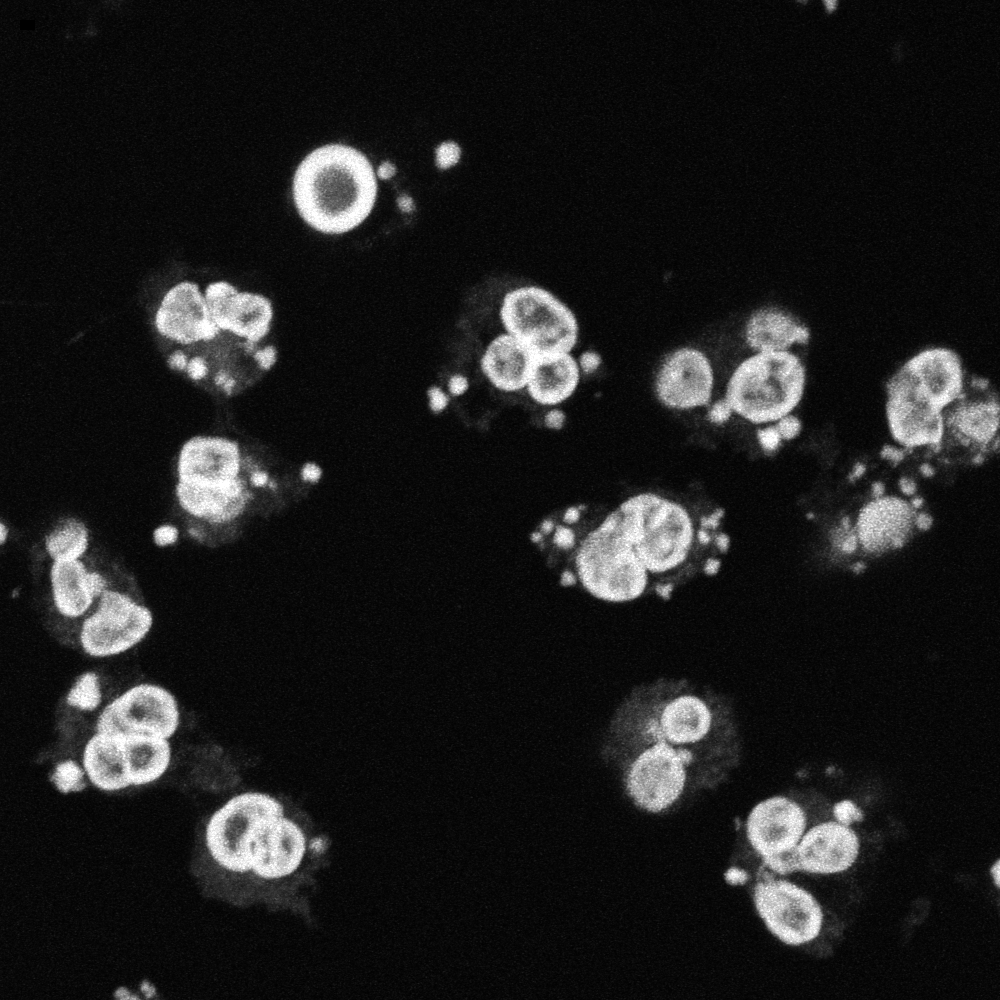

Supplement: Supplementary file 5 — Source data Fig. 2 [file 44319_2024_150_MOESM5_ESM.zip › Main Figure 2/Fig 2B/FB-175 images/trl8 inh/pma nets/gray.png]

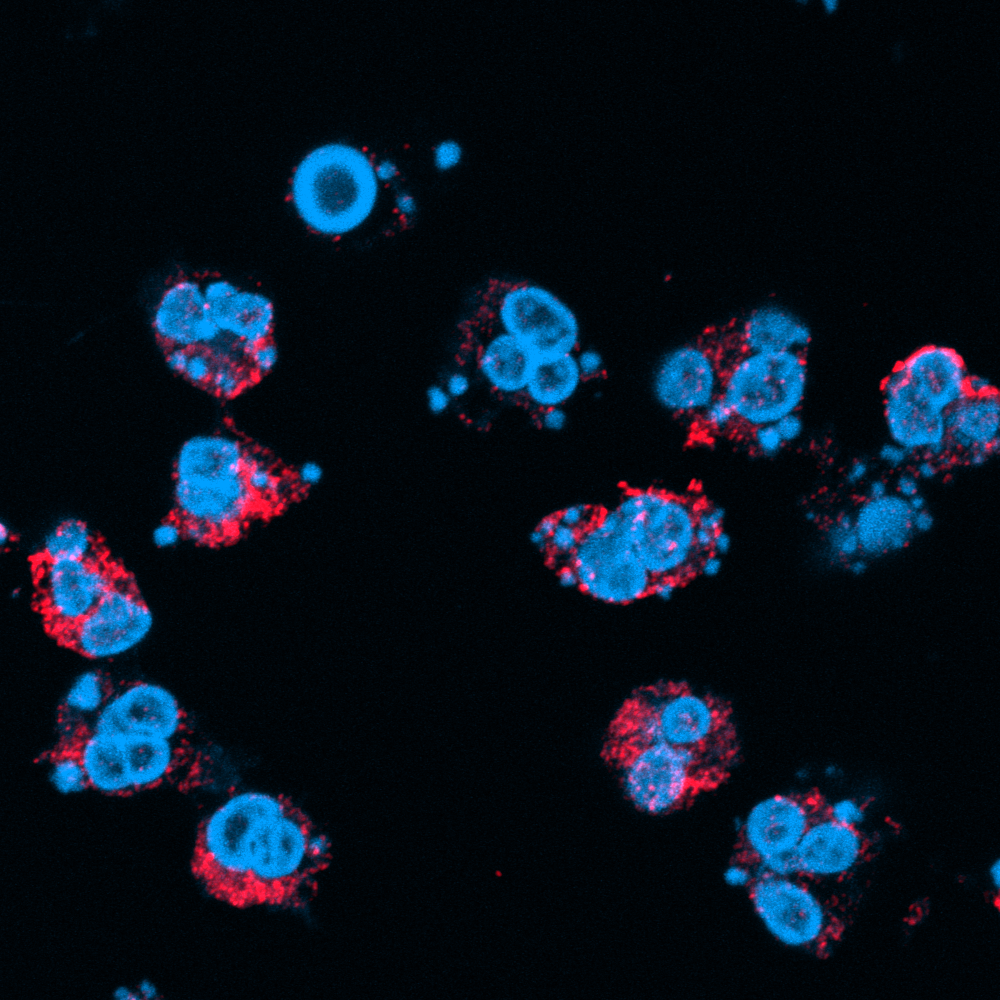

Supplement: Supplementary file 5 — Source data Fig. 2 [file 44319_2024_150_MOESM5_ESM.zip › Main Figure 2/Fig 2B/FB-175 images/trl8 inh/pma nets/MAX_Experiment-3532-Airyscan Processing-22.png]

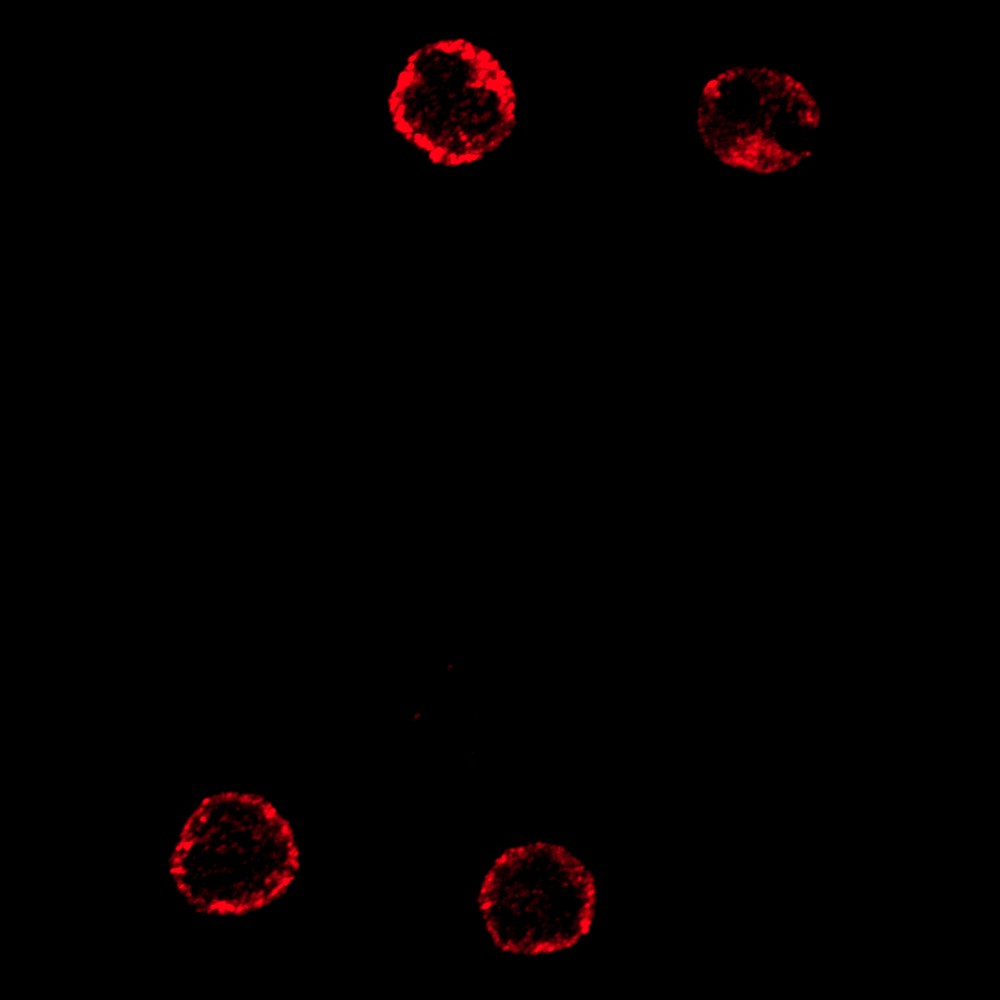

Supplement: Supplementary file 5 — Source data Fig. 2 [file 44319_2024_150_MOESM5_ESM.zip › Main Figure 2/Fig 2B/FB-175 images/trl8 inh/ssrna ll37/C1-MAX_Experiment-3533-Airyscan Processing-23.png]

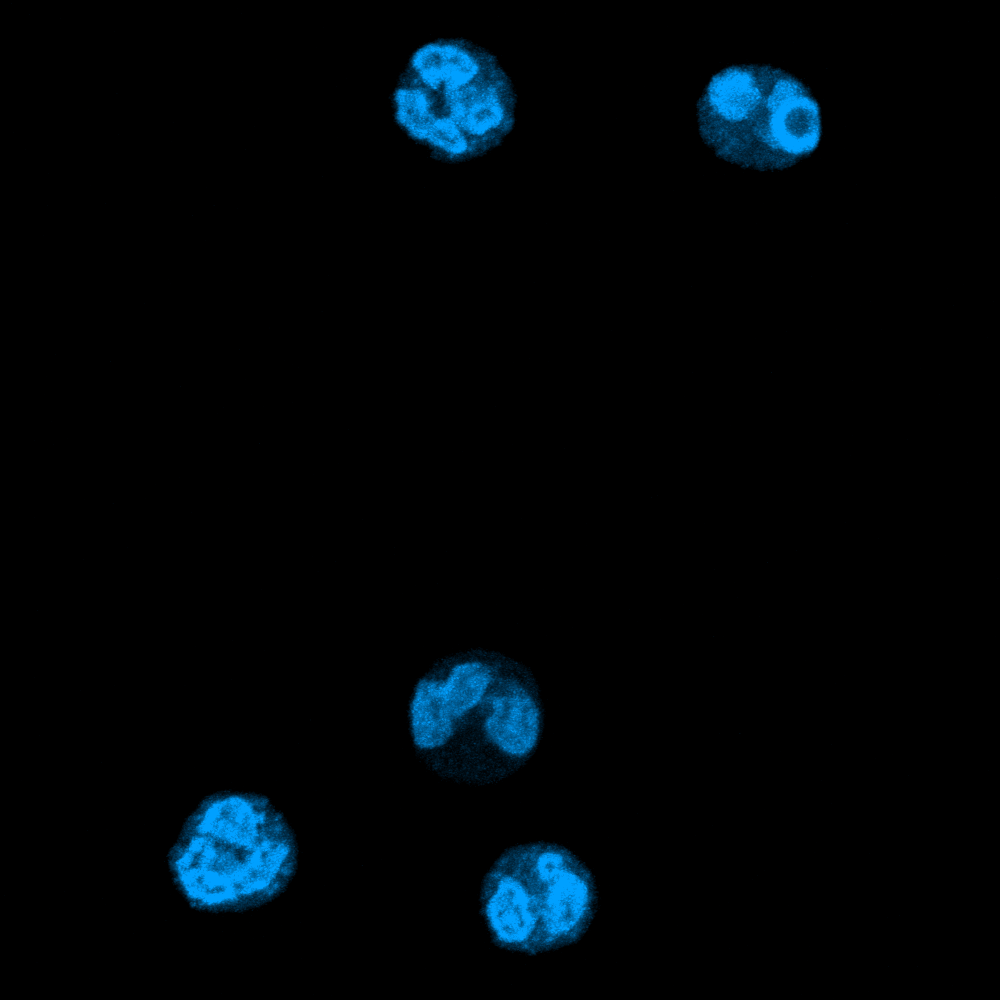

Supplement: Supplementary file 5 — Source data Fig. 2 [file 44319_2024_150_MOESM5_ESM.zip › Main Figure 2/Fig 2B/FB-175 images/trl8 inh/ssrna ll37/C2-MAX_Experiment-3533-Airyscan Processing-23.png]

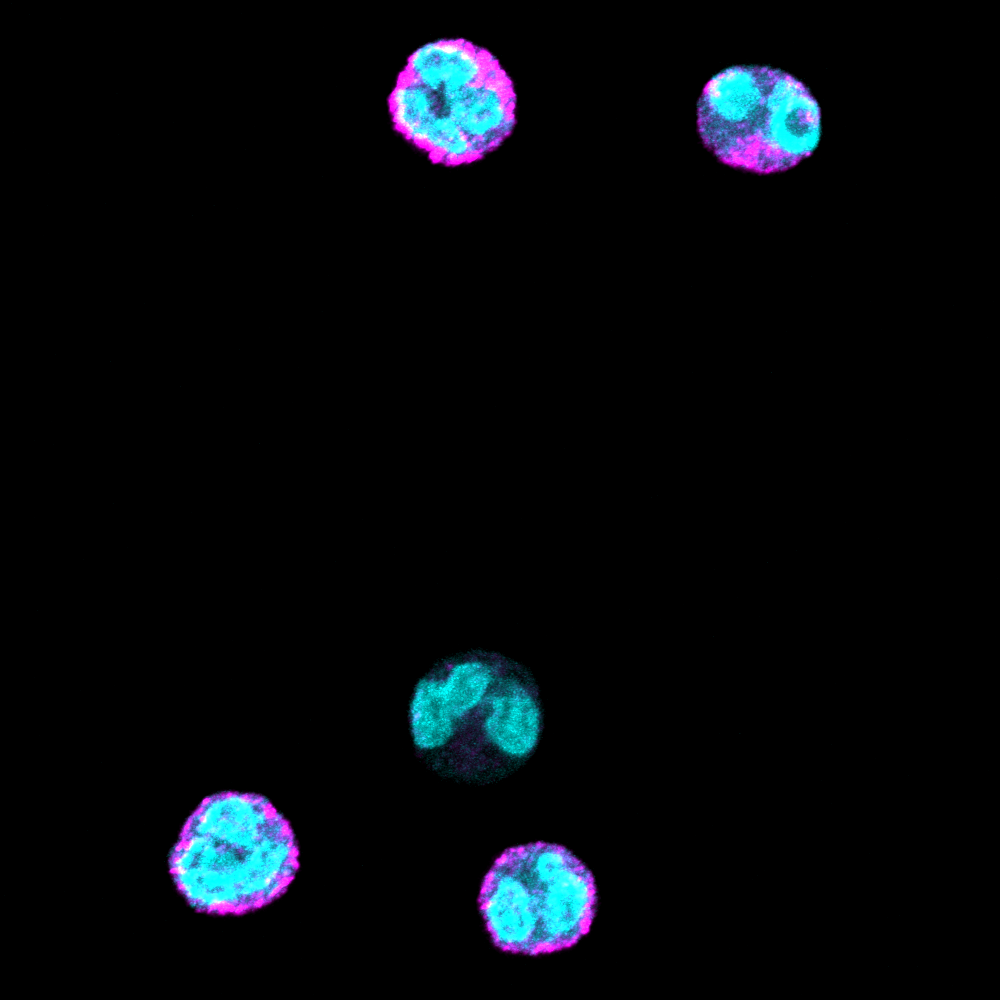

Supplement: Supplementary file 5 — Source data Fig. 2 [file 44319_2024_150_MOESM5_ESM.zip › Main Figure 2/Fig 2B/FB-175 images/trl8 inh/ssrna ll37/comp new.png]

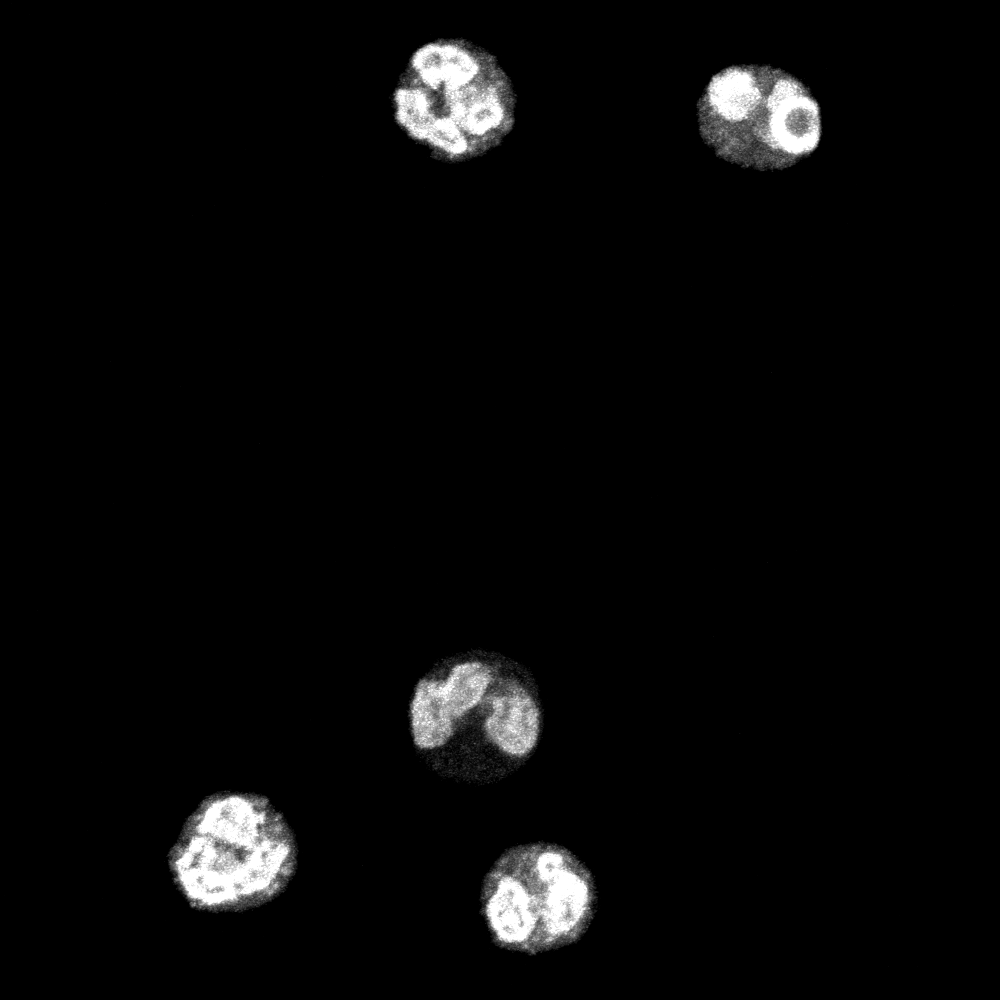

Supplement: Supplementary file 5 — Source data Fig. 2 [file 44319_2024_150_MOESM5_ESM.zip › Main Figure 2/Fig 2B/FB-175 images/trl8 inh/ssrna ll37/gray.png]

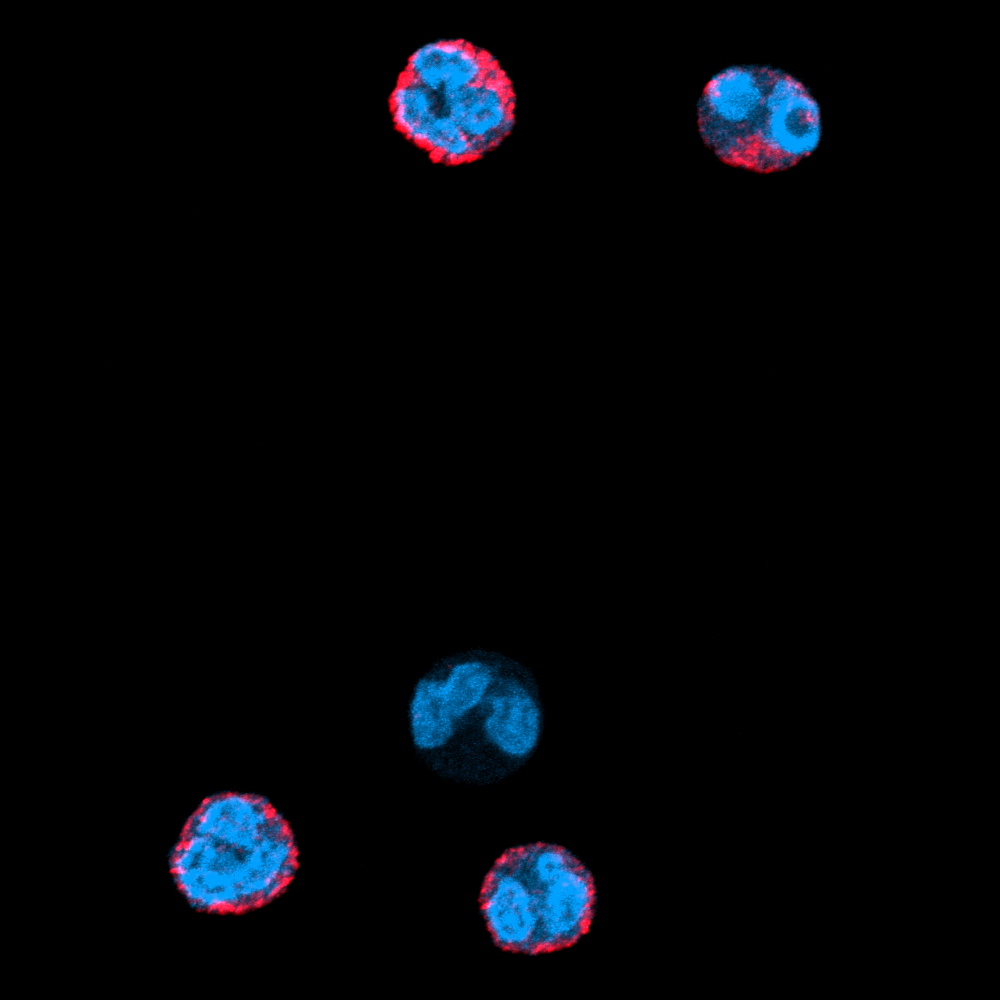

Supplement: Supplementary file 5 — Source data Fig. 2 [file 44319_2024_150_MOESM5_ESM.zip › Main Figure 2/Fig 2B/FB-175 images/trl8 inh/ssrna ll37/MAX_Experiment-3533-Airyscan Processing-23.png]

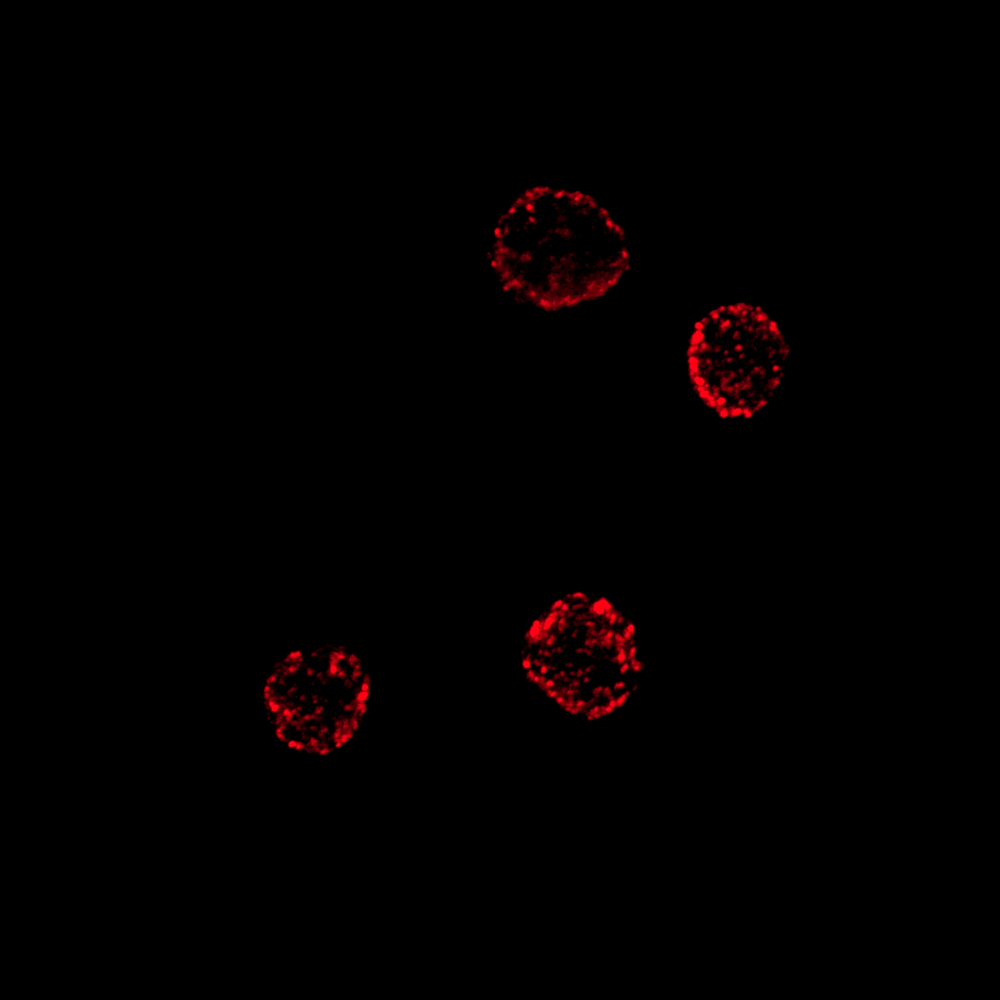

Supplement: Supplementary file 5 — Source data Fig. 2 [file 44319_2024_150_MOESM5_ESM.zip › Main Figure 2/Fig 2B/FB-175 images/trl8 inh/unstimulated/C1-MAX_Experiment-3529-Airyscan Processing-19.png]

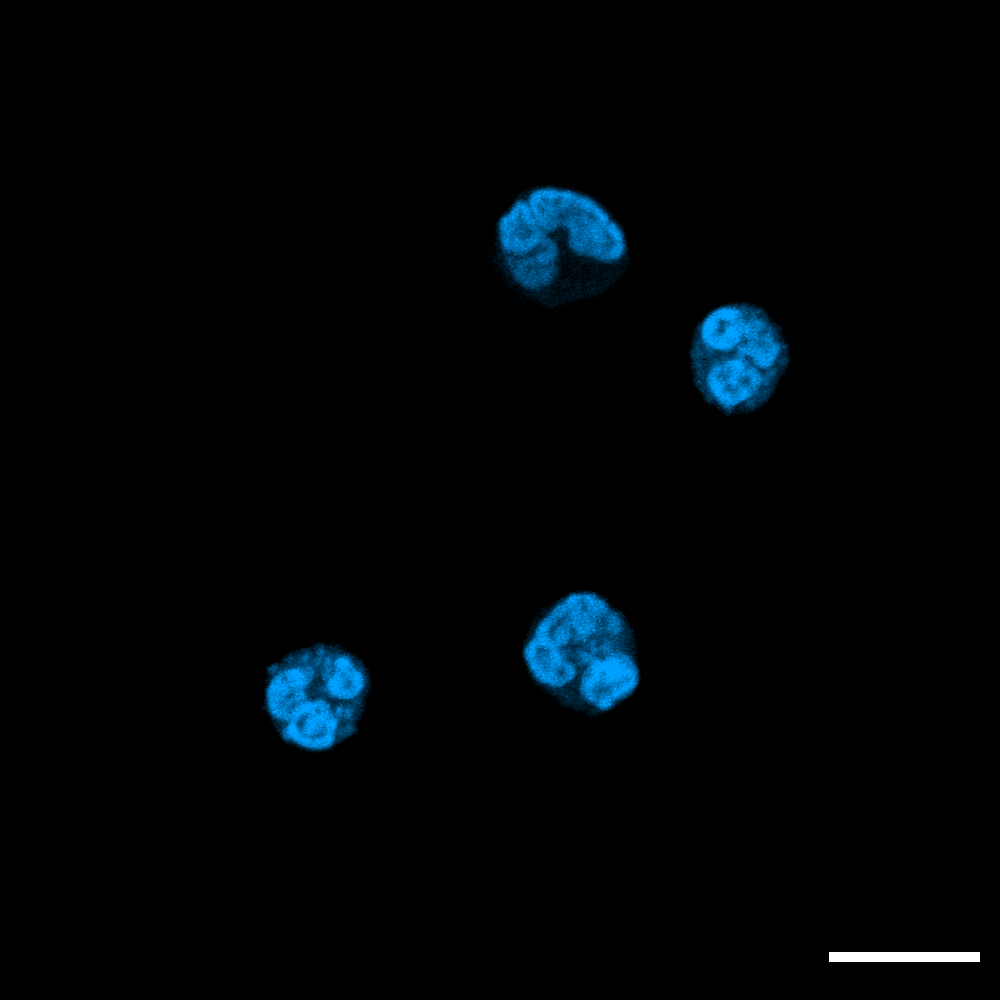

Supplement: Supplementary file 5 — Source data Fig. 2 [file 44319_2024_150_MOESM5_ESM.zip › Main Figure 2/Fig 2B/FB-175 images/trl8 inh/unstimulated/C2-MAX_Experiment-3529-Airyscan Processing-19.png]

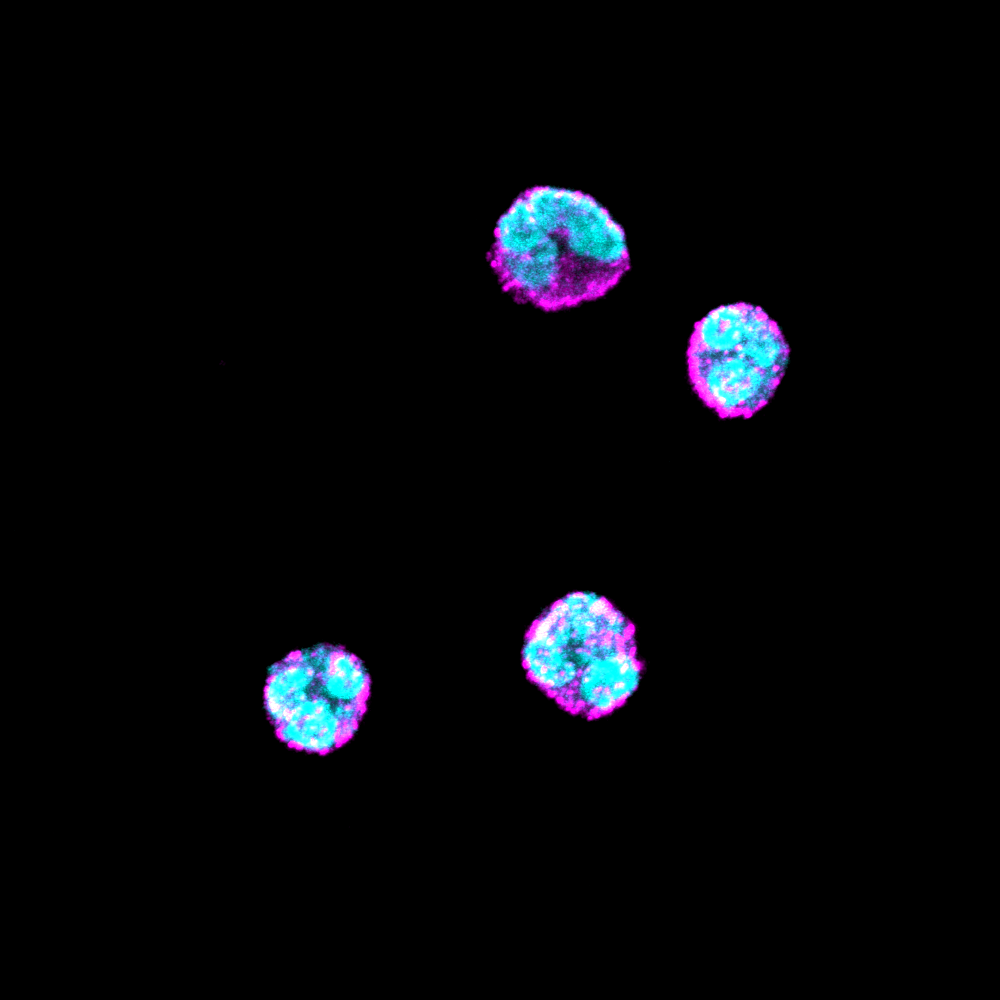

Supplement: Supplementary file 5 — Source data Fig. 2 [file 44319_2024_150_MOESM5_ESM.zip › Main Figure 2/Fig 2B/FB-175 images/trl8 inh/unstimulated/comp new.png]

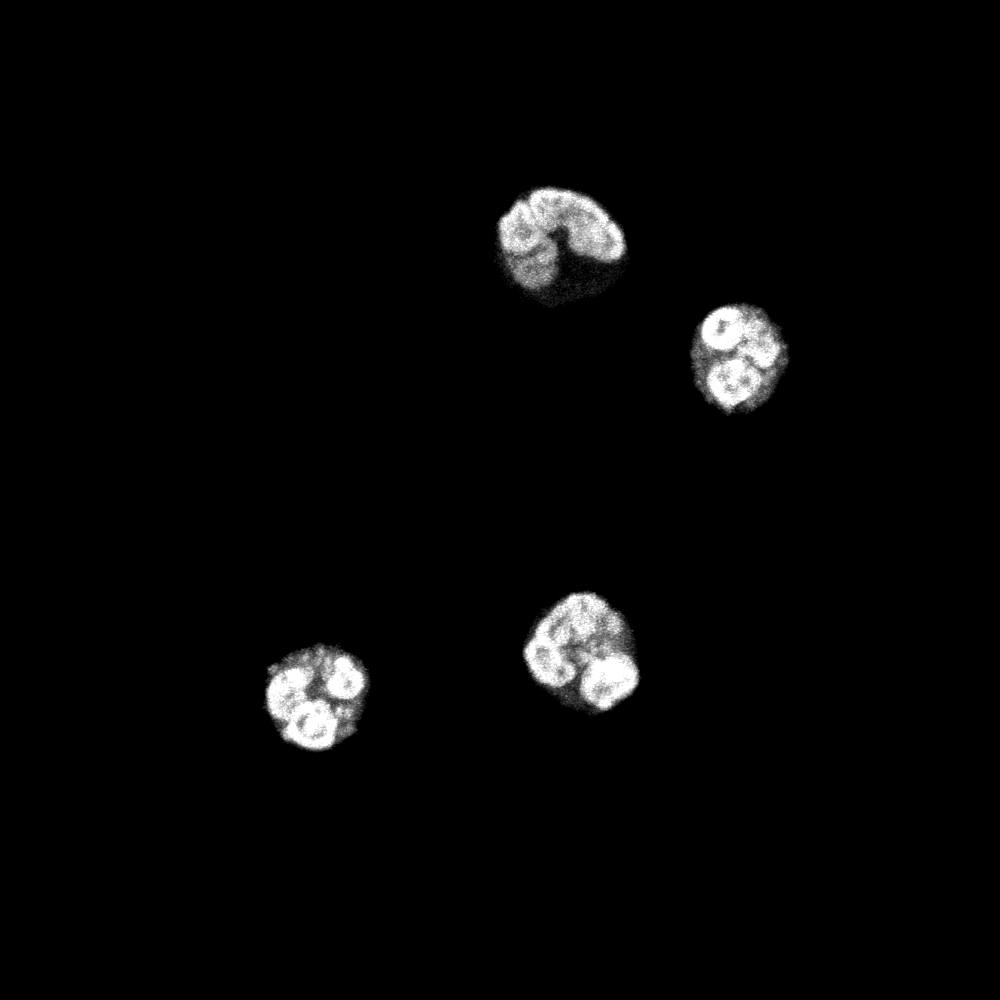

Supplement: Supplementary file 5 — Source data Fig. 2 [file 44319_2024_150_MOESM5_ESM.zip › Main Figure 2/Fig 2B/FB-175 images/trl8 inh/unstimulated/gray.png]

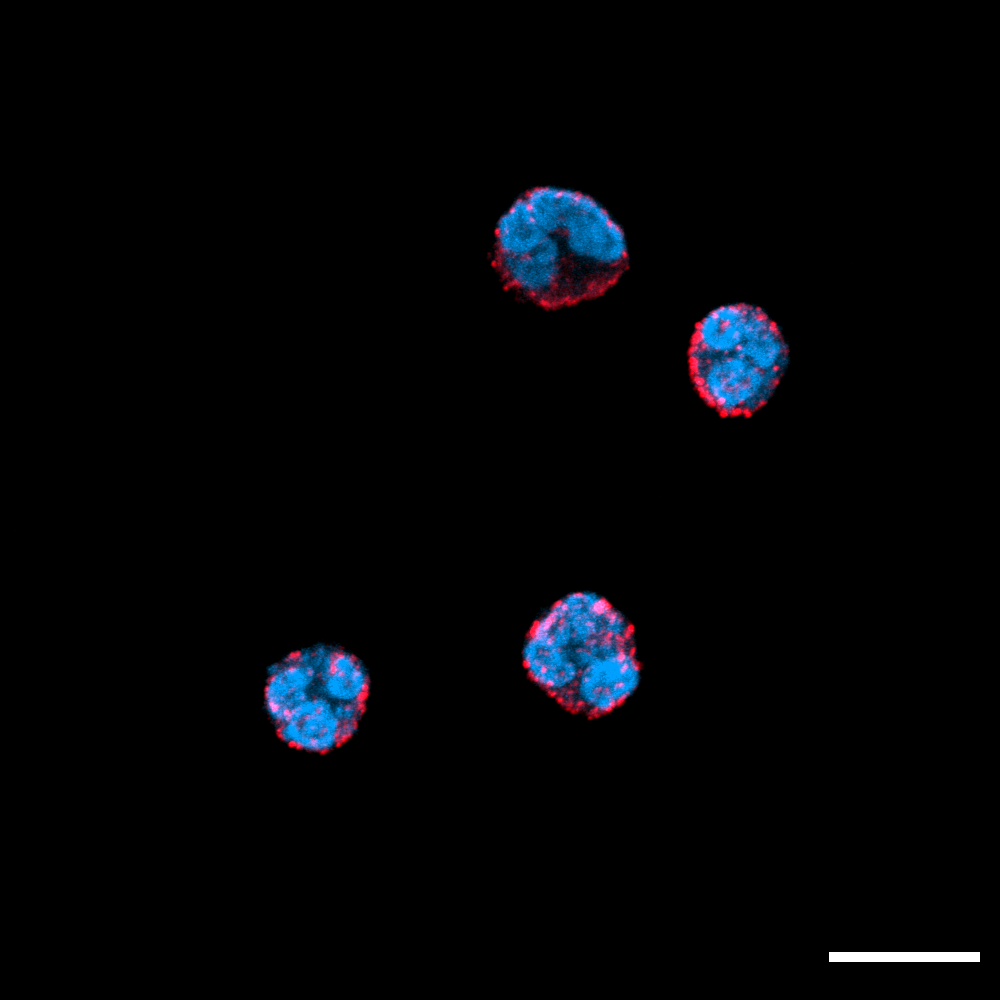

Supplement: Supplementary file 5 — Source data Fig. 2 [file 44319_2024_150_MOESM5_ESM.zip › Main Figure 2/Fig 2B/FB-175 images/trl8 inh/unstimulated/MAX_Experiment-3529-Airyscan Processing-19 scale bar.png]

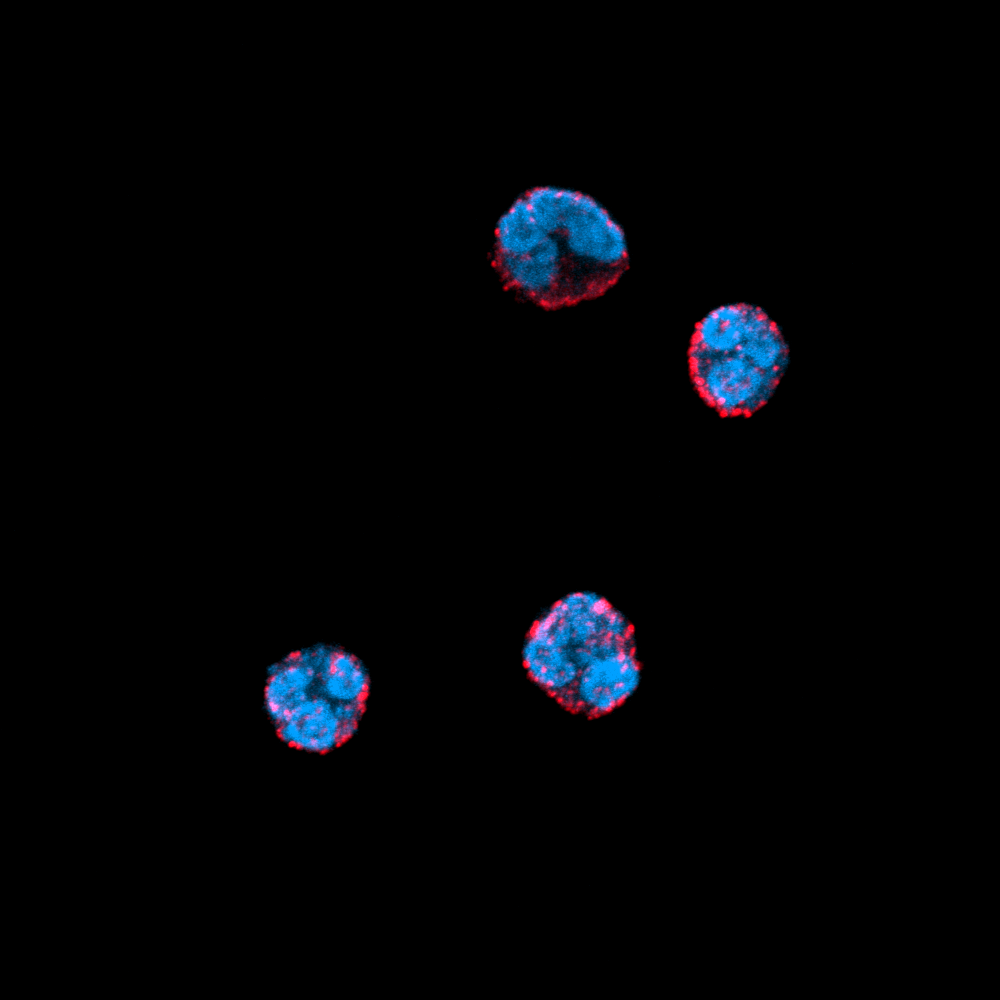

Supplement: Supplementary file 5 — Source data Fig. 2 [file 44319_2024_150_MOESM5_ESM.zip › Main Figure 2/Fig 2B/FB-175 images/trl8 inh/unstimulated/MAX_Experiment-3529-Airyscan Processing-19.png]

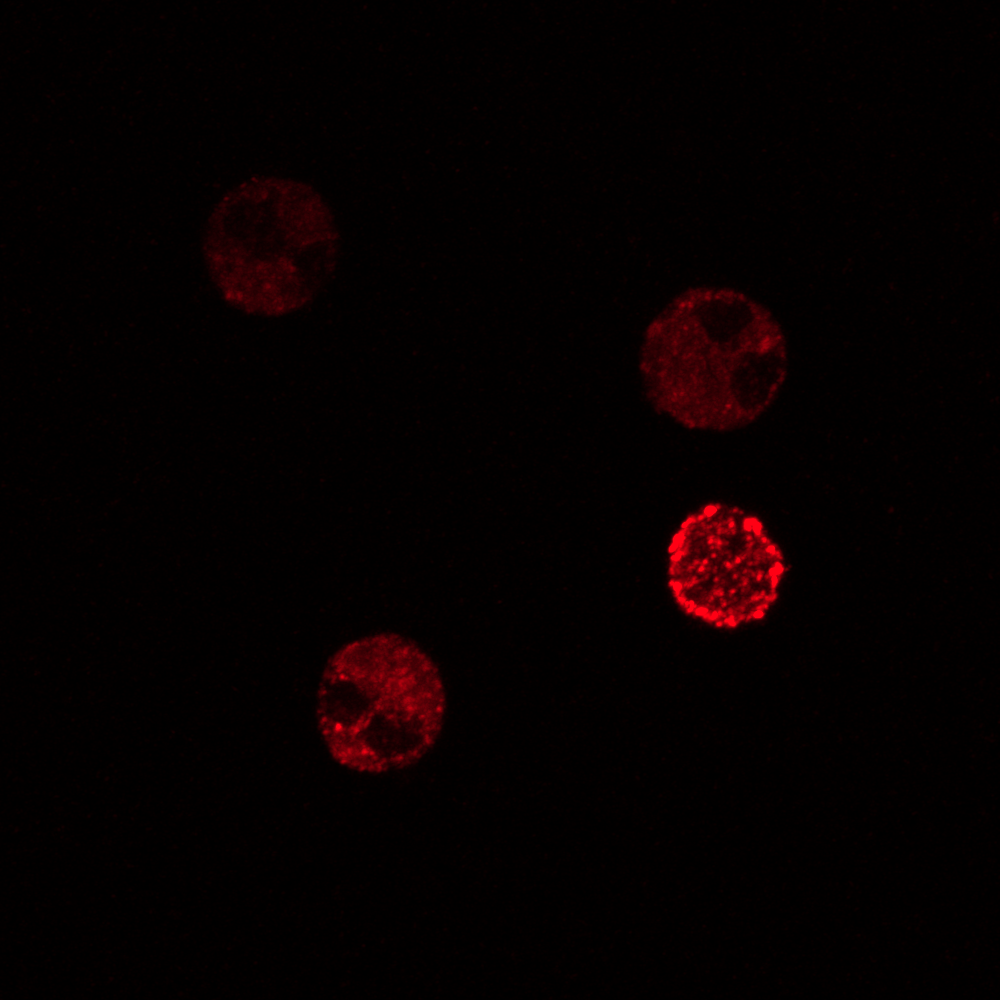

Supplement: Supplementary file 5 — Source data Fig. 2 [file 44319_2024_150_MOESM5_ESM.zip › Main Figure 2/Fig 2B/FB-175 images/untreated/mock nets 1_100/C1-MAX_Experiment-3521-Airyscan Processing-11.png]

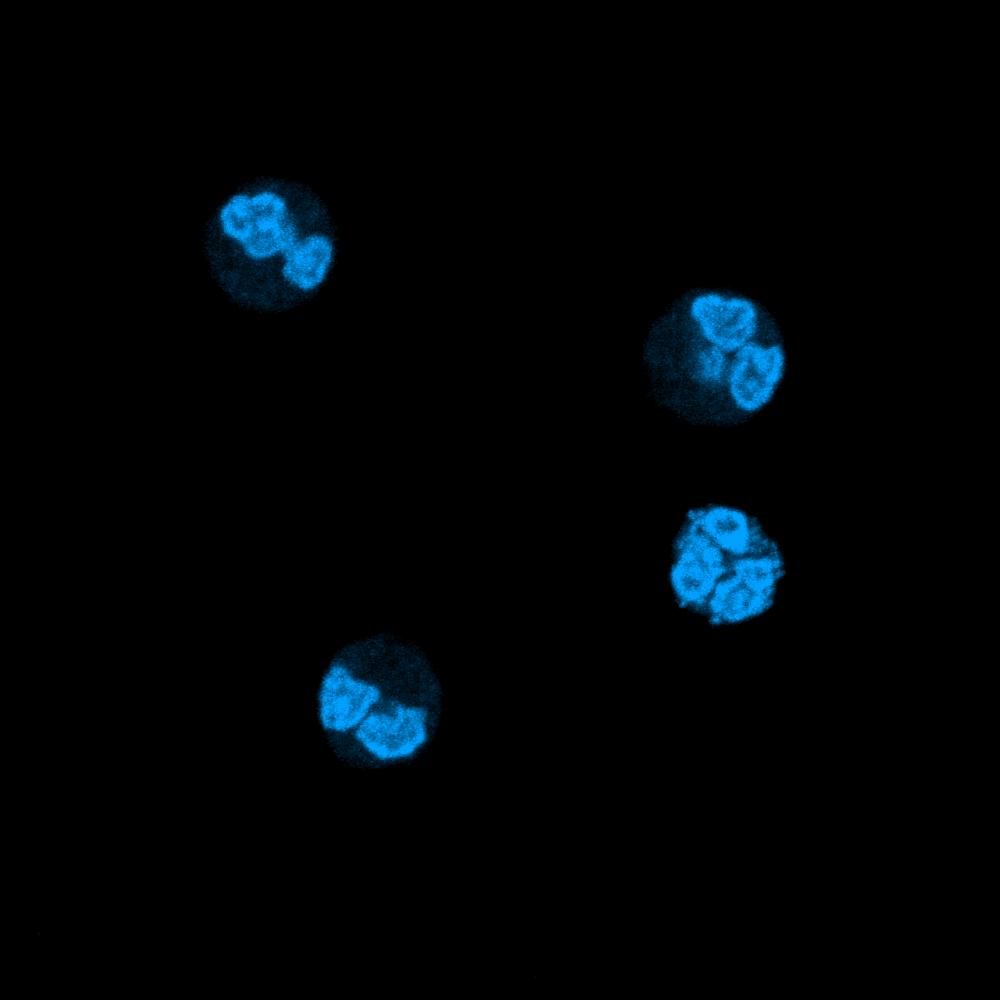

Supplement: Supplementary file 5 — Source data Fig. 2 [file 44319_2024_150_MOESM5_ESM.zip › Main Figure 2/Fig 2B/FB-175 images/untreated/mock nets 1_100/C2-MAX_Experiment-3521-Airyscan Processing-11.png]

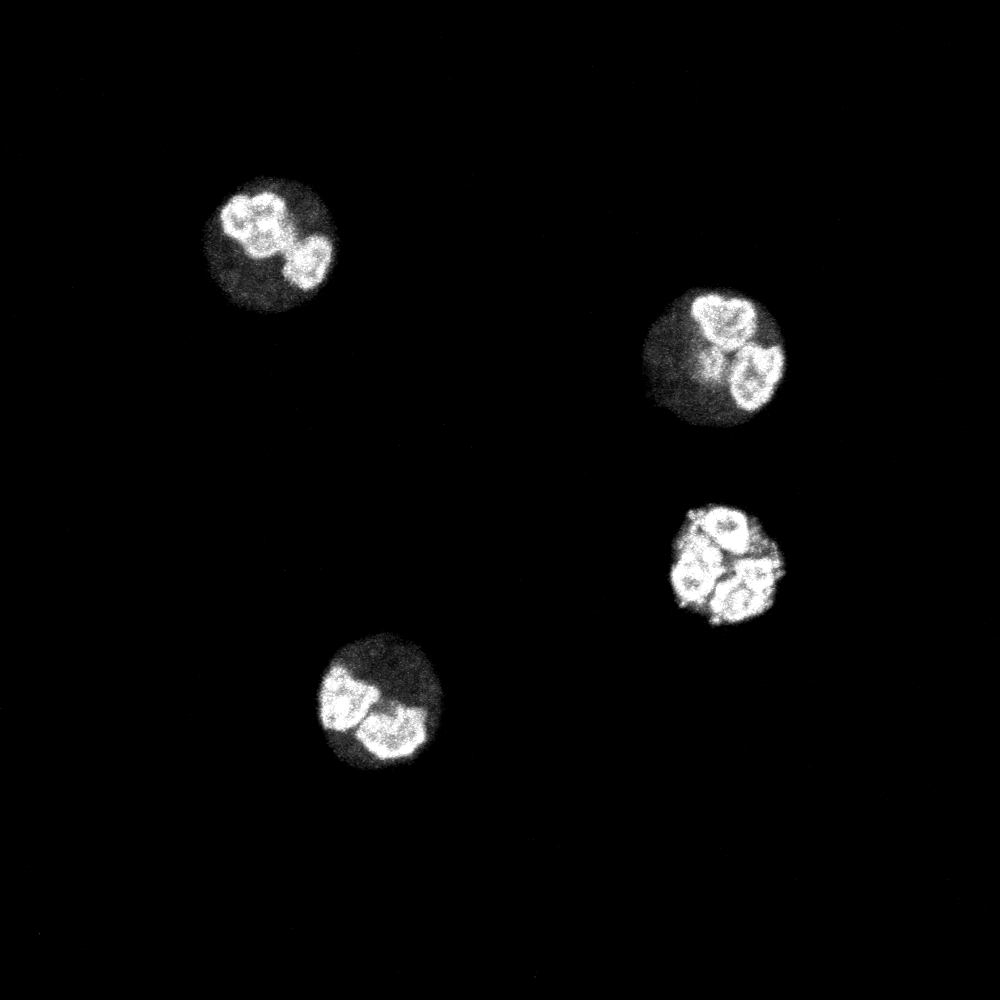

Supplement: Supplementary file 5 — Source data Fig. 2 [file 44319_2024_150_MOESM5_ESM.zip › Main Figure 2/Fig 2B/FB-175 images/untreated/mock nets 1_100/comp gray.png]

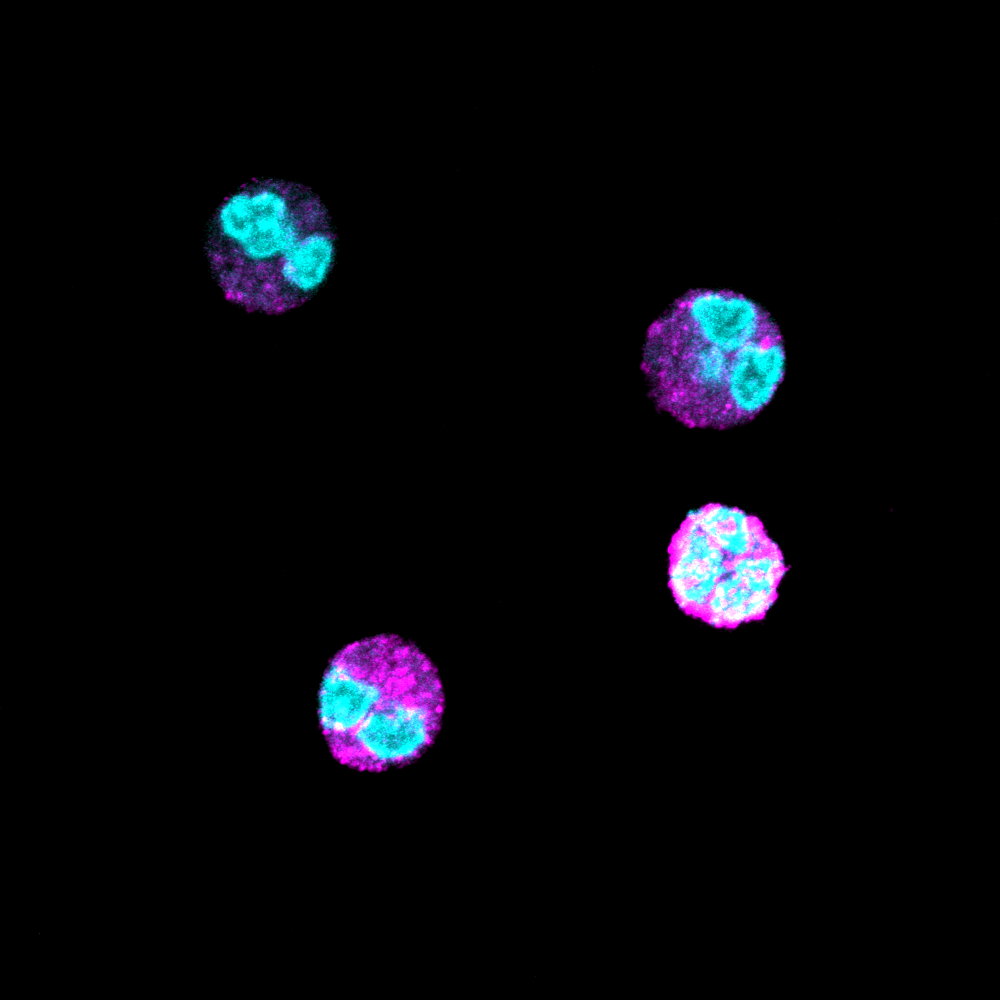

Supplement: Supplementary file 5 — Source data Fig. 2 [file 44319_2024_150_MOESM5_ESM.zip › Main Figure 2/Fig 2B/FB-175 images/untreated/mock nets 1_100/comp new.png]

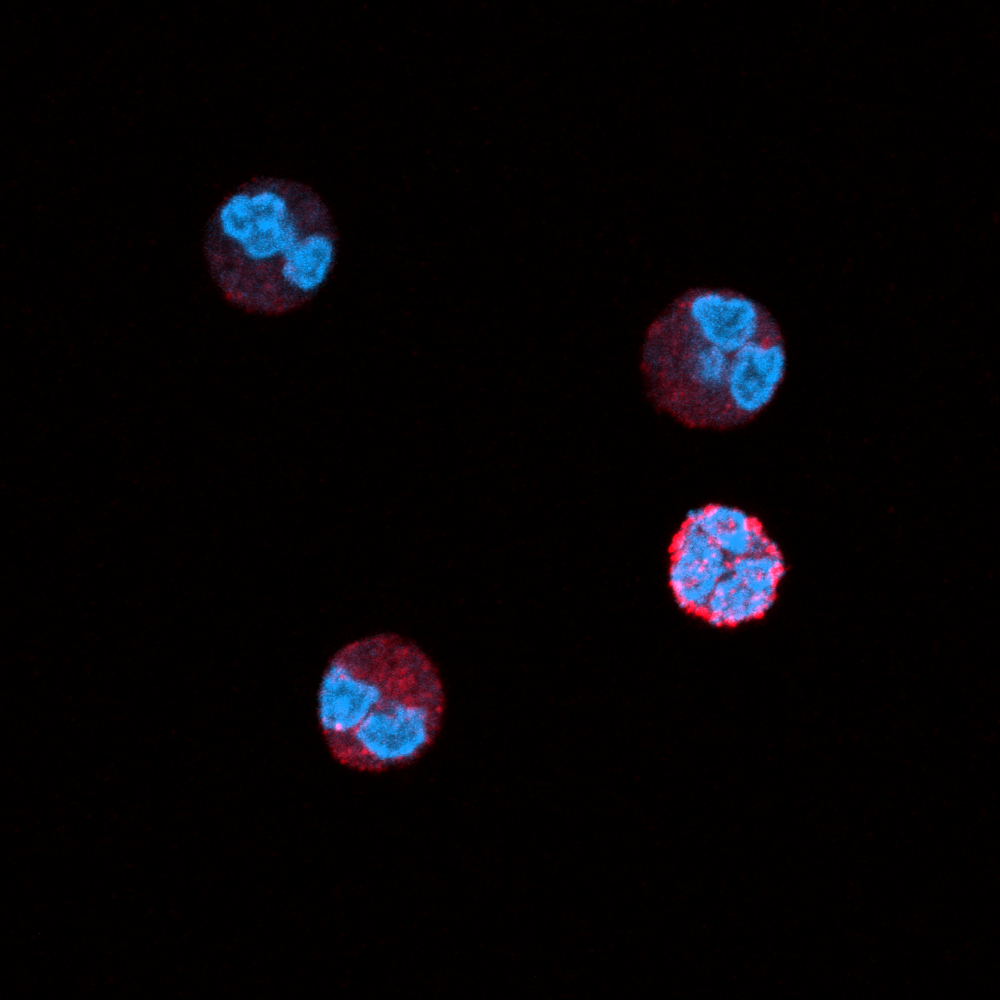

Supplement: Supplementary file 5 — Source data Fig. 2 [file 44319_2024_150_MOESM5_ESM.zip › Main Figure 2/Fig 2B/FB-175 images/untreated/mock nets 1_100/MAX_Experiment-3521-Airyscan Processing-11.png]

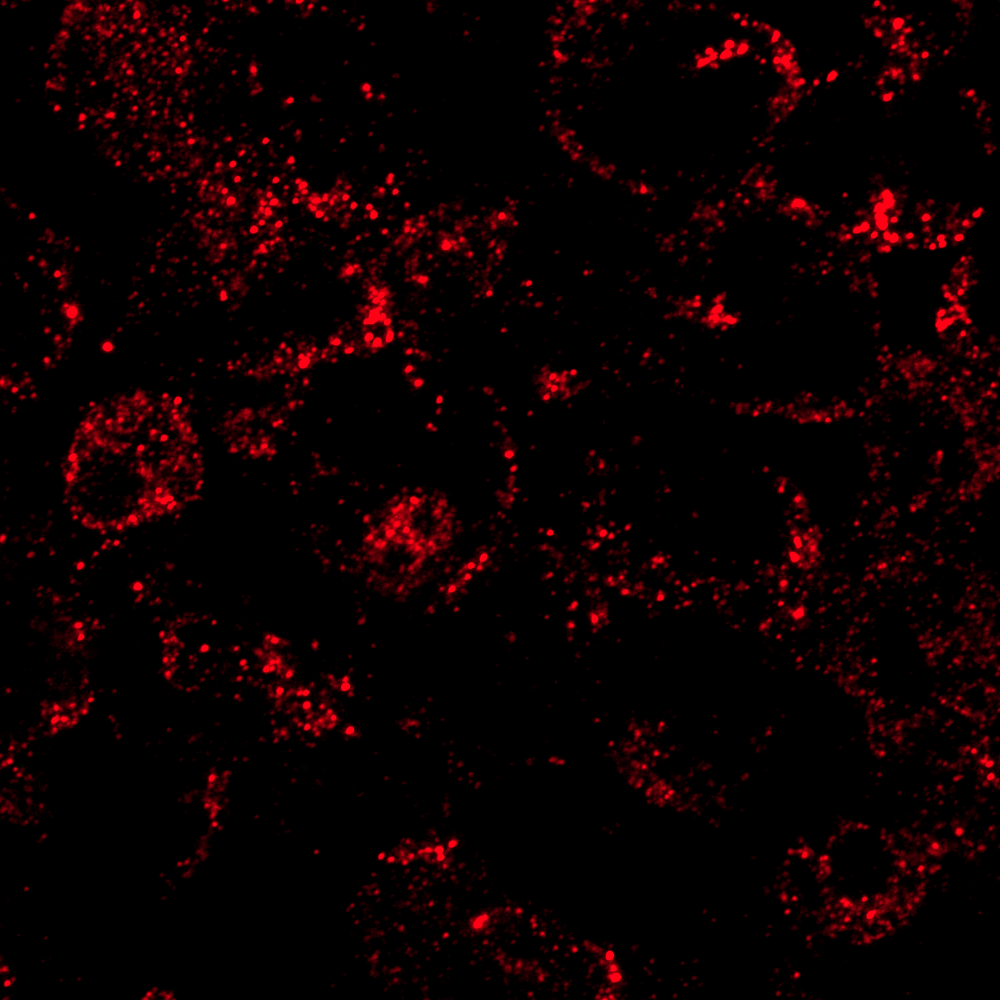

Supplement: Supplementary file 5 — Source data Fig. 2 [file 44319_2024_150_MOESM5_ESM.zip › Main Figure 2/Fig 2B/FB-175 images/untreated/PMA/C1-MAX_Experiment-3520-Airyscan Processing-10.png]

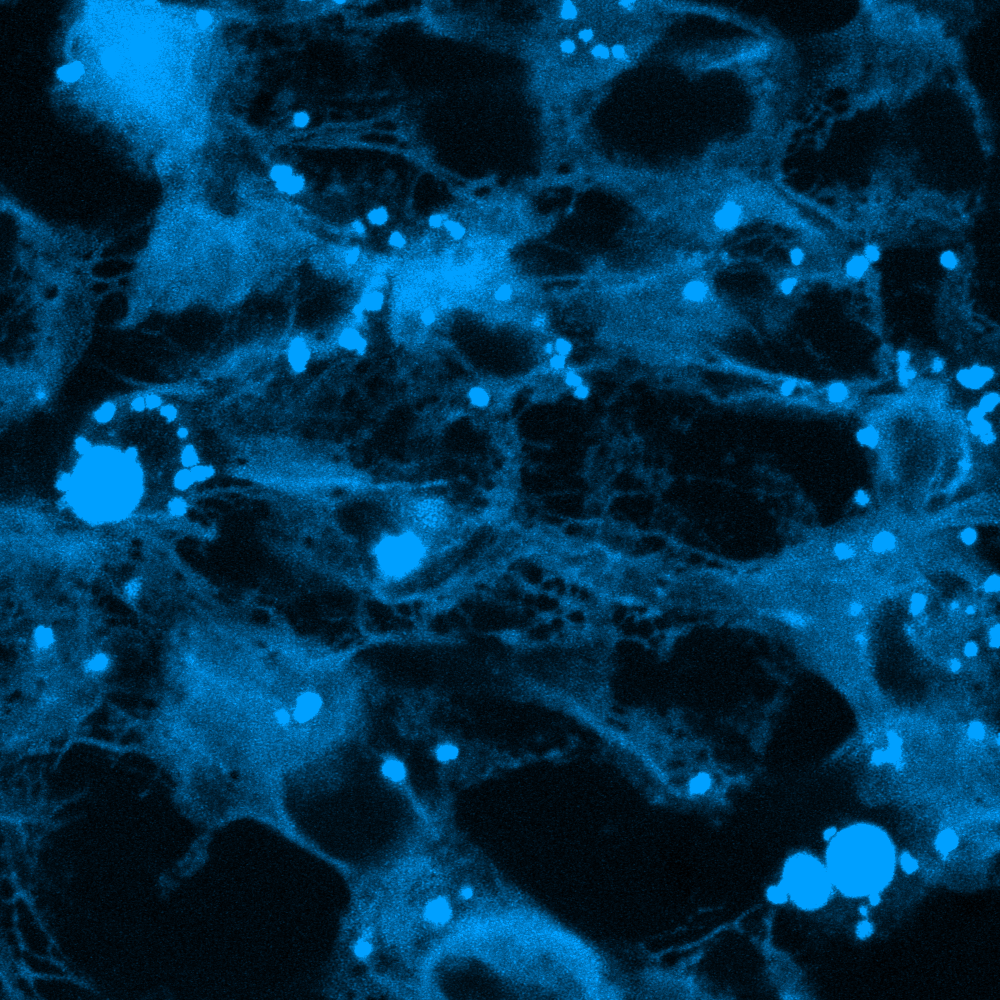

Supplement: Supplementary file 5 — Source data Fig. 2 [file 44319_2024_150_MOESM5_ESM.zip › Main Figure 2/Fig 2B/FB-175 images/untreated/PMA/C2-MAX_Experiment-3520-Airyscan Processing-10.png]

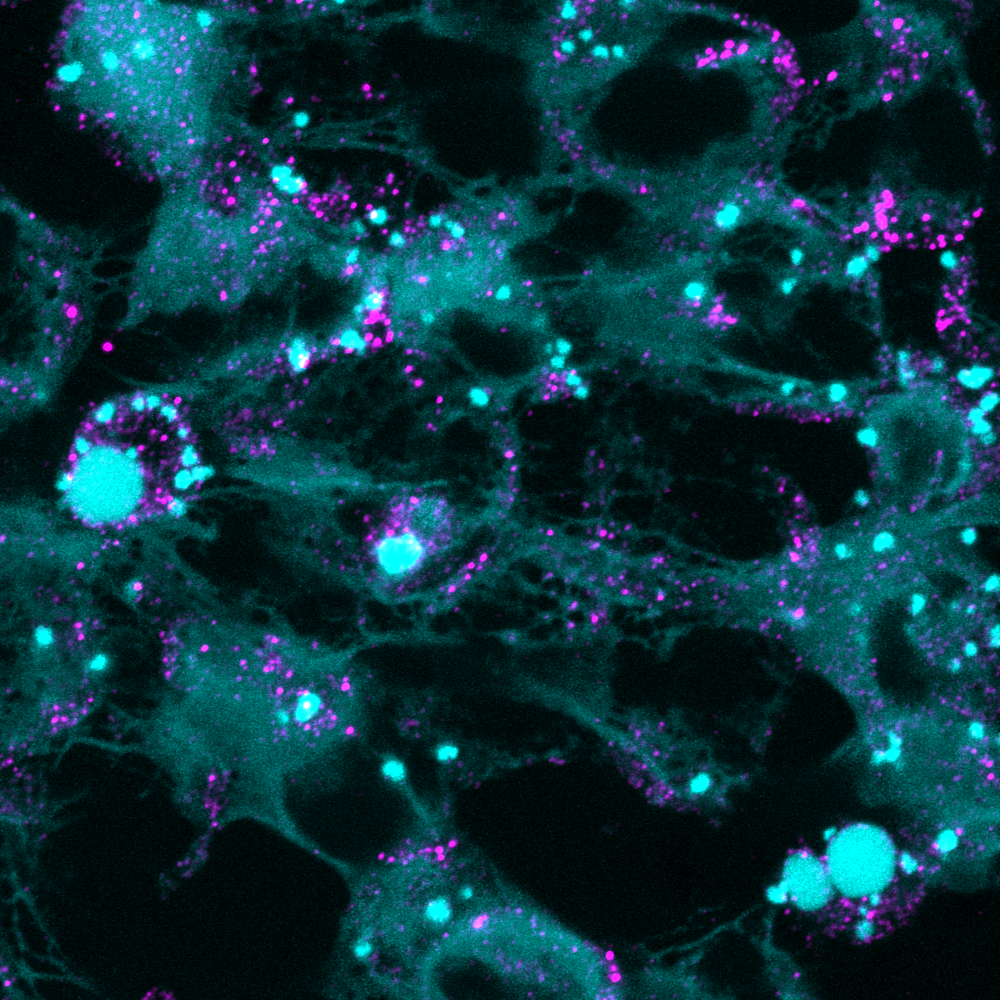

Supplement: Supplementary file 5 — Source data Fig. 2 [file 44319_2024_150_MOESM5_ESM.zip › Main Figure 2/Fig 2B/FB-175 images/untreated/PMA/comp new.png]

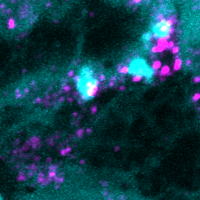

Supplement: Supplementary file 5 — Source data Fig. 2 [file 44319_2024_150_MOESM5_ESM.zip › Main Figure 2/Fig 2B/FB-175 images/untreated/PMA/crop new.png]

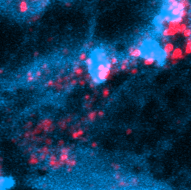

Supplement: Supplementary file 5 — Source data Fig. 2 [file 44319_2024_150_MOESM5_ESM.zip › Main Figure 2/Fig 2B/FB-175 images/untreated/PMA/crop.png]

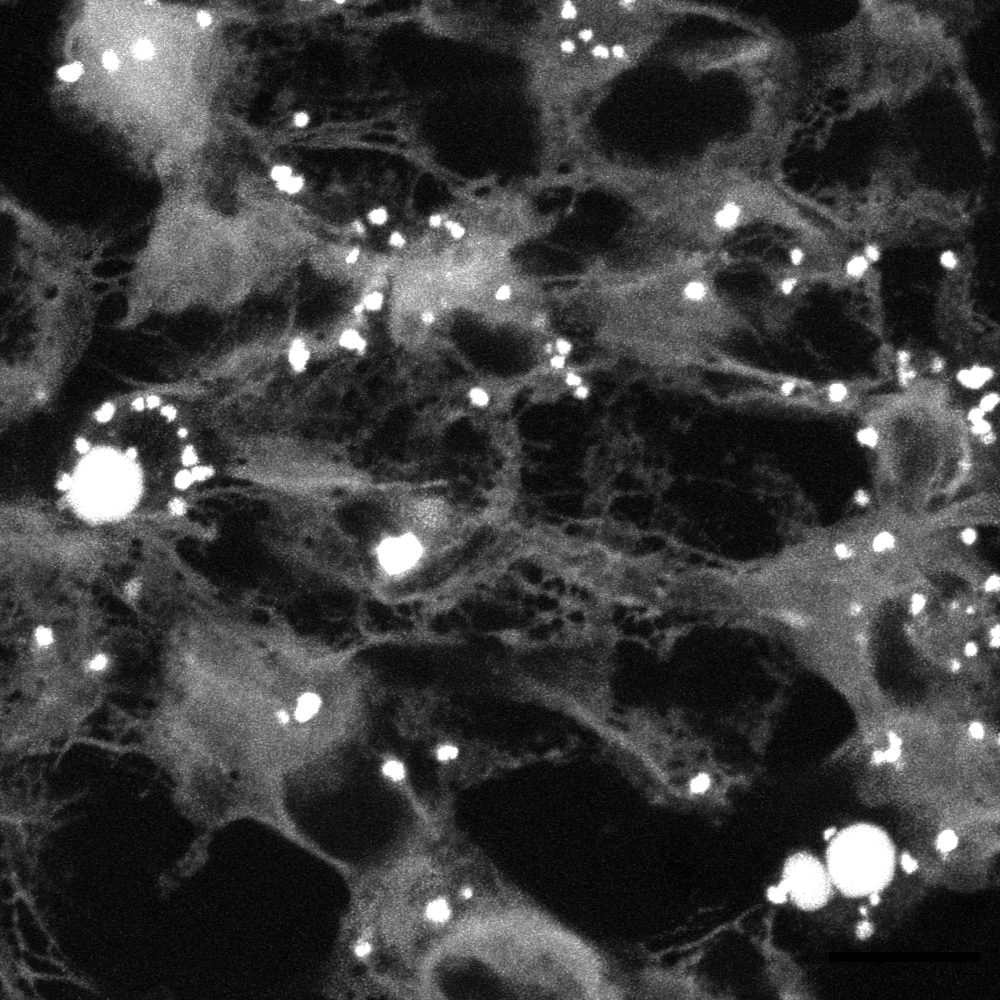

Supplement: Supplementary file 5 — Source data Fig. 2 [file 44319_2024_150_MOESM5_ESM.zip › Main Figure 2/Fig 2B/FB-175 images/untreated/PMA/gray.png]
