# Supplementary figures and images for: naRNA-LL37 composite DAMPs define sterile NETs as self-propagating drivers of inflammation (part 2 of 4)
Source: EMBO Rep. 2024 May 23;25(7):10. doi: 10.1038/s44319-024-00150-5 (PMC11239898; doi:10.1038/s44319-024-00150-5)

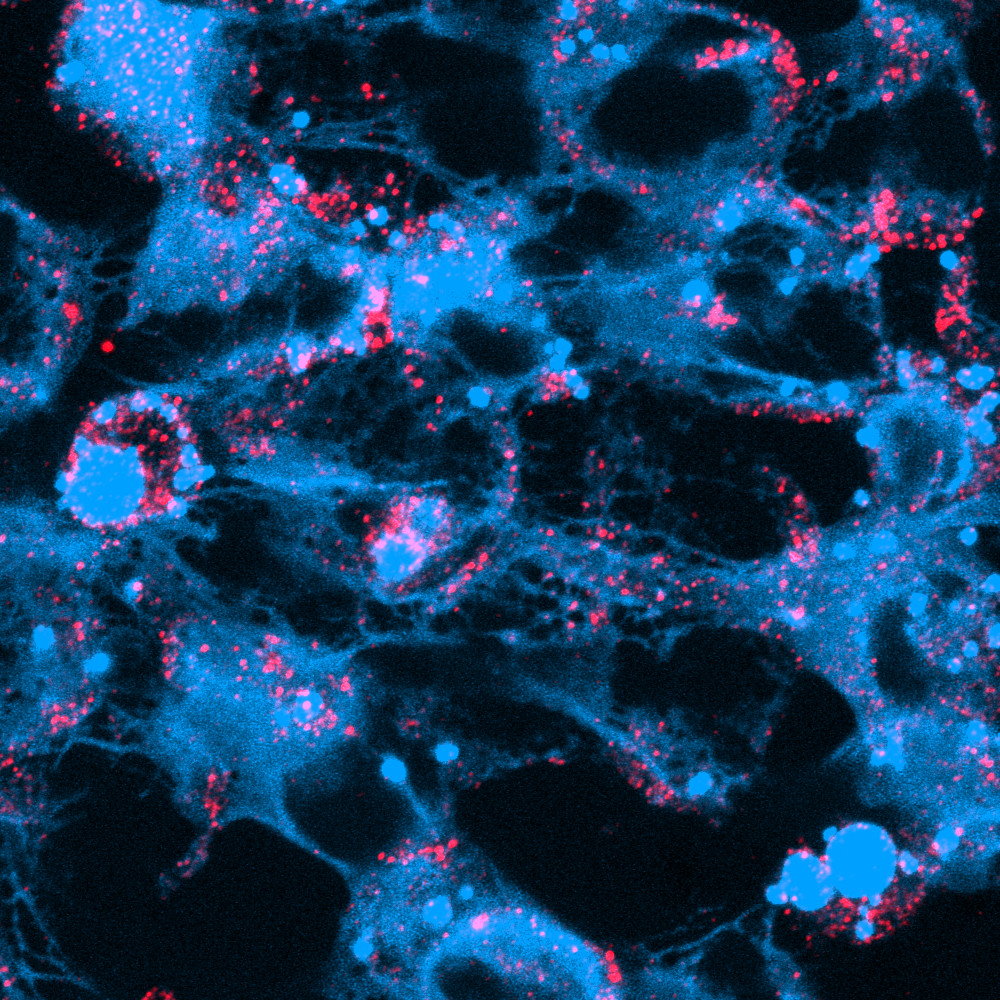

Supplement: Supplementary file 5 — Source data Fig. 2 [file 44319_2024_150_MOESM5_ESM.zip › Main Figure 2/Fig 2B/FB-175 images/untreated/PMA/MAX_Experiment-3520-Airyscan Processing-10.png]

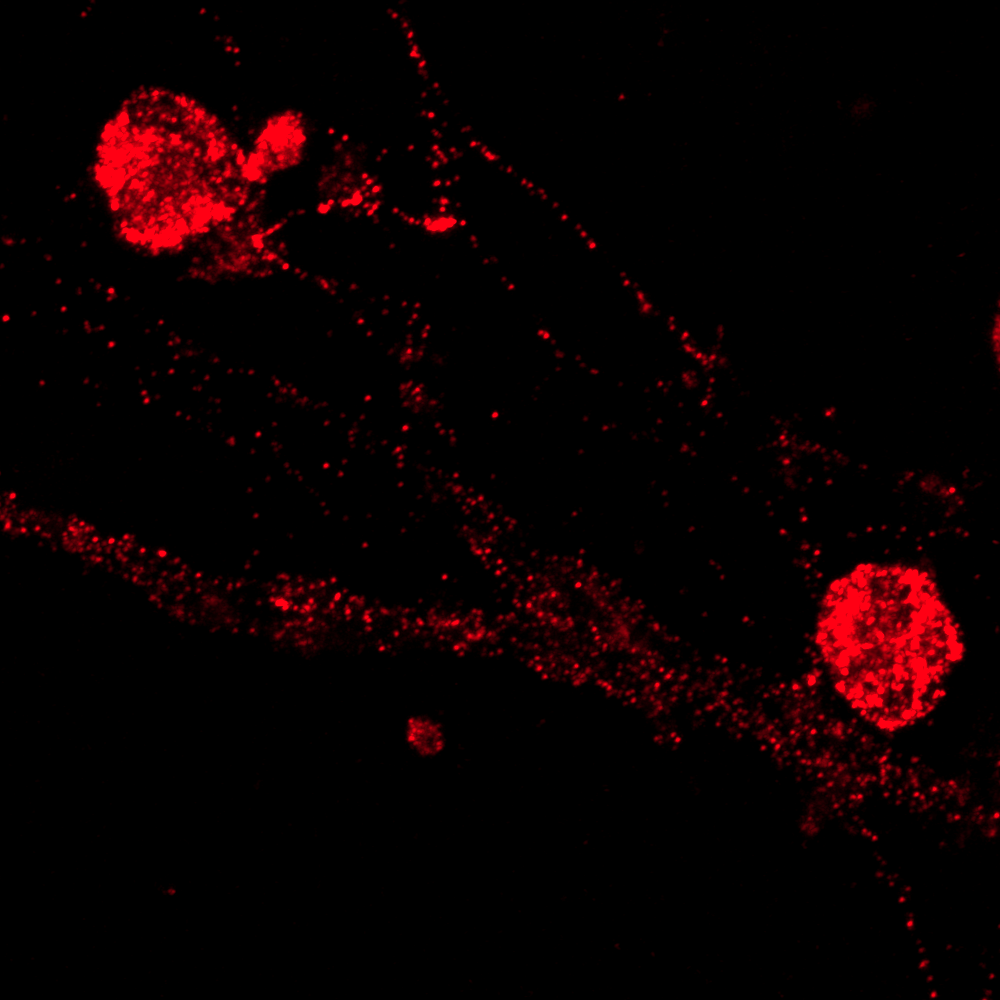

Supplement: Supplementary file 5 — Source data Fig. 2 [file 44319_2024_150_MOESM5_ESM.zip › Main Figure 2/Fig 2B/FB-175 images/untreated/pma nets 1_100/C1-MAX_Experiment-3522-Airyscan Processing-12.png]

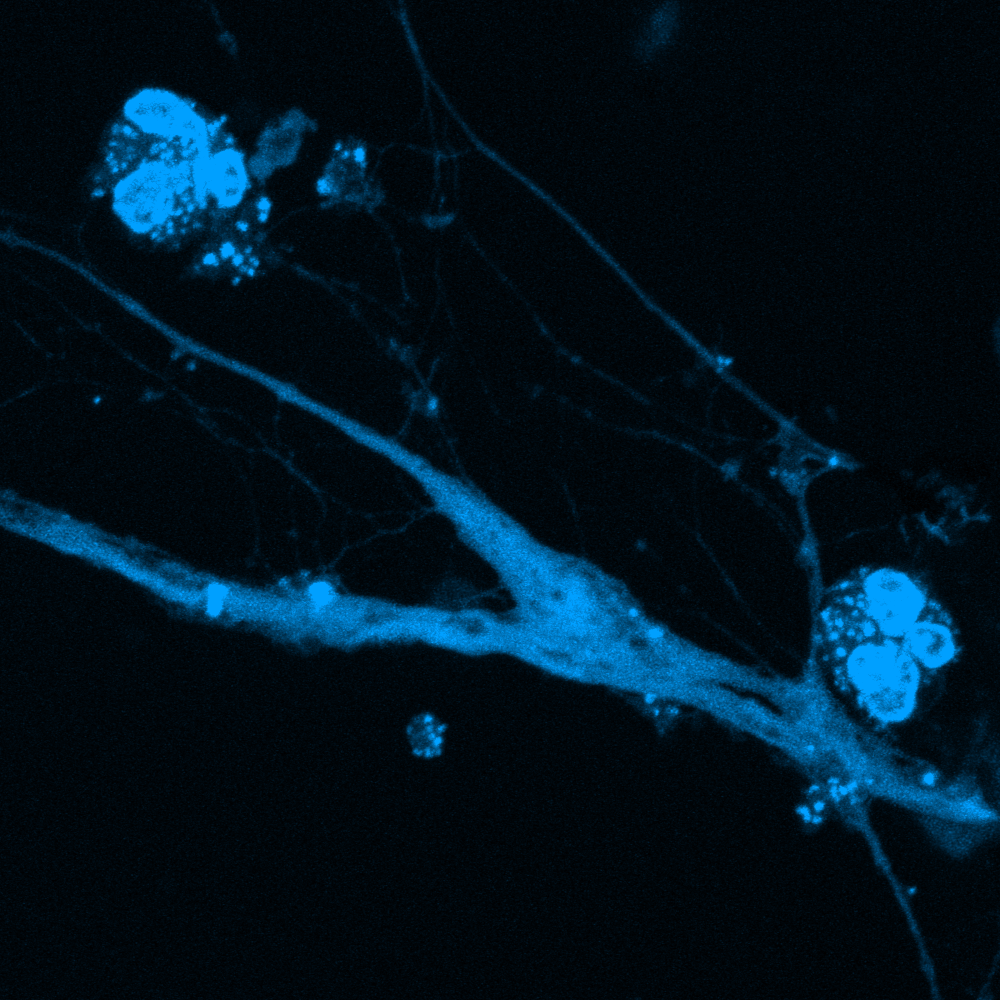

Supplement: Supplementary file 5 — Source data Fig. 2 [file 44319_2024_150_MOESM5_ESM.zip › Main Figure 2/Fig 2B/FB-175 images/untreated/pma nets 1_100/C2-MAX_Experiment-3522-Airyscan Processing-12.png]

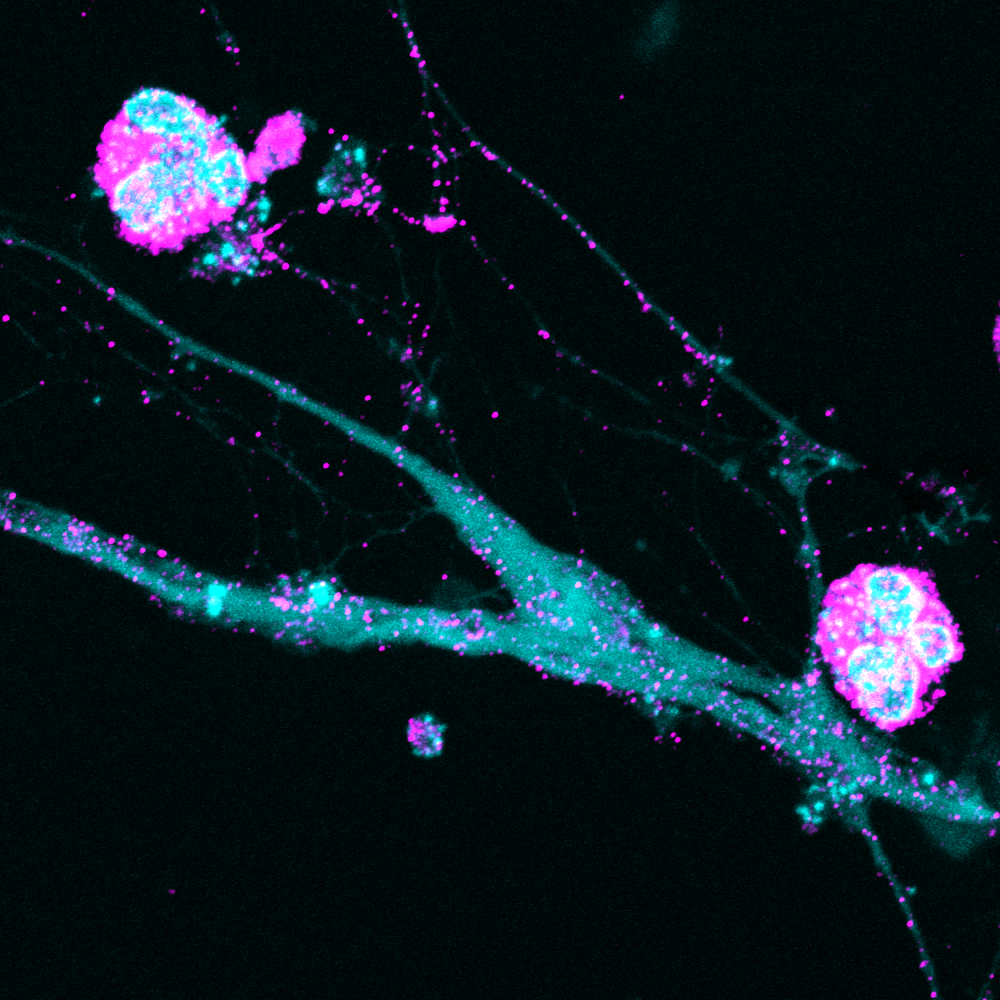

Supplement: Supplementary file 5 — Source data Fig. 2 [file 44319_2024_150_MOESM5_ESM.zip › Main Figure 2/Fig 2B/FB-175 images/untreated/pma nets 1_100/comp new.png]

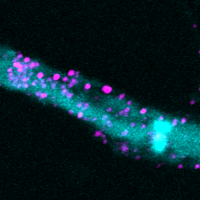

Supplement: Supplementary file 5 — Source data Fig. 2 [file 44319_2024_150_MOESM5_ESM.zip › Main Figure 2/Fig 2B/FB-175 images/untreated/pma nets 1_100/crop new.png]

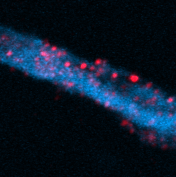

Supplement: Supplementary file 5 — Source data Fig. 2 [file 44319_2024_150_MOESM5_ESM.zip › Main Figure 2/Fig 2B/FB-175 images/untreated/pma nets 1_100/crop.png]

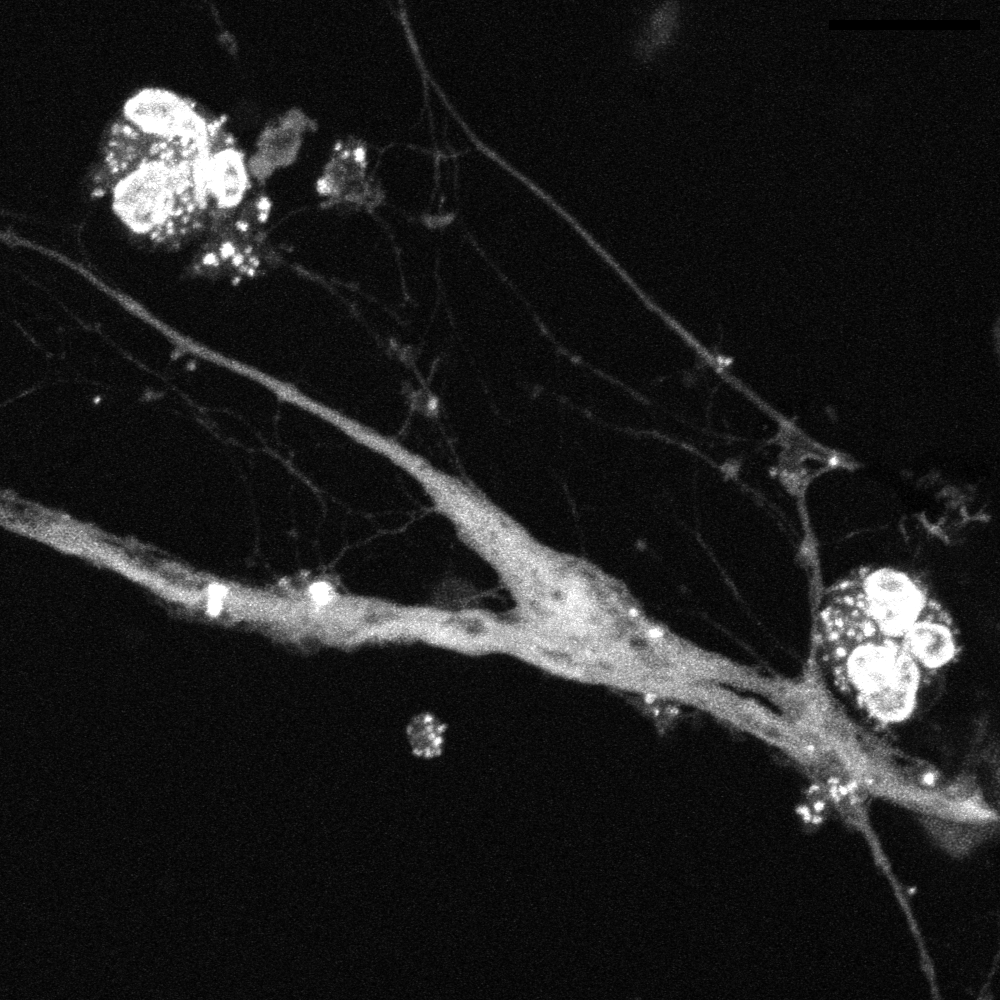

Supplement: Supplementary file 5 — Source data Fig. 2 [file 44319_2024_150_MOESM5_ESM.zip › Main Figure 2/Fig 2B/FB-175 images/untreated/pma nets 1_100/gray.png]

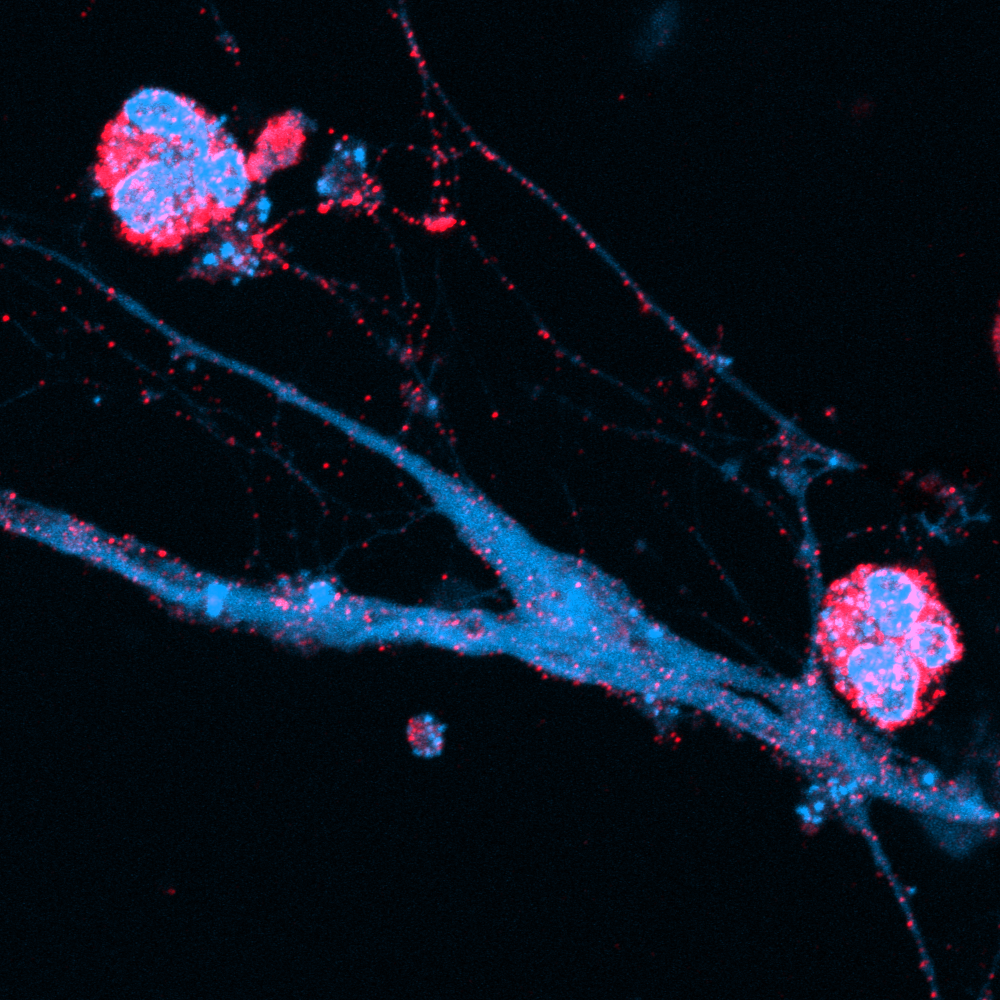

Supplement: Supplementary file 5 — Source data Fig. 2 [file 44319_2024_150_MOESM5_ESM.zip › Main Figure 2/Fig 2B/FB-175 images/untreated/pma nets 1_100/MAX_Experiment-3522-Airyscan Processing-12.png]

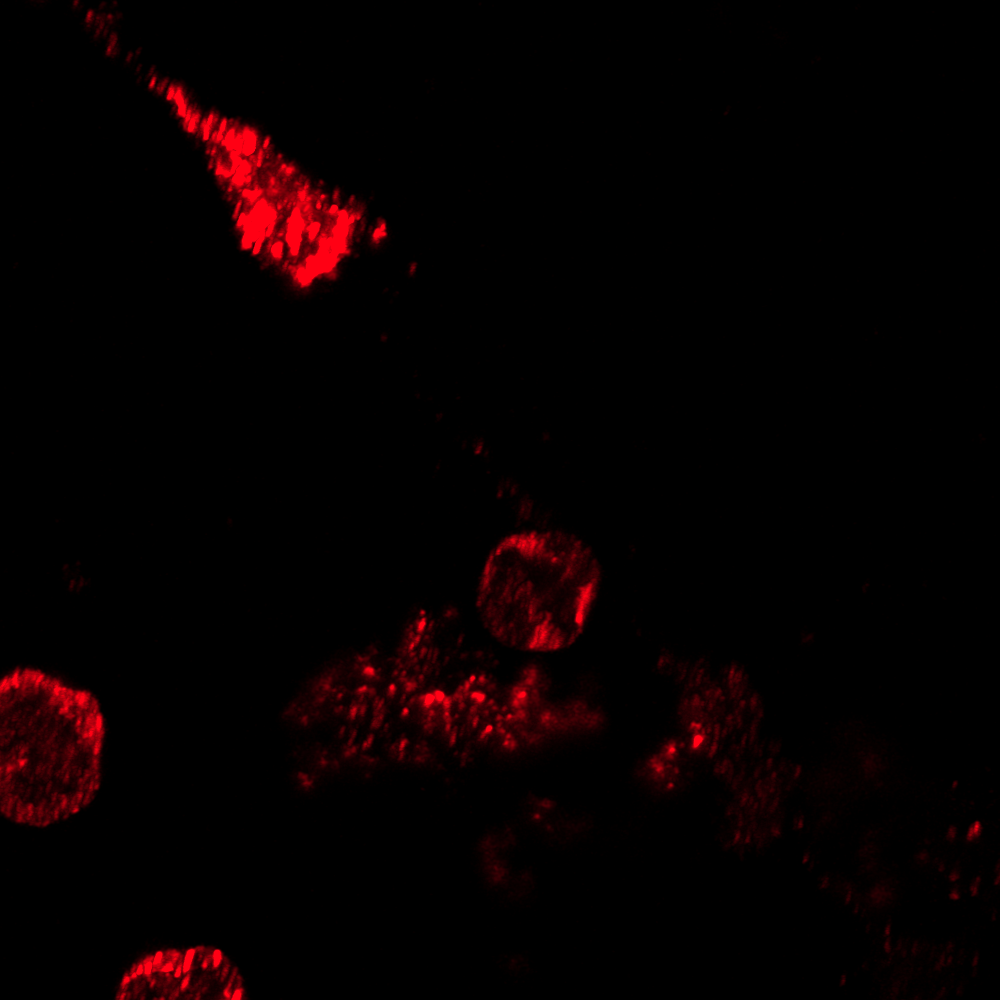

Supplement: Supplementary file 5 — Source data Fig. 2 [file 44319_2024_150_MOESM5_ESM.zip › Main Figure 2/Fig 2B/FB-175 images/untreated/ssRNA ll37/C1-MAX_Experiment-3523-Airyscan Processing-13.png]

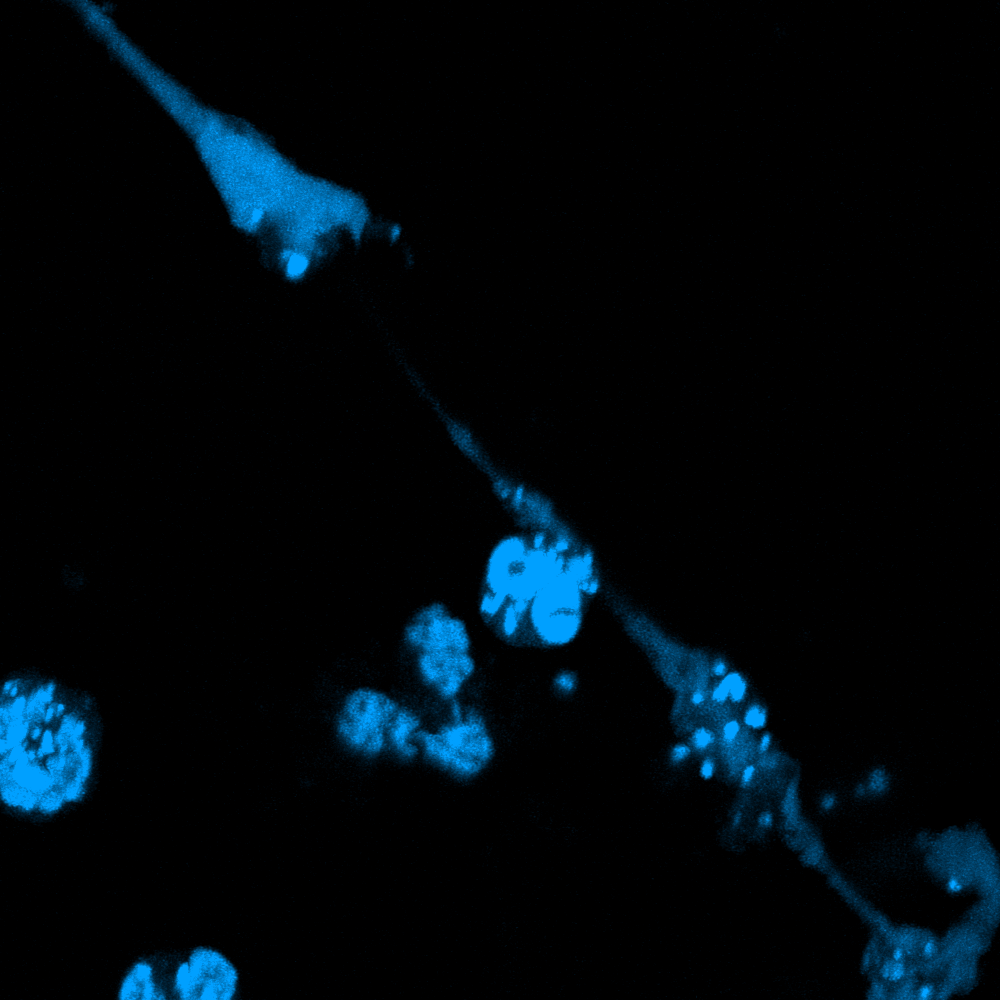

Supplement: Supplementary file 5 — Source data Fig. 2 [file 44319_2024_150_MOESM5_ESM.zip › Main Figure 2/Fig 2B/FB-175 images/untreated/ssRNA ll37/C2-MAX_Experiment-3523-Airyscan Processing-13.png]

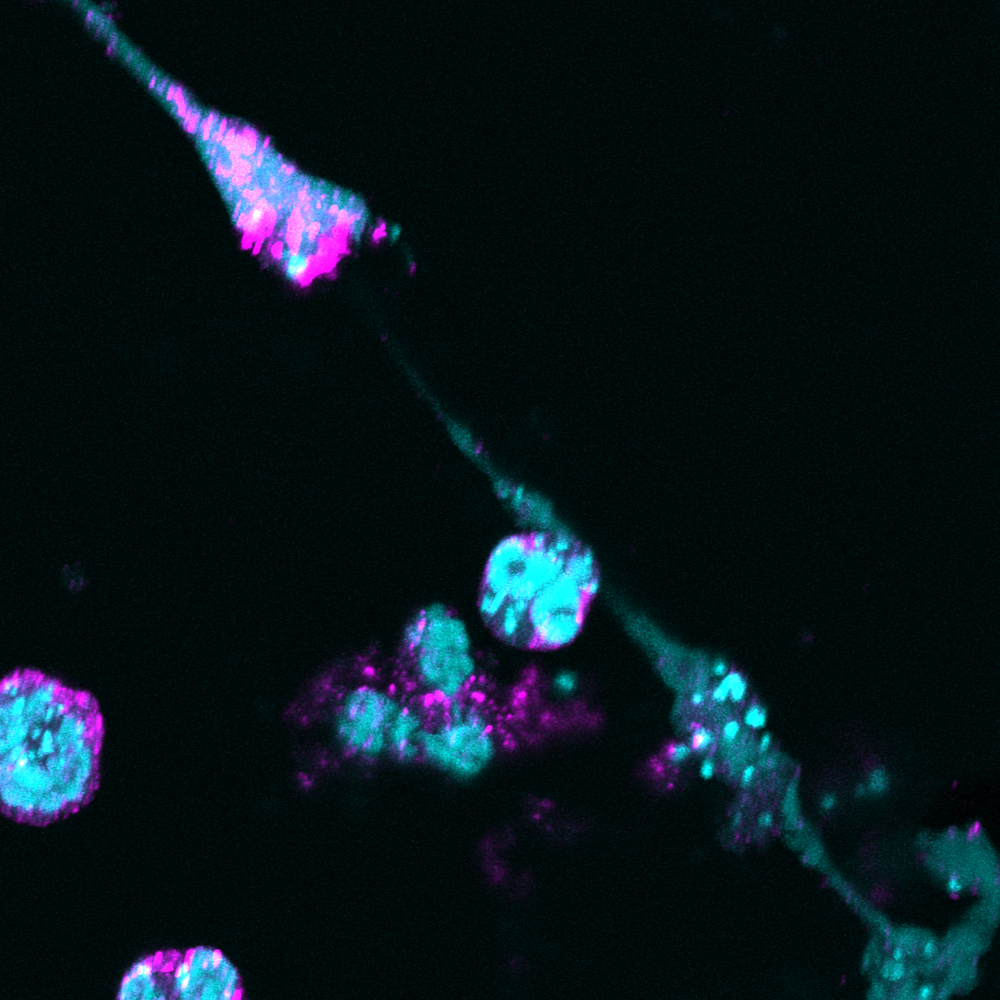

Supplement: Supplementary file 5 — Source data Fig. 2 [file 44319_2024_150_MOESM5_ESM.zip › Main Figure 2/Fig 2B/FB-175 images/untreated/ssRNA ll37/comp new.png]

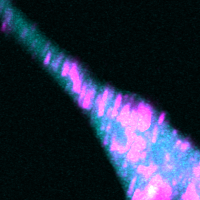

Supplement: Supplementary file 5 — Source data Fig. 2 [file 44319_2024_150_MOESM5_ESM.zip › Main Figure 2/Fig 2B/FB-175 images/untreated/ssRNA ll37/crop new.png]

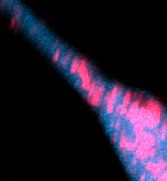

Supplement: Supplementary file 5 — Source data Fig. 2 [file 44319_2024_150_MOESM5_ESM.zip › Main Figure 2/Fig 2B/FB-175 images/untreated/ssRNA ll37/crop.png]

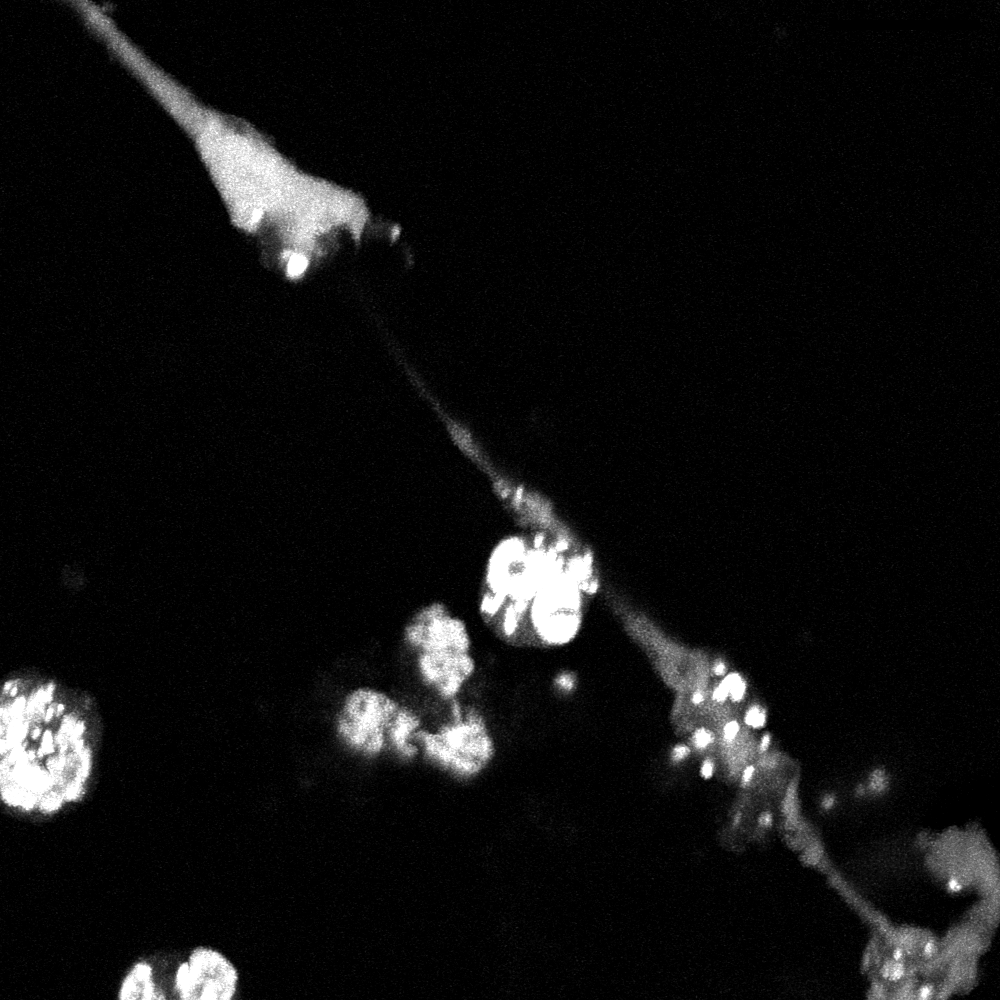

Supplement: Supplementary file 5 — Source data Fig. 2 [file 44319_2024_150_MOESM5_ESM.zip › Main Figure 2/Fig 2B/FB-175 images/untreated/ssRNA ll37/gray.png]

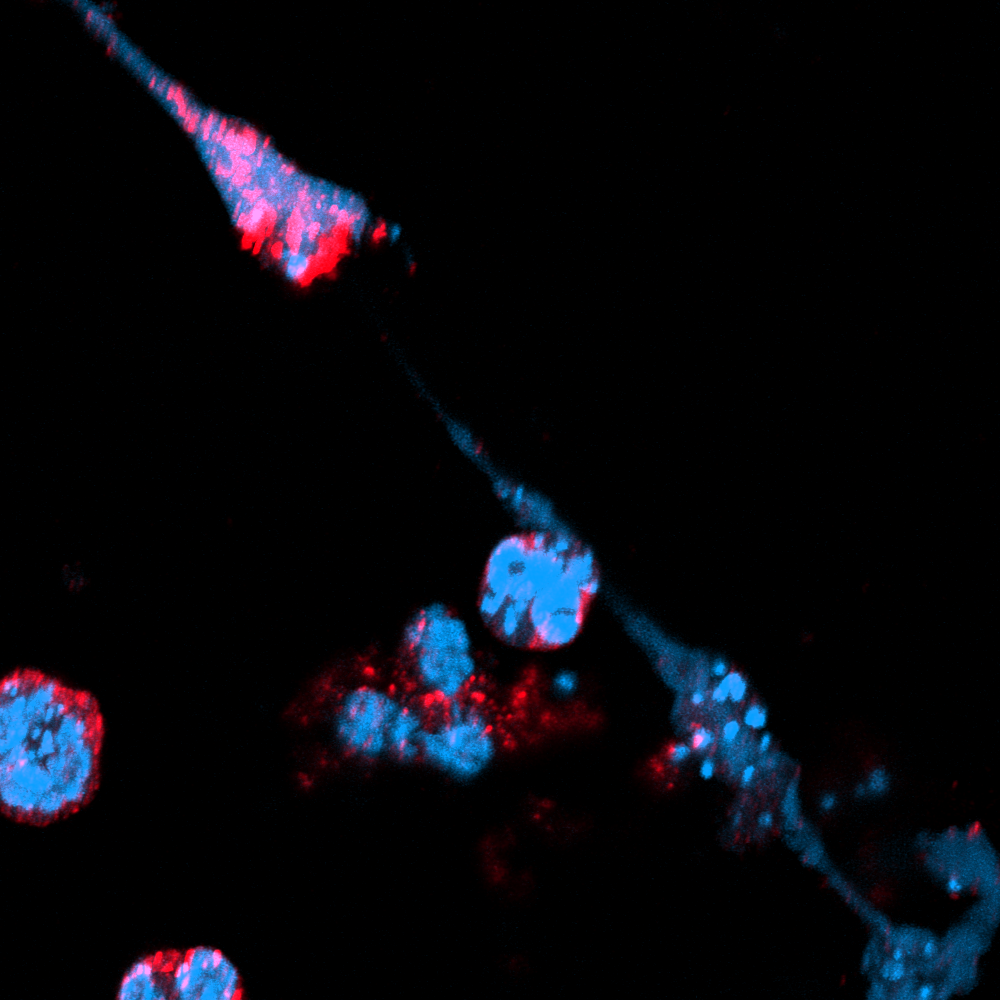

Supplement: Supplementary file 5 — Source data Fig. 2 [file 44319_2024_150_MOESM5_ESM.zip › Main Figure 2/Fig 2B/FB-175 images/untreated/ssRNA ll37/MAX_Experiment-3523-Airyscan Processing-13.png]

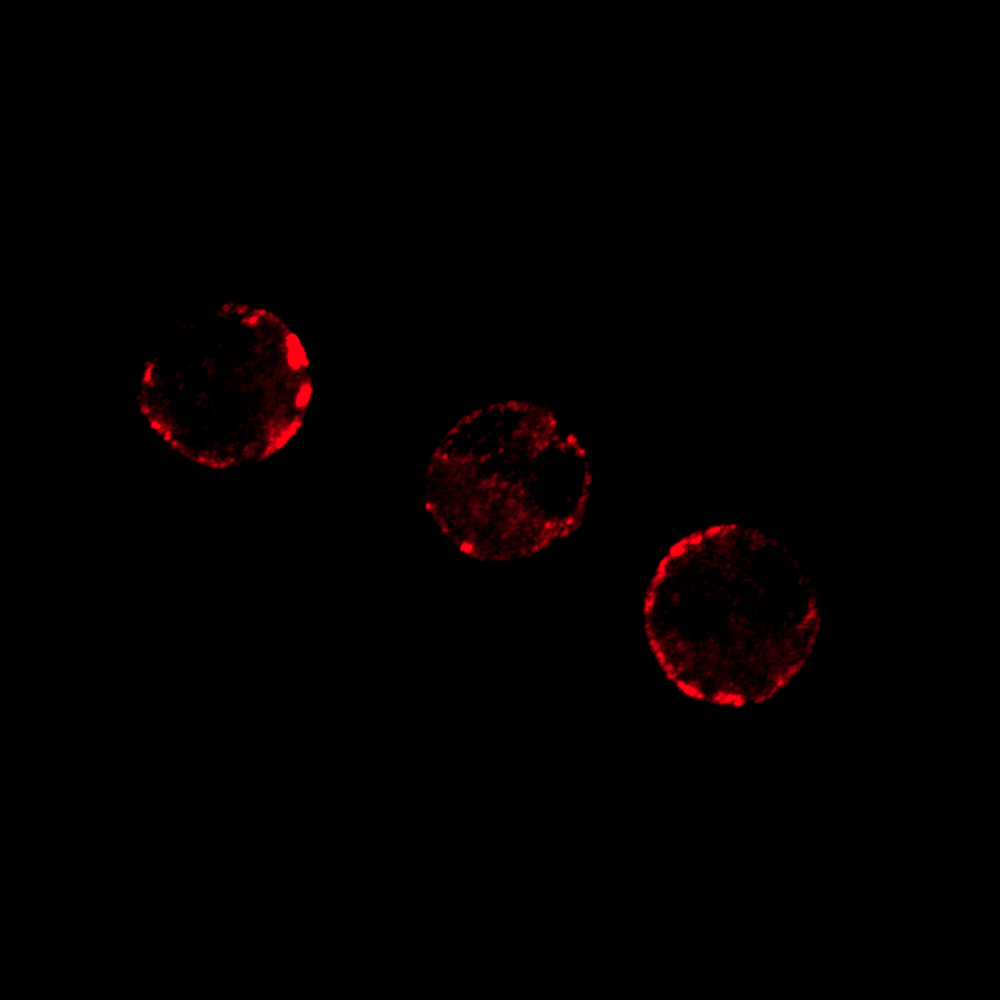

Supplement: Supplementary file 5 — Source data Fig. 2 [file 44319_2024_150_MOESM5_ESM.zip › Main Figure 2/Fig 2B/FB-175 images/untreated/unstimulated/C1-MAX_Experiment-3519-Airyscan Processing-09.png]

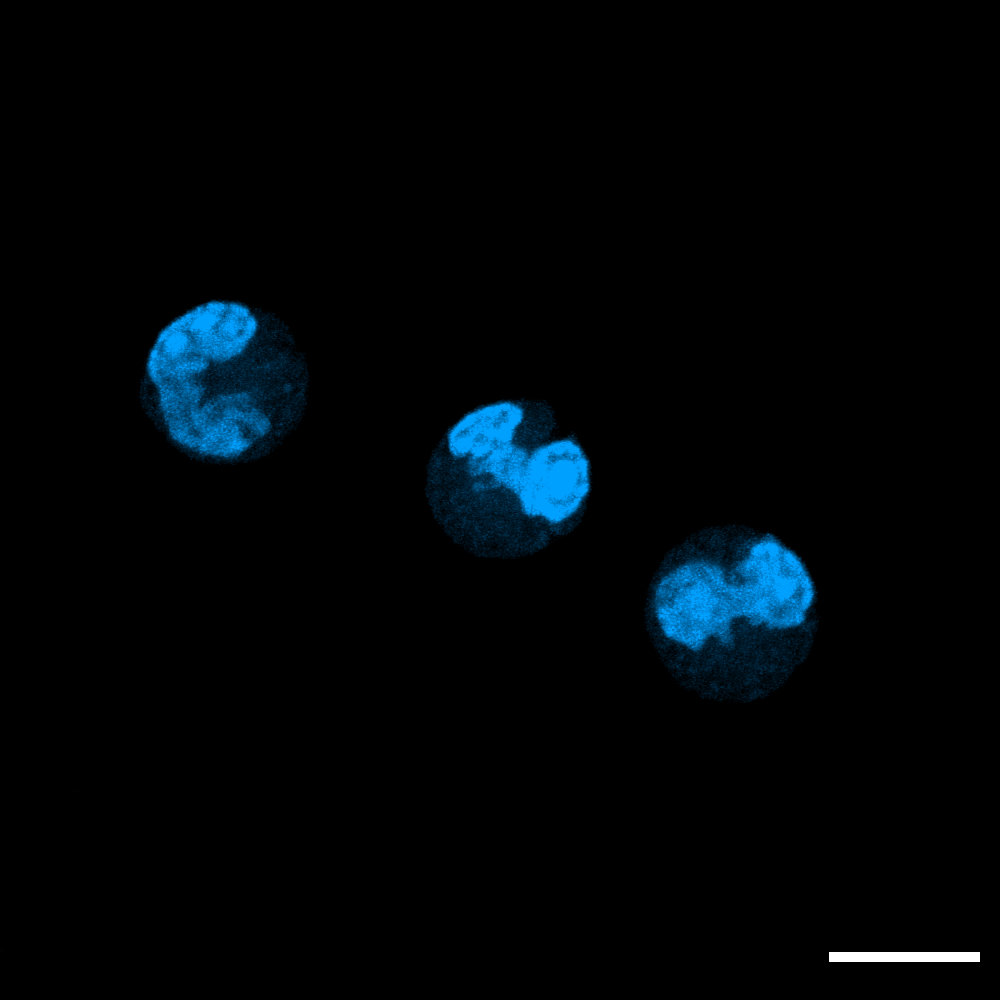

Supplement: Supplementary file 5 — Source data Fig. 2 [file 44319_2024_150_MOESM5_ESM.zip › Main Figure 2/Fig 2B/FB-175 images/untreated/unstimulated/C2-MAX_Experiment-3519-Airyscan Processing-09.png]

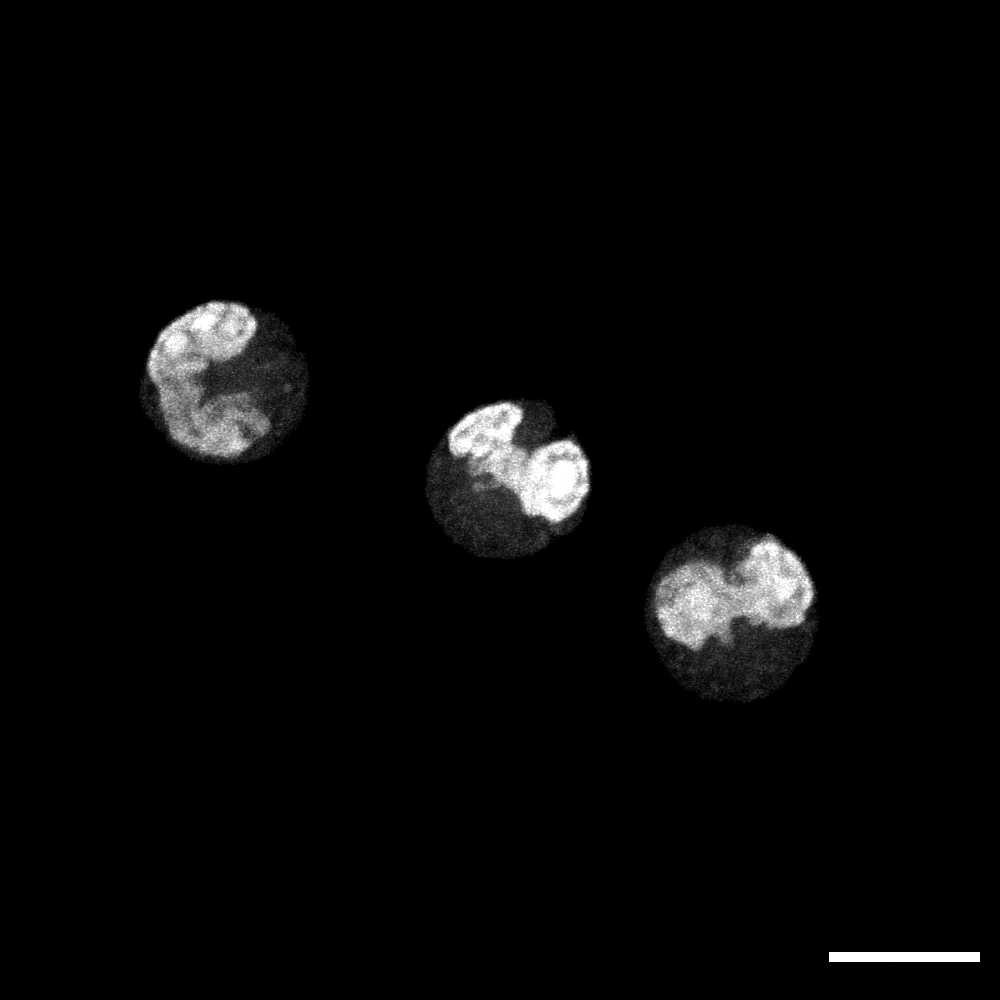

Supplement: Supplementary file 5 — Source data Fig. 2 [file 44319_2024_150_MOESM5_ESM.zip › Main Figure 2/Fig 2B/FB-175 images/untreated/unstimulated/comp new gray.png]

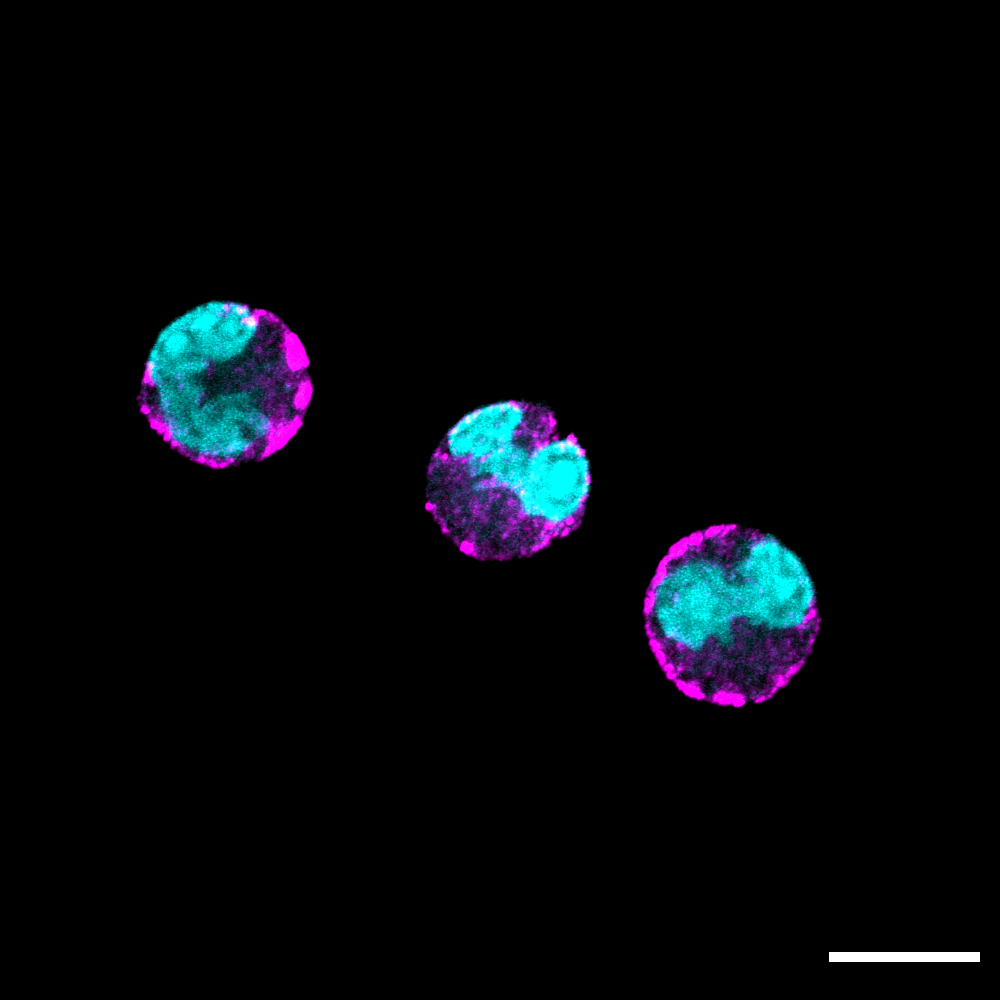

Supplement: Supplementary file 5 — Source data Fig. 2 [file 44319_2024_150_MOESM5_ESM.zip › Main Figure 2/Fig 2B/FB-175 images/untreated/unstimulated/comp new.png]

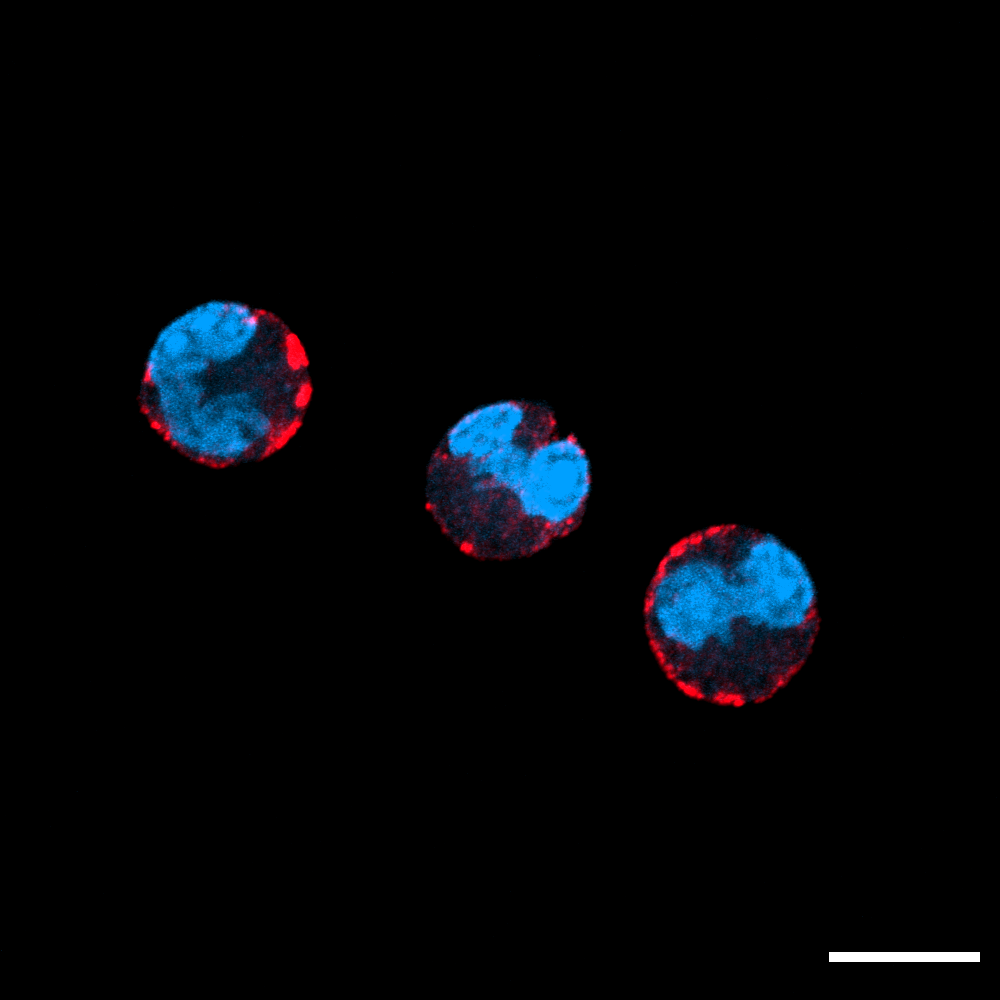

Supplement: Supplementary file 5 — Source data Fig. 2 [file 44319_2024_150_MOESM5_ESM.zip › Main Figure 2/Fig 2B/FB-175 images/untreated/unstimulated/MAX_Experiment-3519-Airyscan Processing-09 scale bar.png]

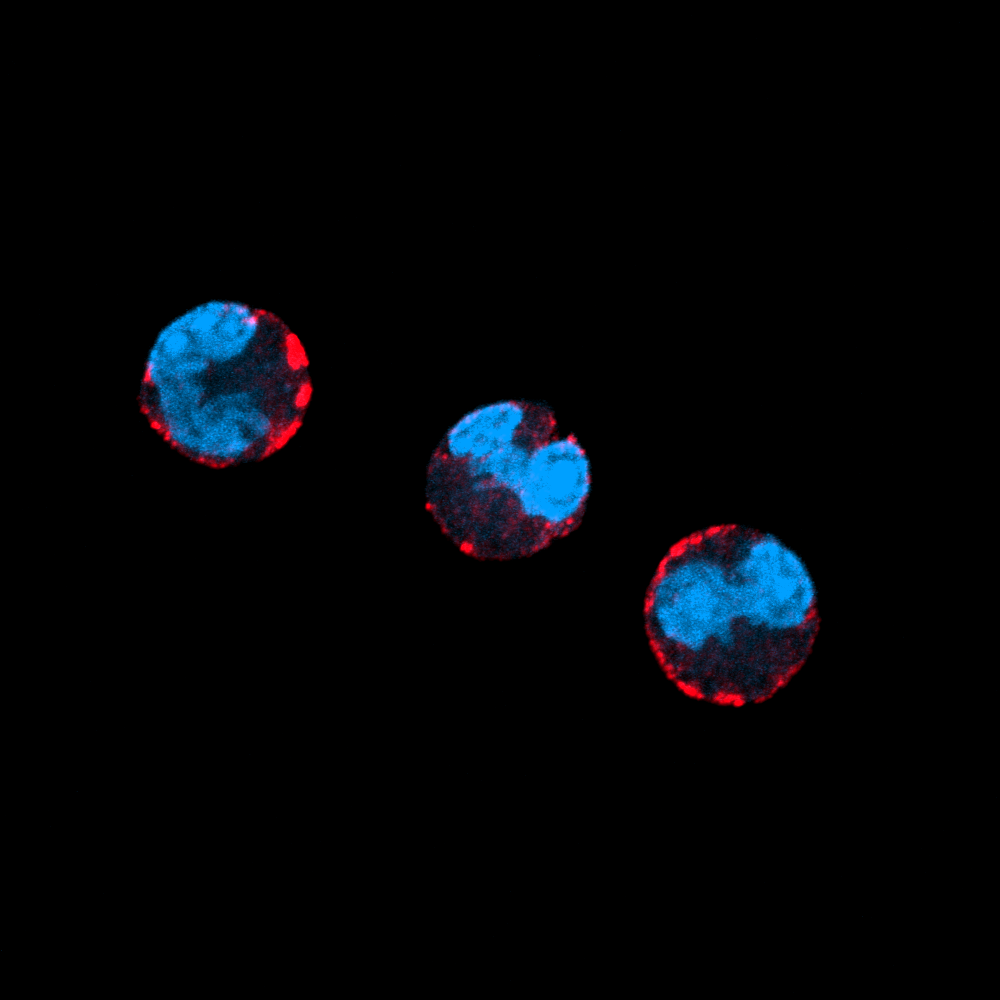

Supplement: Supplementary file 5 — Source data Fig. 2 [file 44319_2024_150_MOESM5_ESM.zip › Main Figure 2/Fig 2B/FB-175 images/untreated/unstimulated/MAX_Experiment-3519-Airyscan Processing-09.png]

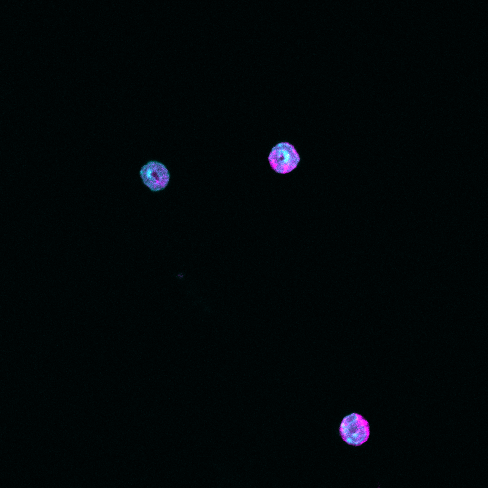

Supplement: Supplementary file 5 — Source data Fig. 2 [file 44319_2024_150_MOESM5_ESM.zip › Main Figure 2/Fig 2D/FB-124 WT and TLR13 KO/KO Mock NETs/comp new.png]

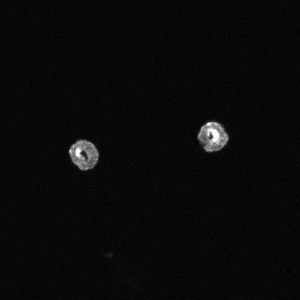

Supplement: Supplementary file 5 — Source data Fig. 2 [file 44319_2024_150_MOESM5_ESM.zip › Main Figure 2/Fig 2D/FB-124 WT and TLR13 KO/KO Mock NETs/gray.png]

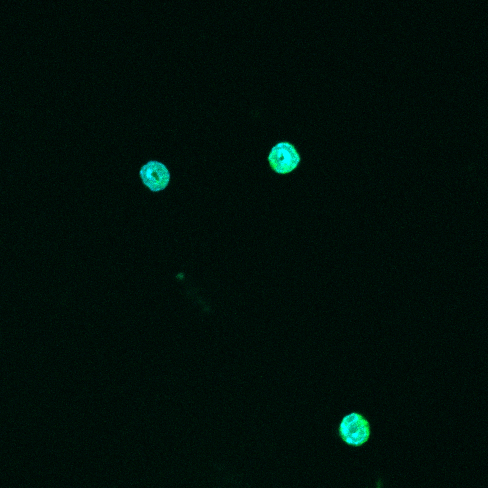

Supplement: Supplementary file 5 — Source data Fig. 2 [file 44319_2024_150_MOESM5_ESM.zip › Main Figure 2/Fig 2D/FB-124 WT and TLR13 KO/KO Mock NETs/new.png]

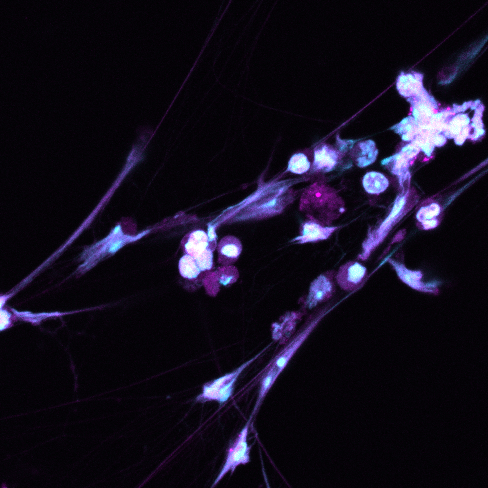

Supplement: Supplementary file 5 — Source data Fig. 2 [file 44319_2024_150_MOESM5_ESM.zip › Main Figure 2/Fig 2D/FB-124 WT and TLR13 KO/KO PMA/comp new.png]

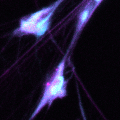

Supplement: Supplementary file 5 — Source data Fig. 2 [file 44319_2024_150_MOESM5_ESM.zip › Main Figure 2/Fig 2D/FB-124 WT and TLR13 KO/KO PMA/crop new.png]

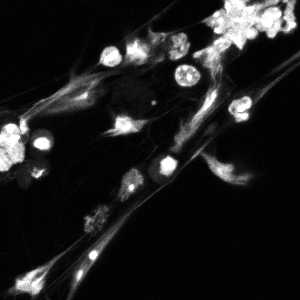

Supplement: Supplementary file 5 — Source data Fig. 2 [file 44319_2024_150_MOESM5_ESM.zip › Main Figure 2/Fig 2D/FB-124 WT and TLR13 KO/KO PMA/gray.png]

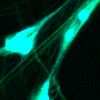

Supplement: Supplementary file 5 — Source data Fig. 2 [file 44319_2024_150_MOESM5_ESM.zip › Main Figure 2/Fig 2D/FB-124 WT and TLR13 KO/KO PMA/new crop.png]

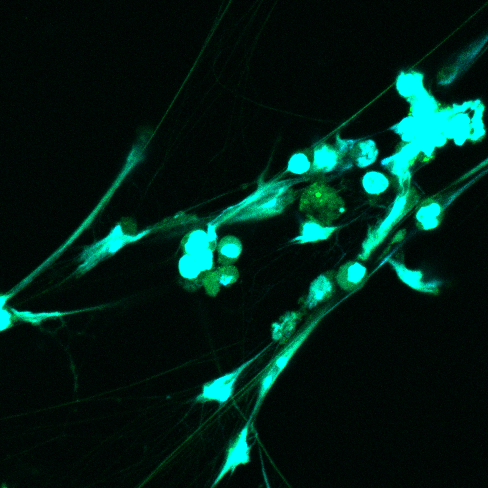

Supplement: Supplementary file 5 — Source data Fig. 2 [file 44319_2024_150_MOESM5_ESM.zip › Main Figure 2/Fig 2D/FB-124 WT and TLR13 KO/KO PMA/new.png]

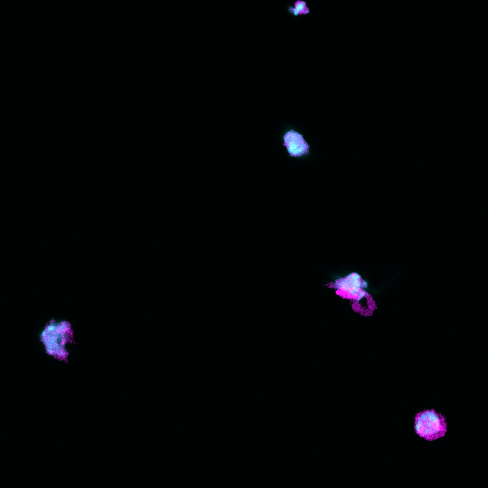

Supplement: Supplementary file 5 — Source data Fig. 2 [file 44319_2024_150_MOESM5_ESM.zip › Main Figure 2/Fig 2D/FB-124 WT and TLR13 KO/KO PMA NETs/comp new.png]

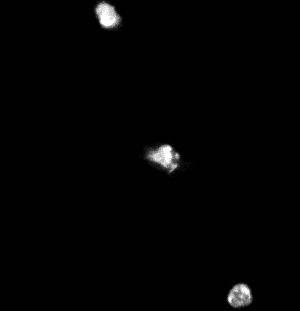

Supplement: Supplementary file 5 — Source data Fig. 2 [file 44319_2024_150_MOESM5_ESM.zip › Main Figure 2/Fig 2D/FB-124 WT and TLR13 KO/KO PMA NETs/gray.png]

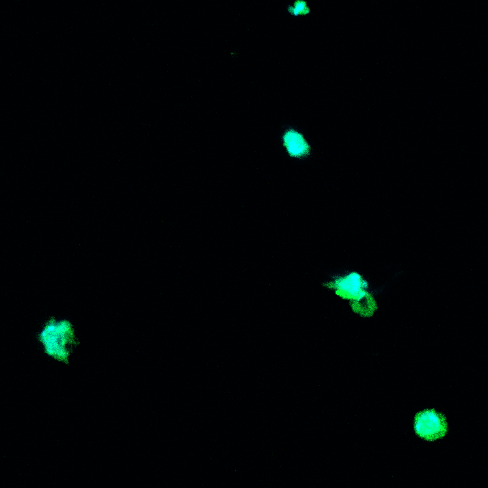

Supplement: Supplementary file 5 — Source data Fig. 2 [file 44319_2024_150_MOESM5_ESM.zip › Main Figure 2/Fig 2D/FB-124 WT and TLR13 KO/KO PMA NETs/new.png]

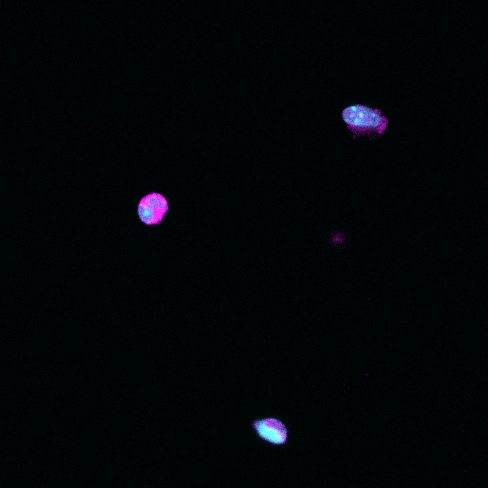

Supplement: Supplementary file 5 — Source data Fig. 2 [file 44319_2024_150_MOESM5_ESM.zip › Main Figure 2/Fig 2D/FB-124 WT and TLR13 KO/KO unstim/comp new.png]

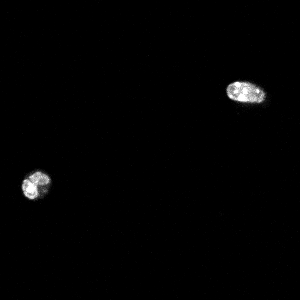

Supplement: Supplementary file 5 — Source data Fig. 2 [file 44319_2024_150_MOESM5_ESM.zip › Main Figure 2/Fig 2D/FB-124 WT and TLR13 KO/KO unstim/gray.png]

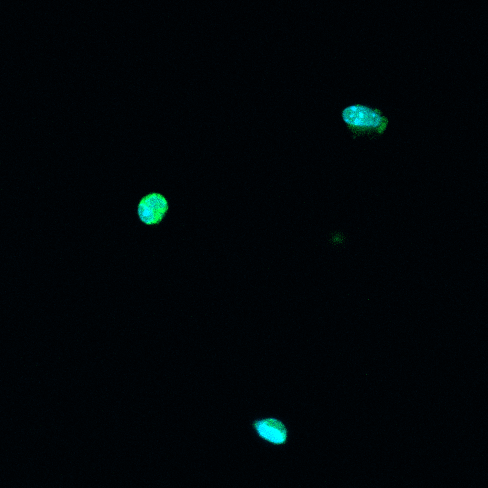

Supplement: Supplementary file 5 — Source data Fig. 2 [file 44319_2024_150_MOESM5_ESM.zip › Main Figure 2/Fig 2D/FB-124 WT and TLR13 KO/KO unstim/new.png]

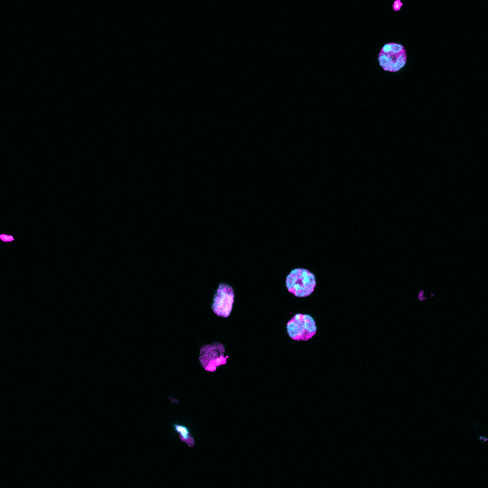

Supplement: Supplementary file 5 — Source data Fig. 2 [file 44319_2024_150_MOESM5_ESM.zip › Main Figure 2/Fig 2D/FB-124 WT and TLR13 KO/WT Mock NETs/comp new.png]

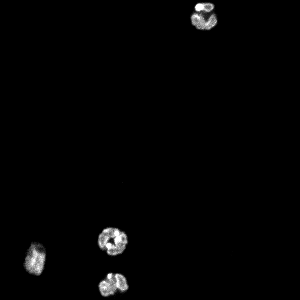

Supplement: Supplementary file 5 — Source data Fig. 2 [file 44319_2024_150_MOESM5_ESM.zip › Main Figure 2/Fig 2D/FB-124 WT and TLR13 KO/WT Mock NETs/gray.png]

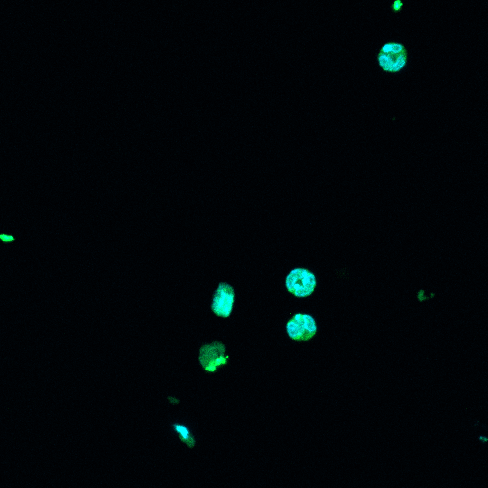

Supplement: Supplementary file 5 — Source data Fig. 2 [file 44319_2024_150_MOESM5_ESM.zip › Main Figure 2/Fig 2D/FB-124 WT and TLR13 KO/WT Mock NETs/new.png]

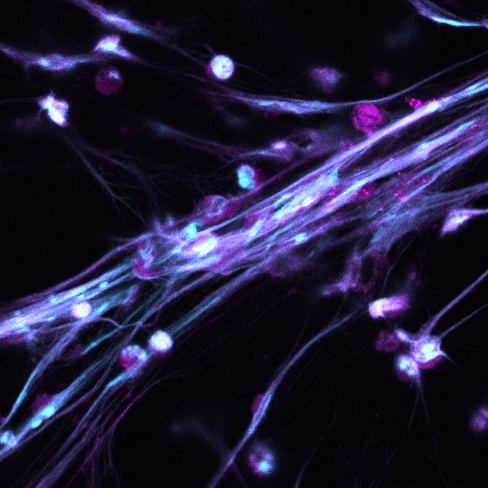

Supplement: Supplementary file 5 — Source data Fig. 2 [file 44319_2024_150_MOESM5_ESM.zip › Main Figure 2/Fig 2D/FB-124 WT and TLR13 KO/WT PMA/comp new.png]

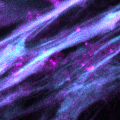

Supplement: Supplementary file 5 — Source data Fig. 2 [file 44319_2024_150_MOESM5_ESM.zip › Main Figure 2/Fig 2D/FB-124 WT and TLR13 KO/WT PMA/crop new.png]

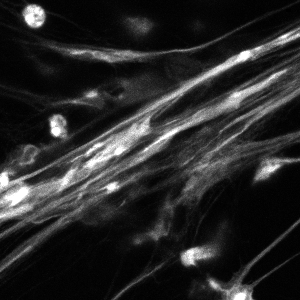

Supplement: Supplementary file 5 — Source data Fig. 2 [file 44319_2024_150_MOESM5_ESM.zip › Main Figure 2/Fig 2D/FB-124 WT and TLR13 KO/WT PMA/gray.png]

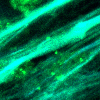

Supplement: Supplementary file 5 — Source data Fig. 2 [file 44319_2024_150_MOESM5_ESM.zip › Main Figure 2/Fig 2D/FB-124 WT and TLR13 KO/WT PMA/new crop.png]

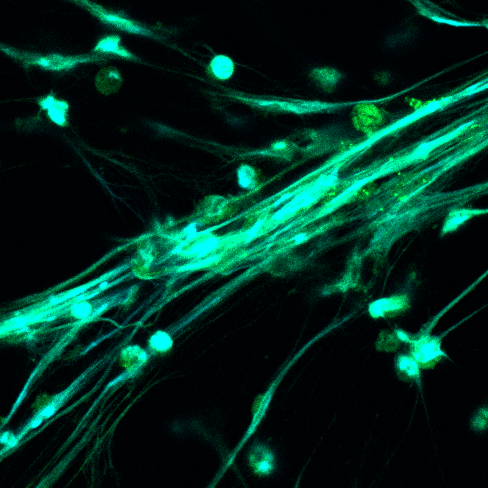

Supplement: Supplementary file 5 — Source data Fig. 2 [file 44319_2024_150_MOESM5_ESM.zip › Main Figure 2/Fig 2D/FB-124 WT and TLR13 KO/WT PMA/new.png]

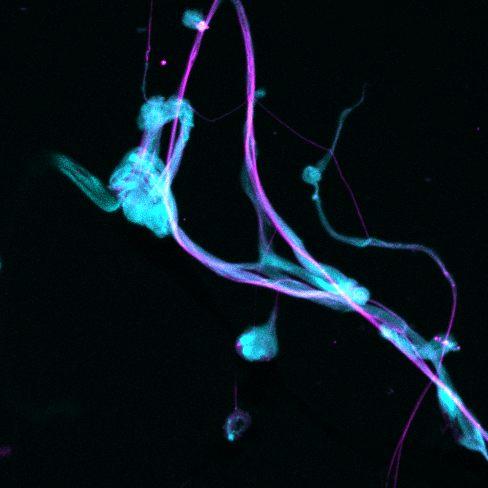

Supplement: Supplementary file 5 — Source data Fig. 2 [file 44319_2024_150_MOESM5_ESM.zip › Main Figure 2/Fig 2D/FB-124 WT and TLR13 KO/WT PMA NETs/comp new.png]

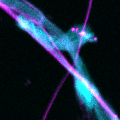

Supplement: Supplementary file 5 — Source data Fig. 2 [file 44319_2024_150_MOESM5_ESM.zip › Main Figure 2/Fig 2D/FB-124 WT and TLR13 KO/WT PMA NETs/crop new.png]

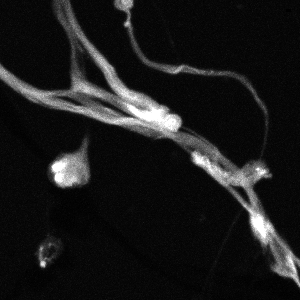

Supplement: Supplementary file 5 — Source data Fig. 2 [file 44319_2024_150_MOESM5_ESM.zip › Main Figure 2/Fig 2D/FB-124 WT and TLR13 KO/WT PMA NETs/gray.png]

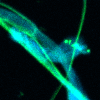

Supplement: Supplementary file 5 — Source data Fig. 2 [file 44319_2024_150_MOESM5_ESM.zip › Main Figure 2/Fig 2D/FB-124 WT and TLR13 KO/WT PMA NETs/new crop.png]

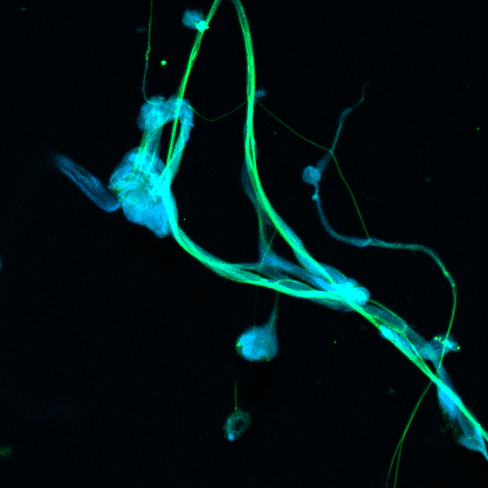

Supplement: Supplementary file 5 — Source data Fig. 2 [file 44319_2024_150_MOESM5_ESM.zip › Main Figure 2/Fig 2D/FB-124 WT and TLR13 KO/WT PMA NETs/new.png]

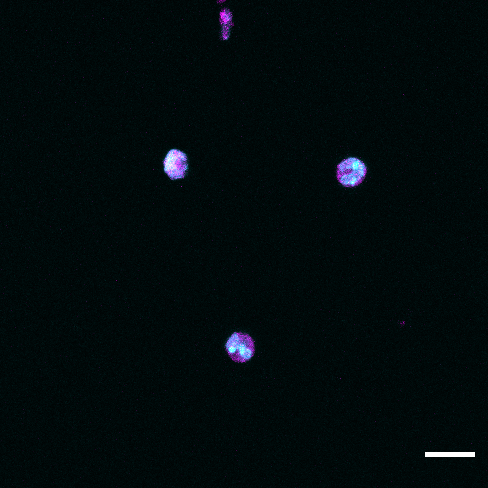

Supplement: Supplementary file 5 — Source data Fig. 2 [file 44319_2024_150_MOESM5_ESM.zip › Main Figure 2/Fig 2D/FB-124 WT and TLR13 KO/WT unstim/como new.png]

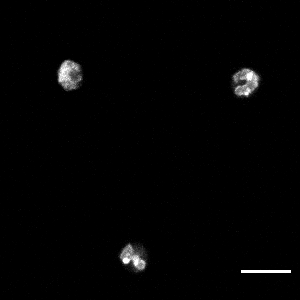

Supplement: Supplementary file 5 — Source data Fig. 2 [file 44319_2024_150_MOESM5_ESM.zip › Main Figure 2/Fig 2D/FB-124 WT and TLR13 KO/WT unstim/gray.png]

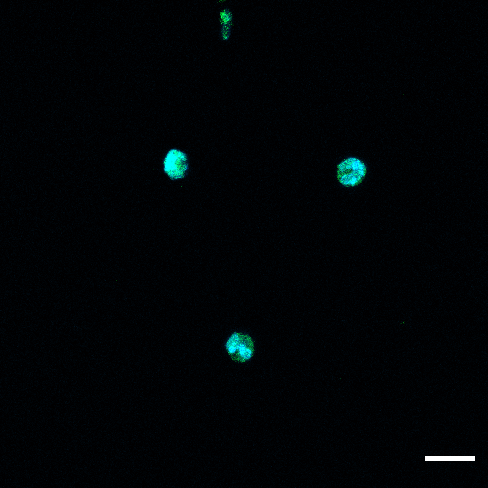

Supplement: Supplementary file 5 — Source data Fig. 2 [file 44319_2024_150_MOESM5_ESM.zip › Main Figure 2/Fig 2D/FB-124 WT and TLR13 KO/WT unstim/MAX_Experiment-2539-Airyscan Processing-36.png]

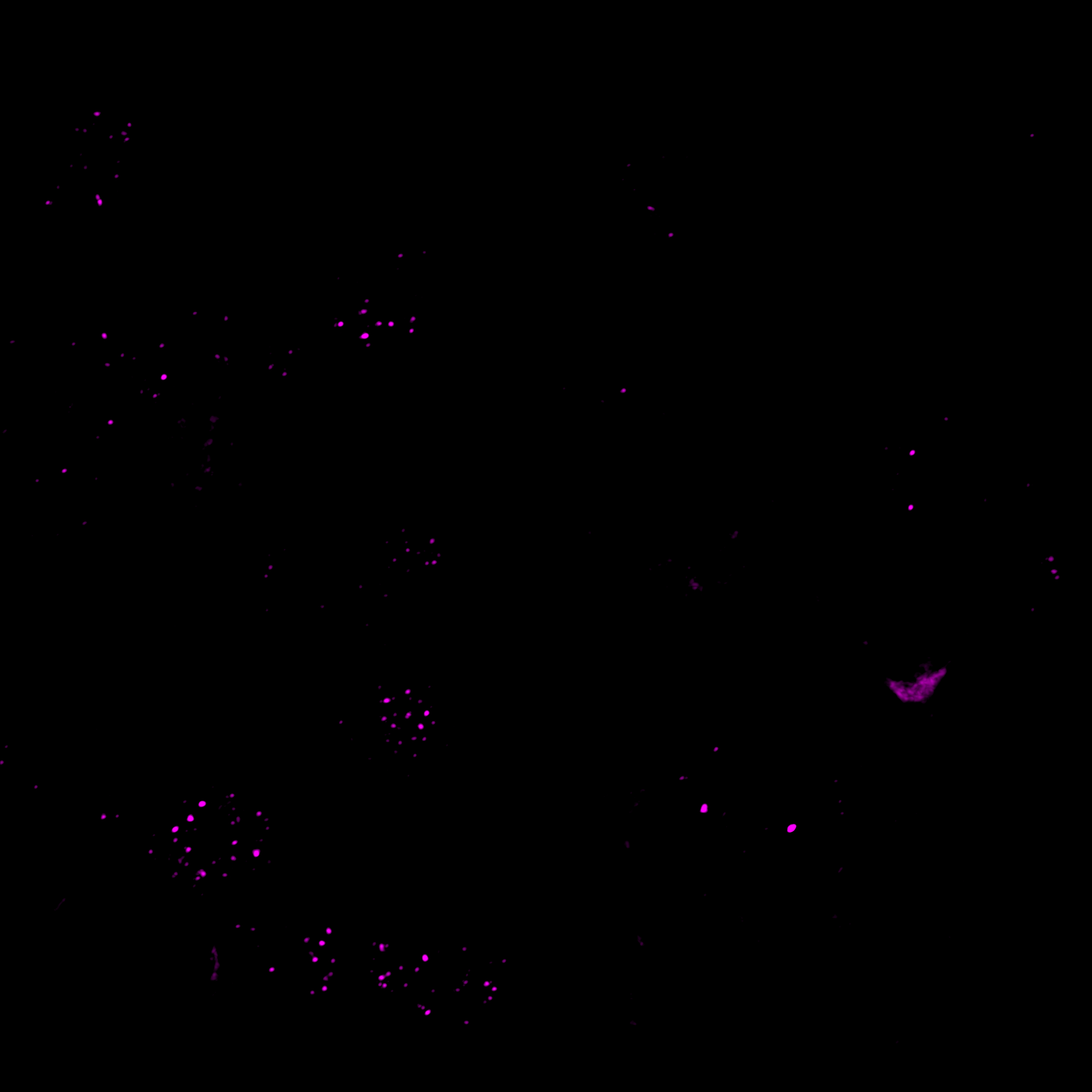

Supplement: Supplementary file 5 — Source data Fig. 2 [file 44319_2024_150_MOESM5_ESM.zip › Main Figure 2/Fig 2H/VNL 035 images/LPS+Nig/asc.png]

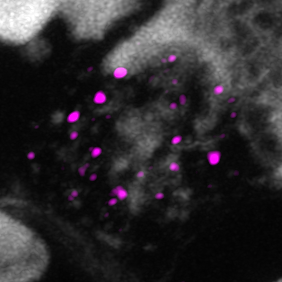

Supplement: Supplementary file 5 — Source data Fig. 2 [file 44319_2024_150_MOESM5_ESM.zip › Main Figure 2/Fig 2H/VNL 035 images/LPS+Nig/Composite crop.png]

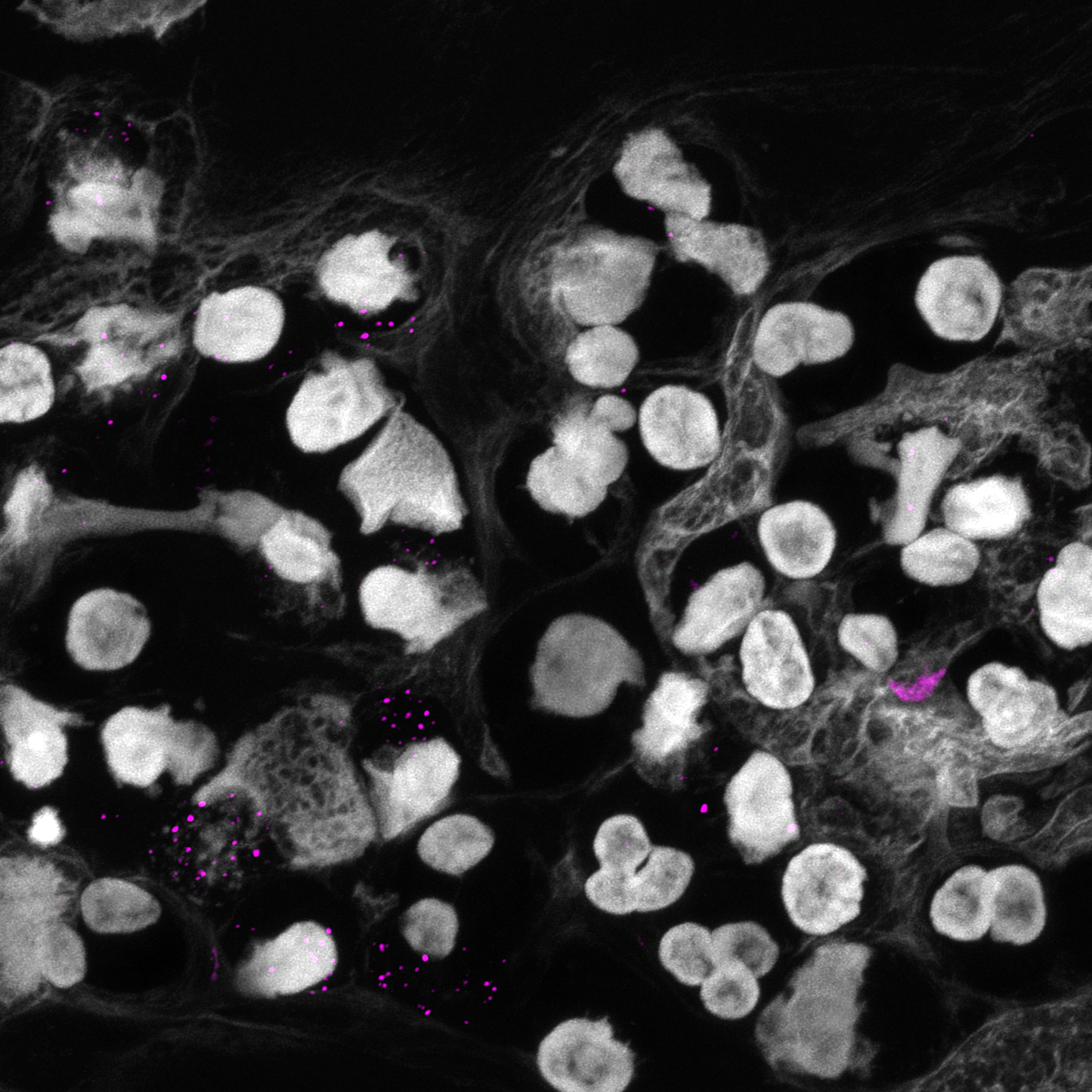

Supplement: Supplementary file 5 — Source data Fig. 2 [file 44319_2024_150_MOESM5_ESM.zip › Main Figure 2/Fig 2H/VNL 035 images/LPS+Nig/Composite.png]

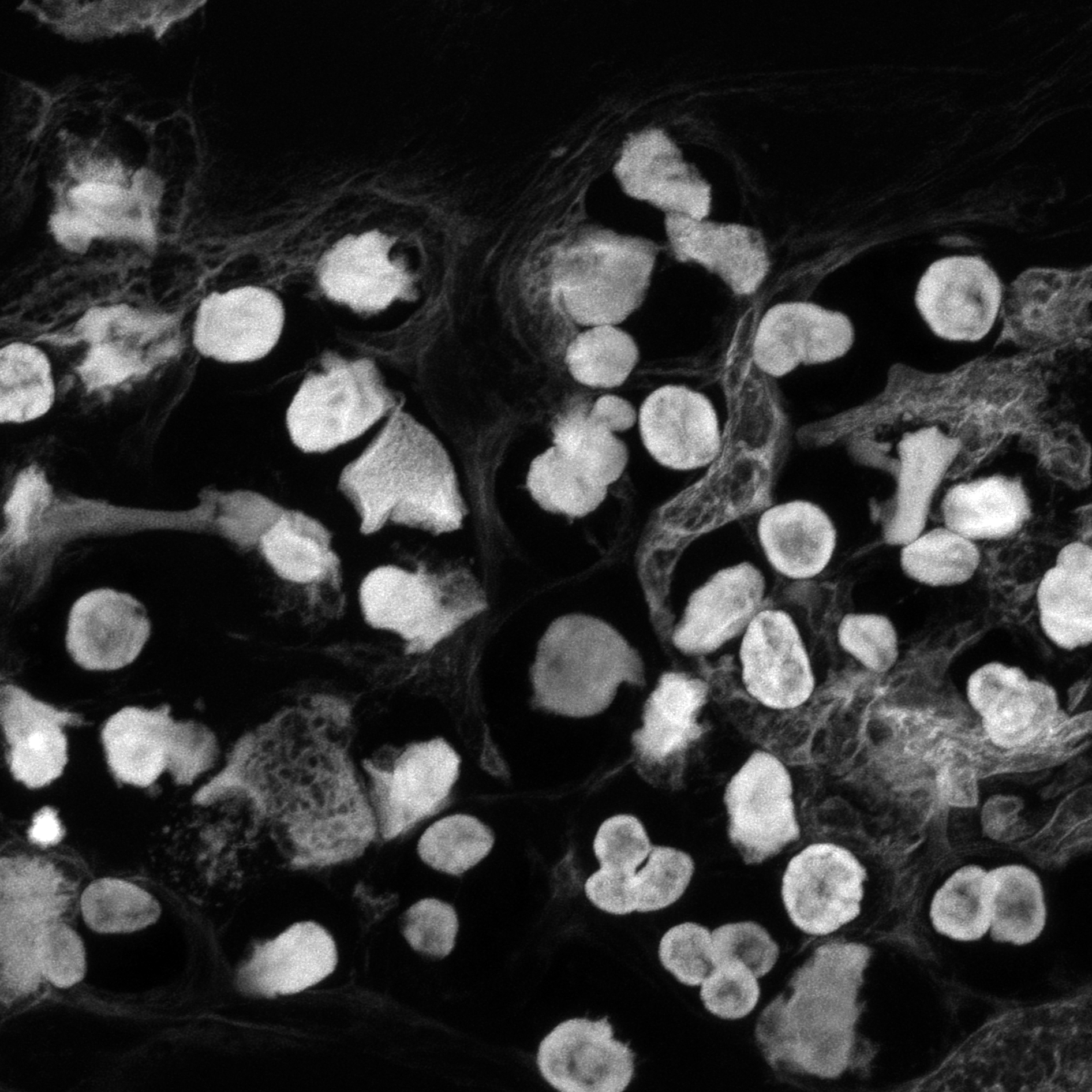

Supplement: Supplementary file 5 — Source data Fig. 2 [file 44319_2024_150_MOESM5_ESM.zip › Main Figure 2/Fig 2H/VNL 035 images/LPS+Nig/hoechst.png]

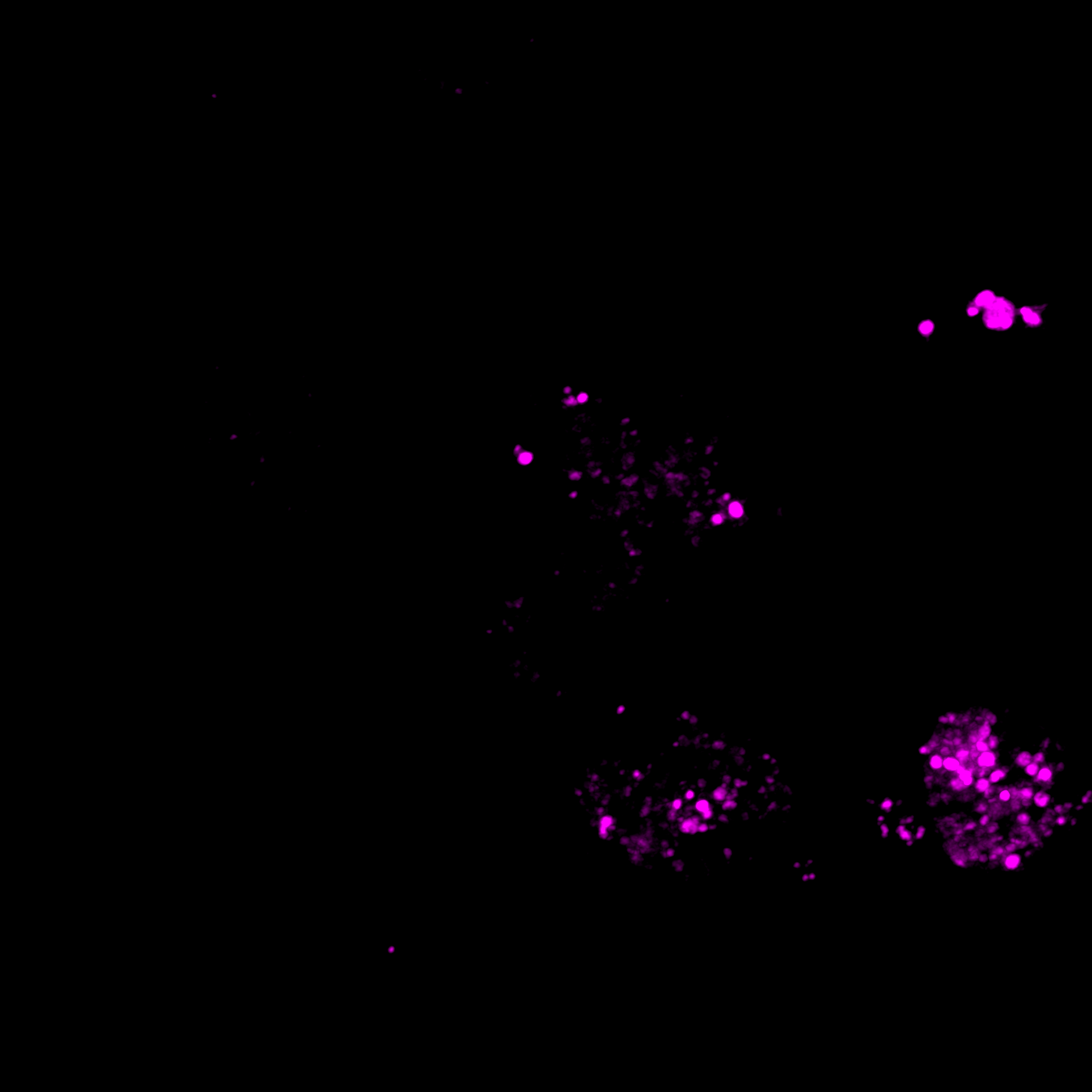

Supplement: Supplementary file 5 — Source data Fig. 2 [file 44319_2024_150_MOESM5_ESM.zip › Main Figure 2/Fig 2H/VNL 035 images/PMA NETs/asc.png]

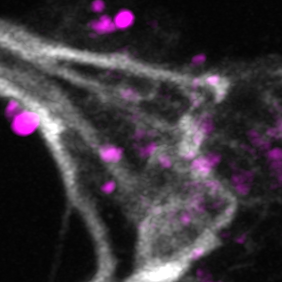

Supplement: Supplementary file 5 — Source data Fig. 2 [file 44319_2024_150_MOESM5_ESM.zip › Main Figure 2/Fig 2H/VNL 035 images/PMA NETs/Composite crop.png]

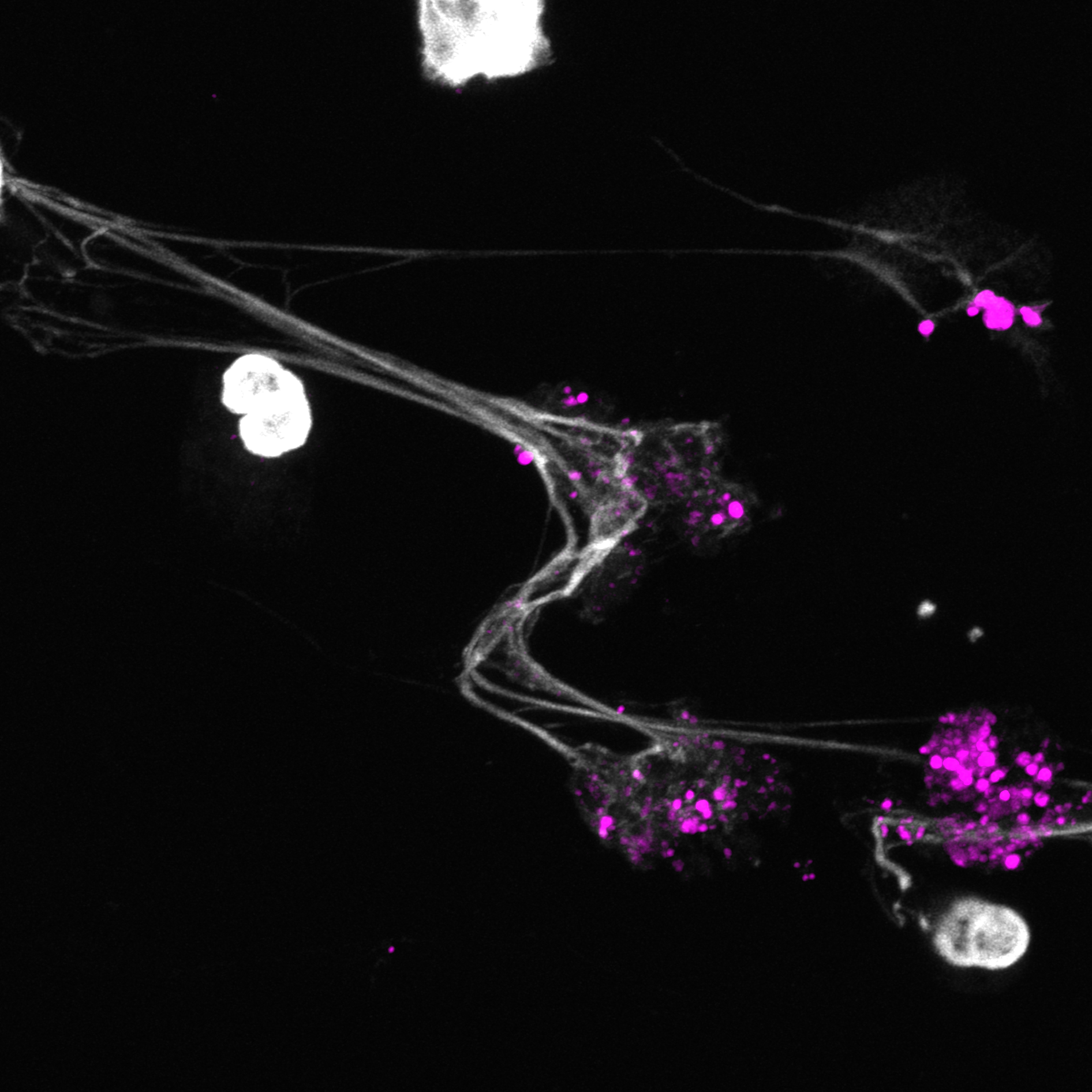

Supplement: Supplementary file 5 — Source data Fig. 2 [file 44319_2024_150_MOESM5_ESM.zip › Main Figure 2/Fig 2H/VNL 035 images/PMA NETs/Composite.png]

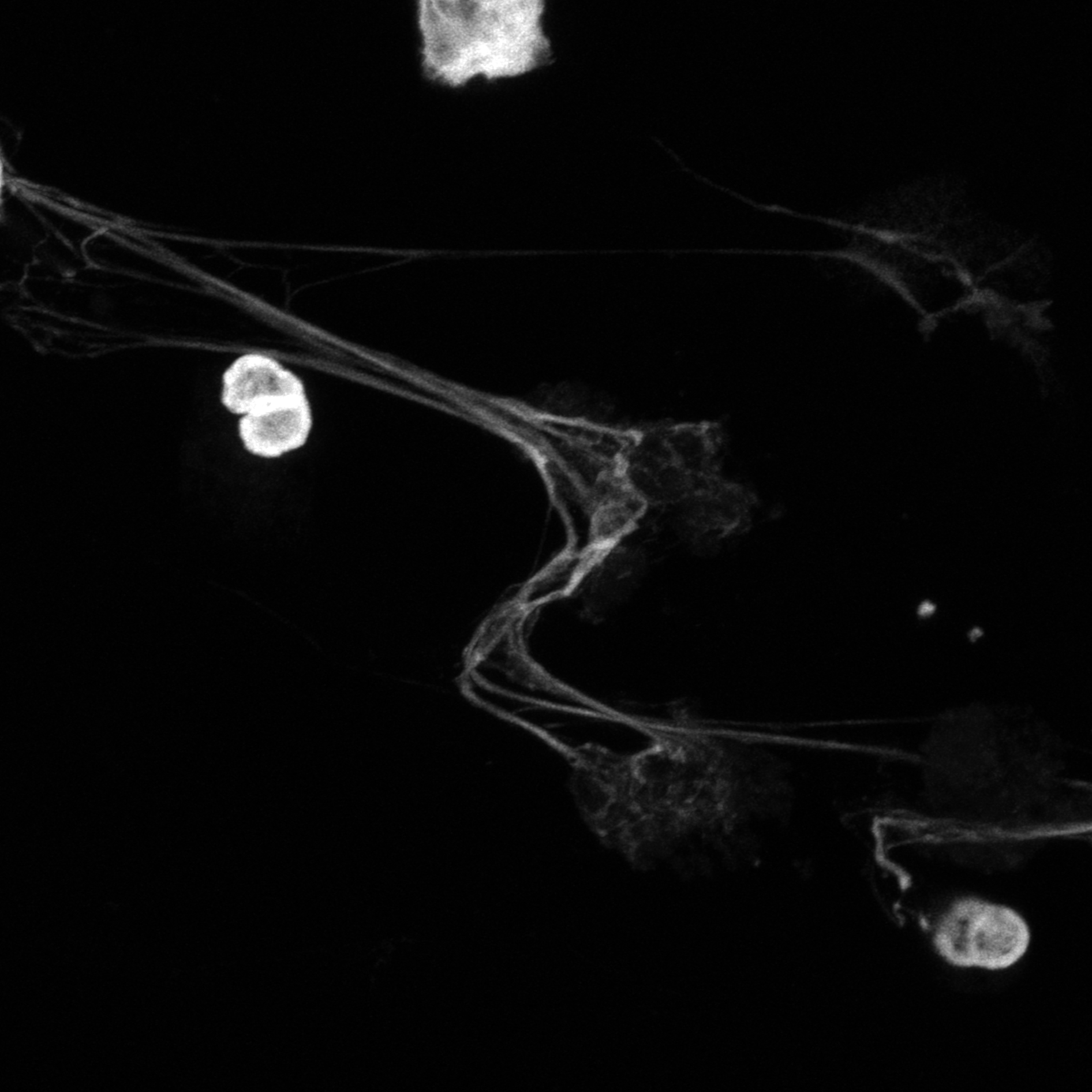

Supplement: Supplementary file 5 — Source data Fig. 2 [file 44319_2024_150_MOESM5_ESM.zip › Main Figure 2/Fig 2H/VNL 035 images/PMA NETs/hoechst.png]

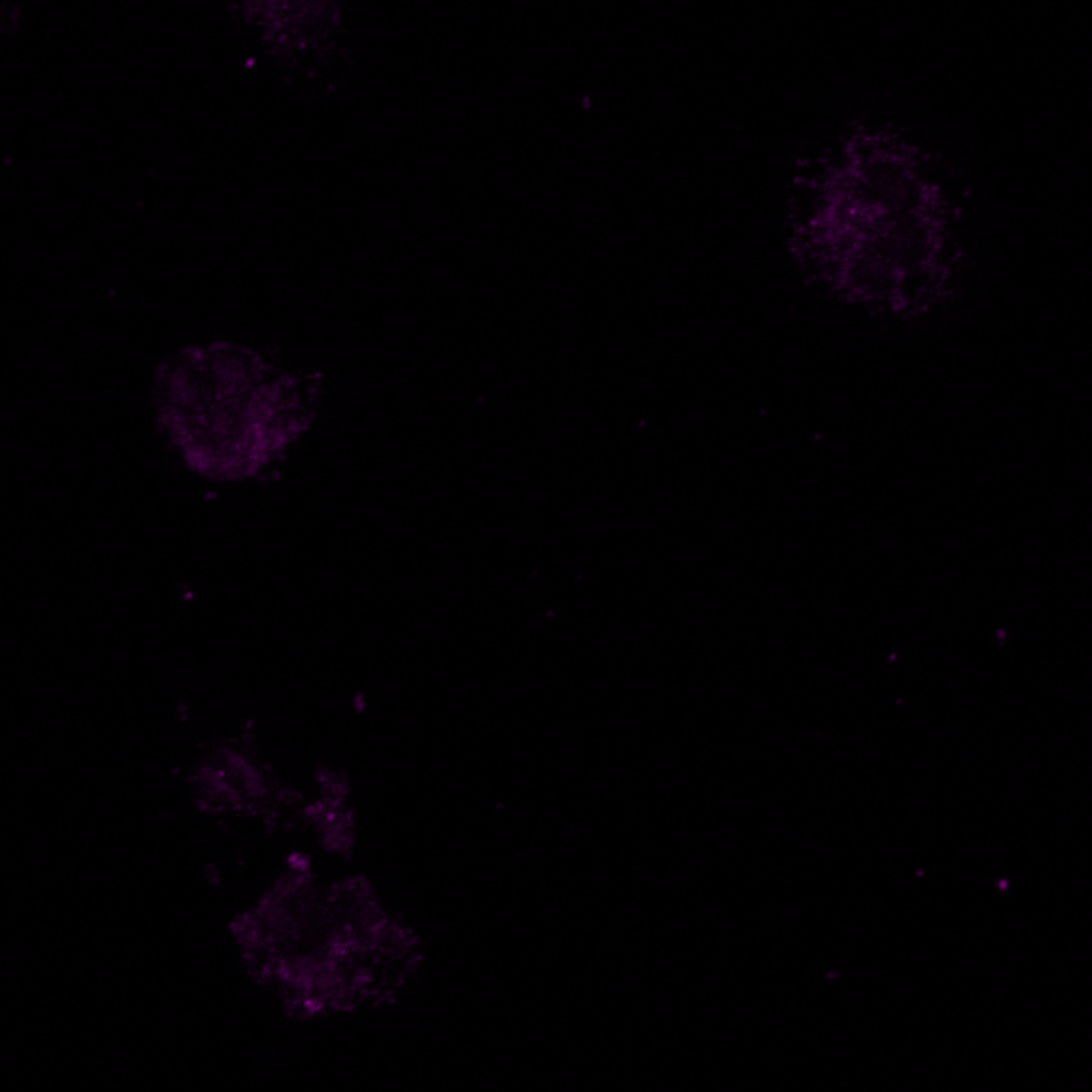

Supplement: Supplementary file 5 — Source data Fig. 2 [file 44319_2024_150_MOESM5_ESM.zip › Main Figure 2/Fig 2H/VNL 035 images/PMA NETs + MCC/asc.png]

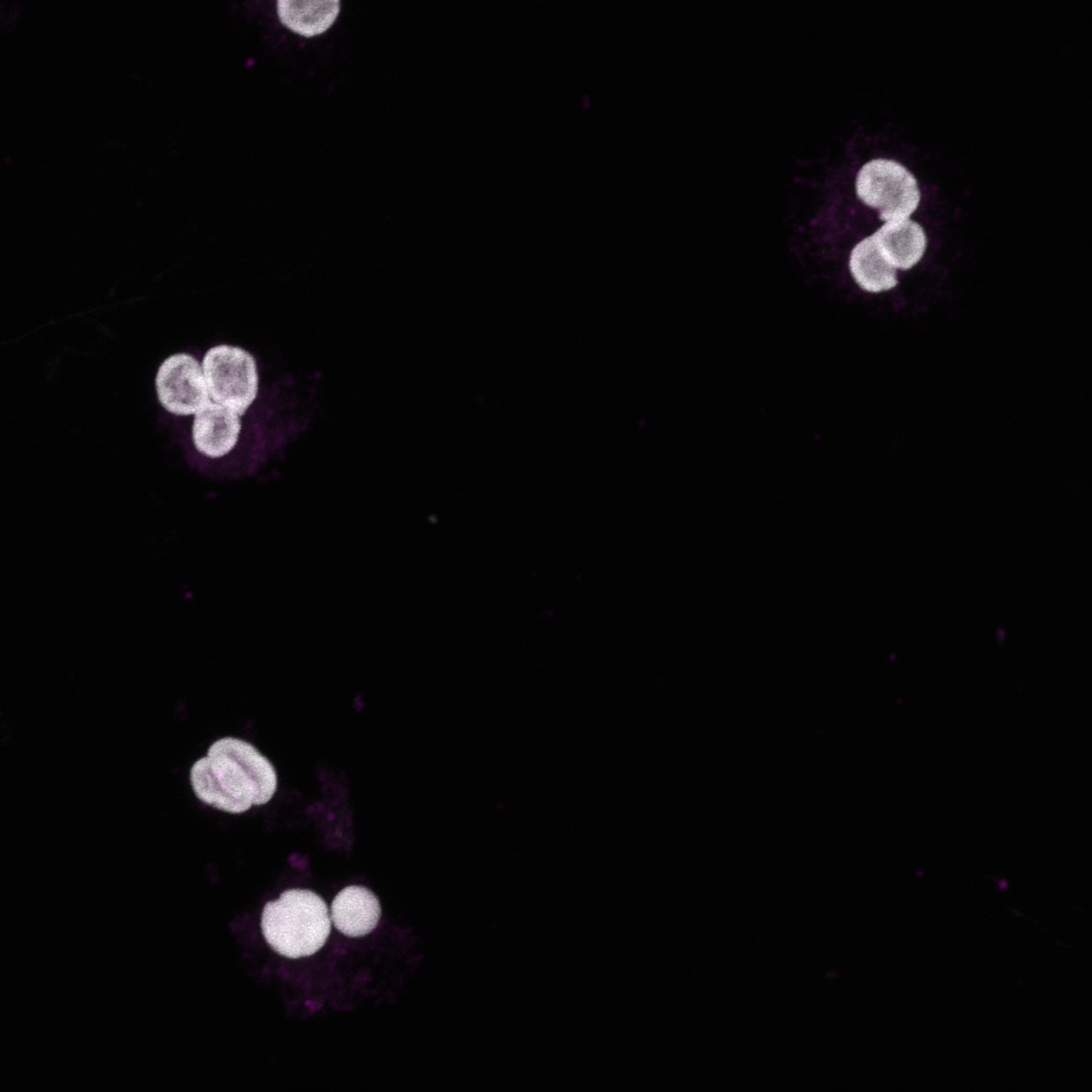

Supplement: Supplementary file 5 — Source data Fig. 2 [file 44319_2024_150_MOESM5_ESM.zip › Main Figure 2/Fig 2H/VNL 035 images/PMA NETs + MCC/Composite.png]

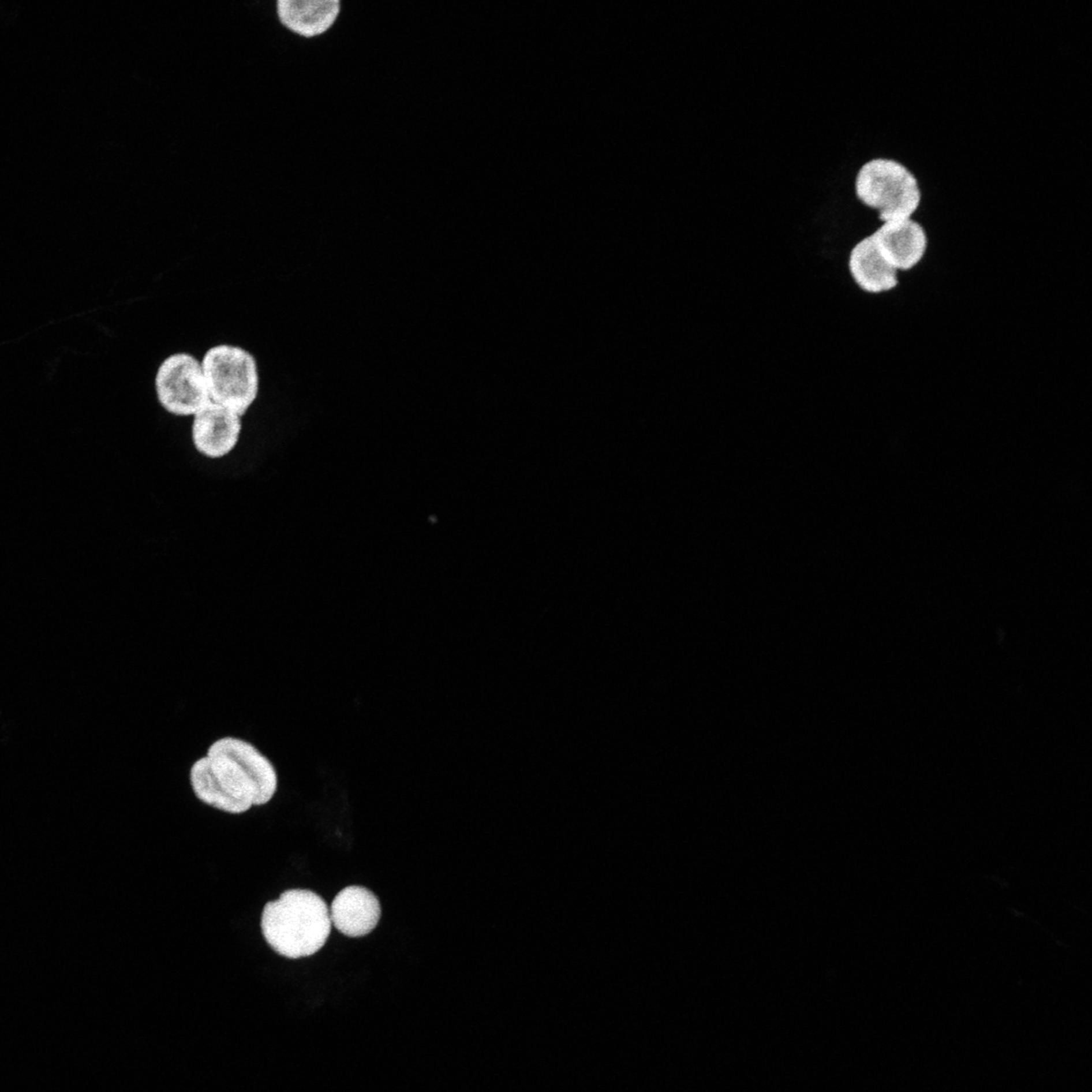

Supplement: Supplementary file 5 — Source data Fig. 2 [file 44319_2024_150_MOESM5_ESM.zip › Main Figure 2/Fig 2H/VNL 035 images/PMA NETs + MCC/hoechst.png]

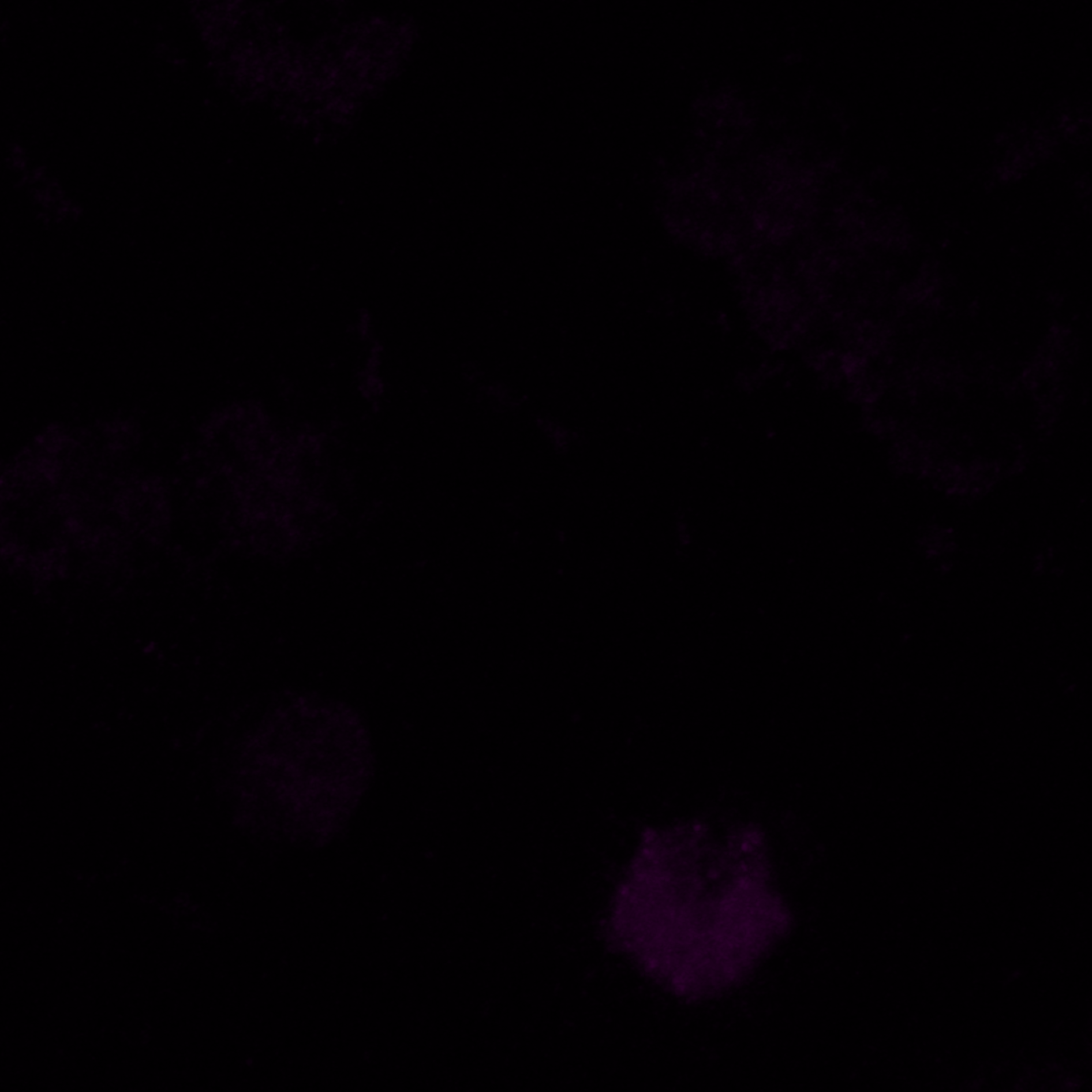

Supplement: Supplementary file 5 — Source data Fig. 2 [file 44319_2024_150_MOESM5_ESM.zip › Main Figure 2/Fig 2H/VNL 035 images/unstim/asc.png]

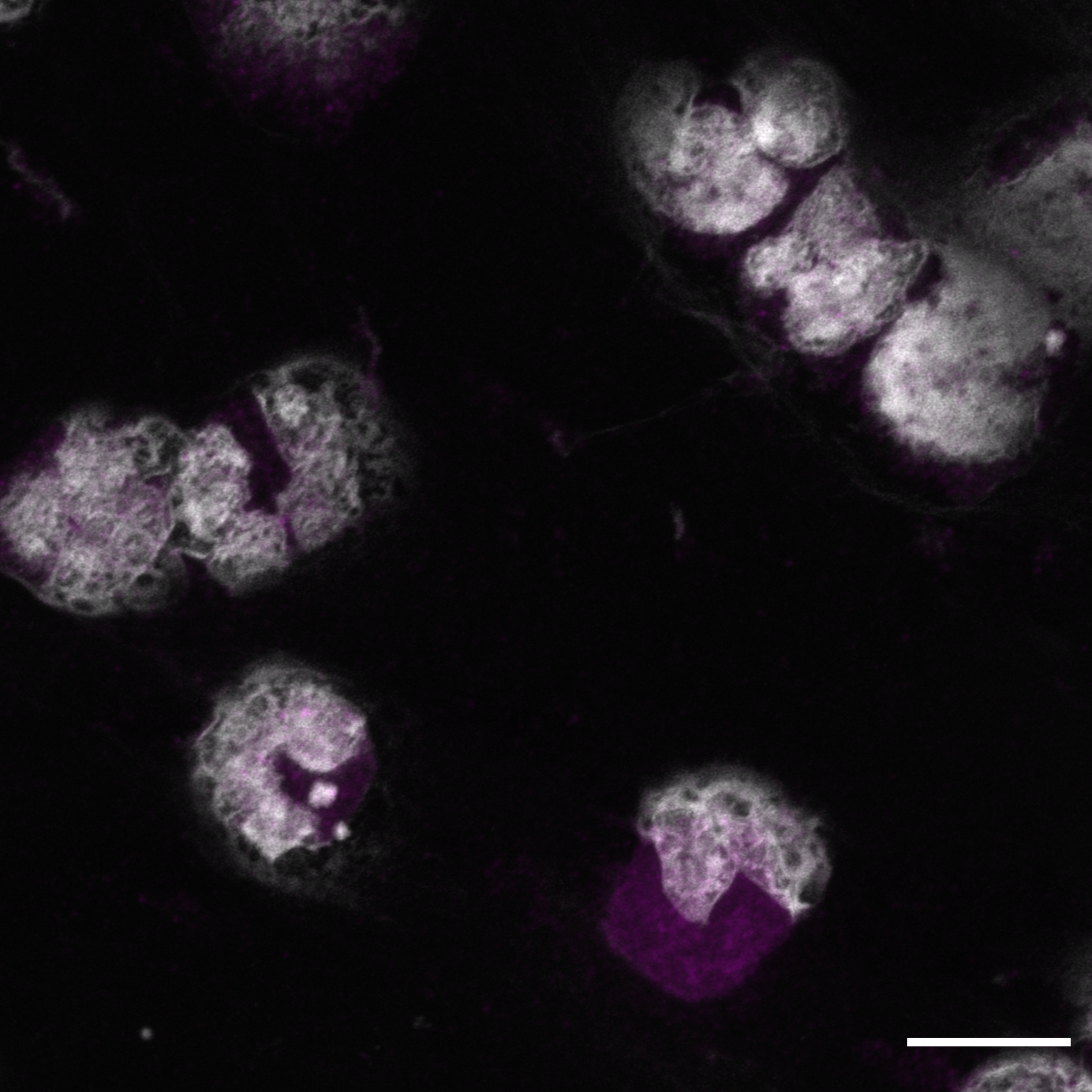

Supplement: Supplementary file 5 — Source data Fig. 2 [file 44319_2024_150_MOESM5_ESM.zip › Main Figure 2/Fig 2H/VNL 035 images/unstim/Composite.png]

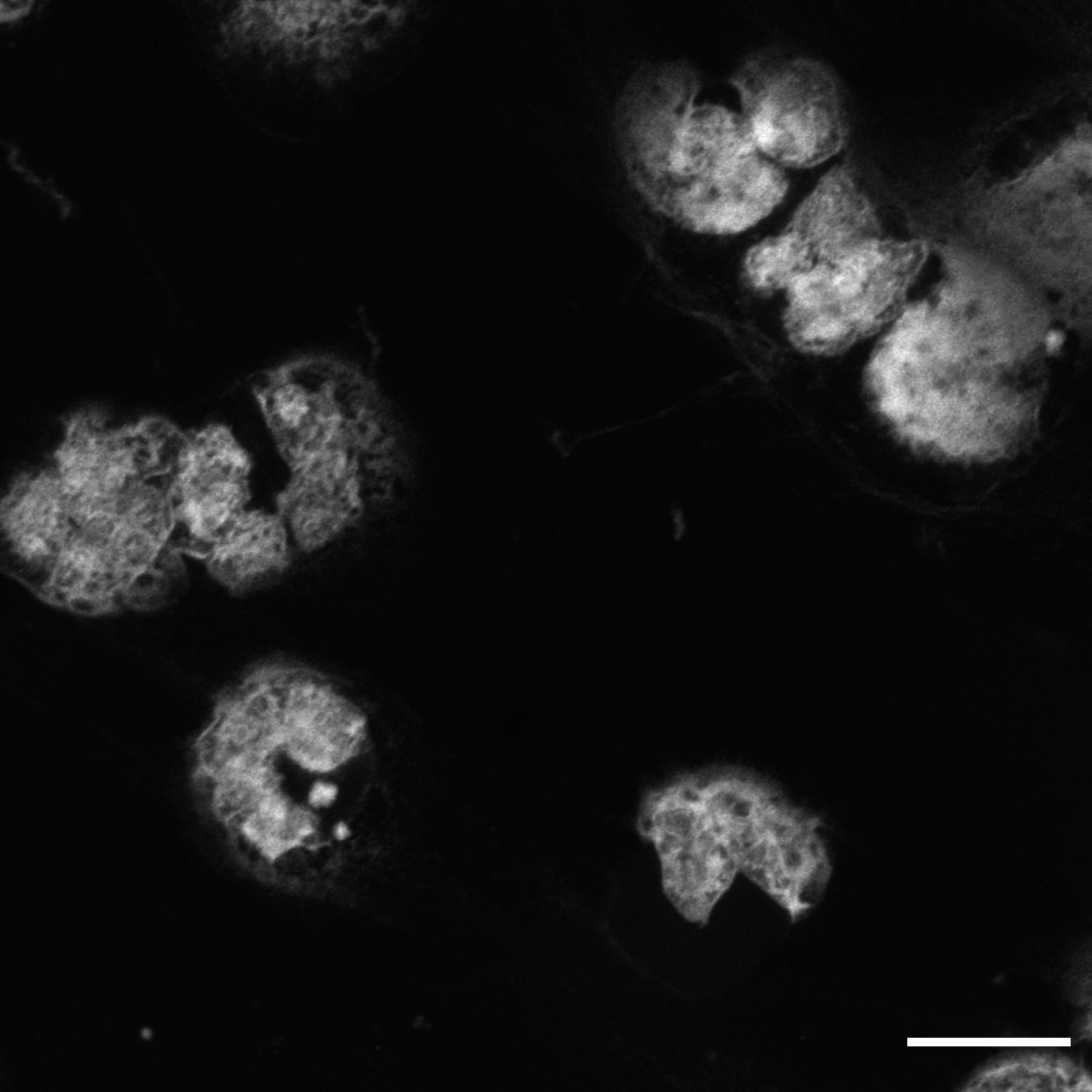

Supplement: Supplementary file 5 — Source data Fig. 2 [file 44319_2024_150_MOESM5_ESM.zip › Main Figure 2/Fig 2H/VNL 035 images/unstim/Hoechst.png]

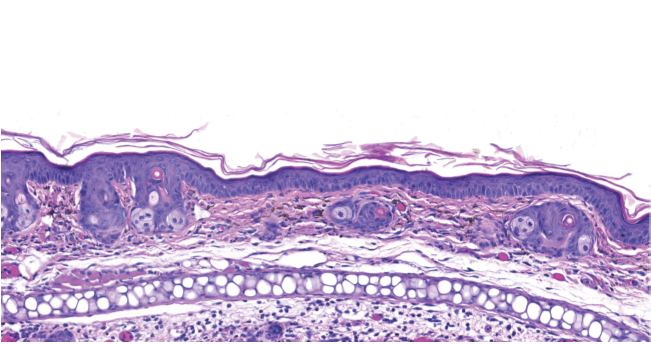

Supplement: Supplementary file 7 — Source data Fig. 4 [file 44319_2024_150_MOESM7_ESM.zip › Main Figure 4/Fig 4F/Tlr13 KO.png]

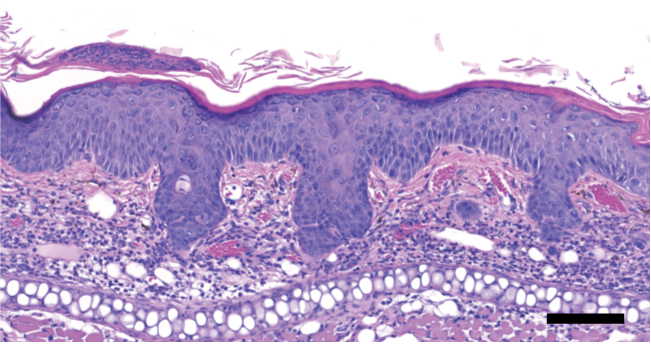

Supplement: Supplementary file 7 — Source data Fig. 4 [file 44319_2024_150_MOESM7_ESM.zip › Main Figure 4/Fig 4F/WT.png]

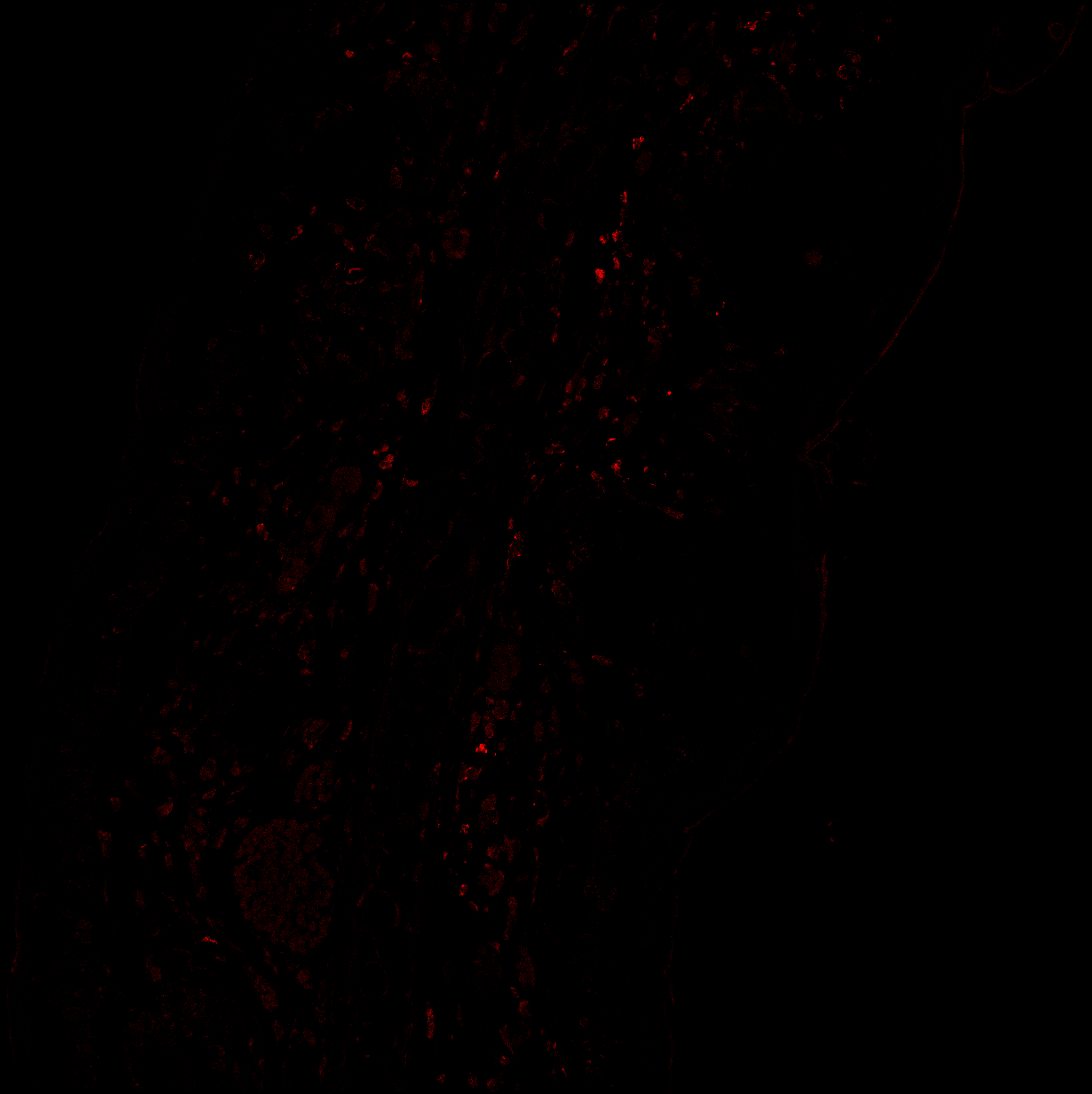

Supplement: Supplementary file 7 — Source data Fig. 4 [file 44319_2024_150_MOESM7_ESM.zip › Main Figure 4/Fig 4G/C1-slide 11 - IMQ - TLR13- 4.tif]

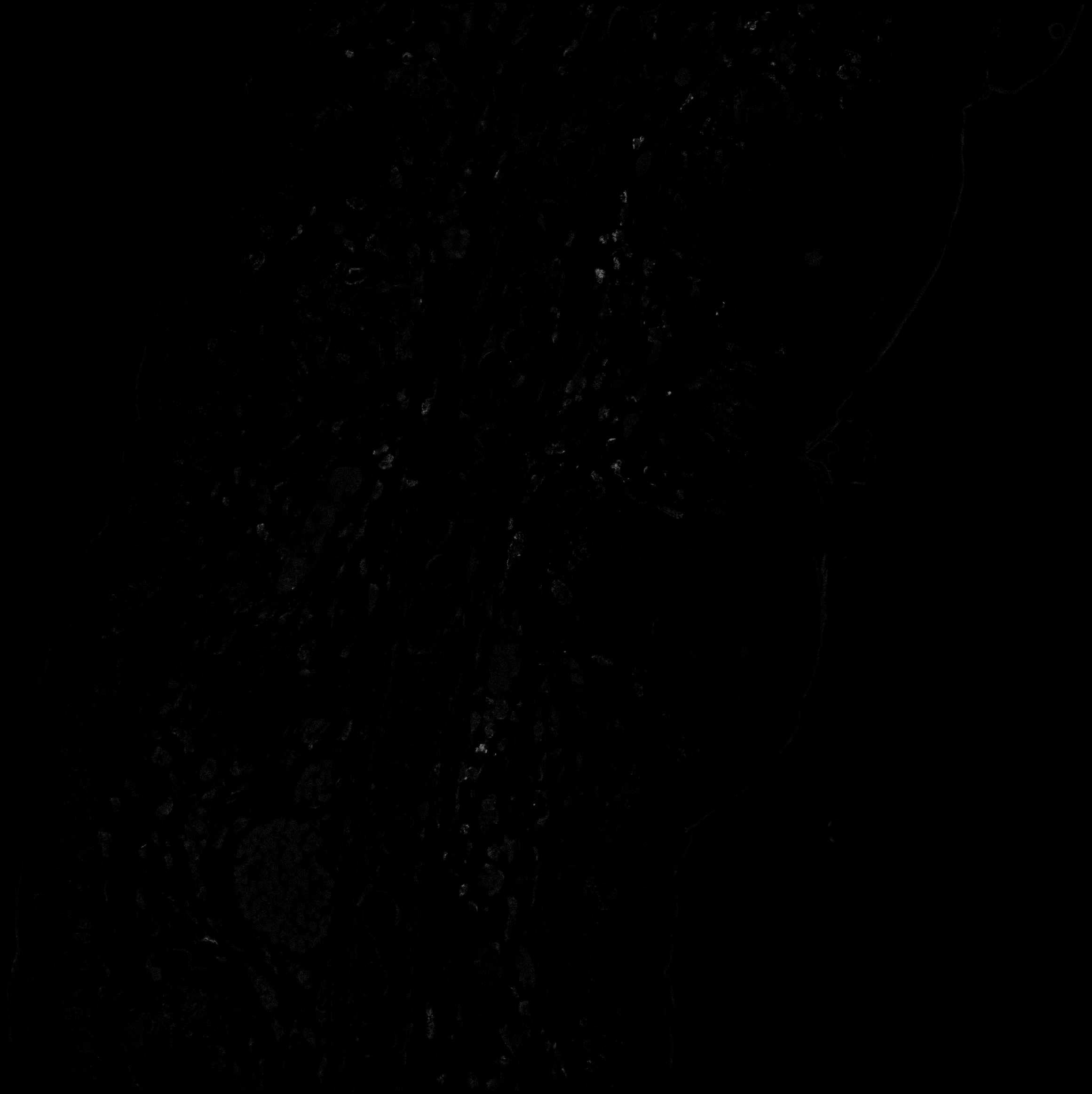

Supplement: Supplementary file 7 — Source data Fig. 4 [file 44319_2024_150_MOESM7_ESM.zip › Main Figure 4/Fig 4G/C1-slide 11 - IMQ - TLR13- 4_sw.tif]

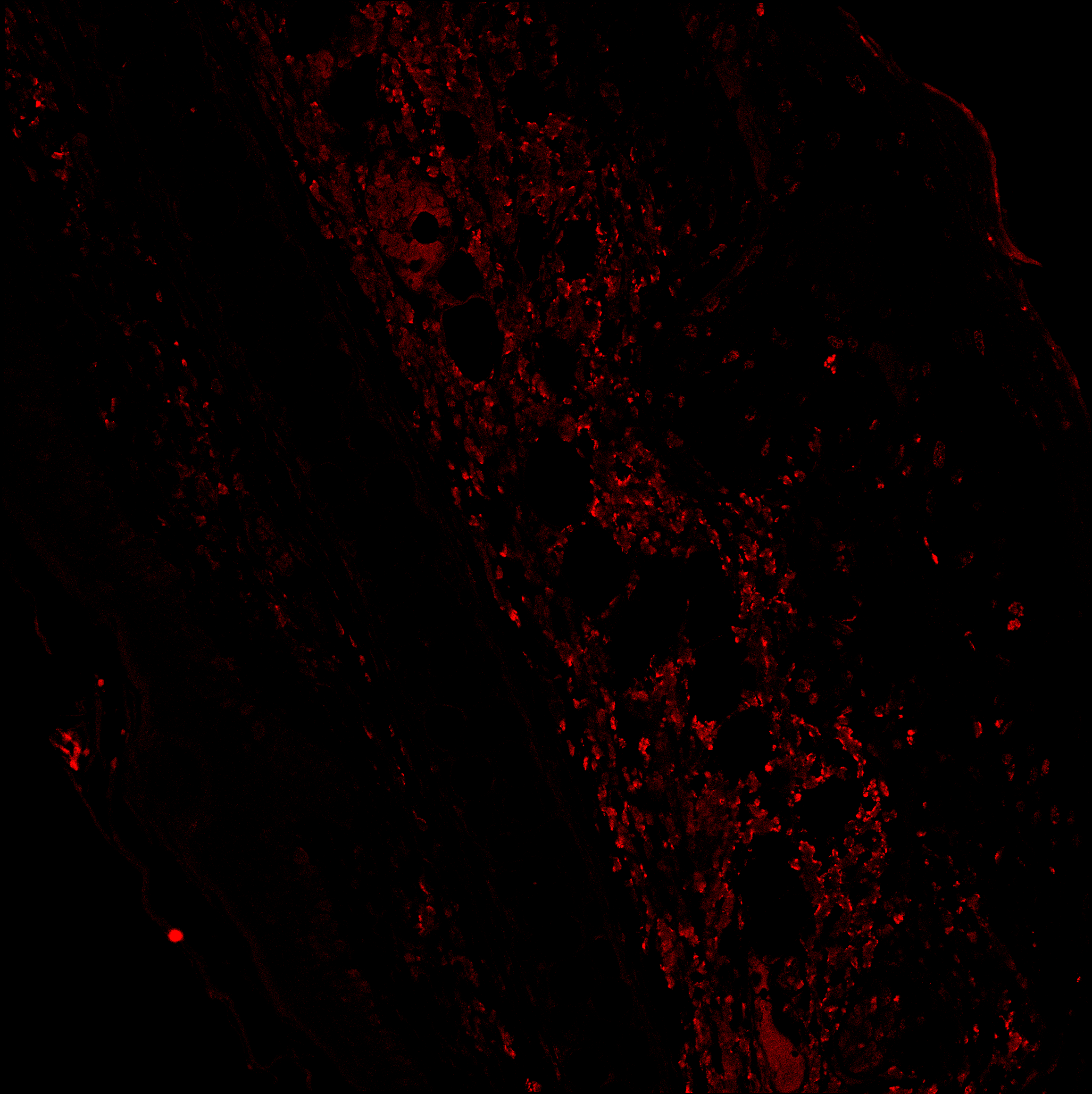

Supplement: Supplementary file 7 — Source data Fig. 4 [file 44319_2024_150_MOESM7_ESM.zip › Main Figure 4/Fig 4G/C1-slide 5 - IMQ - WT -1.tif]

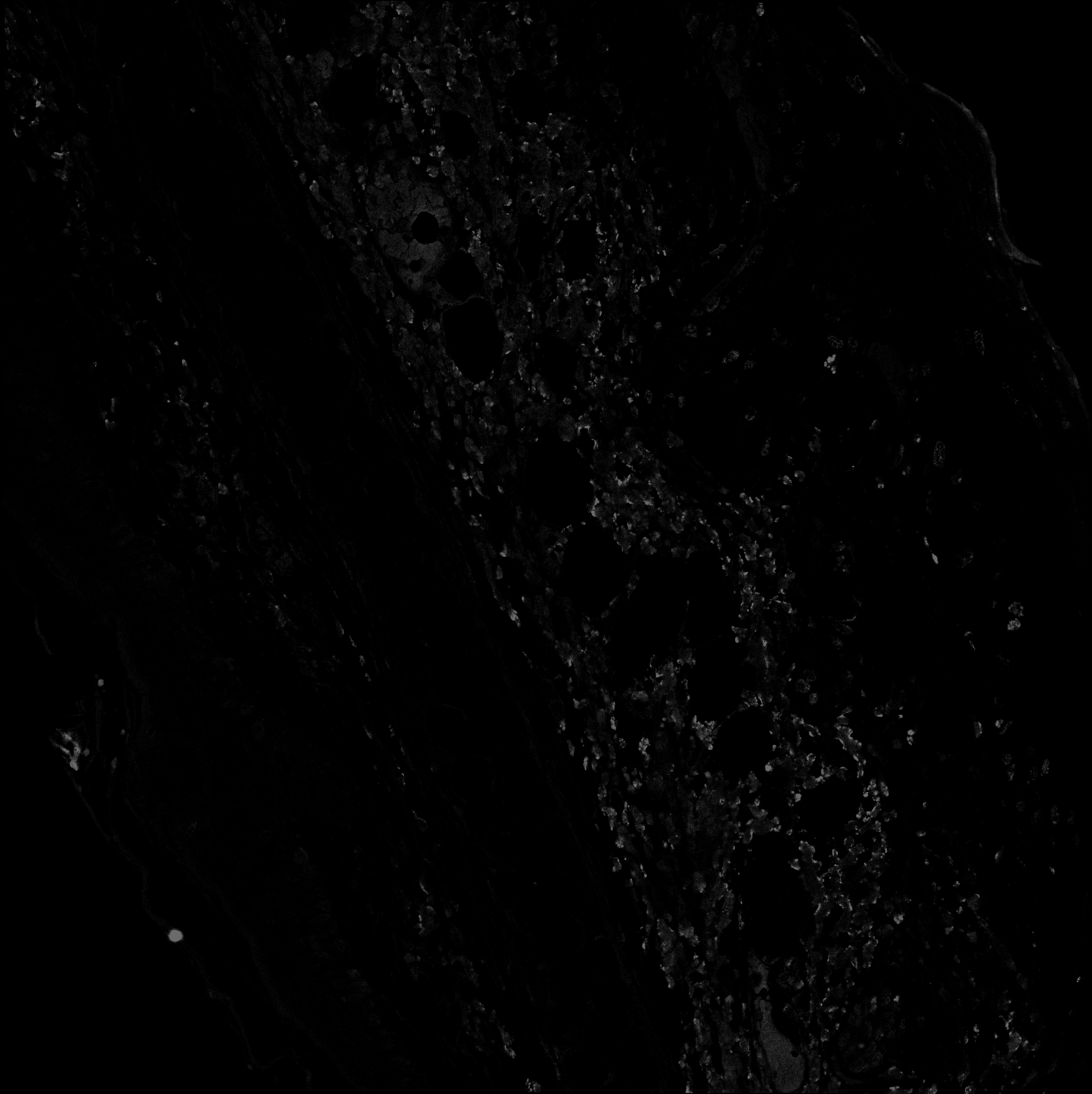

Supplement: Supplementary file 7 — Source data Fig. 4 [file 44319_2024_150_MOESM7_ESM.zip › Main Figure 4/Fig 4G/C1-slide 5 - IMQ - WT -1_sw.tif]

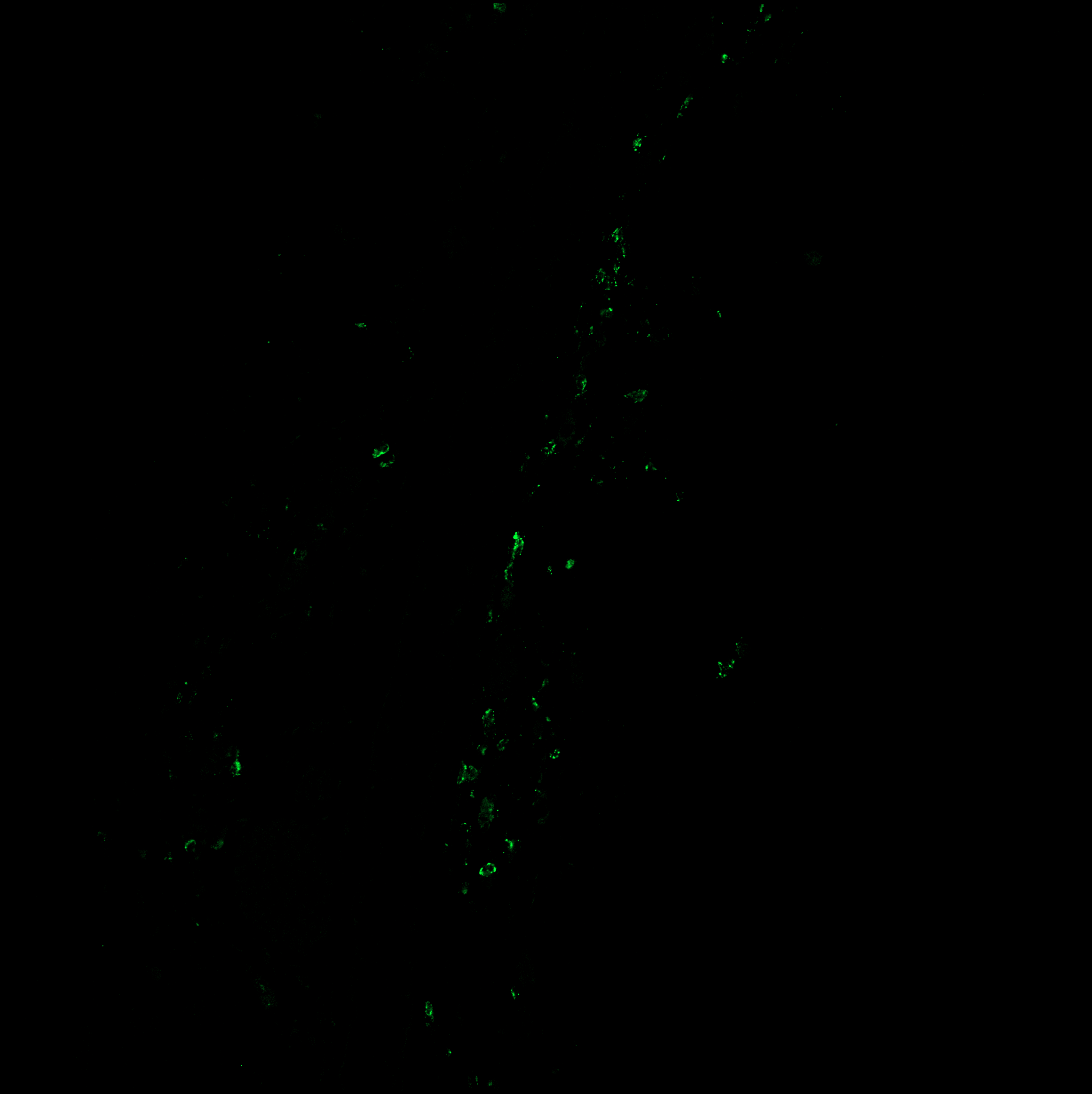

Supplement: Supplementary file 7 — Source data Fig. 4 [file 44319_2024_150_MOESM7_ESM.zip › Main Figure 4/Fig 4G/C2-slide 11 - IMQ - TLR13- 4.tif]

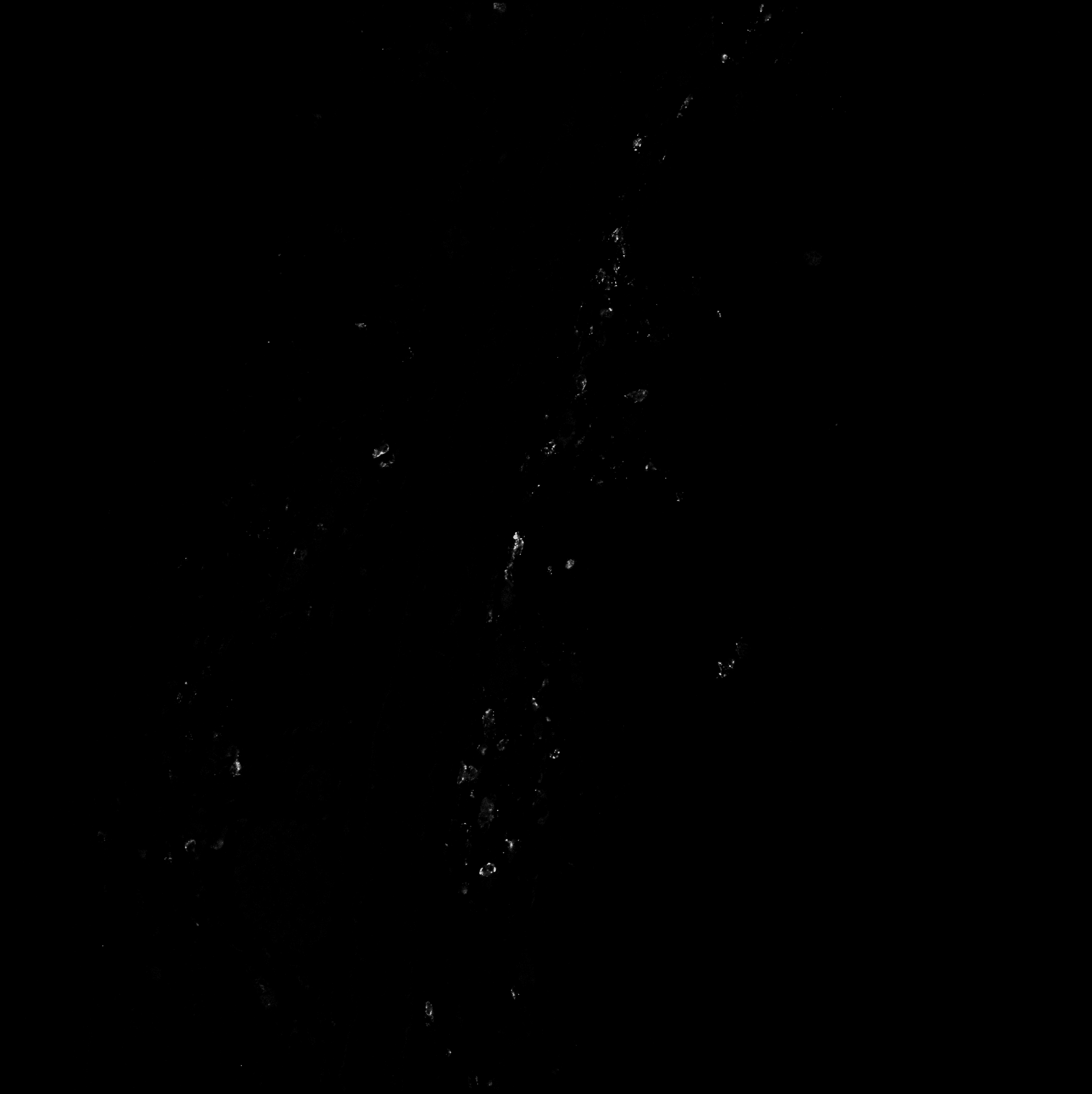

Supplement: Supplementary file 7 — Source data Fig. 4 [file 44319_2024_150_MOESM7_ESM.zip › Main Figure 4/Fig 4G/C2-slide 11 - IMQ - TLR13- 4_sw.tif]

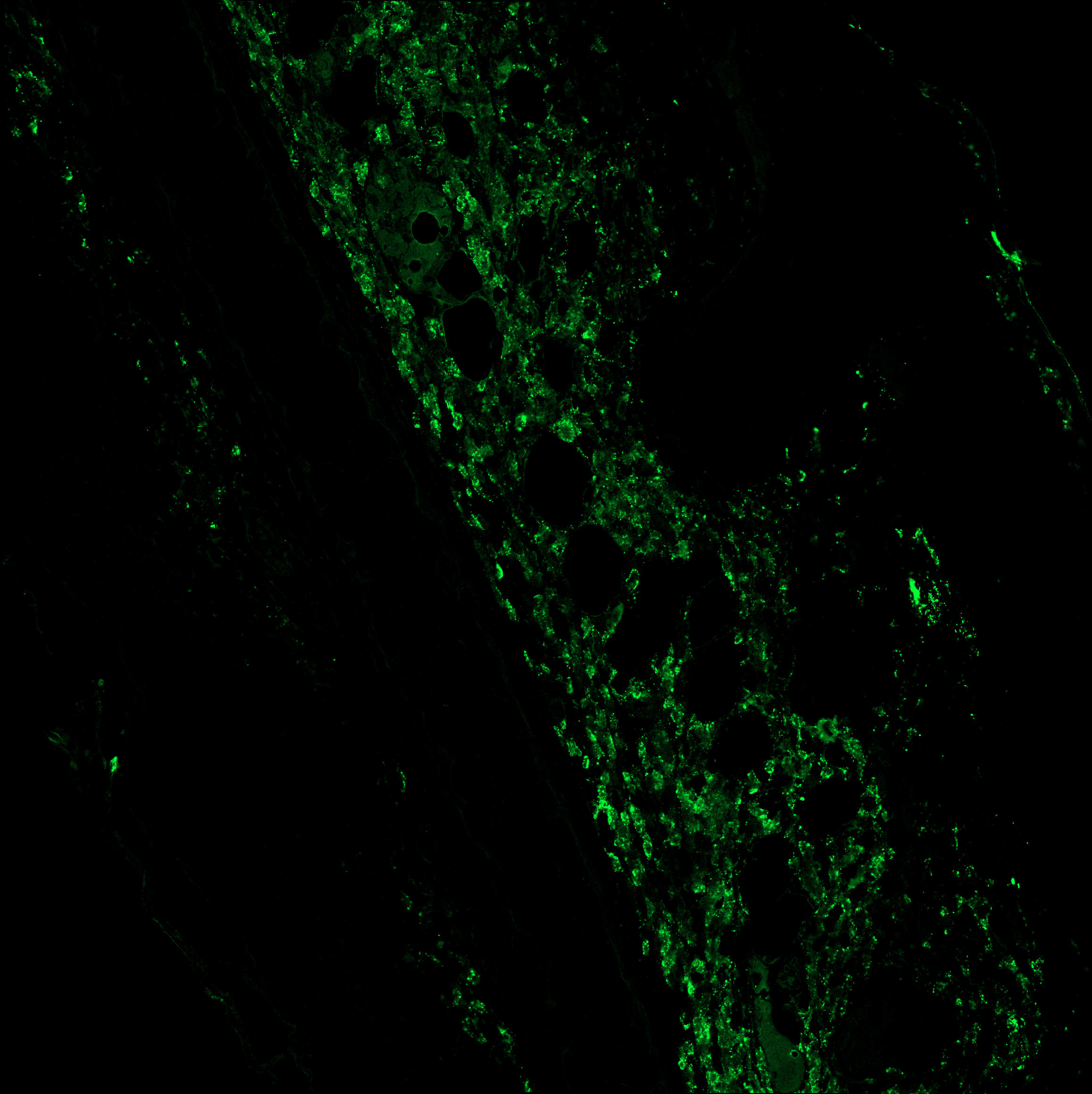

Supplement: Supplementary file 7 — Source data Fig. 4 [file 44319_2024_150_MOESM7_ESM.zip › Main Figure 4/Fig 4G/C2-slide 5 - IMQ - WT -1.tif]

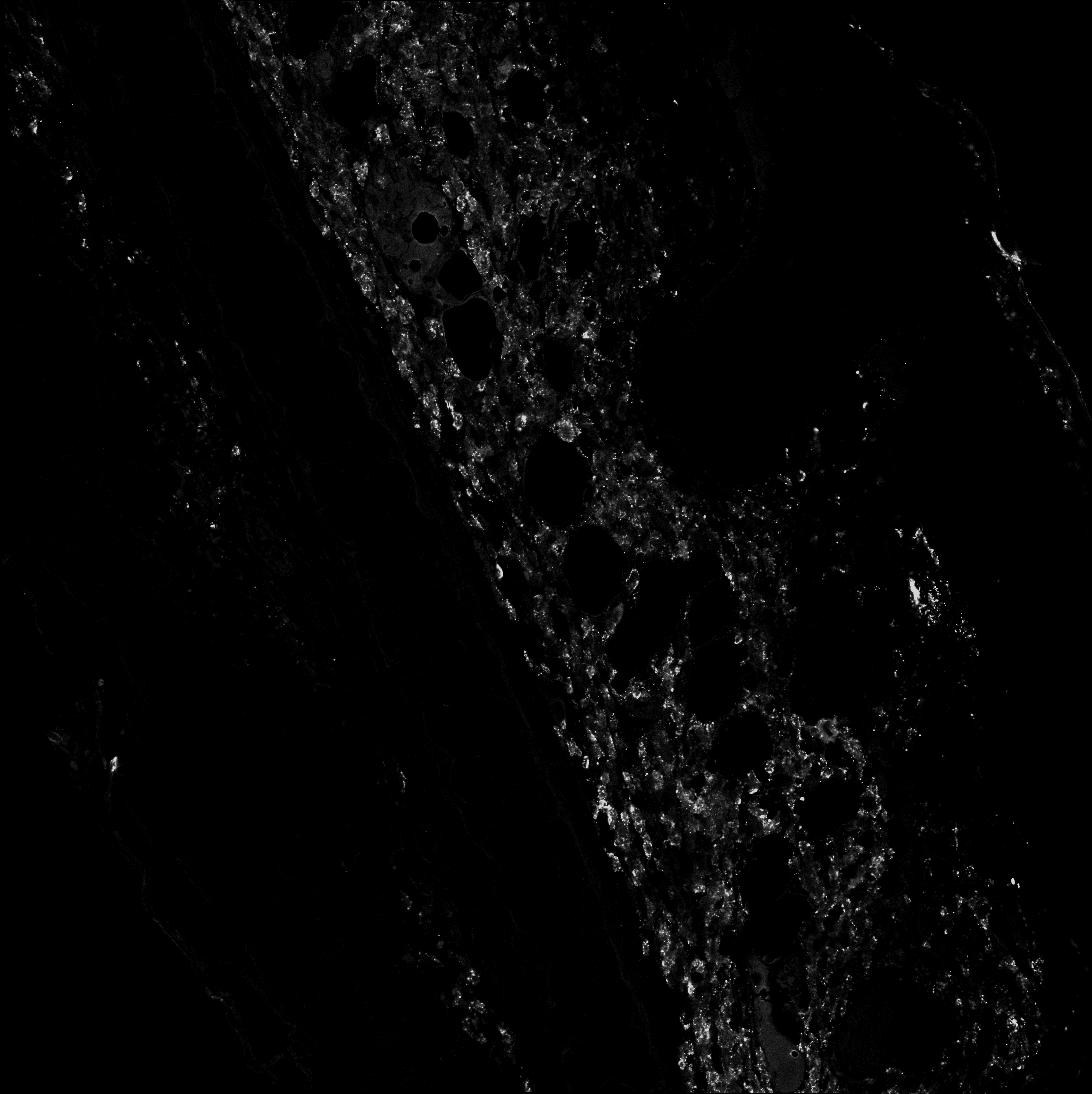

Supplement: Supplementary file 7 — Source data Fig. 4 [file 44319_2024_150_MOESM7_ESM.zip › Main Figure 4/Fig 4G/C2-slide 5 - IMQ - WT -1_Sw.tif]

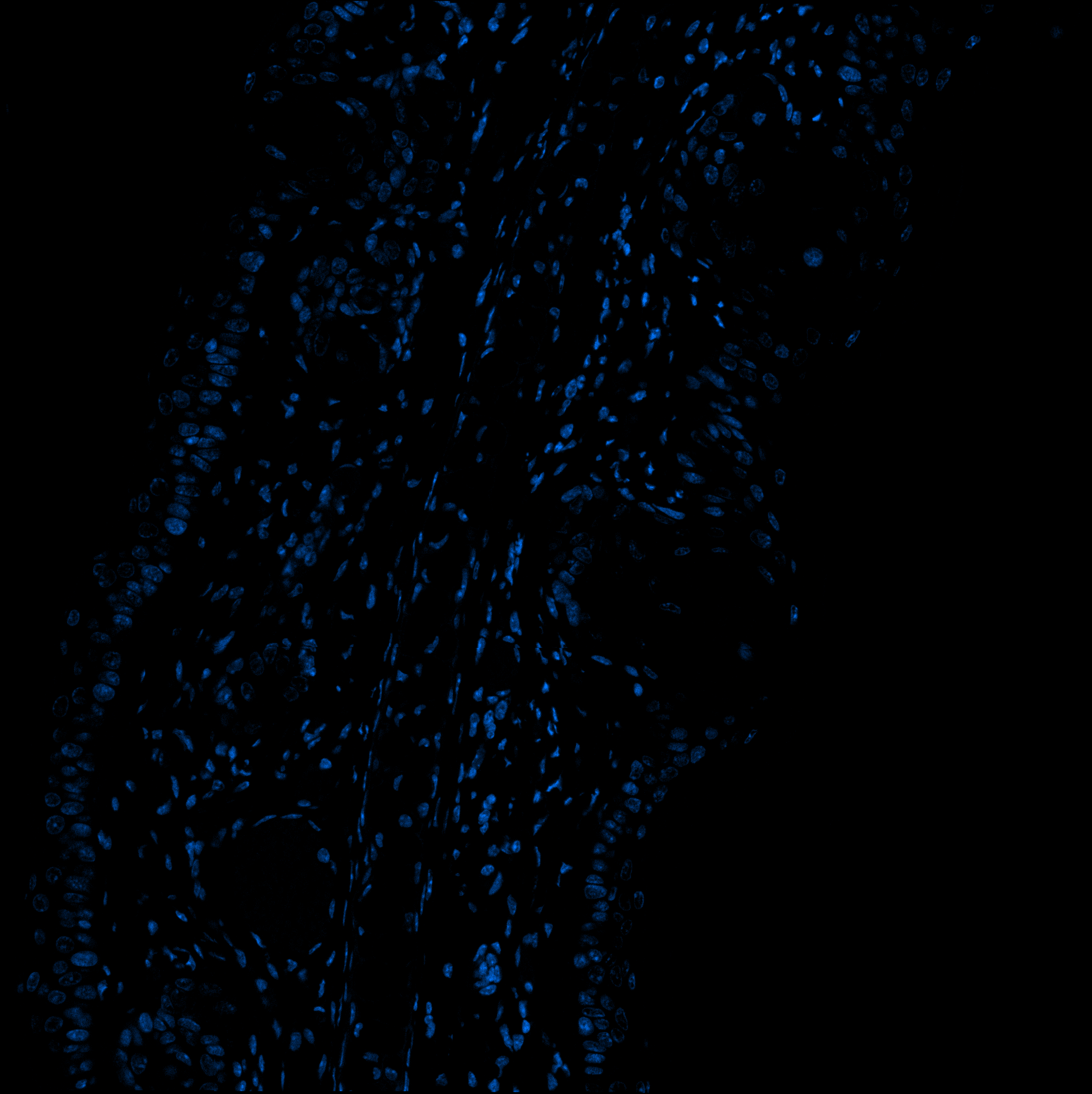

Supplement: Supplementary file 7 — Source data Fig. 4 [file 44319_2024_150_MOESM7_ESM.zip › Main Figure 4/Fig 4G/C3-slide 11 - IMQ - TLR13- 4.tif]

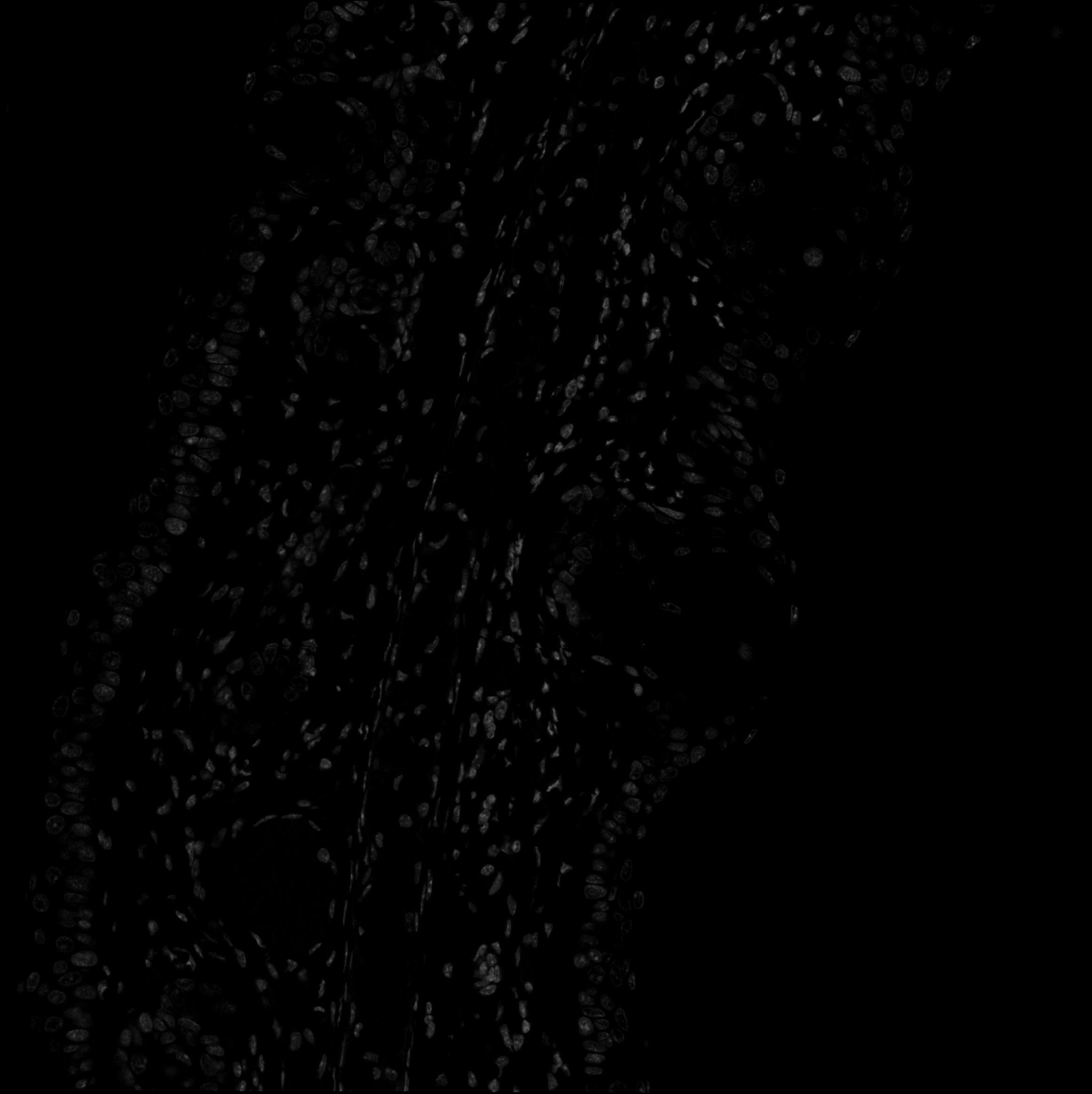

Supplement: Supplementary file 7 — Source data Fig. 4 [file 44319_2024_150_MOESM7_ESM.zip › Main Figure 4/Fig 4G/C3-slide 11 - IMQ - TLR13- 4_sw.tif]

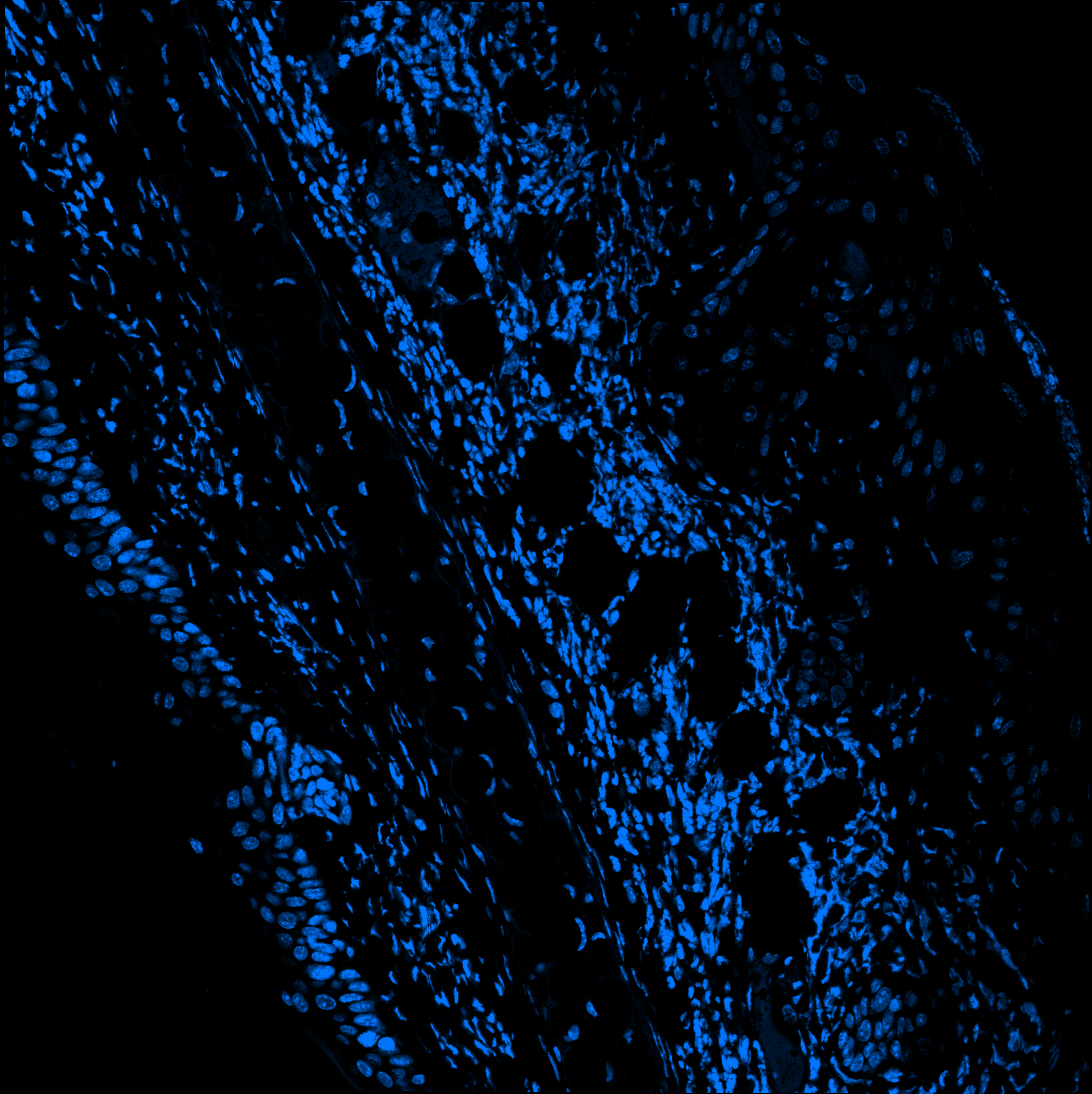

Supplement: Supplementary file 7 — Source data Fig. 4 [file 44319_2024_150_MOESM7_ESM.zip › Main Figure 4/Fig 4G/C3-slide 5 - IMQ - WT -1.tif]

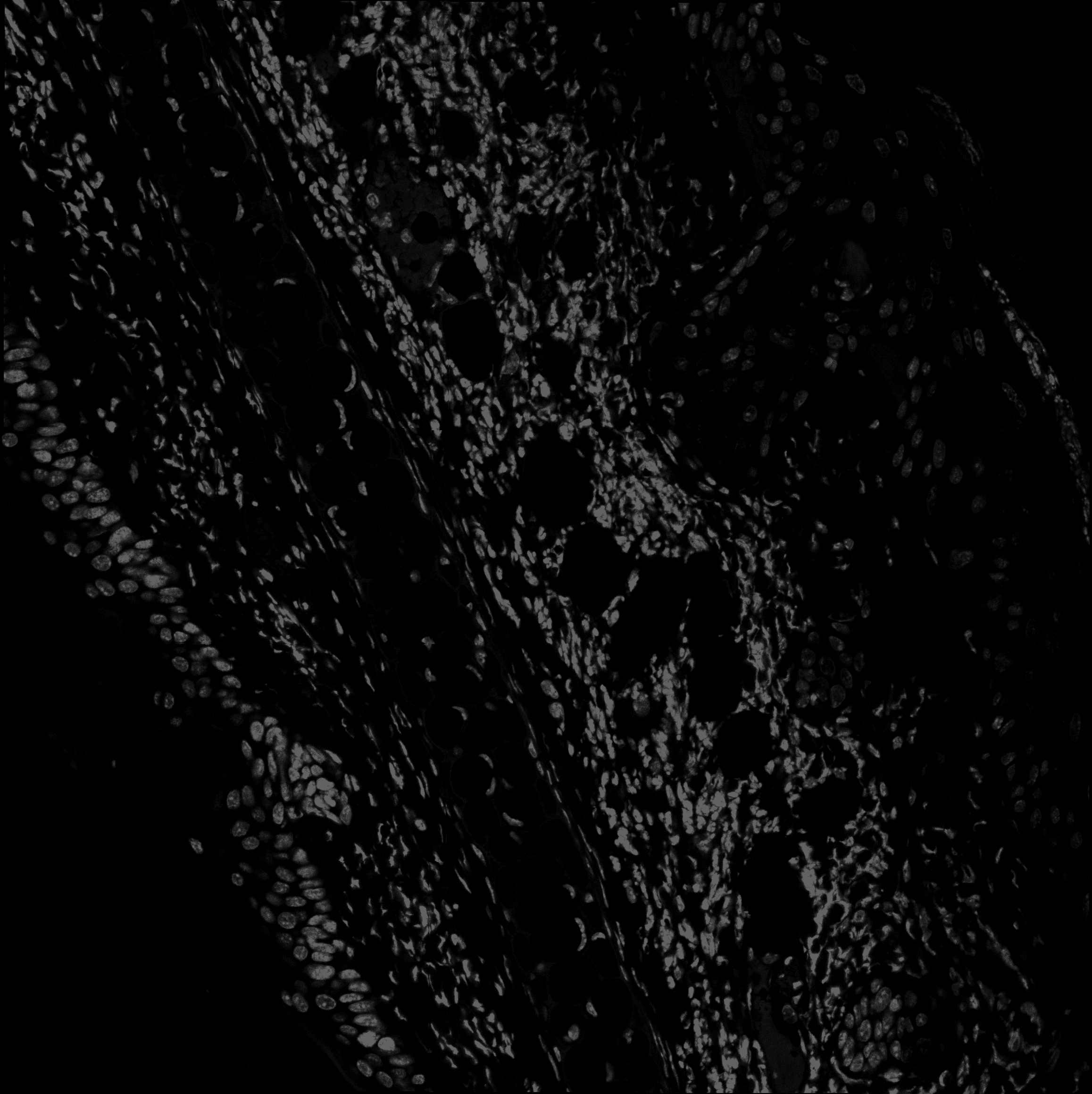

Supplement: Supplementary file 7 — Source data Fig. 4 [file 44319_2024_150_MOESM7_ESM.zip › Main Figure 4/Fig 4G/C3-slide 5 - IMQ - WT -1_sw.tif]

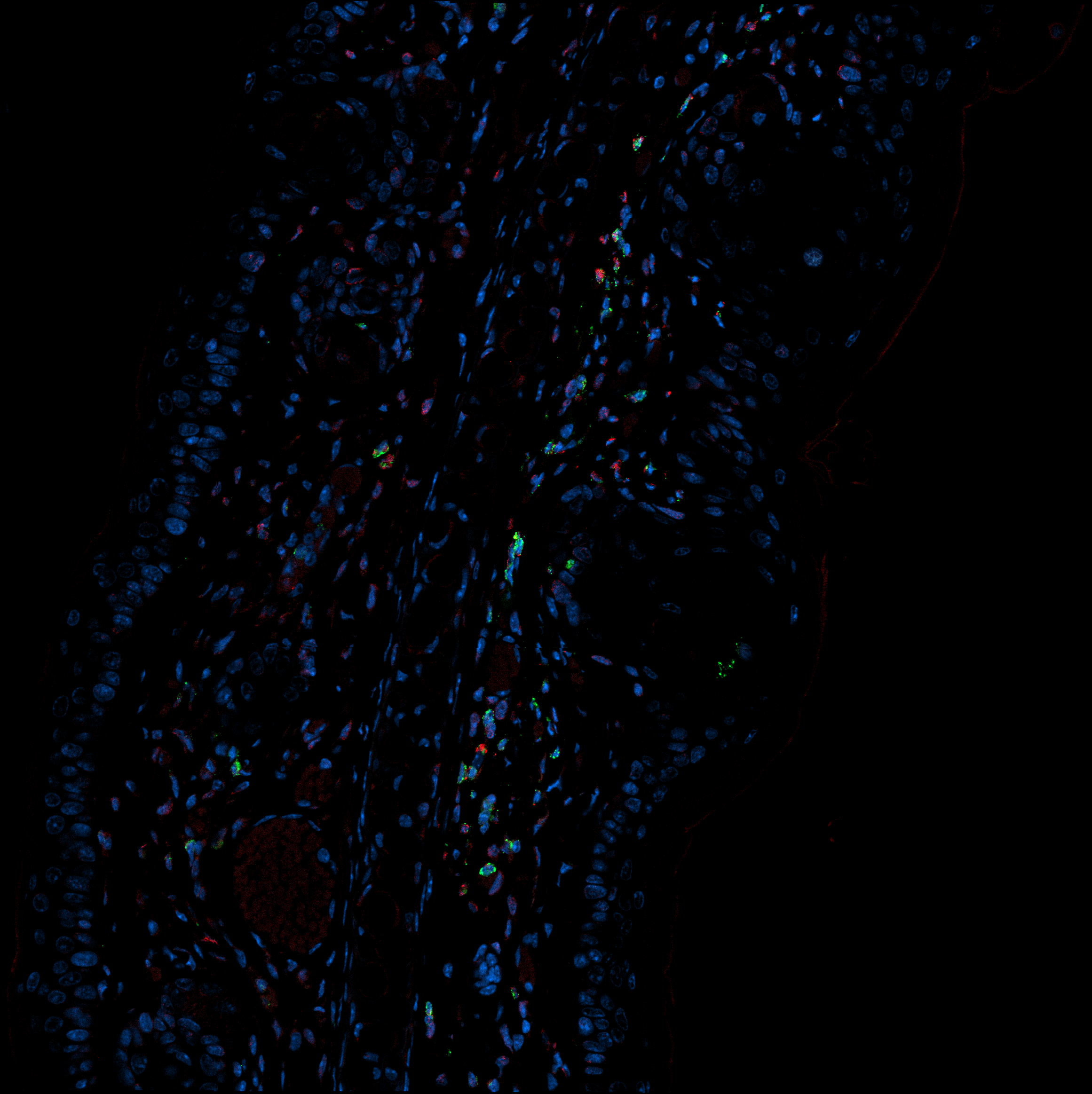

Supplement: Supplementary file 7 — Source data Fig. 4 [file 44319_2024_150_MOESM7_ESM.zip › Main Figure 4/Fig 4G/slide 11 - IMQ - TLR13- 4.czi (RGB).tif]

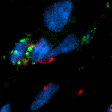

Supplement: Supplementary file 7 — Source data Fig. 4 [file 44319_2024_150_MOESM7_ESM.zip › Main Figure 4/Fig 4G/slide 11 - IMQ - TLR13- 4.czi (RGB)_crop.tif]

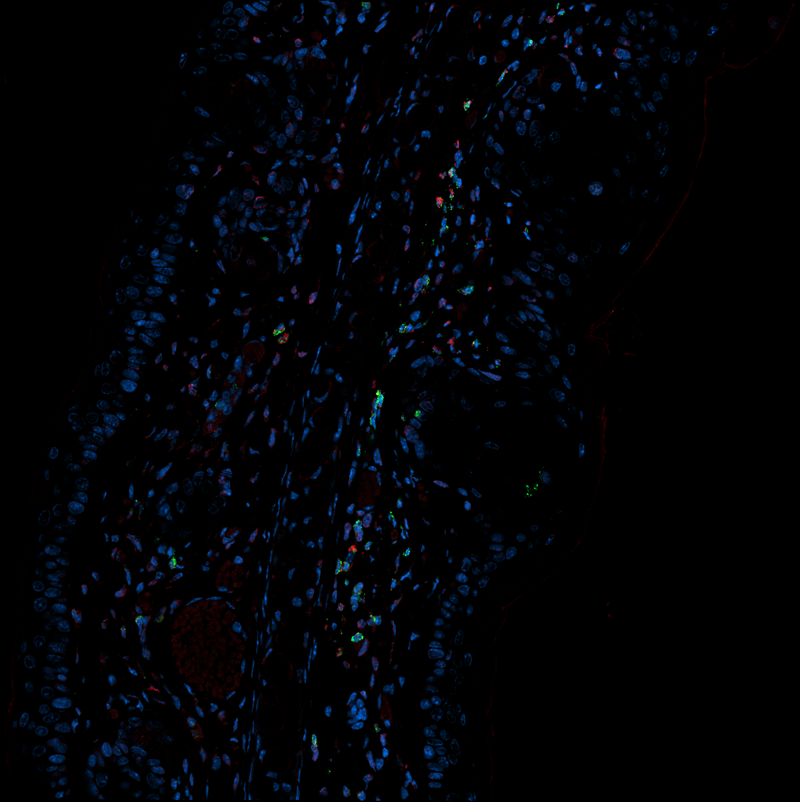

Supplement: Supplementary file 7 — Source data Fig. 4 [file 44319_2024_150_MOESM7_ESM.zip › Main Figure 4/Fig 4G/slide 11 - IMQ - TLR13- 4.czi (RGB)_small.tif]

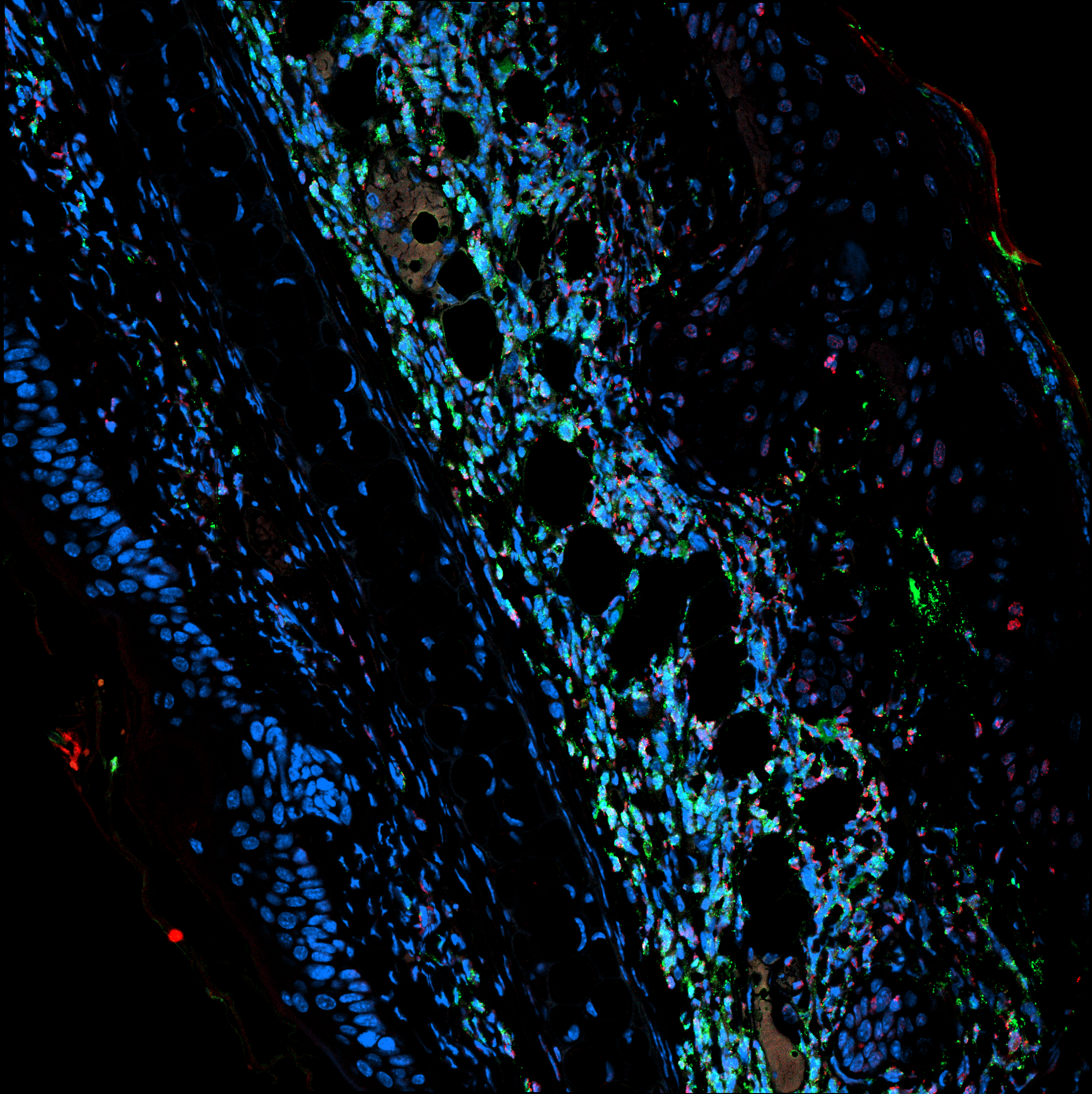

Supplement: Supplementary file 7 — Source data Fig. 4 [file 44319_2024_150_MOESM7_ESM.zip › Main Figure 4/Fig 4G/slide 5 - IMQ - WT -1.czi (RGB).tif]

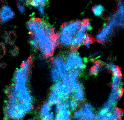

Supplement: Supplementary file 7 — Source data Fig. 4 [file 44319_2024_150_MOESM7_ESM.zip › Main Figure 4/Fig 4G/slide 5 - IMQ - WT -1.czi (RGB)_crop.tif]

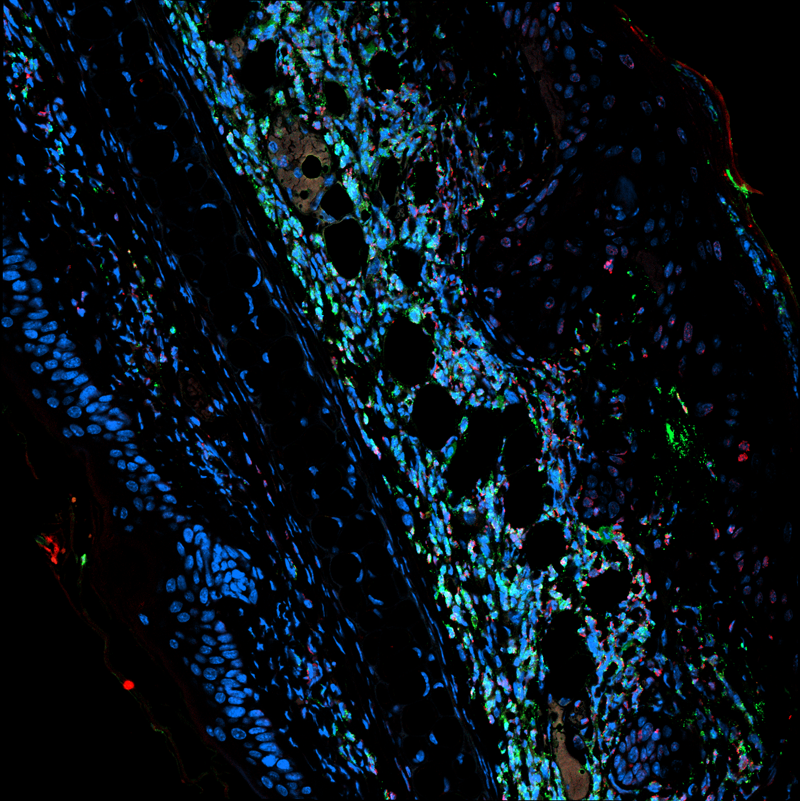

Supplement: Supplementary file 7 — Source data Fig. 4 [file 44319_2024_150_MOESM7_ESM.zip › Main Figure 4/Fig 4G/slide 5 - IMQ - WT -1.czi (RGB)_small.tif]

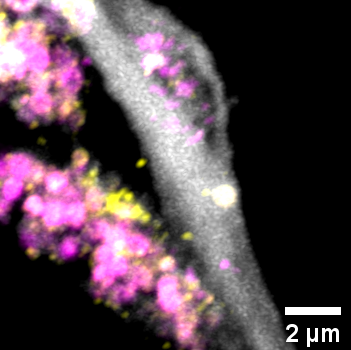

Supplement: Supplementary file 8 — Source data Fig. 5 [file 44319_2024_150_MOESM8_ESM.zip › Main Figure 5/Fig 5A/PMA/pma 2 crop.png]

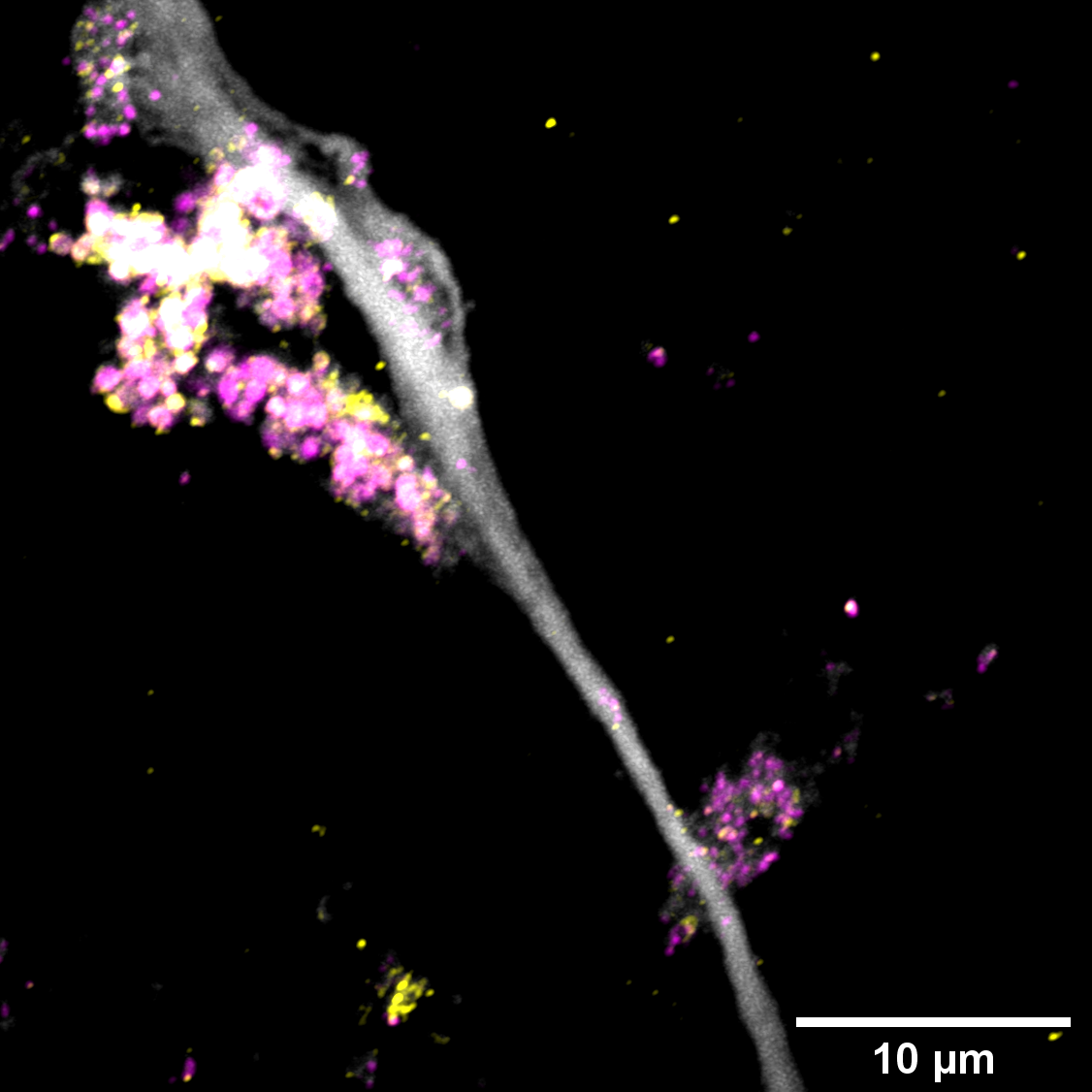

Supplement: Supplementary file 8 — Source data Fig. 5 [file 44319_2024_150_MOESM8_ESM.zip › Main Figure 5/Fig 5A/PMA/pma2.png]

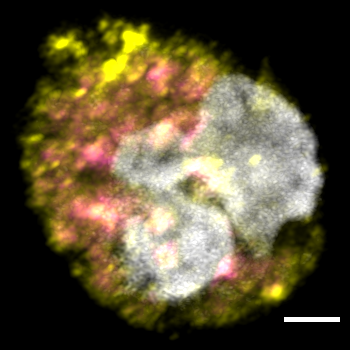

Supplement: Supplementary file 8 — Source data Fig. 5 [file 44319_2024_150_MOESM8_ESM.zip › Main Figure 5/Fig 5A/Unstim/comp 1 crop.png]

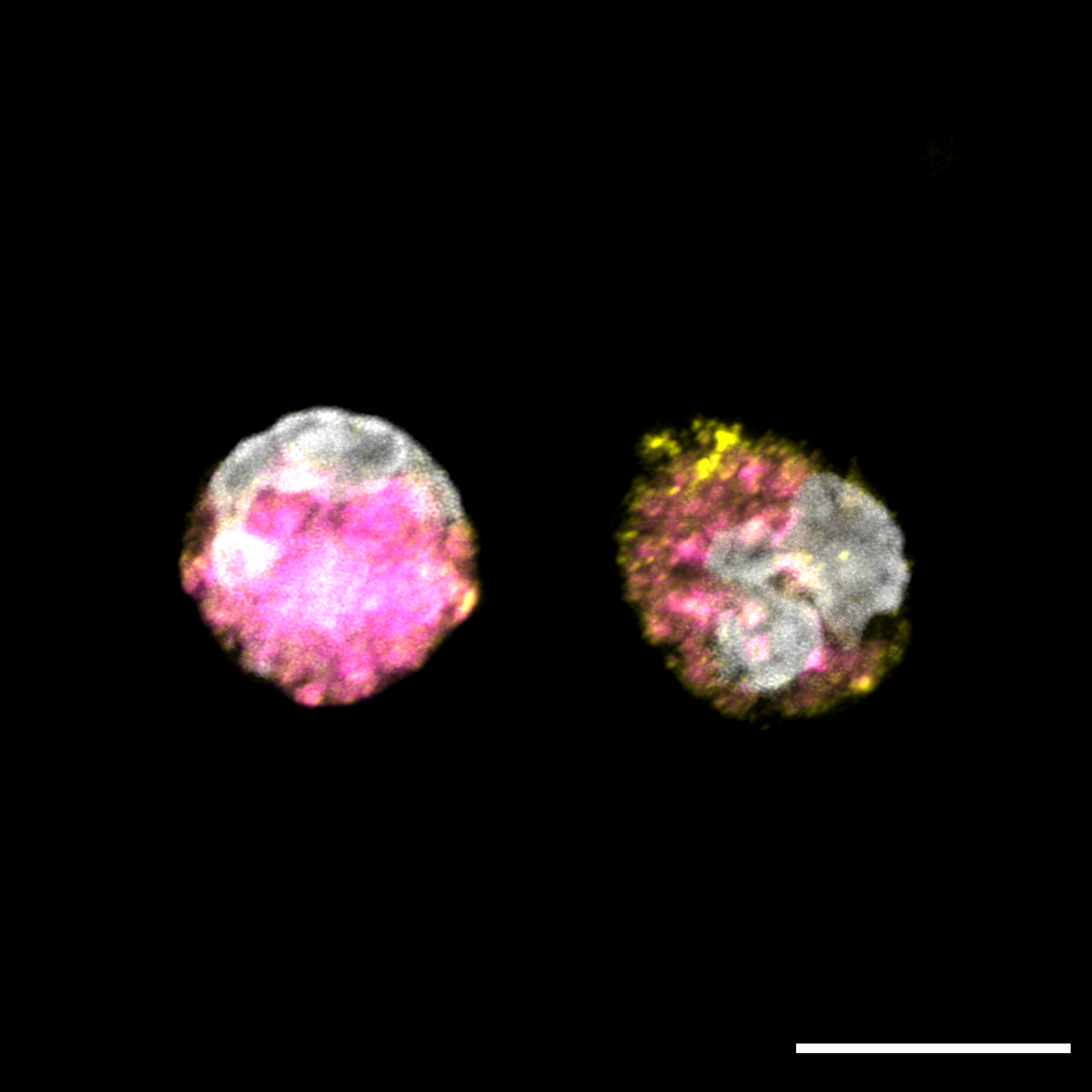

Supplement: Supplementary file 8 — Source data Fig. 5 [file 44319_2024_150_MOESM8_ESM.zip › Main Figure 5/Fig 5A/Unstim/comp 1.png]

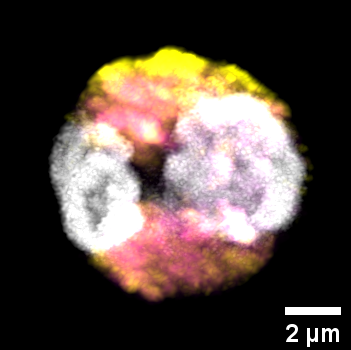

Supplement: Supplementary file 8 — Source data Fig. 5 [file 44319_2024_150_MOESM8_ESM.zip › Main Figure 5/Fig 5A/Unstim/comp2 crop.png]

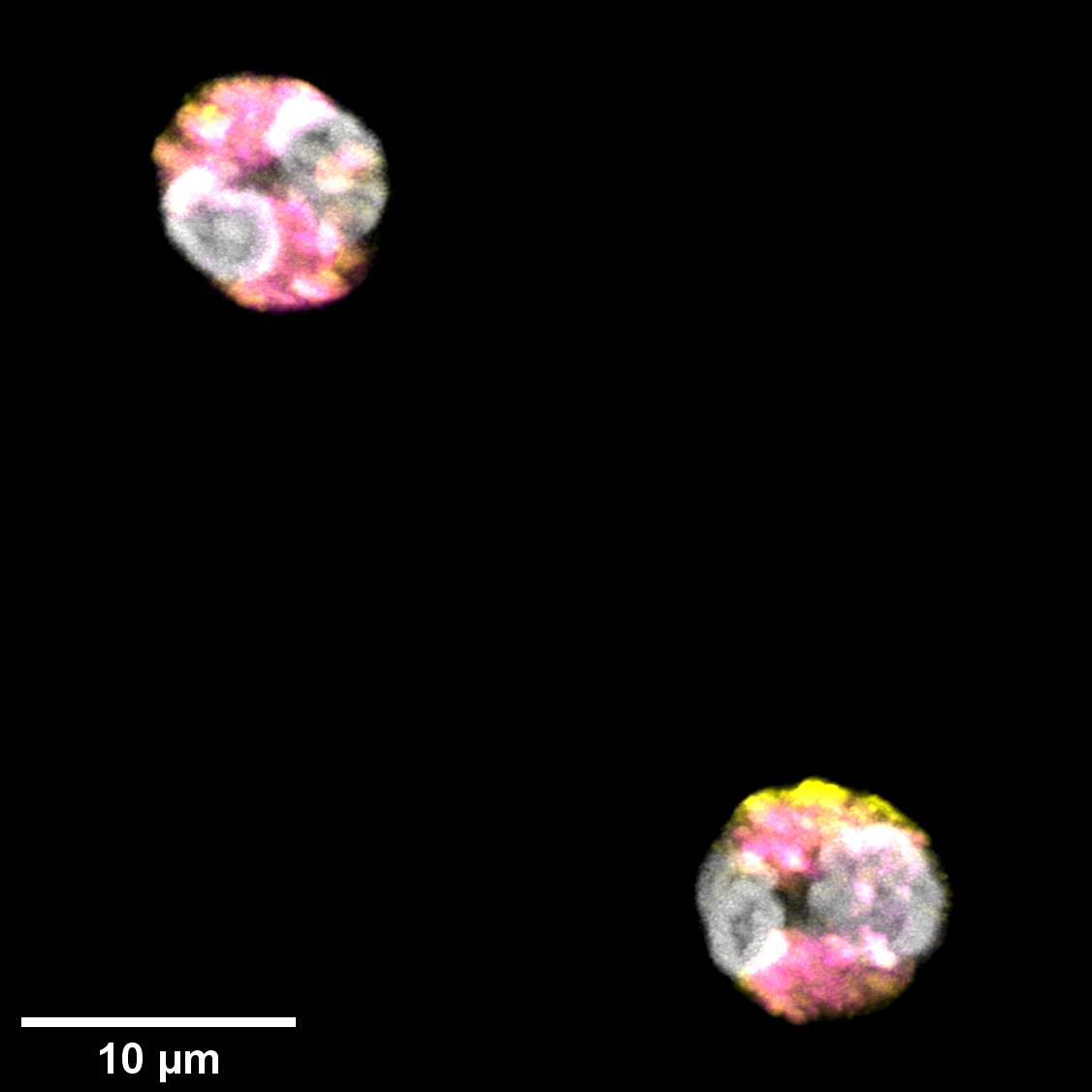

Supplement: Supplementary file 8 — Source data Fig. 5 [file 44319_2024_150_MOESM8_ESM.zip › Main Figure 5/Fig 5A/Unstim/comp2.png]

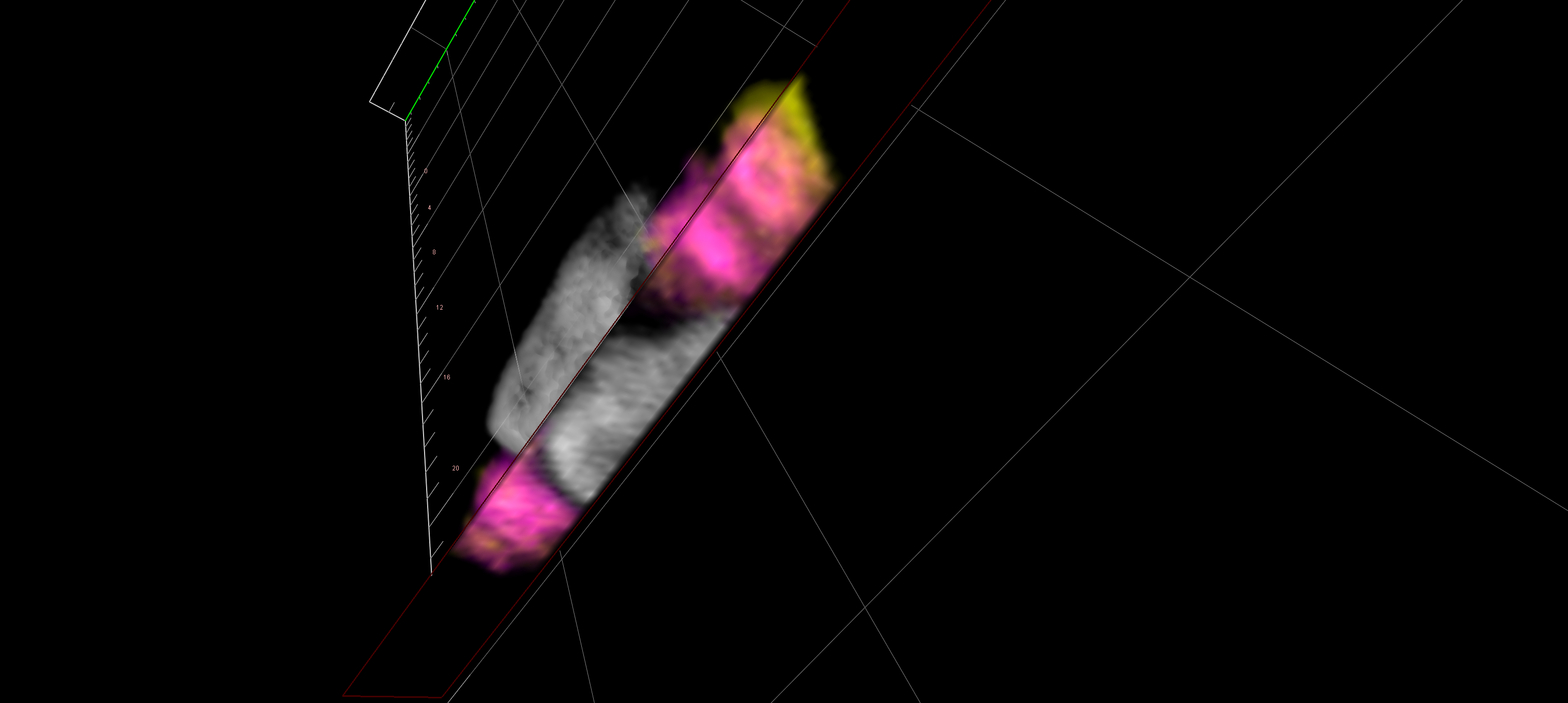

Supplement: Supplementary file 8 — Source data Fig. 5 [file 44319_2024_150_MOESM8_ESM.zip › Main Figure 5/Fig 5A/Unstim/Experiment-60-Airyscan Processing-02 (Snapshot 1).png]

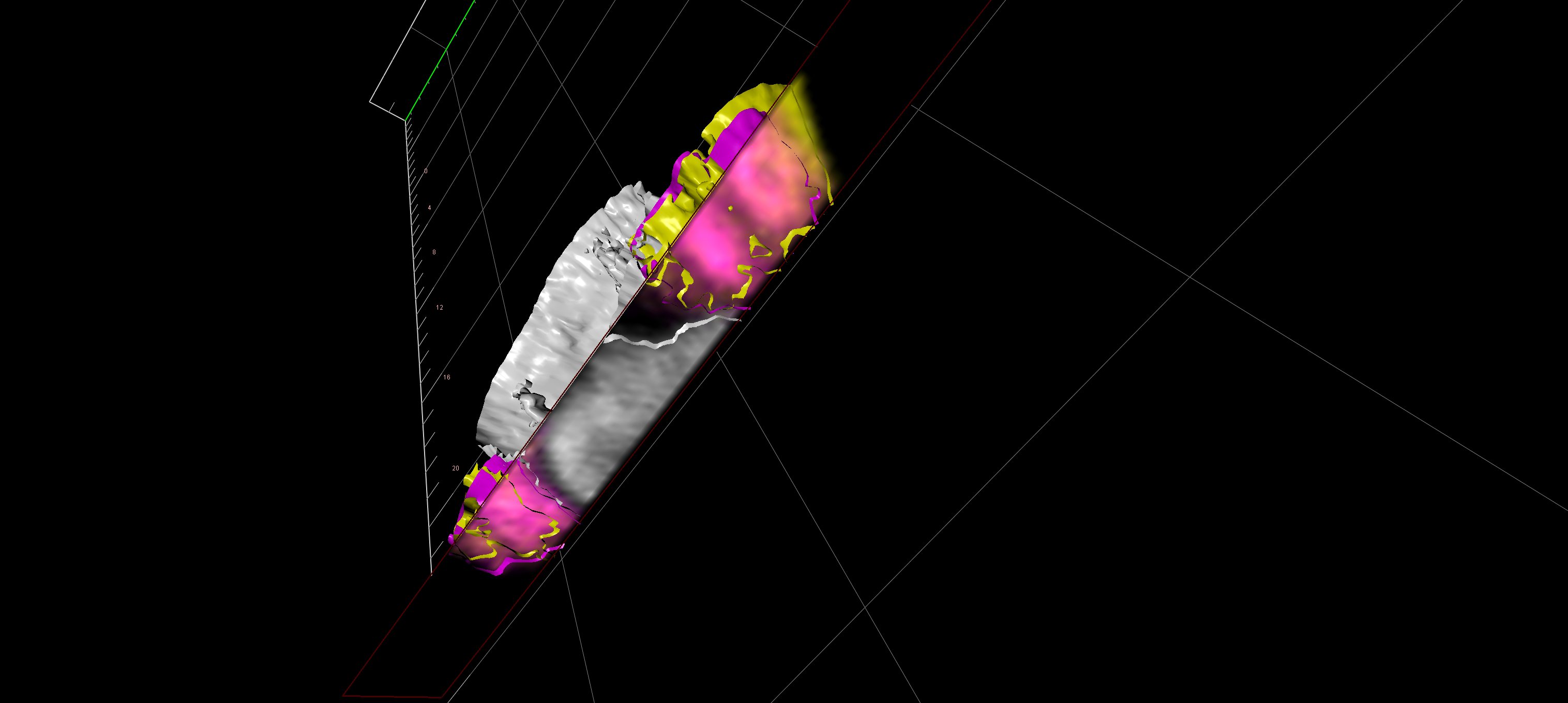

Supplement: Supplementary file 8 — Source data Fig. 5 [file 44319_2024_150_MOESM8_ESM.zip › Main Figure 5/Fig 5A/Unstim/Experiment-60-Airyscan Processing-02 (Snapshot 2).png]

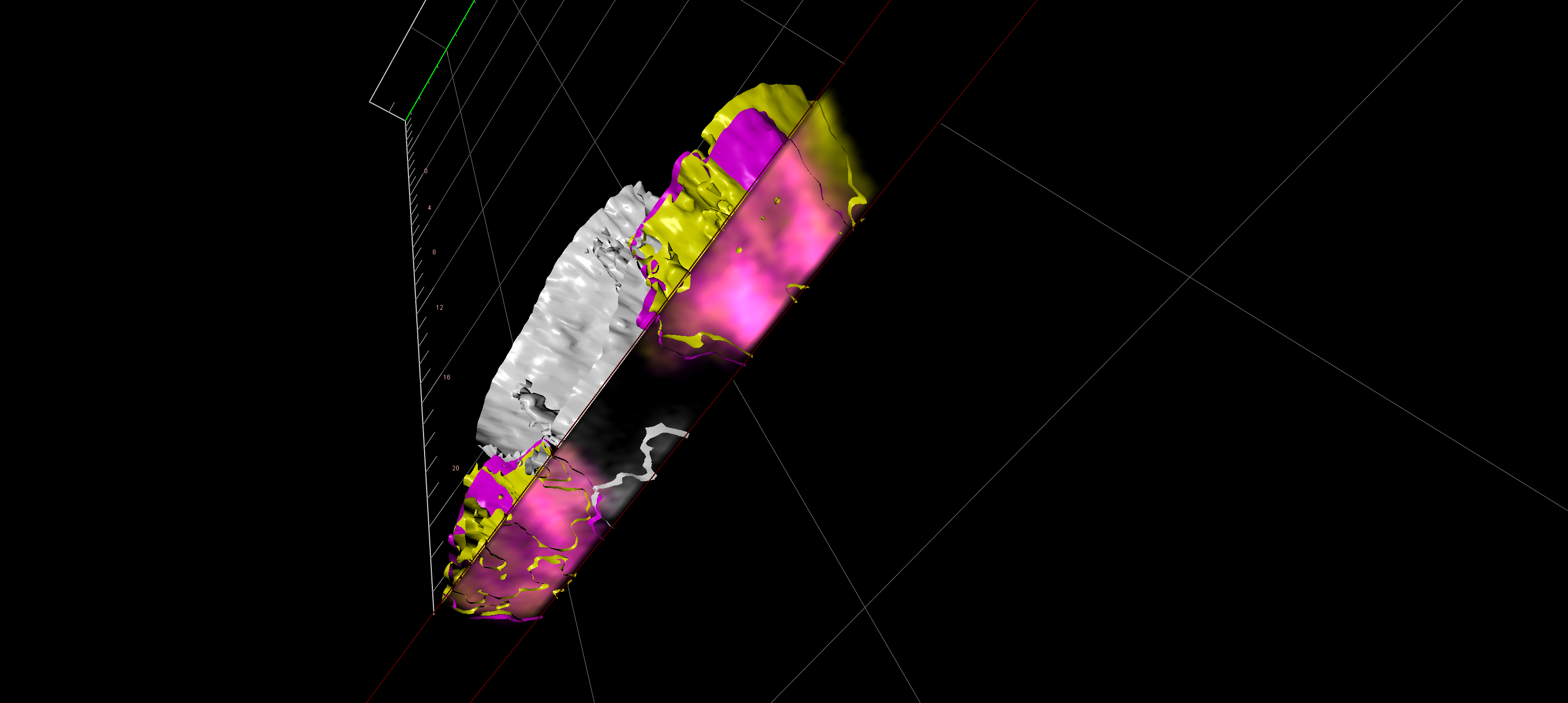

Supplement: Supplementary file 8 — Source data Fig. 5 [file 44319_2024_150_MOESM8_ESM.zip › Main Figure 5/Fig 5A/Unstim/Experiment-60-Airyscan Processing-02 (Snapshot 3).png]

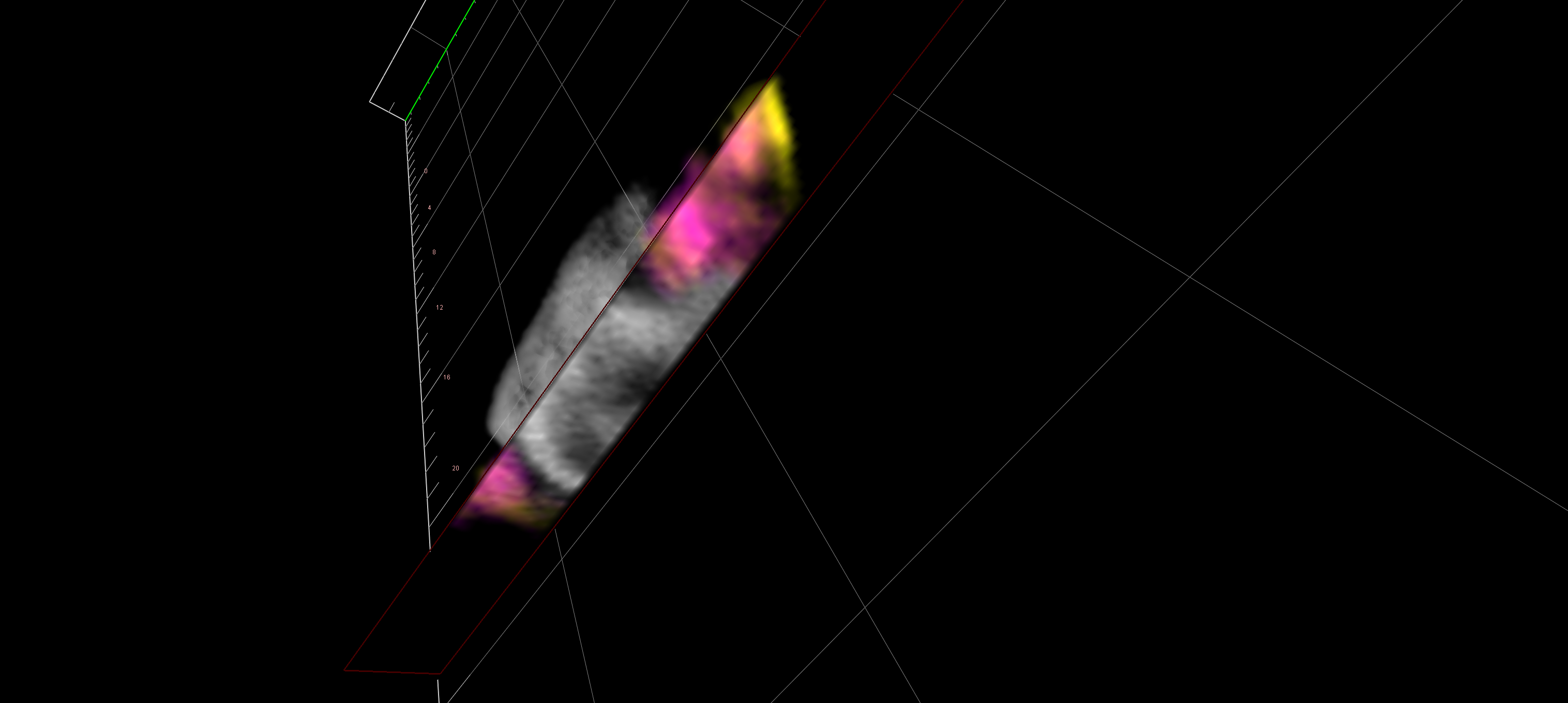

Supplement: Supplementary file 8 — Source data Fig. 5 [file 44319_2024_150_MOESM8_ESM.zip › Main Figure 5/Fig 5A/Unstim/Experiment-60-Airyscan Processing-02 (Snapshot).png]

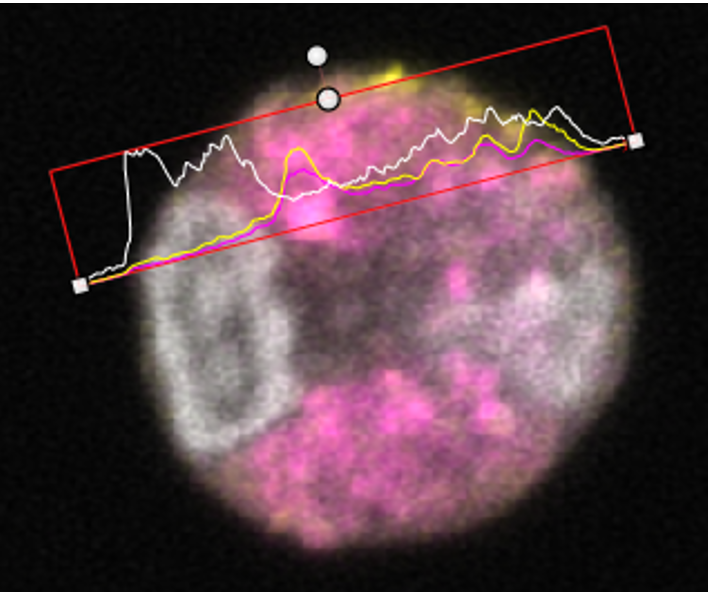

Supplement: Supplementary file 8 — Source data Fig. 5 [file 44319_2024_150_MOESM8_ESM.zip › Main Figure 5/Fig 5C/unstim 1 cropped.png]

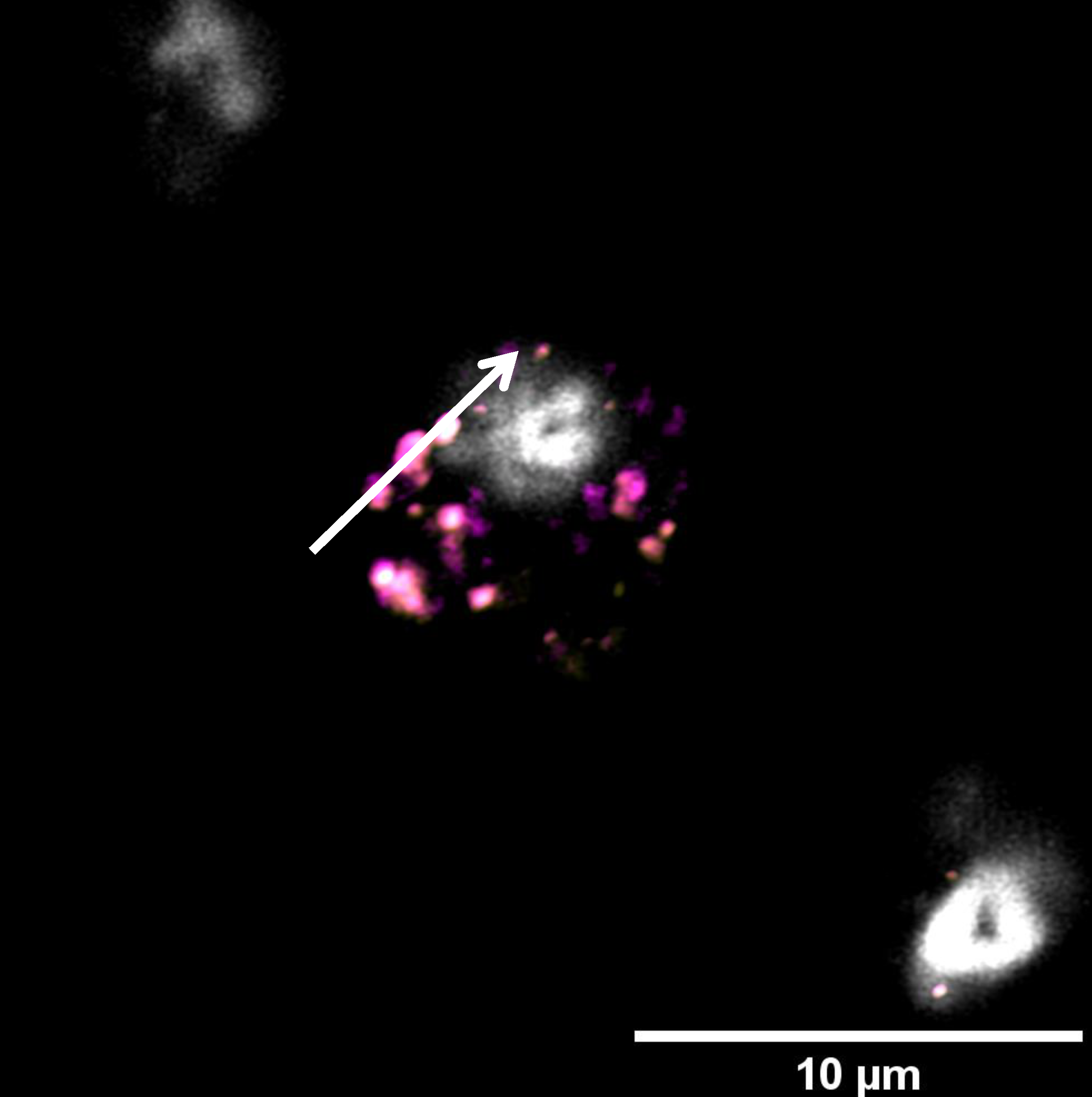

Supplement: Supplementary file 8 — Source data Fig. 5 [file 44319_2024_150_MOESM8_ESM.zip › Main Figure 5/Fig 5D/Fig 5D.tif]

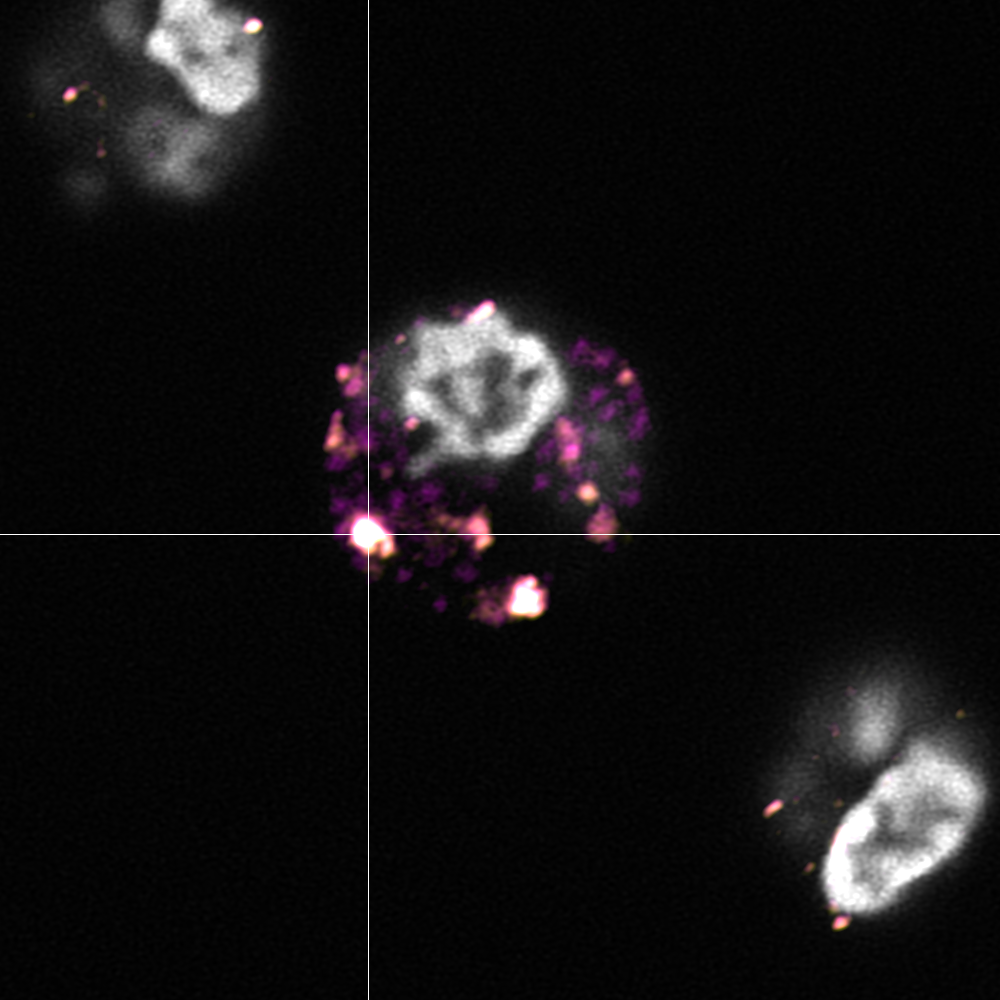

Supplement: Supplementary file 8 — Source data Fig. 5 [file 44319_2024_150_MOESM8_ESM.zip › Main Figure 5/Fig 5E/Experiment-568-Airyscan Processing-36.png]

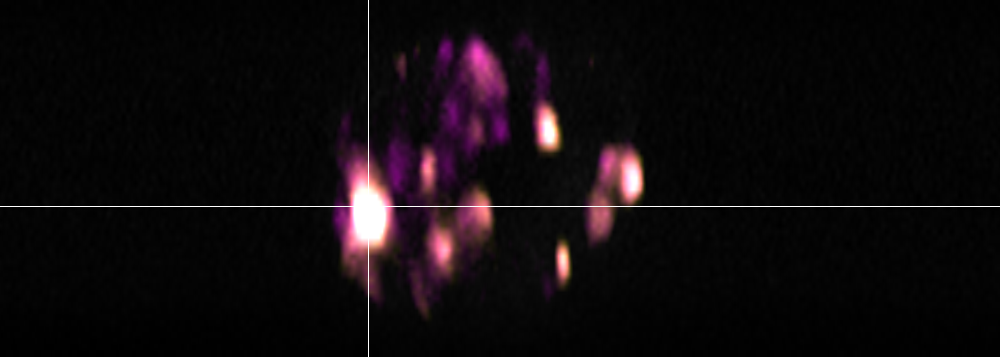

Supplement: Supplementary file 8 — Source data Fig. 5 [file 44319_2024_150_MOESM8_ESM.zip › Main Figure 5/Fig 5E/XZ 534.png]

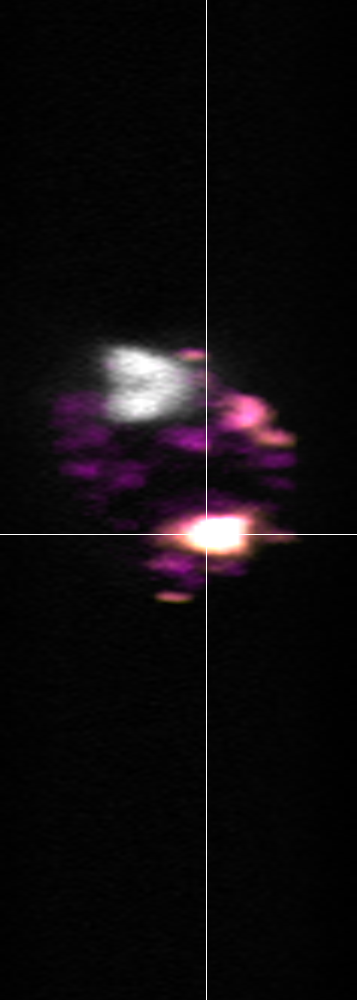

Supplement: Supplementary file 8 — Source data Fig. 5 [file 44319_2024_150_MOESM8_ESM.zip › Main Figure 5/Fig 5E/YZ 368.png]
