# Supplementary figures and images for: naRNA-LL37 composite DAMPs define sterile NETs as self-propagating drivers of inflammation (part 3 of 4)
Source: EMBO Rep. 2024 May 23;25(7):10. doi: 10.1038/s44319-024-00150-5 (PMC11239898; doi:10.1038/s44319-024-00150-5)

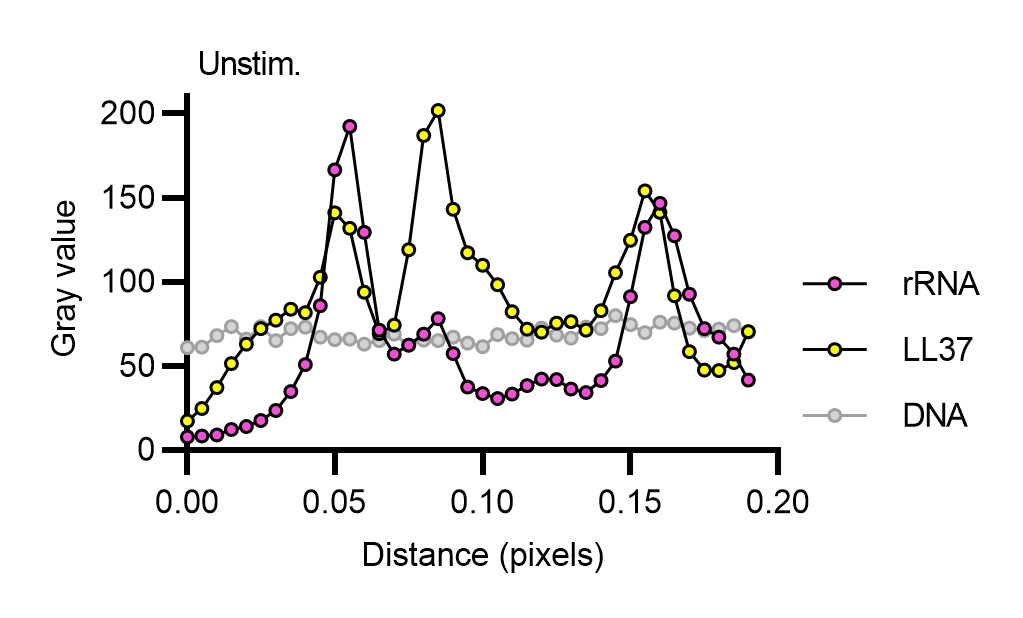

Supplement: Supplementary file 8 — Source data Fig. 5 [file 44319_2024_150_MOESM8_ESM.zip › Main Figure 5/Fig 5F/5F.png]

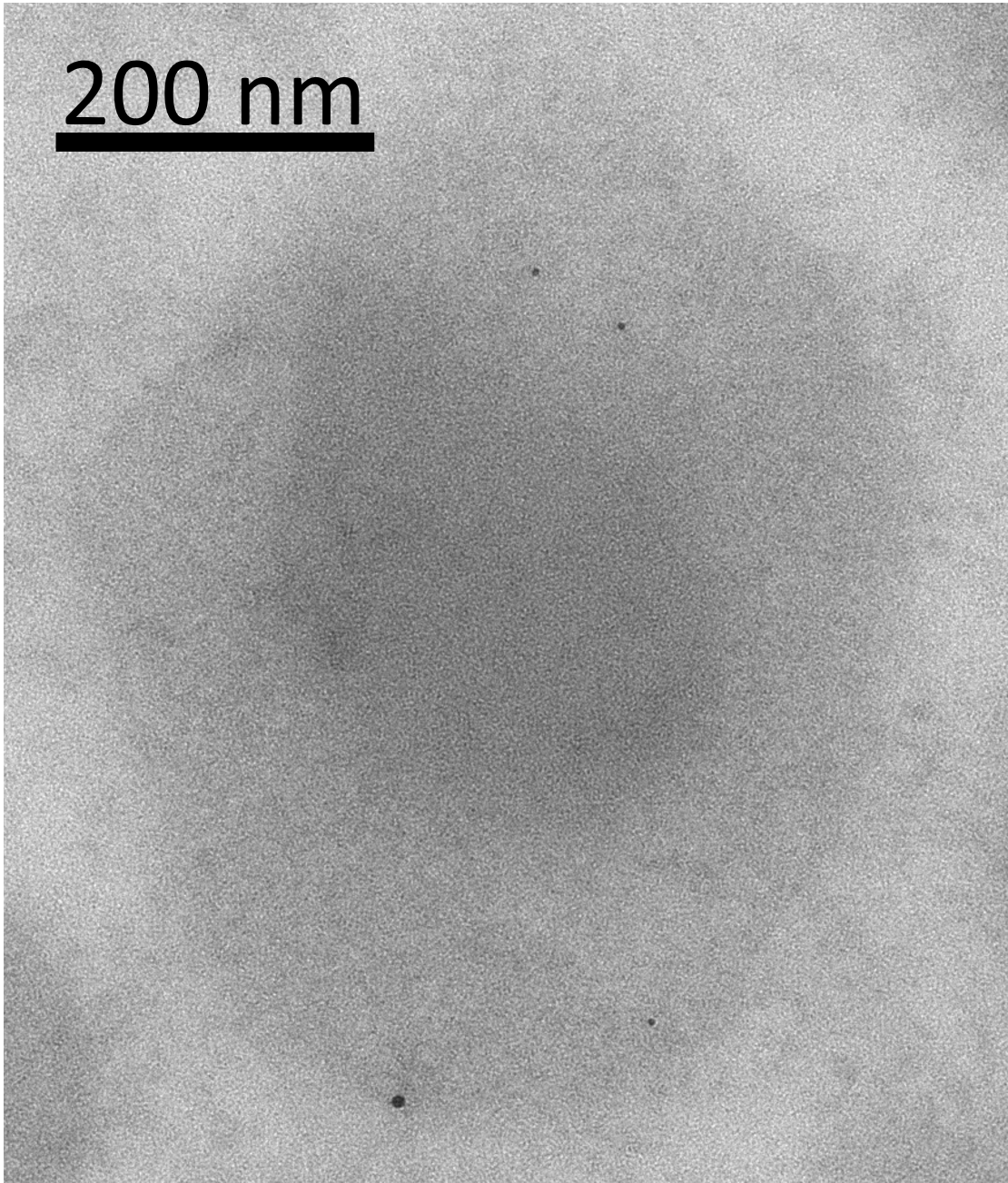

Supplement: Supplementary file 8 — Source data Fig. 5 [file 44319_2024_150_MOESM8_ESM.zip › Main Figure 5/Fig 5G/unstim 1 high zoom.png]

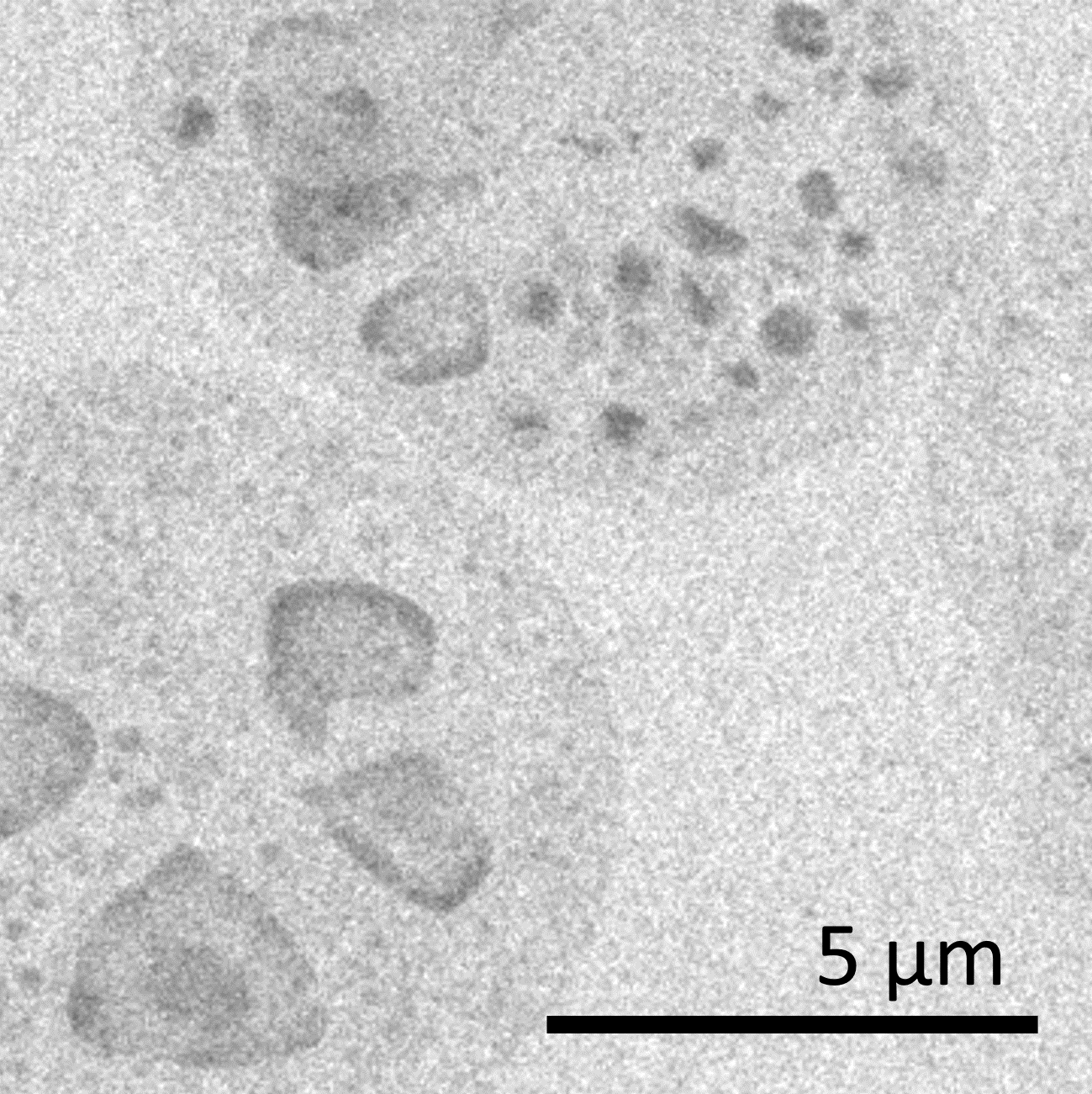

Supplement: Supplementary file 8 — Source data Fig. 5 [file 44319_2024_150_MOESM8_ESM.zip › Main Figure 5/Fig 5G/unstim 1.png]

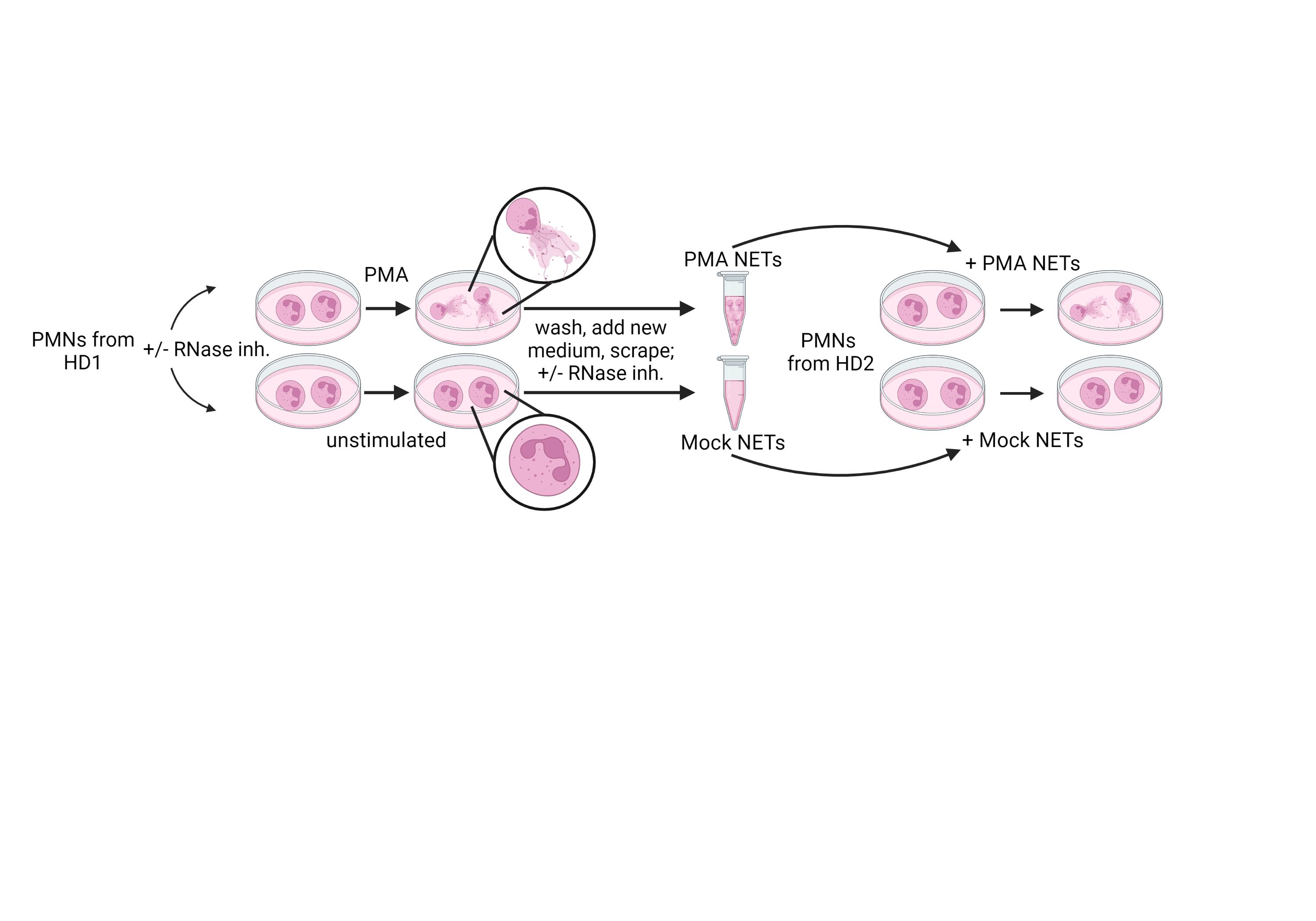

Supplement: Supplementary file 9 — EV Figures Source Data [file 44319_2024_150_MOESM9_ESM.zip › Figure EV2/Fig S2B/workflow creation NET content new (2).png]

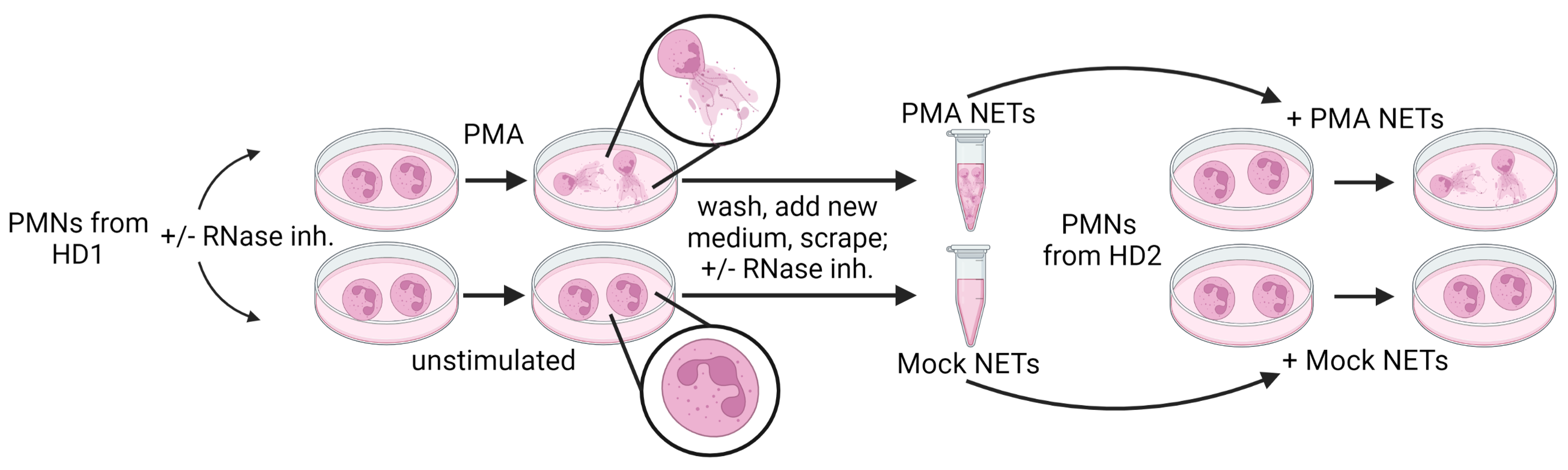

Supplement: Supplementary file 9 — EV Figures Source Data [file 44319_2024_150_MOESM9_ESM.zip › Figure EV2/Fig S2B/workflow creation NET content new cropped.png]

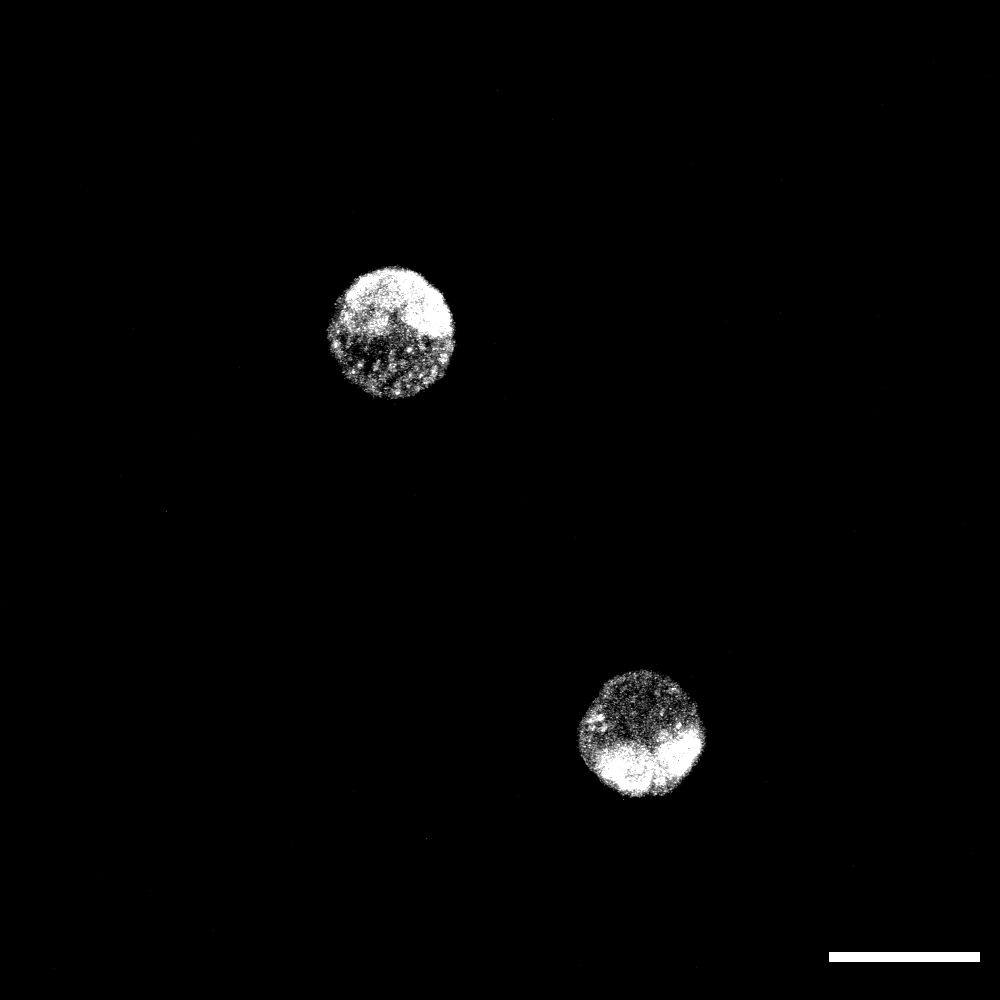

Supplement: Supplementary file 9 — EV Figures Source Data [file 44319_2024_150_MOESM9_ESM.zip › Figure EV2/Fig S2C/image data FB-118/mock+rnase inh. 1_50/comp new grey.png]

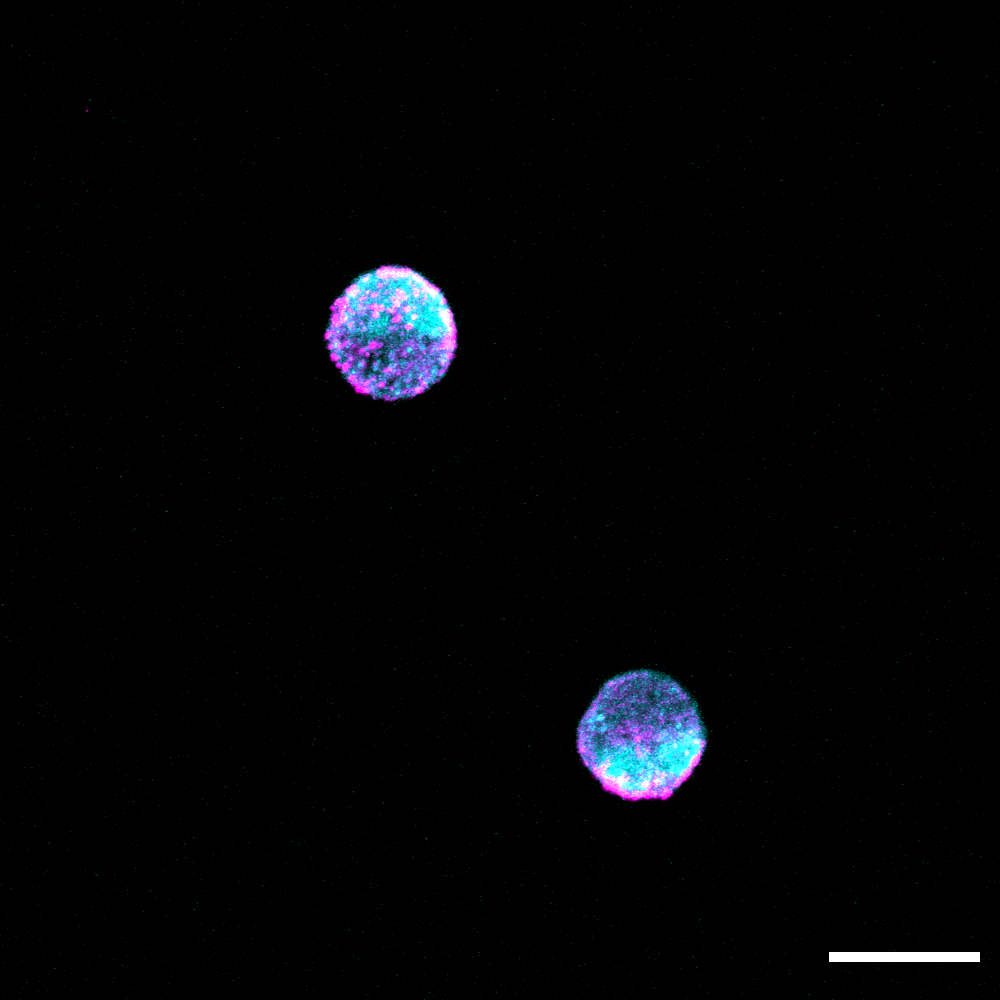

Supplement: Supplementary file 9 — EV Figures Source Data [file 44319_2024_150_MOESM9_ESM.zip › Figure EV2/Fig S2C/image data FB-118/mock+rnase inh. 1_50/comp new.png]

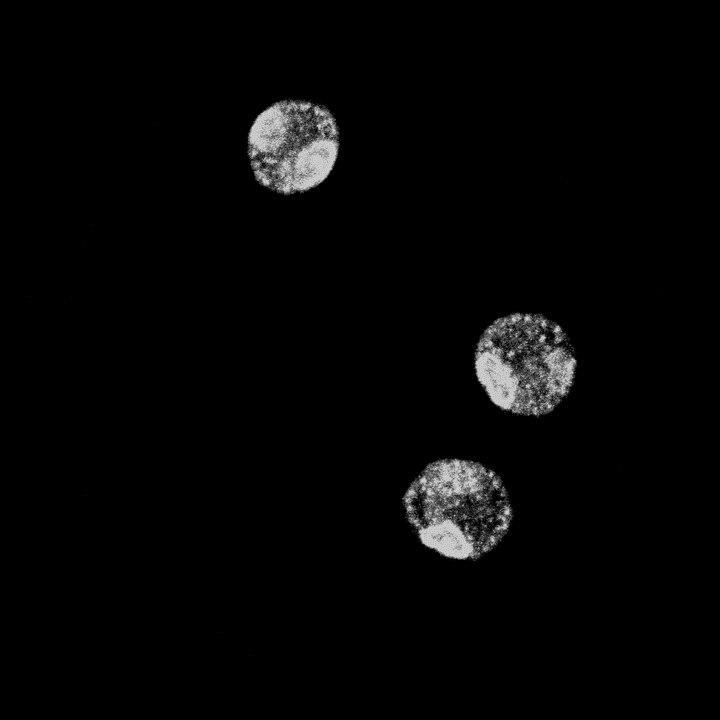

Supplement: Supplementary file 9 — EV Figures Source Data [file 44319_2024_150_MOESM9_ESM.zip › Figure EV2/Fig S2C/image data FB-118/mock+rnase inh. 1_500/Bild1.png]

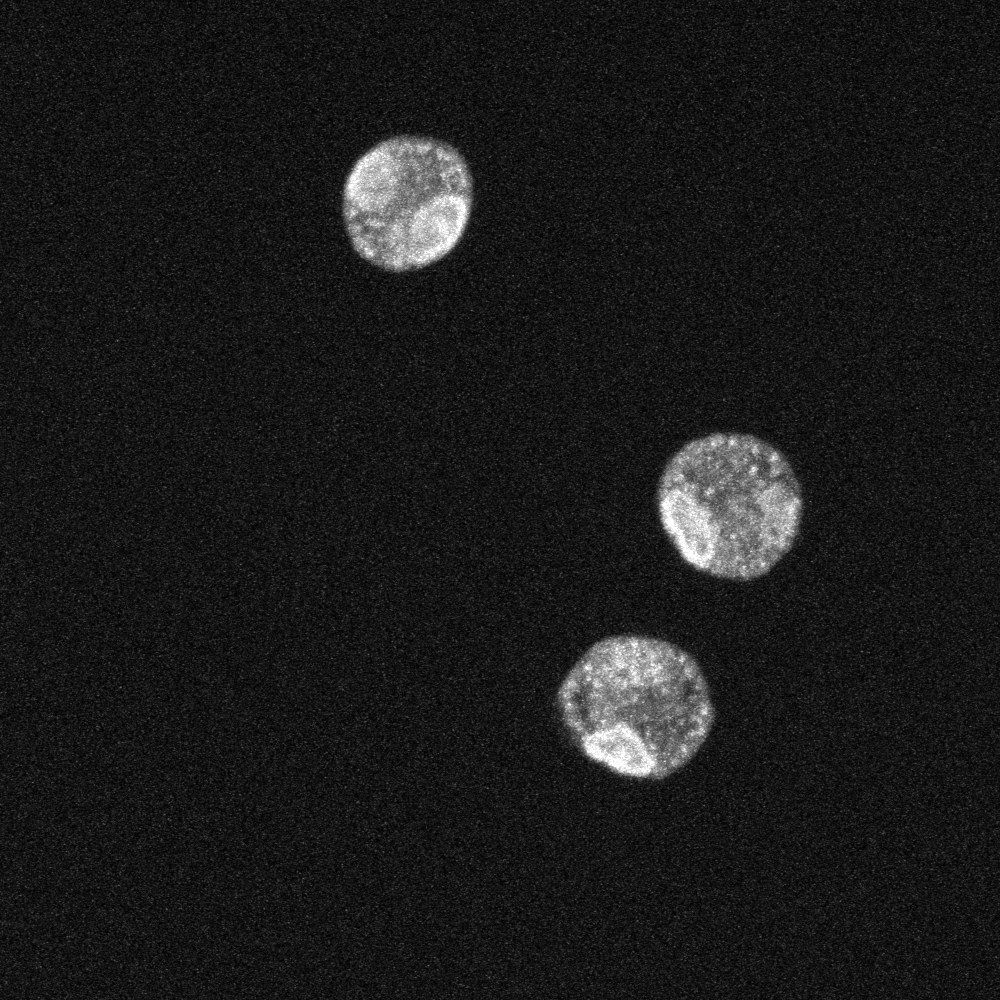

Supplement: Supplementary file 9 — EV Figures Source Data [file 44319_2024_150_MOESM9_ESM.zip › Figure EV2/Fig S2C/image data FB-118/mock+rnase inh. 1_500/comp new grey.png]

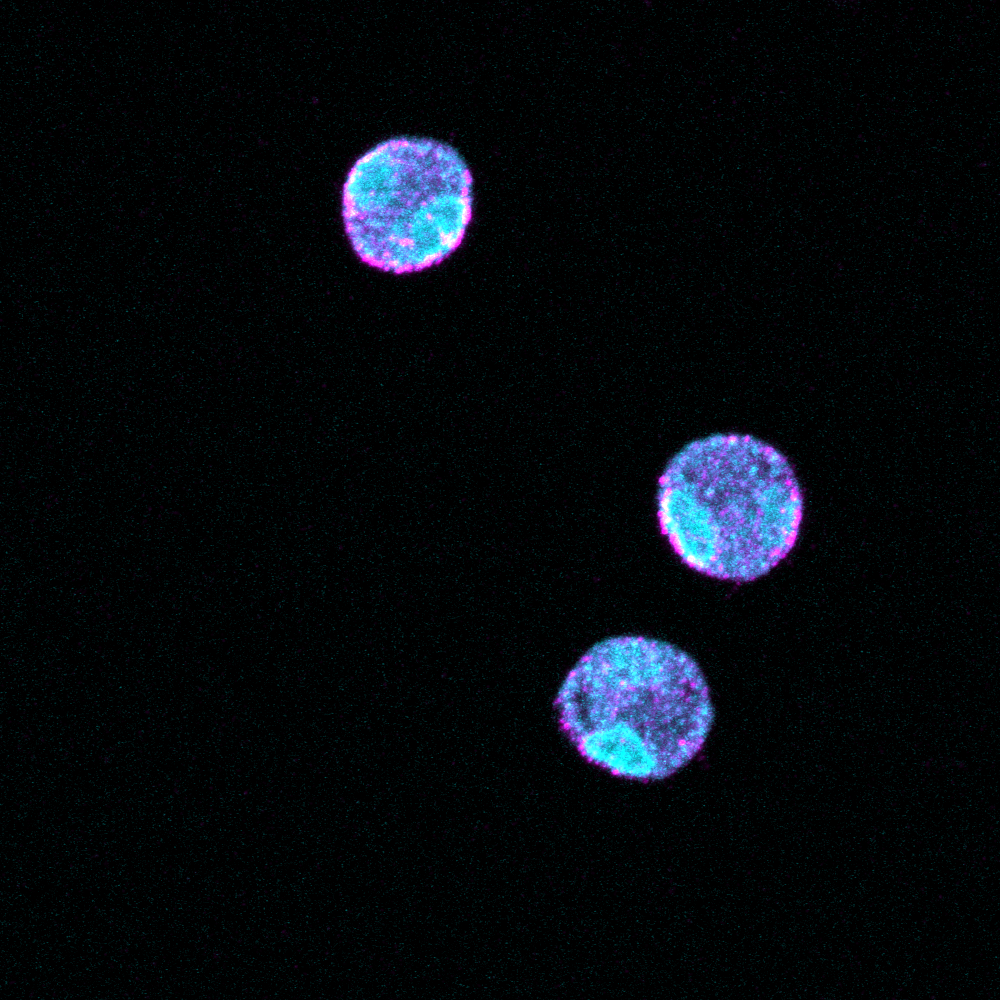

Supplement: Supplementary file 9 — EV Figures Source Data [file 44319_2024_150_MOESM9_ESM.zip › Figure EV2/Fig S2C/image data FB-118/mock+rnase inh. 1_500/comp new.png]

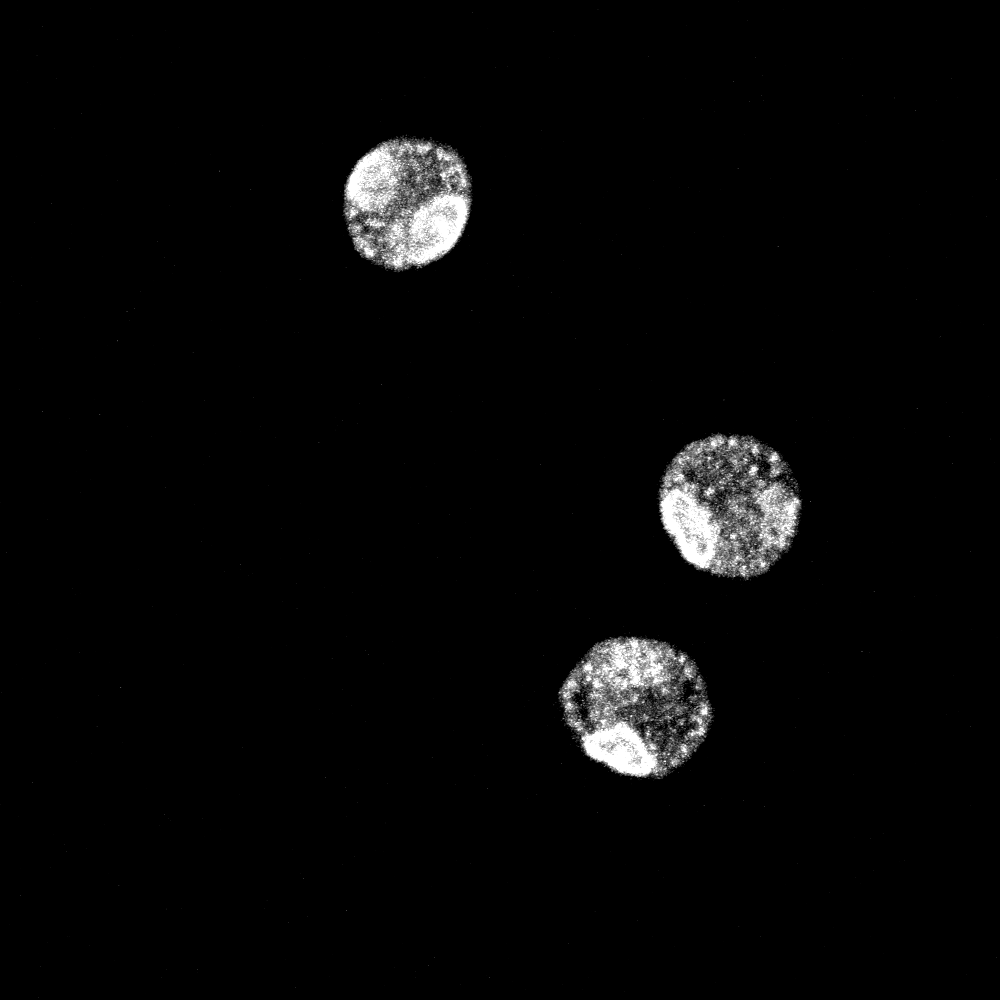

Supplement: Supplementary file 9 — EV Figures Source Data [file 44319_2024_150_MOESM9_ESM.zip › Figure EV2/Fig S2C/image data FB-118/mock+rnase inh. 1_500/scale.png]

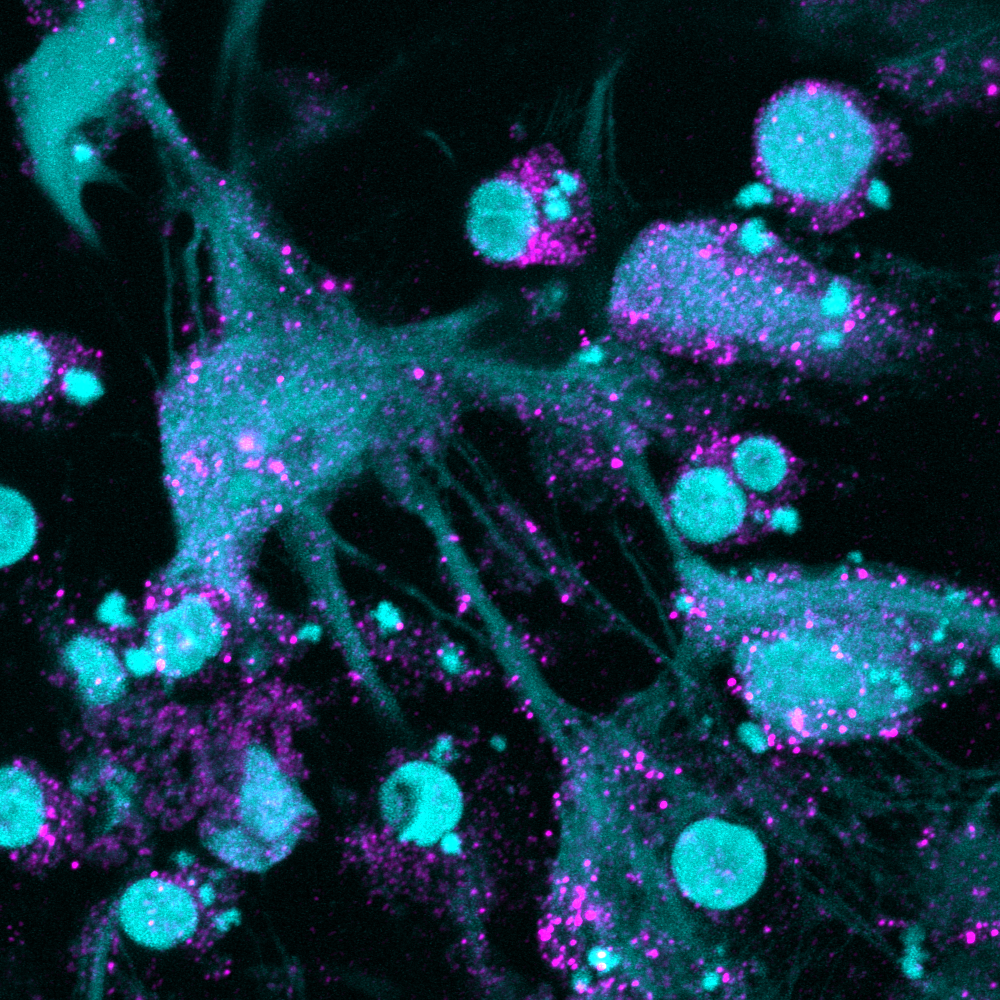

Supplement: Supplementary file 9 — EV Figures Source Data [file 44319_2024_150_MOESM9_ESM.zip › Figure EV2/Fig S2C/image data FB-118/PMA/comp new.png]

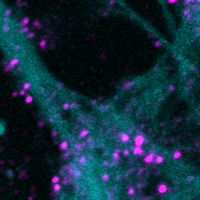

Supplement: Supplementary file 9 — EV Figures Source Data [file 44319_2024_150_MOESM9_ESM.zip › Figure EV2/Fig S2C/image data FB-118/PMA/crop new.png]

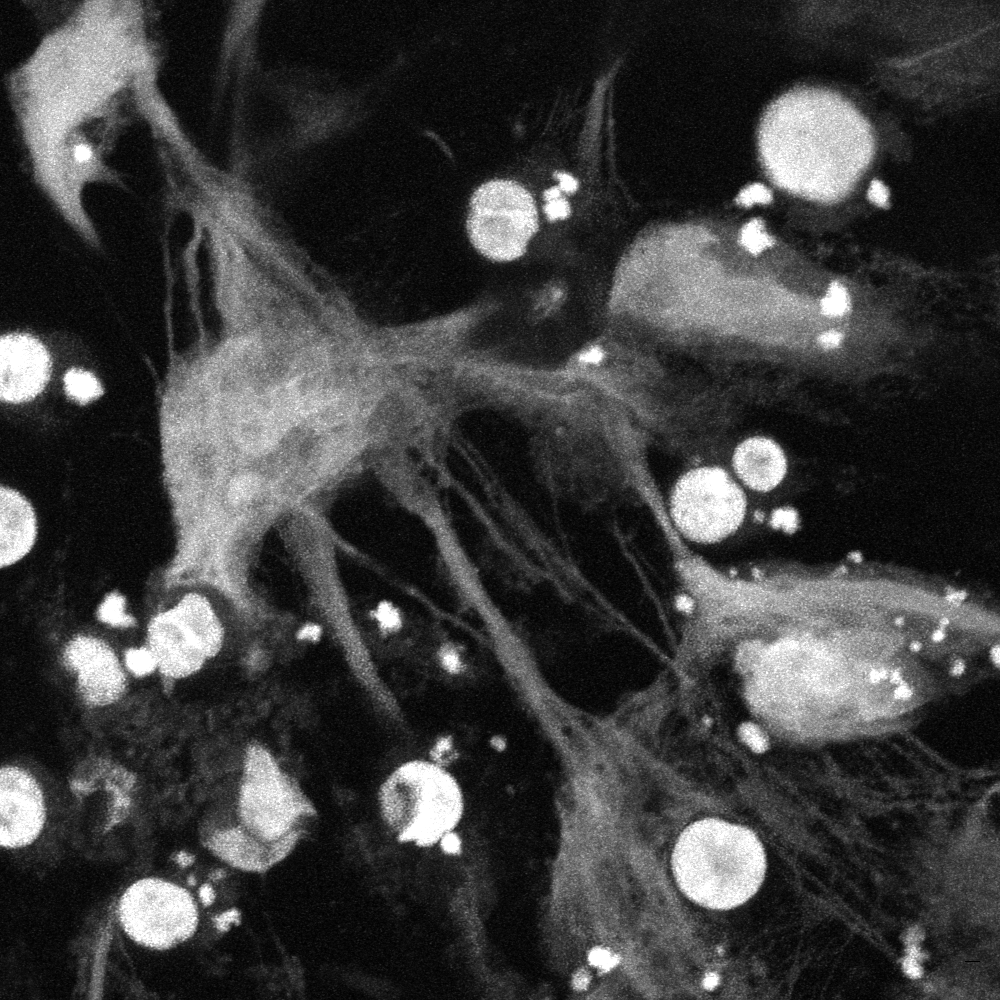

Supplement: Supplementary file 9 — EV Figures Source Data [file 44319_2024_150_MOESM9_ESM.zip › Figure EV2/Fig S2C/image data FB-118/PMA/gray.png]

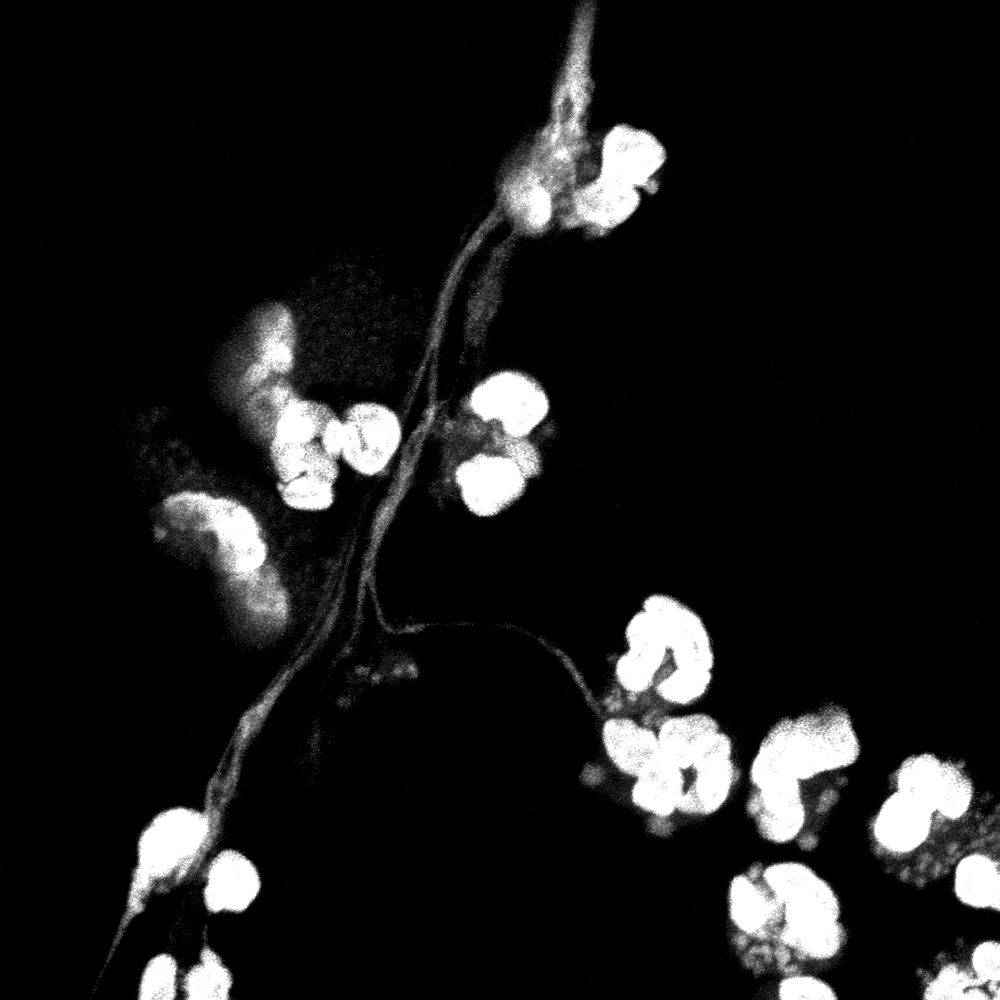

Supplement: Supplementary file 9 — EV Figures Source Data [file 44319_2024_150_MOESM9_ESM.zip › Figure EV2/Fig S2C/image data FB-118/pma nets 1_50/comp new grey.png]

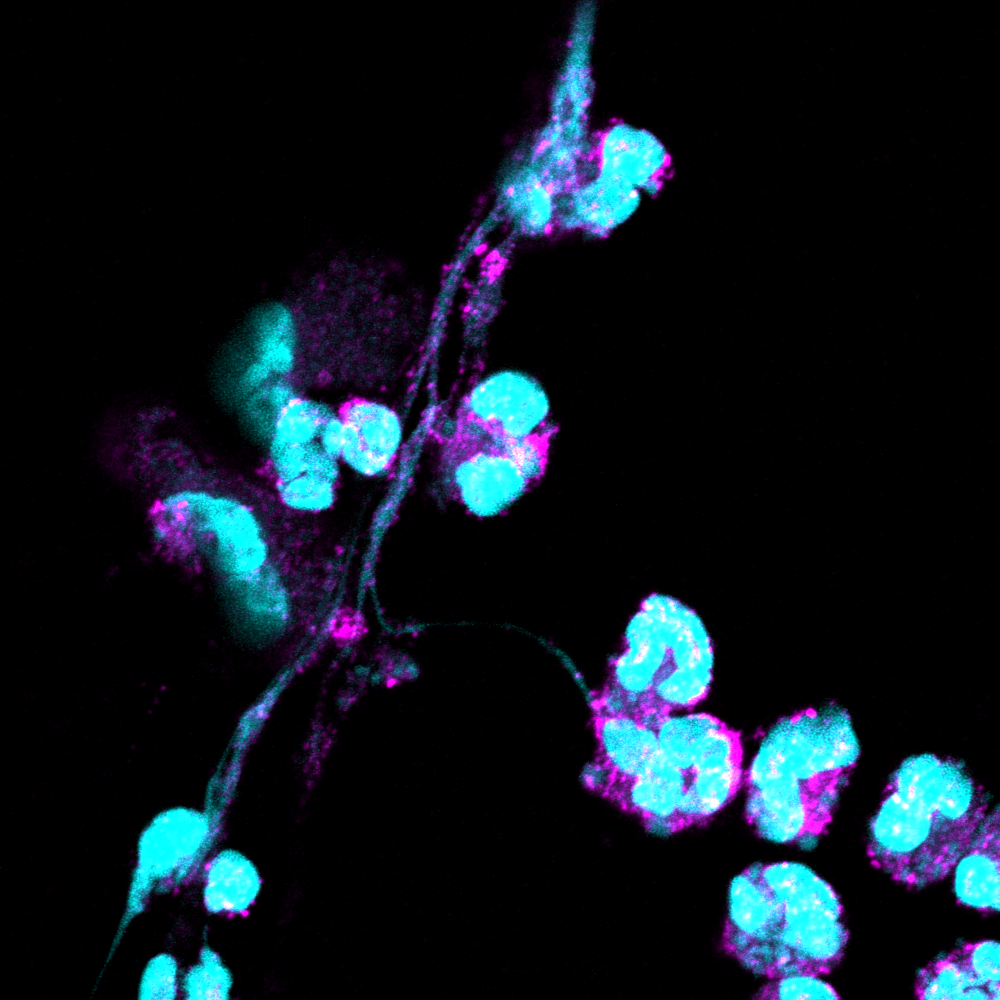

Supplement: Supplementary file 9 — EV Figures Source Data [file 44319_2024_150_MOESM9_ESM.zip › Figure EV2/Fig S2C/image data FB-118/pma nets 1_50/comp new.png]

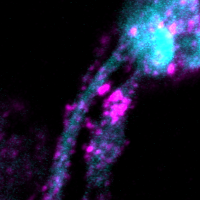

Supplement: Supplementary file 9 — EV Figures Source Data [file 44319_2024_150_MOESM9_ESM.zip › Figure EV2/Fig S2C/image data FB-118/pma nets 1_50/crop new.png]

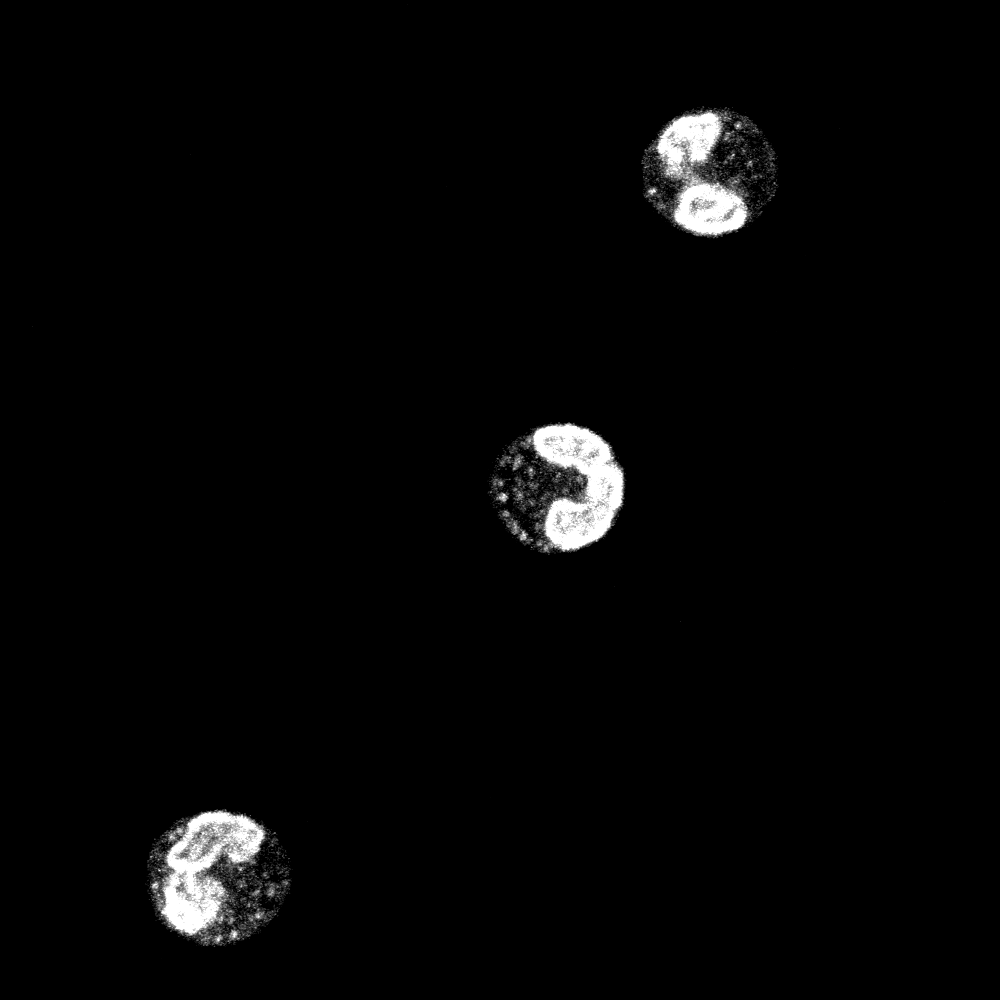

Supplement: Supplementary file 9 — EV Figures Source Data [file 44319_2024_150_MOESM9_ESM.zip › Figure EV2/Fig S2C/image data FB-118/pma nets 1_500/comp new gray.png]

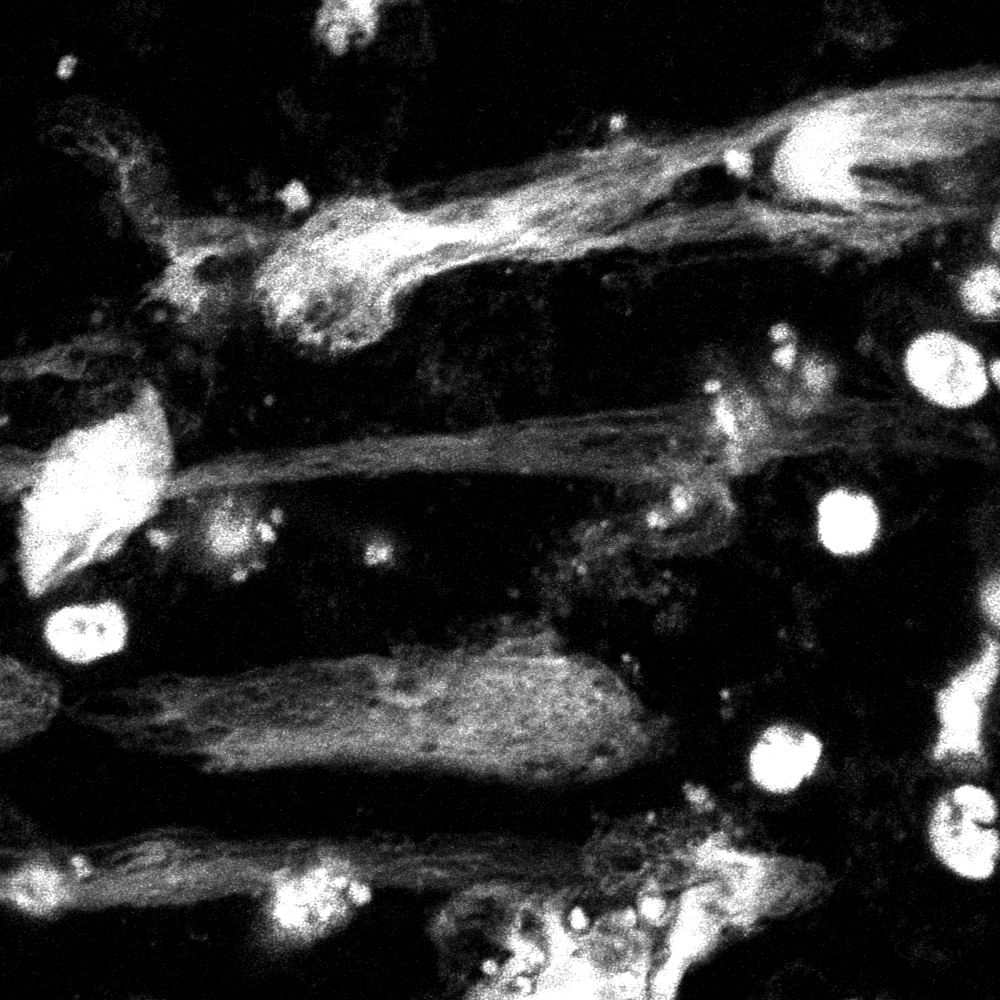

Supplement: Supplementary file 9 — EV Figures Source Data [file 44319_2024_150_MOESM9_ESM.zip › Figure EV2/Fig S2C/image data FB-118/pma nets+rnase inh. 1_50/comp new gray.png]

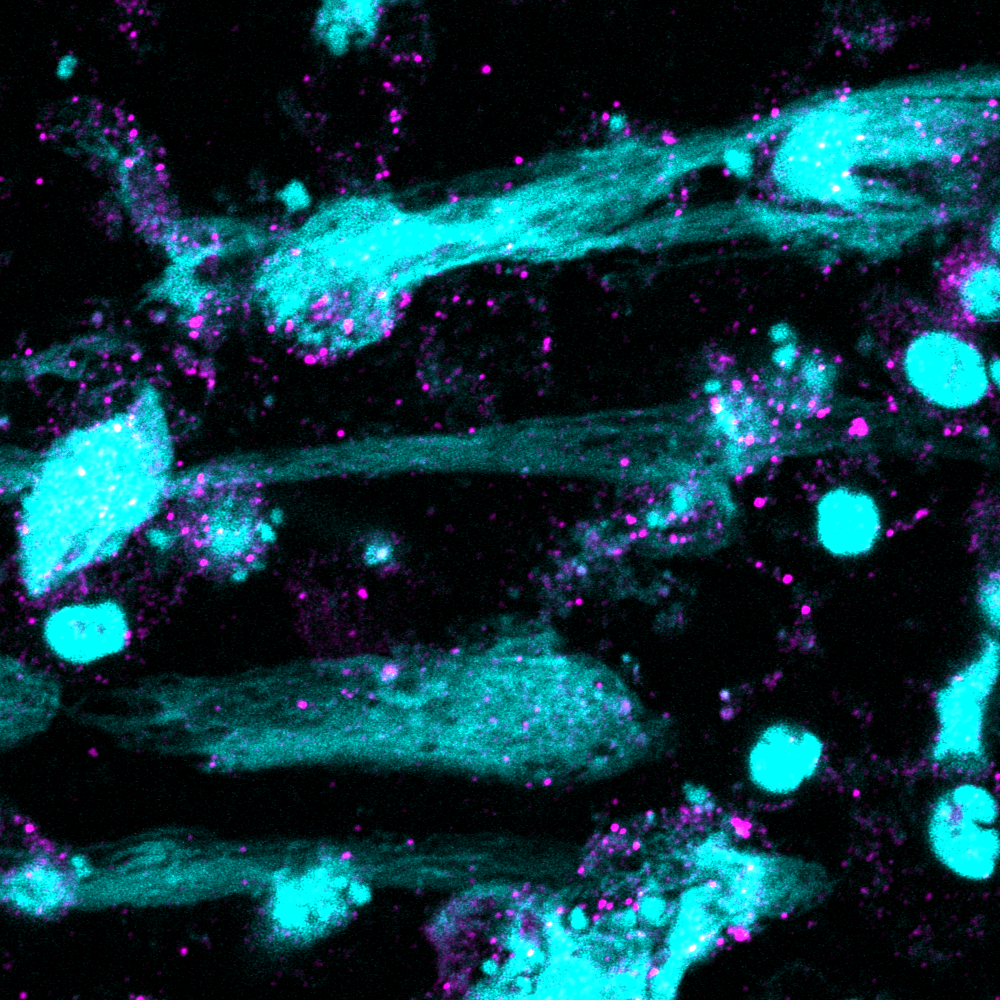

Supplement: Supplementary file 9 — EV Figures Source Data [file 44319_2024_150_MOESM9_ESM.zip › Figure EV2/Fig S2C/image data FB-118/pma nets+rnase inh. 1_50/comp new.png]

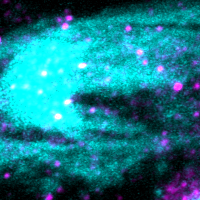

Supplement: Supplementary file 9 — EV Figures Source Data [file 44319_2024_150_MOESM9_ESM.zip › Figure EV2/Fig S2C/image data FB-118/pma nets+rnase inh. 1_50/crop new.png]

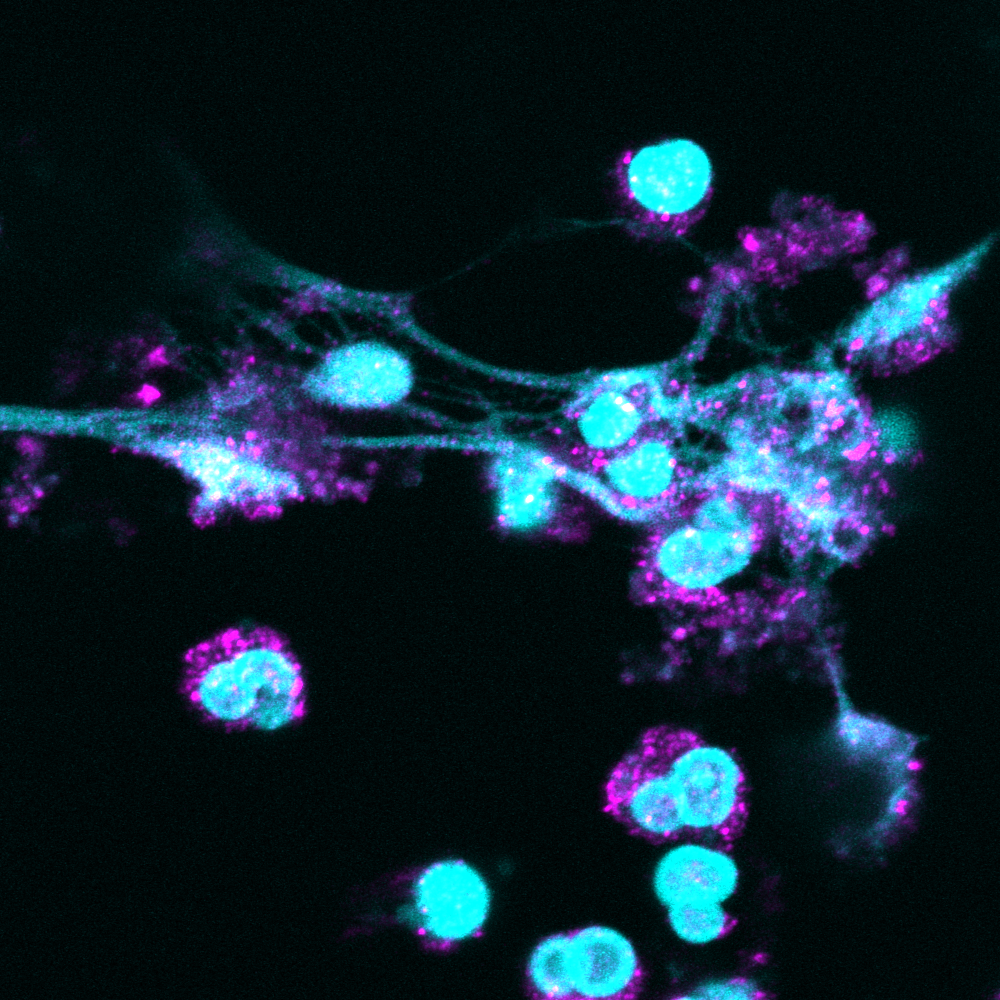

Supplement: Supplementary file 9 — EV Figures Source Data [file 44319_2024_150_MOESM9_ESM.zip › Figure EV2/Fig S2C/image data FB-118/pma nets+rnase inh. 1_500/comp new.png]

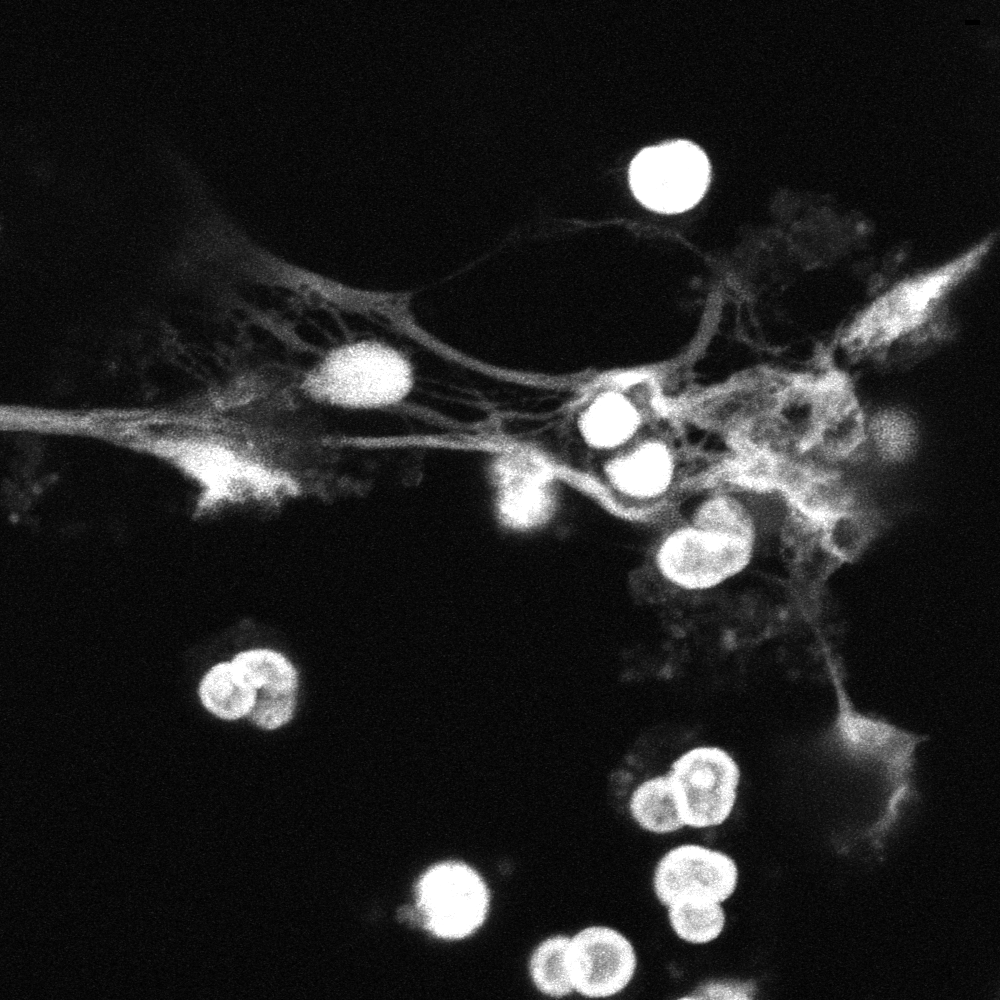

Supplement: Supplementary file 9 — EV Figures Source Data [file 44319_2024_150_MOESM9_ESM.zip › Figure EV2/Fig S2C/image data FB-118/pma nets+rnase inh. 1_500/composite new gray.png]

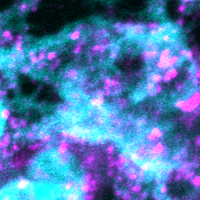

Supplement: Supplementary file 9 — EV Figures Source Data [file 44319_2024_150_MOESM9_ESM.zip › Figure EV2/Fig S2C/image data FB-118/pma nets+rnase inh. 1_500/crop new.png]

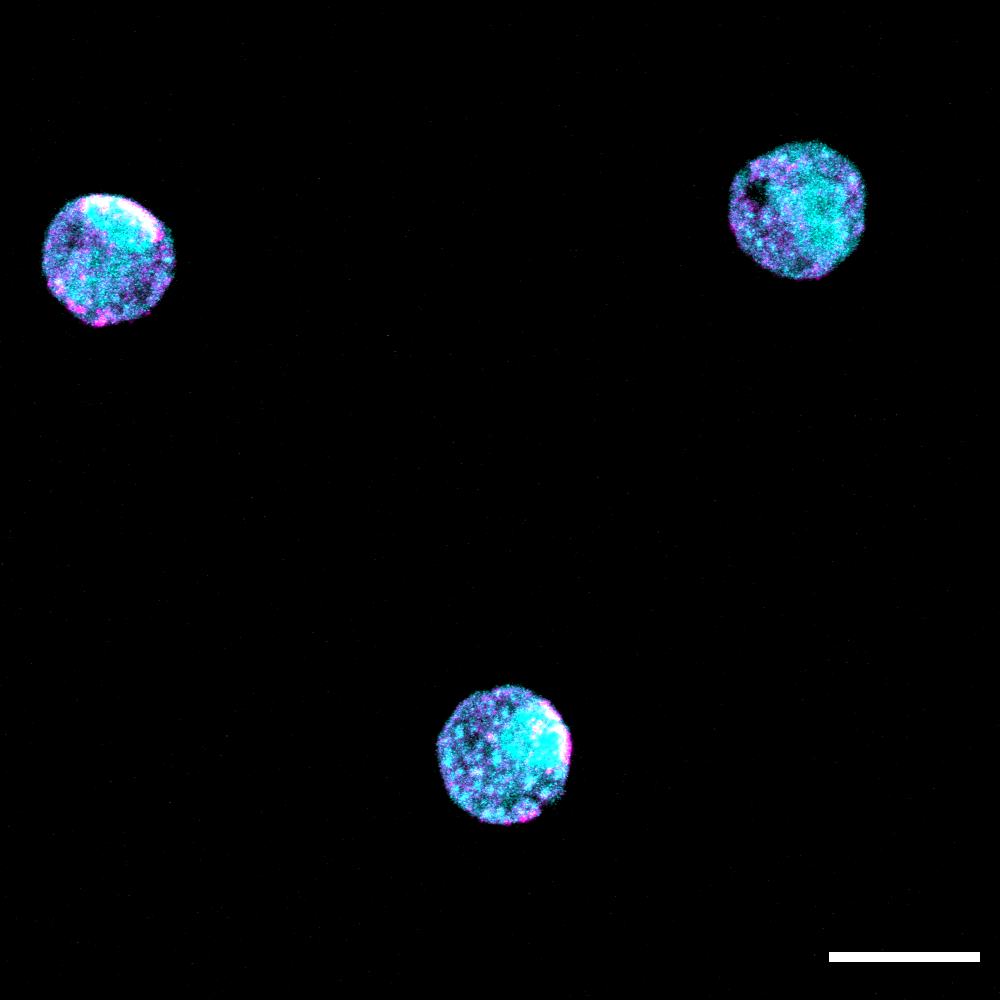

Supplement: Supplementary file 9 — EV Figures Source Data [file 44319_2024_150_MOESM9_ESM.zip › Figure EV2/Fig S2C/image data FB-118/unstim/comp new.png]

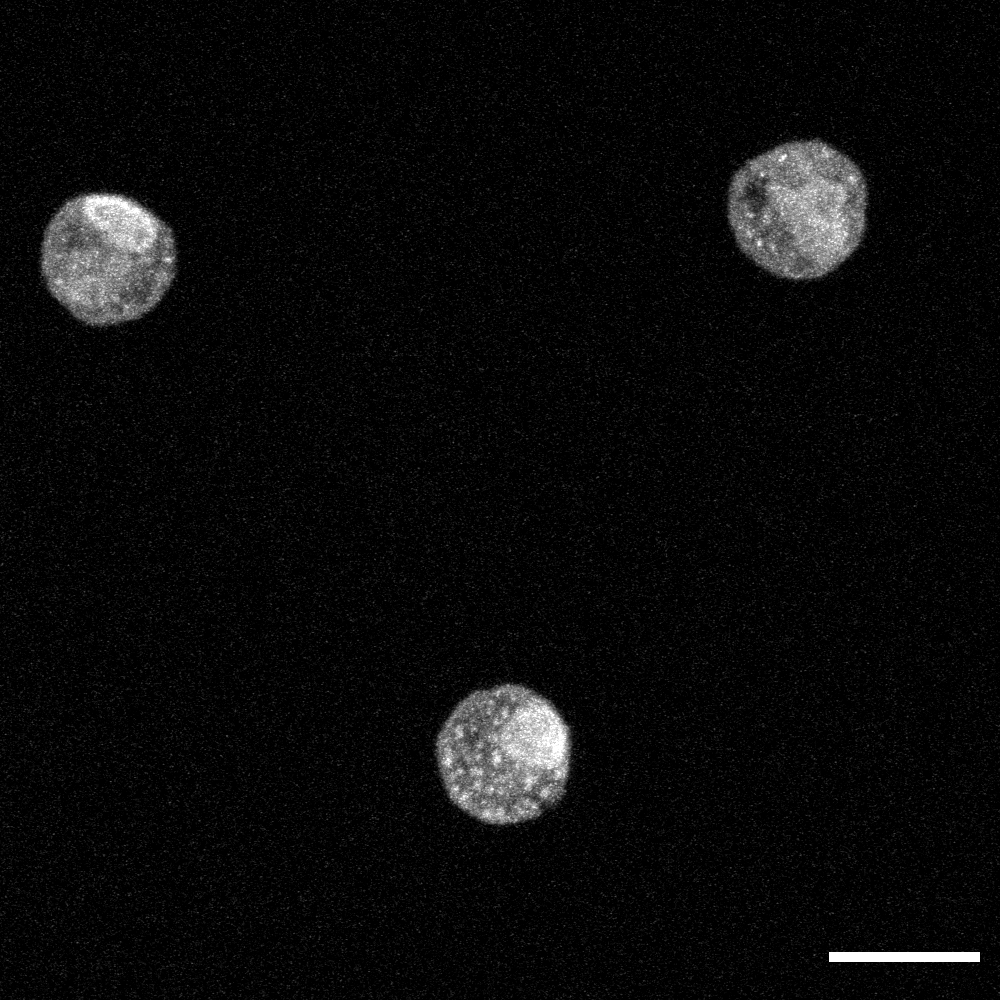

Supplement: Supplementary file 9 — EV Figures Source Data [file 44319_2024_150_MOESM9_ESM.zip › Figure EV2/Fig S2C/image data FB-118/unstim/gray.png]

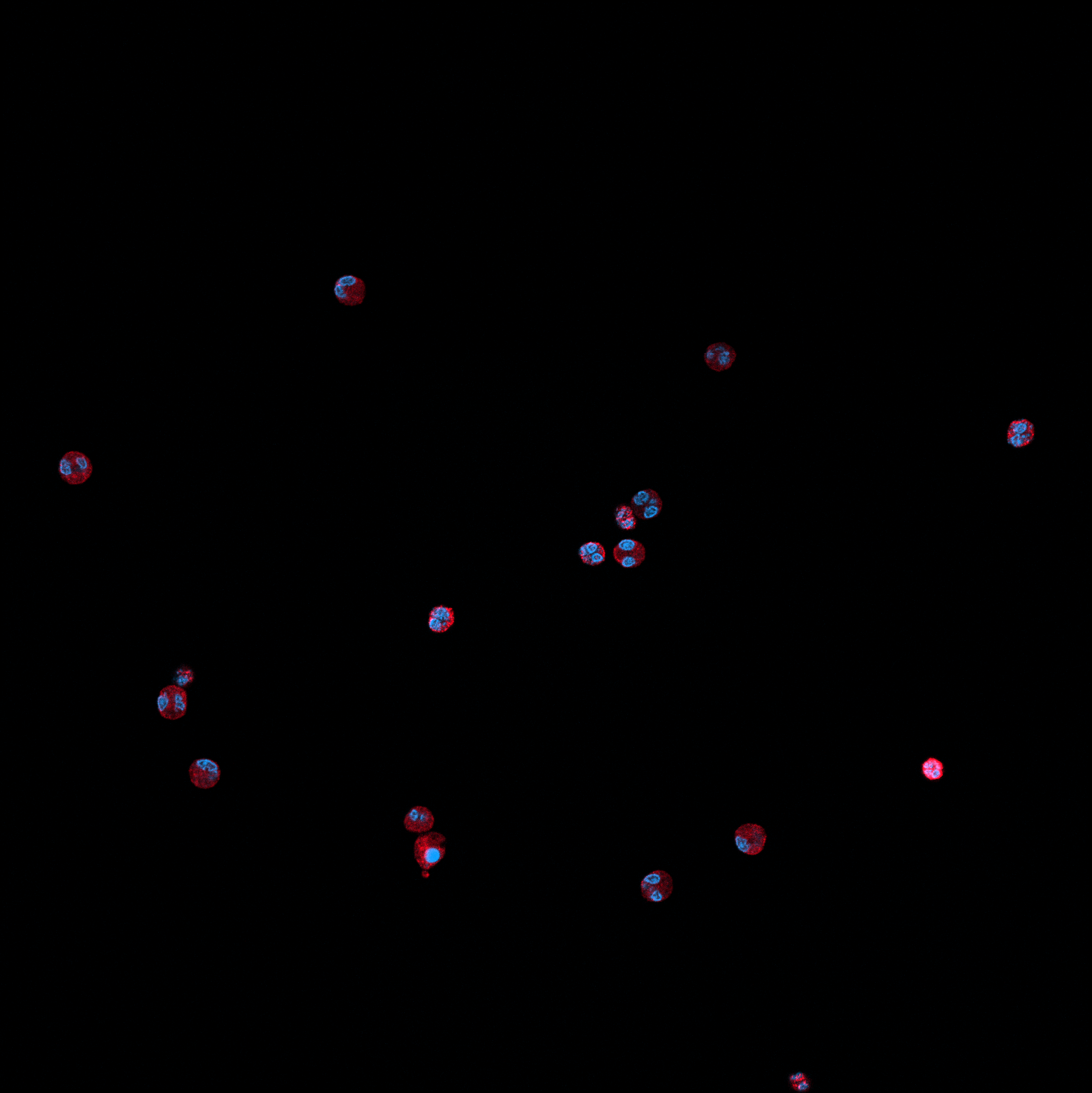

Supplement: Supplementary file 9 — EV Figures Source Data [file 44319_2024_150_MOESM9_ESM.zip › Figure EV2/Fig S2D/image data FB-175/Mock NETs/Experiment-3210-Airyscan Processing-03.png]

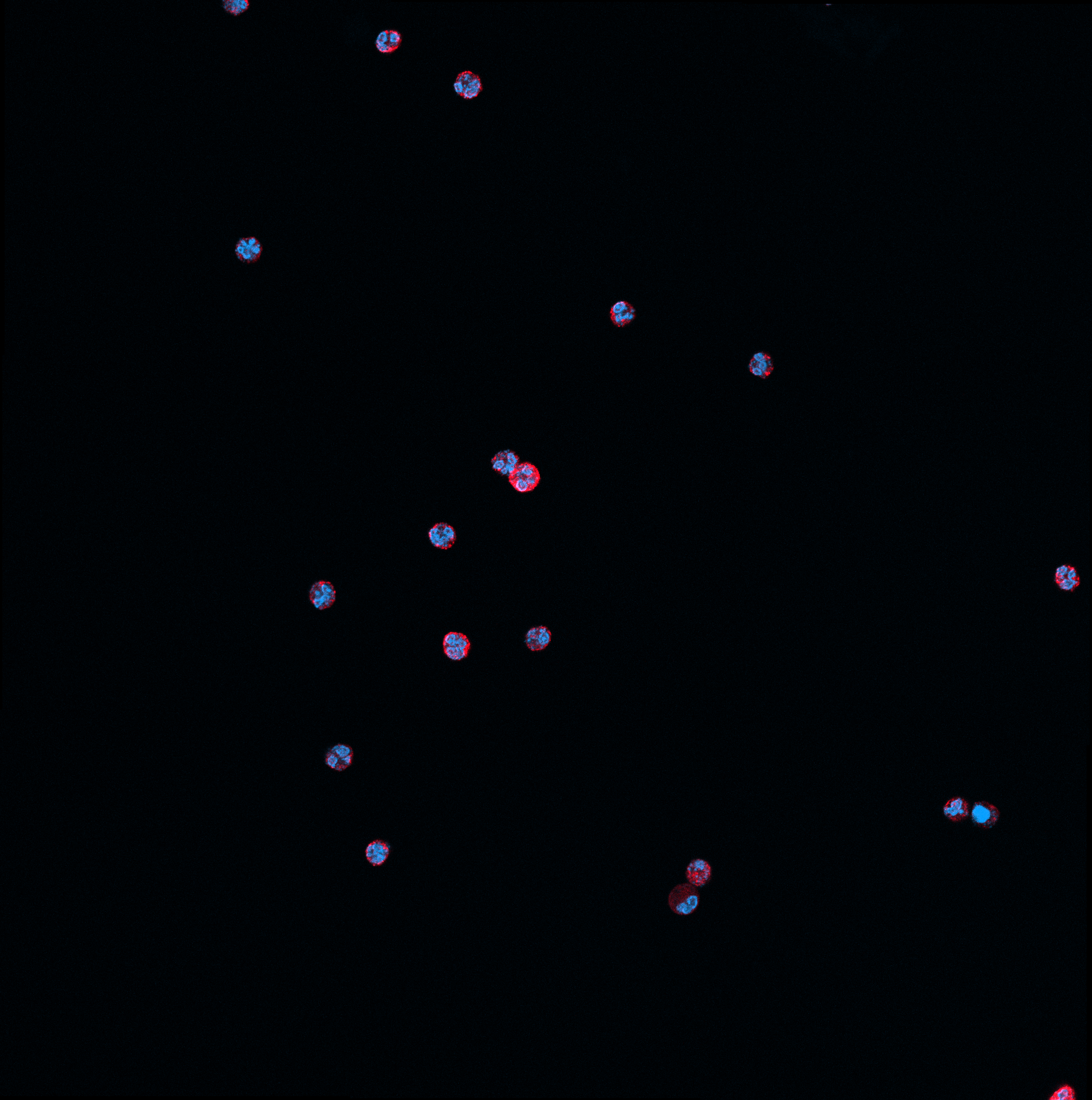

Supplement: Supplementary file 9 — EV Figures Source Data [file 44319_2024_150_MOESM9_ESM.zip › Figure EV2/Fig S2D/image data FB-175/Mock NETs RNase/Experiment-3212-Airyscan Processing-05.png]

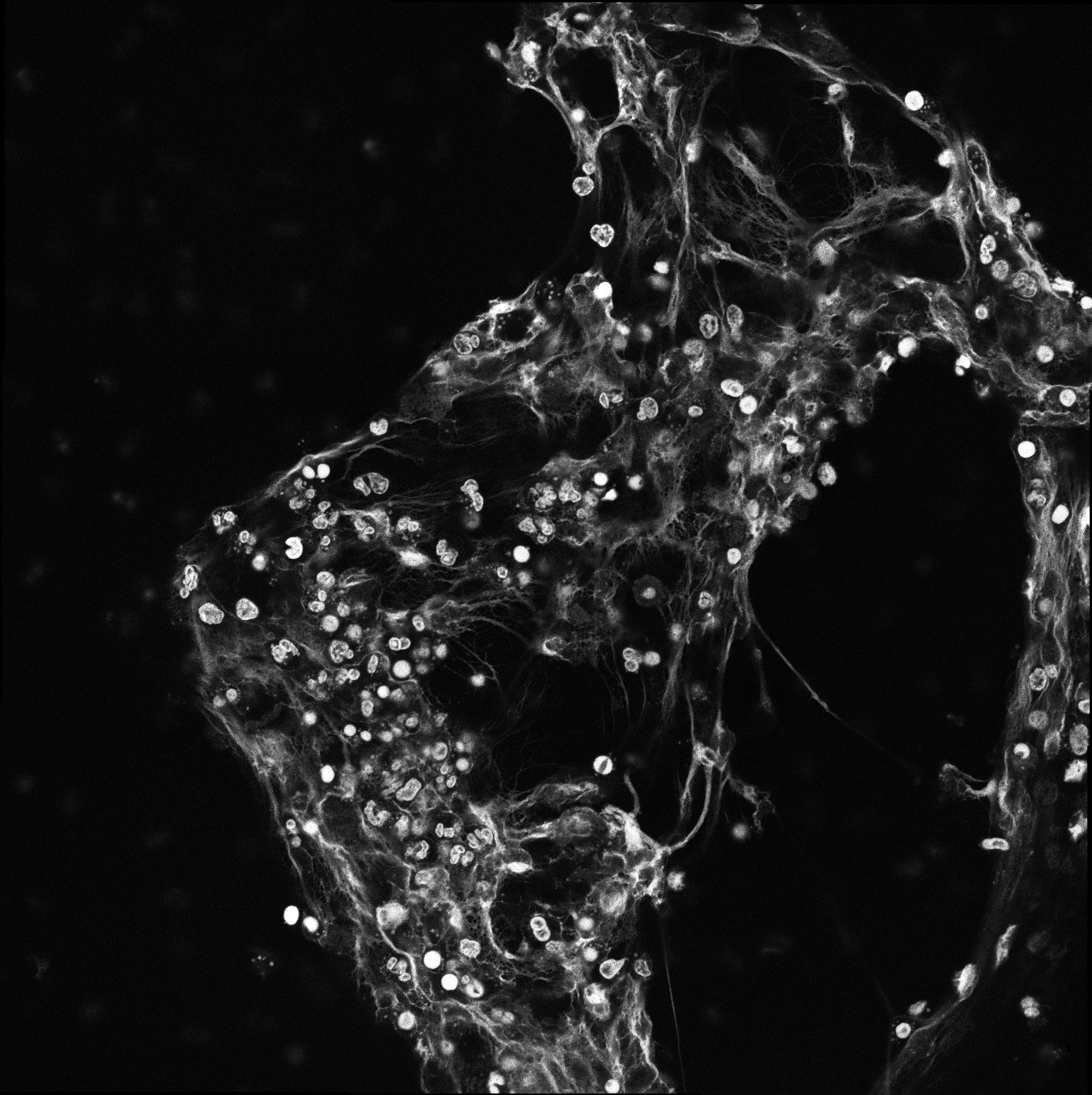

Supplement: Supplementary file 9 — EV Figures Source Data [file 44319_2024_150_MOESM9_ESM.zip › Figure EV2/Fig S2D/image data FB-175/PMA/gray.png]

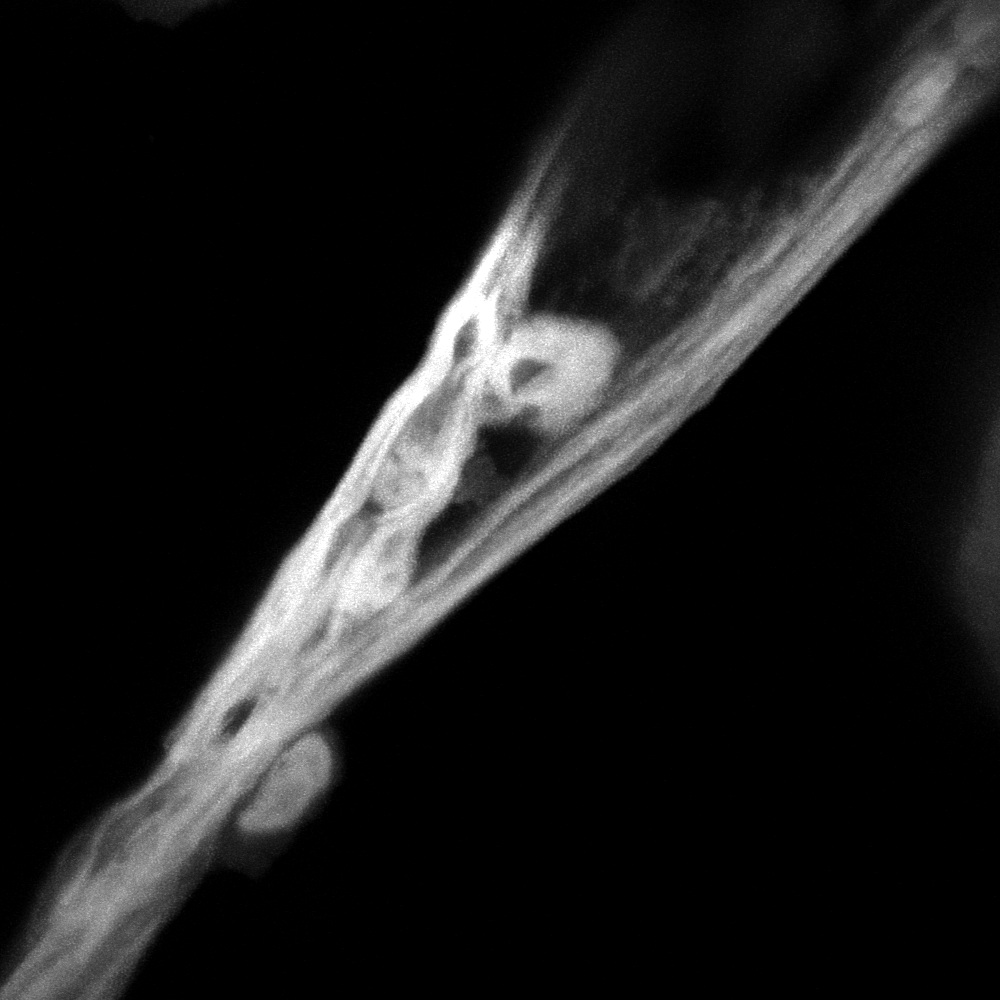

Supplement: Supplementary file 9 — EV Figures Source Data [file 44319_2024_150_MOESM9_ESM.zip › Figure EV2/Fig S2D/image data FB-175/PMA/pma/gray.png]

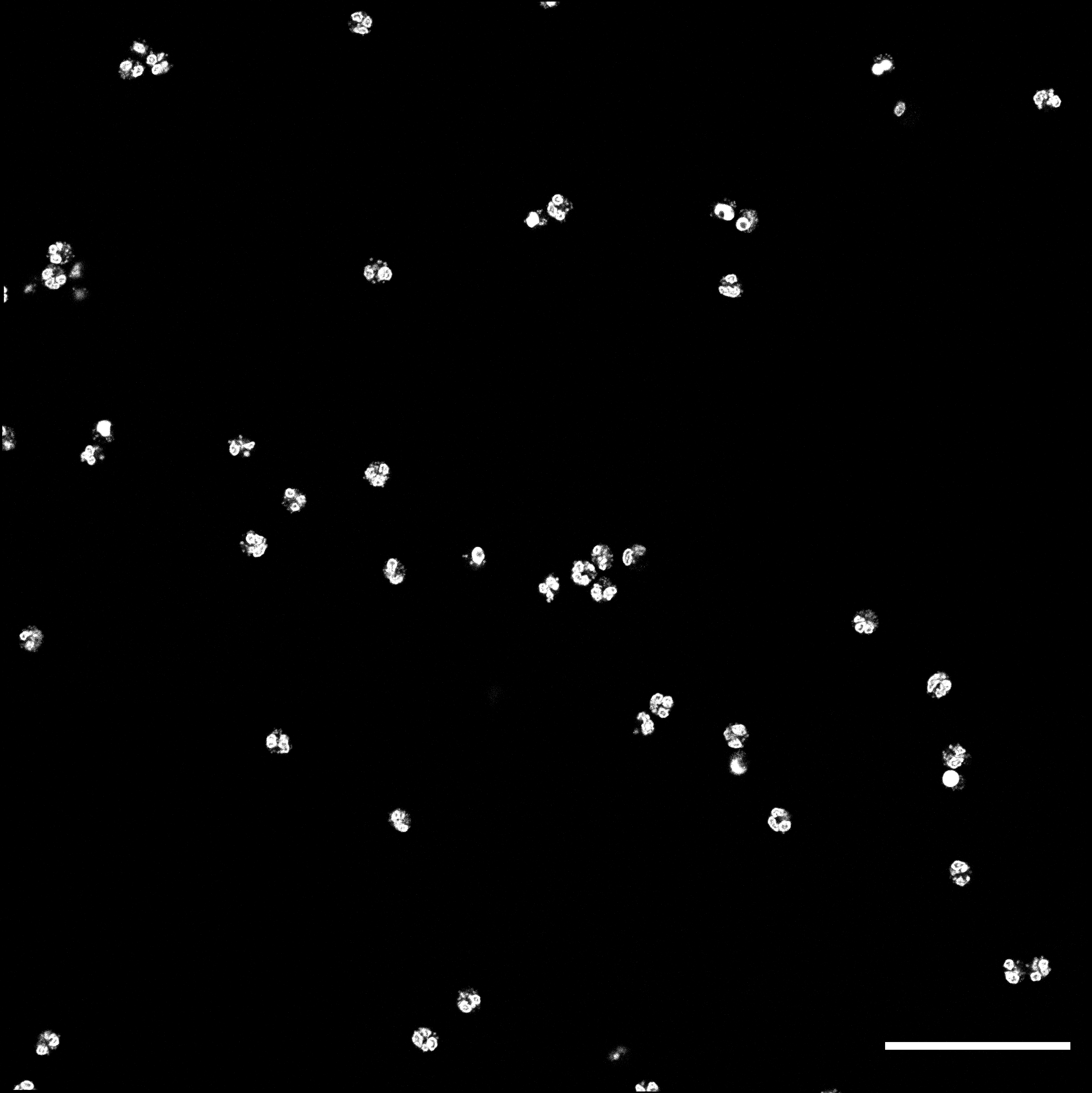

Supplement: Supplementary file 9 — EV Figures Source Data [file 44319_2024_150_MOESM9_ESM.zip › Figure EV2/Fig S2D/image data FB-175/Unstim/comp new gray.png]

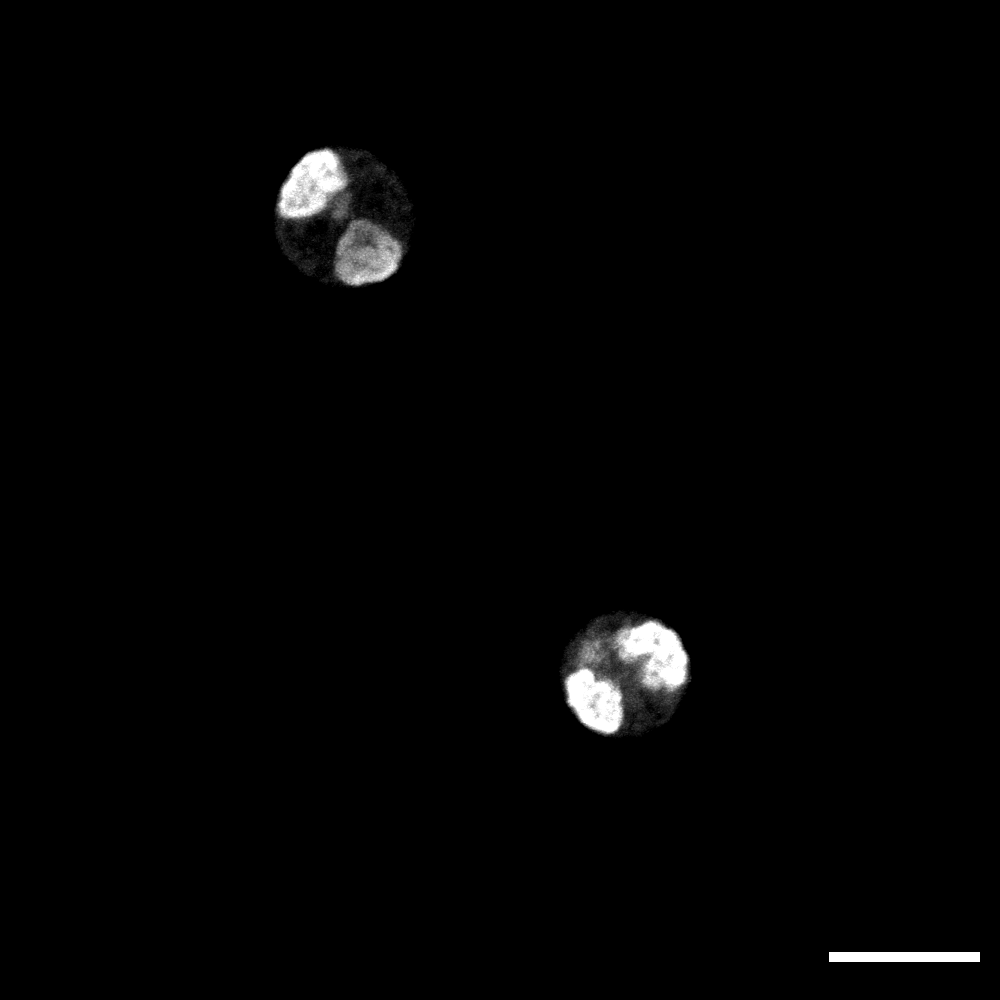

Supplement: Supplementary file 9 — EV Figures Source Data [file 44319_2024_150_MOESM9_ESM.zip › Figure EV2/Fig S2D/image data FB-175/Unstim/unstim/gray.png]

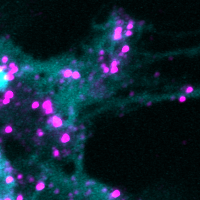

Supplement: Supplementary file 9 — EV Figures Source Data [file 44319_2024_150_MOESM9_ESM.zip › Figure EV2/Fig S2E/image data FB-168/PMA/comp new crop.png]

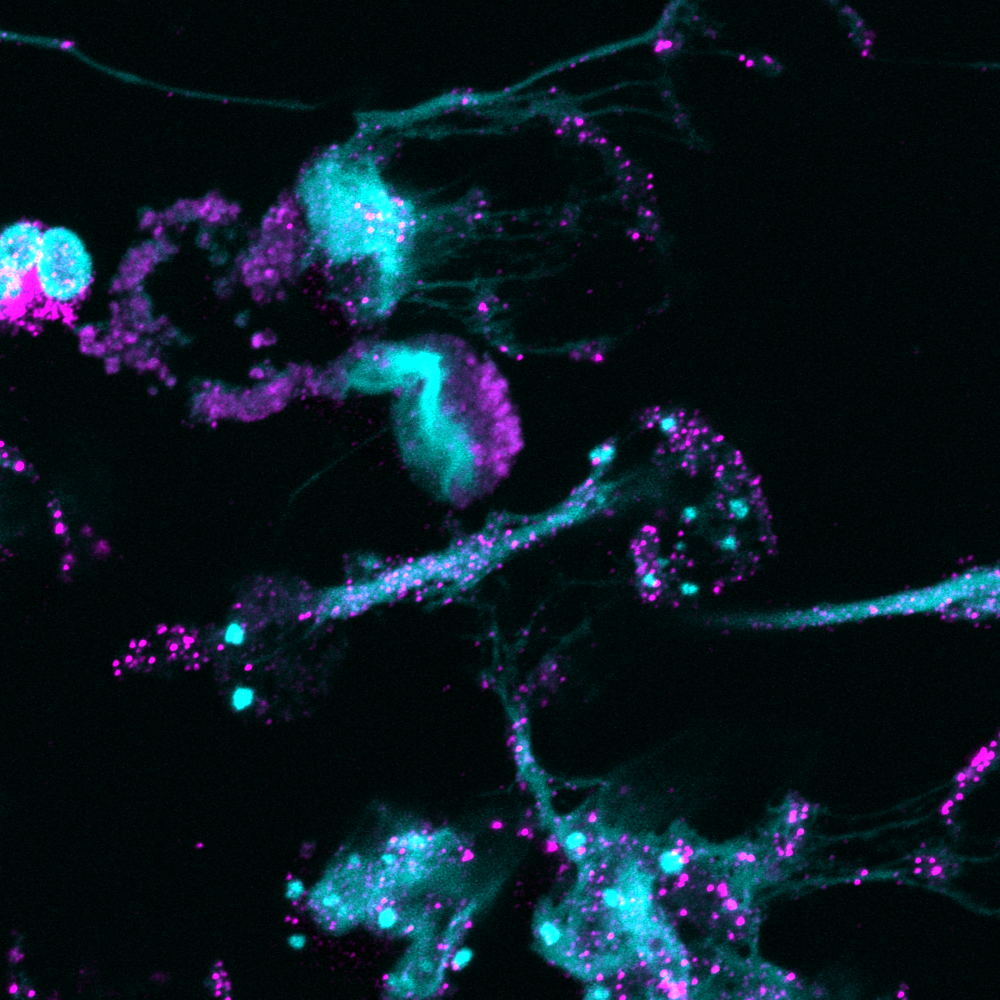

Supplement: Supplementary file 9 — EV Figures Source Data [file 44319_2024_150_MOESM9_ESM.zip › Figure EV2/Fig S2E/image data FB-168/PMA/comp new.png]

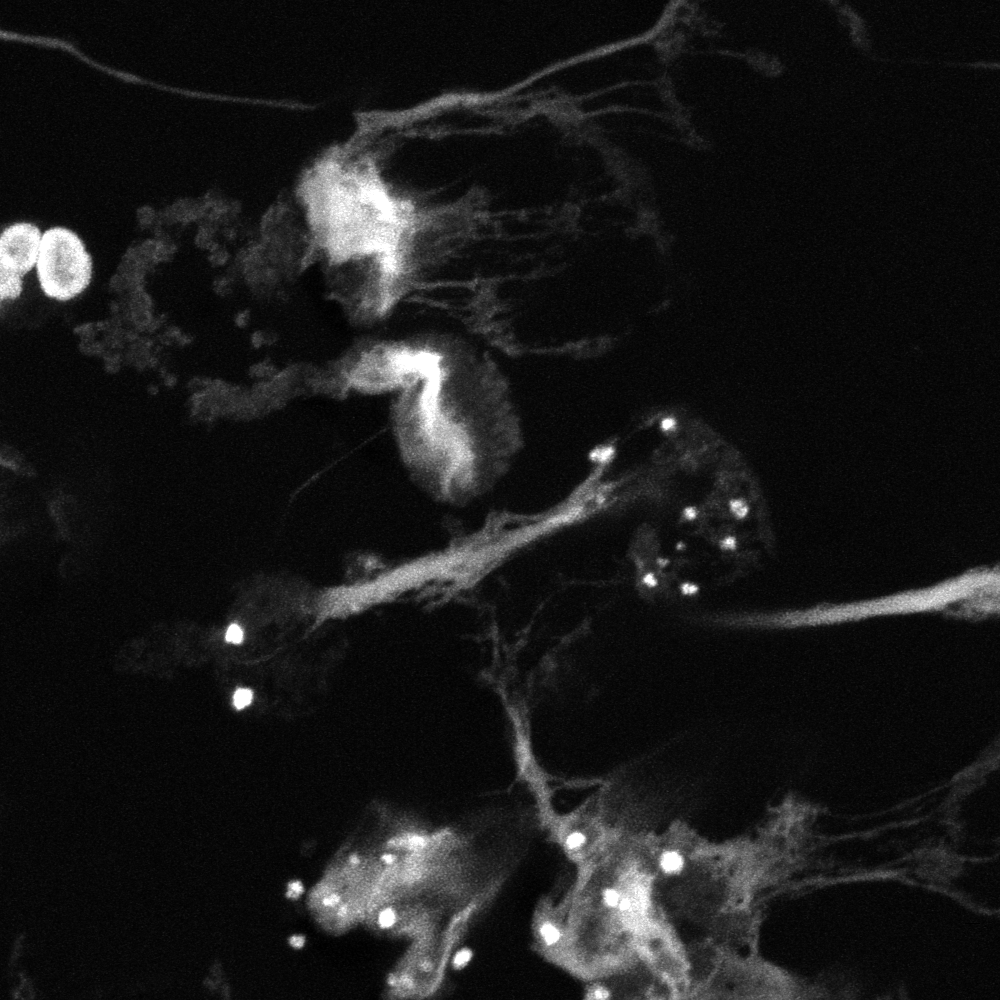

Supplement: Supplementary file 9 — EV Figures Source Data [file 44319_2024_150_MOESM9_ESM.zip › Figure EV2/Fig S2E/image data FB-168/PMA/gray.png]

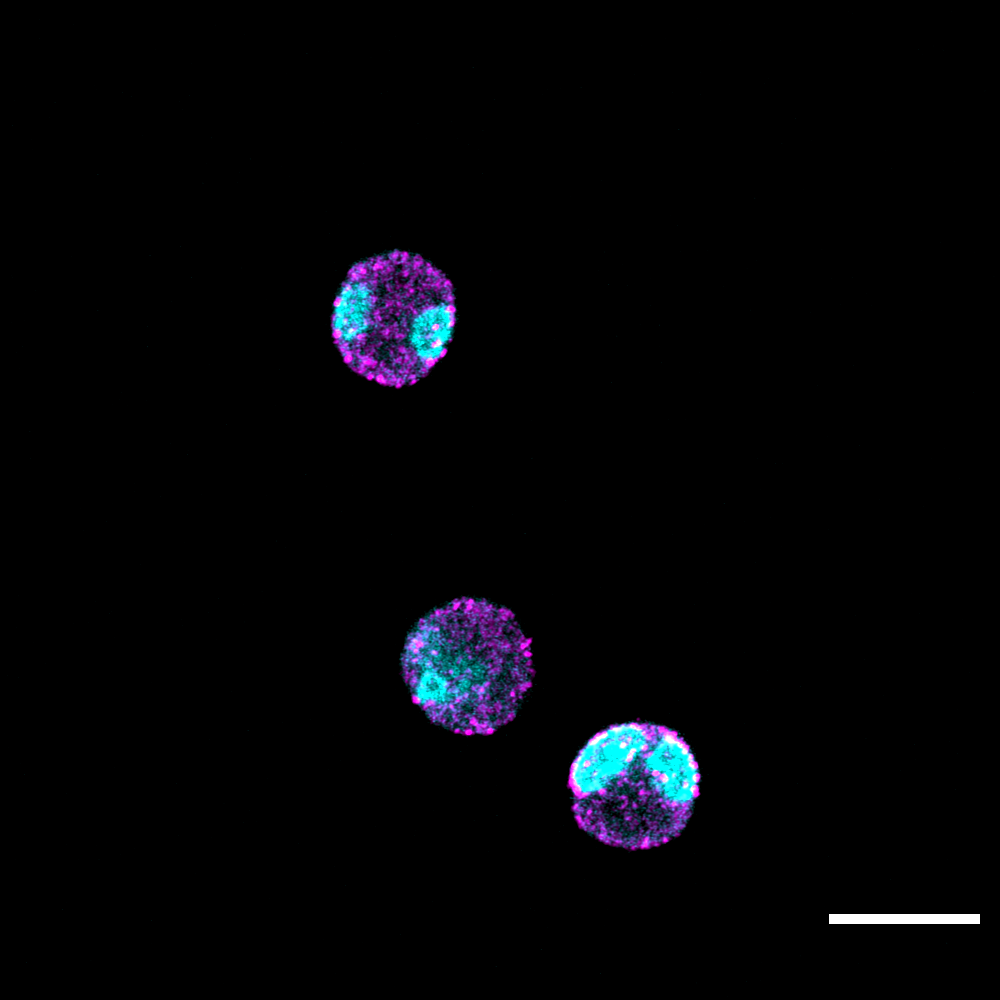

Supplement: Supplementary file 9 — EV Figures Source Data [file 44319_2024_150_MOESM9_ESM.zip › Figure EV2/Fig S2E/image data FB-168/unstim/comp new.png]

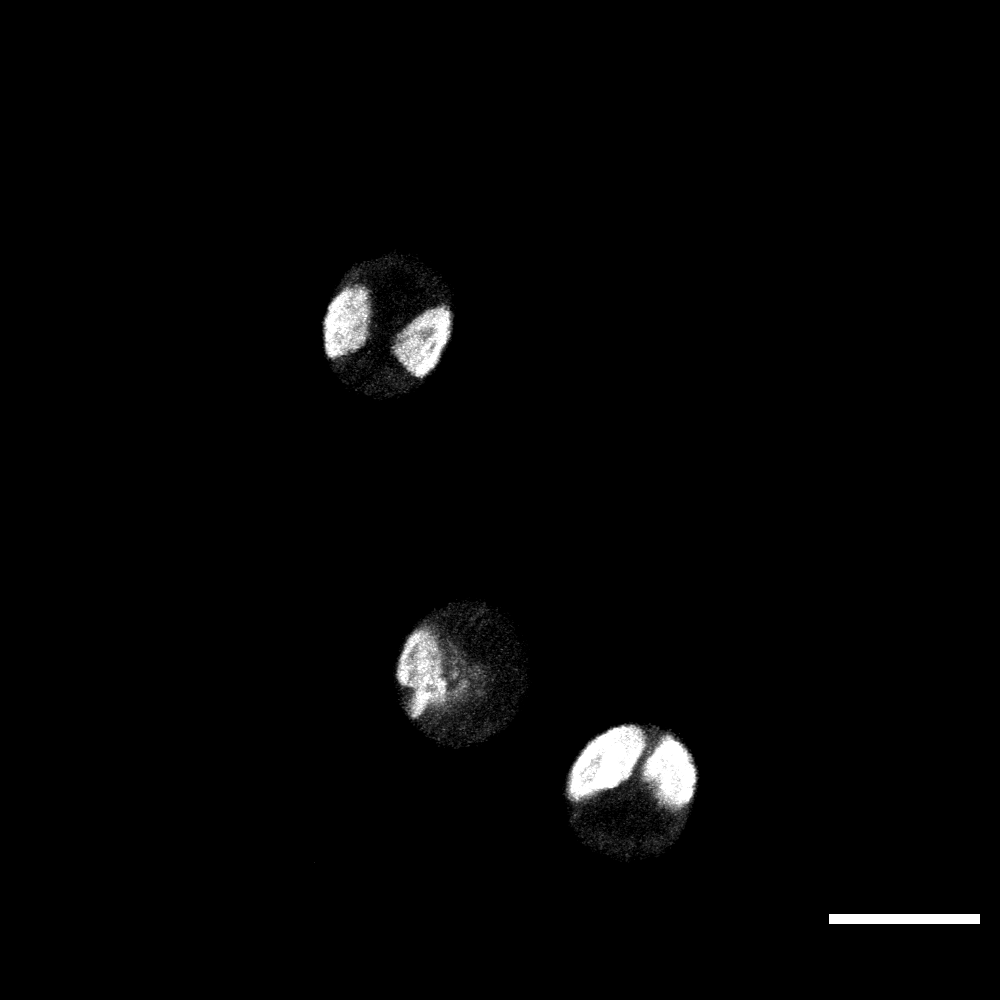

Supplement: Supplementary file 9 — EV Figures Source Data [file 44319_2024_150_MOESM9_ESM.zip › Figure EV2/Fig S2E/image data FB-168/unstim/gray.png]

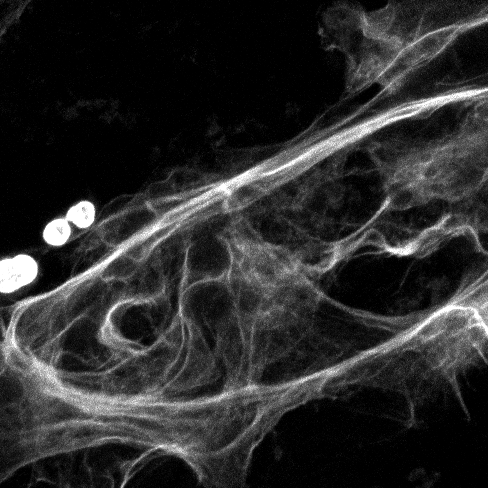

Supplement: Supplementary file 9 — EV Figures Source Data [file 44319_2024_150_MOESM9_ESM.zip › Figure EV1/Fig S1A/secondary antibody controls/pma 2nd only.png]

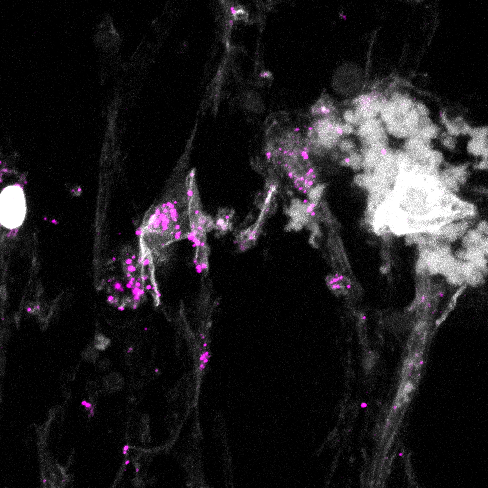

Supplement: Supplementary file 9 — EV Figures Source Data [file 44319_2024_150_MOESM9_ESM.zip › Figure EV1/Fig S1A/secondary antibody controls/pma.png]

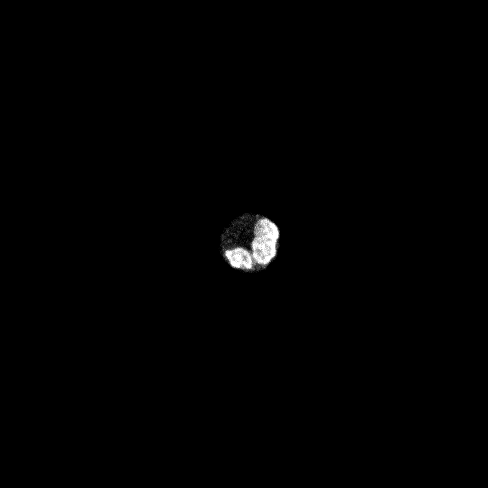

Supplement: Supplementary file 9 — EV Figures Source Data [file 44319_2024_150_MOESM9_ESM.zip › Figure EV1/Fig S1A/secondary antibody controls/unstim 2nd only.png]

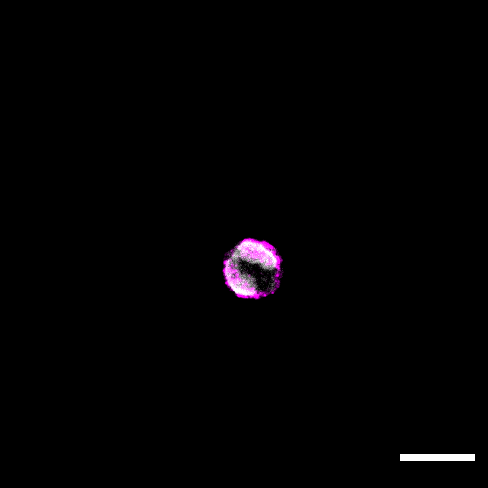

Supplement: Supplementary file 9 — EV Figures Source Data [file 44319_2024_150_MOESM9_ESM.zip › Figure EV1/Fig S1A/secondary antibody controls/unstim.png]

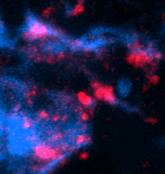

Supplement: Supplementary file 9 — EV Figures Source Data [file 44319_2024_150_MOESM9_ESM.zip › Figure EV1/Fig S1C/FB-176 WT Mice/live candida crop.png]

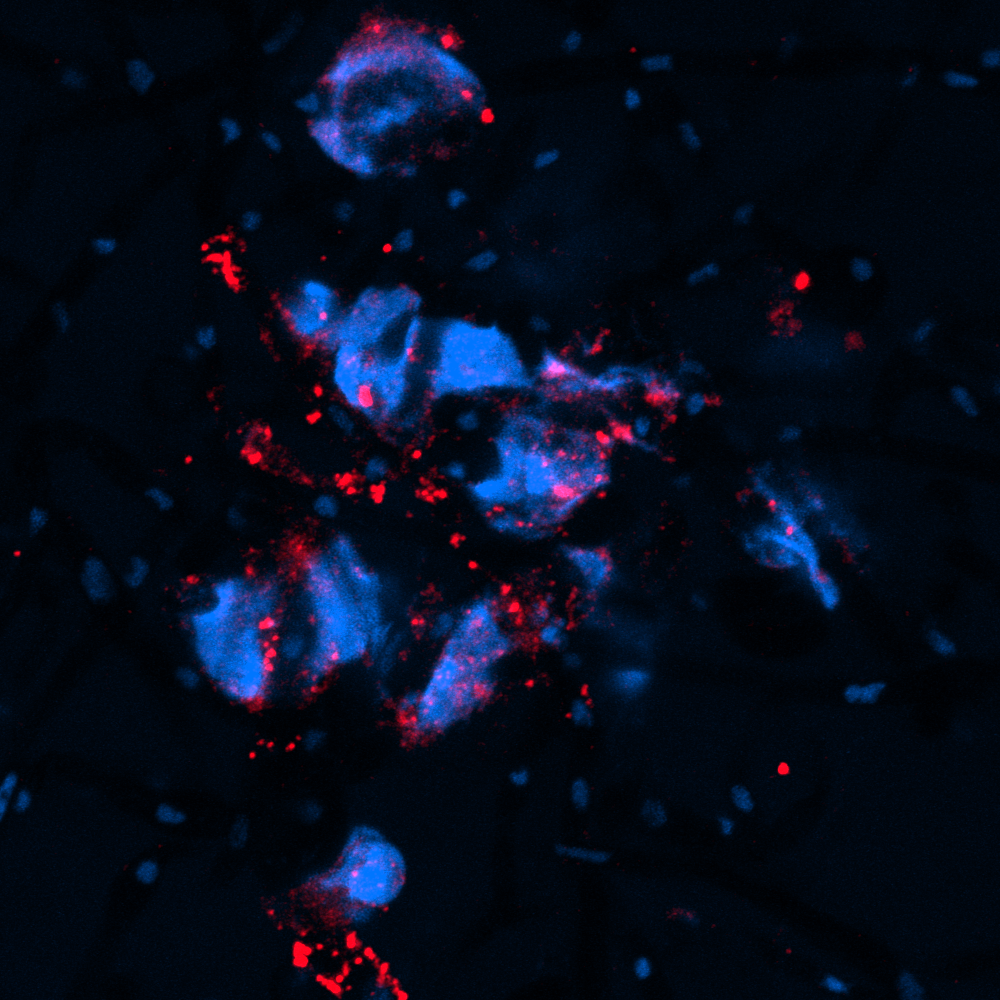

Supplement: Supplementary file 9 — EV Figures Source Data [file 44319_2024_150_MOESM9_ESM.zip › Figure EV1/Fig S1C/FB-176 WT Mice/live candida.png]

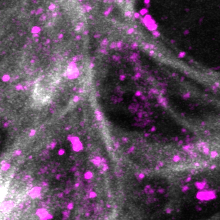

Supplement: Supplementary file 9 — EV Figures Source Data [file 44319_2024_150_MOESM9_ESM.zip › Figure EV1/Fig S1C/FB-176 WT Mice/Nigericin/comp new gray crop.png]

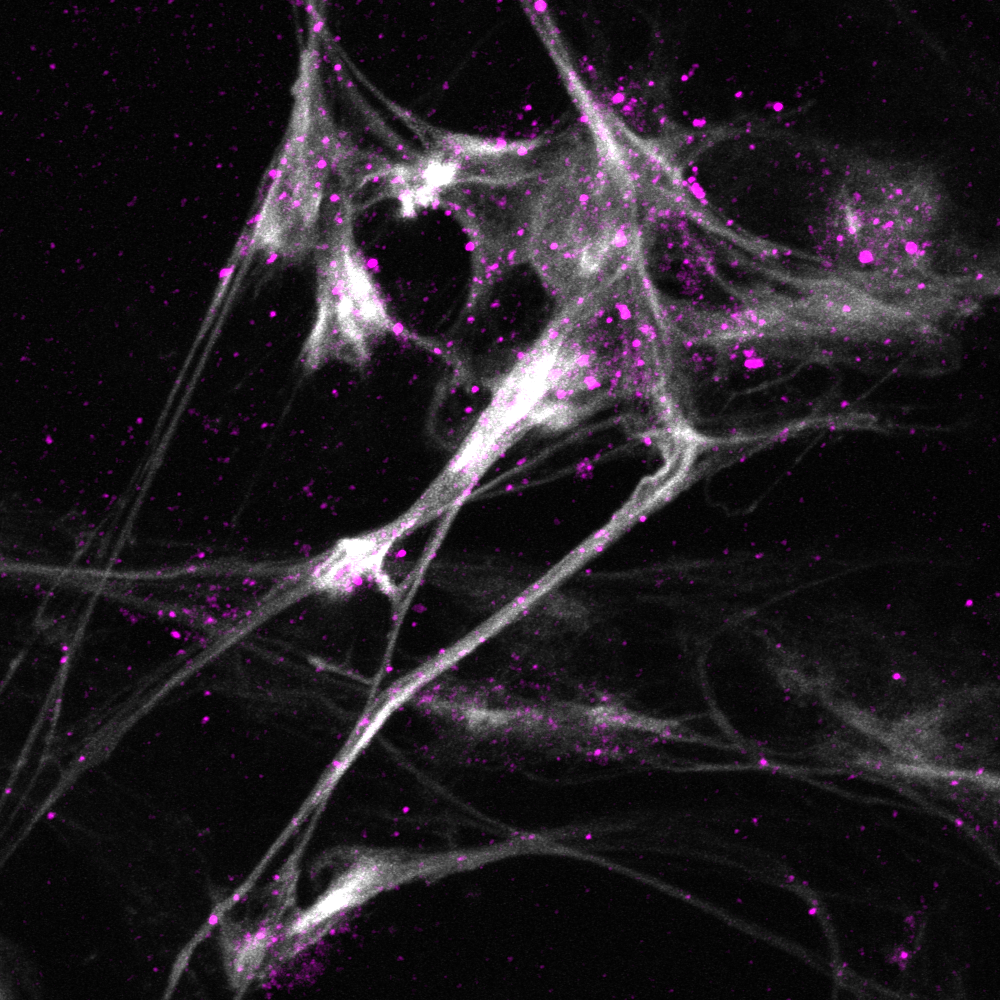

Supplement: Supplementary file 9 — EV Figures Source Data [file 44319_2024_150_MOESM9_ESM.zip › Figure EV1/Fig S1C/FB-176 WT Mice/Nigericin/comp new gray.png]

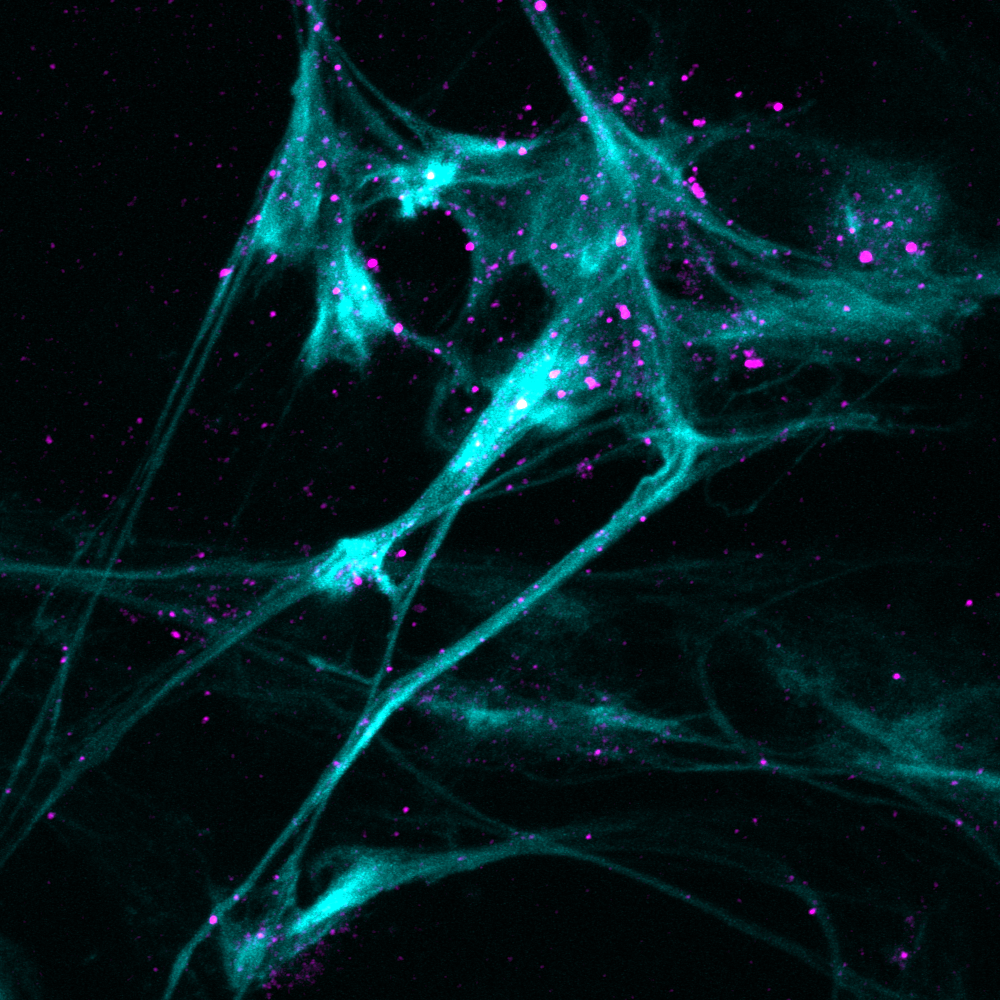

Supplement: Supplementary file 9 — EV Figures Source Data [file 44319_2024_150_MOESM9_ESM.zip › Figure EV1/Fig S1C/FB-176 WT Mice/Nigericin/comp new.png]

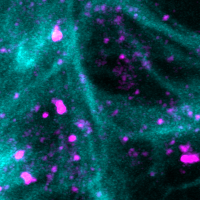

Supplement: Supplementary file 9 — EV Figures Source Data [file 44319_2024_150_MOESM9_ESM.zip › Figure EV1/Fig S1C/FB-176 WT Mice/Nigericin/crop new.png]

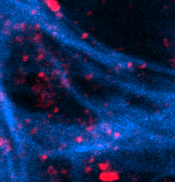

Supplement: Supplementary file 9 — EV Figures Source Data [file 44319_2024_150_MOESM9_ESM.zip › Figure EV1/Fig S1C/FB-176 WT Mice/nigericin crop.png]

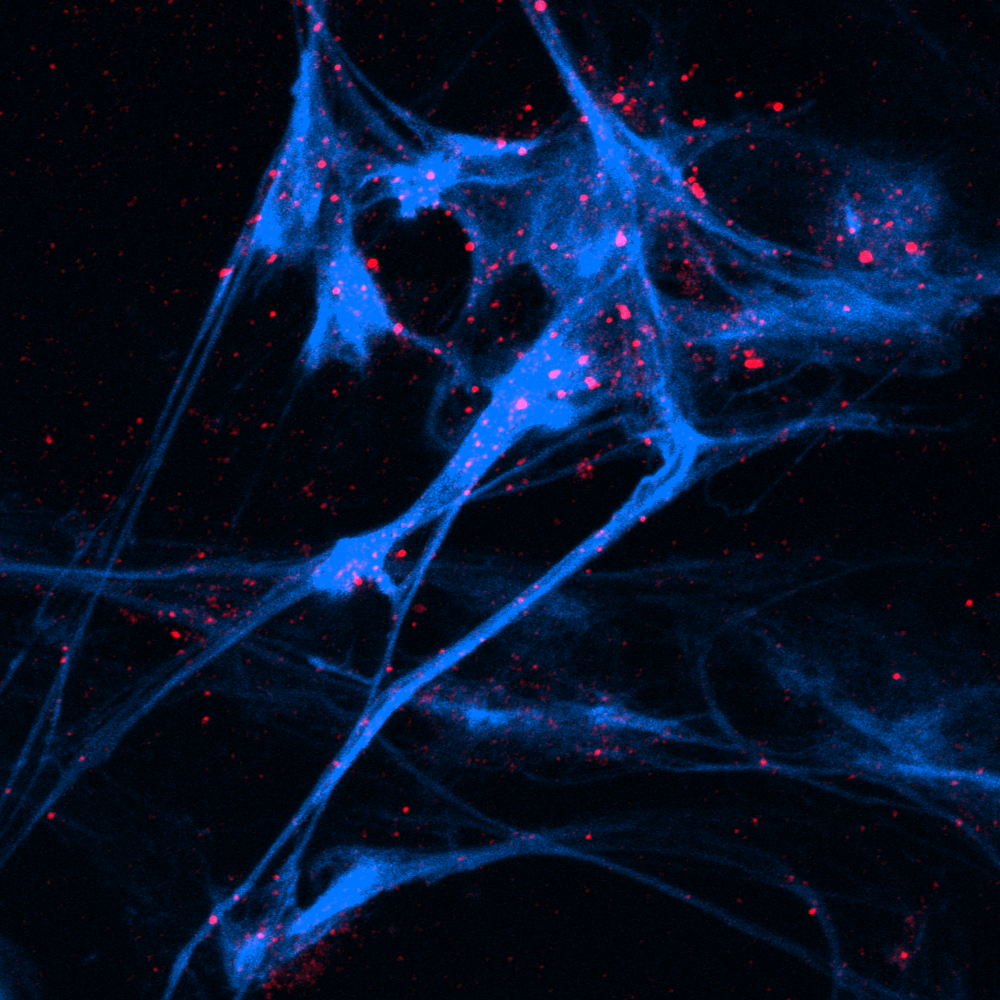

Supplement: Supplementary file 9 — EV Figures Source Data [file 44319_2024_150_MOESM9_ESM.zip › Figure EV1/Fig S1C/FB-176 WT Mice/nigericin.png]

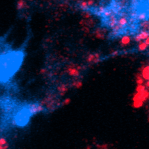

Supplement: Supplementary file 9 — EV Figures Source Data [file 44319_2024_150_MOESM9_ESM.zip › Figure EV1/Fig S1C/FB-176 WT Mice/PMA crop.png]

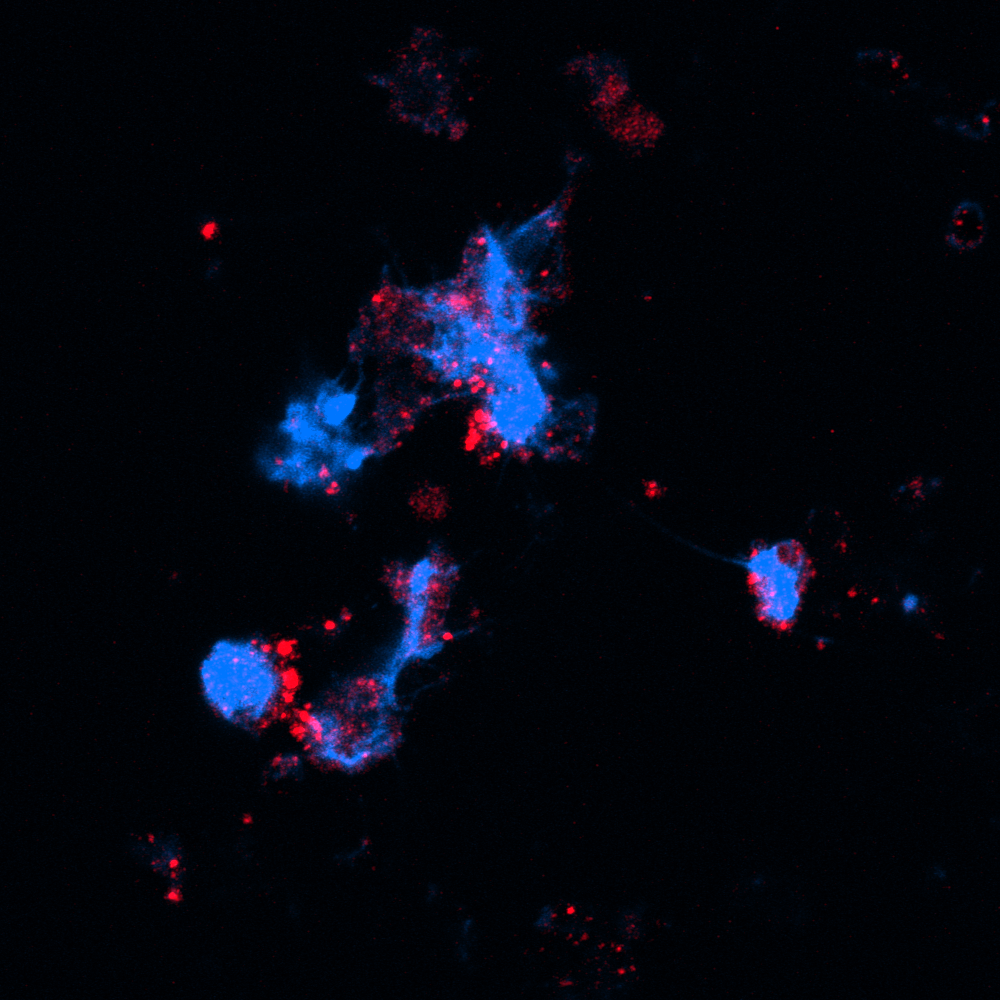

Supplement: Supplementary file 9 — EV Figures Source Data [file 44319_2024_150_MOESM9_ESM.zip › Figure EV1/Fig S1C/FB-176 WT Mice/PMA.png]

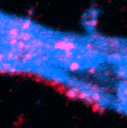

Supplement: Supplementary file 9 — EV Figures Source Data [file 44319_2024_150_MOESM9_ESM.zip › Figure EV1/Fig S1C/FB-176 WT Mice/ssRNA-LL37 crop.png]

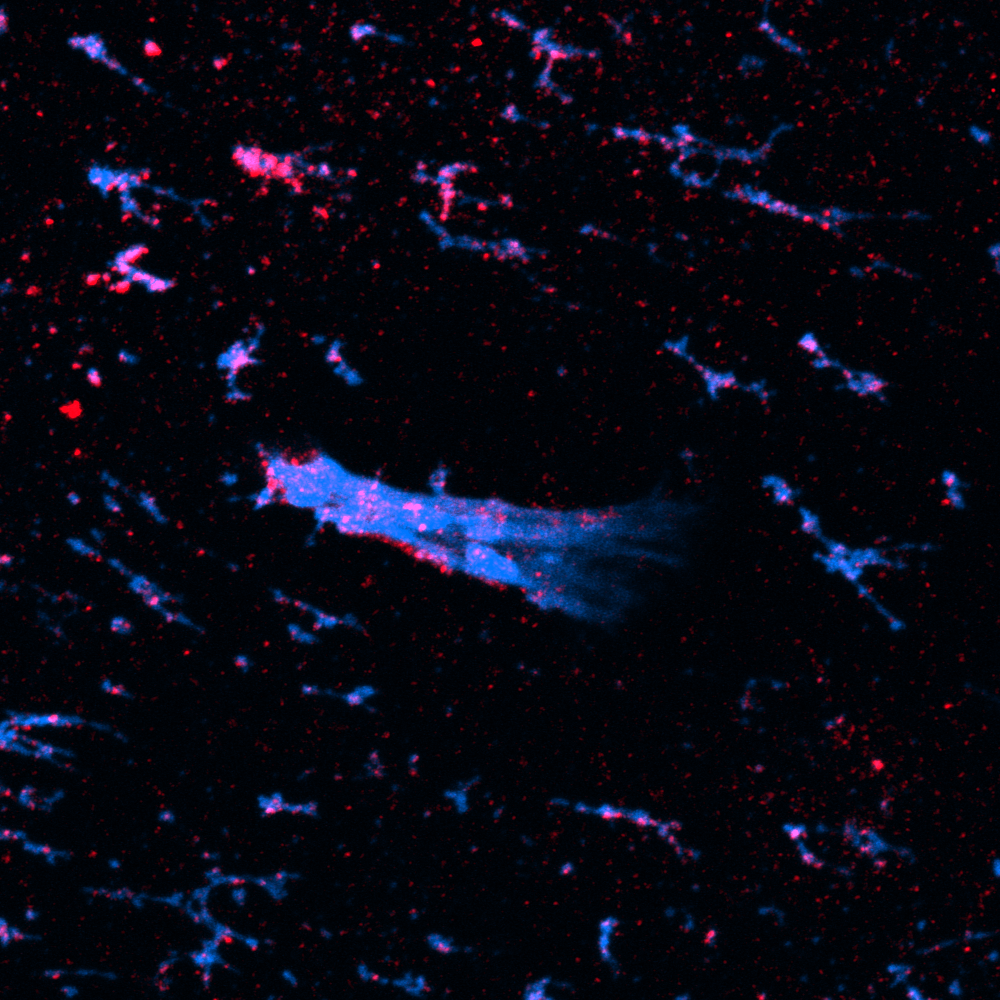

Supplement: Supplementary file 9 — EV Figures Source Data [file 44319_2024_150_MOESM9_ESM.zip › Figure EV1/Fig S1C/FB-176 WT Mice/ssRNA-LL37.png]

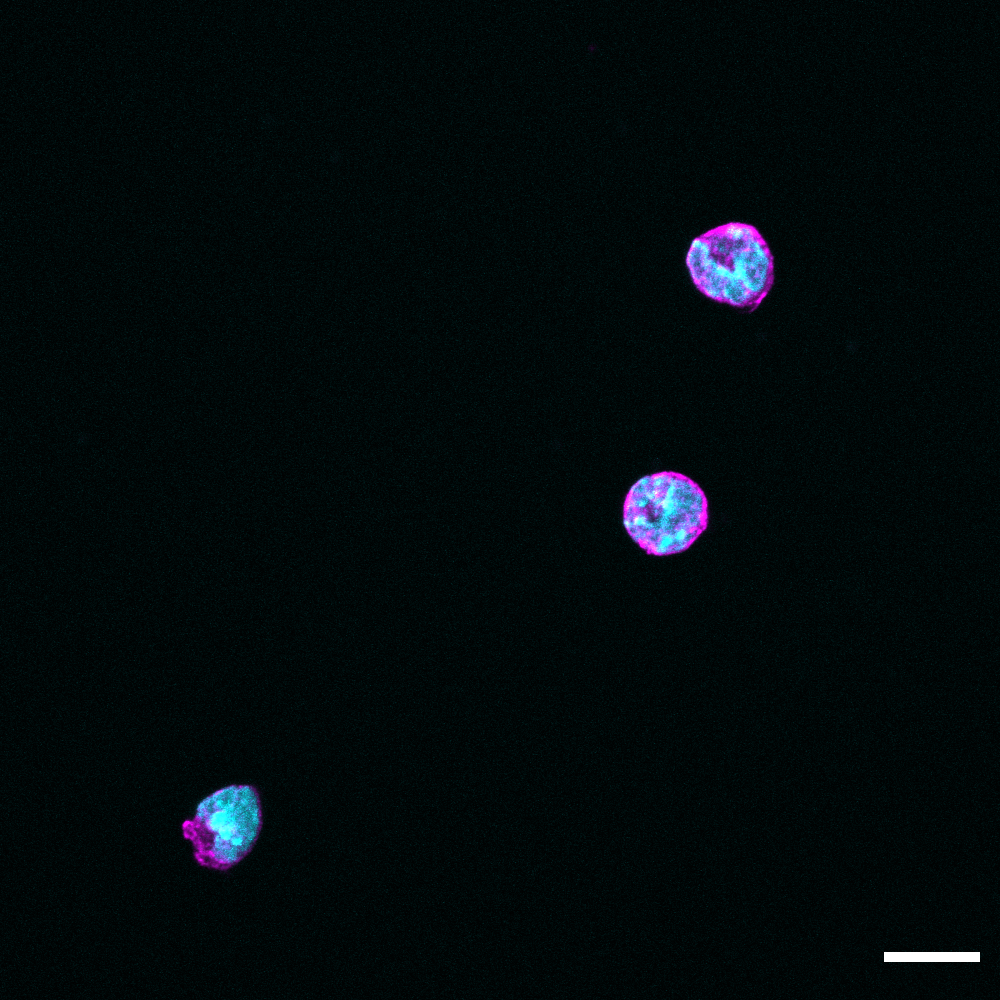

Supplement: Supplementary file 9 — EV Figures Source Data [file 44319_2024_150_MOESM9_ESM.zip › Figure EV1/Fig S1C/FB-176 WT Mice/unstim/como new.png]

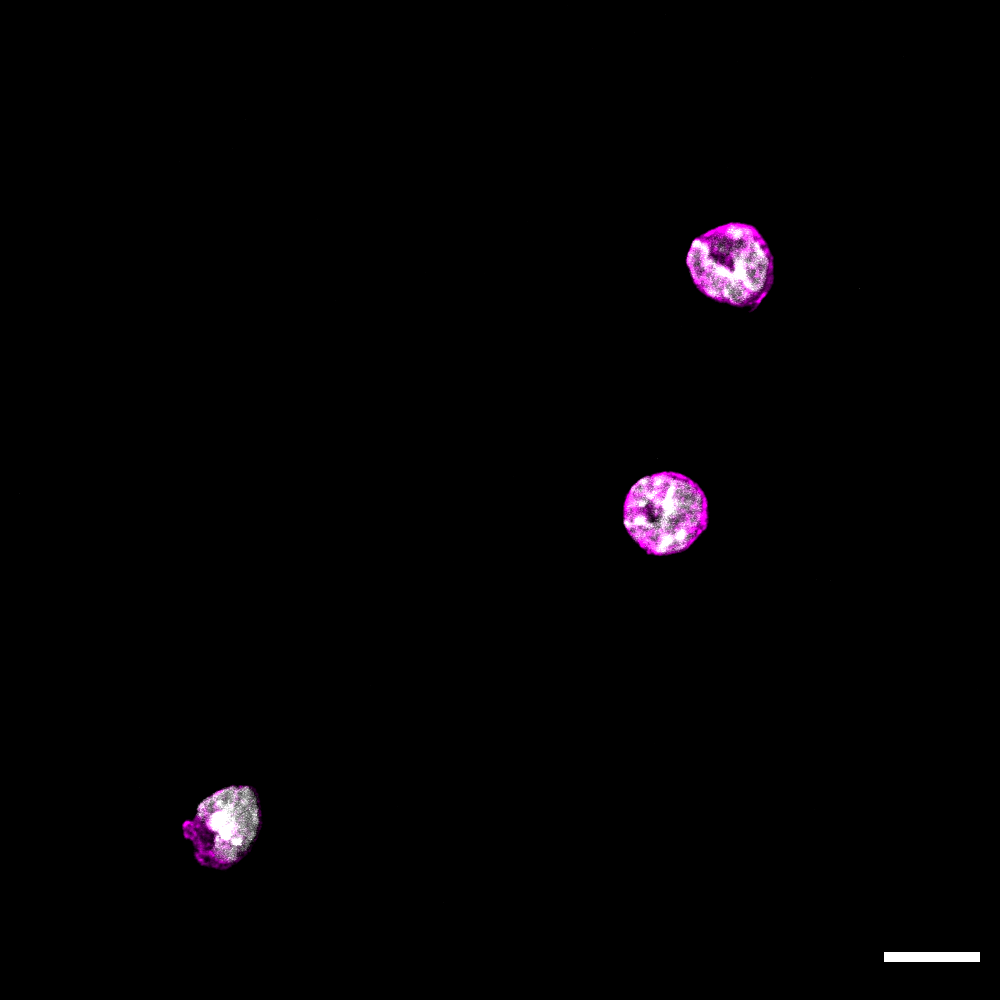

Supplement: Supplementary file 9 — EV Figures Source Data [file 44319_2024_150_MOESM9_ESM.zip › Figure EV1/Fig S1C/FB-176 WT Mice/unstim/comp gray.png]

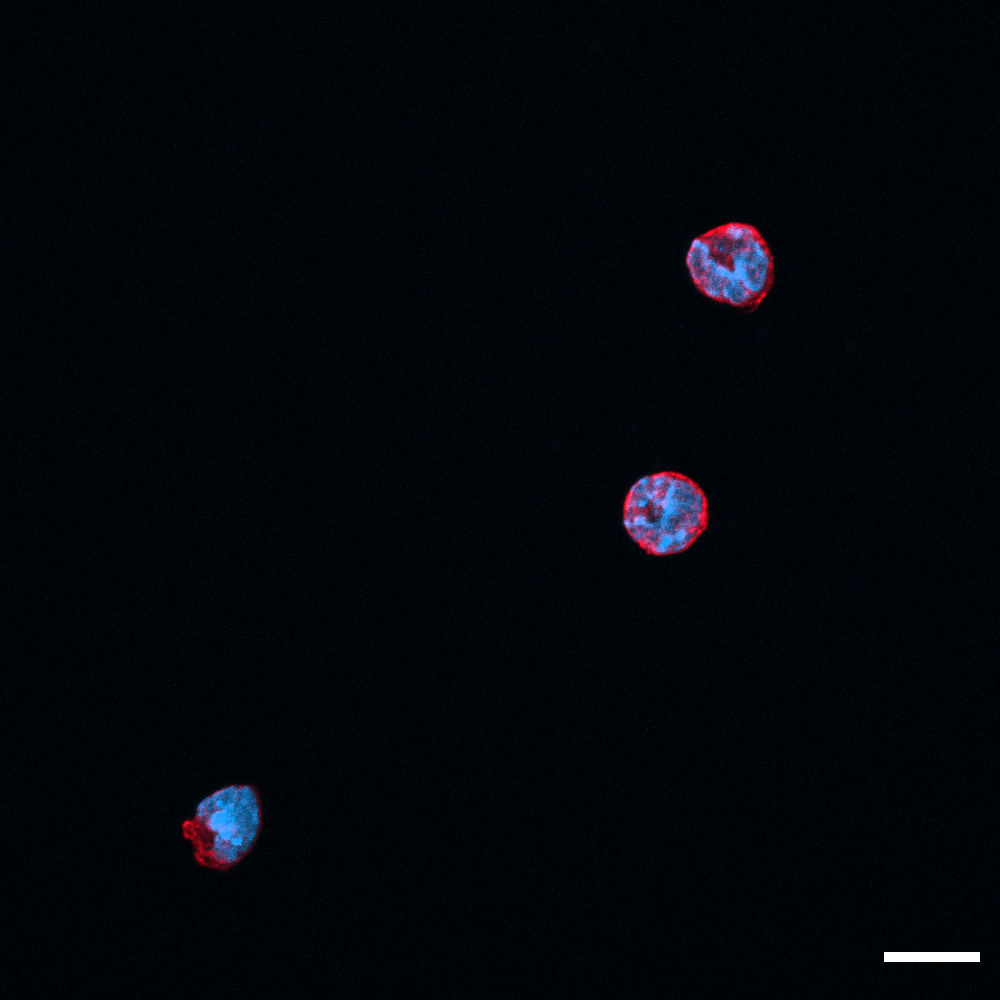

Supplement: Supplementary file 9 — EV Figures Source Data [file 44319_2024_150_MOESM9_ESM.zip › Figure EV1/Fig S1C/FB-176 WT Mice/unstimulated.png]

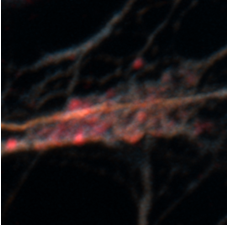

Supplement: Supplementary file 9 — EV Figures Source Data [file 44319_2024_150_MOESM9_ESM.zip › Figure EV1/Fig S1D/PMA-crop.tif]

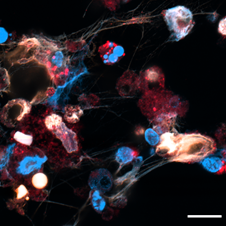

Supplement: Supplementary file 9 — EV Figures Source Data [file 44319_2024_150_MOESM9_ESM.zip › Figure EV1/Fig S1D/PMA.tif]

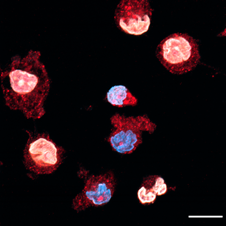

Supplement: Supplementary file 9 — EV Figures Source Data [file 44319_2024_150_MOESM9_ESM.zip › Figure EV1/Fig S1D/Unstim.tif]

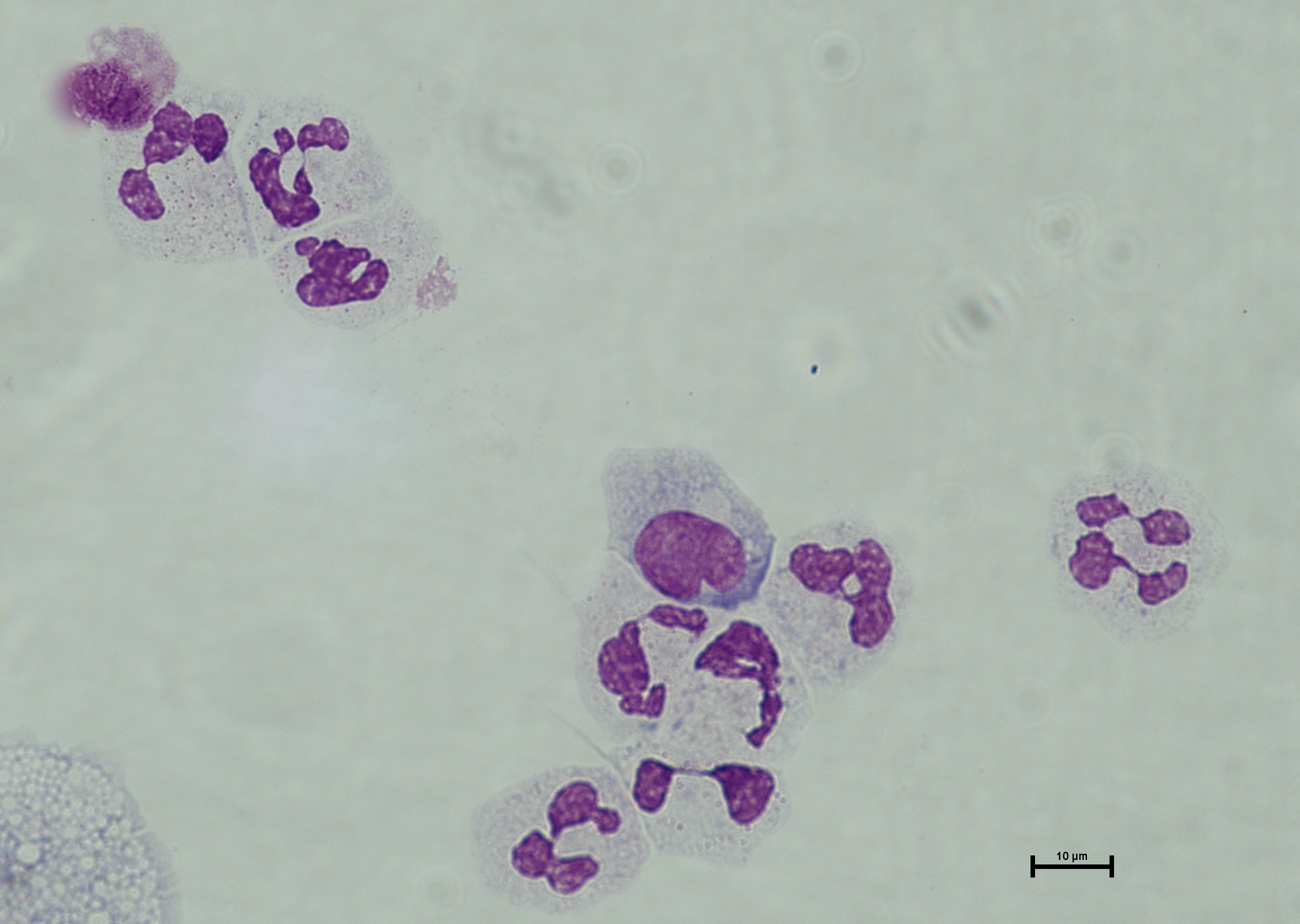

Supplement: Supplementary file 9 — EV Figures Source Data [file 44319_2024_150_MOESM9_ESM.zip › Figure EV1/Fig S1E/cytospin images with scale bar/01.03.2022 control_01.jpg]

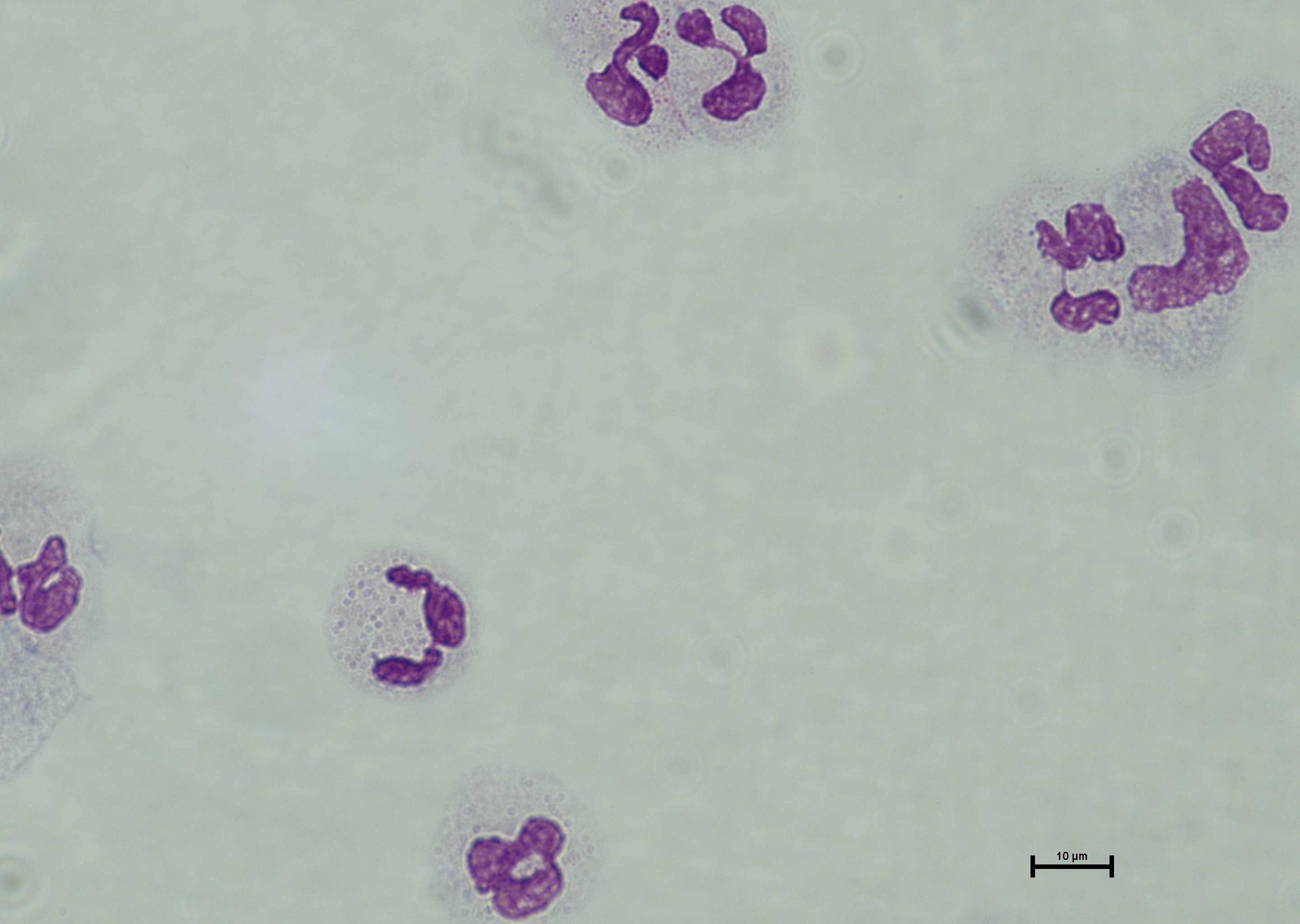

Supplement: Supplementary file 9 — EV Figures Source Data [file 44319_2024_150_MOESM9_ESM.zip › Figure EV1/Fig S1E/cytospin images with scale bar/01.03.2022 control_02.jpg]

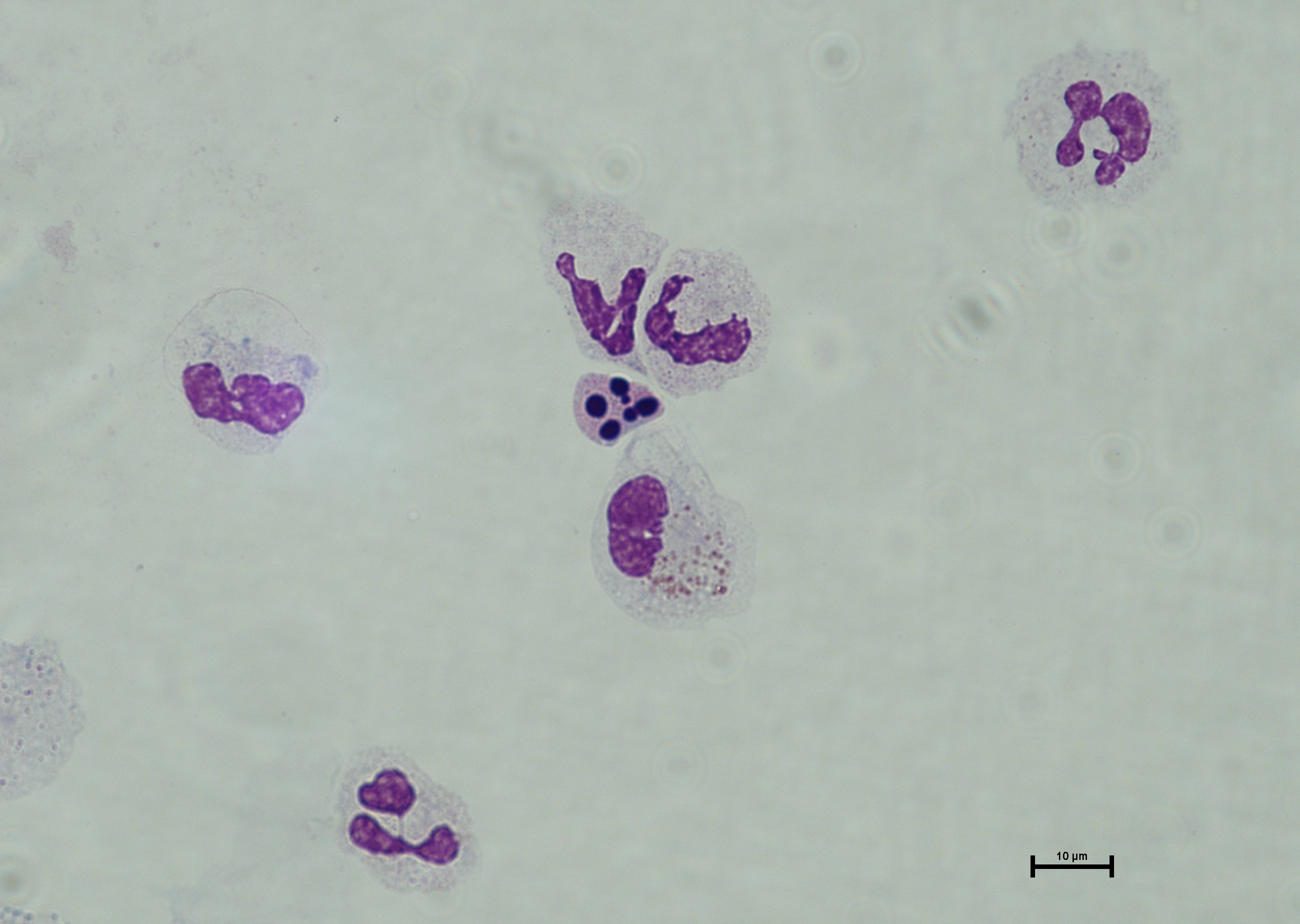

Supplement: Supplementary file 9 — EV Figures Source Data [file 44319_2024_150_MOESM9_ESM.zip › Figure EV1/Fig S1E/cytospin images with scale bar/01.03.2022 control_03.jpg]

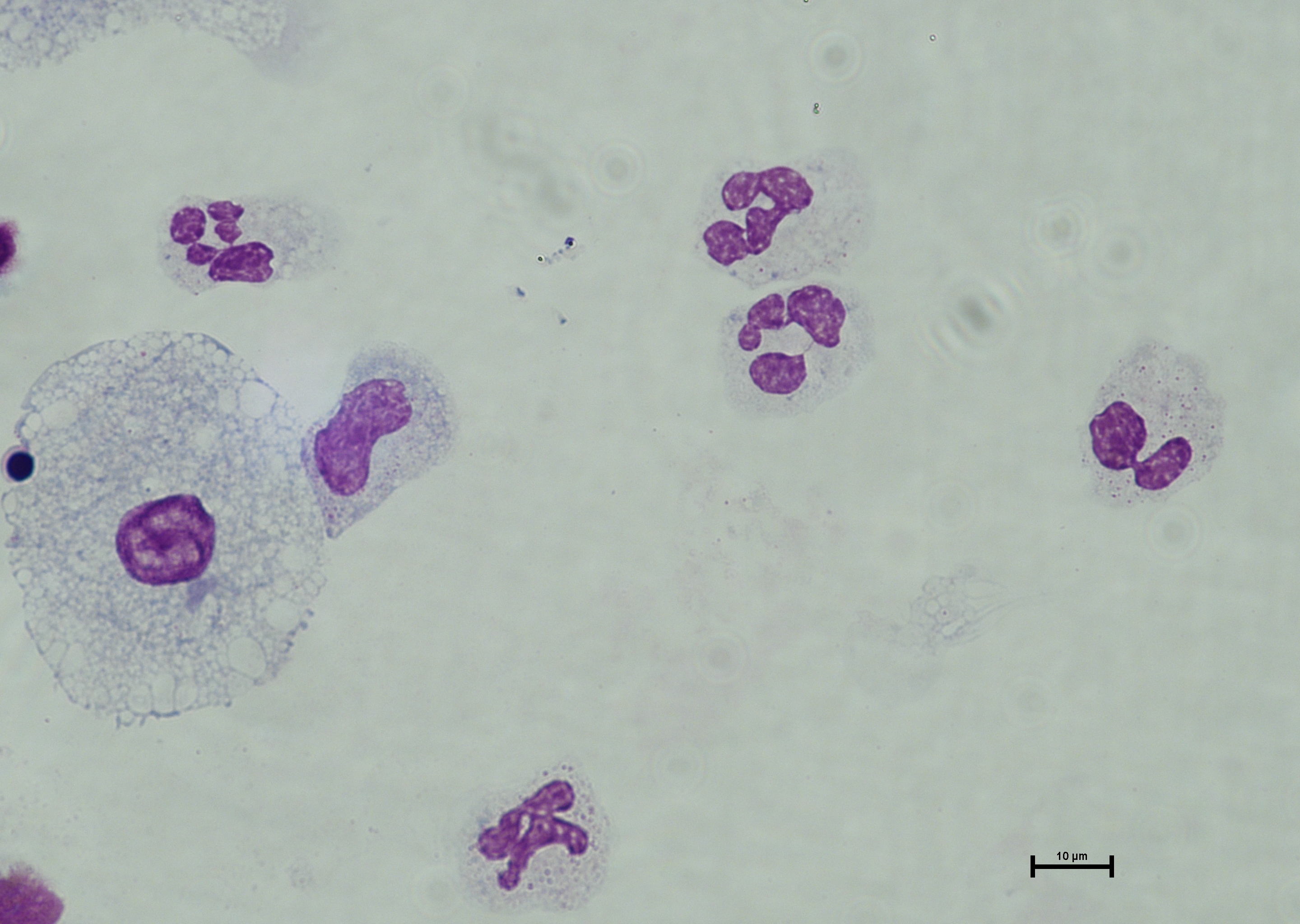

Supplement: Supplementary file 9 — EV Figures Source Data [file 44319_2024_150_MOESM9_ESM.zip › Figure EV1/Fig S1E/cytospin images with scale bar/01.03.2022 control_04.jpg]

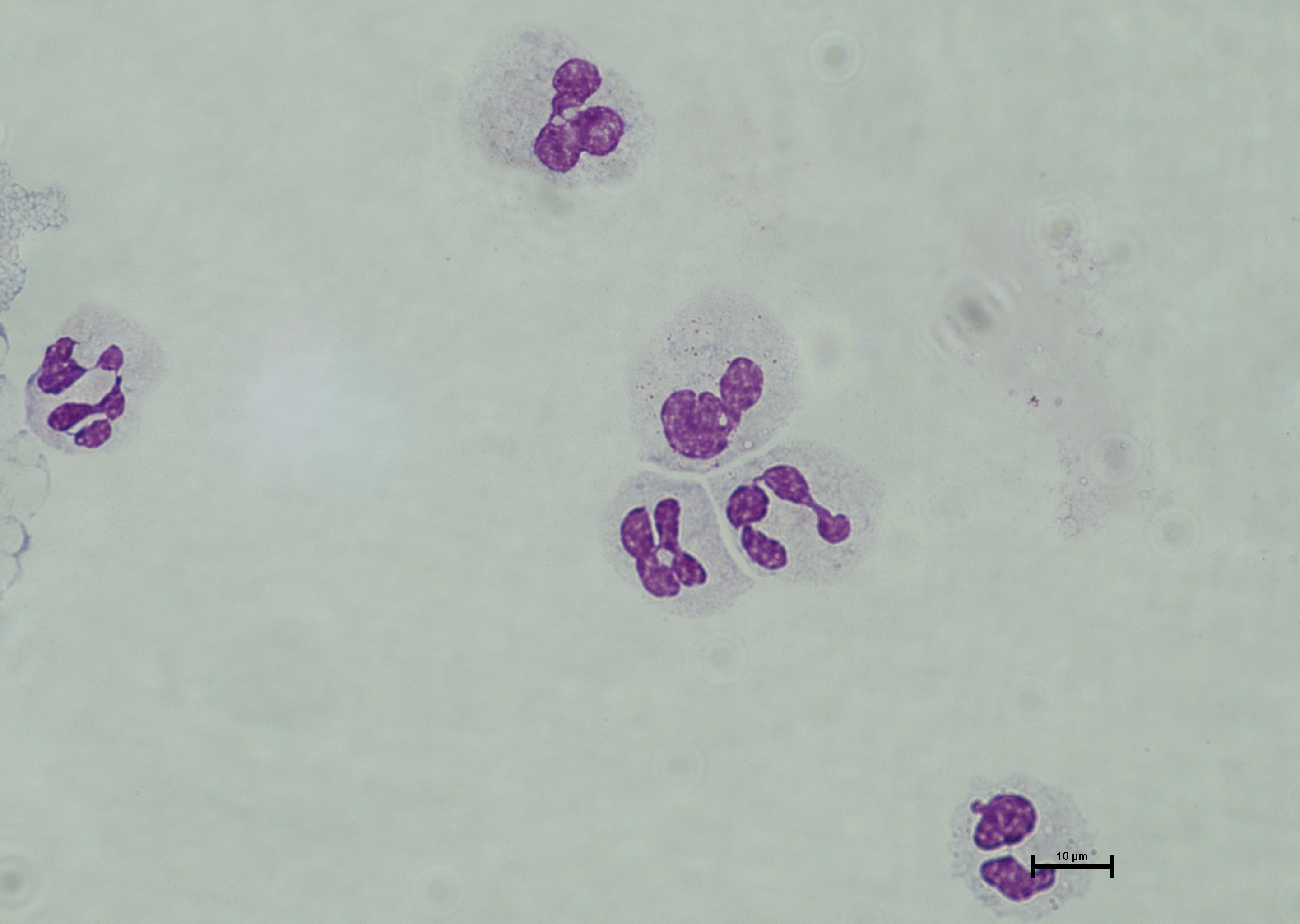

Supplement: Supplementary file 9 — EV Figures Source Data [file 44319_2024_150_MOESM9_ESM.zip › Figure EV1/Fig S1E/cytospin images with scale bar/01.03.2022 control_05.jpg]

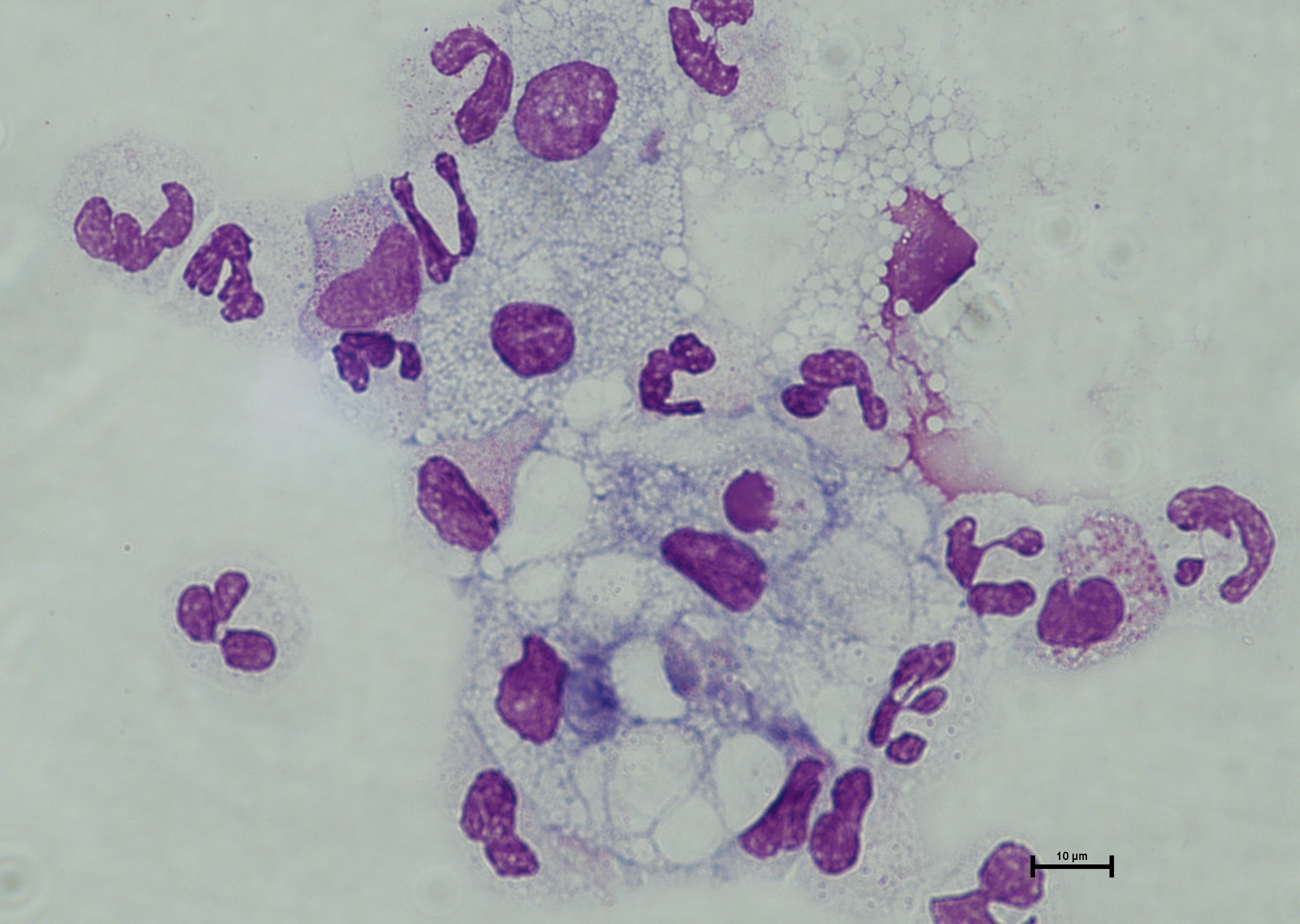

Supplement: Supplementary file 9 — EV Figures Source Data [file 44319_2024_150_MOESM9_ESM.zip › Figure EV1/Fig S1E/cytospin images with scale bar/01.03.2022 control_06.jpg]

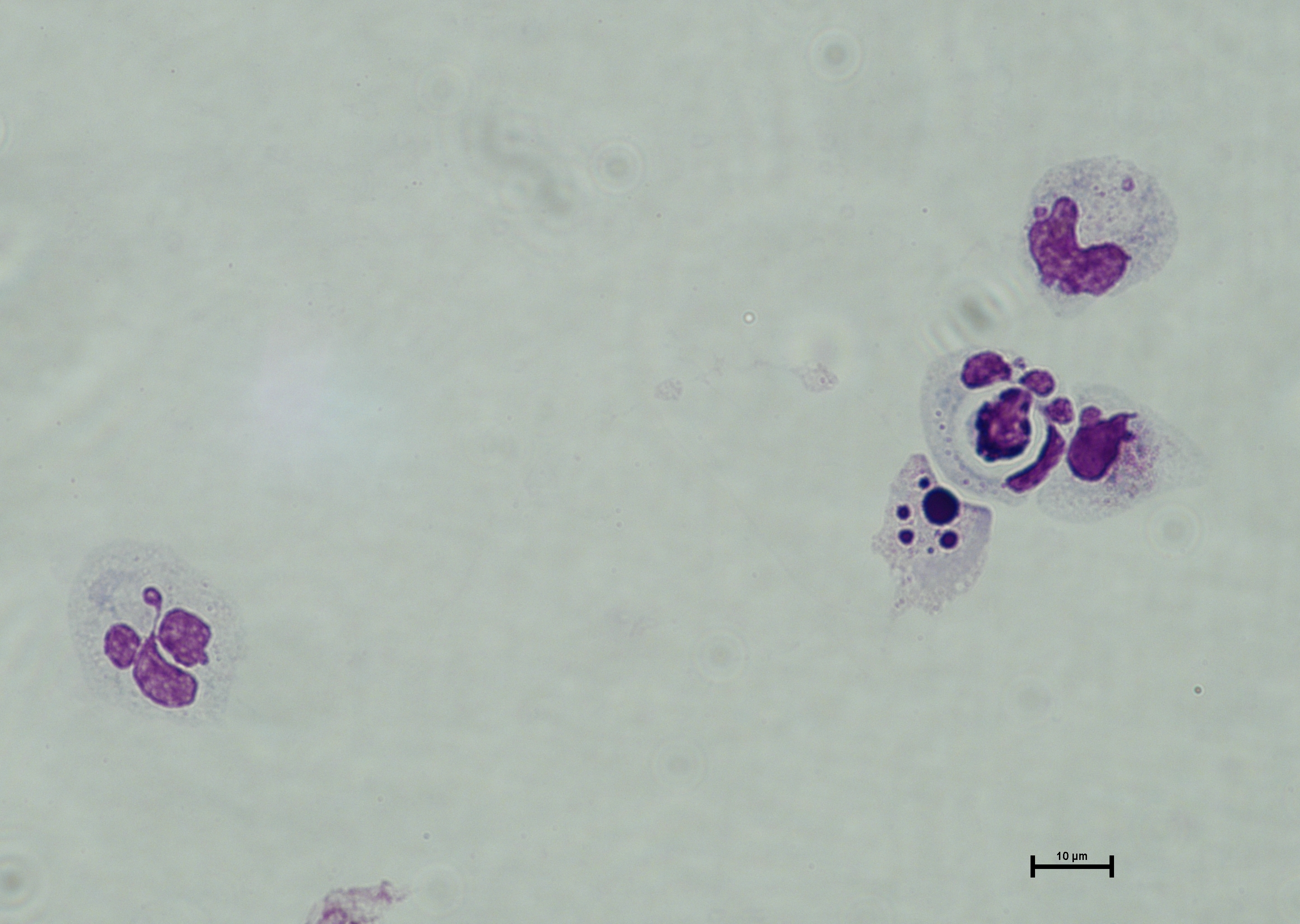

Supplement: Supplementary file 9 — EV Figures Source Data [file 44319_2024_150_MOESM9_ESM.zip › Figure EV1/Fig S1E/cytospin images with scale bar/01.03.2022 treat_01.jpg]

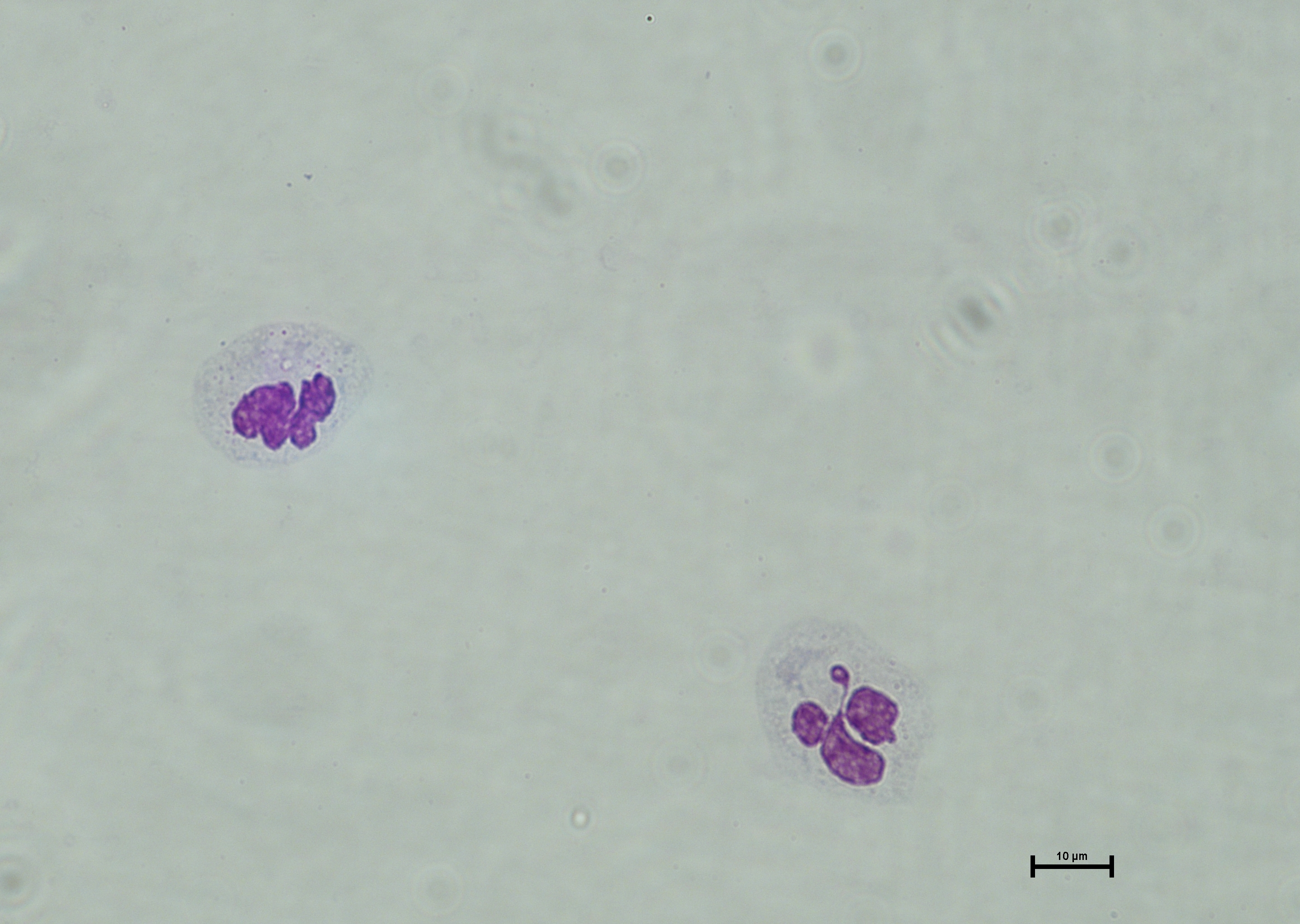

Supplement: Supplementary file 9 — EV Figures Source Data [file 44319_2024_150_MOESM9_ESM.zip › Figure EV1/Fig S1E/cytospin images with scale bar/01.03.2022 treat_02.jpg]

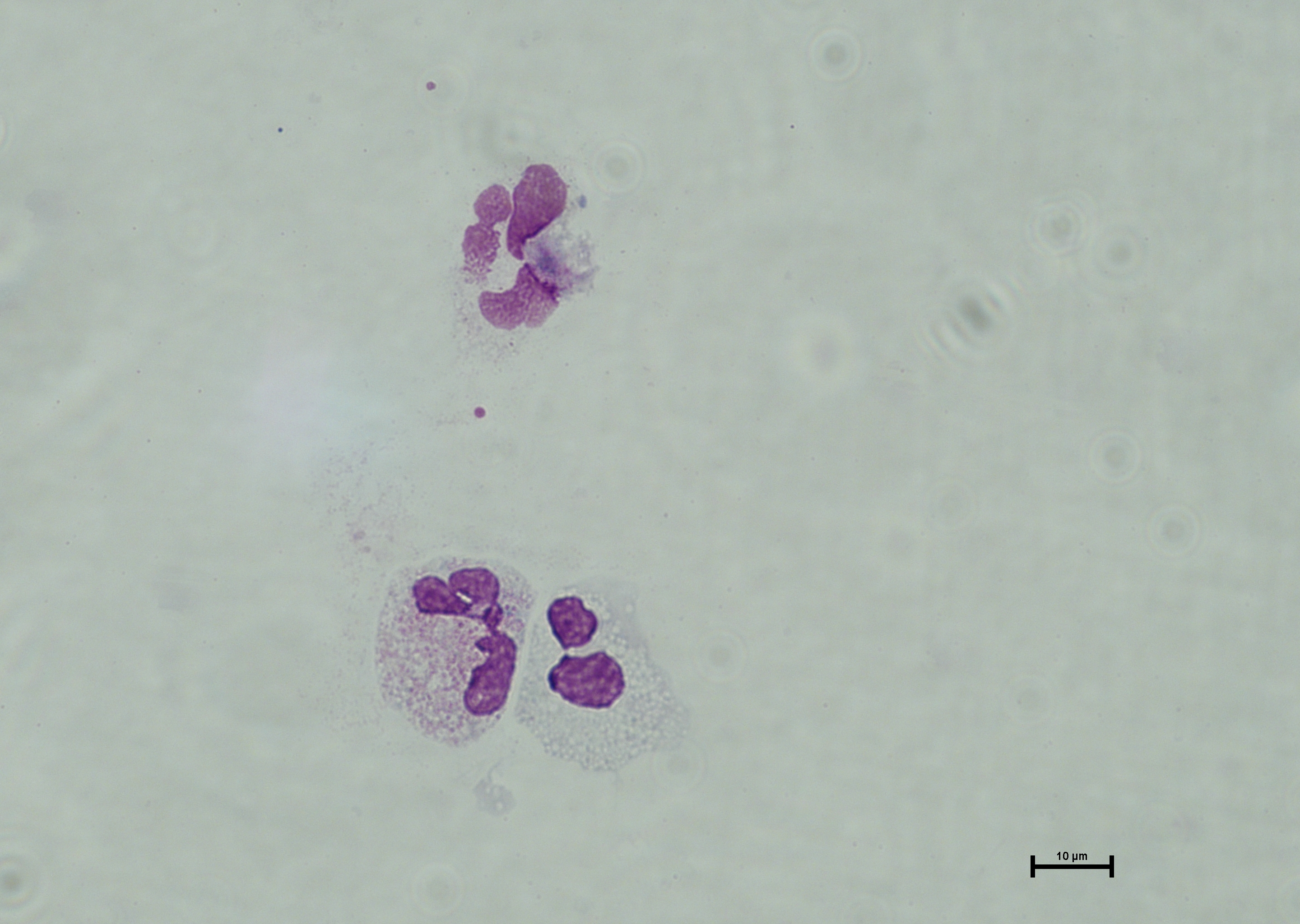

Supplement: Supplementary file 9 — EV Figures Source Data [file 44319_2024_150_MOESM9_ESM.zip › Figure EV1/Fig S1E/cytospin images with scale bar/01.03.2022 treat_03.jpg]

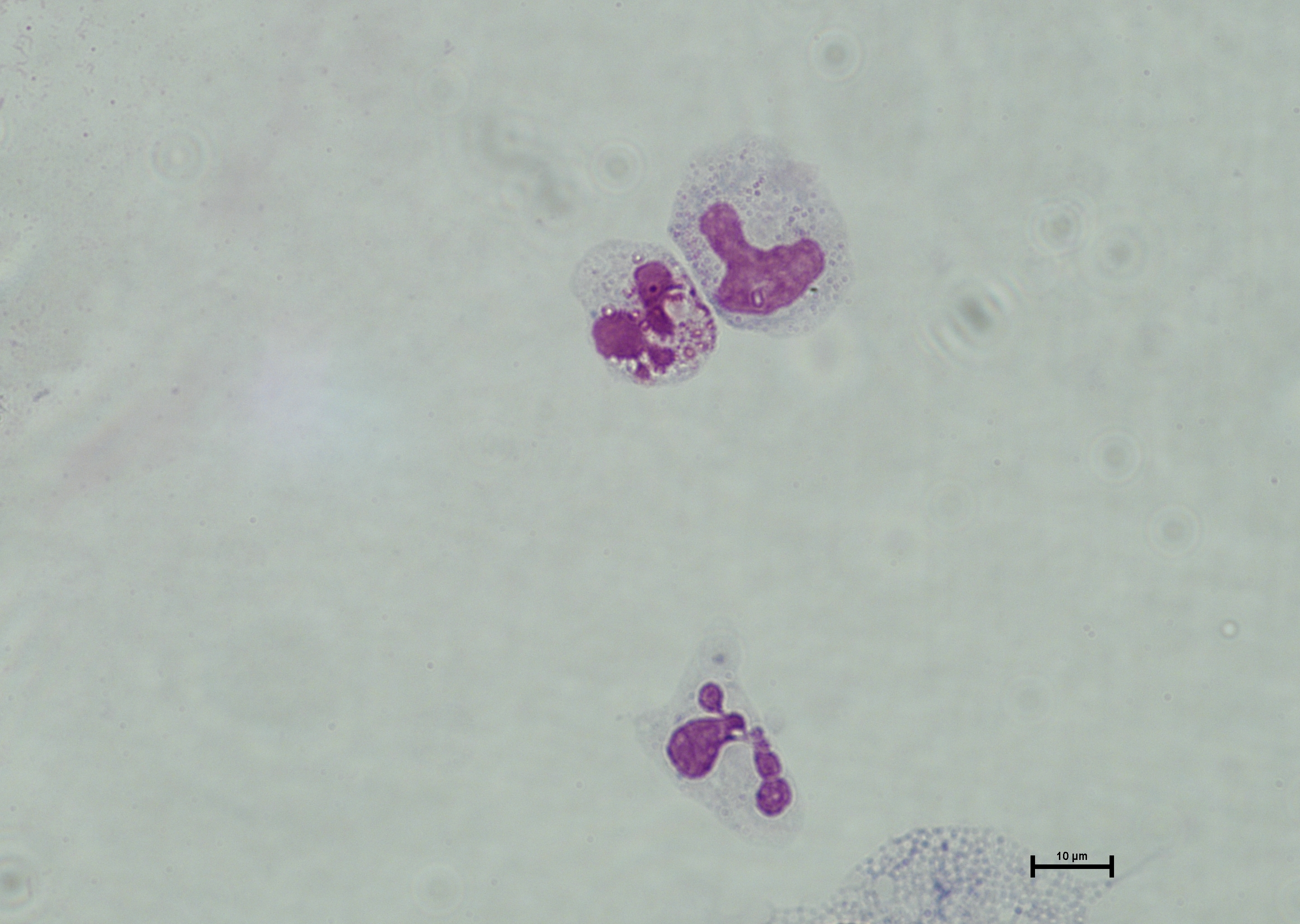

Supplement: Supplementary file 9 — EV Figures Source Data [file 44319_2024_150_MOESM9_ESM.zip › Figure EV1/Fig S1E/cytospin images with scale bar/01.03.2022 treat_04.jpg]

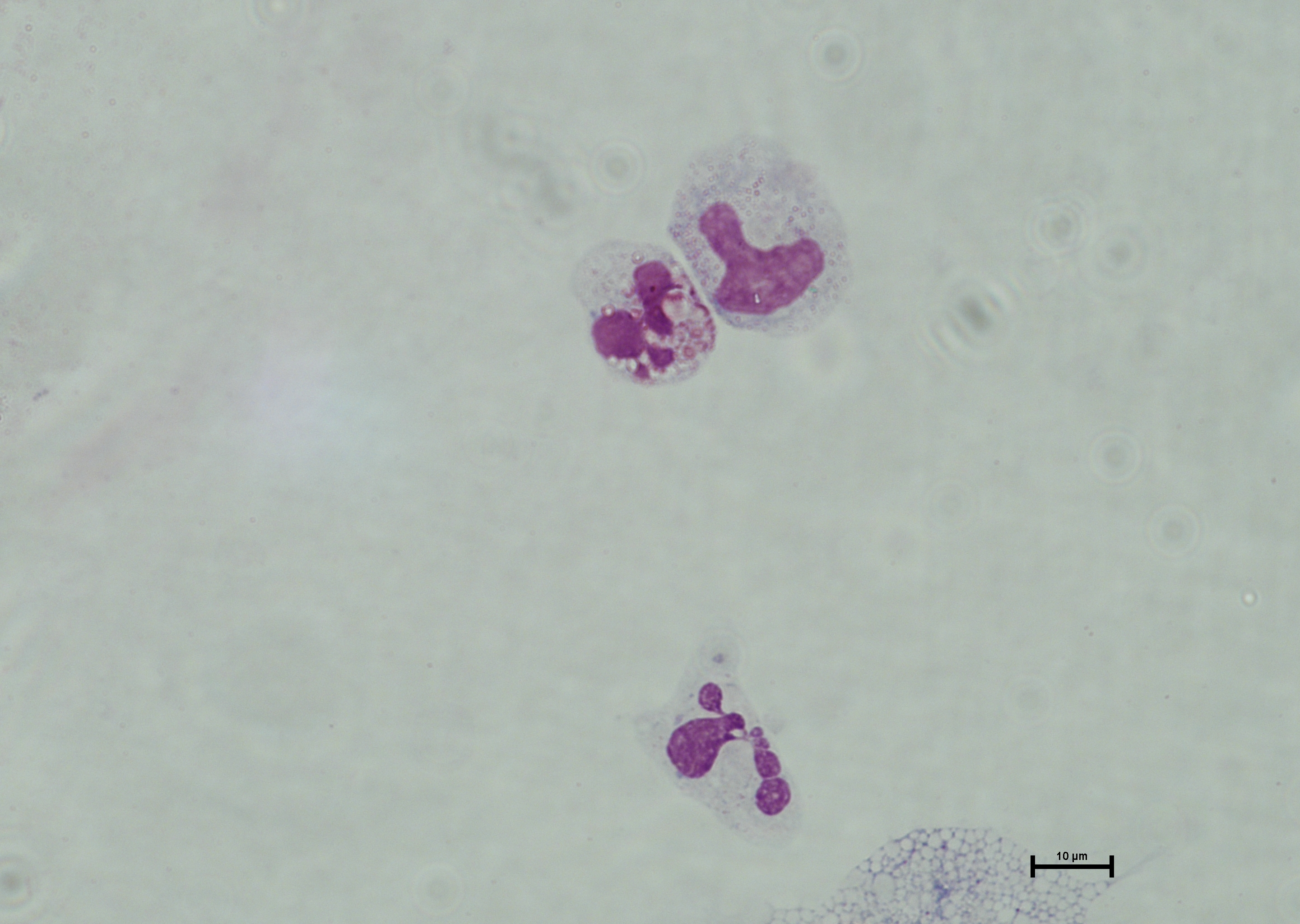

Supplement: Supplementary file 9 — EV Figures Source Data [file 44319_2024_150_MOESM9_ESM.zip › Figure EV1/Fig S1E/cytospin images with scale bar/01.03.2022 treat_05.jpg]

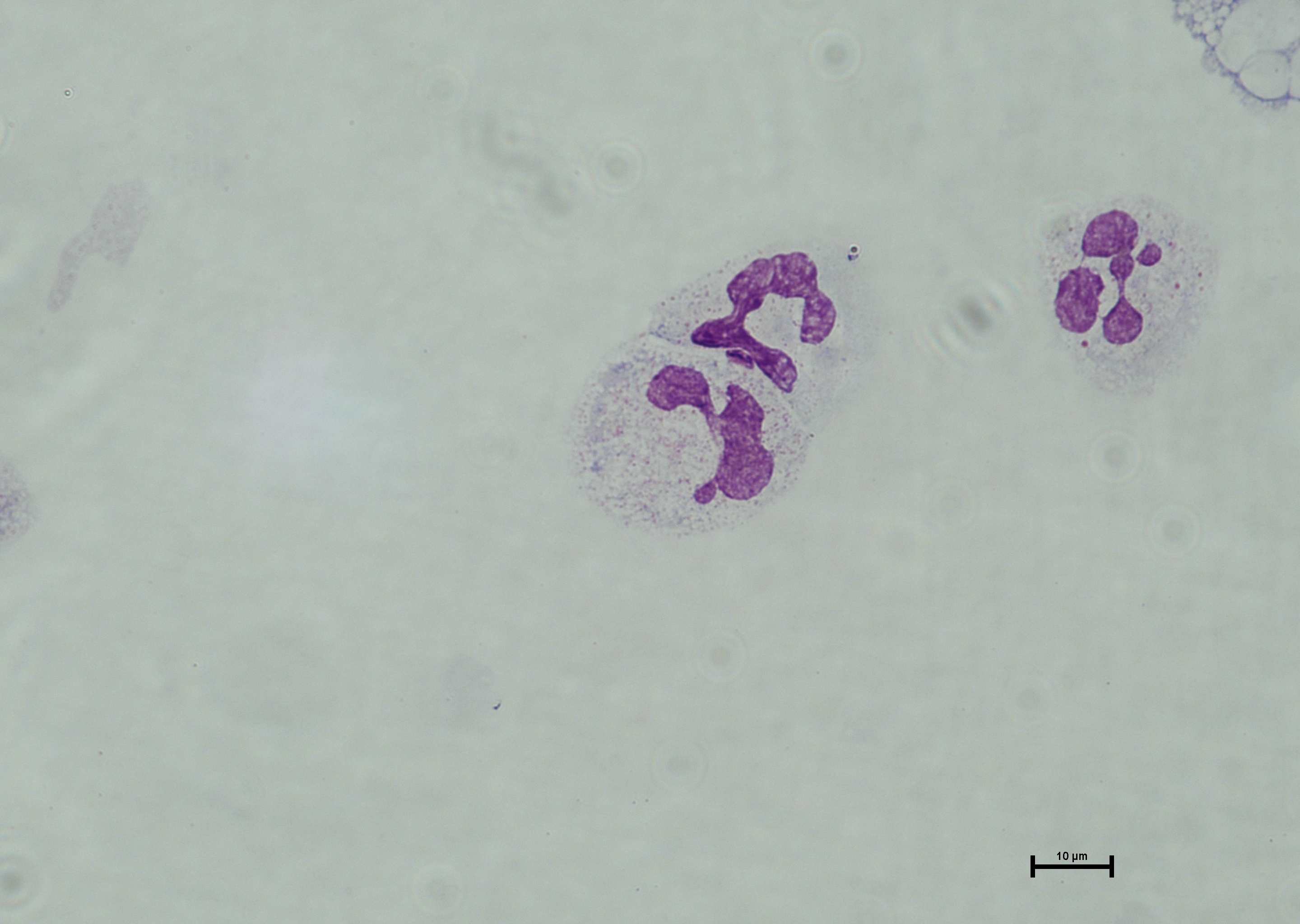

Supplement: Supplementary file 9 — EV Figures Source Data [file 44319_2024_150_MOESM9_ESM.zip › Figure EV1/Fig S1E/cytospin images with scale bar/01.03.2022 treat_06.jpg]

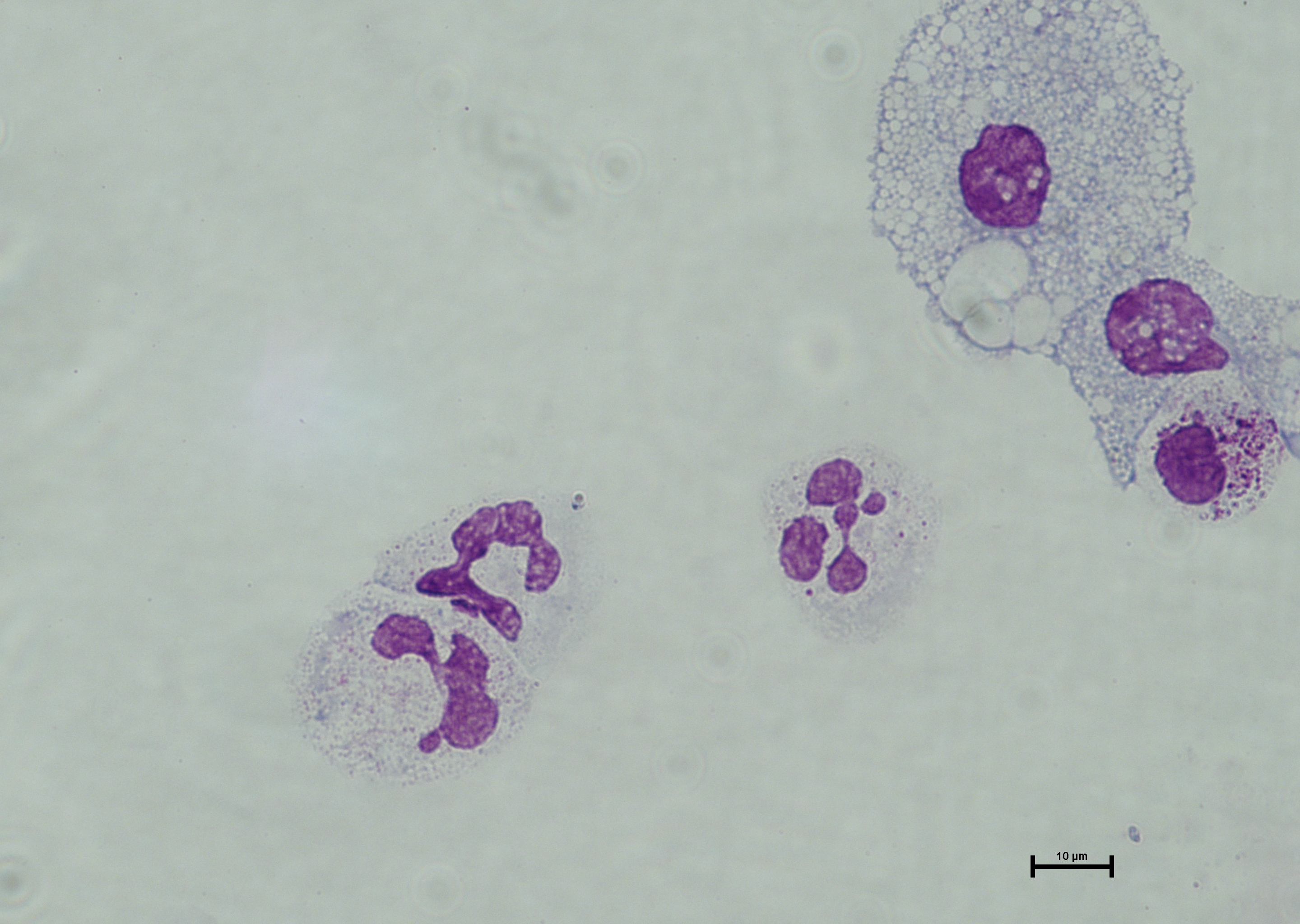

Supplement: Supplementary file 9 — EV Figures Source Data [file 44319_2024_150_MOESM9_ESM.zip › Figure EV1/Fig S1E/cytospin images with scale bar/01.03.2022 treat_07.jpg]

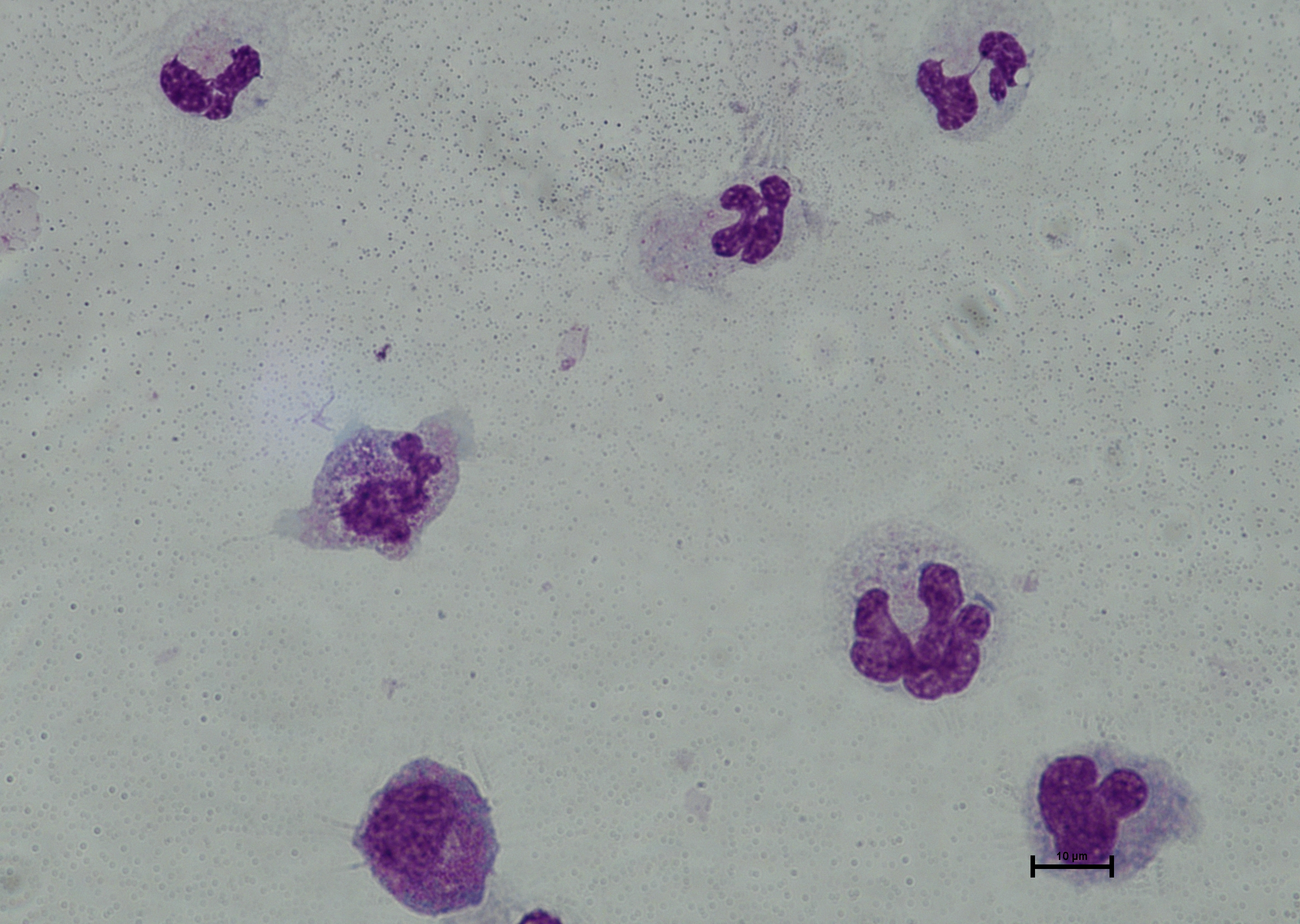

Supplement: Supplementary file 9 — EV Figures Source Data [file 44319_2024_150_MOESM9_ESM.zip › Figure EV1/Fig S1E/cytospin images with scale bar/10.11.2021 control_01.jpg]

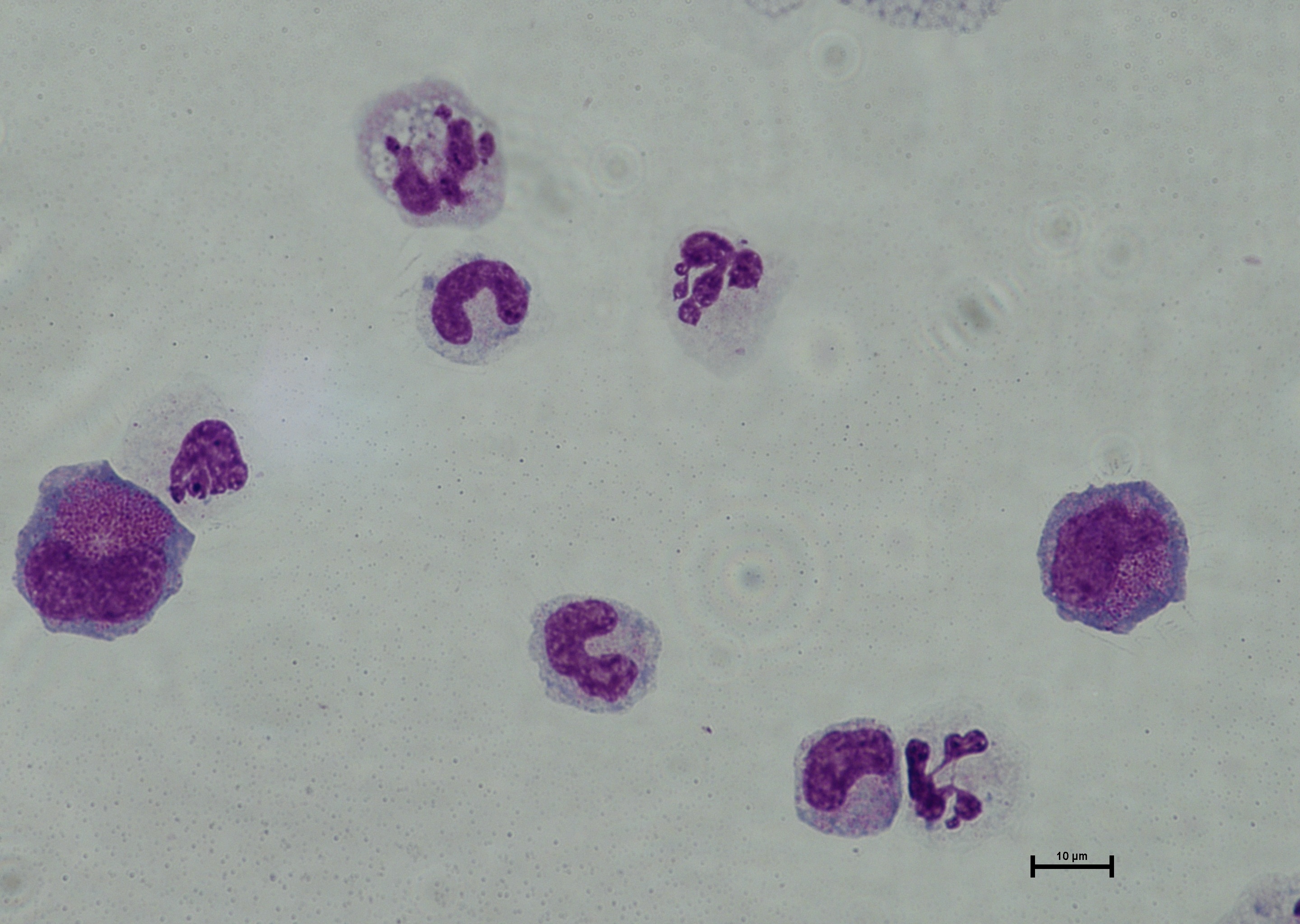

Supplement: Supplementary file 9 — EV Figures Source Data [file 44319_2024_150_MOESM9_ESM.zip › Figure EV1/Fig S1E/cytospin images with scale bar/10.11.2021 control_02.jpg]

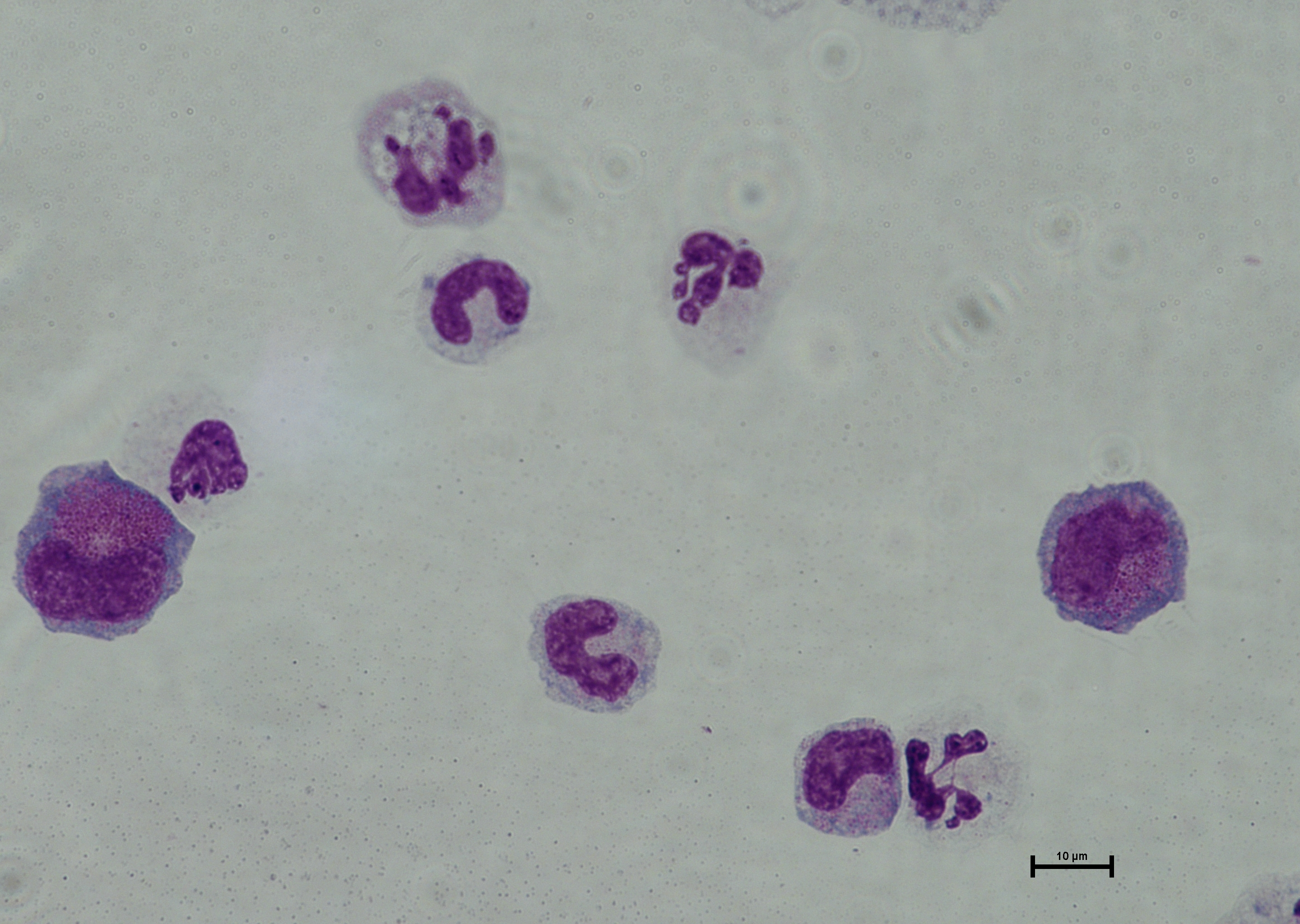

Supplement: Supplementary file 9 — EV Figures Source Data [file 44319_2024_150_MOESM9_ESM.zip › Figure EV1/Fig S1E/cytospin images with scale bar/10.11.2021 control_03.jpg]

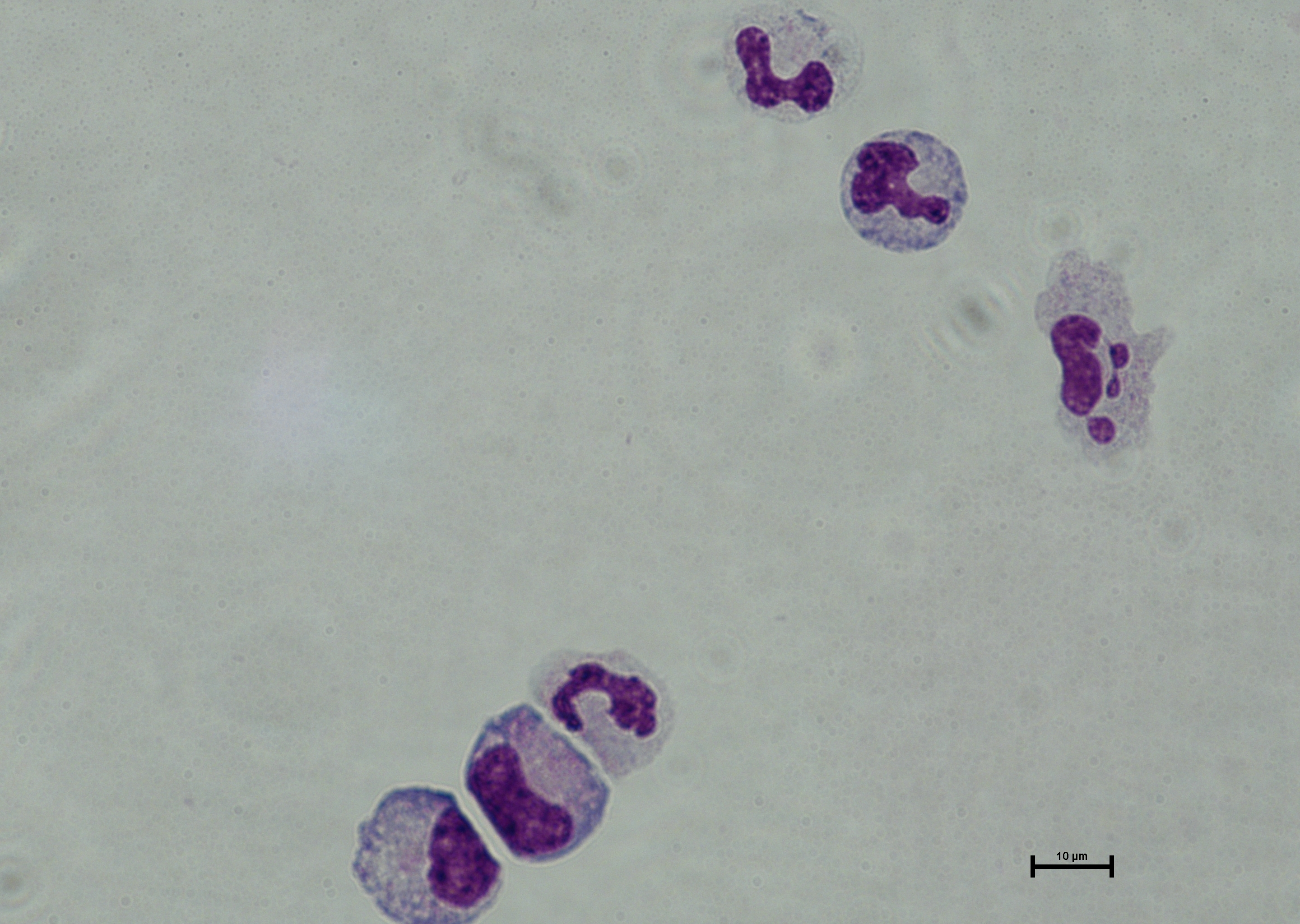

Supplement: Supplementary file 9 — EV Figures Source Data [file 44319_2024_150_MOESM9_ESM.zip › Figure EV1/Fig S1E/cytospin images with scale bar/10.11.2021 control_04.jpg]

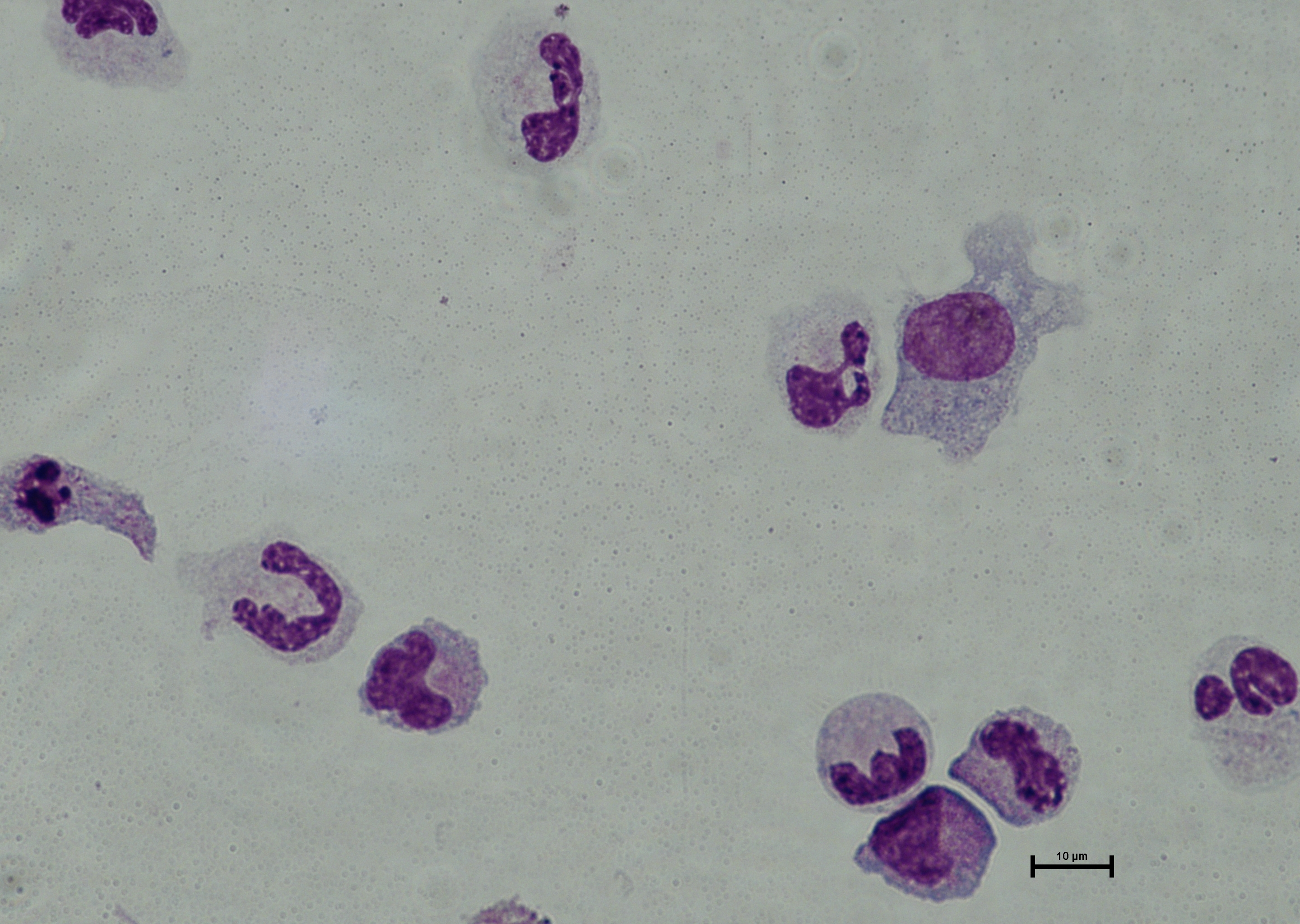

Supplement: Supplementary file 9 — EV Figures Source Data [file 44319_2024_150_MOESM9_ESM.zip › Figure EV1/Fig S1E/cytospin images with scale bar/10.11.2021 control_05.jpg]

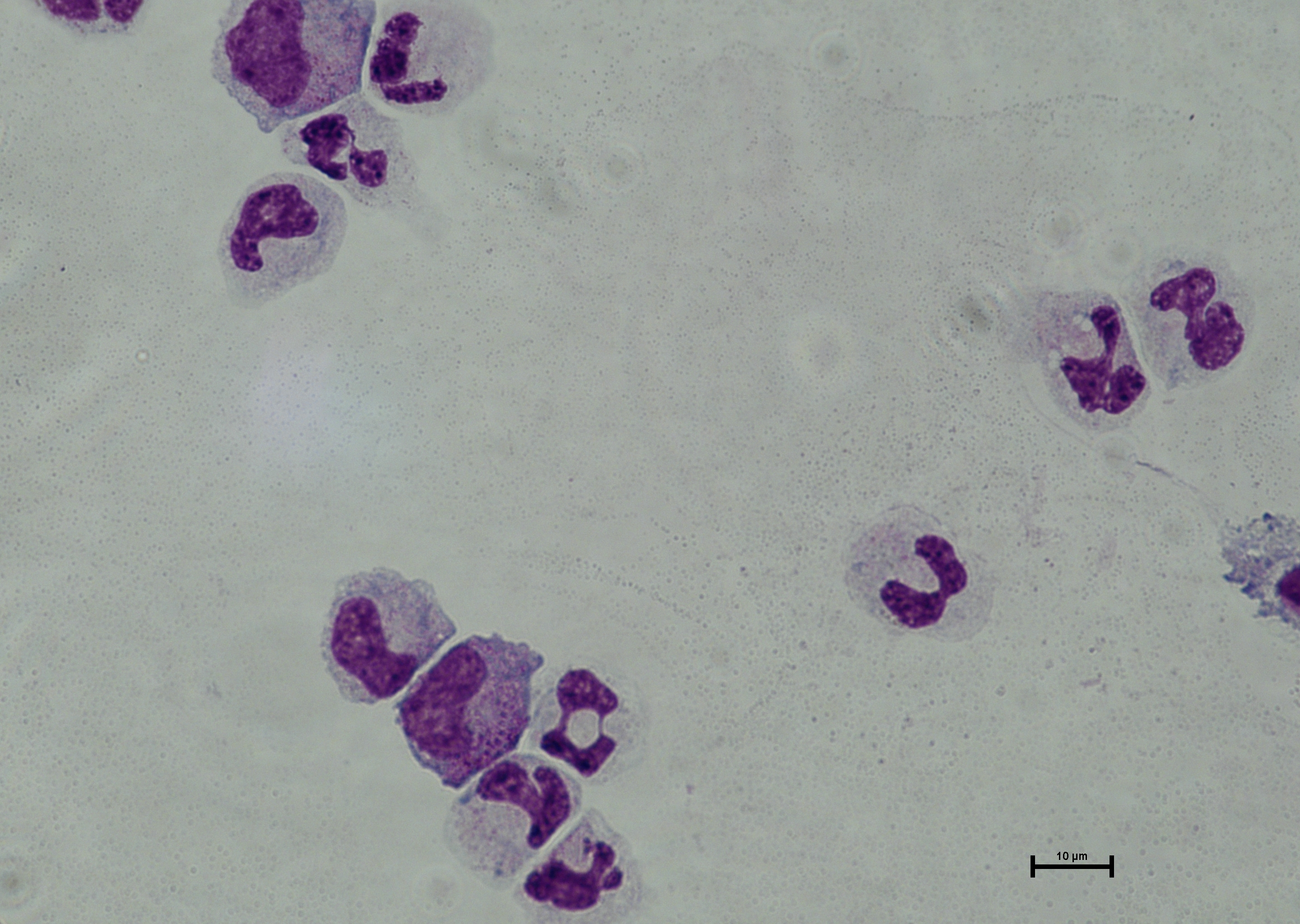

Supplement: Supplementary file 9 — EV Figures Source Data [file 44319_2024_150_MOESM9_ESM.zip › Figure EV1/Fig S1E/cytospin images with scale bar/10.11.2021 control_06.jpg]

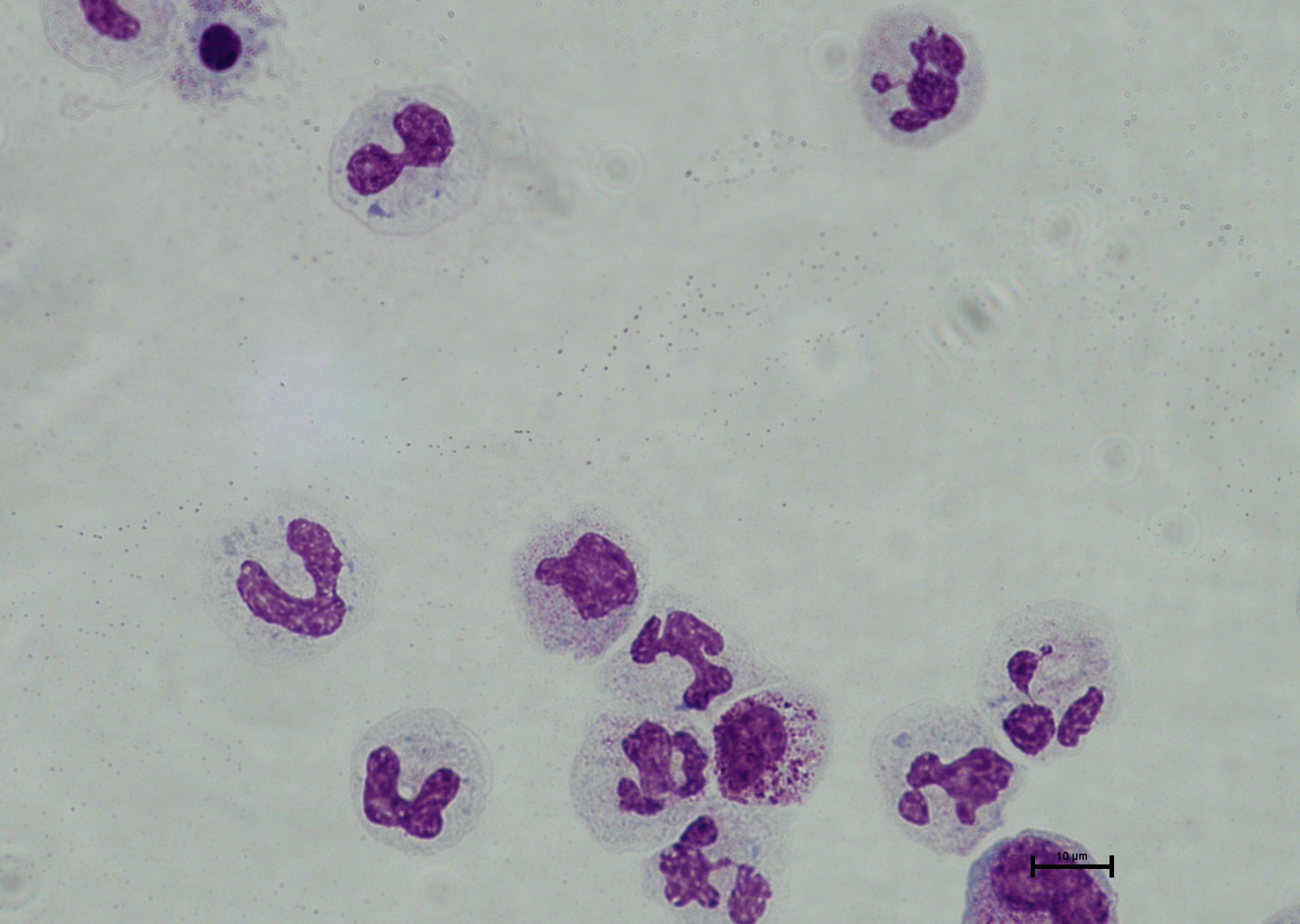

Supplement: Supplementary file 9 — EV Figures Source Data [file 44319_2024_150_MOESM9_ESM.zip › Figure EV1/Fig S1E/cytospin images with scale bar/10.11.2021 treat_01.jpg]

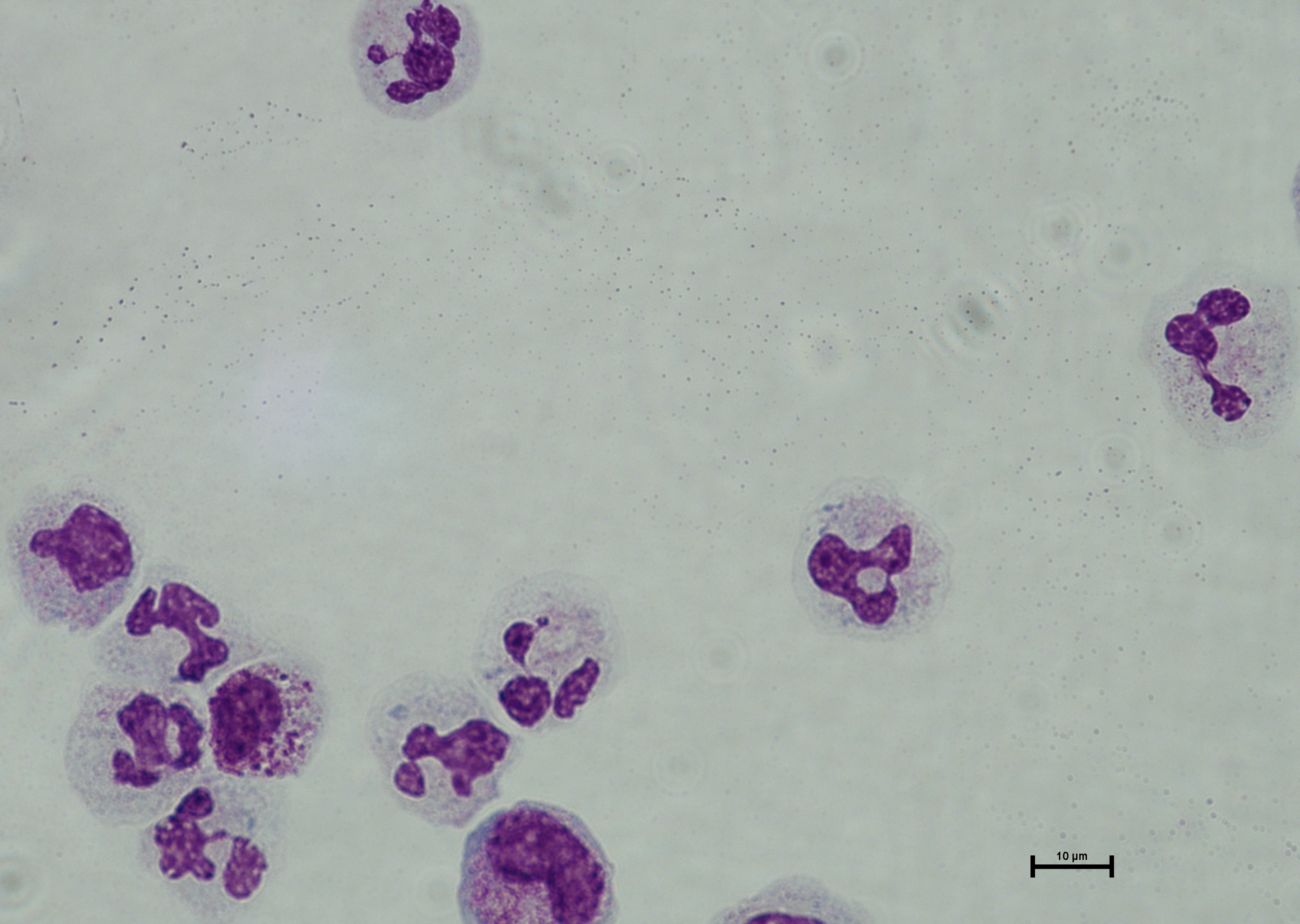

Supplement: Supplementary file 9 — EV Figures Source Data [file 44319_2024_150_MOESM9_ESM.zip › Figure EV1/Fig S1E/cytospin images with scale bar/10.11.2021 treat_02.jpg]

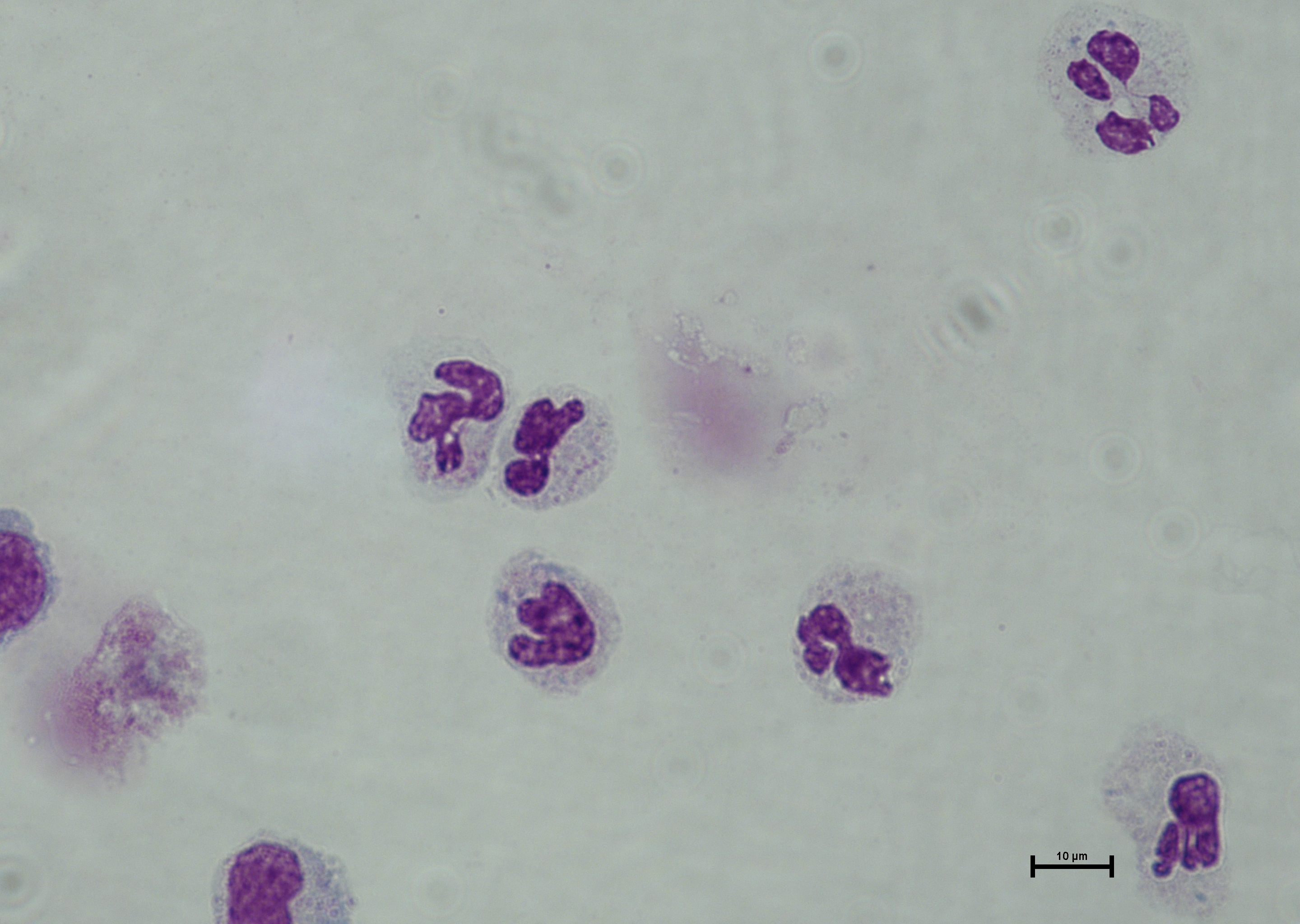

Supplement: Supplementary file 9 — EV Figures Source Data [file 44319_2024_150_MOESM9_ESM.zip › Figure EV1/Fig S1E/cytospin images with scale bar/10.11.2021 treat_03.jpg]

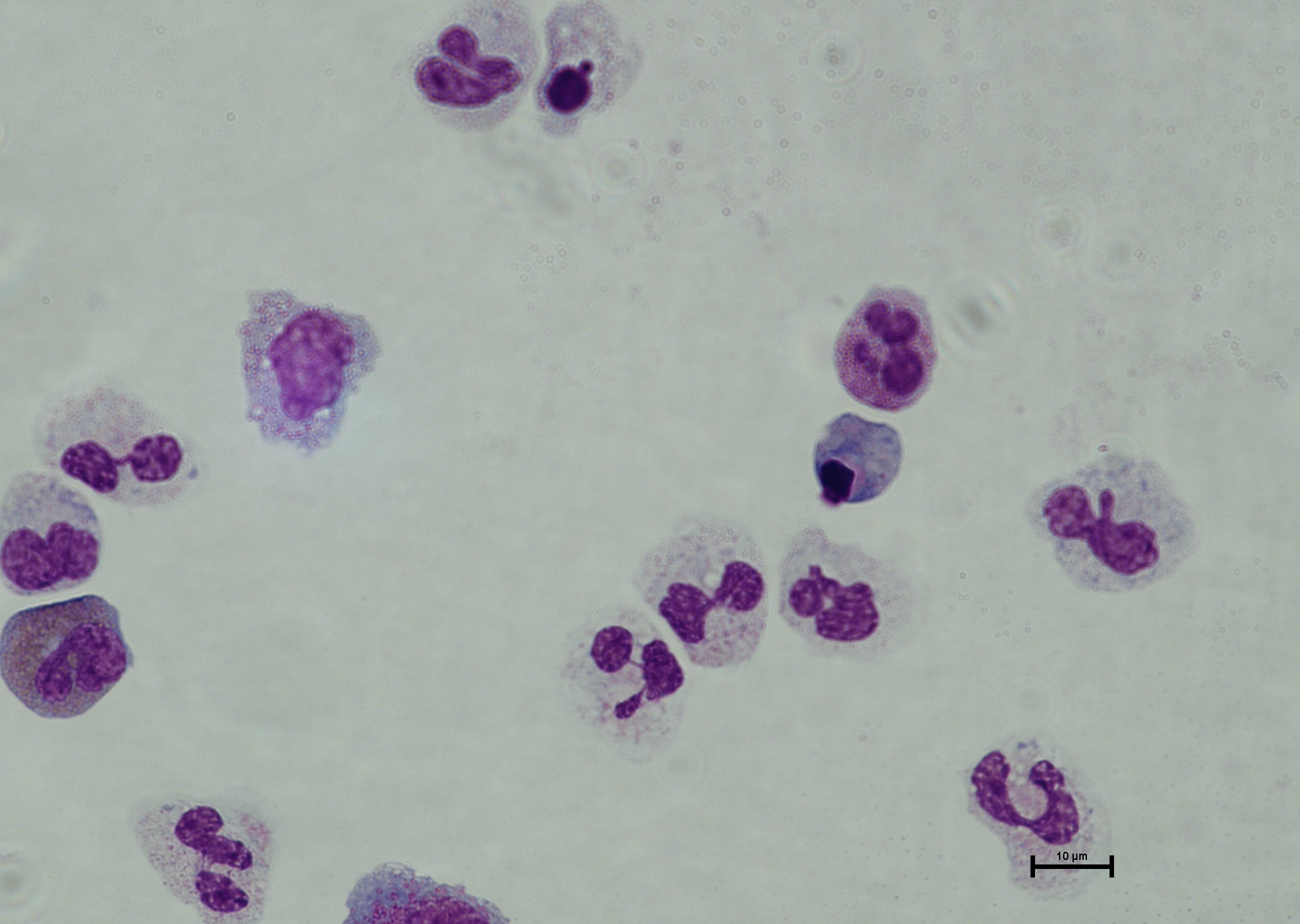

Supplement: Supplementary file 9 — EV Figures Source Data [file 44319_2024_150_MOESM9_ESM.zip › Figure EV1/Fig S1E/cytospin images with scale bar/10.11.2021 treat_04.jpg]

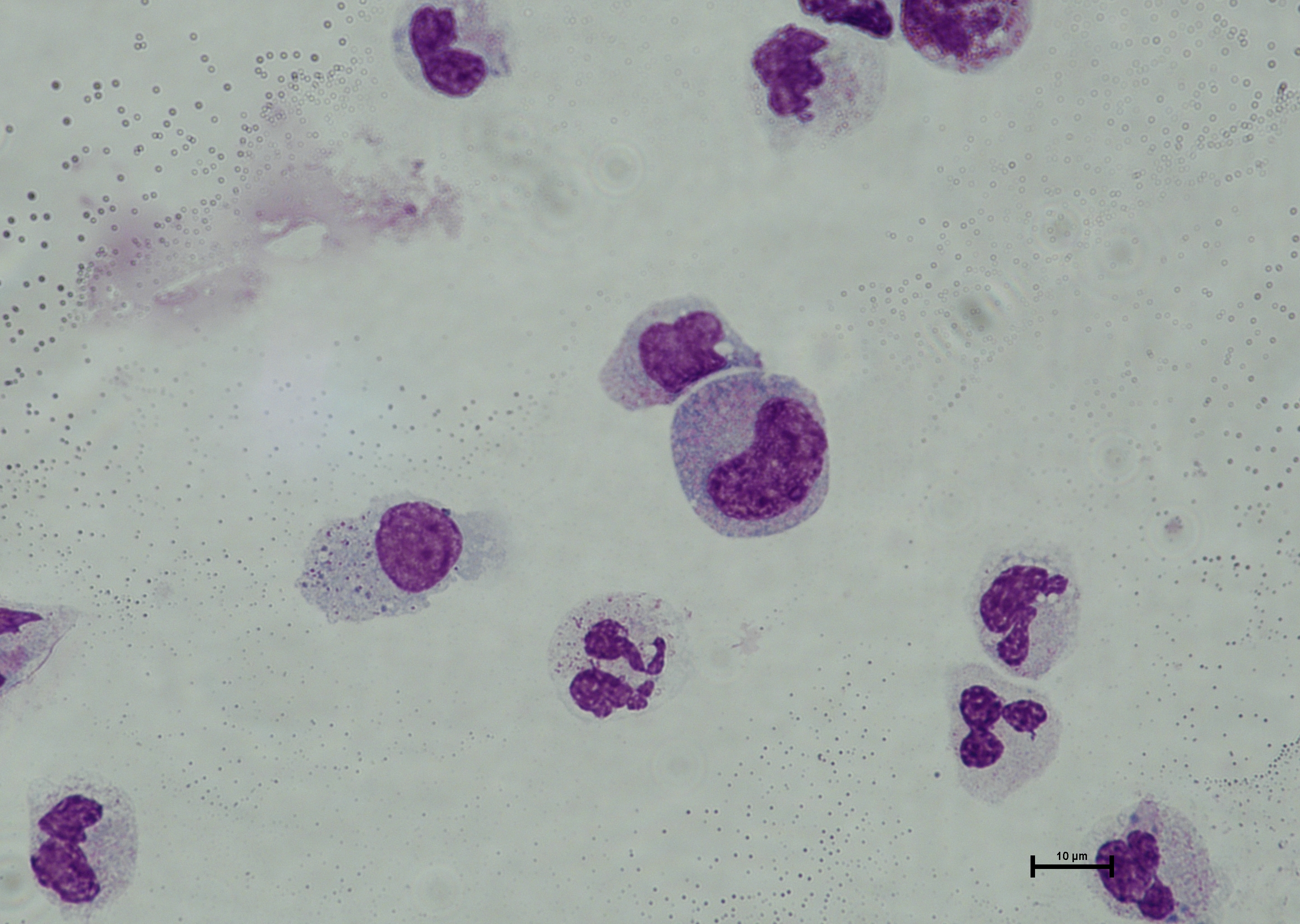

Supplement: Supplementary file 9 — EV Figures Source Data [file 44319_2024_150_MOESM9_ESM.zip › Figure EV1/Fig S1E/cytospin images with scale bar/10.11.2021 treat_05.jpg]

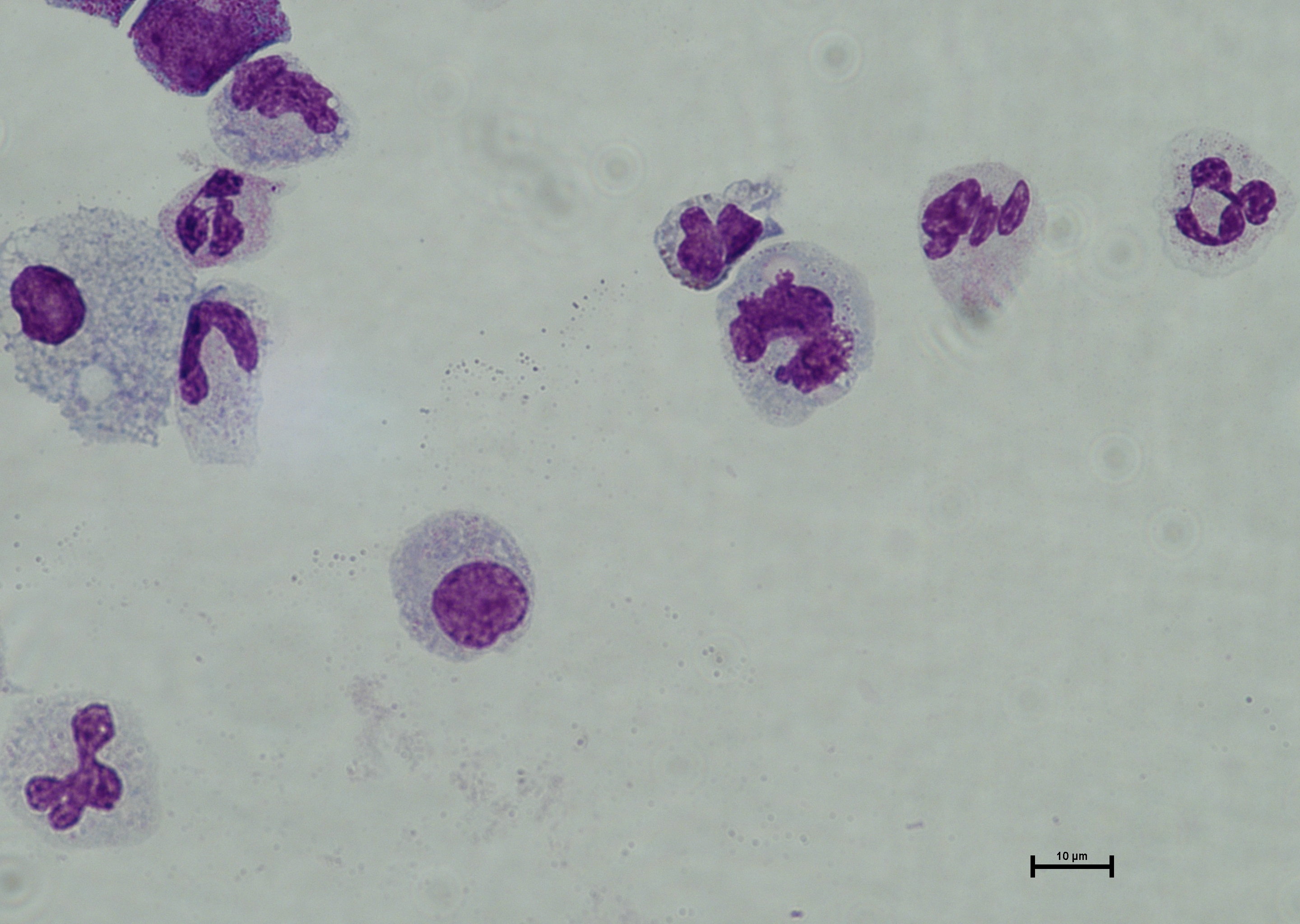

Supplement: Supplementary file 9 — EV Figures Source Data [file 44319_2024_150_MOESM9_ESM.zip › Figure EV1/Fig S1E/cytospin images with scale bar/10.11.2021 treat_06.jpg]

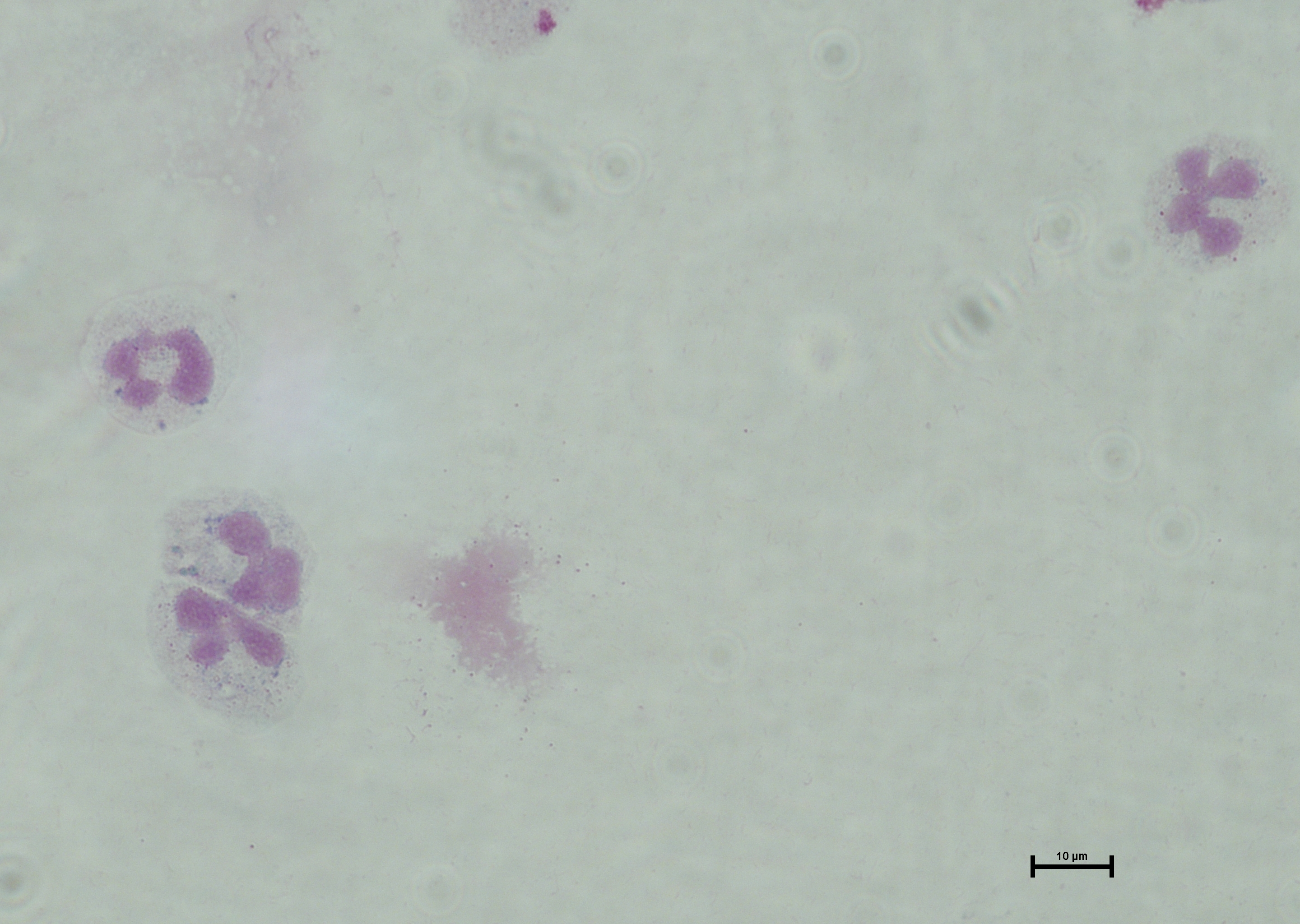

Supplement: Supplementary file 9 — EV Figures Source Data [file 44319_2024_150_MOESM9_ESM.zip › Figure EV1/Fig S1E/cytospin images with scale bar/14.12.2021 mock_01.jpg]

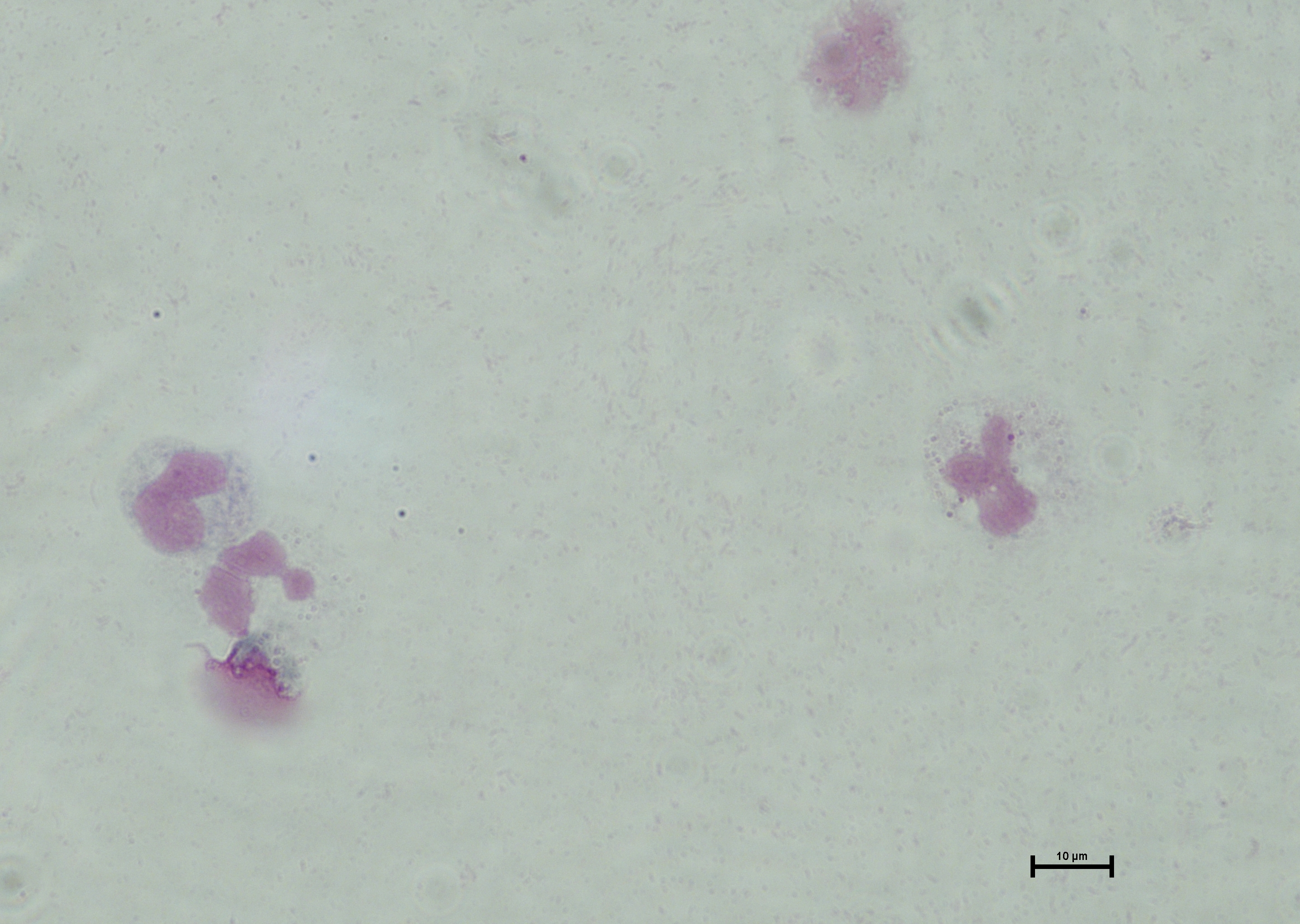

Supplement: Supplementary file 9 — EV Figures Source Data [file 44319_2024_150_MOESM9_ESM.zip › Figure EV1/Fig S1E/cytospin images with scale bar/14.12.2021 mock_02.jpg]

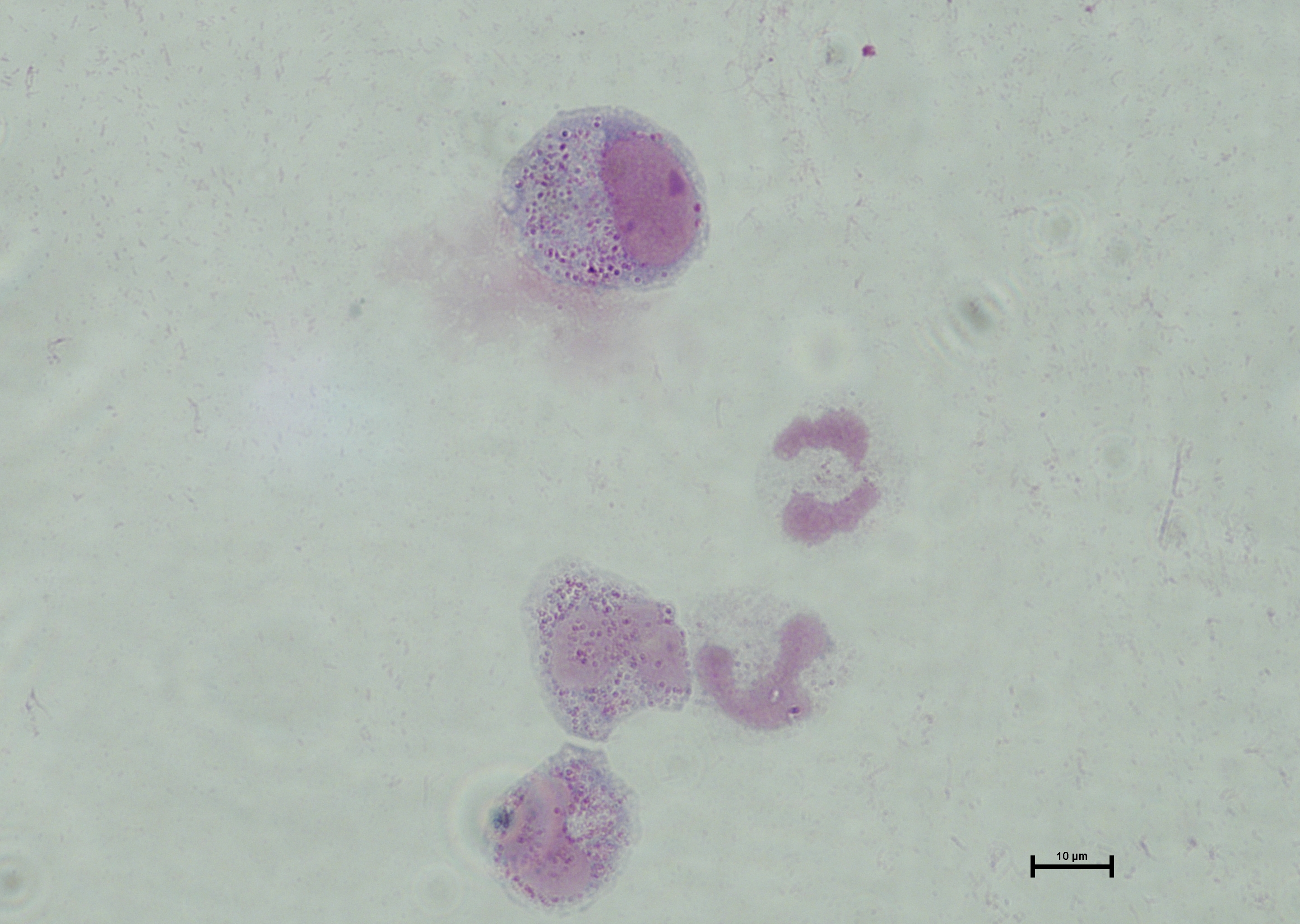

Supplement: Supplementary file 9 — EV Figures Source Data [file 44319_2024_150_MOESM9_ESM.zip › Figure EV1/Fig S1E/cytospin images with scale bar/14.12.2021 mock_03.jpg]

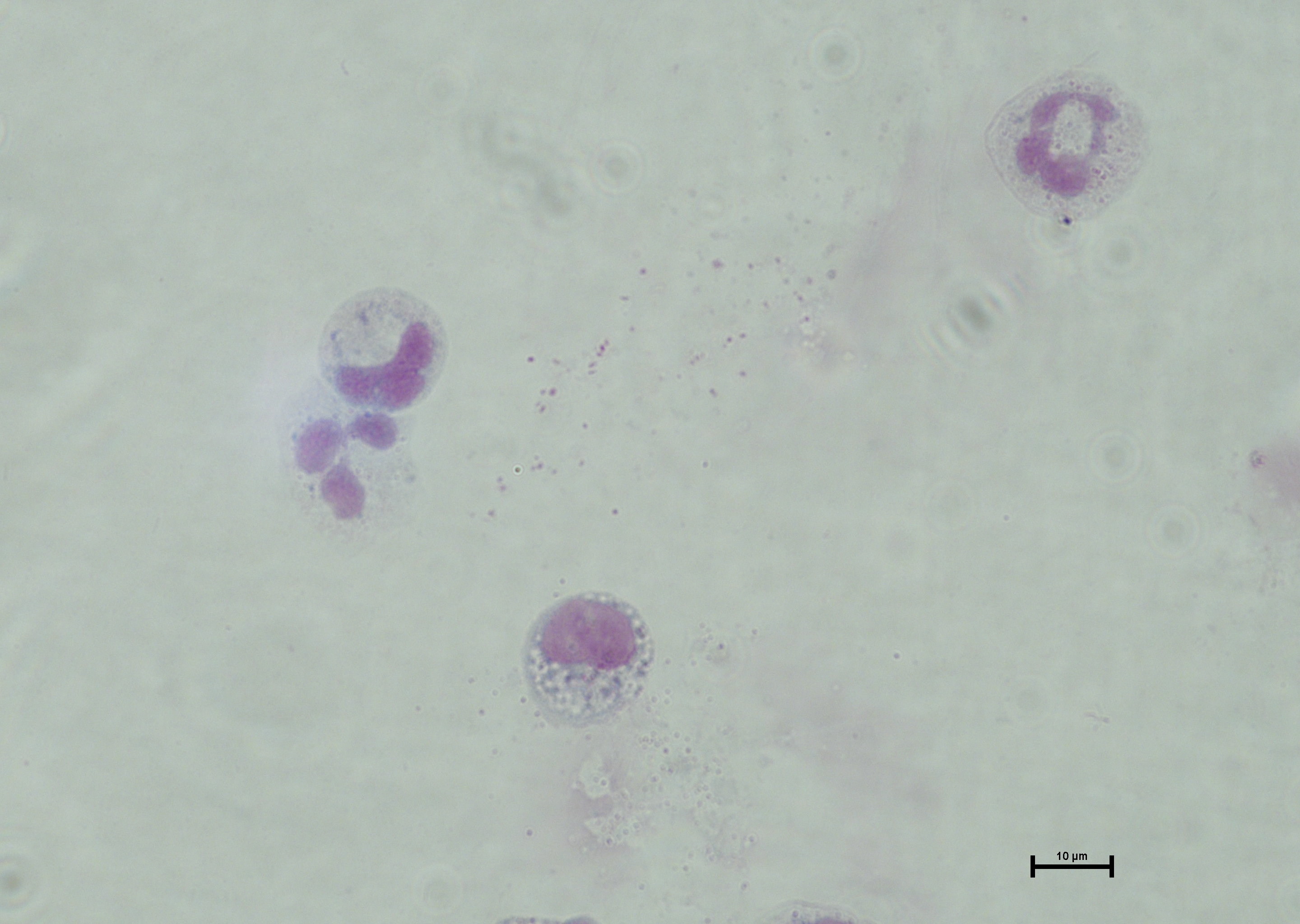

Supplement: Supplementary file 9 — EV Figures Source Data [file 44319_2024_150_MOESM9_ESM.zip › Figure EV1/Fig S1E/cytospin images with scale bar/14.12.2021 mock_04.jpg]

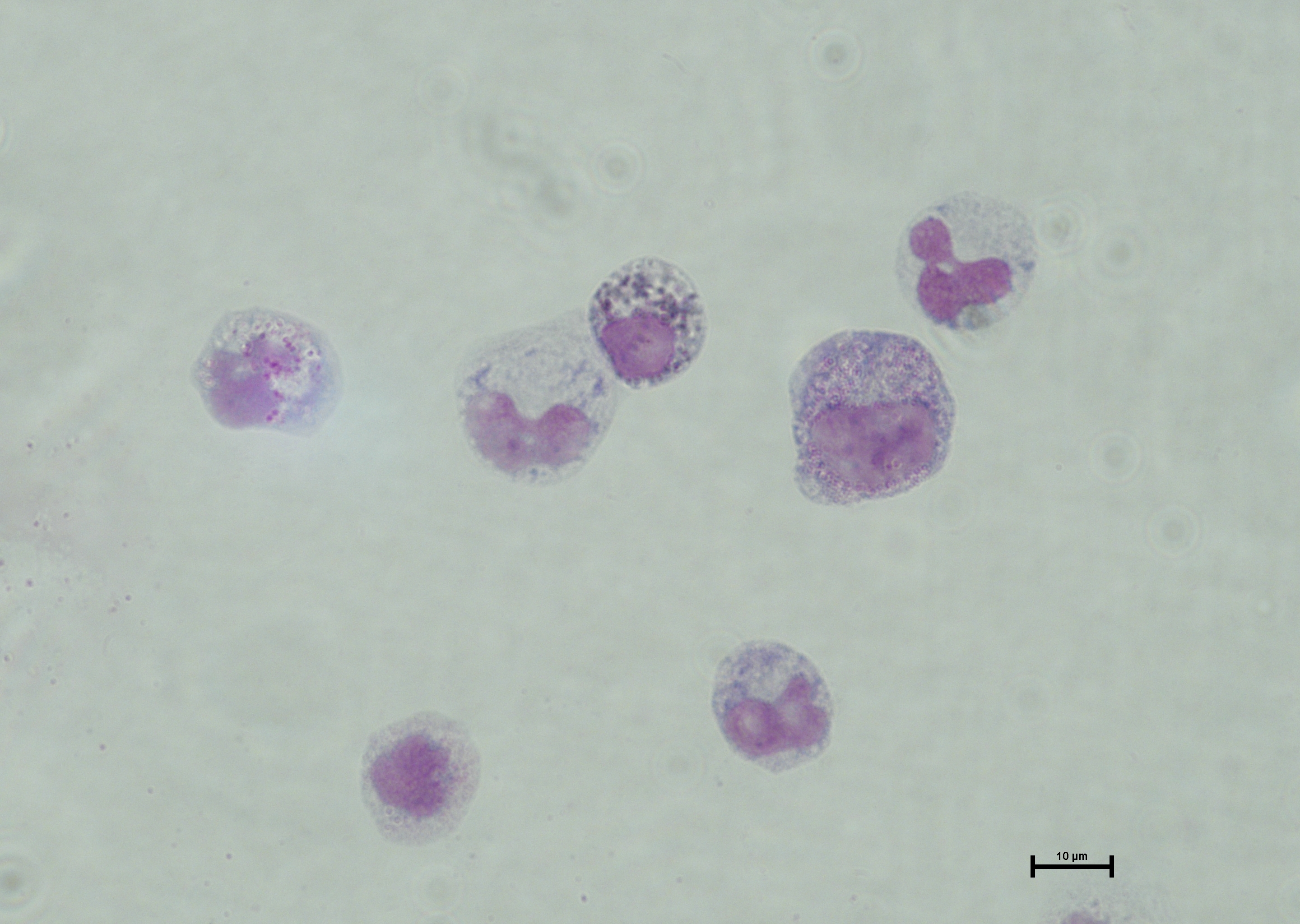

Supplement: Supplementary file 9 — EV Figures Source Data [file 44319_2024_150_MOESM9_ESM.zip › Figure EV1/Fig S1E/cytospin images with scale bar/14.12.2021 mock_05.jpg]

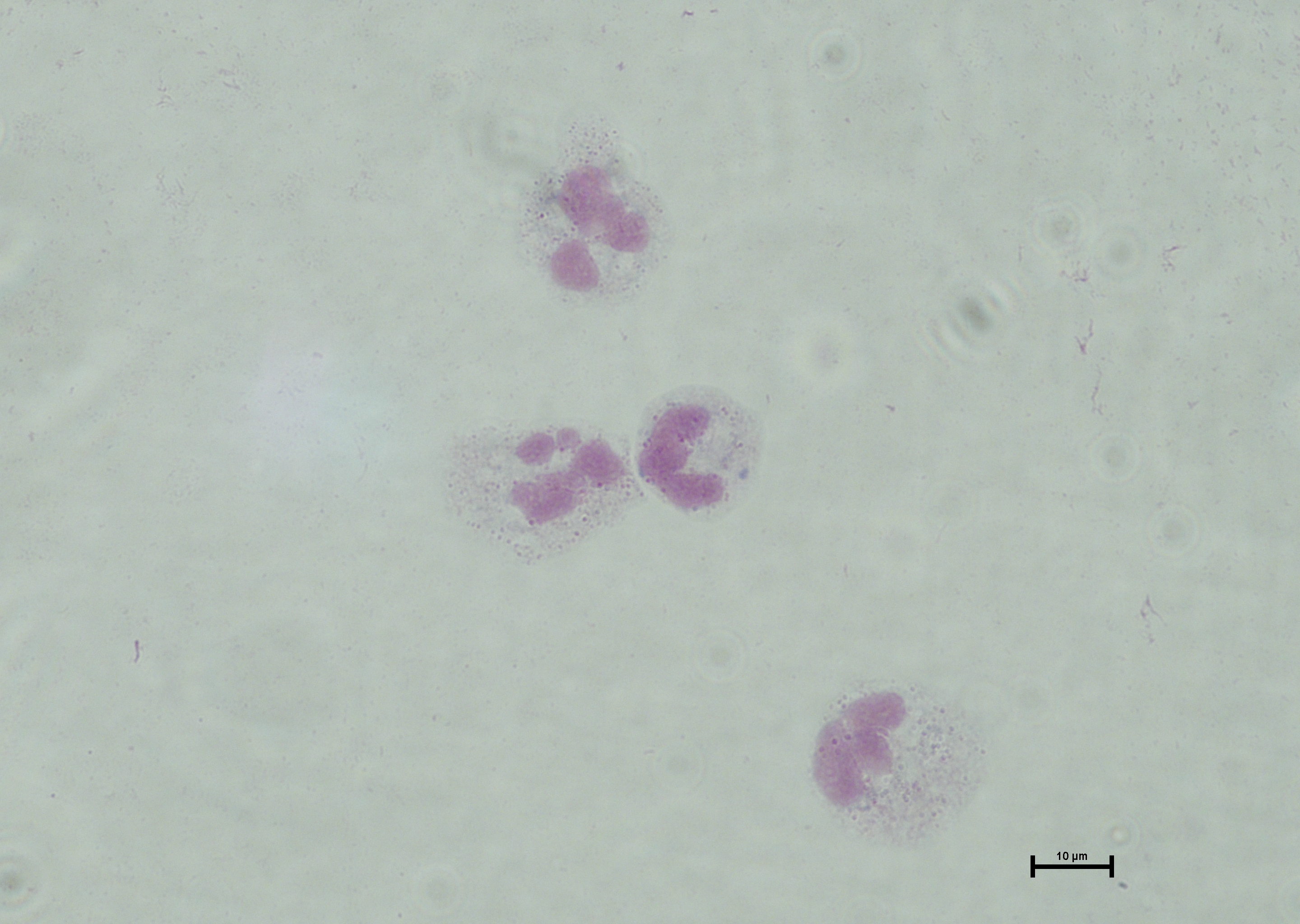

Supplement: Supplementary file 9 — EV Figures Source Data [file 44319_2024_150_MOESM9_ESM.zip › Figure EV1/Fig S1E/cytospin images with scale bar/14.12.2021 mock_06.jpg]

## Slide 1
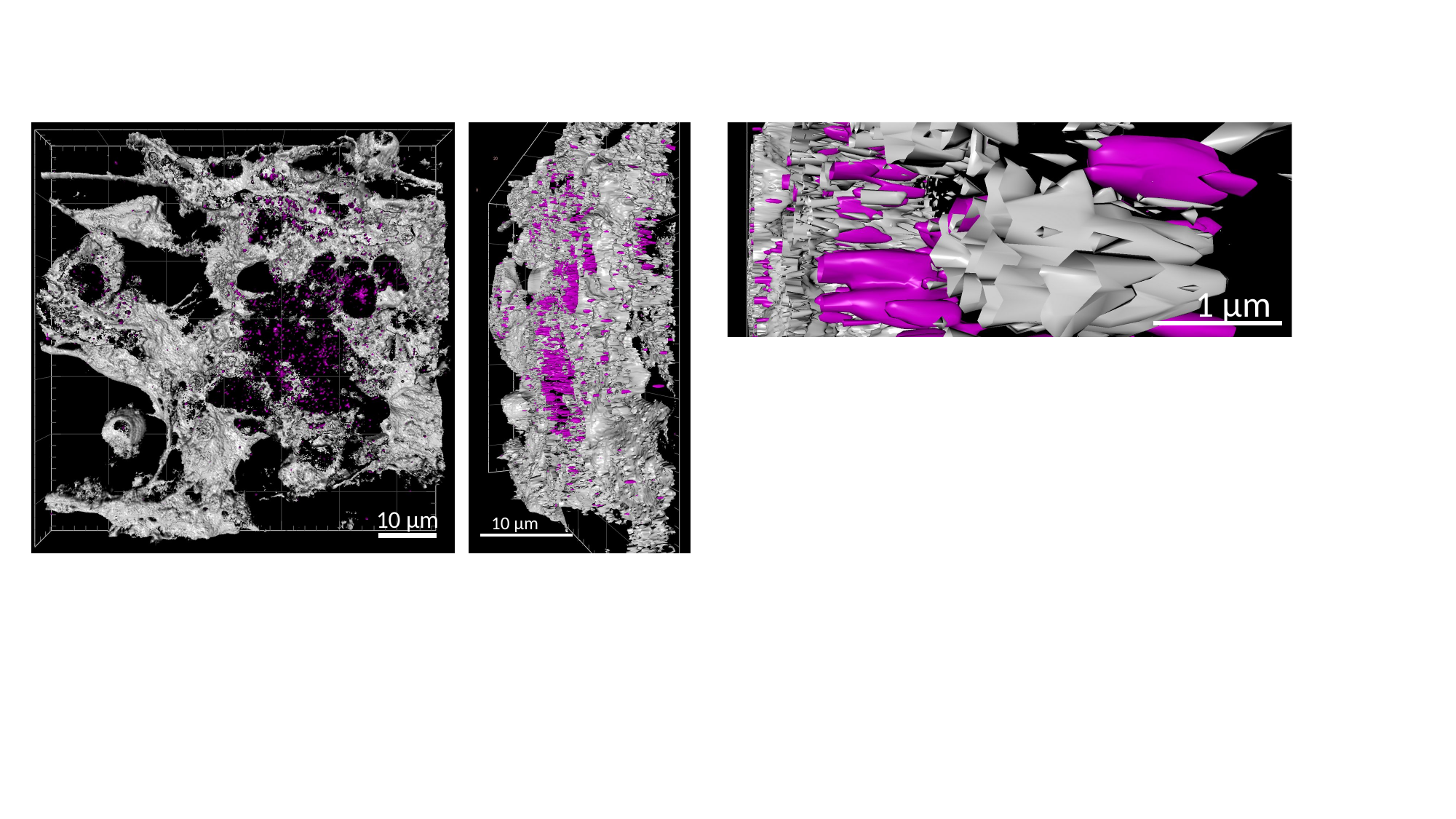

1 μm
10 μm
10 μm

Supplement: Supplementary file 9 — EV Figures Source Data [file 44319_2024_150_MOESM9_ESM.zip › Figure EV1/Fig S1G/3D images/120722 3D.pptx]

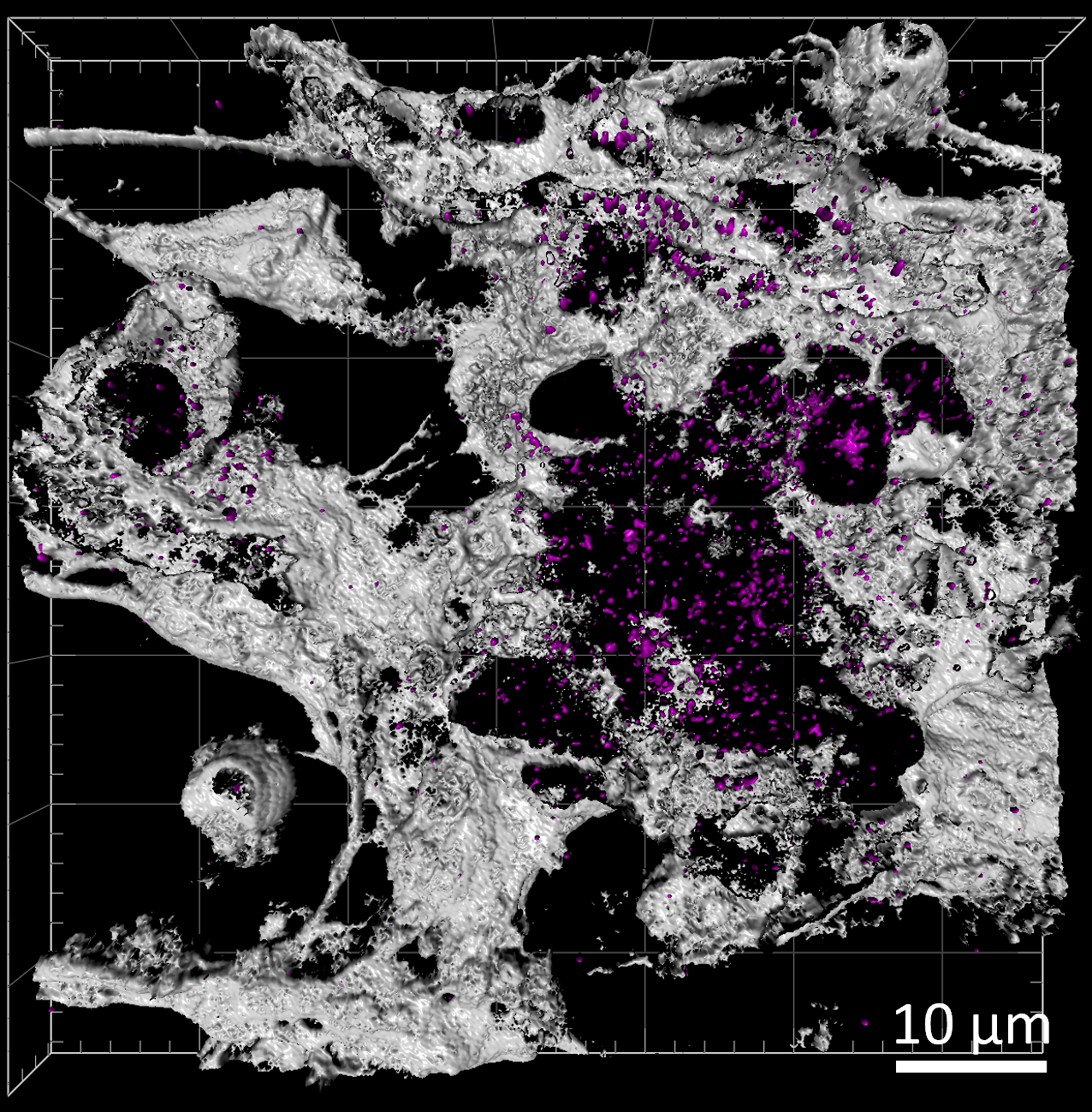

Supplement: Supplementary file 9 — EV Figures Source Data [file 44319_2024_150_MOESM9_ESM.zip › Figure EV1/Fig S1G/3D images/complete scale bar.png]

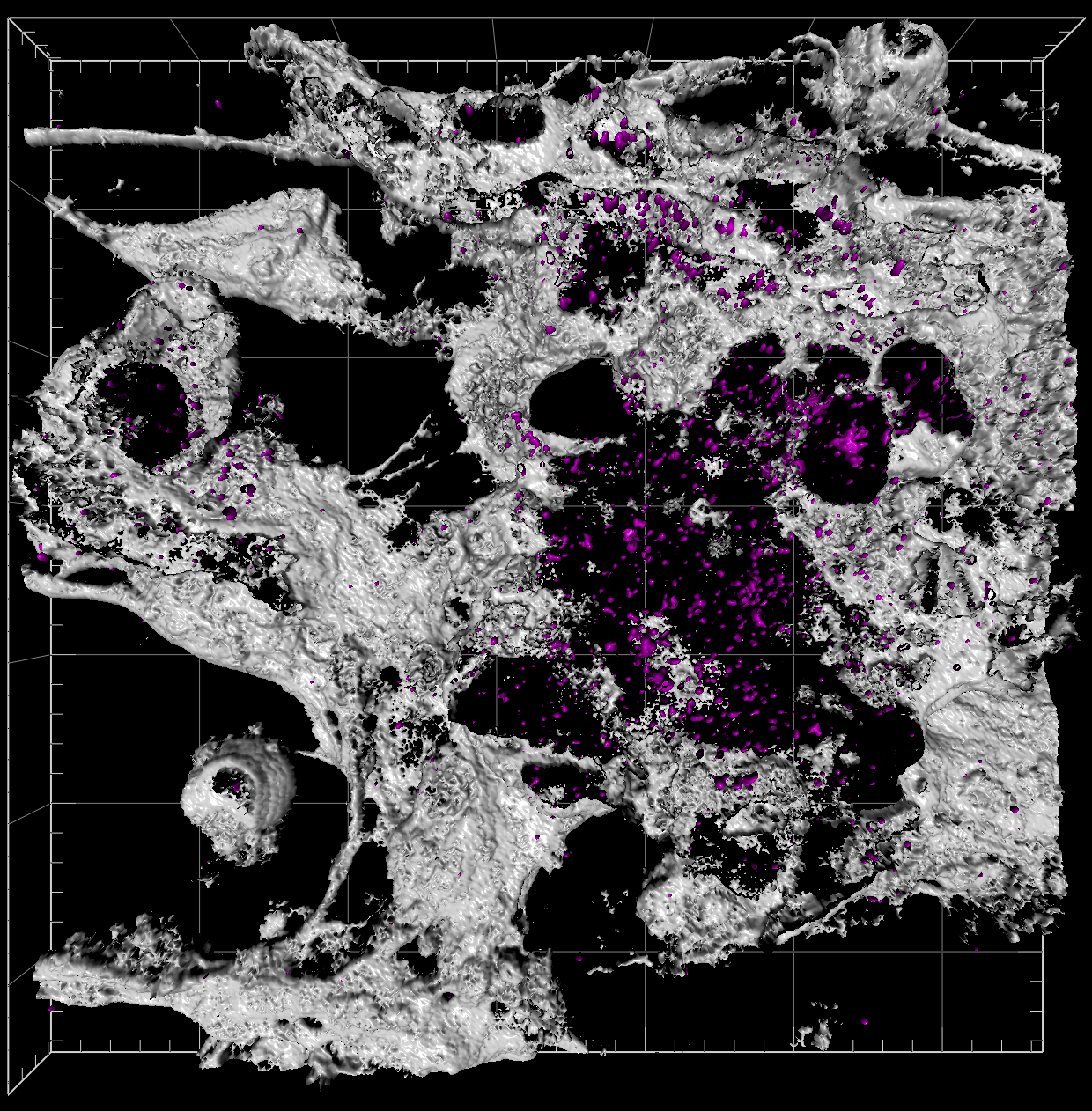

Supplement: Supplementary file 9 — EV Figures Source Data [file 44319_2024_150_MOESM9_ESM.zip › Figure EV1/Fig S1G/3D images/complete.png]

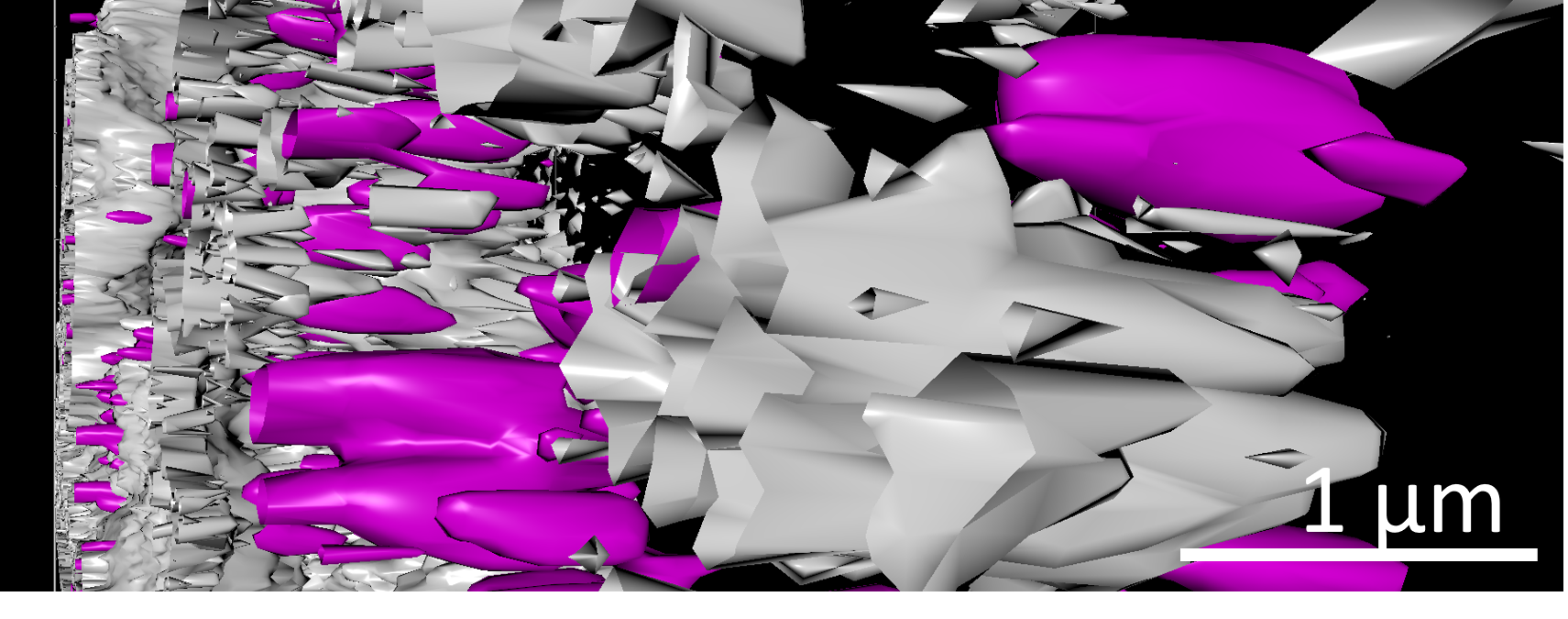

Supplement: Supplementary file 9 — EV Figures Source Data [file 44319_2024_150_MOESM9_ESM.zip › Figure EV1/Fig S1G/3D images/zoom in scale bar.png]

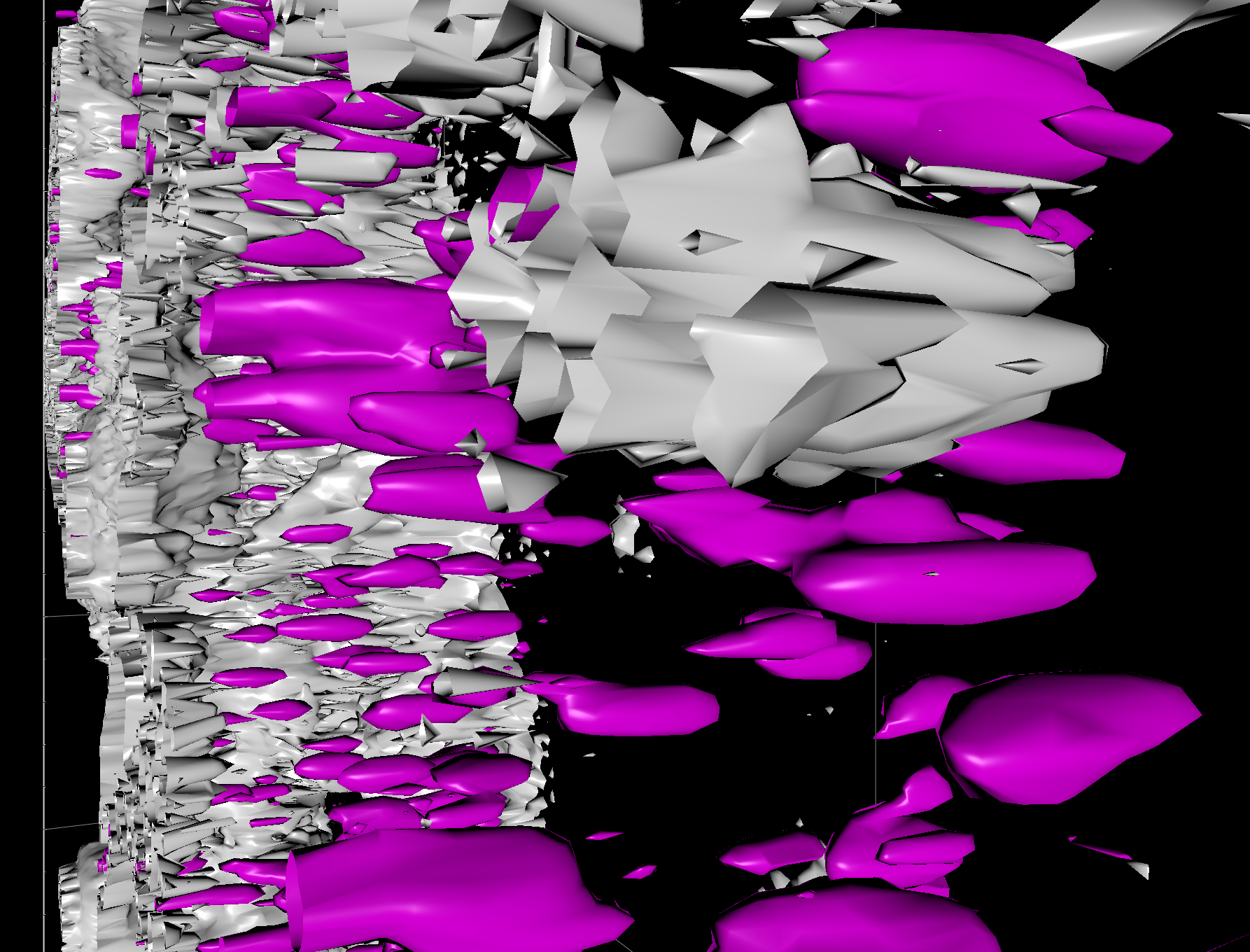

Supplement: Supplementary file 9 — EV Figures Source Data [file 44319_2024_150_MOESM9_ESM.zip › Figure EV1/Fig S1G/3D images/zoom in.png]

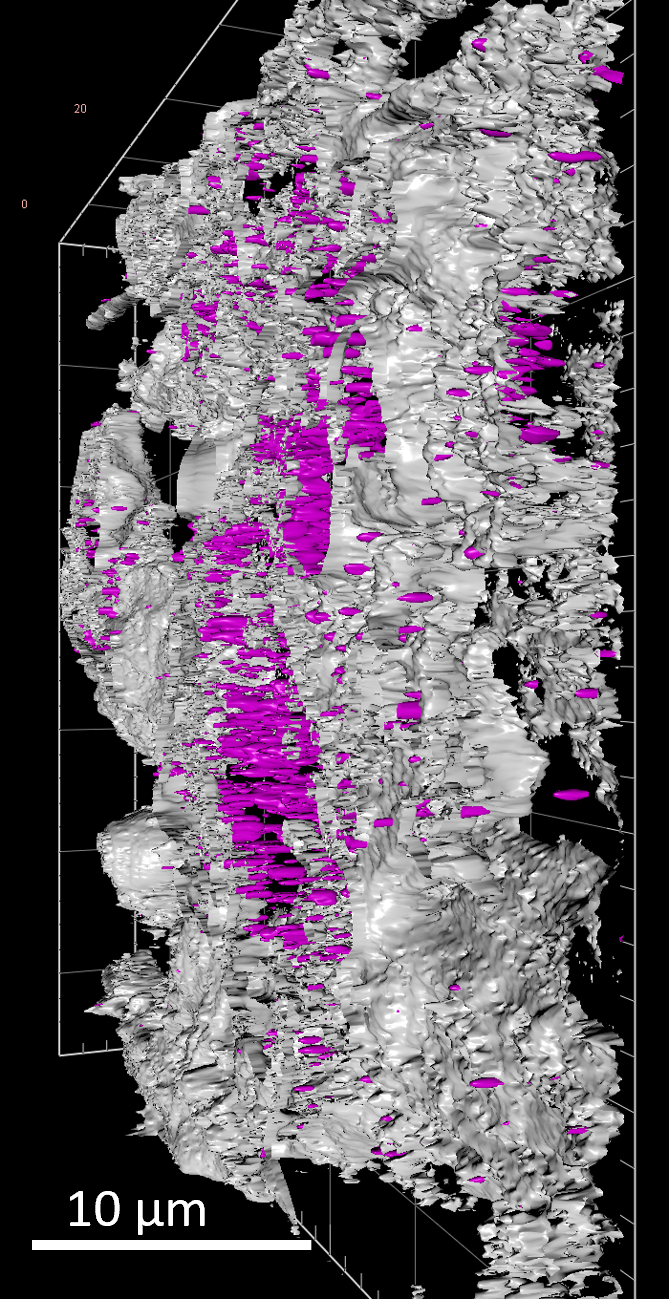

Supplement: Supplementary file 9 — EV Figures Source Data [file 44319_2024_150_MOESM9_ESM.zip › Figure EV1/Fig S1G/3D images/zoom scale bar.png]

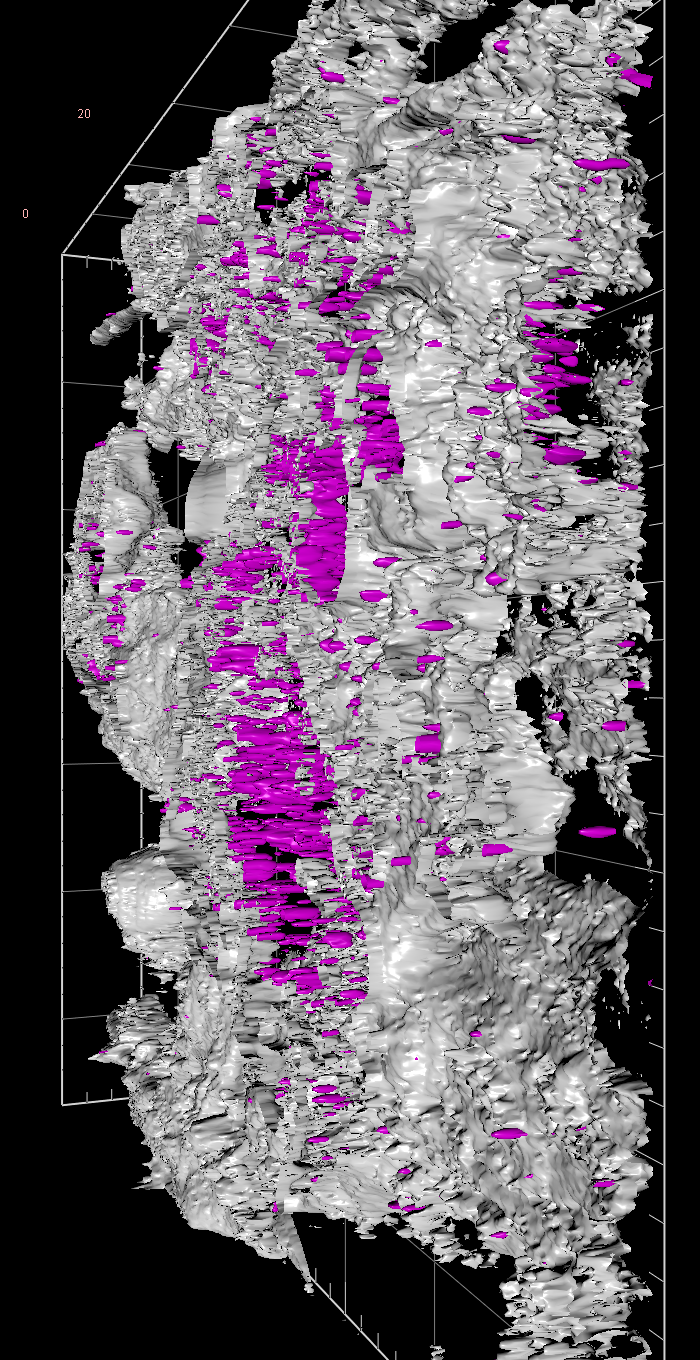

Supplement: Supplementary file 9 — EV Figures Source Data [file 44319_2024_150_MOESM9_ESM.zip › Figure EV1/Fig S1G/3D images/zoom.png]

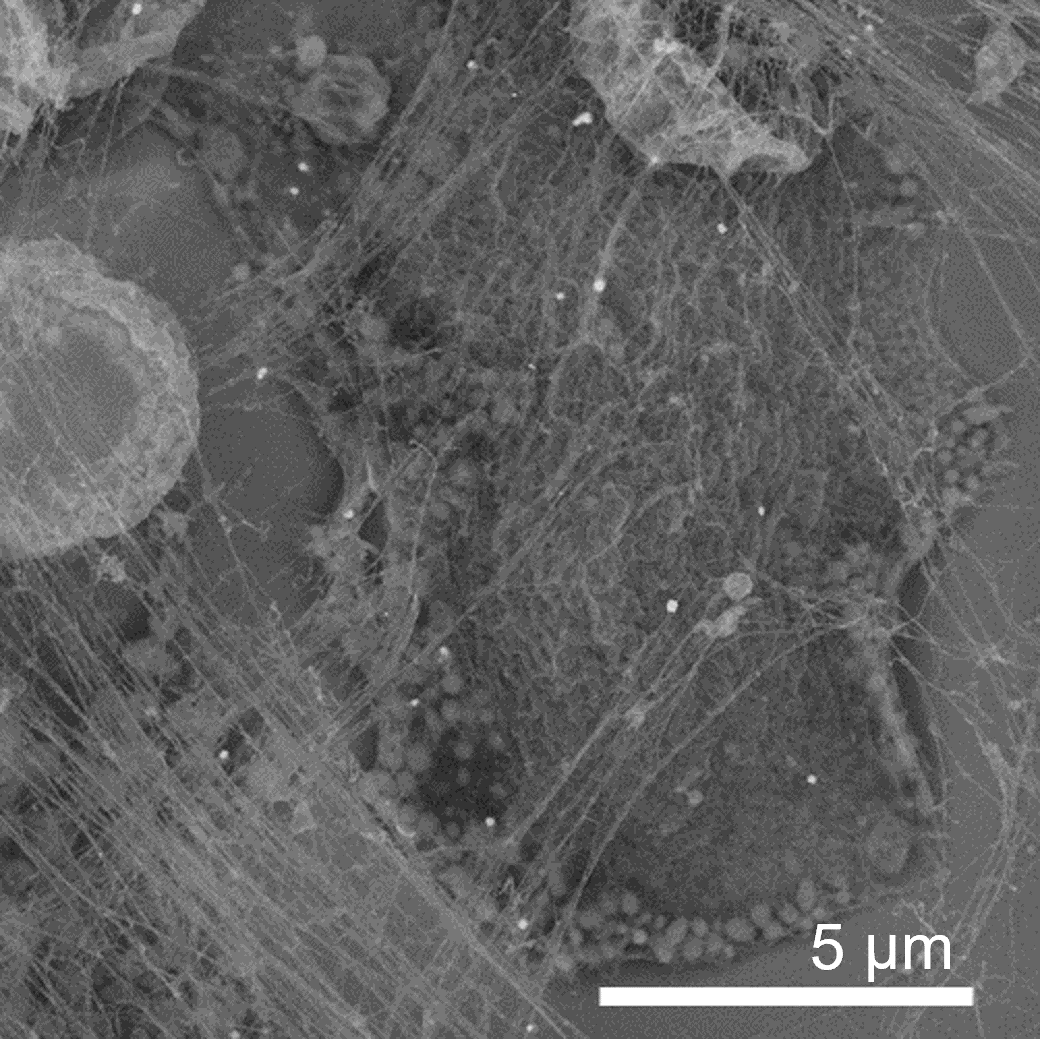

Supplement: Supplementary file 9 — EV Figures Source Data [file 44319_2024_150_MOESM9_ESM.zip › Figure EV1/Fig S1H/neg ctrls EM higher zoom.png]

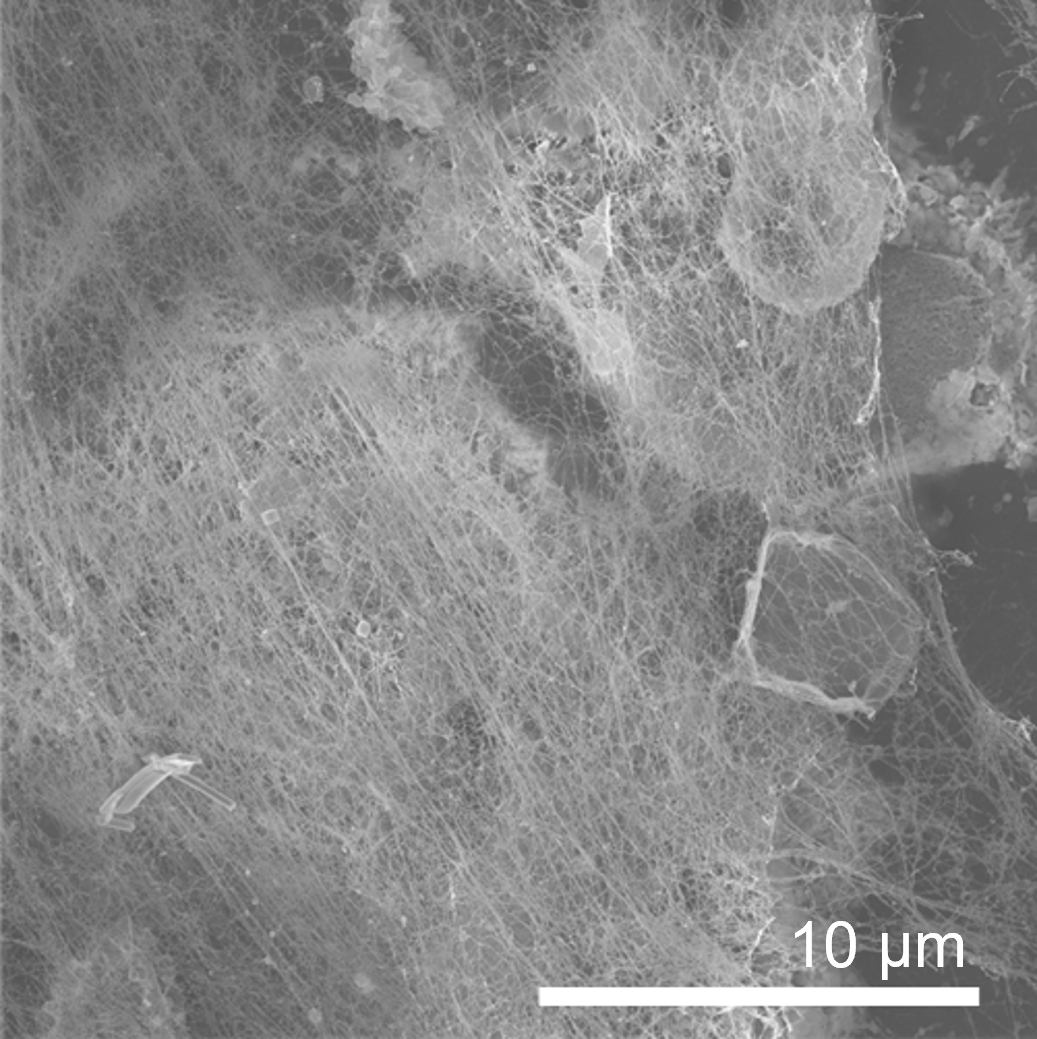

Supplement: Supplementary file 9 — EV Figures Source Data [file 44319_2024_150_MOESM9_ESM.zip › Figure EV1/Fig S1H/neg ctrls EM.png]

## Slide 1
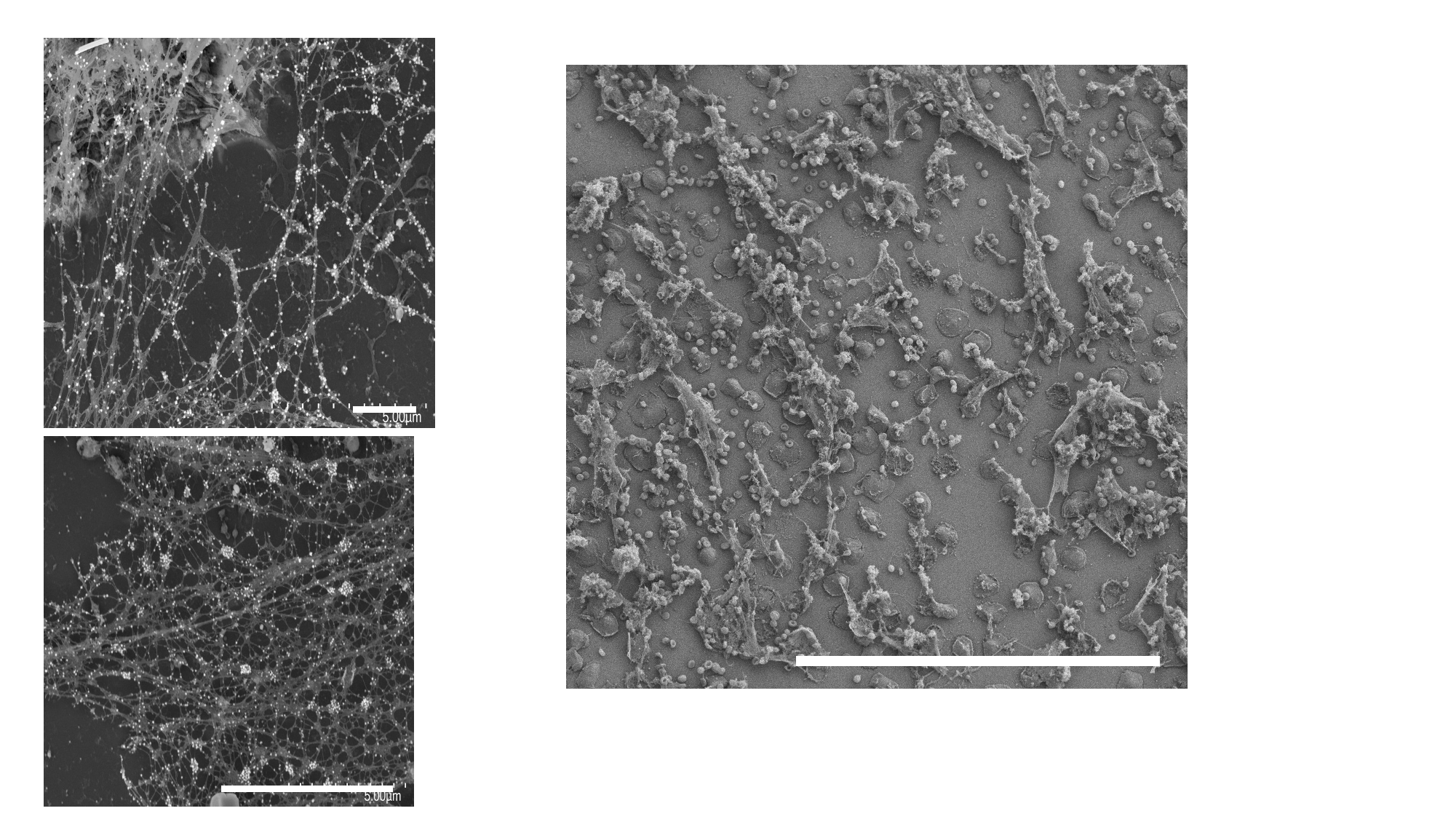

1 μm
200 μm
5 μm

## Slide 2
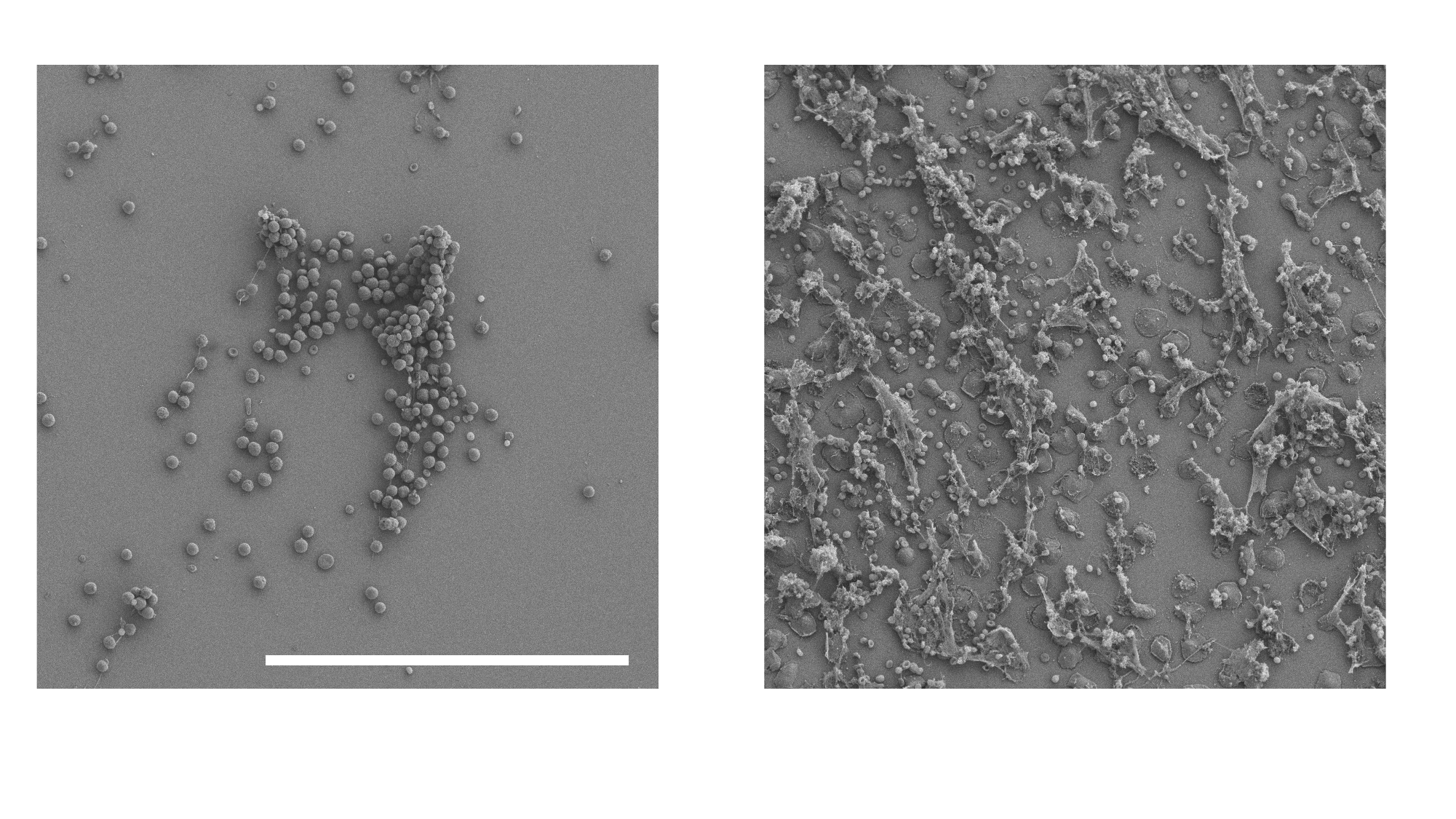

1 μm
5 μm

## Slide 3
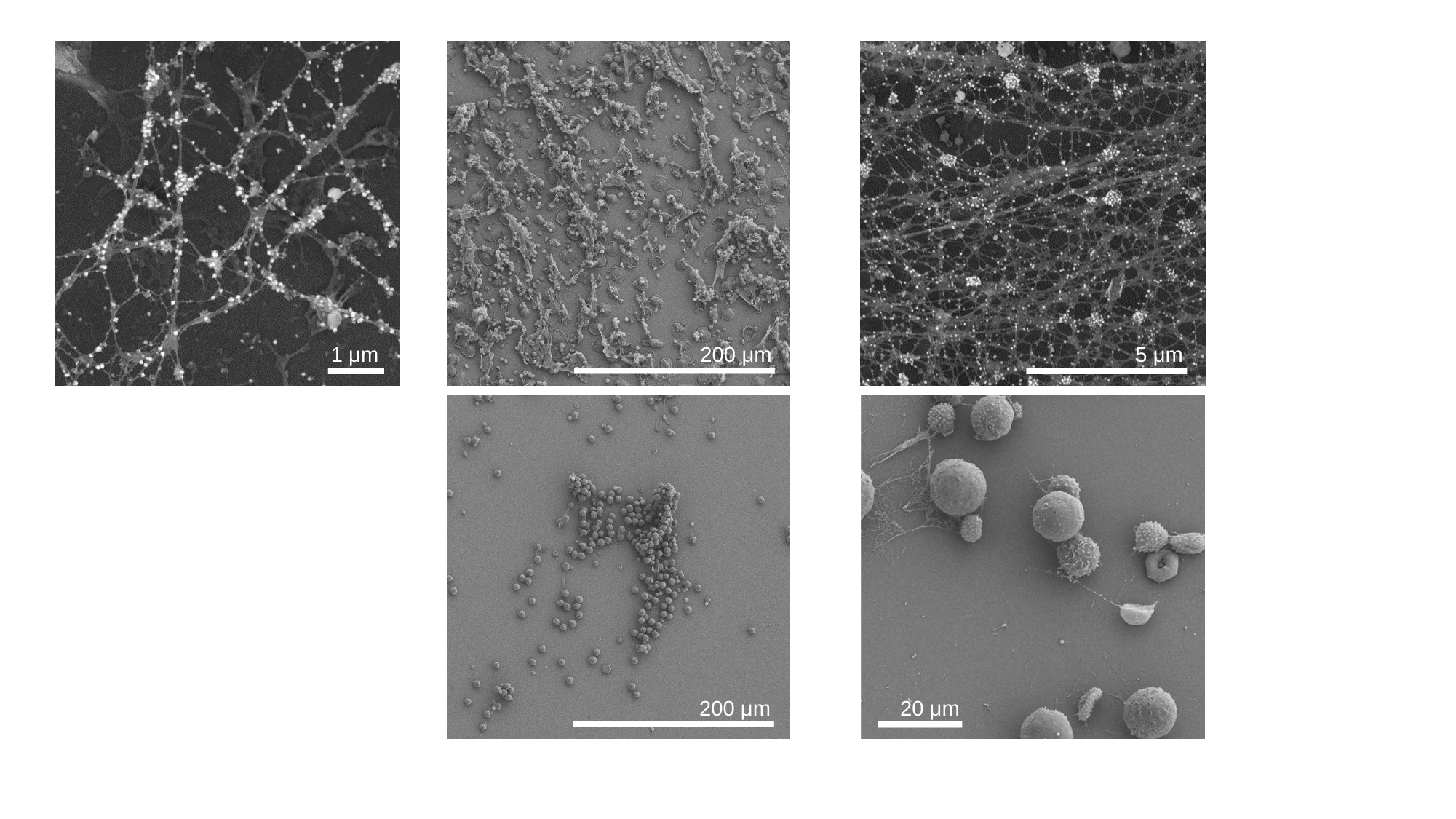

5 μm
200 μm
1 μm
20 μm
200 μm

## Slide 4
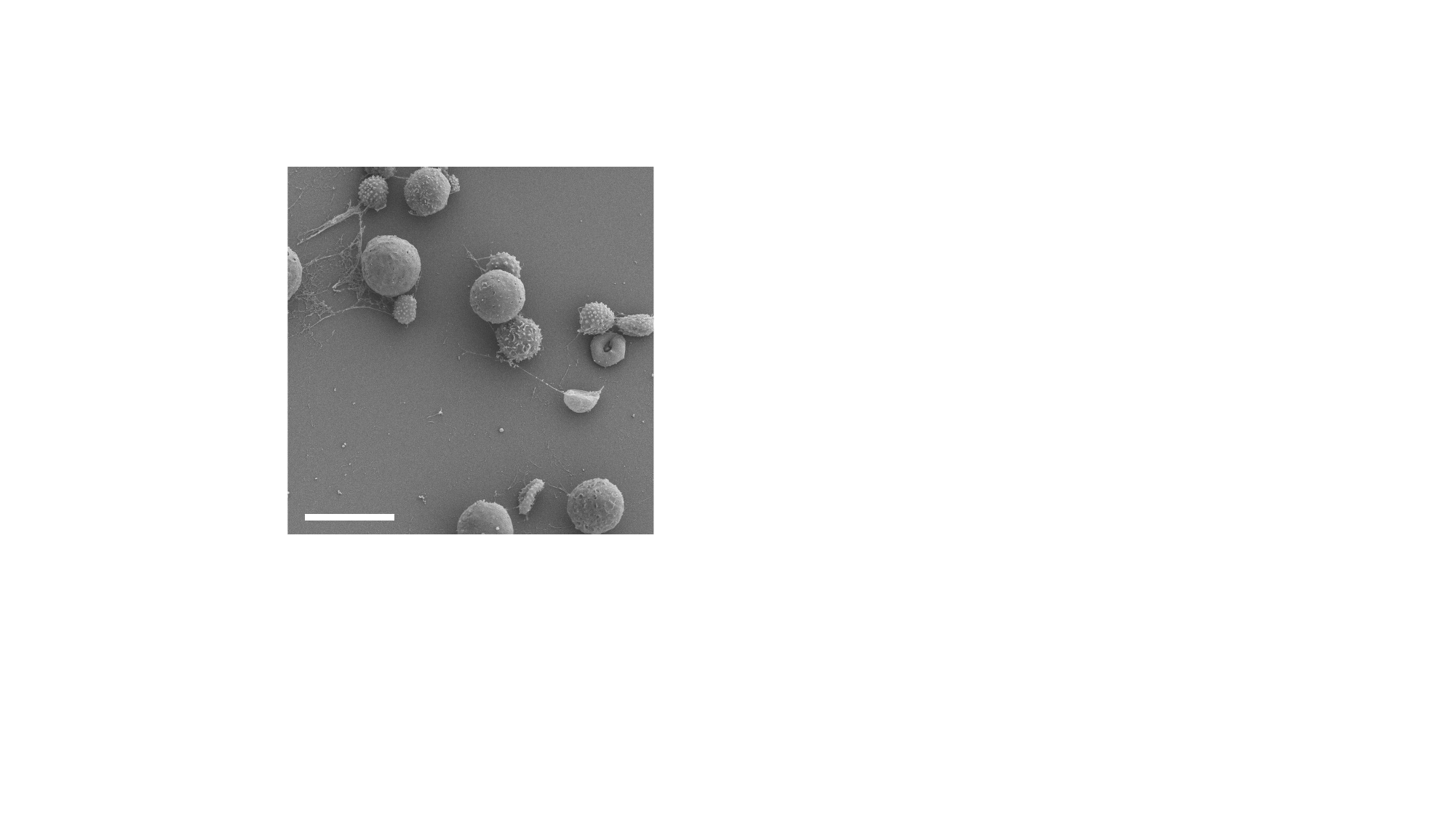

## Slide 5
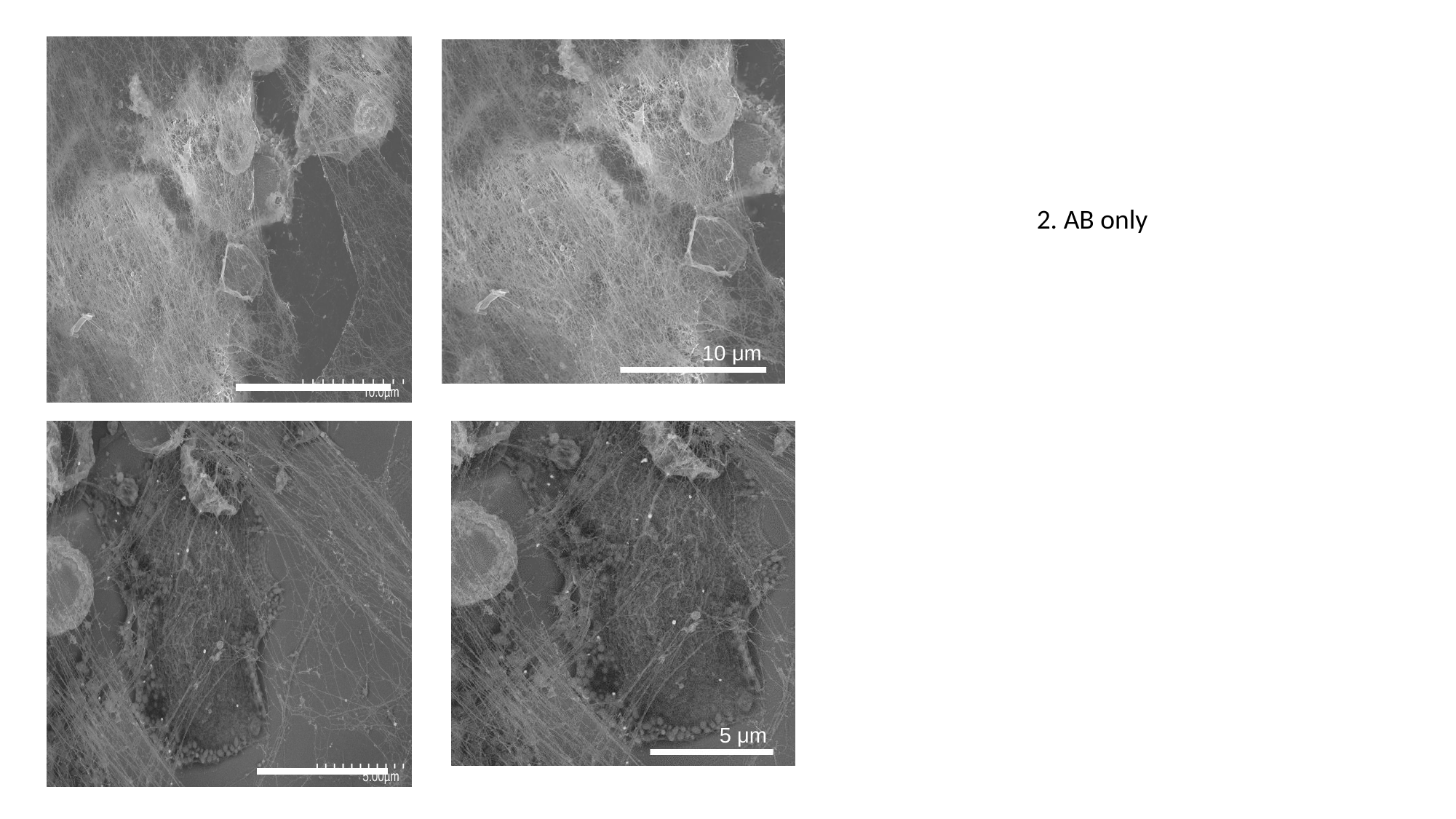

10 μm
2. AB only
5 μm

Supplement: Supplementary file 9 — EV Figures Source Data [file 44319_2024_150_MOESM9_ESM.zip › Figure EV1/Fig S1H/Präsentation1.pptx]
