# Supplementary figures and images for: naRNA-LL37 composite DAMPs define sterile NETs as self-propagating drivers of inflammation (part 4 of 4)
Source: EMBO Rep. 2024 May 23;25(7):10. doi: 10.1038/s44319-024-00150-5 (PMC11239898; doi:10.1038/s44319-024-00150-5)

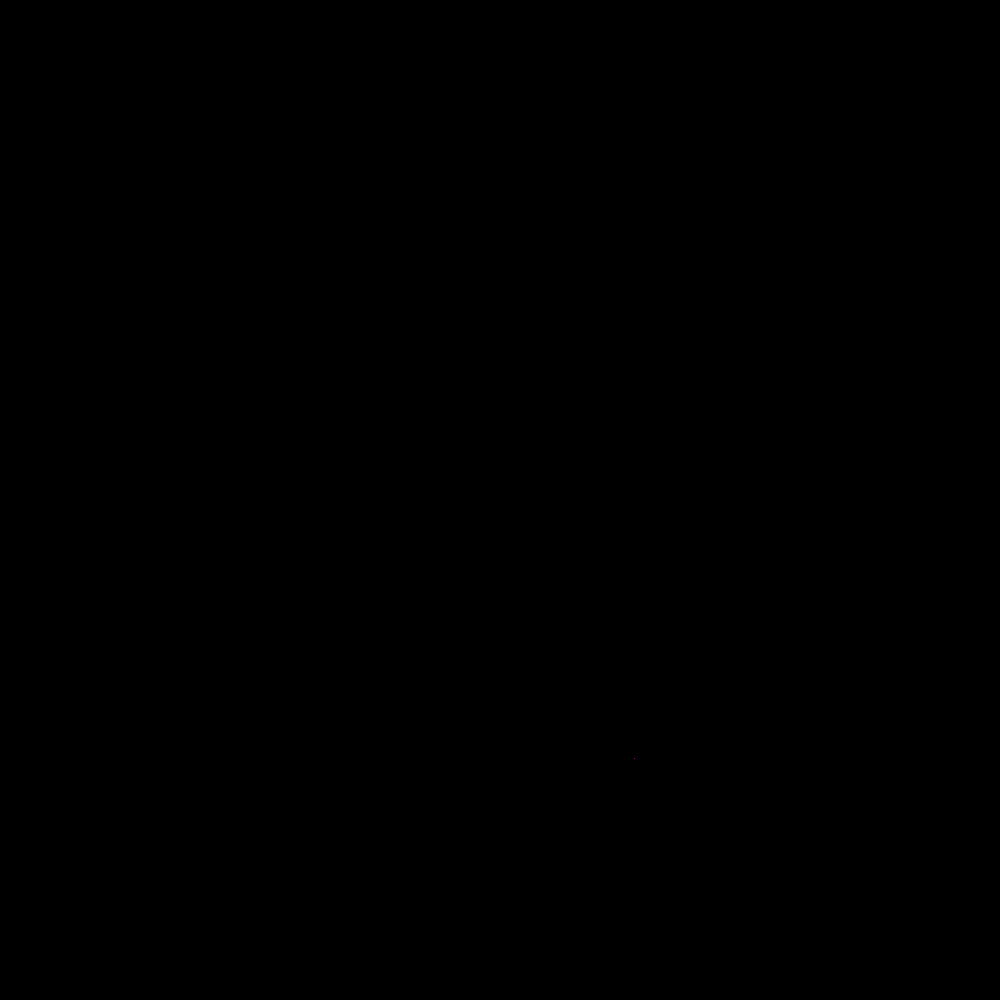

Supplement: Supplementary file 9 — EV Figures Source Data [file 44319_2024_150_MOESM9_ESM.zip › Figure EV5/Fig S5A/rRNA Y10b (secondary only) LL37-dylight550/C1-MAX_Experiment-3579-Airyscan Processing-03.png]

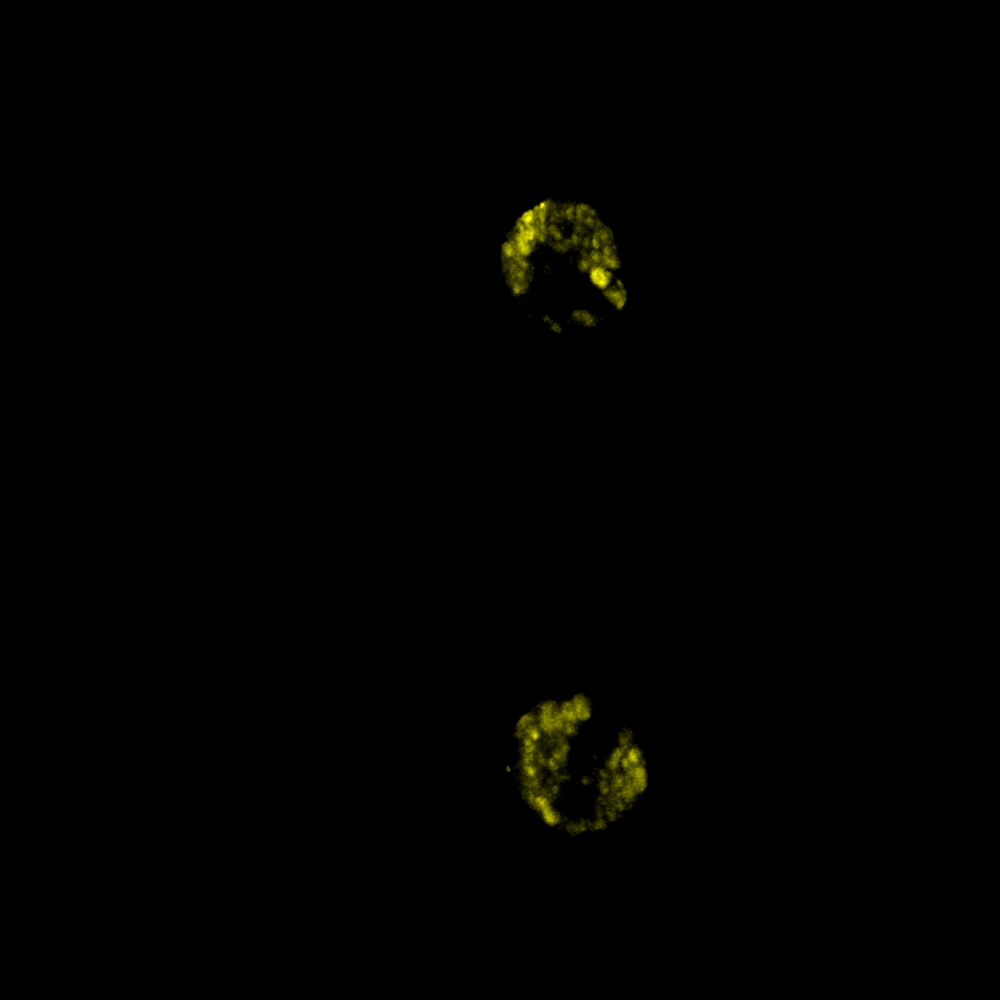

Supplement: Supplementary file 9 — EV Figures Source Data [file 44319_2024_150_MOESM9_ESM.zip › Figure EV5/Fig S5A/rRNA Y10b (secondary only) LL37-dylight550/C2-MAX_Experiment-3579-Airyscan Processing-03.png]

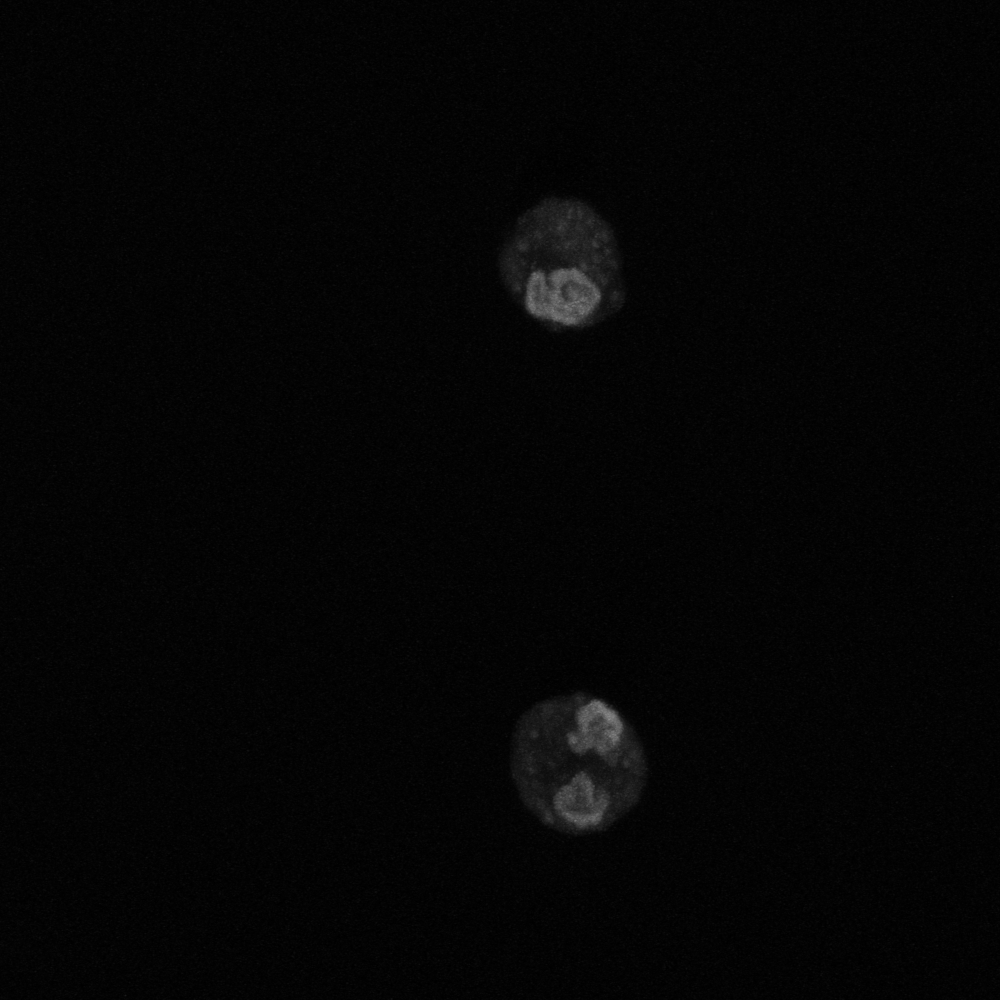

Supplement: Supplementary file 9 — EV Figures Source Data [file 44319_2024_150_MOESM9_ESM.zip › Figure EV5/Fig S5A/rRNA Y10b (secondary only) LL37-dylight550/C3-MAX_Experiment-3579-Airyscan Processing-03.png]

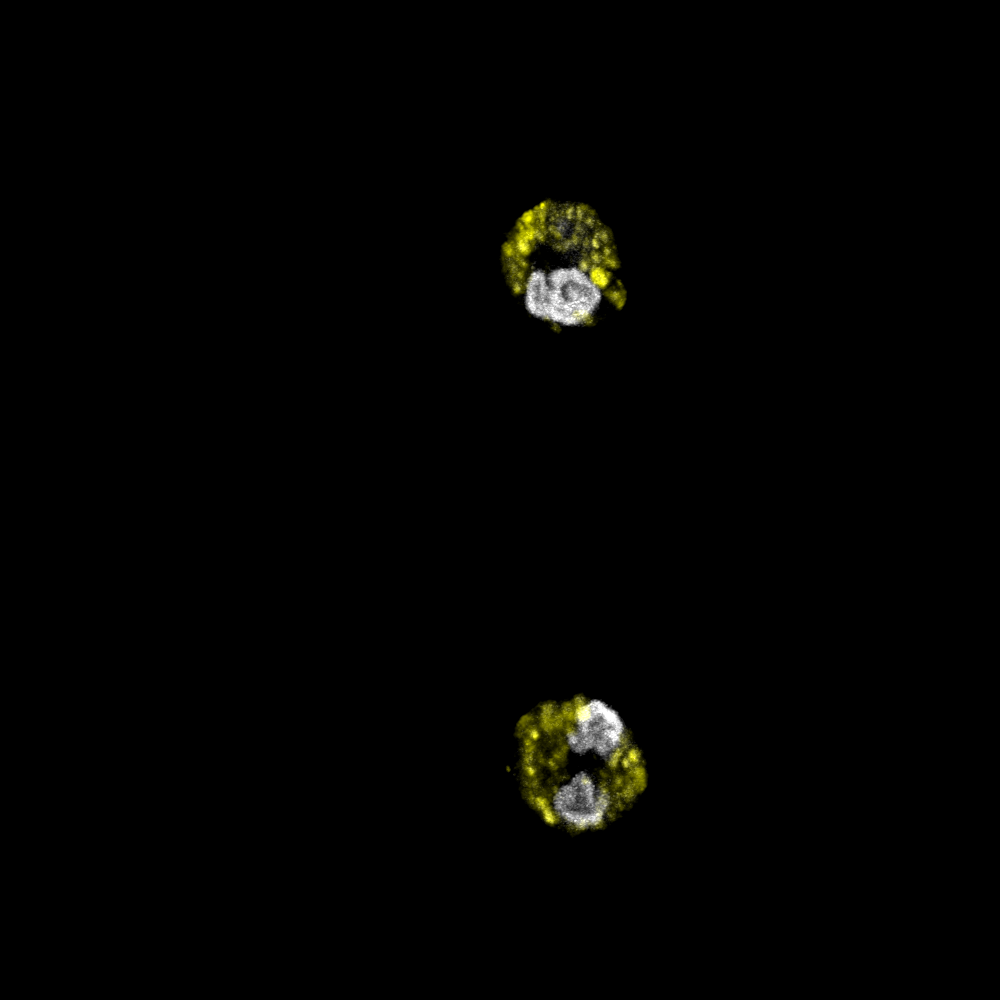

Supplement: Supplementary file 9 — EV Figures Source Data [file 44319_2024_150_MOESM9_ESM.zip › Figure EV5/Fig S5A/rRNA Y10b (secondary only) LL37-dylight550/MAX_Experiment-3579-Airyscan Processing-03.png]

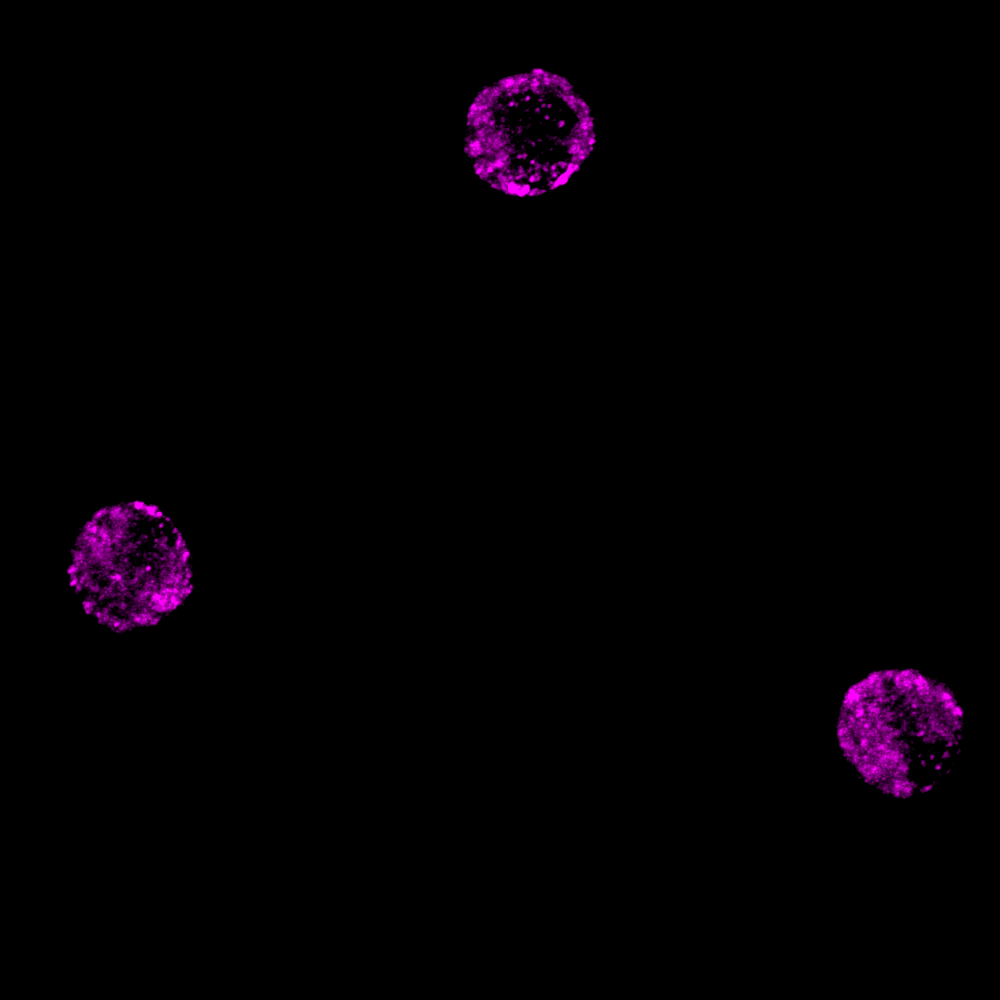

Supplement: Supplementary file 9 — EV Figures Source Data [file 44319_2024_150_MOESM9_ESM.zip › Figure EV5/Fig S5A/rRNA Y10b only (primary and secondary)/C1-MAX_Experiment-3580-Airyscan Processing-04.png]

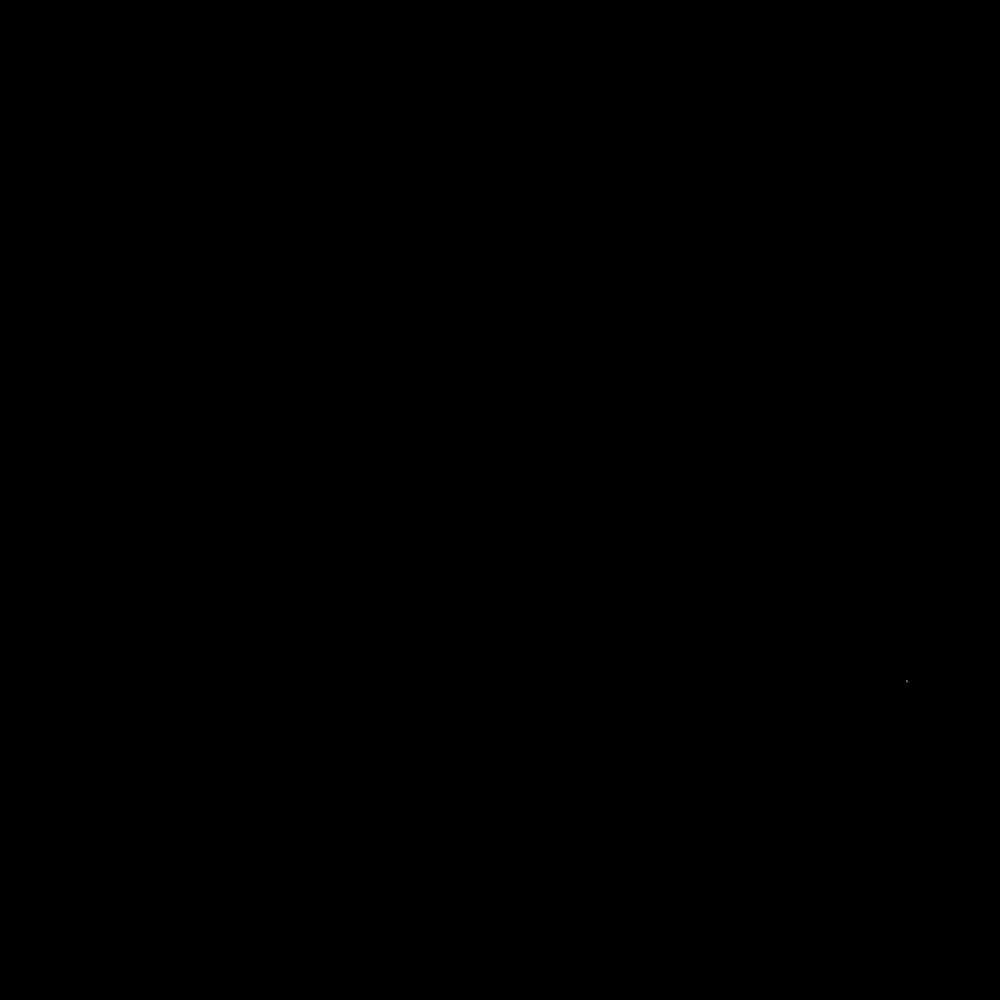

Supplement: Supplementary file 9 — EV Figures Source Data [file 44319_2024_150_MOESM9_ESM.zip › Figure EV5/Fig S5A/rRNA Y10b only (primary and secondary)/C2-MAX_Experiment-3580-Airyscan Processing-04.png]

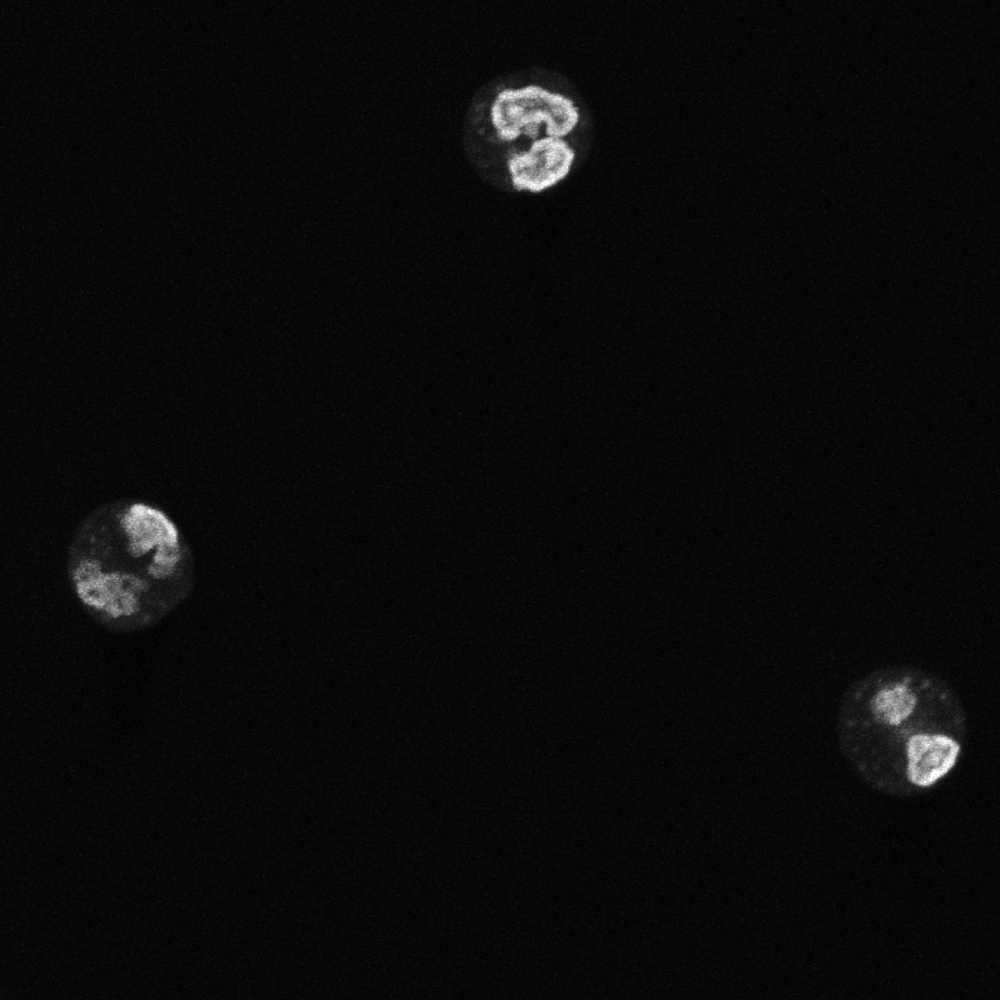

Supplement: Supplementary file 9 — EV Figures Source Data [file 44319_2024_150_MOESM9_ESM.zip › Figure EV5/Fig S5A/rRNA Y10b only (primary and secondary)/C3-MAX_Experiment-3580-Airyscan Processing-04.png]

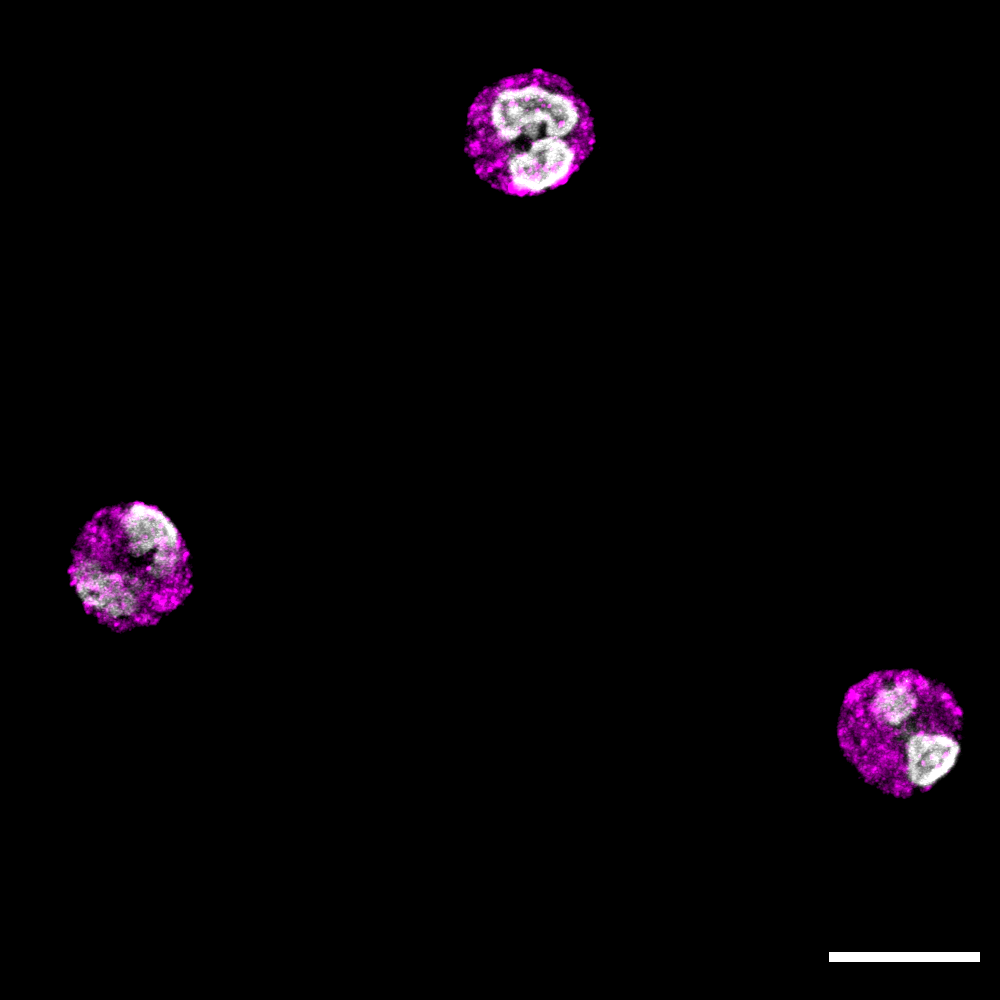

Supplement: Supplementary file 9 — EV Figures Source Data [file 44319_2024_150_MOESM9_ESM.zip › Figure EV5/Fig S5A/rRNA Y10b only (primary and secondary)/MAX_Experiment-3580-Airyscan Processing-04.png]

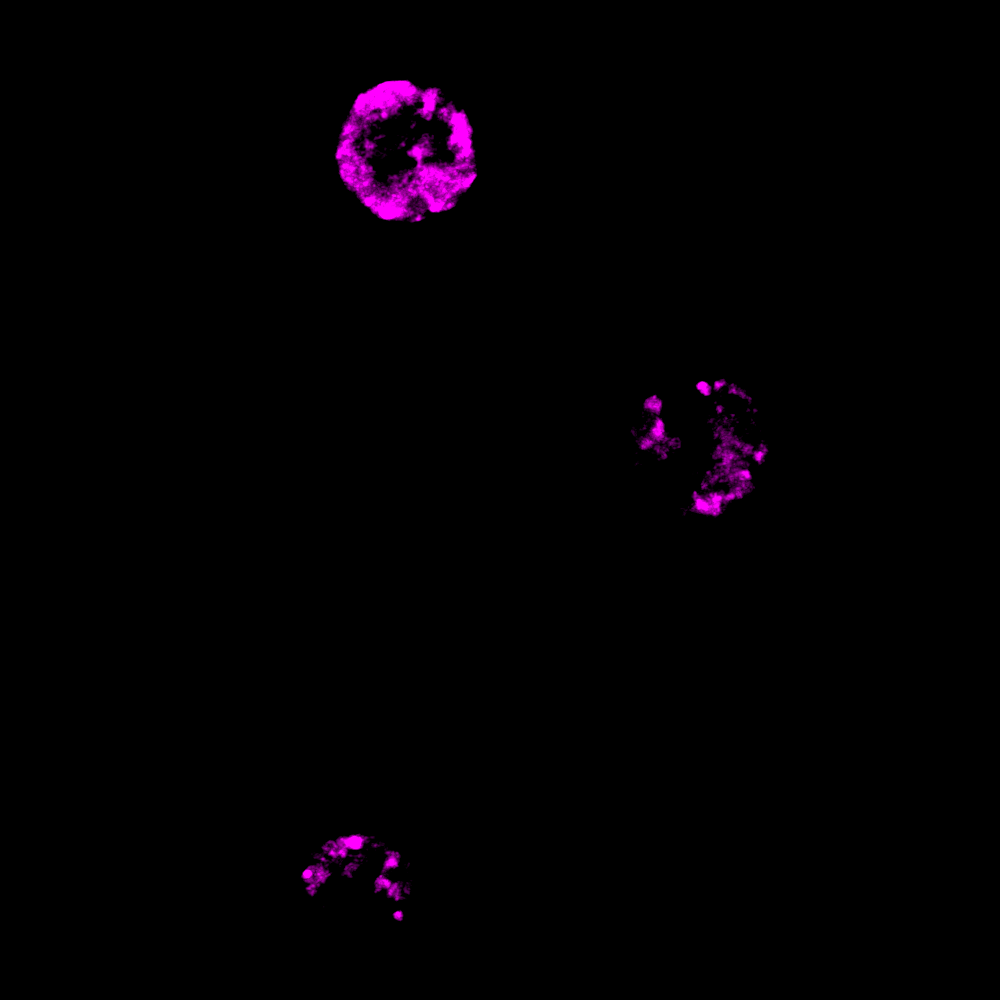

Supplement: Supplementary file 9 — EV Figures Source Data [file 44319_2024_150_MOESM9_ESM.zip › Figure EV5/Fig S5A/rRNA Y10b-AF647 LL37-dylight550/C1-MAX_Experiment-3577-Airyscan Processing-01.png]

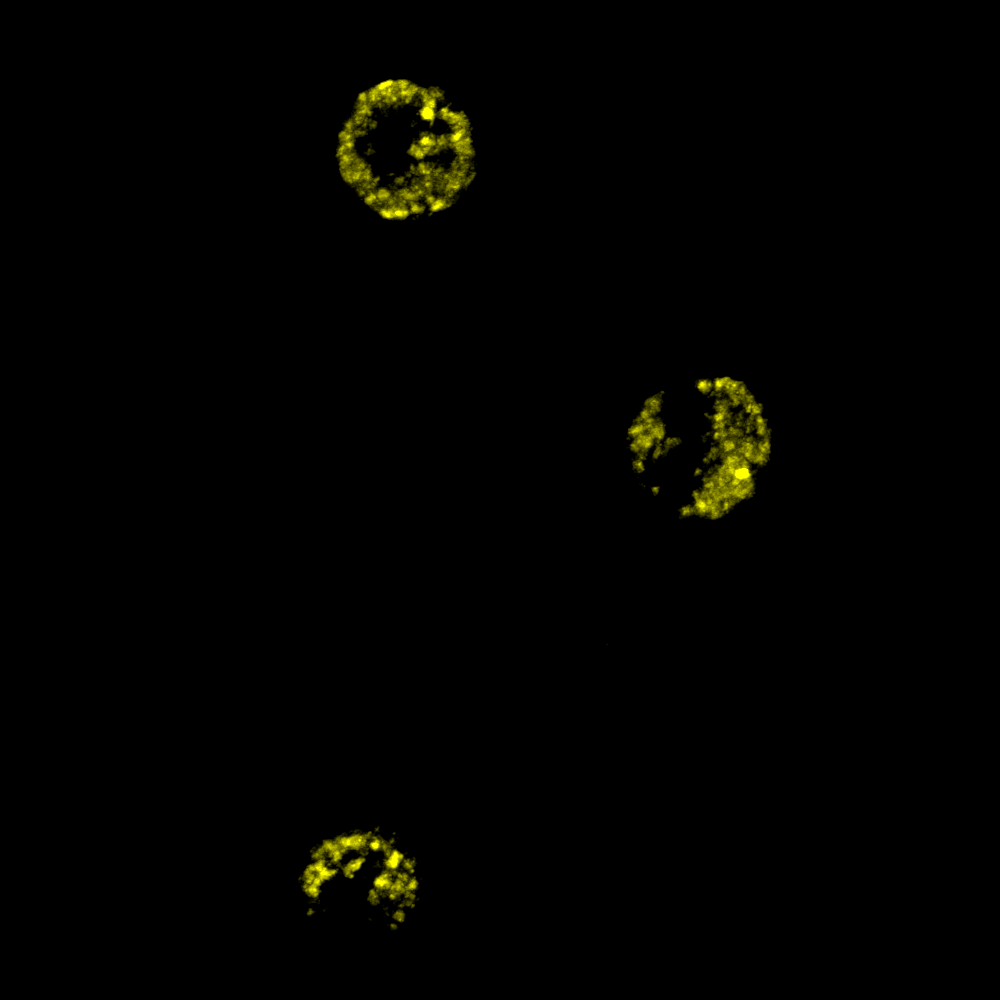

Supplement: Supplementary file 9 — EV Figures Source Data [file 44319_2024_150_MOESM9_ESM.zip › Figure EV5/Fig S5A/rRNA Y10b-AF647 LL37-dylight550/C2-MAX_Experiment-3577-Airyscan Processing-01.png]

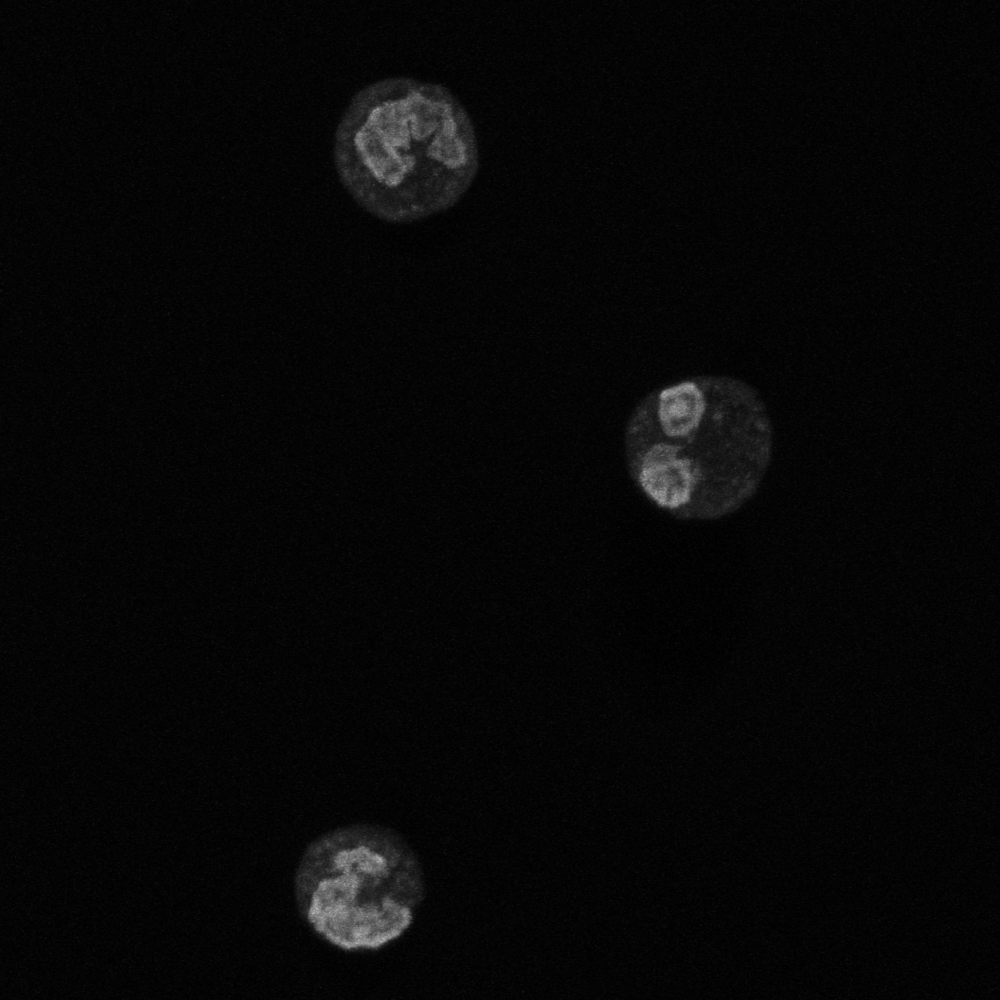

Supplement: Supplementary file 9 — EV Figures Source Data [file 44319_2024_150_MOESM9_ESM.zip › Figure EV5/Fig S5A/rRNA Y10b-AF647 LL37-dylight550/C3-MAX_Experiment-3577-Airyscan Processing-01.png]

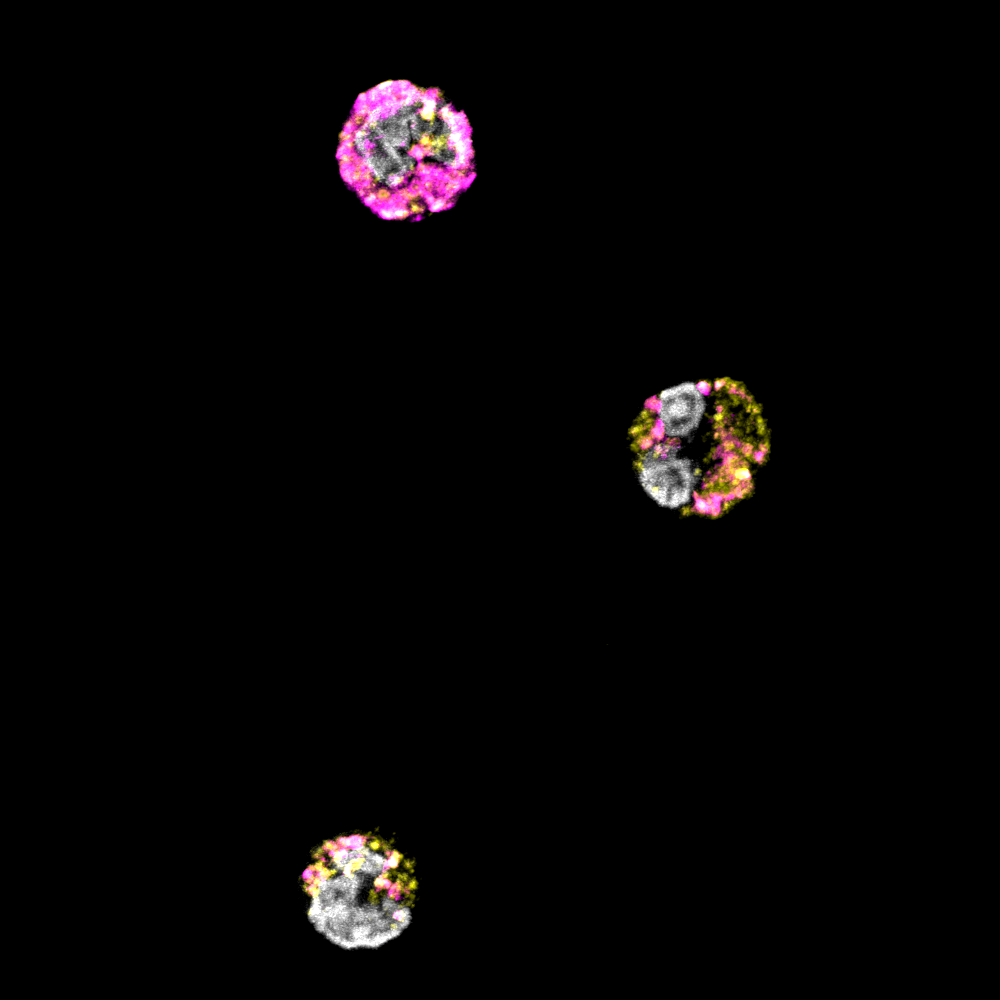

Supplement: Supplementary file 9 — EV Figures Source Data [file 44319_2024_150_MOESM9_ESM.zip › Figure EV5/Fig S5A/rRNA Y10b-AF647 LL37-dylight550/MAX_Experiment-3577-Airyscan Processing-01.png]

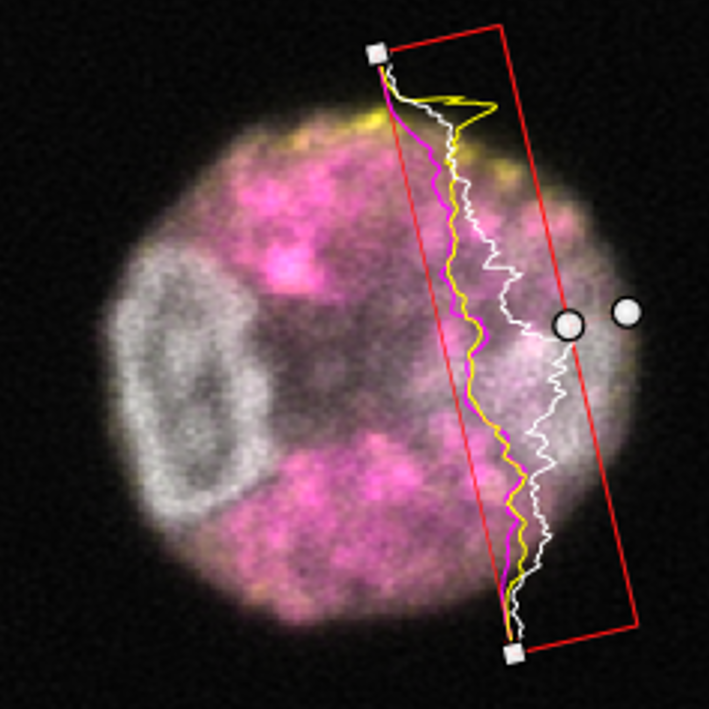

Supplement: Supplementary file 9 — EV Figures Source Data [file 44319_2024_150_MOESM9_ESM.zip › Figure EV5/Fig S5B/images/unstim 2 cropped.png]

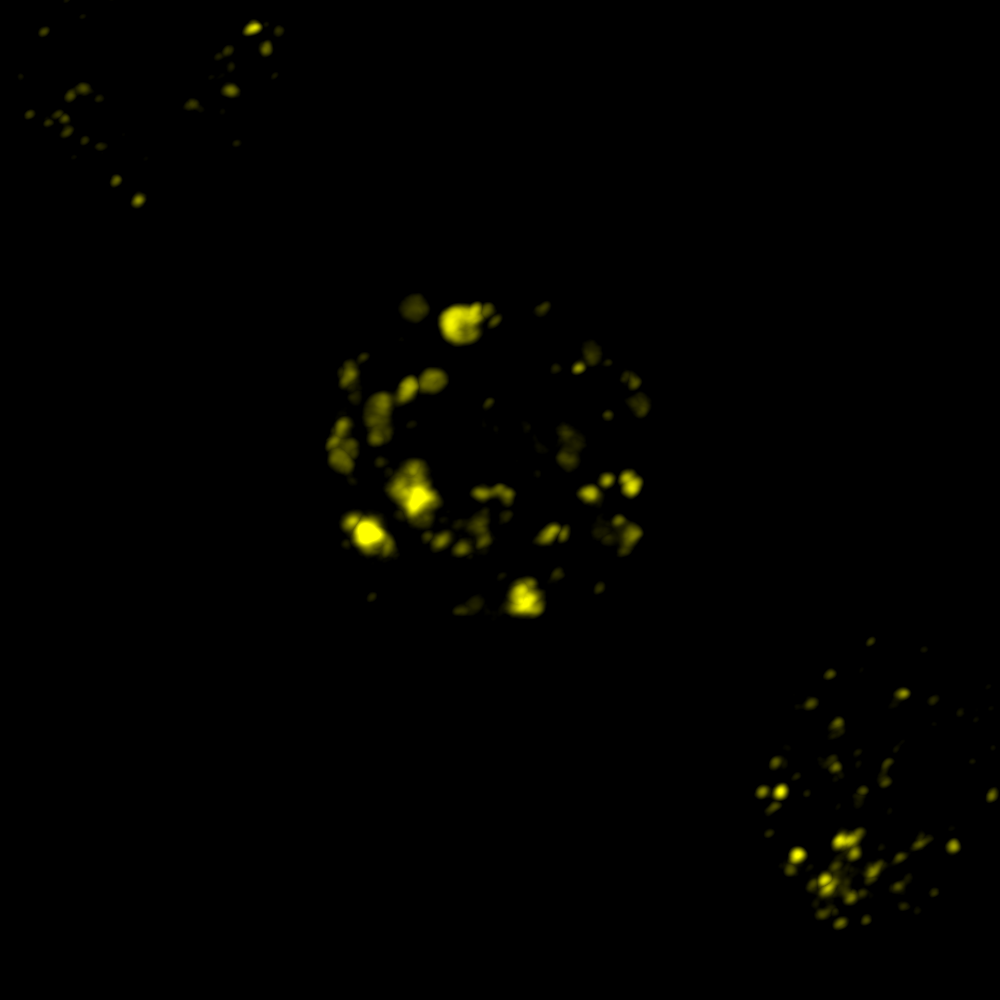

Supplement: Supplementary file 9 — EV Figures Source Data [file 44319_2024_150_MOESM9_ESM.zip › Figure EV5/Fig S5C-D/Close-up image/C1-MAX_Experiment-568-Airyscan Processing-36.png]

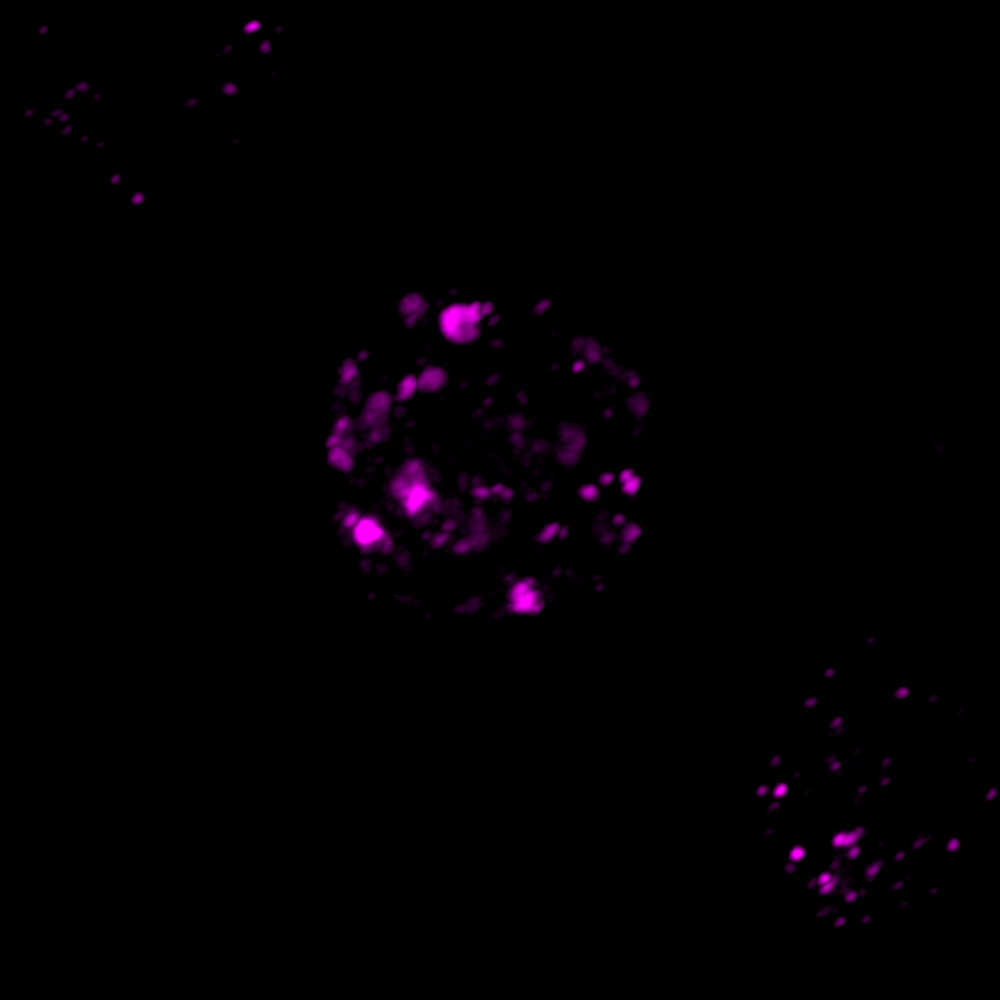

Supplement: Supplementary file 9 — EV Figures Source Data [file 44319_2024_150_MOESM9_ESM.zip › Figure EV5/Fig S5C-D/Close-up image/C2-MAX_Experiment-568-Airyscan Processing-36.png]

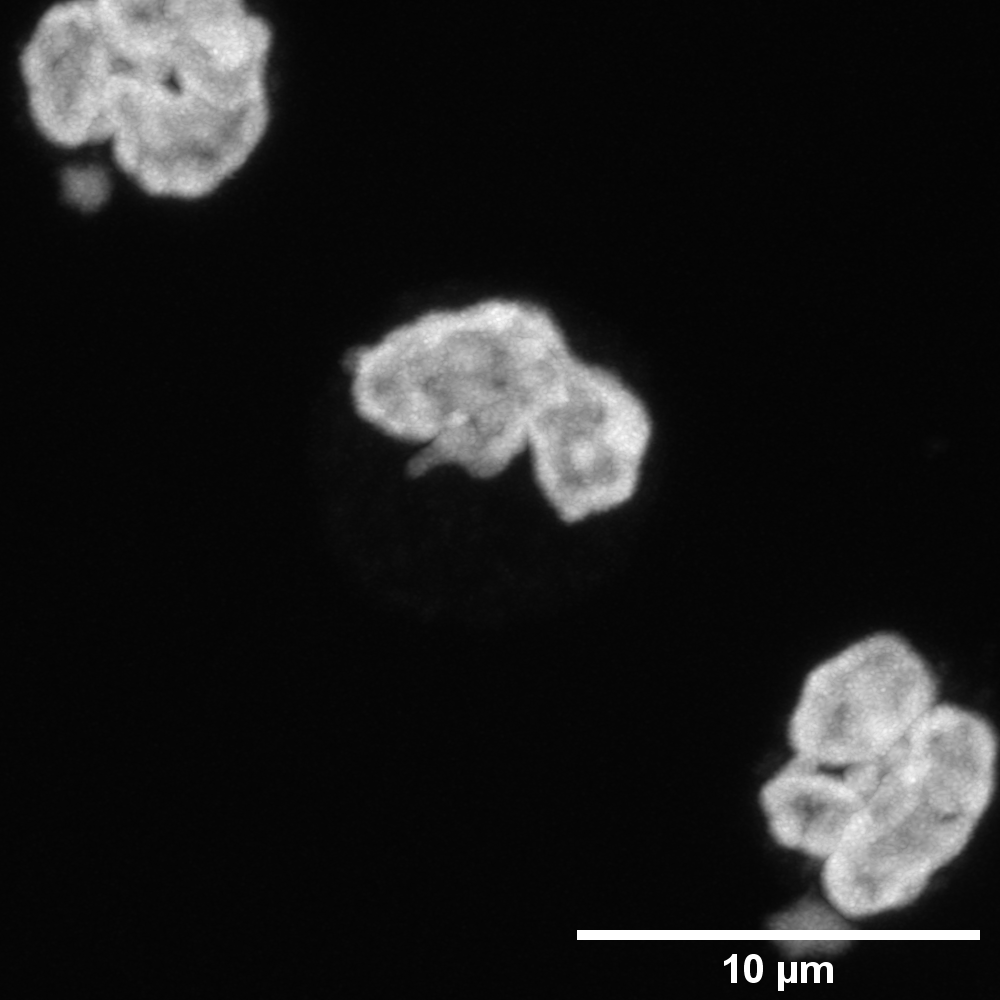

Supplement: Supplementary file 9 — EV Figures Source Data [file 44319_2024_150_MOESM9_ESM.zip › Figure EV5/Fig S5C-D/Close-up image/C3-MAX_Experiment-568-Airyscan Processing-36.png]

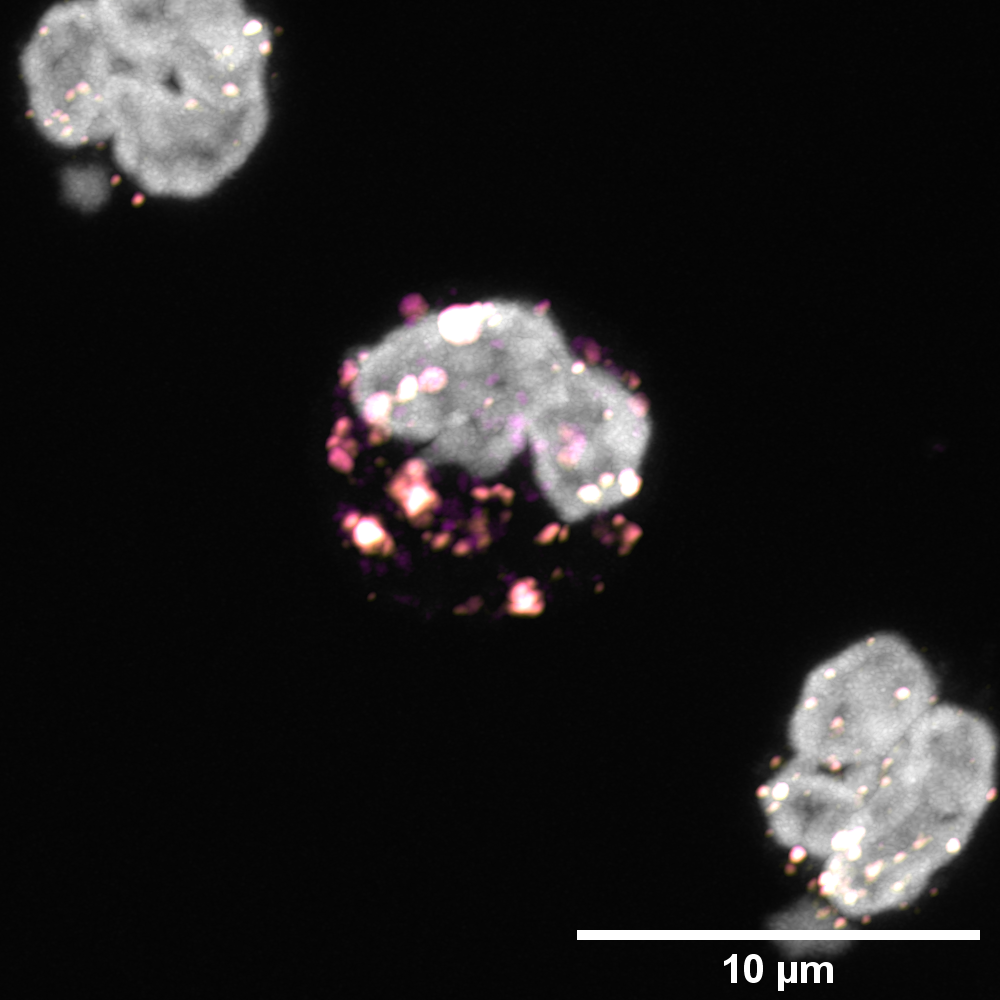

Supplement: Supplementary file 9 — EV Figures Source Data [file 44319_2024_150_MOESM9_ESM.zip › Figure EV5/Fig S5C-D/Close-up image/MAX_Experiment-568-Airyscan Processing-36.png]

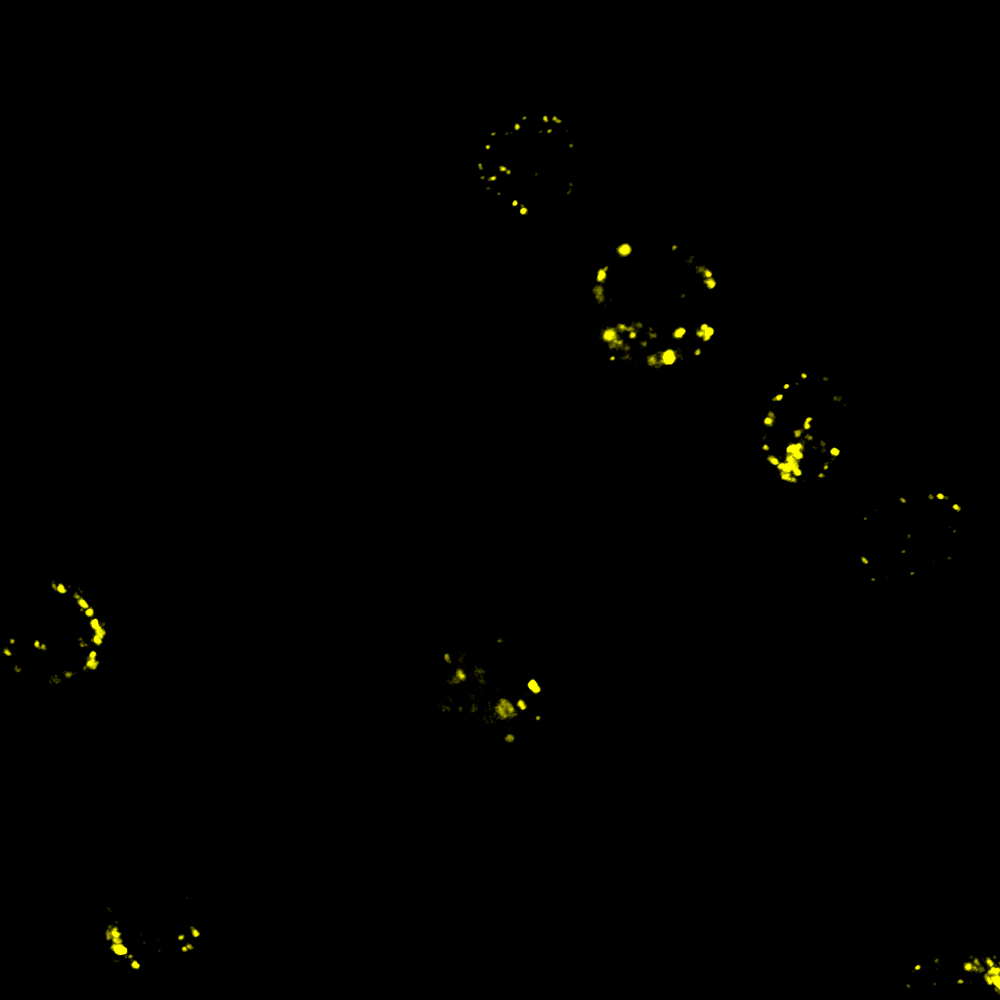

Supplement: Supplementary file 9 — EV Figures Source Data [file 44319_2024_150_MOESM9_ESM.zip › Figure EV5/Fig S5C-D/Overview image/C1-MAX_Experiment-567-Airyscan Processing-35.png]

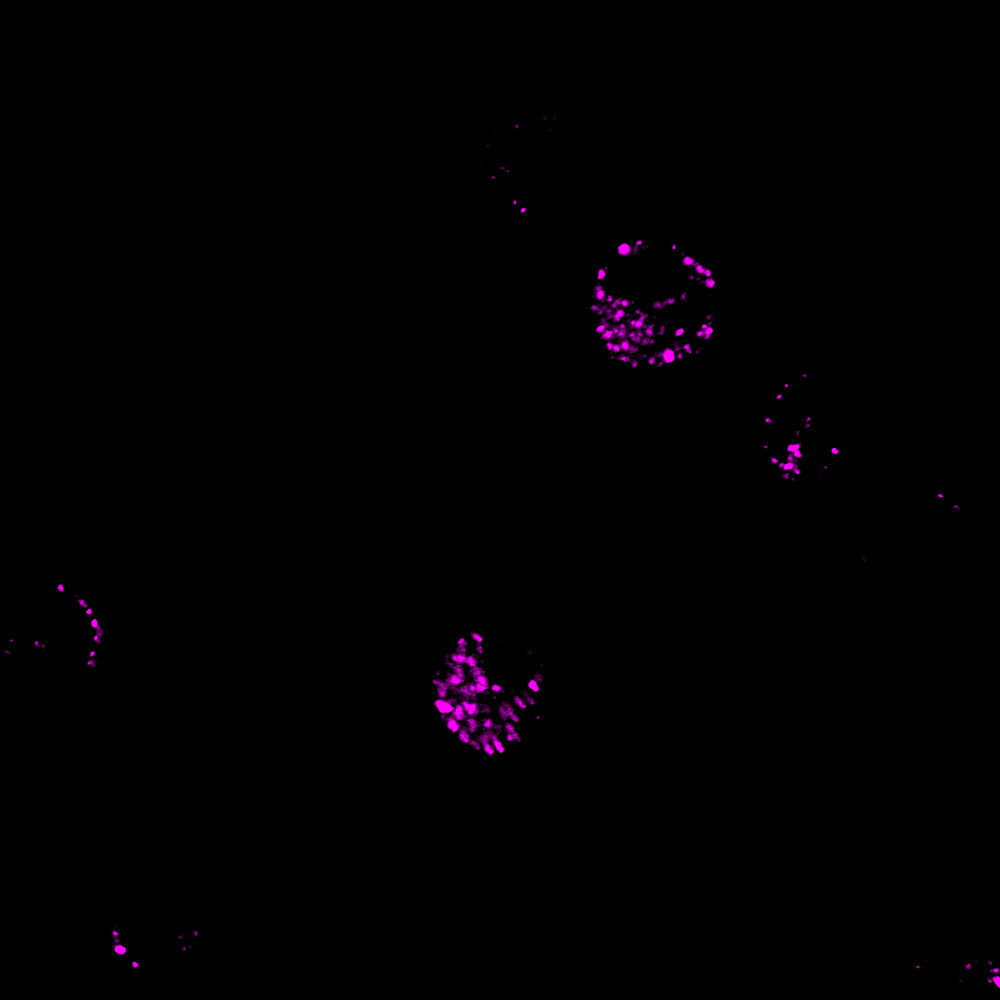

Supplement: Supplementary file 9 — EV Figures Source Data [file 44319_2024_150_MOESM9_ESM.zip › Figure EV5/Fig S5C-D/Overview image/C2-MAX_Experiment-567-Airyscan Processing-35.png]

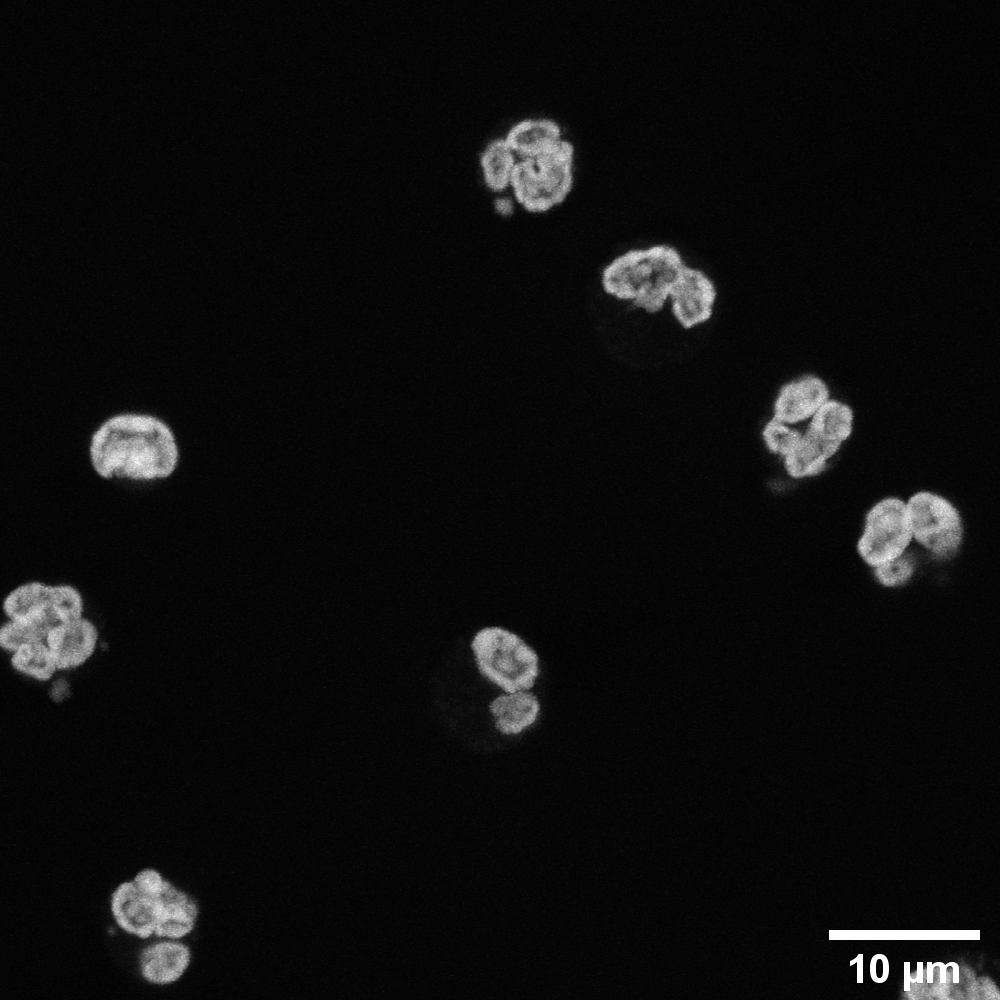

Supplement: Supplementary file 9 — EV Figures Source Data [file 44319_2024_150_MOESM9_ESM.zip › Figure EV5/Fig S5C-D/Overview image/C3-MAX_Experiment-567-Airyscan Processing-35.png]

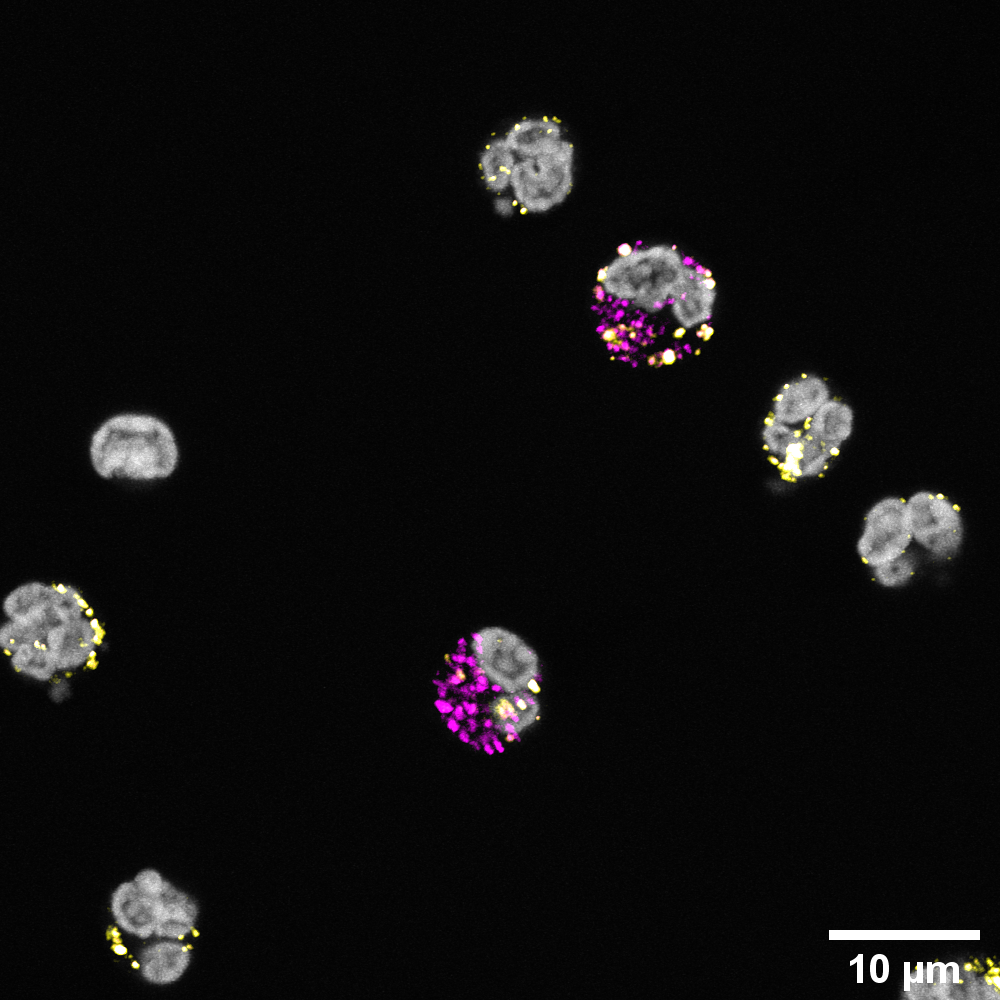

Supplement: Supplementary file 9 — EV Figures Source Data [file 44319_2024_150_MOESM9_ESM.zip › Figure EV5/Fig S5C-D/Overview image/MAX_Experiment-567-Airyscan Processing-35.png]

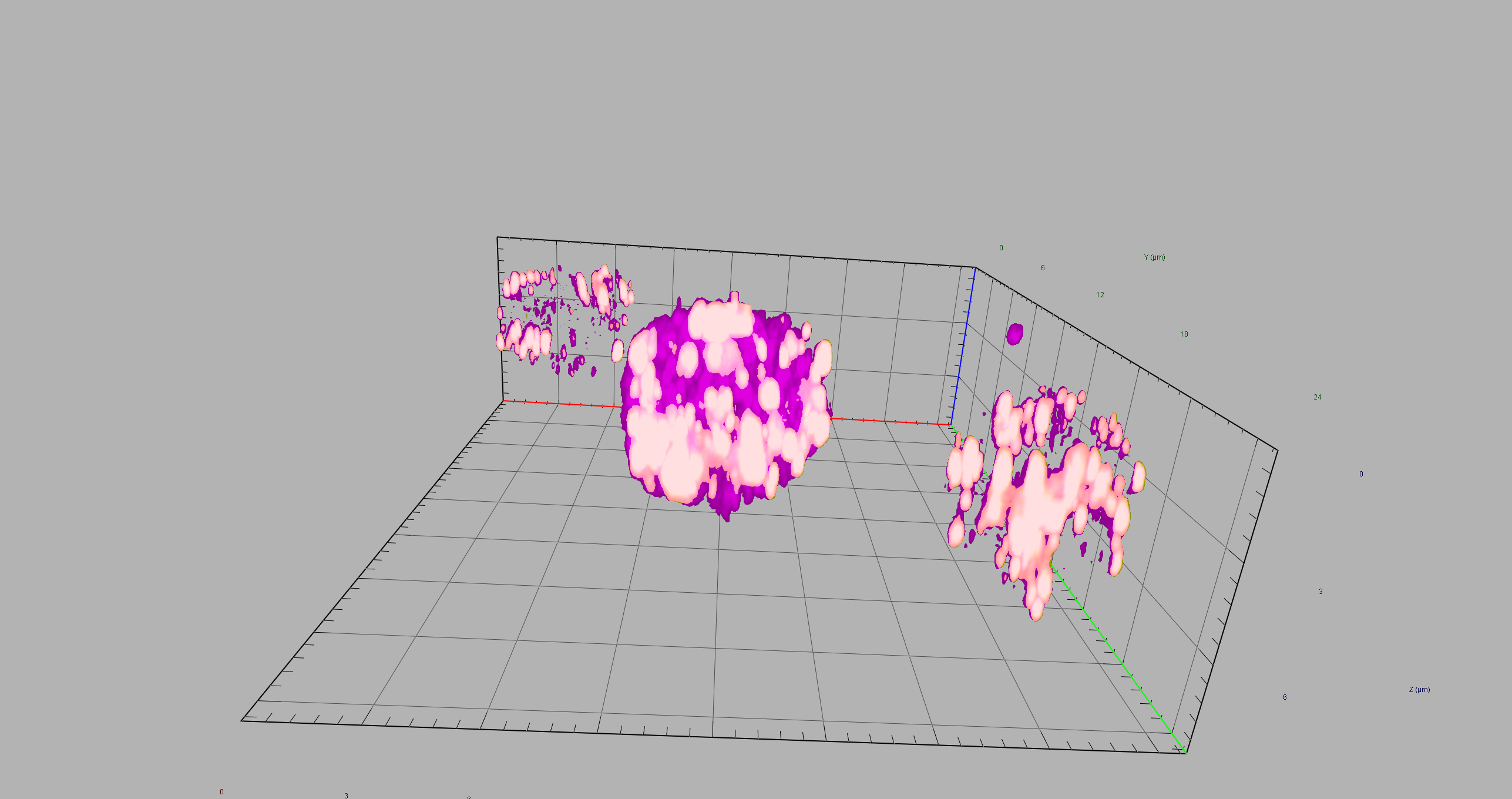

Supplement: Supplementary file 9 — EV Figures Source Data [file 44319_2024_150_MOESM9_ESM.zip › Figure EV5/Fig S5E/Experiment-568-Airyscan Processing-36 (Snapshot 1).tif]

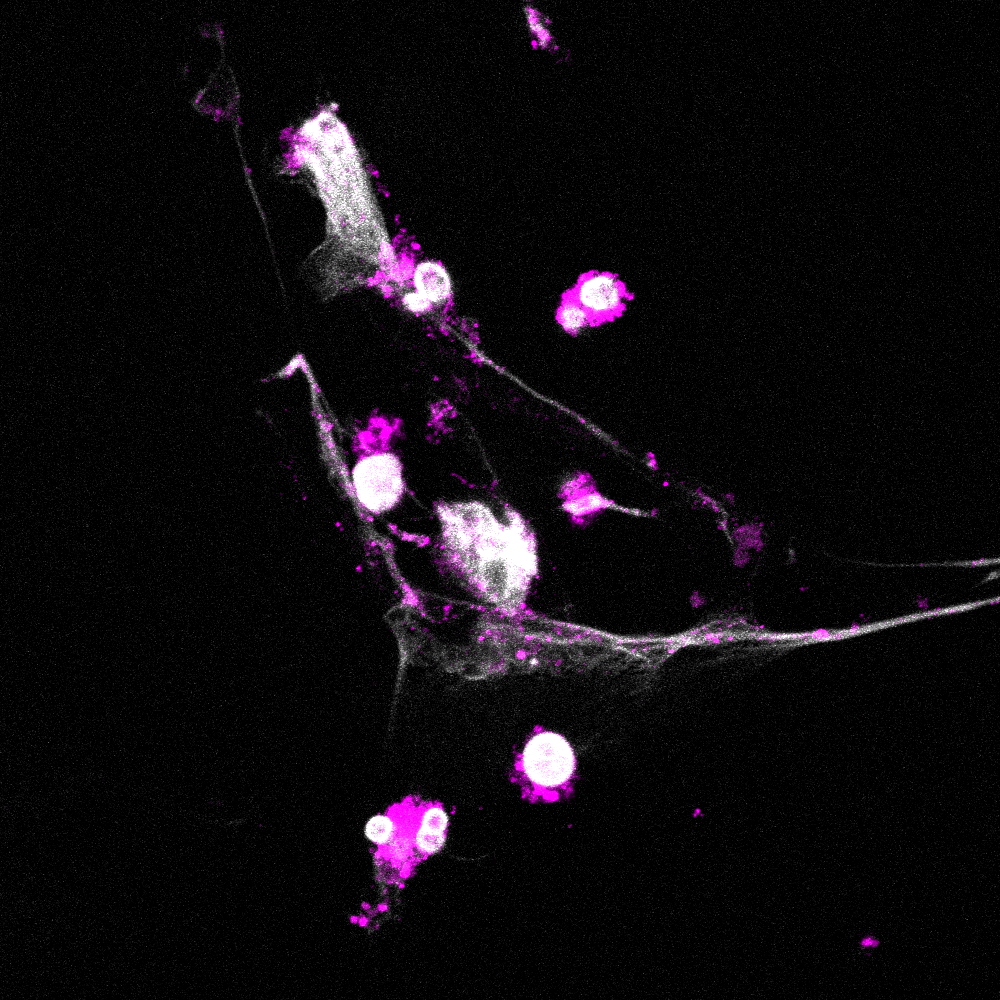

Supplement: Supplementary file 9 — EV Figures Source Data [file 44319_2024_150_MOESM9_ESM.zip › Figure EV5/Fig S5F/Close-up 30 min Composite.png]

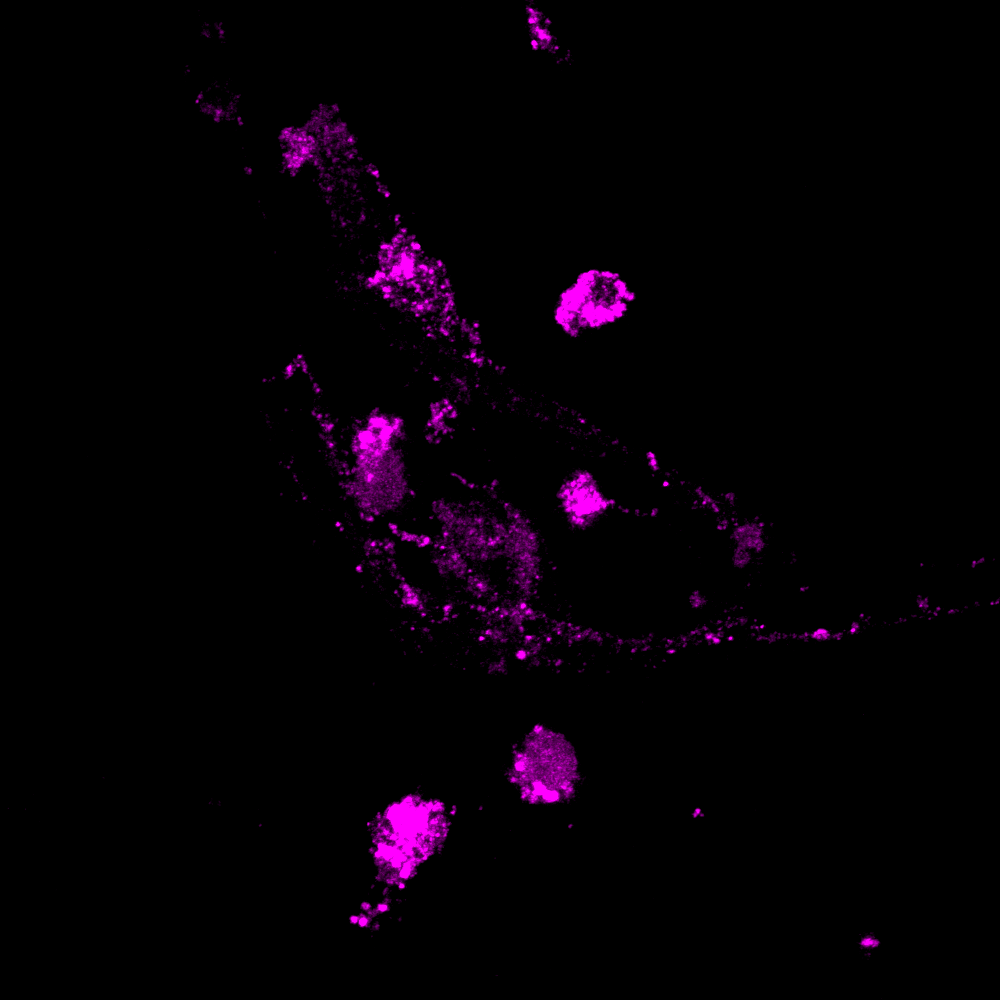

Supplement: Supplementary file 9 — EV Figures Source Data [file 44319_2024_150_MOESM9_ESM.zip › Figure EV5/Fig S5F/Close-up 30 min Hoechst.png]

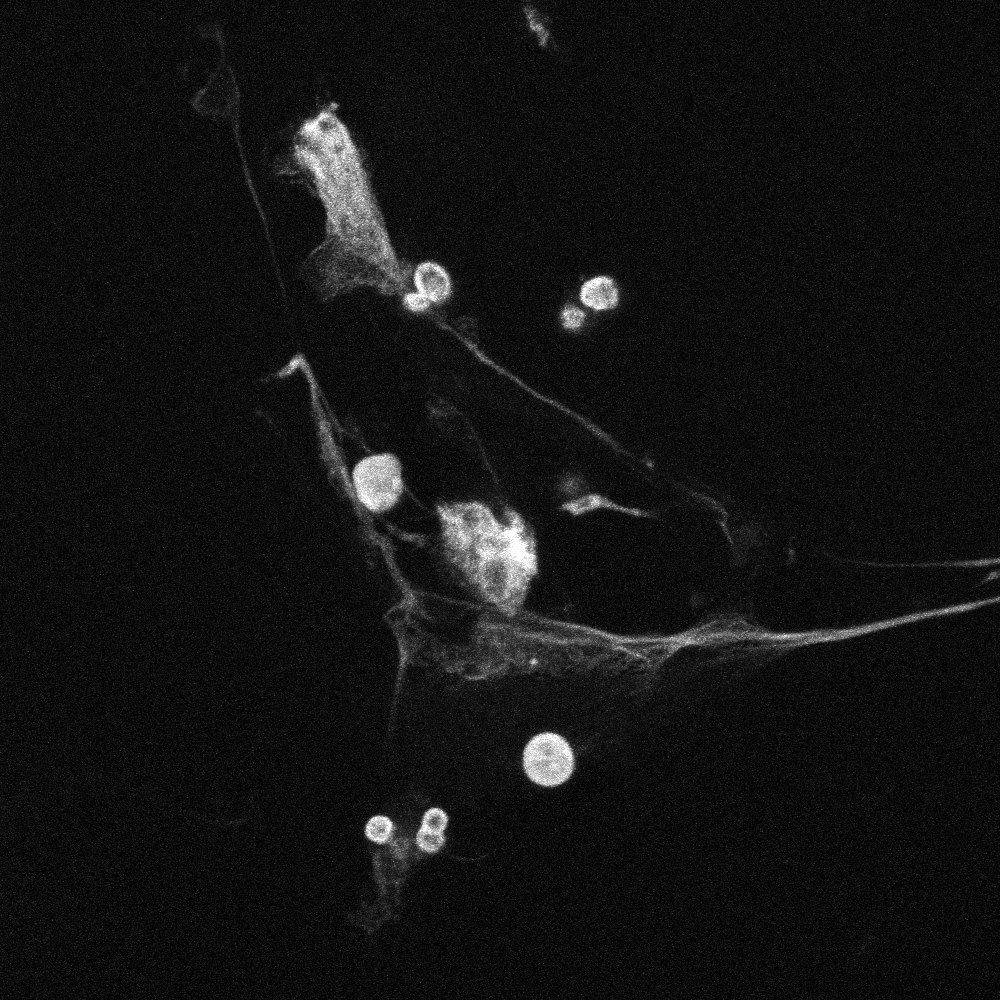

Supplement: Supplementary file 9 — EV Figures Source Data [file 44319_2024_150_MOESM9_ESM.zip › Figure EV5/Fig S5F/Close-up 30 min rRNA.png]

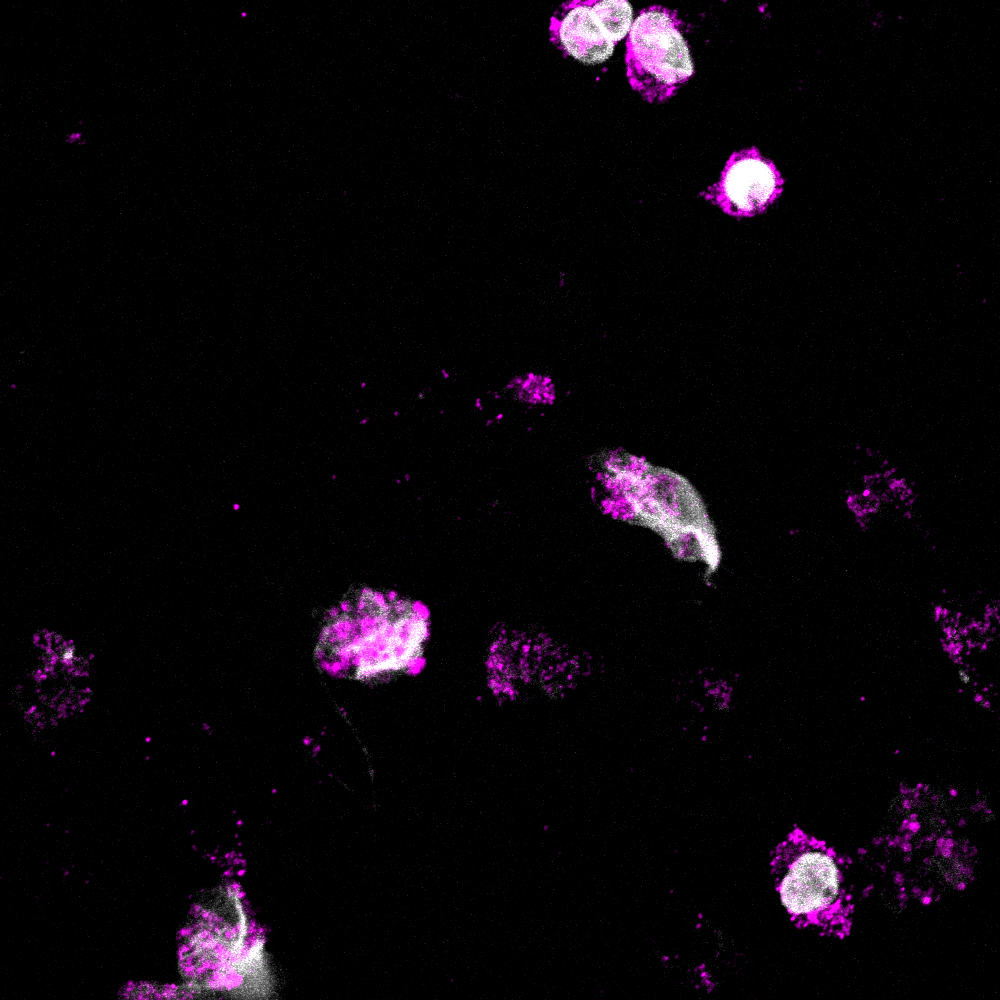

Supplement: Supplementary file 9 — EV Figures Source Data [file 44319_2024_150_MOESM9_ESM.zip › Figure EV5/Fig S5F/Close-up 4 h Composite.png]

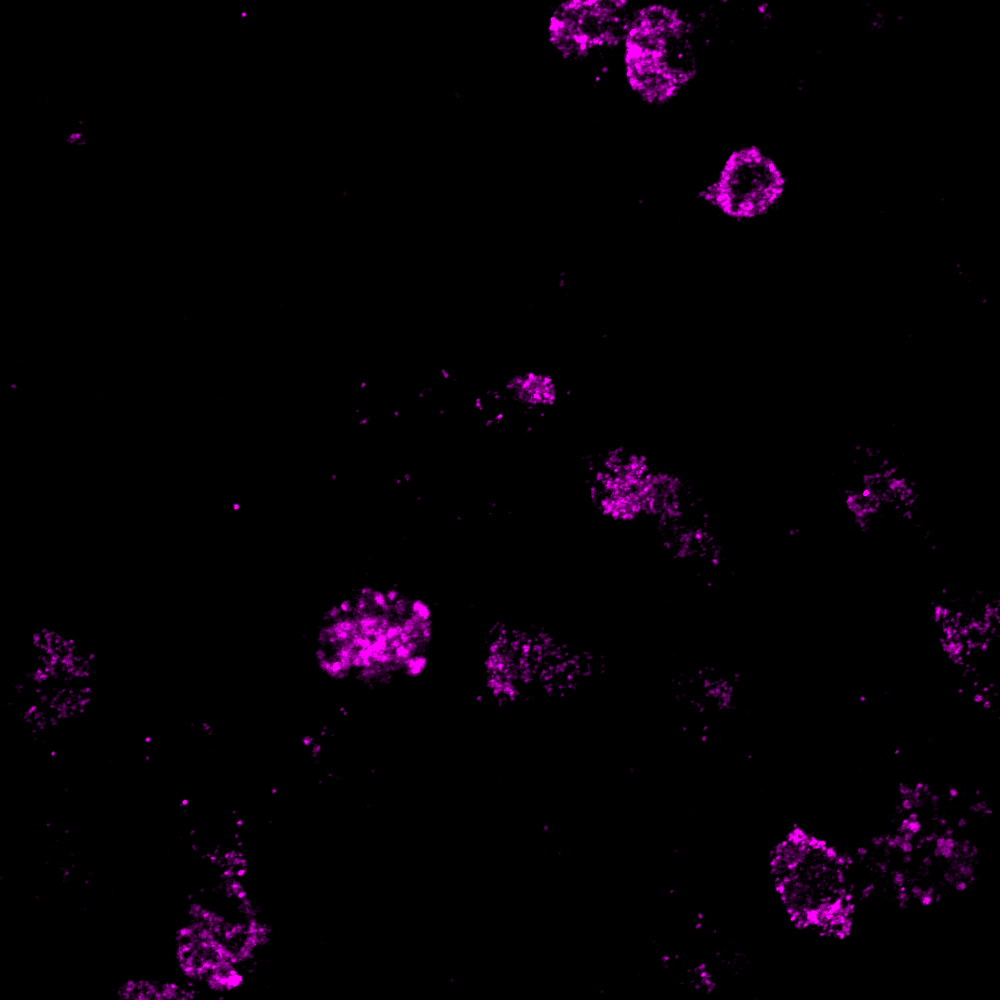

Supplement: Supplementary file 9 — EV Figures Source Data [file 44319_2024_150_MOESM9_ESM.zip › Figure EV5/Fig S5F/Close-up 4 h Hoechst.png]

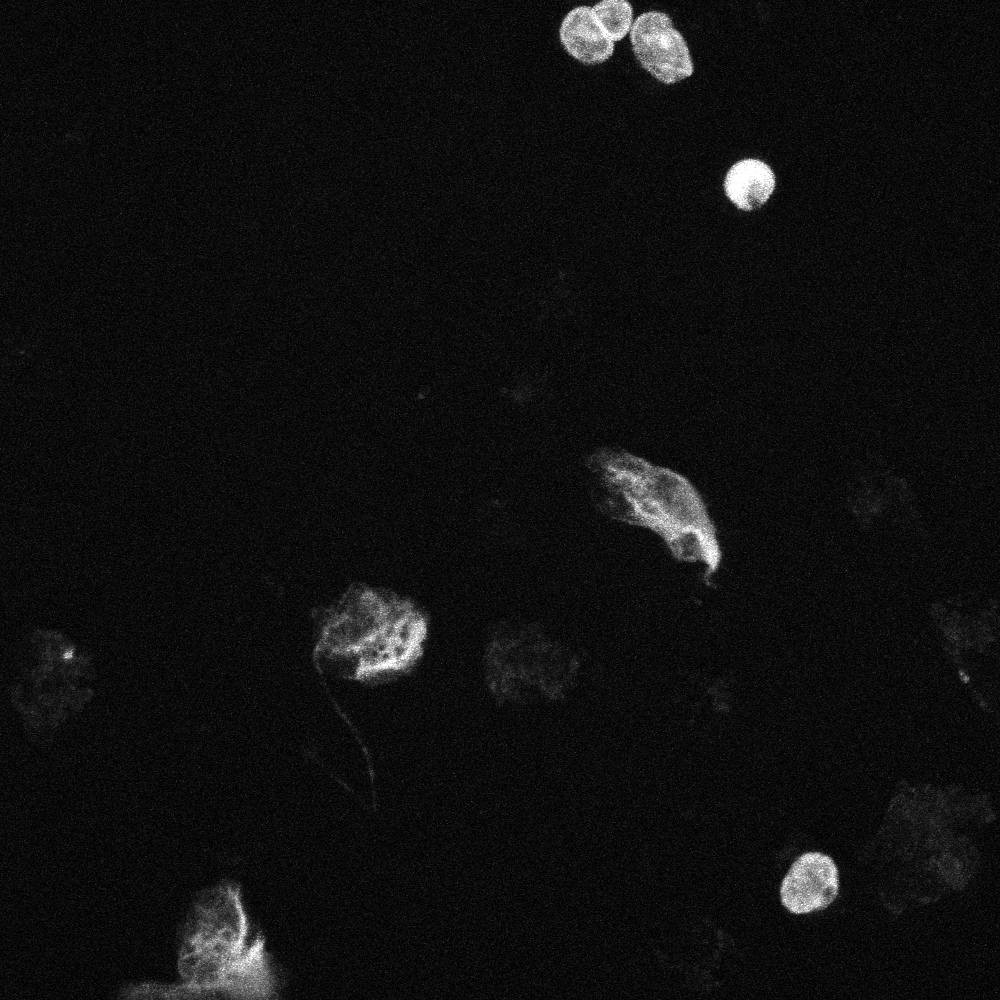

Supplement: Supplementary file 9 — EV Figures Source Data [file 44319_2024_150_MOESM9_ESM.zip › Figure EV5/Fig S5F/Close-up 4 h rRNA.png]

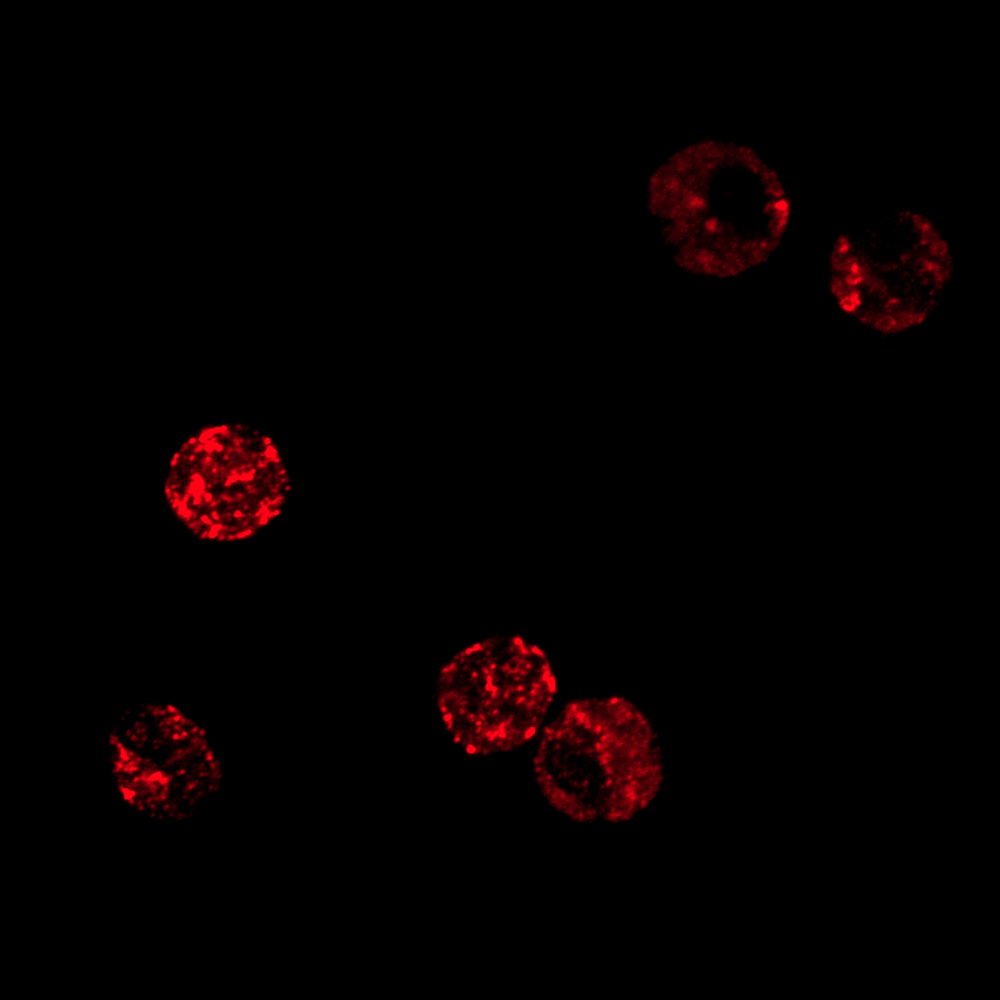

Supplement: Supplementary file 9 — EV Figures Source Data [file 44319_2024_150_MOESM9_ESM.zip › Figure EV3/Fig S3A/image FB-175/pad4/mock nets/C1-MAX_Experiment-3526-Airyscan Processing-16.png]

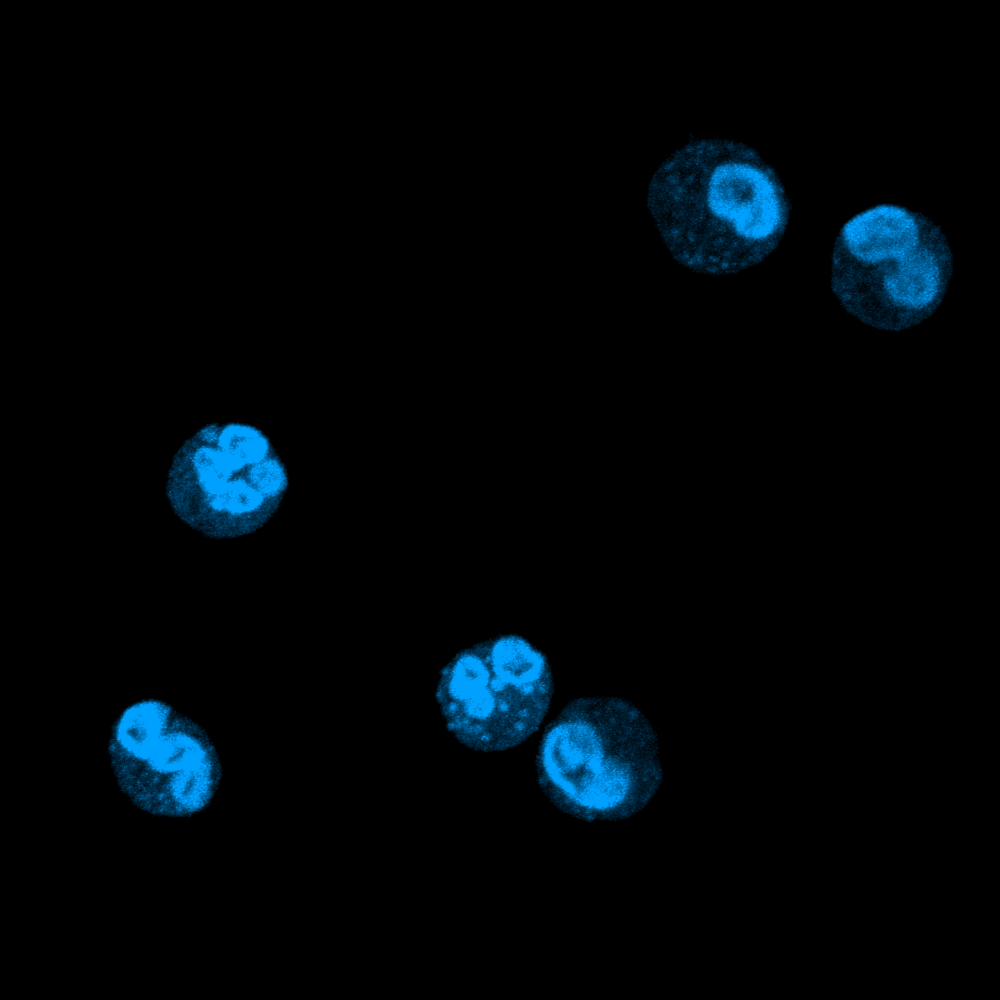

Supplement: Supplementary file 9 — EV Figures Source Data [file 44319_2024_150_MOESM9_ESM.zip › Figure EV3/Fig S3A/image FB-175/pad4/mock nets/C2-MAX_Experiment-3526-Airyscan Processing-16.png]

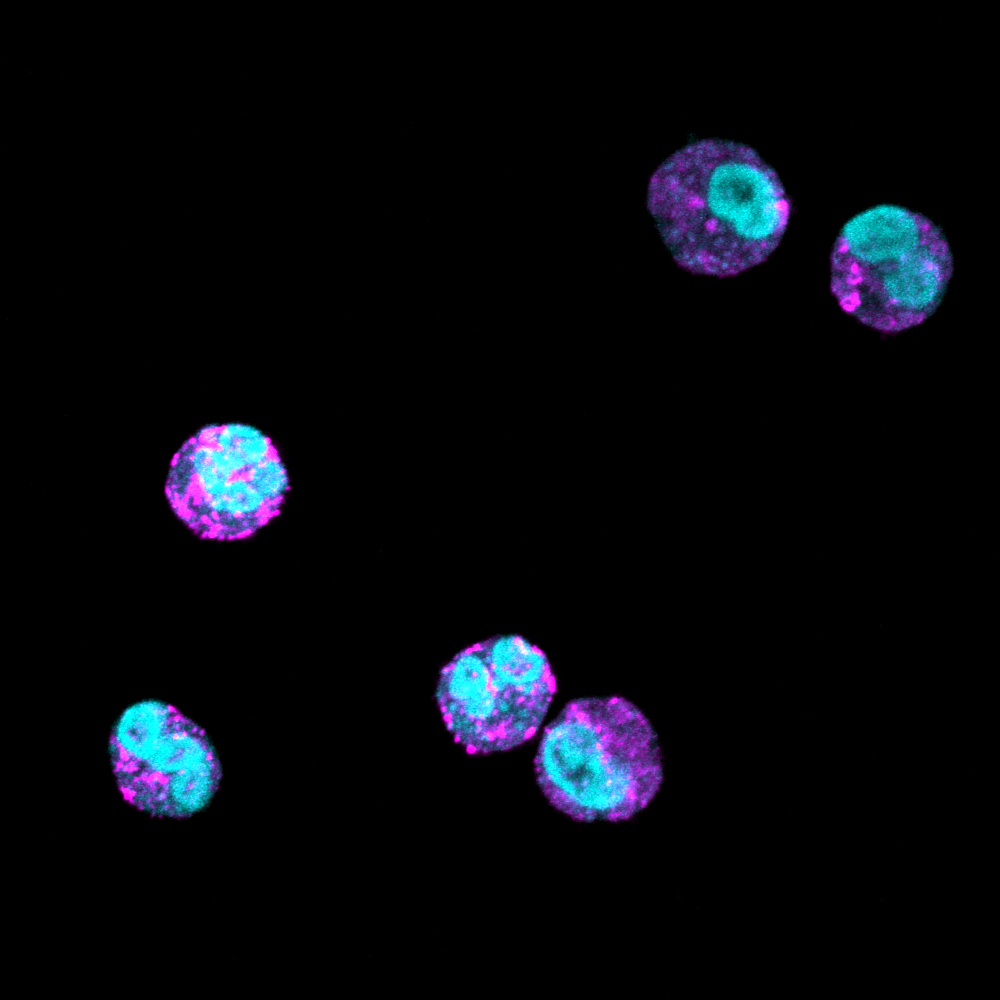

Supplement: Supplementary file 9 — EV Figures Source Data [file 44319_2024_150_MOESM9_ESM.zip › Figure EV3/Fig S3A/image FB-175/pad4/mock nets/comp new.png]

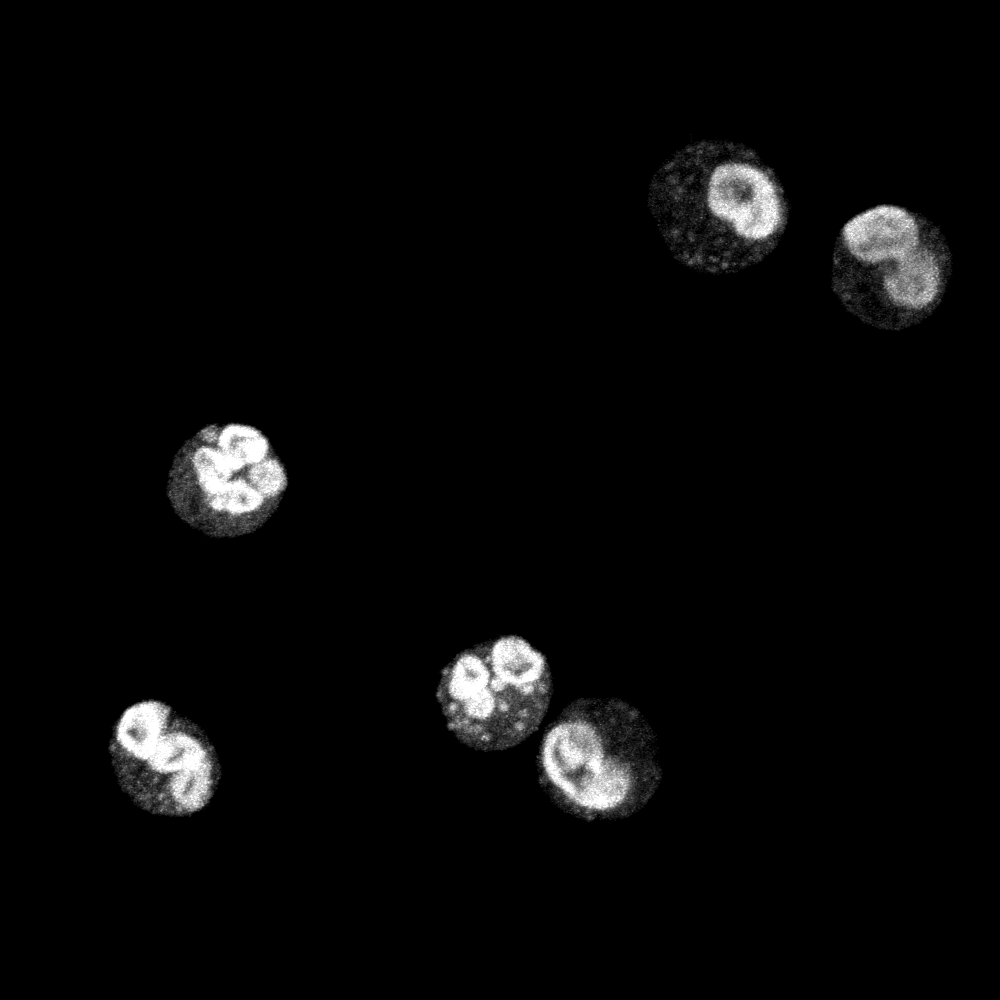

Supplement: Supplementary file 9 — EV Figures Source Data [file 44319_2024_150_MOESM9_ESM.zip › Figure EV3/Fig S3A/image FB-175/pad4/mock nets/gray.png]

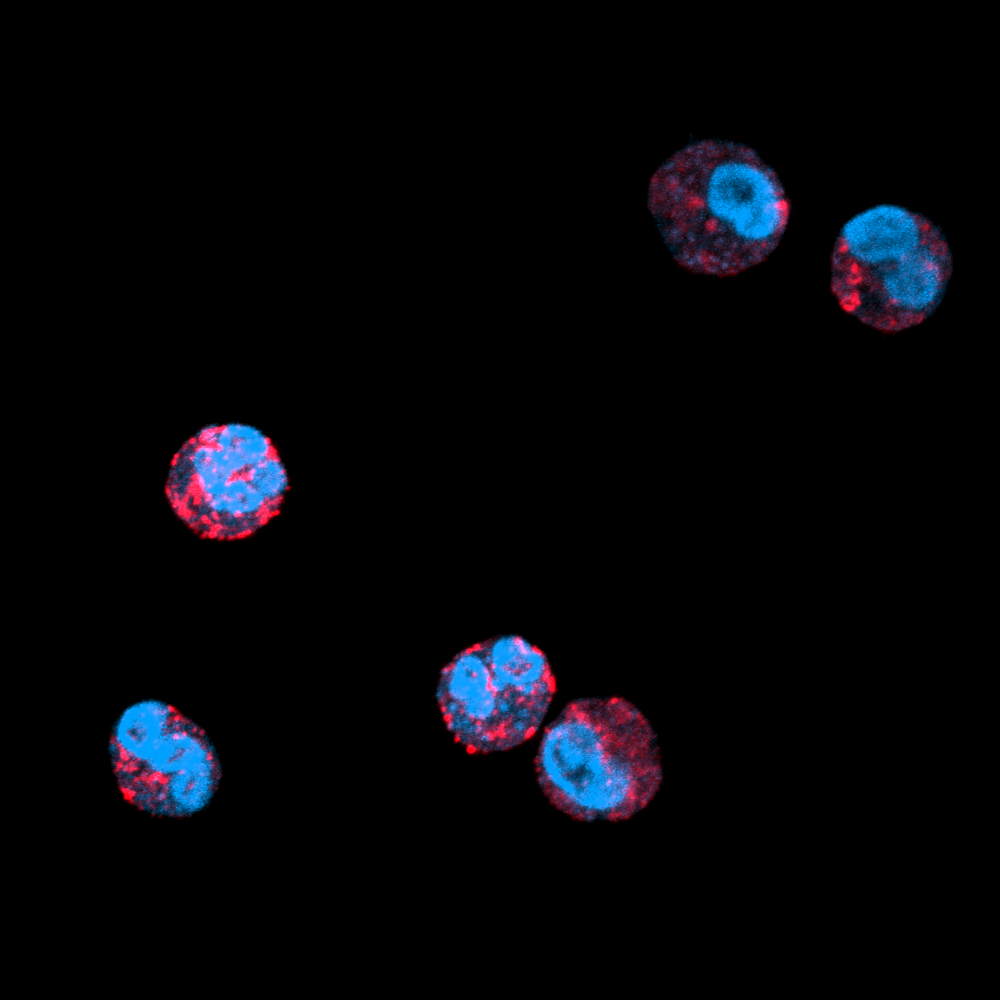

Supplement: Supplementary file 9 — EV Figures Source Data [file 44319_2024_150_MOESM9_ESM.zip › Figure EV3/Fig S3A/image FB-175/pad4/mock nets/MAX_Experiment-3526-Airyscan Processing-16.png]

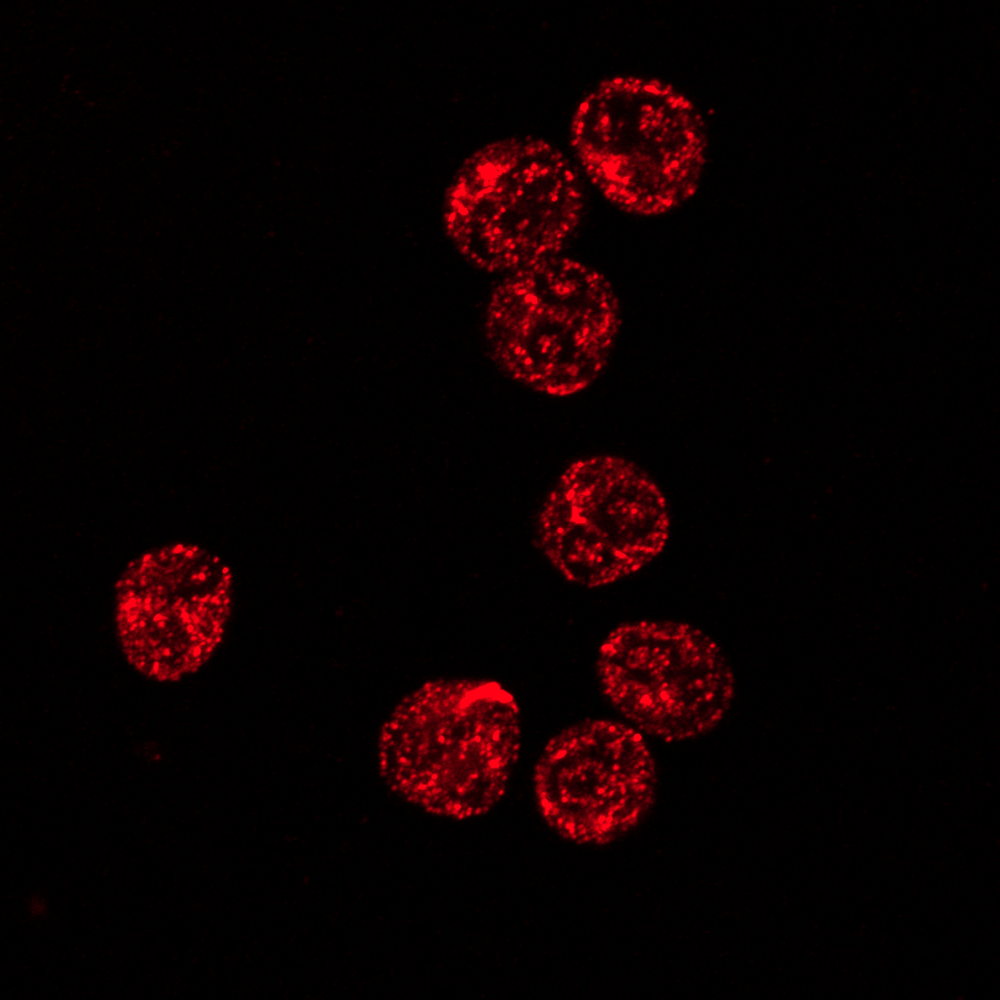

Supplement: Supplementary file 9 — EV Figures Source Data [file 44319_2024_150_MOESM9_ESM.zip › Figure EV3/Fig S3A/image FB-175/pad4/pma/C1-MAX_Experiment-3525-Airyscan Processing-15.png]

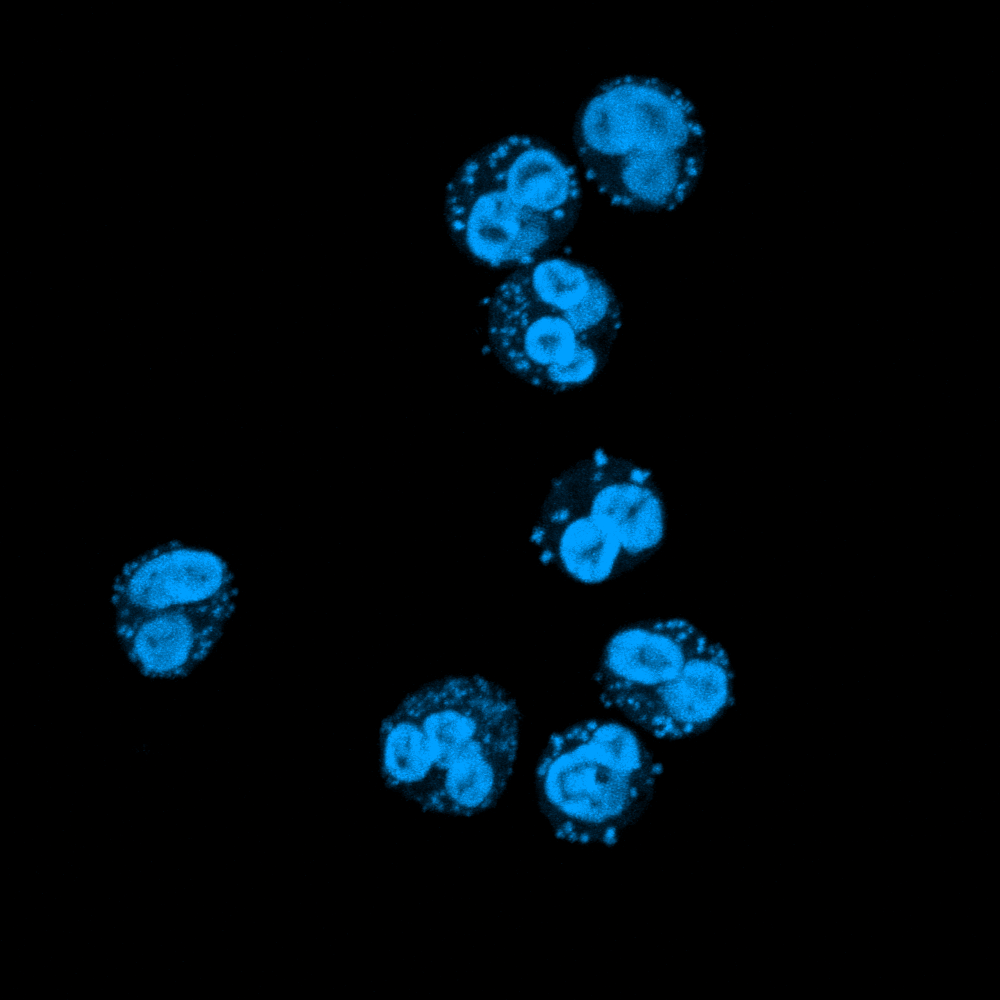

Supplement: Supplementary file 9 — EV Figures Source Data [file 44319_2024_150_MOESM9_ESM.zip › Figure EV3/Fig S3A/image FB-175/pad4/pma/C2-MAX_Experiment-3525-Airyscan Processing-15.png]

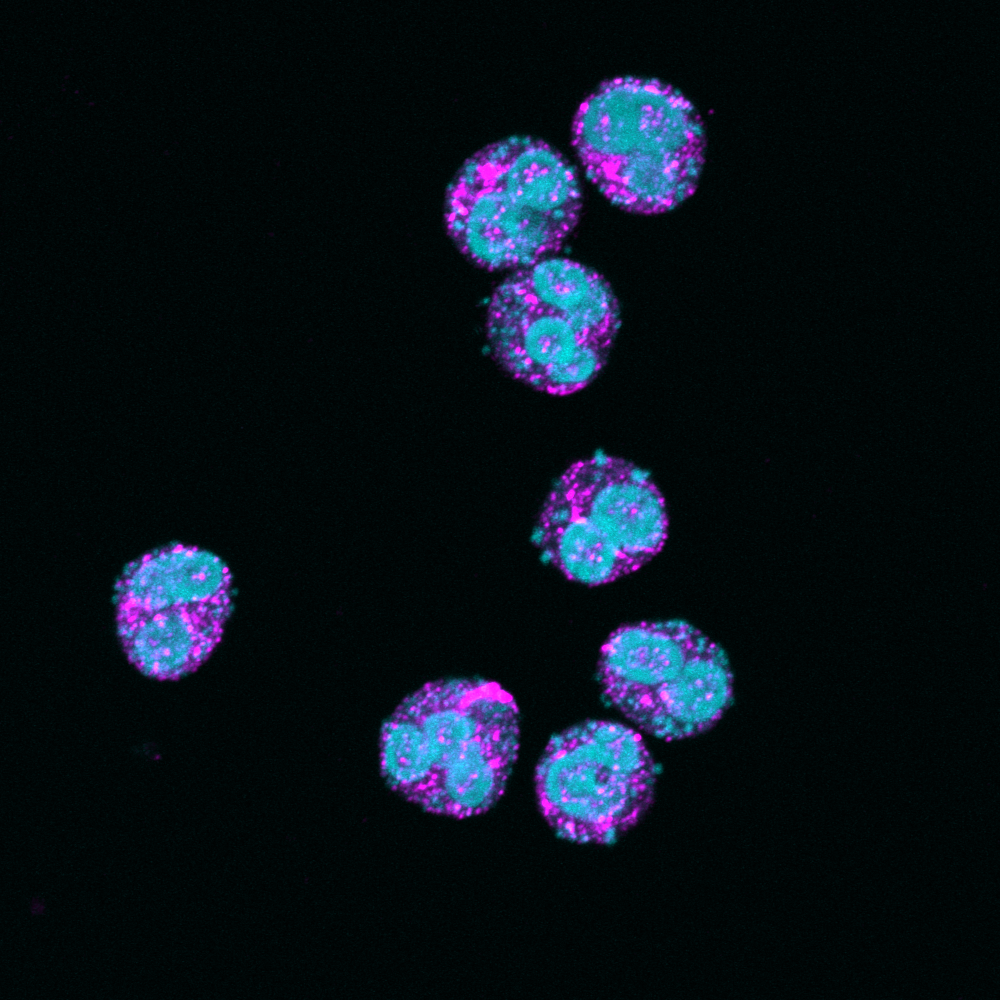

Supplement: Supplementary file 9 — EV Figures Source Data [file 44319_2024_150_MOESM9_ESM.zip › Figure EV3/Fig S3A/image FB-175/pad4/pma/comp new.png]

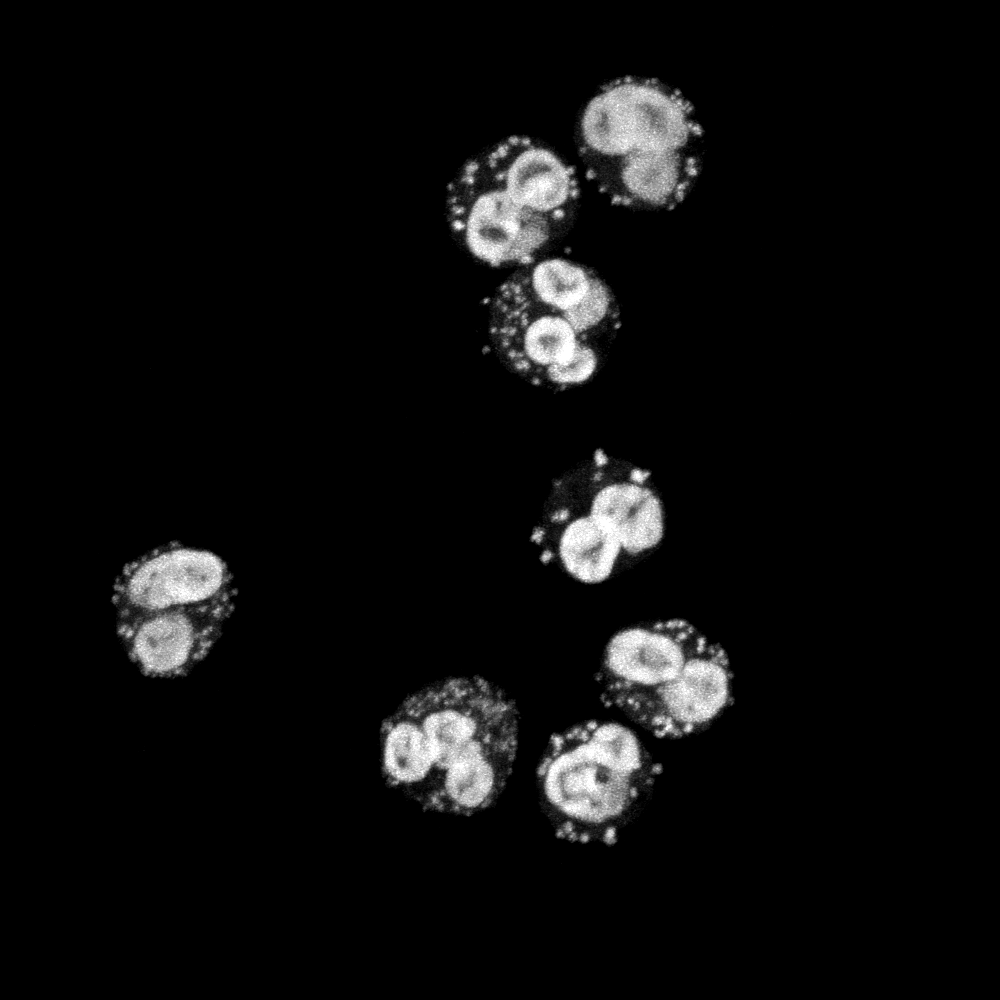

Supplement: Supplementary file 9 — EV Figures Source Data [file 44319_2024_150_MOESM9_ESM.zip › Figure EV3/Fig S3A/image FB-175/pad4/pma/gray.png]

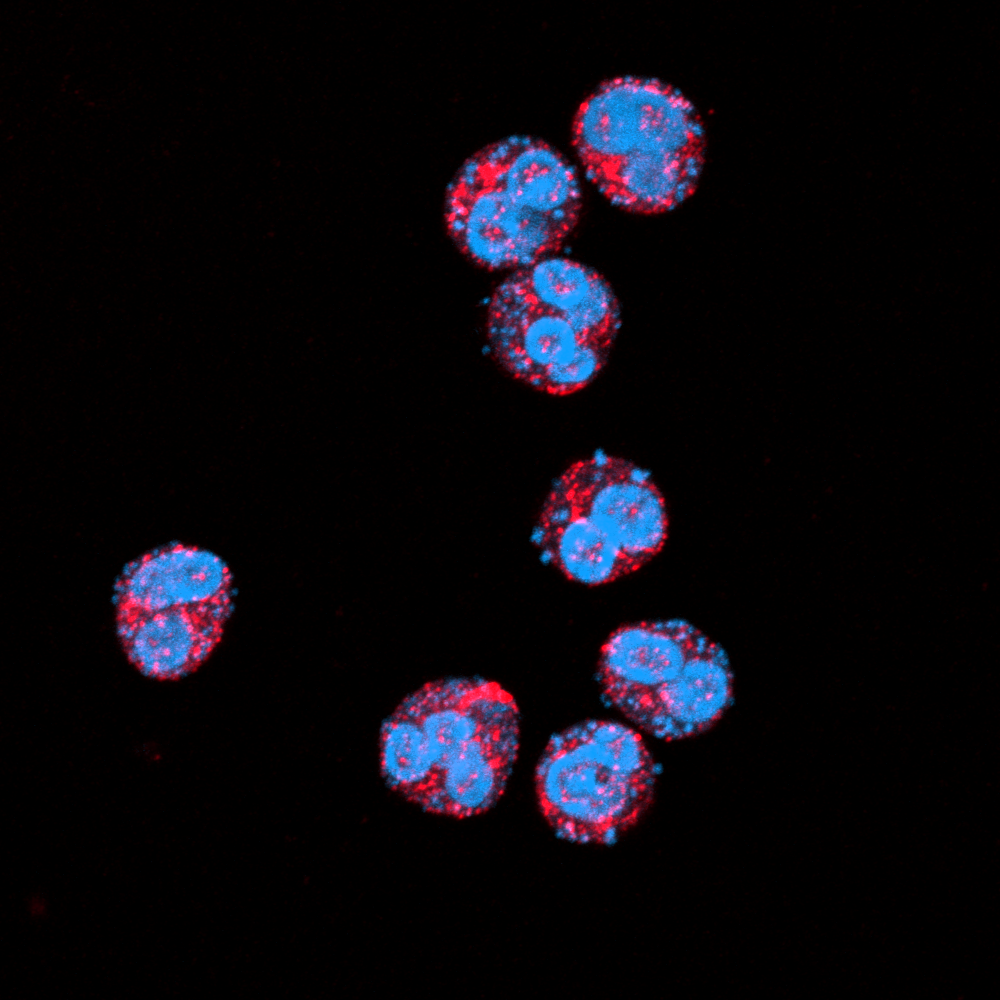

Supplement: Supplementary file 9 — EV Figures Source Data [file 44319_2024_150_MOESM9_ESM.zip › Figure EV3/Fig S3A/image FB-175/pad4/pma/MAX_Experiment-3525-Airyscan Processing-15.png]

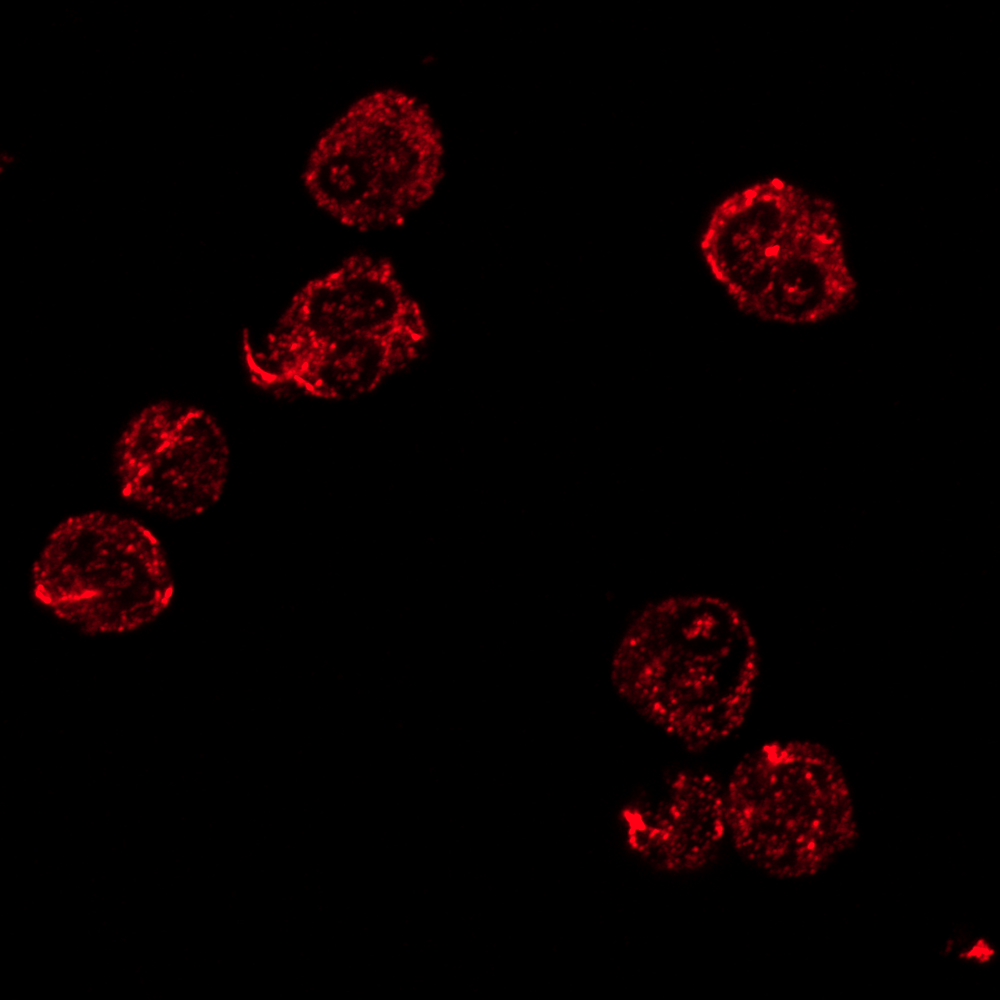

Supplement: Supplementary file 9 — EV Figures Source Data [file 44319_2024_150_MOESM9_ESM.zip › Figure EV3/Fig S3A/image FB-175/pad4/pma nets/C1-MAX_Experiment-3527-Airyscan Processing-17.png]

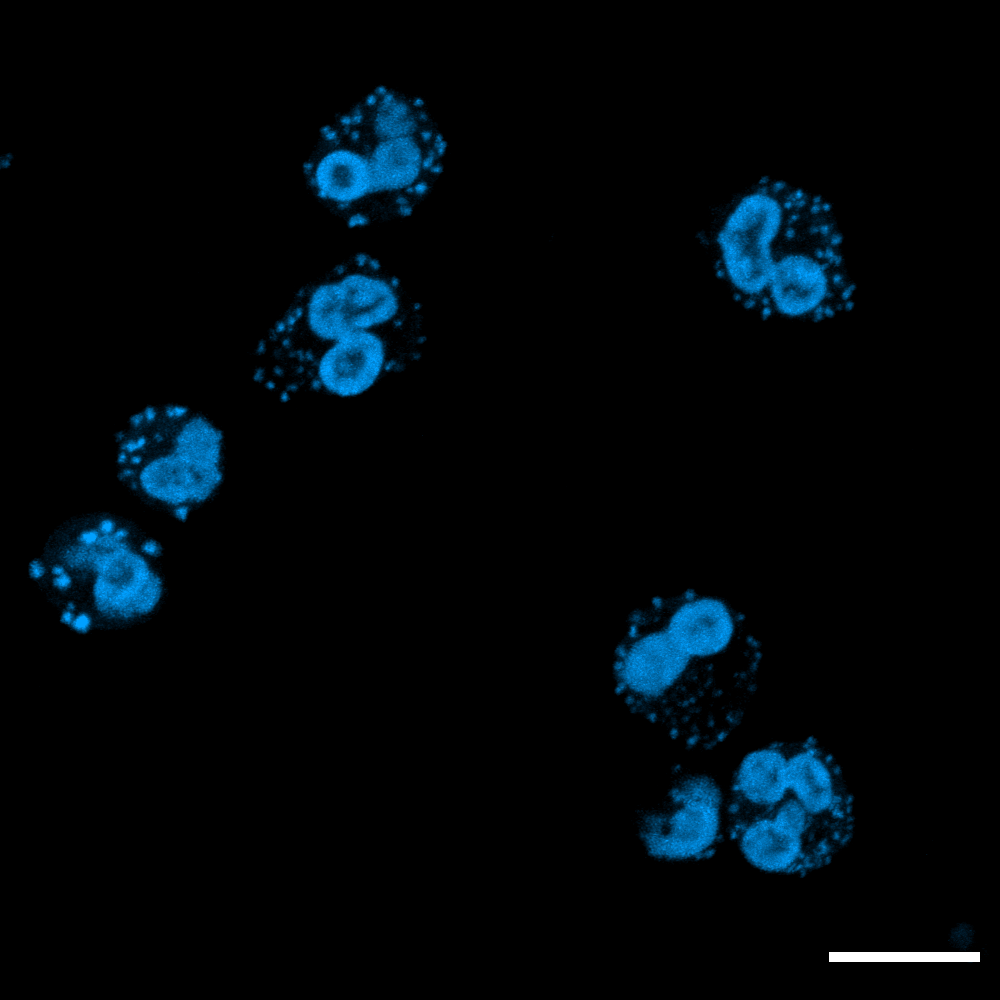

Supplement: Supplementary file 9 — EV Figures Source Data [file 44319_2024_150_MOESM9_ESM.zip › Figure EV3/Fig S3A/image FB-175/pad4/pma nets/C2-MAX_Experiment-3527-Airyscan Processing-17.png]

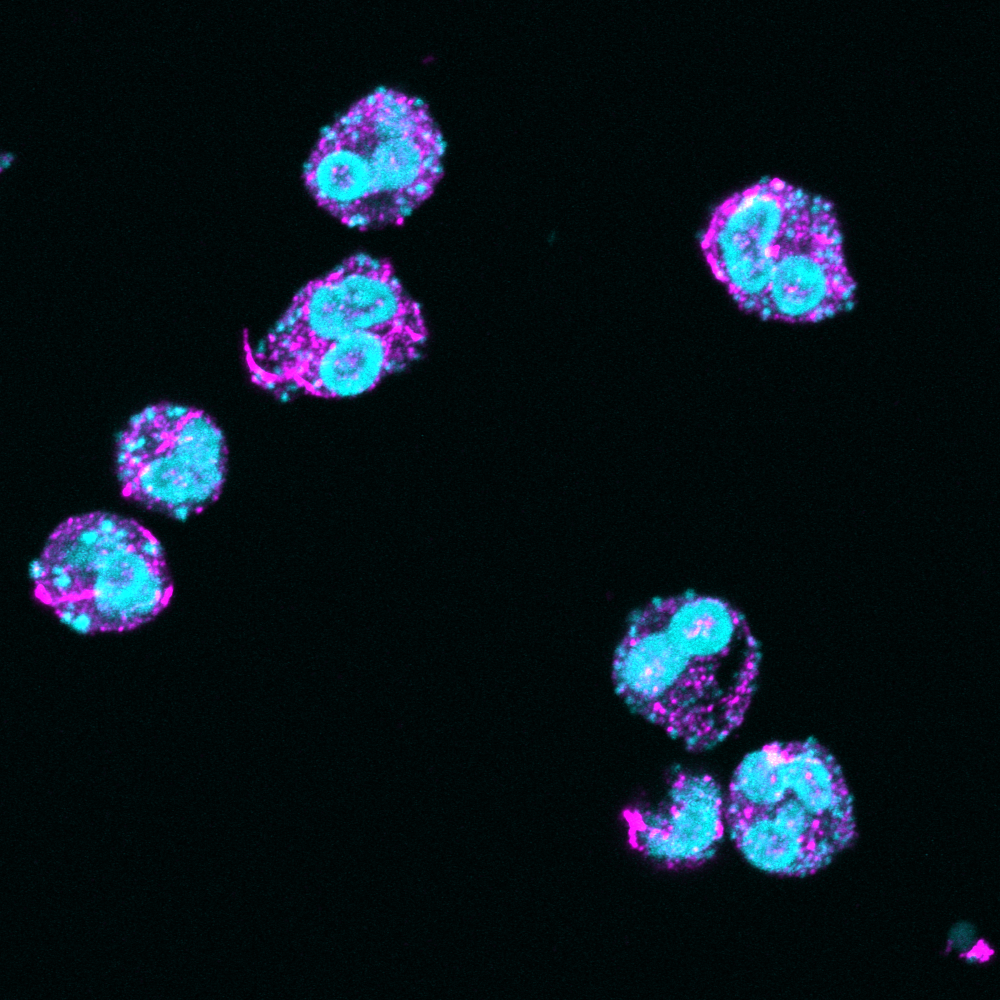

Supplement: Supplementary file 9 — EV Figures Source Data [file 44319_2024_150_MOESM9_ESM.zip › Figure EV3/Fig S3A/image FB-175/pad4/pma nets/comp new.png]

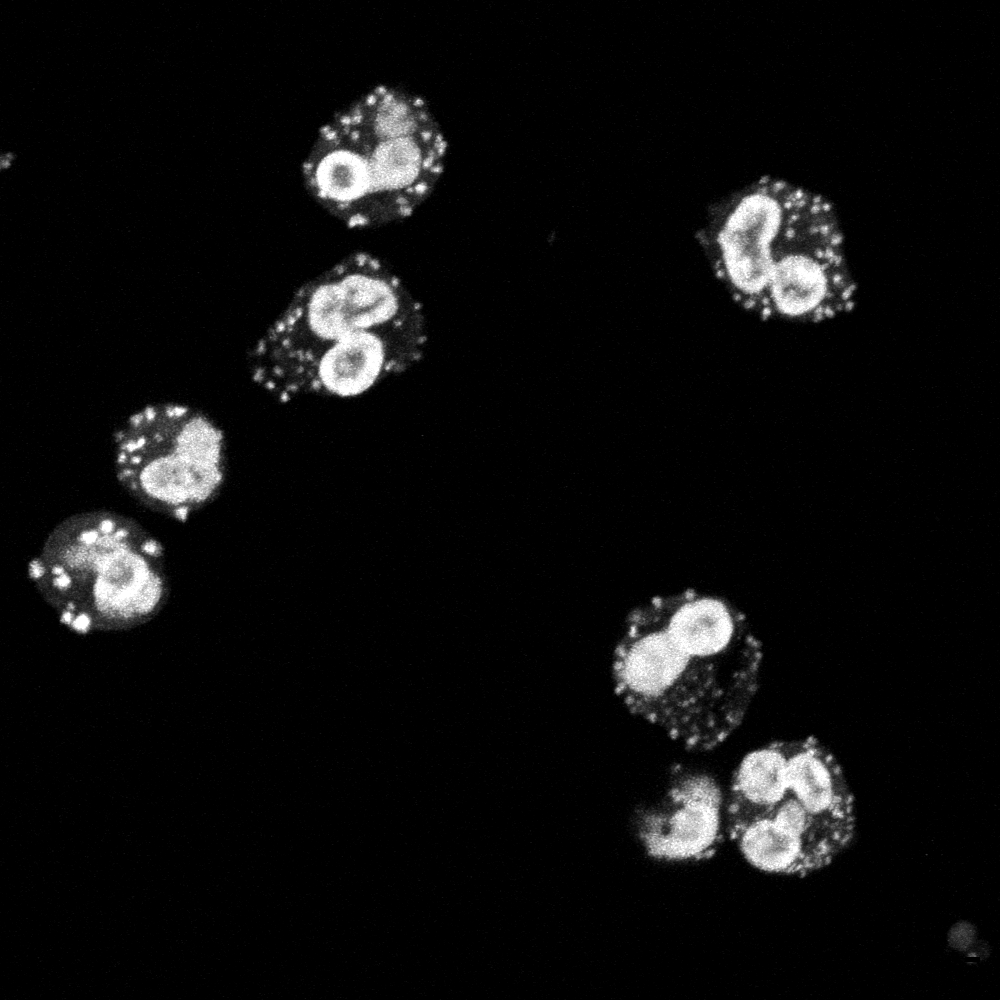

Supplement: Supplementary file 9 — EV Figures Source Data [file 44319_2024_150_MOESM9_ESM.zip › Figure EV3/Fig S3A/image FB-175/pad4/pma nets/gray.png]

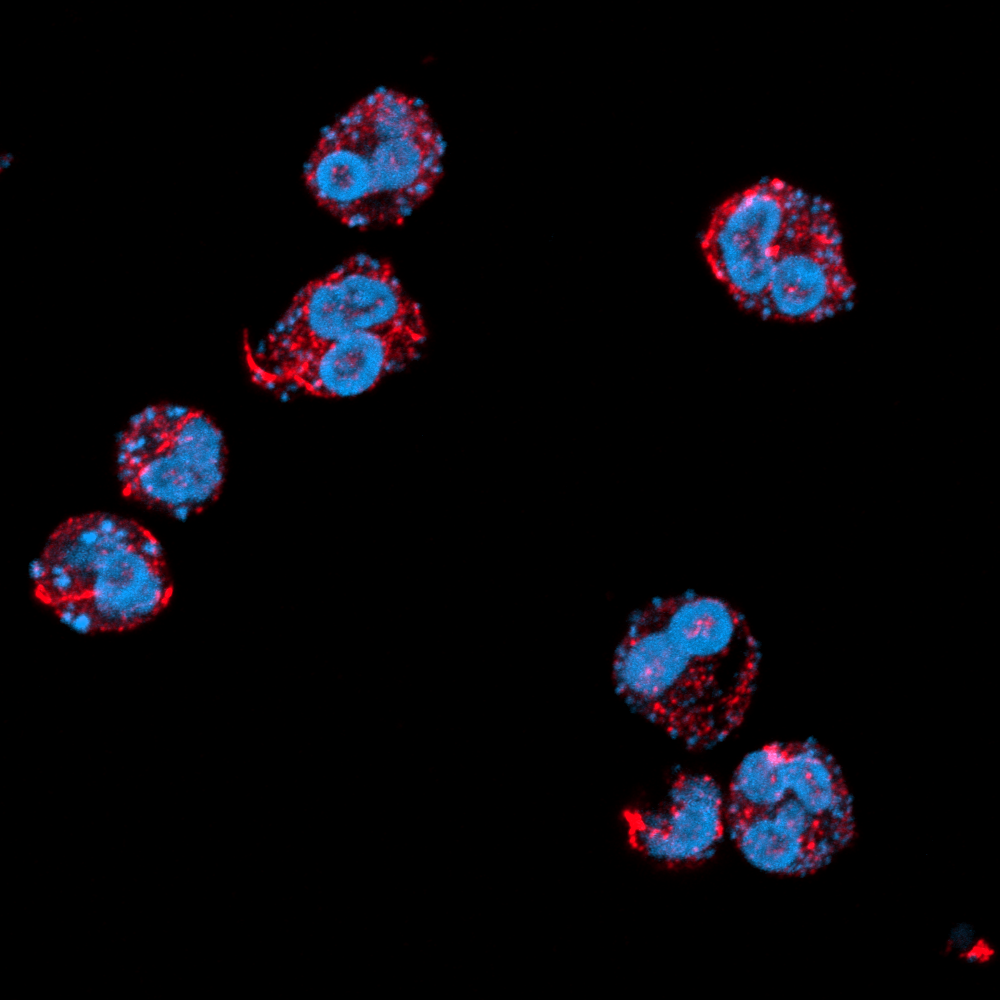

Supplement: Supplementary file 9 — EV Figures Source Data [file 44319_2024_150_MOESM9_ESM.zip › Figure EV3/Fig S3A/image FB-175/pad4/pma nets/MAX_Experiment-3527-Airyscan Processing-17.png]

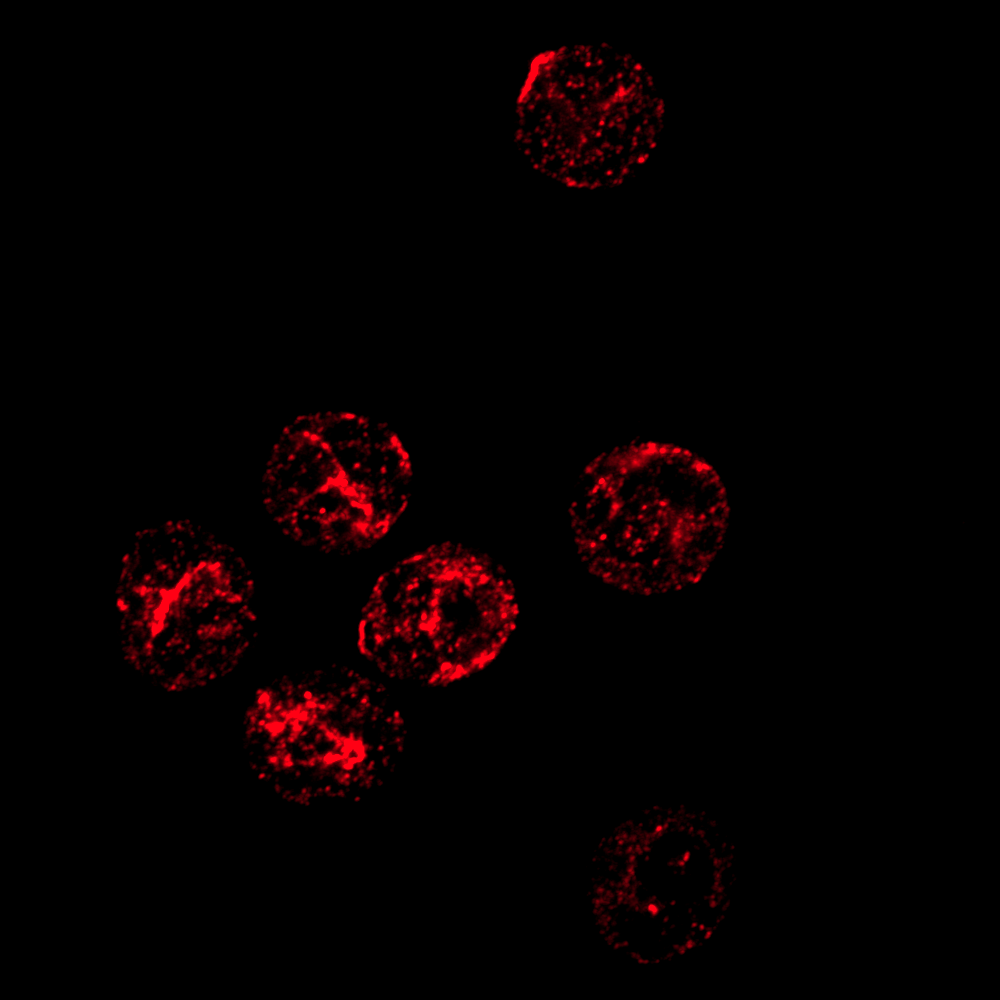

Supplement: Supplementary file 9 — EV Figures Source Data [file 44319_2024_150_MOESM9_ESM.zip › Figure EV3/Fig S3A/image FB-175/pad4/ssrna ll37/C1-MAX_Experiment-3528-Airyscan Processing-18.png]

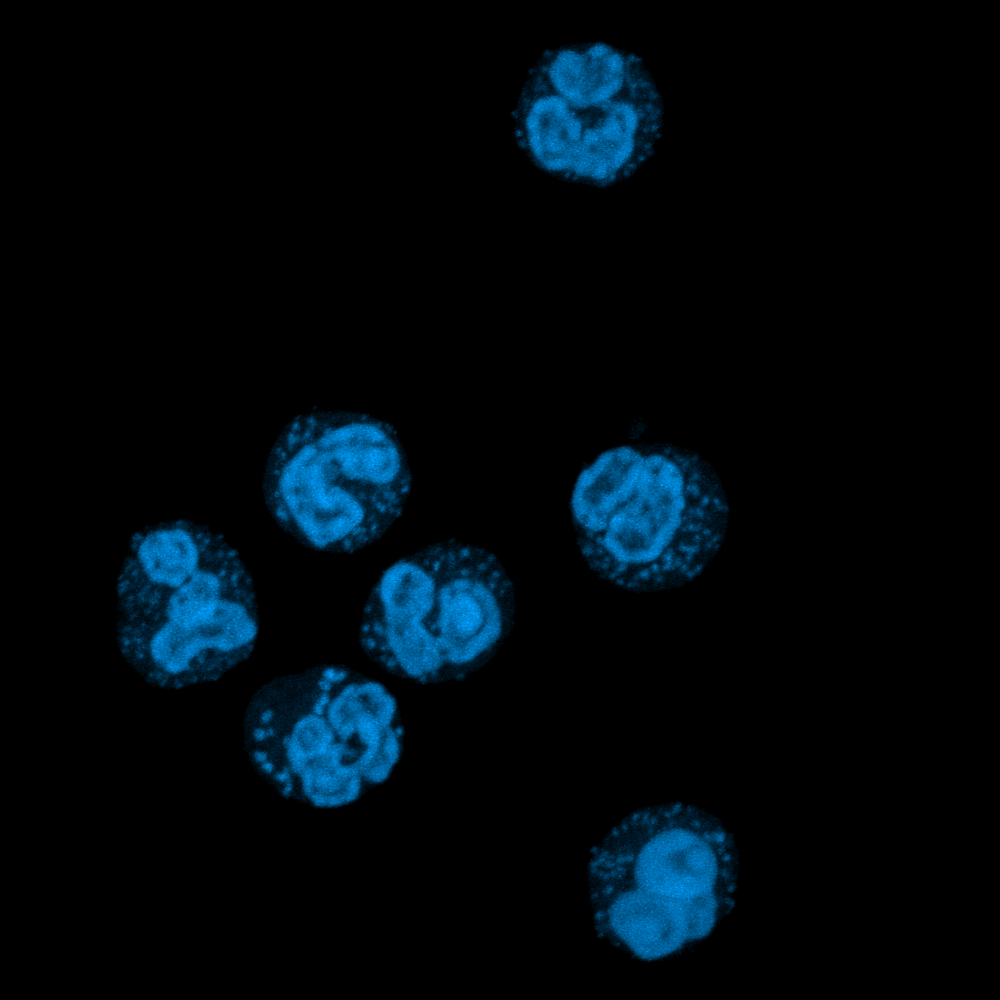

Supplement: Supplementary file 9 — EV Figures Source Data [file 44319_2024_150_MOESM9_ESM.zip › Figure EV3/Fig S3A/image FB-175/pad4/ssrna ll37/C2-MAX_Experiment-3528-Airyscan Processing-18.png]

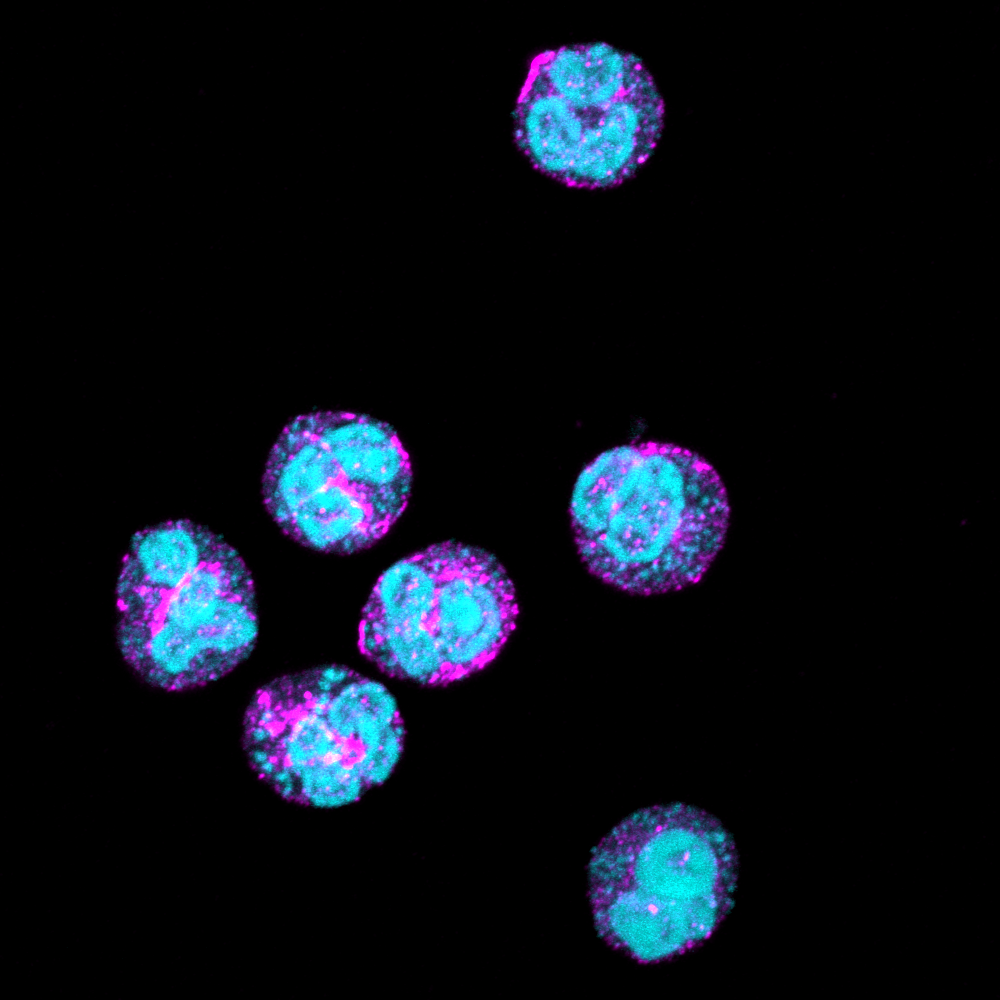

Supplement: Supplementary file 9 — EV Figures Source Data [file 44319_2024_150_MOESM9_ESM.zip › Figure EV3/Fig S3A/image FB-175/pad4/ssrna ll37/comp new.png]

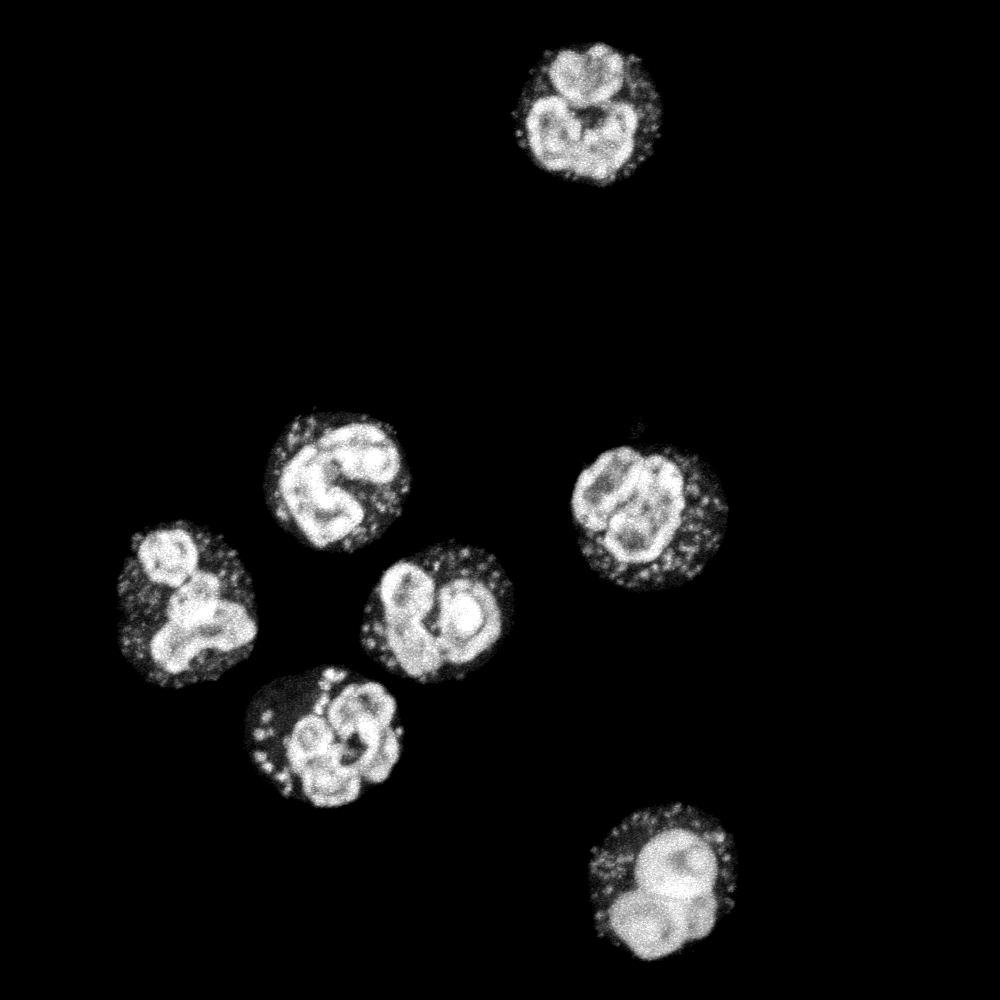

Supplement: Supplementary file 9 — EV Figures Source Data [file 44319_2024_150_MOESM9_ESM.zip › Figure EV3/Fig S3A/image FB-175/pad4/ssrna ll37/gray.png]

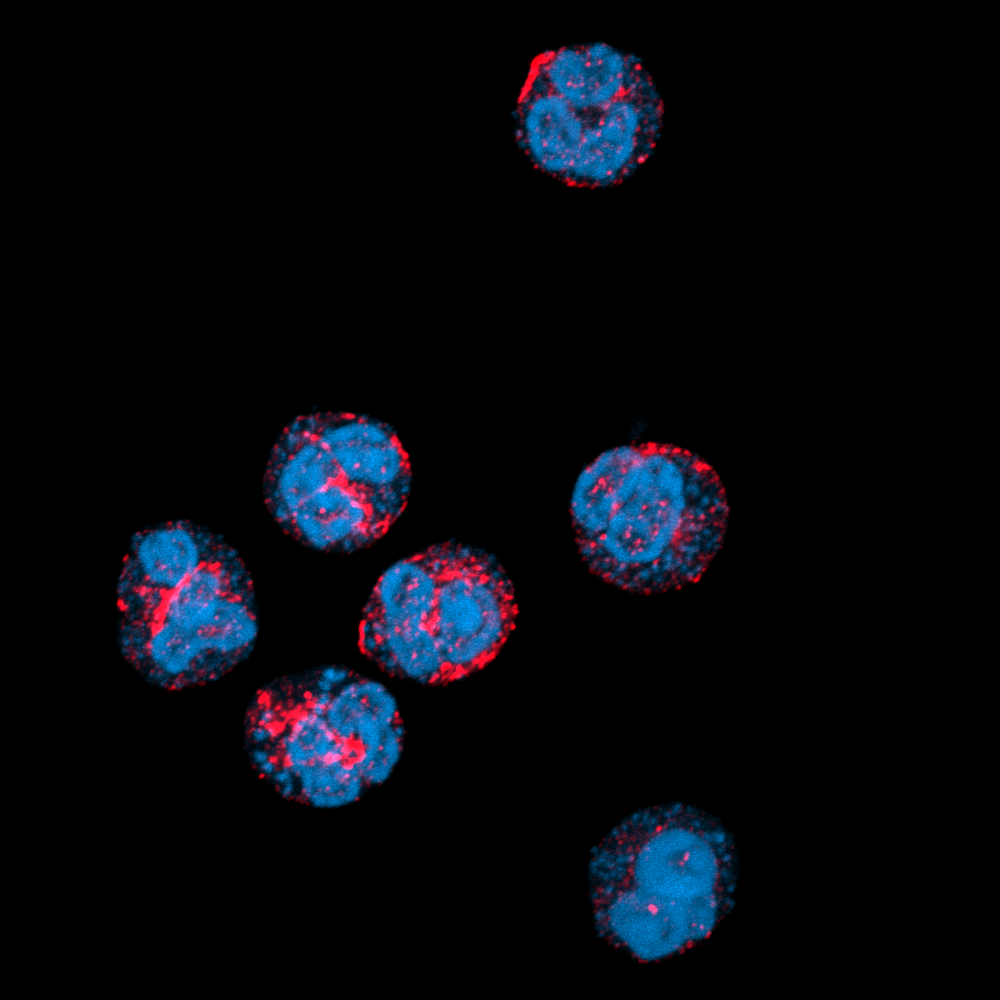

Supplement: Supplementary file 9 — EV Figures Source Data [file 44319_2024_150_MOESM9_ESM.zip › Figure EV3/Fig S3A/image FB-175/pad4/ssrna ll37/MAX_Experiment-3528-Airyscan Processing-18.png]

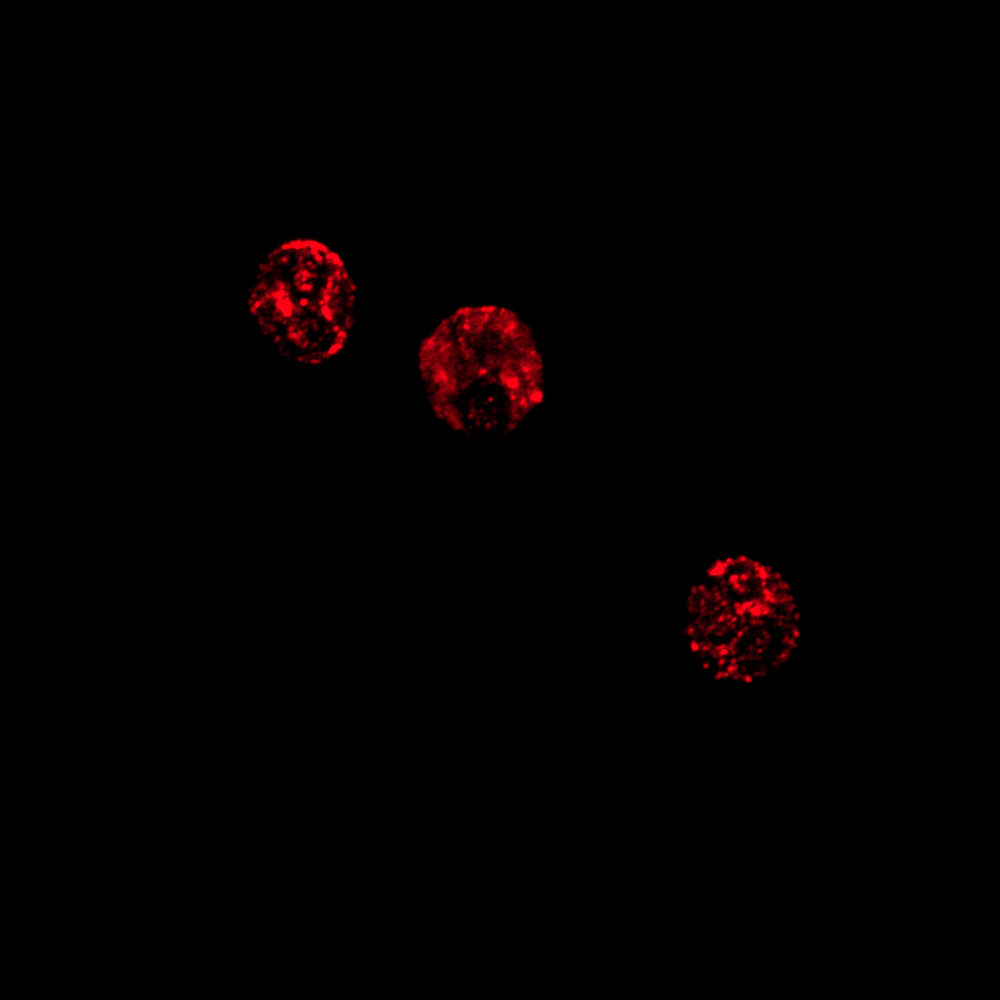

Supplement: Supplementary file 9 — EV Figures Source Data [file 44319_2024_150_MOESM9_ESM.zip › Figure EV3/Fig S3A/image FB-175/pad4/unstimulated/C1-MAX_Experiment-3524-Airyscan Processing-14.png]

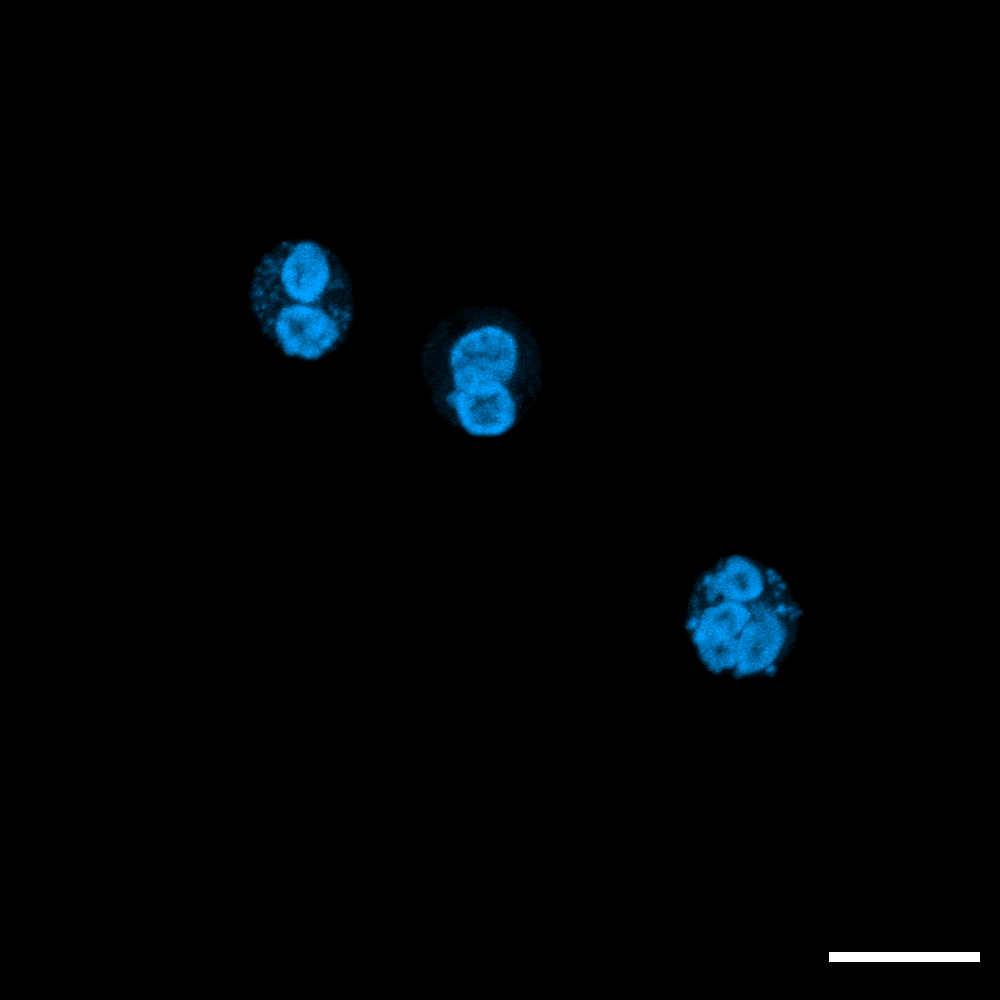

Supplement: Supplementary file 9 — EV Figures Source Data [file 44319_2024_150_MOESM9_ESM.zip › Figure EV3/Fig S3A/image FB-175/pad4/unstimulated/C2-MAX_Experiment-3524-Airyscan Processing-14.png]

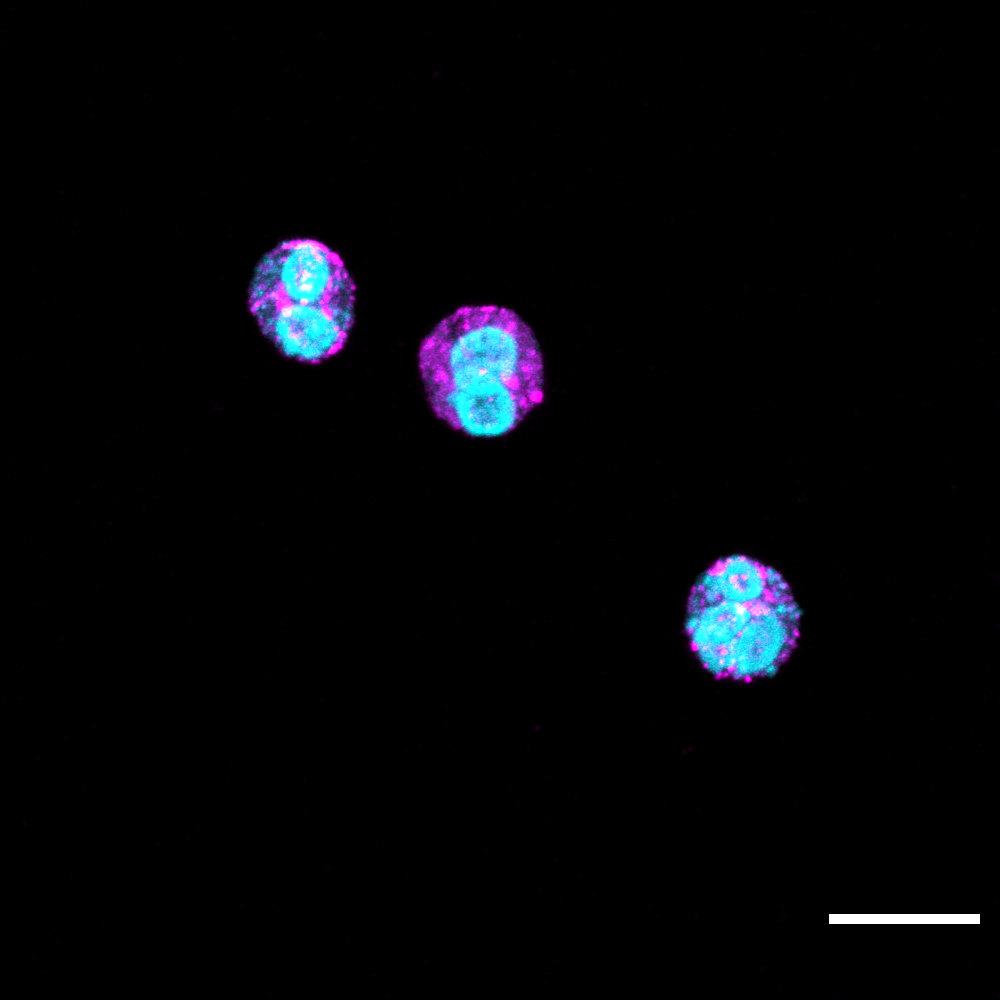

Supplement: Supplementary file 9 — EV Figures Source Data [file 44319_2024_150_MOESM9_ESM.zip › Figure EV3/Fig S3A/image FB-175/pad4/unstimulated/comp new.png]

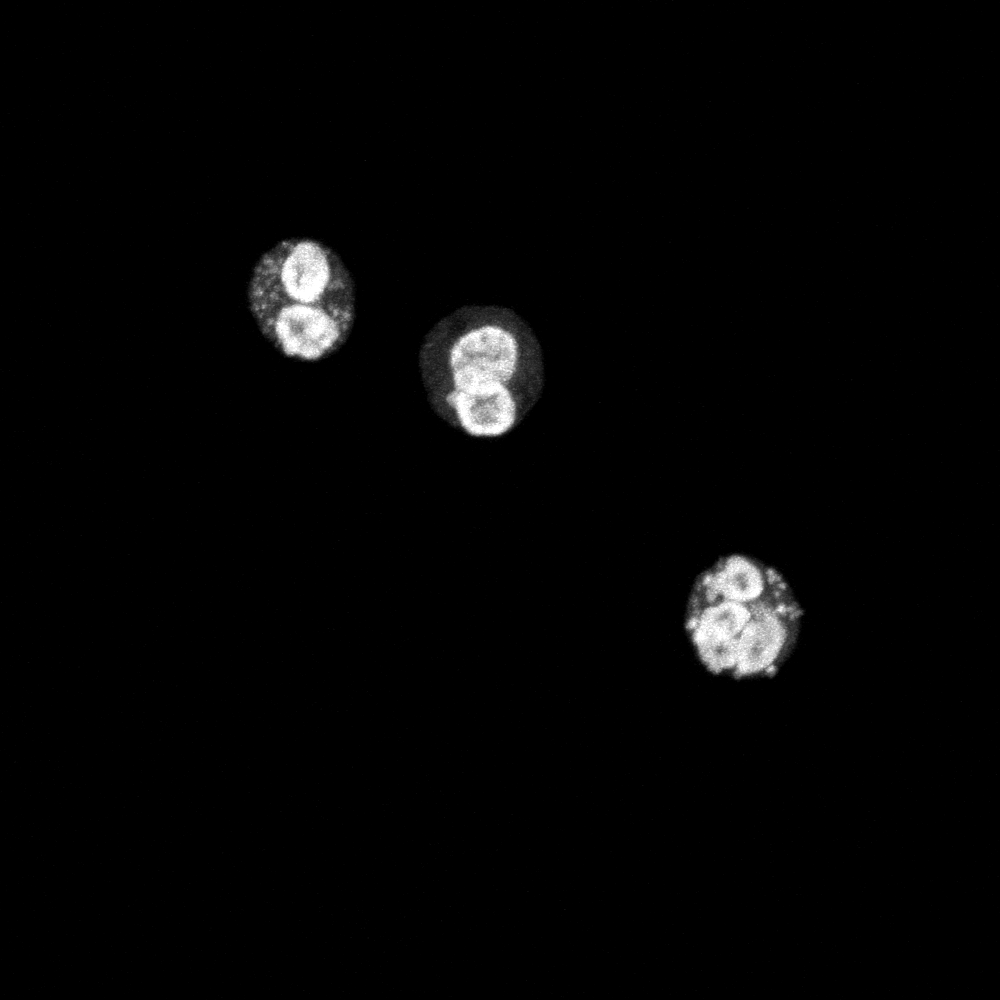

Supplement: Supplementary file 9 — EV Figures Source Data [file 44319_2024_150_MOESM9_ESM.zip › Figure EV3/Fig S3A/image FB-175/pad4/unstimulated/gray.png]

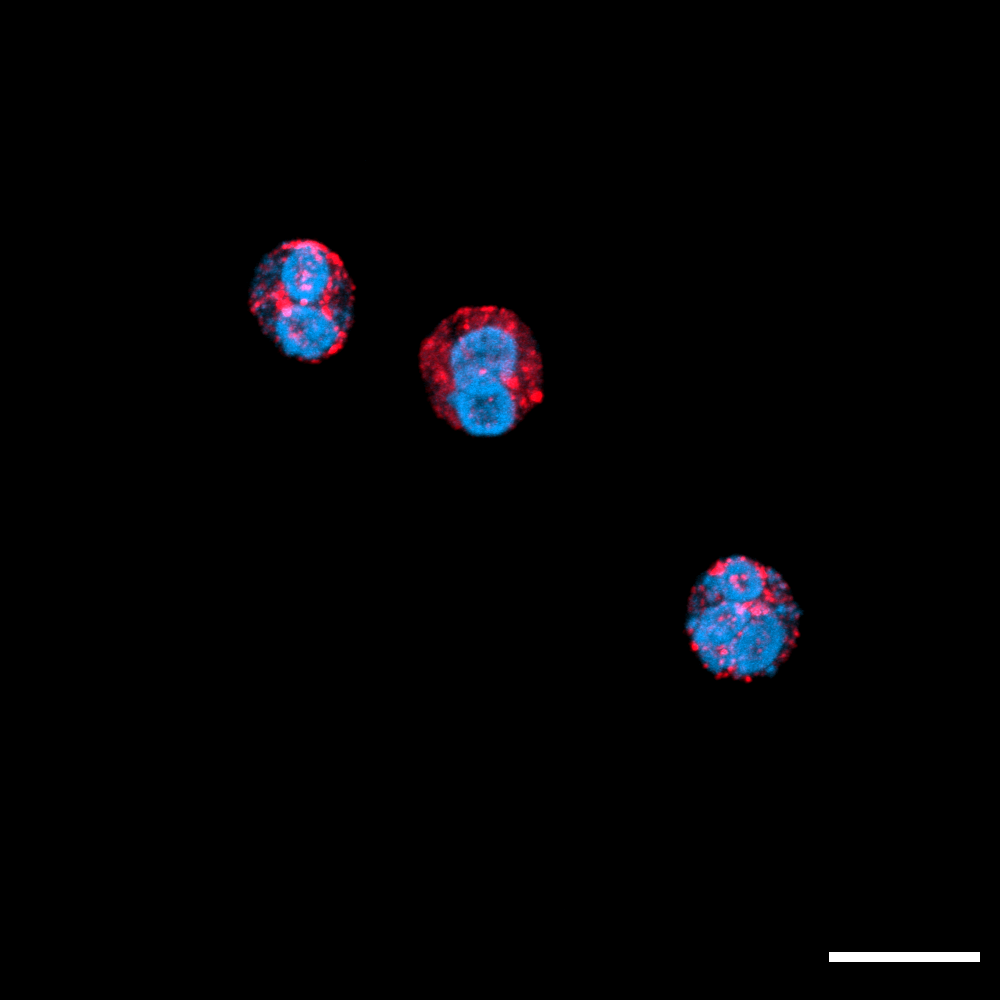

Supplement: Supplementary file 9 — EV Figures Source Data [file 44319_2024_150_MOESM9_ESM.zip › Figure EV3/Fig S3A/image FB-175/pad4/unstimulated/MAX_Experiment-3524-Airyscan Processing-14 scale bar.png]

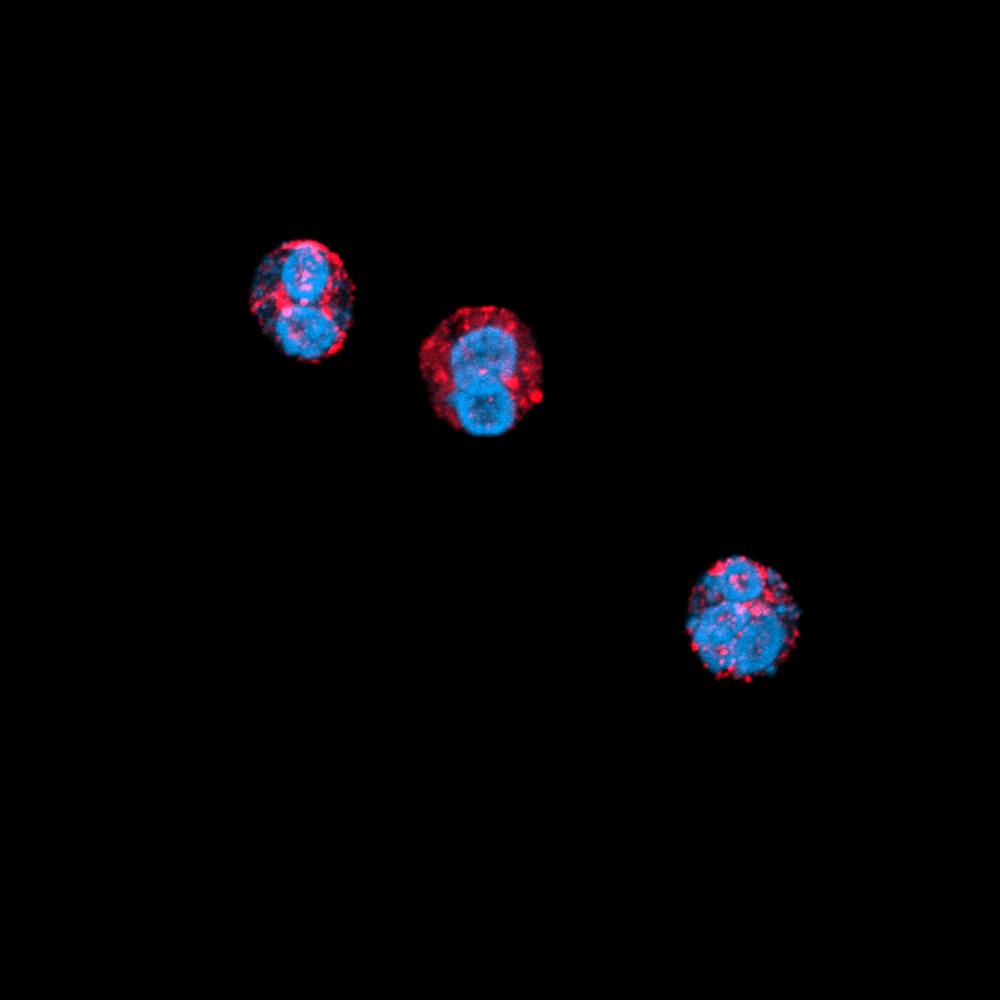

Supplement: Supplementary file 9 — EV Figures Source Data [file 44319_2024_150_MOESM9_ESM.zip › Figure EV3/Fig S3A/image FB-175/pad4/unstimulated/MAX_Experiment-3524-Airyscan Processing-14.png]
