# Supplementary material for: naRNA-LL37 composite DAMPs define sterile NETs as self-propagating drivers of inflammation
Source: EMBO Rep. 2024 May 23;25(7):10. doi: 10.1038/s44319-024-00150-5 (PMC11239898; doi:10.1038/s44319-024-00150-5)
Supplement: Supplementary file 11 — Expanded View Figures [file 44319_2024_150_MOESM11_ESM.pdf]

Expanded View Figures

Figure EV1. Controls, IF microscopy of murine bone marrow-derived neutrophils and of stem cell-derived PMNs, 3D reconstruction of naRNA in NETs, controls of electron microscopy and analysis of isolated naRNA.

(**A**) Confocal microscopy of unstimulated or PMA (600 nM) stimulated primary human PMNs after 3 h and stained for naRNA (anti-rRNA Y10b, magenta) and DNA (Hoechst 33342, white). Complete staining and secondary antibody controls only ($n = 3$ biological replicates, representative images, scale bar: 10 μm, white arrows indicate NETs). (**B**) Confocal microscopy of unstimulated primary human PMNs (control to Fig. 1A) after 3 h and stained for naRNA (anti-rRNA Y10b, magenta) and DNA (Hoechst 33342, white, $n = 3$ biological replicates, representative images, scale bar: 10 μm, white arrows indicate NETs). (**C**) Confocal microscopy of primary murine BM-PMNs of C57BL/6 WT mice stimulated as indicated for 16 h and stained as in (**B**) ($n = 3$ biological replicates, representative images, scale bar: 10 μm, white arrows indicate NETs). (**D**) Confocal microscopy of primary human stem cells differentiated in vitro with/without 100 μM 5-ethynyluridine (5-EU), click-labeled with a fluorescent dye (yellow, total RNA), and stained for naRNA (anti-rRNA Y10b, magenta) and DNA (Hoechst 33342, white, $n = 3$ biological replicates, representative images, scale bar: 10 μm, 2 μm in cropped image, white arrows indicate NETs). (**E**) Brightfield microscopy analysis of control cytospun of primary human stem cell-derived PMNs shown in (**A**) ($n = 3$ biological replicates, representative images, scale bar: 10 μm). (**F**) FACS analysis of cells shown in (**D**) and (**E**) ($n = 3$ biological replicates, representative data of one biological replicate shown). (**G**) As in (**B**) showing 3D image reconstruction of NETs from z-stacks created with ZenBlue3 ($n = 3$ biological replicates, representative images, scale bar as indicated). (**H**) Scanning electron microscopy of PMA-treated human primary PMNs showing only secondary antibody staining (no primary antibody) control of Fig. 1C ($n = 1$ biological replicate, representative data; the image on the right is a composite image with signals from secondary electron and backscattered electron detectors for topography and additional material information, respectively). (**I**) Agilent TapeStation quantification of naRNA isolated from mock or PMA NETs (from $n = 4$–6 biological replicates, combined data, each dot represents one biological replicate). Data information: In (**I**), data are presented as mean + SD. $^{*}p < 0.05$ according to Mann–Whitney test. Please note that the panel shown in B also appears in Fig. EV2C as these two experiments were carried out simultaneously or were part of the same experiment, and hence control conditions (e.g., unstimulated) are identical. Source data are available online for this figure.

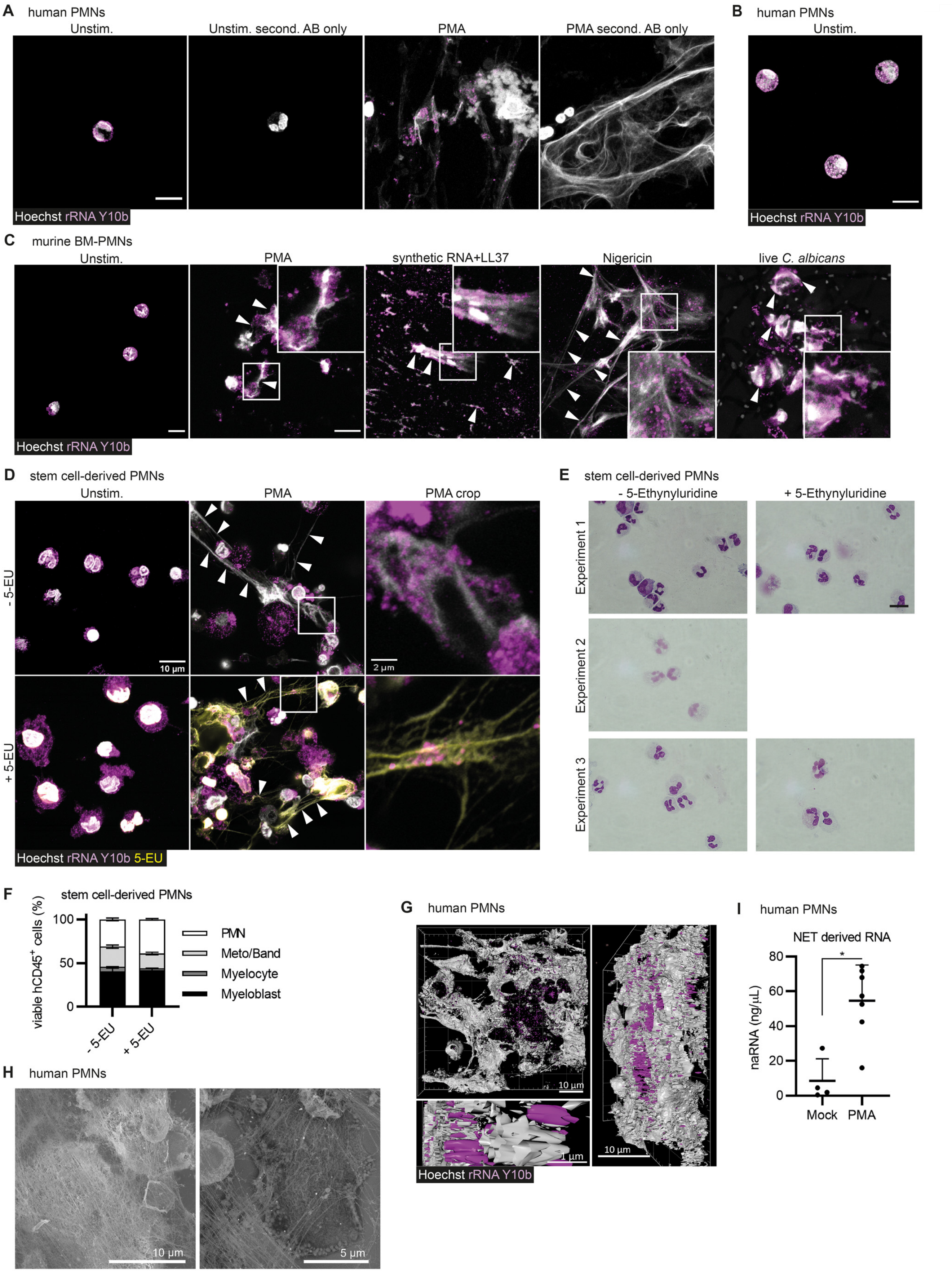
A human PMNs
Unstim.
Unstim. second. AB only
PMA
PMA second. AB only
Hoechst rRNA Y10b
B human PMNs
Unstim.
Hoechst rRNA Y10b
C murine BM-PMNs
Unstim.
PMA
synthetic RNA+LL37
Nigericin
live C. albicans
Hoechst rRNA Y10b
D stem cell-derived PMNs
Unstim.
PMA
PMA crop
- 5-EU
+ 5-EU
10 µm
2 µm
Hoechst rRNA Y10b 5-EU
E stem cell-derived PMNs
- 5-Ethynyluridine
+ 5-Ethynyluridine
Experiment 1
Experiment 2
Experiment 3
F stem cell-derived PMNs
viable hCD45+ cells (%)
100
50
0
-5-EU
+5-EU
PMN
Meto/Band
Myelocyte
Myeloblast
G human PMNs
10 µm
1 µm
10 µm
Hoechst rRNA Y10b
H human PMNs
10 µm
5 µm
I human PMNs
NET derived RNA
naRNA (ng/µL)
80
60
40
20
0
*
Mock
PMA

 © The Author(s)

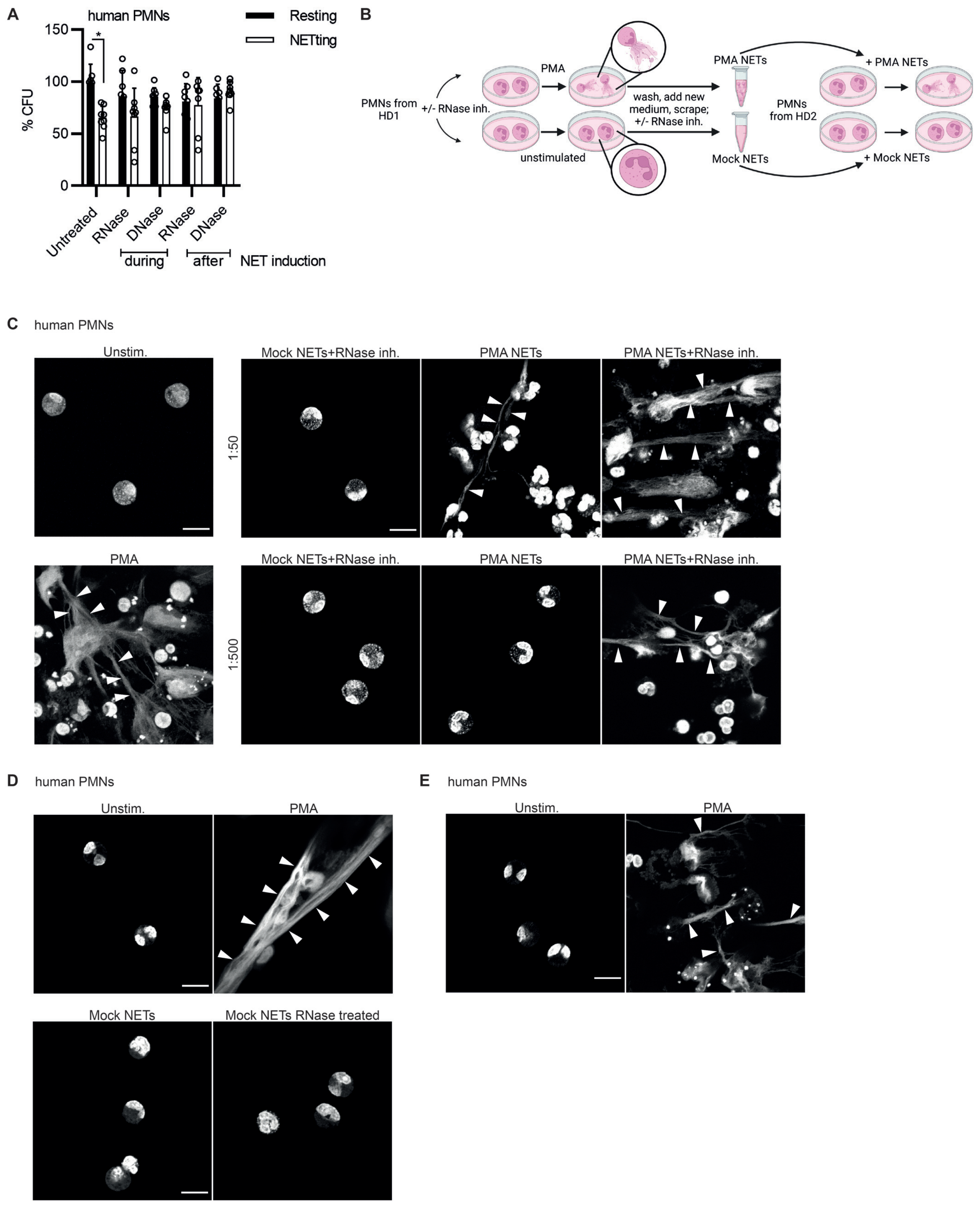
A
human PMNs
Resting
NETting
150
100
50
0
% CFU
*
Untreated
RNase
DNase
RNase
DNase
during
after
NET induction
B
PMNs from HD1
+/- RNase inh.
PMA
unstimulated
wash, add new medium, scrape; +/- RNase inh.
PMA NETs
Mock NETs
PMNs from HD2
+ PMA NETs
+ Mock NETs
C
human PMNs
Unstim.
PMA
1:50
1:500
Mock NETs+RNase inh.
PMA NETs
PMA NETs+RNase inh.
D
human PMNs
Unstim.
PMA
Mock NETs
Mock NETs RNase treated
E
human PMNs
Unstim.
PMA

Figure EV2. Antibacterial effect of NETs on live *S. aureus*, isolation of NET content and controls of IF microscopy.

(**A**) Extracellular bactericidal activity of human PMNs/NETs after infection with *S. aureus* and treatment with RNase A and DNase I during or after formation of PMA-induced NETs ($n = 8$ biological replicates, combined data). (**B**) Workflow for NET content preparation from one donor and transfer to naive human primary PMNs from a second donor (created with BioRender.com). (**C**) Confocal microscopy of primary human PMNs stimulated for 3 h with PMA (600 nM) or NET content (harvested with/without RNase inhibitor and diluted 1:50 or 1:500), and then stained for NETs/DNA (Hoechst 33342, $n = 9$ biological replicates, representative images, scale bar: 10 μm, white arrows indicate NETs). (**F**) As in (**C**) but with/without pre-digestion of NET content with RNase A (controls to Fig. 1F, $n = 3$ biological replicates, representative images, scale bar: 10 μm, white arrows indicate NETs). (**E**) Confocal microscopy of unstimulated or PMA-stimulated (3 h) primary human PMNs (controls to Fig. 1H), subsequently stained for DNA (Hoechst 33342, white, $n = 9$ biological replicates, representative images, scale bar: 10 μm, white arrows indicate NETs). Data information: In (**A**), data are presented as mean + SD. $*p < 0.05$ according to one-way ANOVA. Please note that the panel shown in (**C**) also appears in Fig. EV1B as these two experiments were carried out simultaneously or were part of the same experiment, and hence control conditions (e.g., unstimulated) are identical. Source data are available online for this figure.

 © The Author(s)

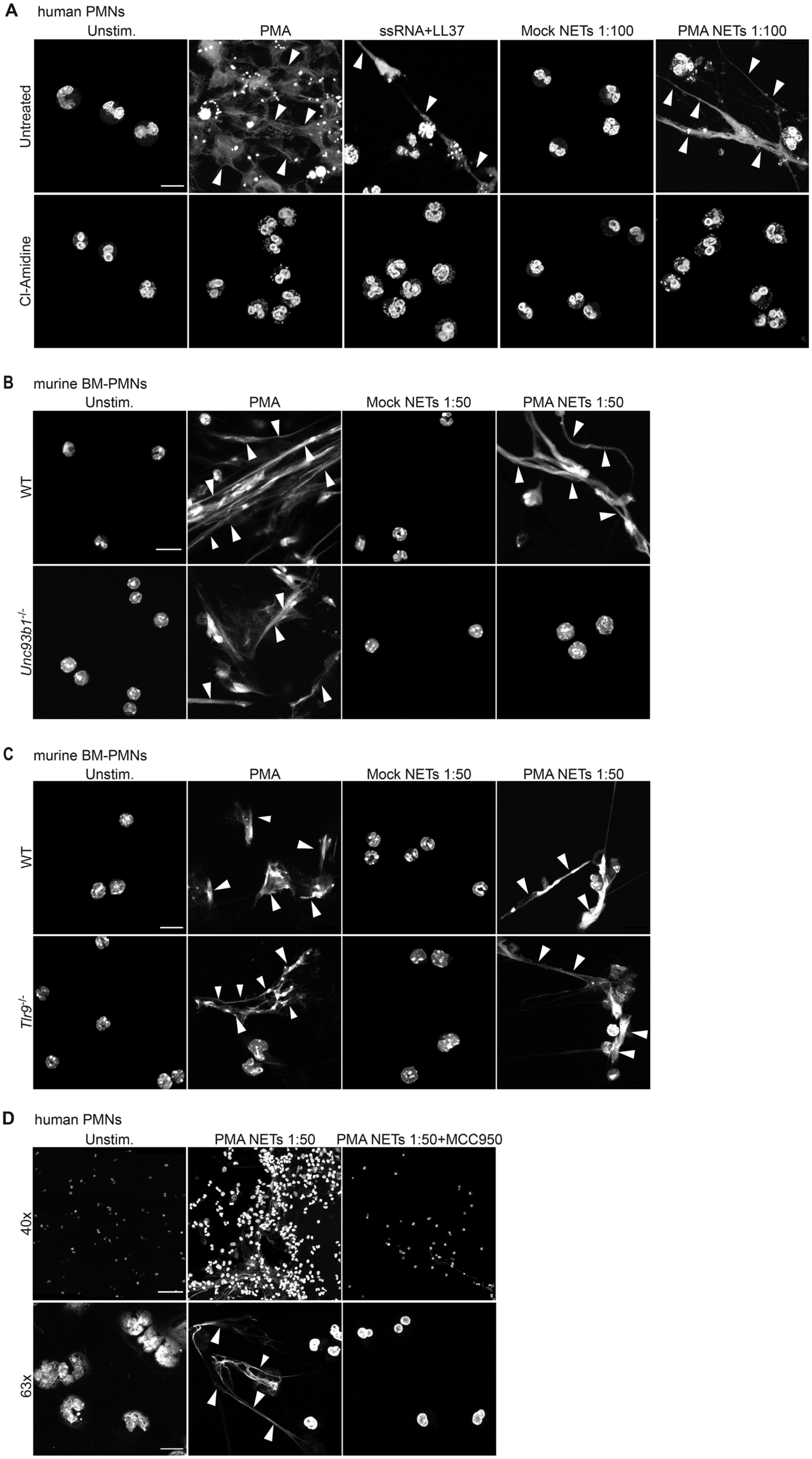
A
human PMNs
Unstim.
PMA
ssRNA+LL37
Mock NETs 1:100
PMA NETs 1:100
Untreated
Cl-Amidine
B
murine BM-PMNs
Unstim.
PMA
Mock NETs 1:50
PMA NETs 1:50
WT
Unc93b1-/-
C
murine BM-PMNs
Unstim.
PMA
Mock NETs 1:50
PMA NETs 1:50
WT
Tlr9-/-
D
human PMNs
Unstim.
PMA NETs 1:50
PMA NETs 1:50+MCC950
40x
63x

Figure EV3. Inhibition of PAD4 in human PMNs during NET formation assay, *Unc93b1*$^{-/-}$ and *Tlr9*$^{-/-}$ BM-PMN stimulation with human NETs and inhibition of NLRP3 in human PMNs during NET formation assay.

(**A**) Confocal microscopy of primary human PMNs, stimulated for 3 h in the presence or absence of the pan-PAD-inhibitor Cl-amidine (200 µM), and subsequently stained for DNA (Hoechst 33342, white) ($n = 3$ biological replicates, representative images; scale bar 10 µm, white arrows indicate NETs). (**B**) Confocal microscopy of primary C57BL/6 WT or *Unc93b1*$^{-/-}$ murine BM-PMNs stimulated for 16 h as indicated in (**A**) ($n = 3$ biological replicates WT, $n = 1$ *Unc93b1*$^{-/-}$ biological replicate, representative images, scale bar: 10 µm, white arrows indicate NETs). (**C**) Confocal microscopy of primary C57BL/6 WT or *Tlr9*$^{-/-}$ murine BM-PMNs stimulated for 16 h as indicated in (**A**) ($n = 3$ biological replicates, representative images, scale bar: 10 µm, white arrows indicate NETs). (**D**) As in (**A**) in the presence or absence of the NLRP3-inhibitor MCC950 (10 µM, $n = 3$ biological replicates, representative images; scale bar 50 µm for 40× and 10 µm for 63×, white arrows indicate NETs). Data information: Please note that selected panels in (**A**), (**B**), and (**D**) also appear in Fig. E2B, (**D**) and (**H**), respectively, as these two experiments were carried out simultaneously or were part of the same experiment, and hence control conditions (e.g., unstimulated) are identical. Source data are available online for this figure.

 © The Author(s)

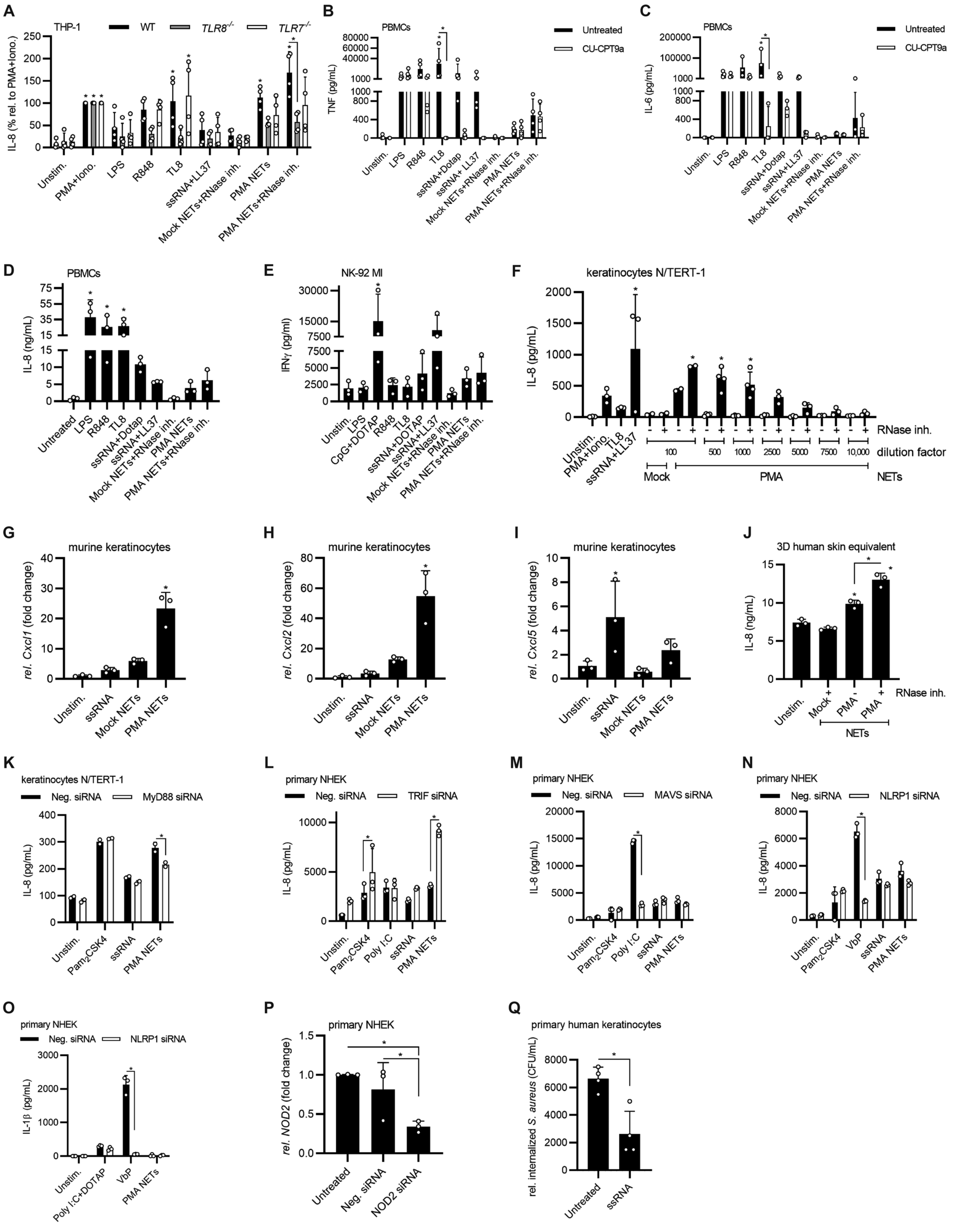
A
THP-1
WT
TLR8-/-
TLR7-/-
IL-8 (% rel. to PMA+Iono.)
Unstim.
PMA+Iono.
LPS
R848
TL8
ssRNA+LL37
Mock NETs+RNase inh.
PMA NETs
PMA NETs+RNase inh.
B
PBMCs
Untreated
CU-CPT9a
TNF (pg/mL)
Unstim.
LPS
R848
TL8
ssRNA+Dotap
ssRNA+ LL37
Mock NETs+RNase inh.
PMA NETs
PMA NETs+RNase inh.
C
PBMCs
Untreated
CU-CPT9a
IL-6 (pg/mL)
D
PBMCs
IL-8 (ng/mL)
Untreated
LPS
R848
TL8
ssRNA+Dotap
ssRNA+LL37
Mock NETs+RNase inh.
PMA NETs
PMA NETs+RNase inh.
E
NK-92 MI
IFNγ (pg/ml)
Unstim.
LPS
CpG+DOTAP
R848
TL8
ssRNA+DOTAP
ssRNA+LL37
Mock NETs+RNase inh.
PMA NETs
PMA NETs+RNase inh.
F
keratinocytes N/TERT-1
IL-8 (pg/mL)
Unstim.
PMA+Iono.
TL8
ssRNA+LL37
RNase inh.
100 500 1000 2500 5000 7500 10,000
dilution factor
Mock
PMA
NETs
G
murine keratinocytes
rel. Cxcl1 (fold change)
Unstim.
ssRNA
Mock NETs
PMA NETs
H
murine keratinocytes
rel. Cxcl2 (fold change)
I
murine keratinocytes
rel. Cxcl5 (fold change)
J
3D human skin equivalent
IL-8 (ng/mL)
Unstim.
Mock
PMA
PMA
RNase inh.
NETs
K
keratinocytes N/TERT-1
Neg. siRNA
MyD88 siRNA
IL-8 (pg/mL)
Unstim.
Pam2CSK4
ssRNA
PMA NETs
L
primary NHEK
Neg. siRNA
TRIF siRNA
IL-8 (pg/mL)
Unstim.
Pam2CSK4
Poly I:C
ssRNA
PMA NETs
M
primary NHEK
Neg. siRNA
MAVS siRNA
IL-8 (pg/mL)
N
primary NHEK
Neg. siRNA
NLRP1 siRNA
IL-8 (pg/mL)
Unstim.
Pam2CSK4
VbP
ssRNA
PMA NETs
O
primary NHEK
Neg. siRNA
NLRP1 siRNA
IL-1β (pg/mL)
Unstim.
Poly I:C+DOTAP
VbP
PMA NETs
P
primary NHEK
rel. NOD2 (fold change)
Untreated
Neg. siRNA
NOD2 siRNA
Q
primary human keratinocytes
rel. internalized S. aureus (CFU/mL)
Untreated
ssRNA

Figure EV4. Immune responses of PBMCs and human and murine keratinocytes to NETs.

(**A**) Levels of IL-8, as measured by triplicate ELISA in WT, *TLR8*$^{-/-}$, and *TLR7*$^{-/-}$ THP-1 cells stimulated as indicated for 18 h. Values were normalized to PMA+ionomycin control (n = 4 biological replicates, combined data, each dot represents one biological replicate). (**B–D**) Levels of TNF (**B**, $n = 4$ biological replicates), IL-6 (**C**, $n = 3$ biological replicates), and IL-8 (**D**, $n = 3$ biological replicates), as measured by triplicate ELISA in primary human PBMCs stimulated as indicated with/without CU-CPT9a for 24 h (combined data, each dot represents one biological replicate). (**E**) Levels of IFN-γ, as measured by triplicate upon release from NK-92 MI cells stimulated as indicated for 24 h ($n = 3$ biological replicates, combined data, each dot represents one biological replicate). (**F**) Levels of IL-8, as measured by triplicate ELISA upon release from N/TERT-1 keratinocytes stimulated as indicated for 24 h ($n = 3$ biological replicates, combined data, each dot represents one biological replicate). (**G–I**) Fold changes in the expression of (**G**) *Cxcl1*, (**H**) *Cxcl2,* or (**I**) *Cxcl5* in murine C57BL/6 WT keratinocytes stimulated as indicated for 1 h. qPCR was performed in triplicate and fold changes were calculated relative to untreated control ($n = 3$ biological replicates, combined data, each dot represents one biological replicate). (**J**) Levels of IL-8 as measured by triplicate ELISA upon release from NHEK 3D human skin equivalent constructs stimulated as indicated for 24 h ($n = 3$ biological replicates, representative of one biological replicate is shown, each dot represents one technical replicate). (**K**) Levels of IL-8, as measured by triplicate ELISA in N/TERT-1 keratinocytes stimulated as indicated with/without MyD88 siRNA knockdown for 24 h ($n = 1$ biological replicate, each dot represents one technical replicate). (**L–O**) Levels of IL-8 (**L–N**) or IL-1β (**O**), as measured by ELISA upon release from primary human normal keratinocytes (NHEK) stimulated as indicated with/without TRIF (**L**), MAVS (**M**) or NLRP1 (**N**, **O**) siRNA knockdown for 24 h ($n = 1$ biological replicate, each dot represents one technical replicate). (**P**) Fold changes in the expression of *NOD2* in primary human normal keratinocytes (NHEK) after NOD2 siRNA knockdown. qPCR was performed in triplicate and fold changes were calculated relative to unstimulated control (control to Fig. 3G, $n = 3$ biological replicates, combined data, each dot represents one biological replicate). (**Q**) In vitro colonization assay (CFU) of primary human keratinocytes primed as indicated for 24 h and subsequently exposed to *S. aureus* for 1 h ($n = 4$ biological replicates, combined data, each dot represents one technical replicate). Data information: In (**A–Q**), data are presented as mean + SD. In (**A–P**), $^*p < 0.05$ according to one-way ANOVA. In (**Q**), $^*p < 0.05$ according to Student's t-test. Source data are available online for this figure.

 © The Author(s)

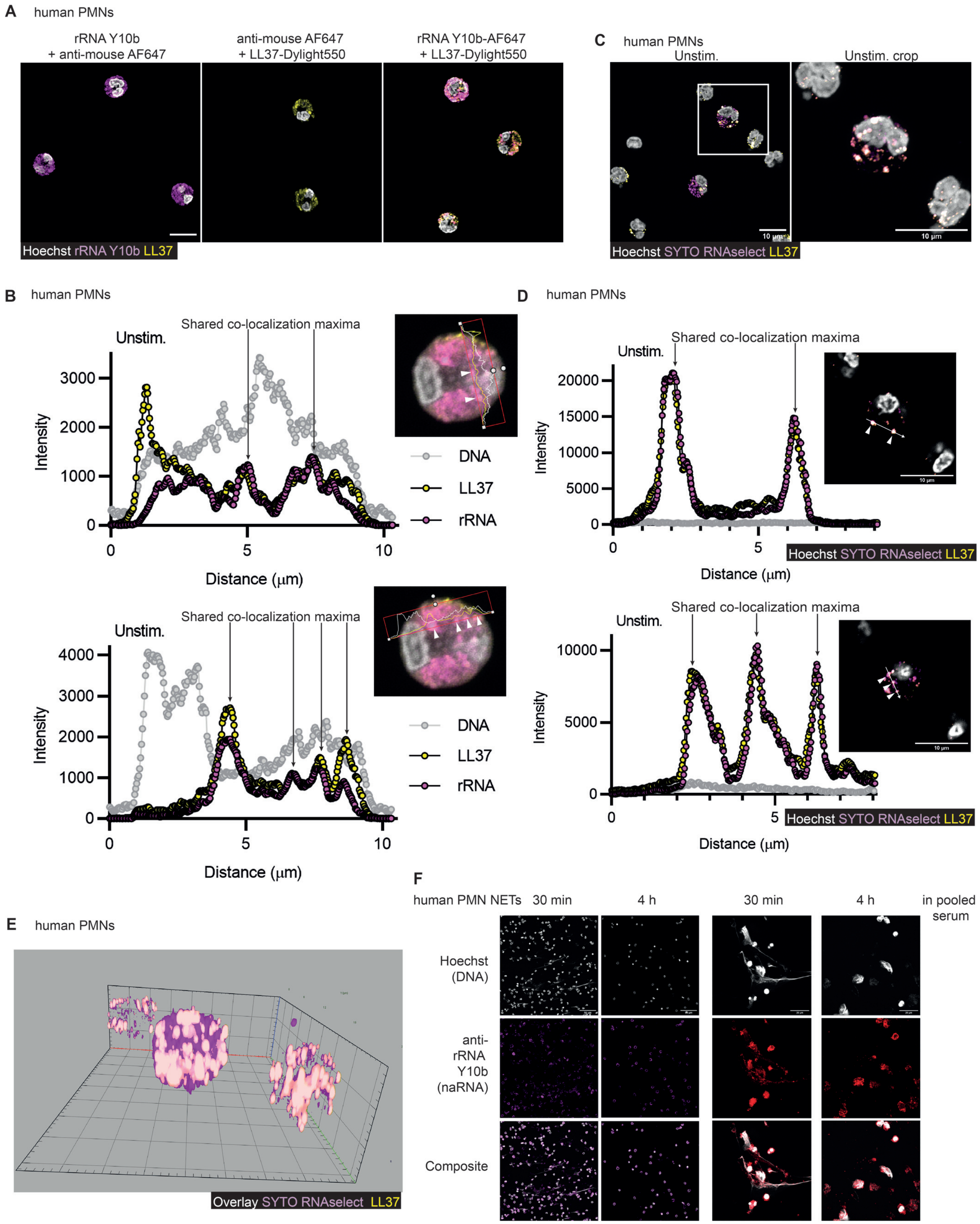
A human PMNs
rRNA Y10b + anti-mouse AF647
anti-mouse AF647 + LL37-Dylight550
rRNA Y10b-AF647 + LL37-Dylight550
Hoechst rRNA Y10b LL37
C human PMNs
Unstim.
Unstim. crop
10 µm
Hoechst SYTO RNAselect LL37
B human PMNs
Unstim.
Shared co-localization maxima
Intensity
Distance (µm)
DNA
LL37
rRNA
D human PMNs
Unstim.
Shared co-localization maxima
Intensity
Distance (µm)
Hoechst SYTO RNAselect LL37
E human PMNs
Overlay SYTO RNAselect LL37
F
human PMN NETs
30 min
4 h
in pooled serum
Hoechst (DNA)
anti-rRNA Y10b (naRNA)
Composite

Figure EV5. Pre-association of naRNA and LL37 in resting and NET-releasing healthy donor neutrophils and graphical abstract.

(**A**) Confocal microscopy of primary human PMNs left untreated and stained for RNA only (mouse anti-human rRNA Y10b + anti-mouse AF647, magenta), secondary antibody control for RNA and staining for LL37 (anti-mouse AF647 (magenta) + rabbit anti-human LL37-Dylight550 (yellow)) or counterstaining for rRNA Y10b and LL37 (mouse anti-human rRNA Y10b-AF647 (magenta) + rabbit anti-human LL37-Dylight550 (yellow)) and DNA (Hoechst 33342, white, $n = 3$ biological replicates, representative images, scale bar 10 μm). Controls for Figs. 5A,C and EV5B. (**B**) Confocal microscopy with line plot analysis of primary human PMNs stimulated as indicated for 3 h and stained for naRNA (anti-rRNA Y10b, magenta), LL37 (anti-hLL37-DyLight550, yellow) and DNA (Hoechst 33342, white, $n = 3$ biological replicates, representative images). The line plot analysis of LL37, RNA, and DNA staining was performed using ZenBlue3 software. One to two different line plots from the same representative image are shown. Additional examples of images shown in Fig. 5C. (**C**) Confocal microscopy of primary human PMNs left untreated and stained for naRNA (SYTO RNAselect, magenta), LL37 (anti-hLL37-DyLight550, yellow), and DNA (Hoechst 33342, white, $n = 3$ biological replicates, representative images, scale bar 10 μm). (**D**) Line plot analysis of LL37, RNA, and DNA staining of (**A**). The analysis was performed using ZenBlue3 software. Three different line plots from the same representative image are shown (scale bar 10 μm). Areas of intensity overlap show up as white. (**E**) 3D reconstructions of z-stacks from (**A**). (**F**) Confocal microscopy of PMA-induced NETs from primary human PMNs incubated for 30 min or 4 h with human serum and stained for DNA (Hoechst 33342, white) and naRNA (anti-rRNA Y10b, magenta or red). Lower magnification (left, scale bar = 50 μm) and higher magnification (right, scale bar = 20 μm) for one presentative of $n = 2$ biological replicates shown. Data information: Please note that selected panels in (**D**) also appear in Fig. 5C and D, respectively, as these two experiments were carried out simultaneously or were part of the same experiment. Source data are available online for this figure.

 © The Author(s)
